# Safety and antiviral activity of triple combination broadly neutralizing monoclonal antibody therapy against HIV-1: a phase 1 clinical trial

**DOI:** 10.1038/s41591-022-01815-1

**Published:** 2022-05-12

**Authors:** Boris Julg, Kathryn E. Stephenson, Kshitij Wagh, Sabrina C. Tan, Rebecca Zash, Stephen Walsh, Jessica Ansel, Diane Kanjilal, Joseph Nkolola, Victoria E. K. Walker-Sperling, Jasper Ophel, Katherine Yanosick, Erica N. Borducchi, Lori Maxfield, Peter Abbink, Lauren Peter, Nicole L. Yates, Martina S. Wesley, Tom Hassell, Huub C. Gelderblom, Allen deCamp, Bryan T. Mayer, Alicia Sato, Monica W. Gerber, Elena E. Giorgi, Lucio Gama, Richard A. Koup, John R. Mascola, Ana Monczor, Sofia Lupo, Charlotte-Paige Rolle, Roberto Arduino, Edwin DeJesus, Georgia D. Tomaras, Michael S. Seaman, Bette Korber, Dan H. Barouch

**Affiliations:** 1 Ragon Institute of MGH, MIT and Harvard, Cambridge, MA USA; 2 Center for Virology and Vaccine Research, Beth Israel Deaconess Medical Center, Boston, MA USA; 3 Theoretical Biology and Biophysics Group, Los Alamos National Laboratory, Los Alamos, NM USA; 4 New Mexico Consortium, Los Alamos, NM USA; 5 Duke Human Vaccine Institute, Duke University, Durham, NC USA; 6 International AIDS Vaccine Initiative, New York, NY USA; 7 Present Address: Icosavax Inc., Seattle, WA USA; 8 Statistical Center for HIV/AIDS Research and Prevention, Fred Hutchinson Cancer Research Center, Seattle, WA USA; 9 Vaccine Research Center, National Institute of Health, Bethesda, MD USA; 10 Houston AIDS Research Team, McGovern Medical School at The University of Texas Health Science Center at Houston, Houston, TX USA; 11 Orlando Immunology Center, Orlando, FL USA

**Keywords:** Antibodies, Retrovirus, HIV infections

## Abstract

HIV-1 therapy with single or dual broadly neutralizing antibodies (bNAbs) has shown viral escape, indicating that at least a triple bNAb therapy may be needed for robust suppression of viremia. We performed a two-part study consisting of a single-center, randomized, double-blind, dose-escalation, placebo-controlled first-in-human trial of the HIV-1 V2-glycan-specific antibody PGDM1400 alone or in combination with the V3-glycan-specific antibody PGT121 in 24 adults without HIV in part 1, as well as a multi-center, open-label trial of the combination of PGDM1400, PGT121 and the CD4-binding-site antibody VRC07-523LS in five viremic adults living with HIV not on antiretroviral therapy (ART) in part 2 (NCT03205917). The primary endpoints were safety, tolerability and pharmacokinetics for both parts and antiviral activity among viremic adults living with HIV and not on ART for part 2 of the study. The secondary endpoints were changes in CD4^+^ T cell counts and development of HIV-1 sequence variations associated with PGDM1400, PGT121 and VRC07-523LS resistance in part 2. Intravenously administered PGDM1400 was safe and well-tolerated at doses up to 30 mg kg^−1^ and when given in combination with PGT121 and VRC07-523LS. A single intravenous infusion of 20 mg kg^−1^ of each of the three antibodies reduced plasma HIV RNA levels in viremic individuals by a maximum mean of 2.04 log_10_ copies per ml; however, viral rebound occurred in all participants within a median of 20 days after nadir. Rebound viruses demonstrated partial to complete resistance to PGDM1400 and PGT121 in vitro, whereas susceptibility to VRC07-523LS was preserved. Viral rebound occurred despite mean VRC07-523LS serum concentrations of 93 µg ml^−1^. The trial met the pre-specified endpoints. Our data suggest that future bNAb combinations likely need to achieve broad antiviral activity, while also maintaining high serum concentrations, to mediate viral control.

## Abstract (web summary)

A combination of three monoclonal antibodies transiently reduced viremia in people living with HIV-1 and not on antiretroviral therapy, but it did not prevent viral rebound. Further studies are needed to determine if this approach can be optimized.

## Main

HIV-1-specific bNAbs targeting multiple epitope regions of the HIV-1 envelope trimer (Env) have demonstrated the ability to robustly reduce plasma viremia in people living with HIV not on ART as well as to modestly delay viral rebound in individuals during an analytical antiretroviral treatment interruption (ATI)^1–8^. Rapid selection of neutralization-resistant viral variants resulting in therapeutic failure has been observed in all referenced studies, and it has become evident that bNAb monotherapy is insufficient for viral control due to the frequent presence of pre-existing escape mutations in the substantially diverse within-host HIV quasispecies. Combination of two bNAbs with complementary epitope specificities—the CD4-binding-site (CD4bs) antibody 3BNC117 and the V3-glycan antibody 10-1074—were able to suppress viral rebound in a subset of individuals for an extended period during ATI; in contrast, viral breakthrough was observed in individuals in the presence of baseline escape or when one of the antibodies fell below the therapeutic threshold, resulting in functional monotherapy^8^.

It has, therefore, been postulated that three bNAbs targeting different epitope regions would be necessary to overcome viral variants with potentially pre-existent escape mutations and provide sufficient control of the virus to prevent development of novel resistance. Complementary viral coverage resulting in extended breadth and potency has been modeled for multiple bNAb combinations^9^, and the combination of the CD4bs antibody VRC07-523LS, the V3-glycan antibody PGT121 and the V2-apex antibody PGDM1400 has been identified to neutralize 99% of a panel of 374 cross-clade HIV-1 strains, of which 82% would be neutralized with at least two active antibodies (with 80% inhibitory concentration (IC_80_) of <10 µg ml^−1^)^9^. However, it is important to remember that such panels reflect single variants and not the complexity of within-host diversity found in natural infection.

Although both VRC07-523LS and PGT121 have demonstrated robust antiviral activity in viremic people living with HIV, PGDM1400 has not been evaluated in humans thus far. This antibody was originally identified in donor 84 of the International AIDS Vaccine Initiative (IAVI) Protocol G cohort and is exceptionally broad and potent, covering 83% of a panel of 106 cross-clade pseudoviruses at a median 50% inhibitory concentration (IC_50_) of 0.003 µg ml^−1^, being ten- to 100-fold more potent than CD4bs antibodies such as VRC01 and 3BNC117 (ref. ^10^). Indeed, PGDM1400 provided highly potent antiviral activity in non-human primate simian–human immunodeficiency virus (SHIV) SF162P3 challenge studies^11,12^. Here, we evaluated the safety, tolerability and pharmacokinetics of PGDM1400 when given intravenously, alone or in combination with PGT121 and VRC07-523LS, in adults without HIV and determined the antiviral activity of all three bNAbs in viremic adults living with HIV not on ART.

## Results

### Study population

**Extended Data Fig. 1:**
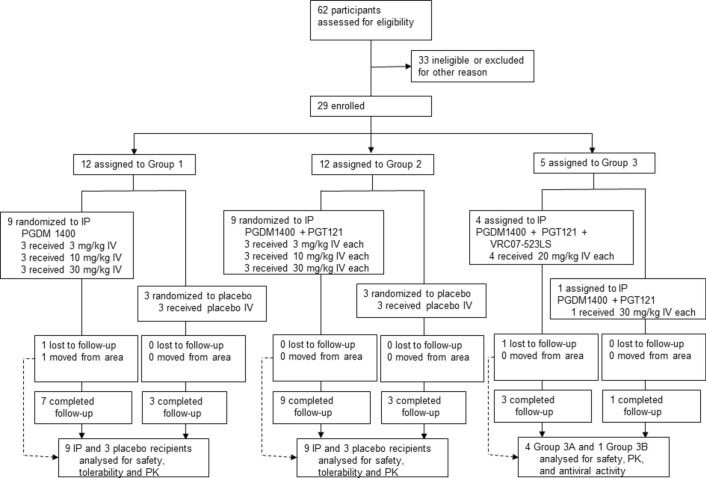
Participant enrollment and study design. A total of 62 volunteers without and with HIV were screened for study participation. In individuals without HIV, PGDM1400 was sequentially administered alone or followed by PGT121 in a single IV infusion at increasing doses of 3 mg/kg (Groups 1A and 2A), 10 mg/kg (Groups 1B and 2B), or 30 mg/kg (Groups 1C and 2C) for each bNAb. Viremic participants with HIV (Group 3) received a single IV dose of PGDM1400, PGT121 and VRC07-523LS at 20 mg/kg each (Group 3A) or a single dose of PGDM1400 and PGT121 at 30 mg/kg each (Group 3B). Of a total of 62 subjects screened, 24 participants without HIV and 5 participants with HIV were enrolled. Of the 29 participants enrolled, 26 completed the study on the planned schedule, and three terminated the study early (one Group 1B participant and one Group 3A participant were lost to follow-up and one Group 1C participant moved out of the area). However, no participants were excluded from the safety analyses as all participants had accrued follow-up time after IP administration.

To determine whether the triple combination of PGDM1400, PGT121 and VRC07-523LS is safe and active against HIV in humans, we initiated a two-part phase 1 study. Part 1 was a single-center, randomized, double-blind, dose-escalation, placebo-controlled study to evaluate three intravenous doses of PGDM1400 alone or in combination with PGT121 (3 mg kg^−1^, 10 mg kg^−1^ and 30 mg kg^−1^ per antibody, respectively) in adults without HIV. Each participant in part 1 received either PGDM1400 or placebo (group 1, *n* = 12) or PGDM1400+PGT121 or placebo (group 2, *n* = 12), randomized at a ratio of 3:1 each (Table 1 and Extended Data Fig. 1). Part 2 of the study was a multi-center, open-label trial of a single intravenous administration of 20 mg kg^−1^ each of PGDM1400, PGT121 and VRC07-523LS (group 3A, *n* = 4) or a single infusion of 30 mg kg^−1^ of PGDM1400+PGT121 (group 3B, *n* = 1) in viremic adults living with HIV not on ART (Table 1 and Extended Data Fig. 1). All participants were based in the United States and likely infected with HIV-1 subtype B. Sixty-two participants were screened, and 33 were found to be ineligible or excluded for other reasons (Extended Data Fig. 1). The first participant was enrolled on 27 November 2017, and the last participant was enrolled on 16 October 2019.

**Table 1:** Study participant demographics and baseline characteristics

	HIV-1 uninfected (*n* = 24)		HIV-1 infected (*n* = 5)
	Active (*n* = 18)	Placebo (*n* = 6)	
Gender (% male)	55.6%	50%	100%
Mean age, years (range)	27.1 (20–49)	31.5 (26–42)	38 (24–59)
Race, *n* (%)			
White	8 (44.4)	4 (66.7)	3 (60)
Black or African American	3 (16.7)	1 (16.7)	2 (40)
Asian	5 (27.8)	1 (16.7)	0
White, Slavic	1 (5.6)	0	0
Unknown	1 (5.6)	0	0
Ethnicity, *n* (%)			
Hispanic or Latino	2 (11.1)	0	2 (40)
Not Hispanic and Not Latino	16 (88.9)	6 (100)	3 (60)
CD4^+^ T cell count (day 0)			
Mean absolute (cells per μl)	N/A	N/A	505 (392–575)
Mean relative (%)	N/A	N/A	28.4 (21.8–36)
HIV-1 RNA levels (day 0)			
Geometric mean (copies per ml)	N/A	N/A	16,066 (2,770–163,130)
Mean log	N/A	N/A	4.2 (3.44–5.21)

### Safety and tolerability

Antibody serum concentrations, hematology and clinical chemistry labs were monitored closely, and plasma HIV-1 RNA levels and CD4^+^ T cell counts were assessed regularly in the participants with HIV. PGDM1400 was generally safe and well-tolerated at all doses tested, in participants without and with HIV, and when given alone or in combination with PGT121 or with PGT121 and VRC07-523LS (Supplementary Tables 1–3). Furthermore, the triple combination itself was well-tolerated, and no grade 3, grade 4 or serious adverse events and no treatment-related laboratory changes were observed during 56 days of follow-up for each group (Supplementary Tables 1–3). CD4^+^ T cell counts did not significantly change after bNAb infusions in the participants with HIV, and baseline CD4^+^ T cell counts largely remaining in the normal range (median absolute CD4^+^ T cell count was 520 cells per μl; Supplementary Table 4).

### Pharmacokinetics

**Fig. 1:**
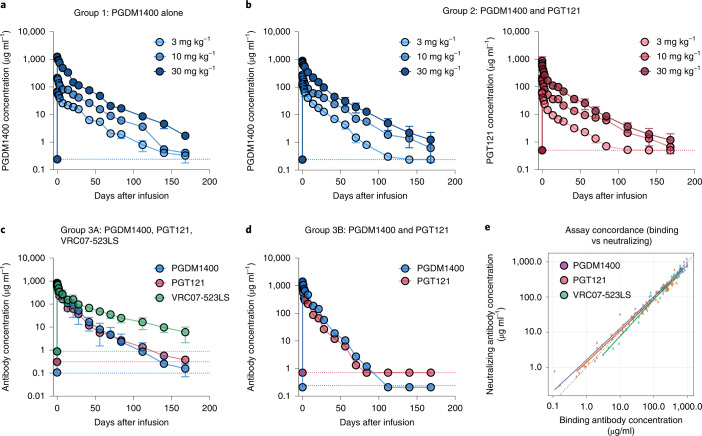
PGDM1400, PGT121 and VRC07-523LS pharmacokinetics. Serum levels of PGDM1400, PGT121 and VRC07-523LS as determined by BAMA. Mean values for each dose group with s.e.m. for PGDM1400 dosed alone (group 1, adults without HIV) (**a**), PGDM1400 and PGT121 dosed sequentially (group 2, adults without HIV) (**b**), PGDM1400, PGT121 and VRC07-523LS dosed sequentially (group 3A, adults with HIV) (**c**) and PGDM1400 and PGT121 dosed sequentially (group 3B, adults with HIV) (**d**). Dotted lines at the bottom indicate lower limit of detection of the assays, color-coded according to antibody. Each sample was measured in duplicate. Serum *t*_1/2_ of PGDM1400 is 20.8 days in adults without HIV when dosed alone, 17.4 days in adults without HIV when dosed in combination with PGT121 (*P* = 0.05) and 11 days in adults with HIV when dosed in combination. (Supplementary Tables 7 and 8). **e**, Concordance among PGDM1400, PGT121 and VRC07-523LS concentrations measured by binding and neutralizing antibody assays, shown exemplary for group 3A participants. Neutralizing assays used 6540.v4.c1, CH505TF.N334S.N160A.N280D.1 and CAP220.2.00_A8_5B to measure PGDM1400, PGT121 and VRC07-523LS concentrations, respectively. The concordance for PGDM1400, PGT121 and VRC07-523LS were each *ρ*_c_ = 0.98 in group 3A (substantial agreement)(Pearson’s correlation). The dotted line is the identity line. The solid line is the trend line. Data are colored by bNAb.

Anti-idiotype-specific Binding Antibody Multiplex Assay (BAMA) and TZM-bl neutralization assays were used to individually measure PGDM1400, PGT121 and VRC07-523LS levels in serum (Fig. 1 and Supplementary Tables 5–7). The median (minimum, maximum) PGDM1400 elimination half-life (*t*_1/2_) estimates for the groups without HIV were 20.77 days (17.8, 24.7) when given alone and 17.4 days (15.3, 24.1) when co-administered with PGT121, respectively (Fig. 1a,b and Supplementary Tables 5 and 7). There was a trend of shorter PGDM1400 *t*_1/2_ when co-administered with PGT121 (*P* = 0.05) (Supplementary Table 8). The median (minimum, maximum) PGT121 elimination *t*_1/2_ estimates for the group without HIV was 20.2 days (15.8, 27.8). In the group with HIV, the median (minimum, maximum) elimination *t*_1/2_ estimates were 11 days (10.1, 18.7) for PGDM1400, 11.8 days (10.4, 20.5) for PGT121 and 29.3 days (27.4, 37.9) for VRC07-523LS, given that the latter bNAb is equipped with the half-life-extending LS variant^13^. Serum neutralizing activity for each of the three bNAbs was also measured using a combination of three pseudoviruses, each with respective neutralization susceptibility to one but not the other two bNAbs as follows: 6540.v4.c1 (PGDM1400), CH505TF.N334S.N160A.N280D.1 (PGT121) and CAP220.2.00_A8_5B (VRC07-523LS). bNAb concentrations measured by binding and neutralizing antibody assays generally displayed moderate to substantial concordance (*ρ*_c_ > 0.9) (Fig. 1e).

### Antiviral activity

**Fig. 2:**
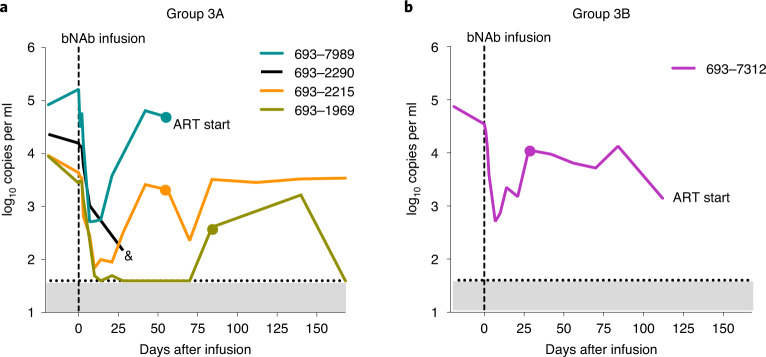
HIV-1 RNA levels. Plasma HIV-1 RNA levels (RNA copies per ml) are shown after PGDM1400, PGT121 and VRC07-523LS infusion at 20 mg kg^−1^ each (group 3A) (**a**) and after PGDM1400 and PGT121 infusion at 30 mg kg^−1^ each (group 3B) (**b**) in viremic participants with HIV not on ART. The dotted line indicates the LLoQ for HIV-1 RNA levels (40 copies per ml). Dots indicates when a sample was collected for sequencing. If and when ART was started is indicated in the figures. The symbol ‘&’ indicates the time point when participant 693–2290 was lost to follow-up.

Baseline viral loads (VLs) at the day of bNAb infusion in five viremic participants with HIV not on ART, enrolled in group 3, varied from 2,770 to 163,130 copies per ml (mean, 44,136 copies per ml) (Fig. 2 and Supplementary Table 9). One individual in group 3A (participant 693–2290) missed several visits and eventually was lost to follow-up on day 28 and was, therefore, excluded from further virological analyses. After a single infusion of PGDM1400, PGT121 and VRC07-523LS at 20 mg kg^−1^ each (Fig. 2a), all other three participants in group 3A showed a rapid decrease in their viral loads between baseline (mean of screening and day 0) and day 7 that varied between −1.50 and −2.21 log_10_ copies per ml with a mean of −1.76 log_10_ copies per ml. The median time to reach the nadir in viremia was 10 days (range, 8–15), and the mean drop in VL was −2.04 log_10_ copies per ml at nadir. Viral rebound, as defined by a confirmed increase of ≥0.5 log_10_ copies per ml above nadir, occurred between 13 days and 70 days after nadir (median, 20 days) or days 21–85 after bNAb infusion, with VL levels trending toward day 0 pre-infusion levels. The one participant in group 3B (participant 693–7312), who received PGDM1400 and PGT121 at 30 mg kg^−1^ each, showed a decrease in plasma VL of −2.16 log_10_ (day 7 difference from mean of screening and day 0) with a nadir at day 6 after infusion, before VL rebounded by day 30 (24 days after nadir). The VL, however, remained lower than the pre-infusion baseline (mean, −0.9 log_10_ copies per ml) until ART was started on day 108.

### bNAb resistance

**Fig. 3:**
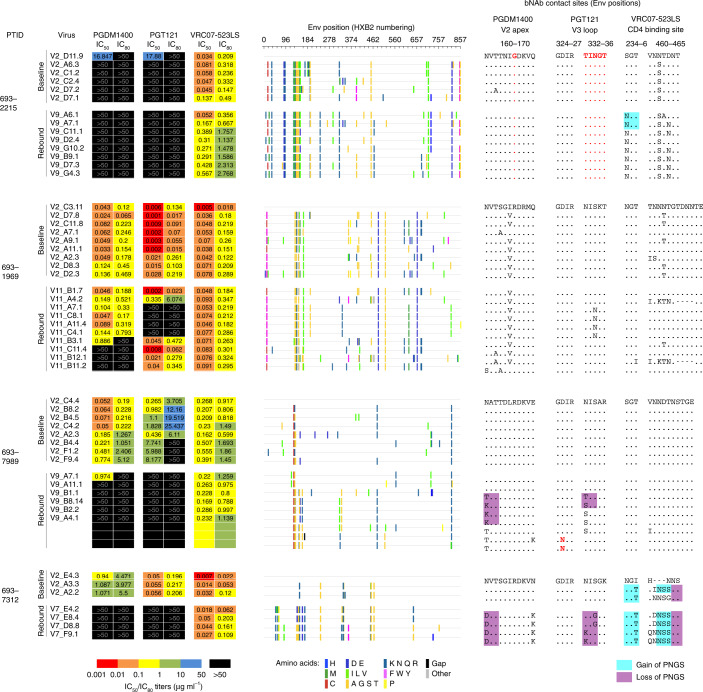
Neutralization sensitivity to bNAbs and escape variants. Left: For each participant, the pseudovirus IC_50_ and IC_80_ values (µg ml^−1^) for each bNAb are shown. Note: participant 693–7312 was treated with PGT121 and PGDM1400 dual therapy. Center: Highlighter plots showing amino acid *Env* mutations in participant viruses. The first baseline virus for each participant is treated as the reference sequence, and all amino acid mutations away from this reference *Env* are shown. Right: Env sequences for critical epitope sites for each of the bNAbs are shown. The first baseline *Env* for each participant is taken as the reference sequence, with dots for subsequent *Env*s indicating identity to the reference *Env*. Resistance mutations to each bNAb are highlighted in red. Gain or loss of PNGS as compared to the reference *Env* are highlighted with cyan or purple boxes, respectively. Note: loss of glycans 160 and 332 are associated with resistance to PGDM1400 and PGT121, respectively, whereas gain of glycan 234 and in hypervariable V5 loop are associated with resistance to VRC07-523LS. PTID, participant ID.

**Extended Data Fig. 2:**
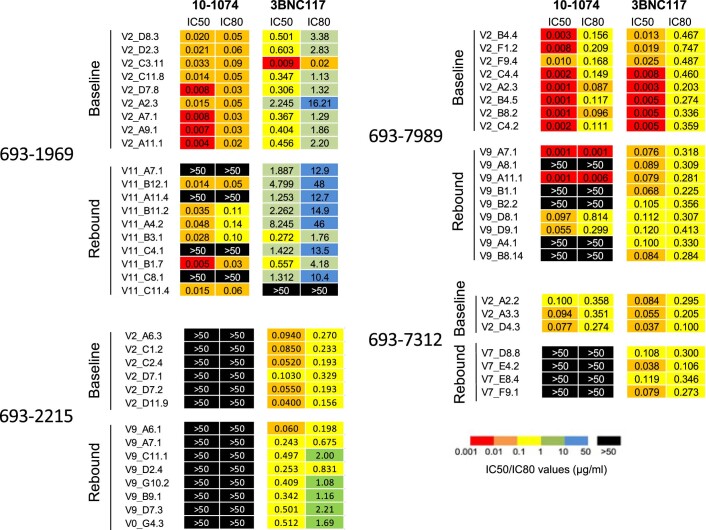
Neutralization sensitivity to bNAbs. For each participant, the pseudovirus IC50 and IC80 values (µg/ml) for the V3 glycan antibody 10-1074 and the CD4 binding site antibody 3BNC117 are shown.

**Extended Data Fig. 3:**
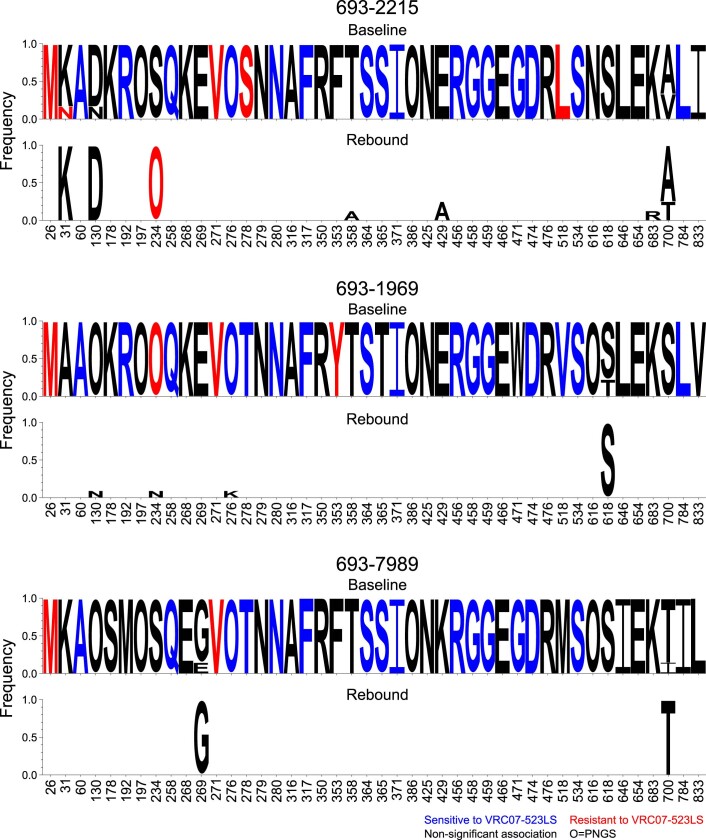
Mutations at VRC07-523LS signature sites. All HXB2 sites significantly associated with sensitivity/resistance to VRC07-523LS from Bricault et al.^1^ are shown. At these sites, amino acids are shown as logos with height of the letter indicating its frequency. O represents Asn in an N-linked glycan sequon (N-X-S/T where X is not Pro). For each participant, top logos show the baseline Envs, and bottom show rebound Envs. To highlight differences, amino acids in the rebound Envs that are invariant from the baseline Envs are whitened out. If the baseline Envs had two or more variants, from which one was found in the rebound Envs, those variants are still shown (for example site 31 in participant 693-2215). Blue amino acids are associated with sensitivity to VRC07-523LS, red with resistance, and black showed no significant associations.

**Extended Data Fig. 4:**
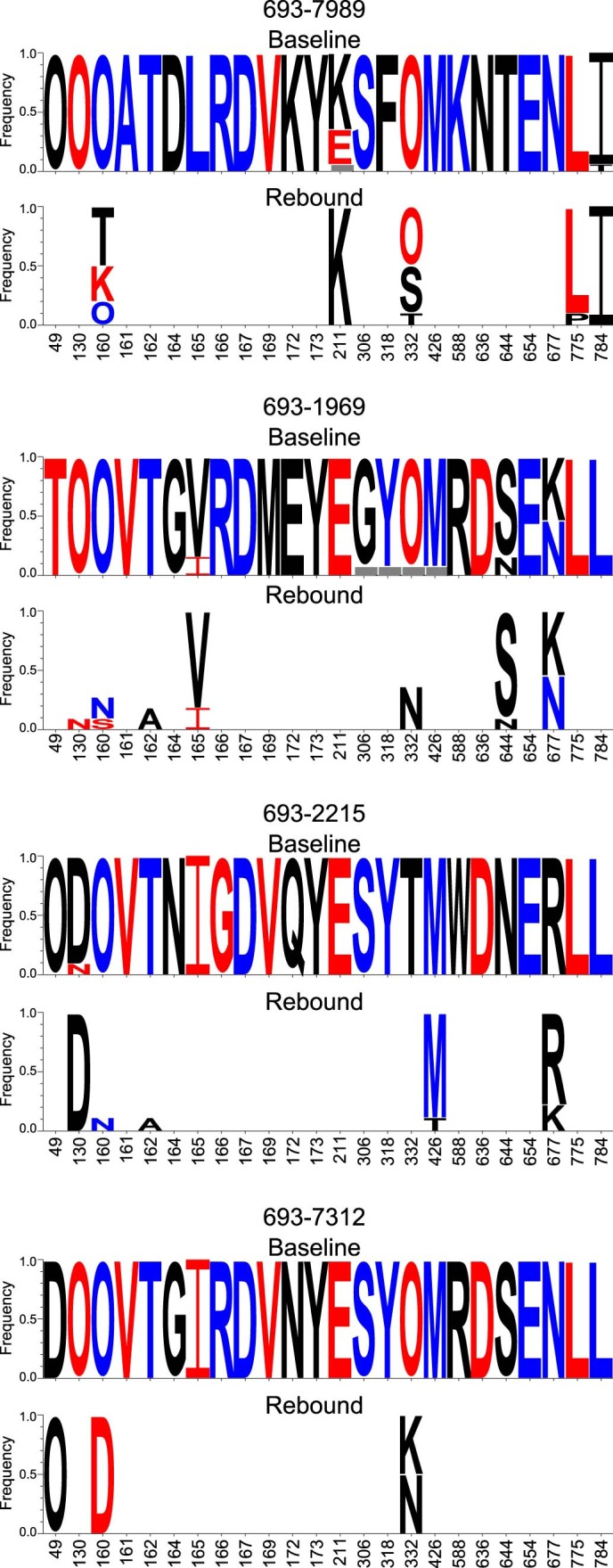
Mutations at PGDM1400 signature sites. All isolated Envs from each participant are used for these analyses. All HXB2 sites significantly associated with sensitivity/resistance to PGDM1400 from Bricault et al.^1^ are shown. At these sites, frequency of amino acids are shown as logos with height of the letter indicating its frequency. For each PTID, top logos show the baseline Envs, and bottom show rebound Envs. To highlight differences, amino acids in the rebound Envs that are invariant from the baseline Envs are whitened out. If the baseline Envs had two or more variants, from which one was found in the rebound Envs, those variants are still shown (for example site 211 in 693-7989). Blue amino acids are associated with sensitivity to PGDM1400, red with resistance, and black showed no significant associations. O represents Asn in an N-linked glycan sequon (N-X-S/T where X is not Pro). Grey box indicates deletion.

**Extended Data Fig. 5:**
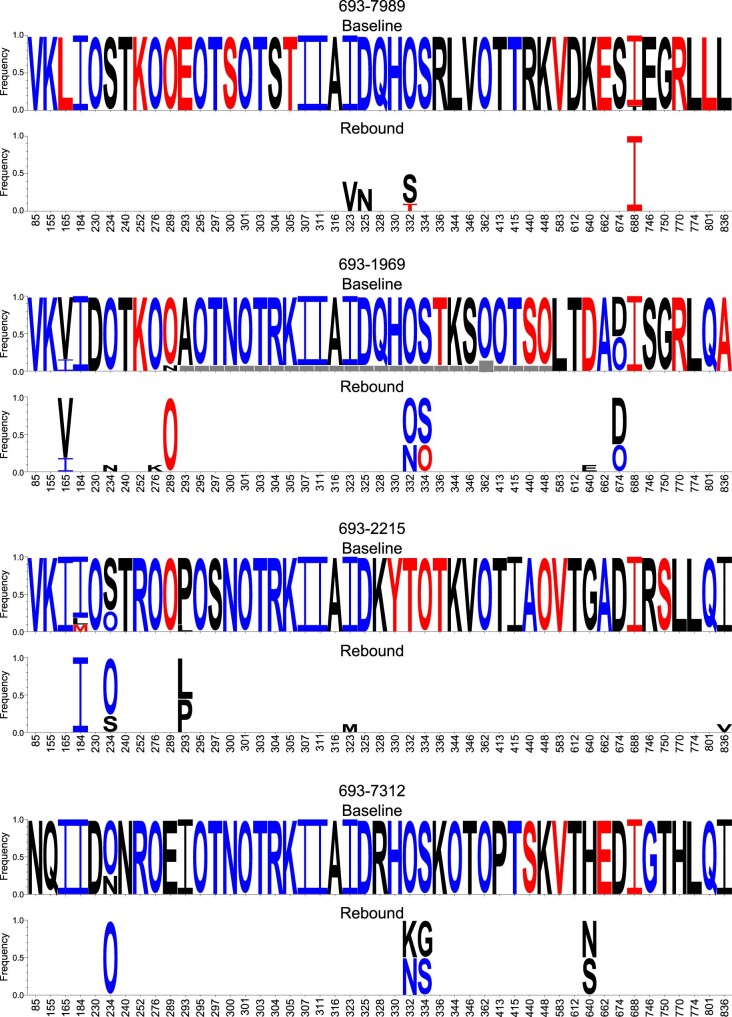
Mutations at PGT121 signature sites. Same as Extended Data Figs. 3 and 4, but using PGT121 signature sites from Bricault et al^1^.

For the four participants of group 3 for whom full follow-up visits and samples were available, HIV-1 pseudoviruses constructed from plasma single-genome amplification (SGA) of circulating viruses were tested for bNAb sensitivity in TZM-bl assay at baseline and during viral rebound. In total, we generated 43 sequences from baseline and 37 sequences from rebound viruses (average 13 baseline and 11 rebound sequences per participant, respectively, excluding 693–7312, from whom three baseline and four rebound sequences were generated). All participants either showed resistance to PGDM1400 and PGT121 at baseline (participant 693–2215) or showed viral escape at rebound (participants 693–1969, 693–7989 and 693–7312 (PGT121+PGDM1400-only therapy). We then analyzed *Env* sequences from participant isolates with the matched neutralization data to identify the mutations underlying the resistance patterns (Fig. 3 and Extended Data Fig. 2). Analyses of additional *Env*s that could not be made as pseudoviruses corroborated the findings below (Extended Data Figs. 3–5).

Viral escape from PGDM1400 for participants 693–1969, 693–7312 and 693–7989 was mediated by the loss of the potential N-linked glycosylation site (PNGS) at residue 160, which is a key Env glycan contact for V2 apex bNAbs^14^ and is strongly associated with susceptibility^15^. Each participant showed different patterns for losing this glycan site through mutations at either residue 160 and/or 162 (for example, N160D for participant 693–7312; N160S and T162A for participant 693–1969), and multiple resistance mutations were also found within individual participants (693–1969 and 693–7989). For participant 693–2215, both baseline and rebound viruses were resistant to PGDM1400, likely explained by the presence of glycine at the Env site 166, instead of the more common arginine at this site. G-166 is a known resistance-associated mutation for PGDM1400 (ref. ^15^) at a critical bNAb contact site^14^. For participant 693–7989, two rebound viruses that were resistant to PGDM1400 did not show any canonical resistance mutations, such as those identified as signatures in Bricault et al.^15^ and from previous mutagenesis studies compiled on the Los Alamos HIV Databse CATNAP (https://www.hiv.lanl.gov/content/index)^16^. These two viruses did have a V120I mutation, which could be a candidate mutation for resistance based on its structural proximity to PGDM1400-like bNAb contact sites^14^.

The escape from PGT121 occurred for all participants predominantly due to the loss of the PNGS at 332, which is a key contact glycan for V3 glycan bNAbs^17^ and a requisite for susceptibility to such bNAbs^15^. This occurred by different routes across participants, either gaining PNGS at 334 (participants 693–2215 and 693–1969) or not (participants 693–7312 and 693–7989). In the latter two participants, 2–3 different resistance mutations per participant were found in the rebound viruses. Two rebound viruses in participant 693–7989 escaped PGT121 not by losing N332 glycan but, instead, by the D325N mutation, which is also a resistance signature for PGT121 (ref. ^15^) and was found in clinical escape from monotherapy with PGT121 (ref. ^18^) and with 10–1074 (refs. ^2,4,8^). For two rebound viruses from participant 693–7989 (A7.1 and A11.1), none of the V3 loop resistance mutations was found; however, they did have mutations that shifted a hypervariable V1 PNGS two sites toward the N-terminus relative to other viruses from this participant, and V1 loop glycans have been shown to affect V3 glycan bNAb sensitivity^15,19,20^.

**Fig. 4:**
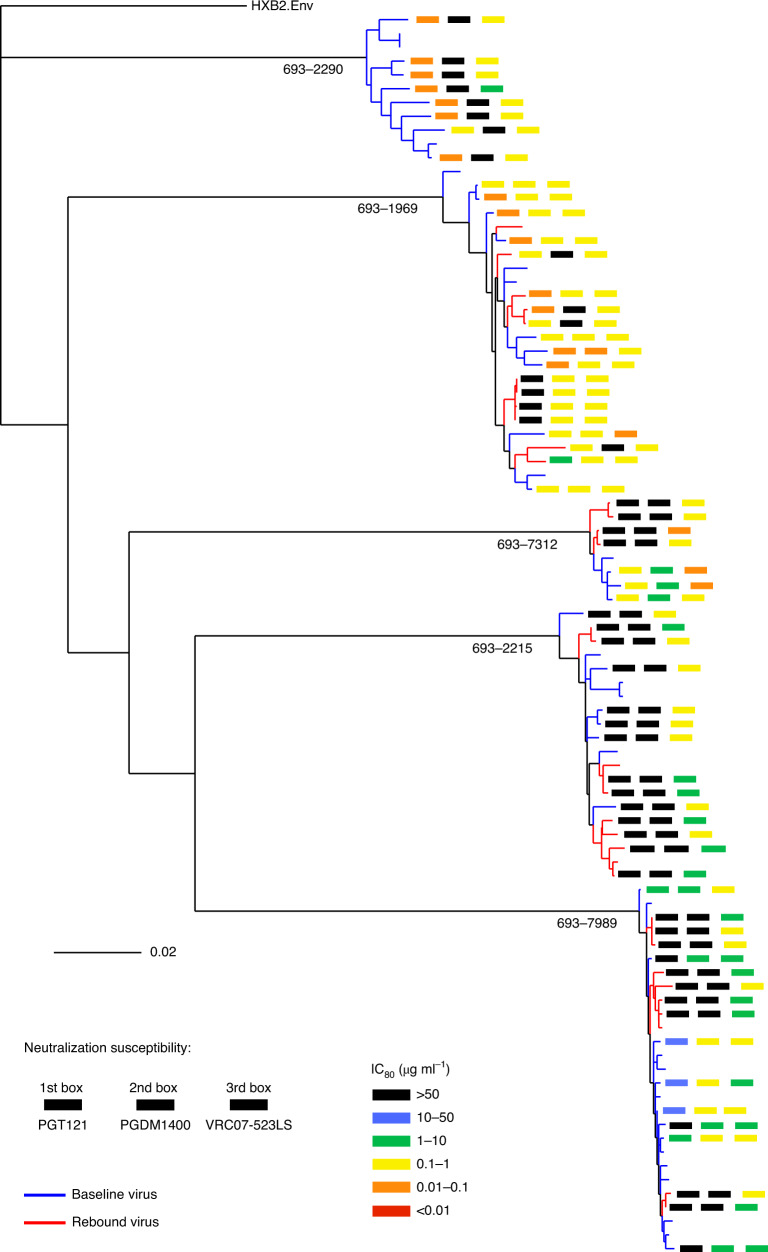
Phylogenetic tree of participant baseline and viral rebound *Env*s. Baseline *Env*s are colored by blue tips and rebound *Env*s by red. For Envs tested for bNAb neutralization (Fig. 3), boxes next to the tips indicate IC_80_ values for PGT121, PGDM1400 and VRC07-523LS, going from left to right, color-coded using the scheme in the legend. Participant IDs are shown at the root of each participant *Env* cluster.

Analyses of the phylogenetic relationships between baseline and rebound viruses showed that bNAb escape was polyclonal (Fig. 4). Because our sampling of viral diversity of circulating strains, although typical for such studies^4^, is only a limited snapshot of the full diversity within each participant, our analyses are not powered to address the question of whether the bNAb-resistant rebound viruses were present at low frequency in baseline plasma, were mutated from baseline viruses and/or were activated from the latent reservoir.

**Fig. 5:**
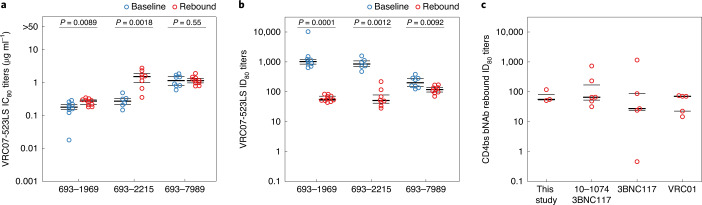
VRC07-523LS neutralizing activity for baseline and rebound viruses. **a**, VRC07-523LS IC80 values (µg ml^−1^) are shown for each participant with baseline viruses shown in blue and rebound viruses in red (baseline and rebound viruses for 693–1969: nine and ten viruses; 693–2215: six and eight viruses; 693–7989: eight and ten viruses, respectively). Thick horizontal black lines indicate medians, and thin black lines indicate 25th and 75th percentiles. *P* values indicate significance from one-sided Wilcoxon rank-sum test. **b**, VRC07-523LS plasma ID_80_ titers are shown for each participant. Same color scheme, number of viruses examined and statistics as in **a**. **c**, Comparison of CD4bs bNAb rebound ID_80_ titers across different CD4bs bNAbs. Each point represents the per-participant median CD4bs bNAb ID_80_ titers for rebound viruses from each study. Medians are shown by thick black lines and 25th and 75th percentiles by thin black lines. No significant differences were observed between any two studies. Studies: Bar-On et al.^4^, Caskey et al.^3^ and Lynch et al.^1^.

VRC07-523LS resistance at viral rebound developed in two of the three participants receiving triple bNAb therapy (Fig. 3). Although rebound viruses for participant 693–1969 were significantly more resistant, the median IC_80_ increase was only 1.5-fold (Fig. 5a). For participant 693–2215, rebound viruses were 5.6-fold more resistant as compared to baseline viruses. For participant 693–7989, no appreciable change in VRC07-523LS susceptibility was found between baseline and rebound viruses. However, some rebound viruses showed complete resistance (IC_50_ > 50 µg ml^−1^) to the CD4bs antibody 3BNC117 (Extended Data Fig. 2).

**Extended Data Fig. 6:**
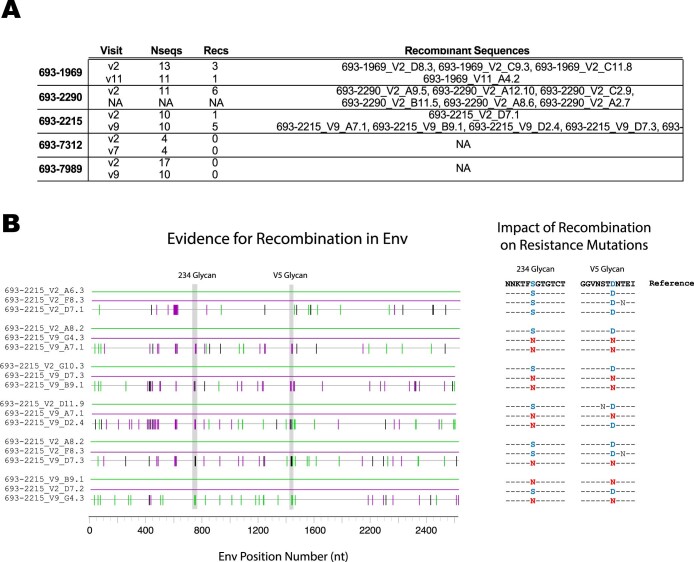
Recombination detection in three out of five samples. A total of 16 recombinants were detected in 3 out of 5 samples (A). Each row shows the total number of sequences at each visit per participant (NSeq), the total number of recombinants detected (NRecs), and the sequence names of the recombinants (last column on the right). No recombinants were detected in participants 693-7312 and 693-7989, although our power to detect recombination suffered from the low number of sequences (range 4-17 per participant, per visit; median=10). All recombinants were found to be statistically significant after multiple testing correction using a Wald-Wolfowitz Runs Test statistic implemented by the LANL tool RAPR^2^. (B) Impact of recombination on participant 693-2215. The full Env genome of all recombinant triplets from participant 693-2215 is shown on the left, solid green and purple lines for the parental strains at the top, and gray for the recombinant below, with tic marks indicating the sites where the daughter strain differs from at least one parent, color coded as follows: green if the recombinant matches the green parent at that site, purple if the recombinant matches the purple parent, and black if it doesn’t match either parental strain. Vertical gray bands show the positions in the genome of the 234 glycan and the V5 glycan where we find evidence of recombination favoring the resistant form over the sensitive one. This is highlighted on the right, with the reference amino acids surrounding the two glycan regions shown at the top and below the corresponding sequence for each recombinant triplet. Dashes indicate amino acids were the sequences match the reference. Mutations at the glycans are shown in red if they confer resistance and blue if they confer sensitivity. The top triplet shows the only recombinant found at the first time point where no resistant mutations were detected. In the remaining triplets, recombination preferentially selects the resistant mutation 4 out of 4 times for the glycan at position 234, and 3 out of 4 times at the site of the V5 glycan.

For participants 693–1969 and 693–7989, no relevant mutations associated with VRC07-523LS resistance were detected in rebound viruses as compared to baseline viruses (Extended Data Fig. 3), consistent with the absent or subtle neutralization resistance change. For participant 693–2215, all rebound viruses gained a glycan at site 234, and all but two rebound viruses gained a glycan in hypervariable V5 loop. Both of these glycan gain mutations have been shown to be associated with neutralization resistance to VRC07-523LS^15^. We also detected a role for recombination in selecting for VRC07-523LS-resistant mutations in participant 693–2215 (Extended Data Fig. 6). Overall, our recombination analyses detected recombinant Envs in participants 693–1969 and 693–2215 as well as among the baseline sequences from participant 693–2290, who was subsequently lost to follow-up (Extended Data Fig. 6a). Although we could not detect any recombinants in other patients, this is likely due to the limited sampling of patient viral diversity rather than absence of recombination. In participant 693–2215, we found evidence that the resistance-conferring glycan at 234, found in all rebound viruses, as well as the glycan in the hypervariable V5 loop, were favored by recombination: four out of four and three out of four recombination events, respectively, from parental strains discordant at these sites carried forward the resistant-conferring mutations in the recombinant daughter Env (Extended Data Fig. 6b). The same kind of analysis was not possible for the other two participants because 693–1969 had no known VRC07-523LS resistance mutations in the rebound population, and 693–2290 was lost to follow-up before viral rebound.

To further understand why viral rebound occurs without developing substantial VRC07-523LS neutralization resistance, we analyzed VRC07-523LS serum ID_80_ titers for the baseline and rebound viruses. Two non-exclusive mechanisms could allow viral escape: (1) evolution of viral resistance to VRC07-523LS neutralization and (2) decay of VRC07-523LS concentrations to sub-therapeutic levels. Serum ID_80_ titers capture both of these mechanisms as they are based on both VRC07-523LS serum concentrations and the neutralization sensitivity of viruses (as described in the Methods). For each participant in our study, ID_80_ titers of VRC07-523LS at the time of viral rebound were significantly lower than those at day 7 after bNAb administration, regardless of whether VRC07-523LS neutralization resistance (increased IC_80_ values) developed or not (Fig. 5b). The median rebound serum ID_80_ titers were also similar across participants (51–118). This result suggests that viral escape from VRC07-523LS occurs below a common threshold of serum neutralizing activity against contemporaneous viruses, which can be reached by either viruses developing neutralization resistance (participant 693–2215) or decay of bNAb concentrations (participant 693–7989), or by a combination of both (participant 693–1969). Although the low number of participants precluded robust statistical analyses, these data still suggest that maintaining ID_80_ titers above the threshold of ~1,000 could improve viral control.

**Extended Data Fig. 7:**
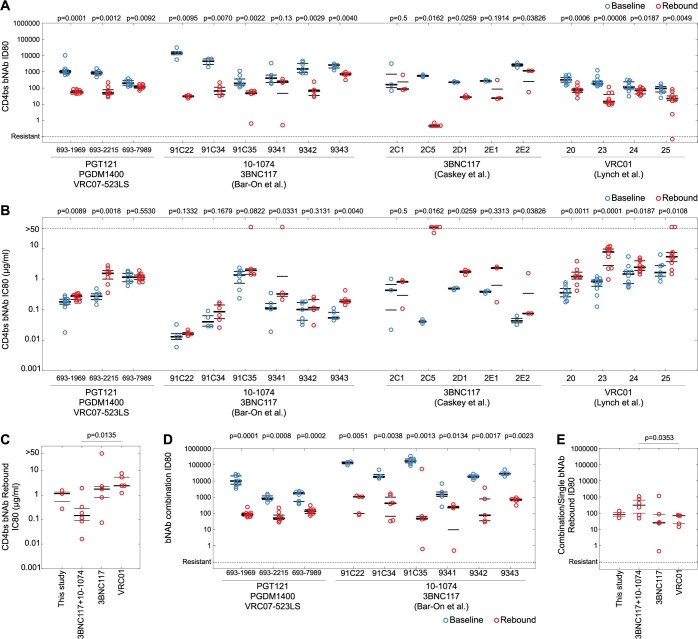
Comparison of CD4bs bNAb and bNAb combination neutralizing activity from this study to other studies. (**a**) CD4bs bNAb serum ID80 titers at baseline and rebound from each participant across the 4 studies^3–5^. Similar to Fig. 4b, see methods for ID80 calculation details. A one-sided Wilcoxon rank sum test was used to calculate statistical significance of differences between baseline and rebound titers for each patient, and p-values are shown on the top of the panel. All participants except 9341, 2C1 and 2E1, showed significantly lower serum ID80 titers at rebound as compared to baseline. (**b**) CD4bs bNAb IC80 values (µg/ml) as tested in vitro against baseline and rebound viruses from each participant across the studies. Similar to (A). Most participants showed significant difference (p < 0.05 using one-sided Wilcoxon rank sum test), with the exception of participants 693-7989, 91C22, 91C34, 91C35, 9342, 2C1 and 2E1 for whom no significant differences were found (p > 0.05). (**c**) Summary of in vitro IC80 values (µg/ml) of rebound viruses per study. Each point shows the per-participant median CD4bs bNAb IC80 value (µg/ml) for rebound viruses from each study. The only significant difference using two-sided Wilcoxon rank sum test was found to be between 3BNC117 + 10-1074 and VRC01 studies (p = 0.0134), and a trend when comparing this study to VRC01 (p = 0.0719); all other comparisons resulted in p > 0.17 (p-values not shown). (**d**) Same approach as (A) but using bNAb combination serum ID80 titers (see methods for combination ID80 titer calculation details). All participants showed significantly lower ID80 titers (that is due to higher resistance) for rebound viruses as compared to baseline viruses (p < 0.05 using one-sided Wilcoxon rank sum test). (**e**) Same approach (C) but summarizing the combination serum ID80 titers at rebound across studies. For 3BNC117 and VRC01 groups, single bNAb rebound ID80 titers are shown. The only significant difference was observed between the 3BNC117 + 10-1074 and VRC01 studies, with the former having significantly higher rebound ID80 titers than the latter (p = 0.0353 using two-sided Wilcoxon rank sum test). All other comparisons were not significant (p > 0.23; p-values not shown).

Comparison to other clinical studies testing CD4bs bNAbs in a post hoc analysis showed that similar mechanisms of viral escape are at play. During both 3BNC117 monotherapy^3^ or dual therapy^4^, and during VRC01 monotherapy^1^, viral rebound in viremic individuals was observed at similar serum ID_80_ titer ranges for the respective bNAb, as compared to VRC07-523LS in this study (Fig. 5c and Extended Data Fig. 7a). Furthermore, rebound viruses demonstrated a similar pattern of in vitro IC_80_ value changes to 3BNC117 and VRC01 (that is, unchanged susceptibility or increased resistance), as was observed for VRC07-523LS in this study (Extended Data Fig. 7b,c), including six study participants who showed no significant change in 3BNC117 IC_80_ values between baseline and rebound viruses (four from dual therapy and two from monotherapy studies). Despite some minor differences, the IC_80_ values for rebound viruses across these previous studies were fairly similar to VRC07-523LS in this study. Together, these results suggest that, unlike the development of complete neutralization resistance against V2 apex and V3 glycan-targeted bNAbs by individual mutations at the target epitope, viral escape from CD4bs bNAbs involves a combination of the evolution of partial CD4bs bNAb escape as well as the decay of serum bNAb concentrations.

**Extended Data Fig. 8:**
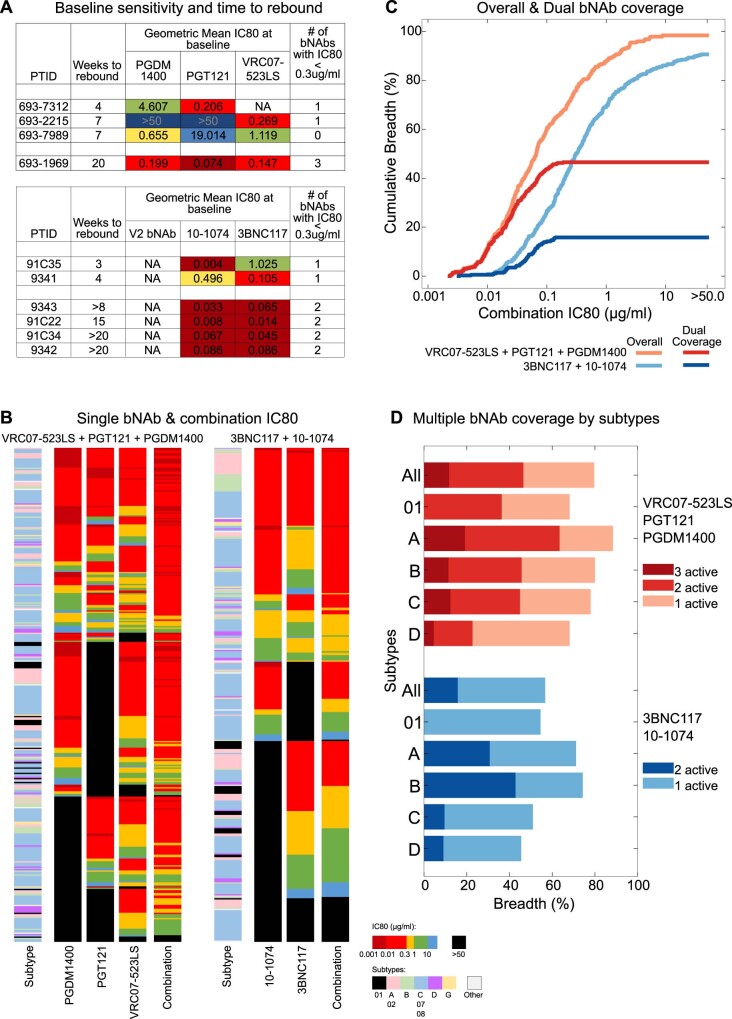
Neutralizing activity of bNAb combinations against global heterologous viruses. (**a**) Geometric mean IC80 values (µg/ml) of baseline viruses for this study (top) and from Bar-On et al^3^ (bottom). The participants are ordered according to time to rebound. These data suggest that longer times to rebound were found for those participants whose baseline IC80 < 0.3 µg/ml for at least 2 bNAbs in the therapeutic combination. (**b**) IC80 values for individual and combination bNAbs. Data are shown as heatmaps with viruses on the rows. Viral subtypes and individual bNAb and bNAb combination IC80 values are shown as columns. Color coding of subtypes and IC80 values is shown on the bottom right. Viruses are ordered according to the number of bNAbs with IC80 < 0.3 µg/ml, with most number of bNAbs active on top and least on the bottom, and according to neutralization sensitivity to each bNAb. The ordering of viruses between the left and the right panels is different. The single bNAb neutralization data for these non-tier-1 374 viruses was obtained from CATNAP, as previously described6, combination IC80 values were predicted using Bliss-Hill model assuming each bNAb at equal concentrations. Combination IC80 values are the sum of concentrations of each bNAb in the combination for predicted 80% neutralization of a given virus. (**c**) Overall and dual coverage breadth potency curves for VRC07-523LS + PGT121 + PGDM1400 (salmon and red, respectively) and for 3BNC117 + 10-1074 (light and dark blue, respectively). For dual coverage a virus is considered only if it is neutralized by 2 or 3 bNAbs at individual bNAb IC80 < 0.3 µg/ml, based on the suggested trend of this metric associated with longer time of viral control from panel (A). (**d**) Subtype-specific coverage of single, dual and triple bNAb activity for VRC07-523LS + PGT121 + PGDM1400 (shades of red), and for 3BNC117 + 10-1074 (shades of blue). Single bNAb activity threshold is IC80 < 0.3 µg/ml.

Indeed, serum bNAb combination ID_80_ titers for rebound viruses for the triple bNAb combination studied here were not significantly different than the dual bNAb therapy used in a previous study by Bar-On et al.^4^; and although the triple combination rebound ID_80_ titers in our study were ~5-fold lower than the dual combination in Bar-On et al.^4^, they tended to be higher than in the single bNAb studies (Extended Data Fig. 7d,e). In this post hoc analysis, we noticed a trend between longer times to rebound when having at least two bNAbs with geometric mean IC_80_ < 0.3 µg ml^−1^ for baseline viruses when combining our data and the Bar-On et al.^4^ study (Extended Data Fig. 8a). Applying the IC_80_ < 0.3 µg ml^−1^ threshold to a panel of 374 cross-clade pseudoviruses^9^, as previously reported, we found that using three bNAbs improved the neutralization coverage as compared to a dual bNAb cocktail; however, still less than 50% of viruses would be neutralized by two active bNAbs in this triple bNAb combination (Extended Data Fig. 8b–d). These data emphasize the critical need to further extend bNAb breadth, either by adding additional antibodies into bNAb combinations, for enhanced potency and coverage, or by further engineering multi-specific monoclonals to achieve this goal.

## Discussion

In this study, we established the safety and tolerability of the PGDM1400 antibody when administered intravenously, alone or in combination with the bNAbs PGT121 and VRC07-523LS in participants with and without HIV. To our knowledge, this is the first report of a triple antibody combination in humans for the treatment of HIV-1. PGDM1400 is one of three V2-targeting antibodies that are being developed, along with CAP256-VRC26.25 (ref. ^21^) and its half-life-optimized version CAP256V2LS. This bNAb class, although with limited breadth against clade B viruses^9^, has superior potency compared to other bNAb classes and is, therefore, a promising component of bNAb combinations. Indeed, PGDM1400 is also being explored in combination with VRC07-523LS and PGT121 or 10-1074 in participants without HIV in the ongoing HVTN130 study (NCT03928821) with the goal to develop passive immunization strategies for HIV prevention.

Unfortunately, despite the extensive breadth of this bNAb combination, viral rebound in the presence of selected resistance mutations against PGDM1400 and PGT121 occurred rapidly in the studied participants. This might be explained, in part, by the potentially increased prevalence of PGDM1400 and PGT121 escape mutations in circulating viruses, as we previously reported^12^. Furthermore, escape from PGDM1400 and PGT121 might more readily develop compared to the escape from CD4bs antibodies, as the critical variable loop glycans that are targeted by both antibodies are prone to deletion and modification. This also implies that such different barriers to resistance for individual bNAbs or bNAb classes might need to be considered when selecting antibodies for treatment regimens. The most durable control was seen for participant 693–1969 who had highly sensitive baseline viruses to all three bNAbs (Fig. 3) and who demonstrated lower than baseline HIV RNA levels through the end of the study. Nevertheless, although bNAb administration potentially resulted in a more rapid initial viral decline than what has historically been reported during ART initiation^22^, the overall therapeutic efficacy of the bNAbs in the setting of active plasma viremia did not reach the level of virological suppression of current ART regimens, specifically those that include integrase inhibitors^23^. Furthermore, given the variable neutralization profiles of bNAbs against global viruses, the ability to neutralize most viruses with three active bNAbs would likely require combinations of at least four bNAbs targeting different epitopes^9,24^ (Extended Data Fig. 8). This could potentially be achieved by engineered multi-valent antibodies such as the tri-specific antibody SAR441236 (ref. ^25^) that combines the CD4bs specificity of VRC01-LS, PGDM1400 and the gp41 MPER binding of 10E8v4-variant, which is currently under clinical evaluation (NCT03705169). Viral replication and breakthrough viremia during treatment with VRC07-523LS, and CD4bs bNAbs in general, can occur despite substantial bNAb concentrations in serum and/or relatively low-level increase in neutralization resistance. Although the apparent lack of complete escape from VRC07-523LS neutralization in the presence of serum bNAb levels might suggest that such escape could be difficult for viruses to achieve, it also does not seem to be required to sustain robust plasma viremia. By maintaining higher levels of the bNAb, such as the suggested ID_80_ titers above the threshold of ~1,000, enhanced and perhaps longer-term control of viremia might be achieved; however, this would need to be confirmed in future clinical studies. Furthermore, another defining factor for the efficacy of bNAb combinations might be the pre-treatment level of viral replication. In our study, bNAbs were administered to viremic participants with up to ~160,000 HIV RNA copies per ml at the time of infusion. The antiviral activity and the ability to control virus more robustly might be enhanced in individuals with low-level viremia and/or low-level viral replication. Indeed, this was reported for macaques where PGT121 administration led to long-term SHIV control in animals with low baseline viral loads^26^, and we also previously observed that PGT121 suppressed viral replication for extended periods in a subset of participants with low baseline HIV RNA levels^18^. This, therefore, suggests, that although daily ART, and likely future long-acting antiretrovirals (ARVs), reliably suppress HIV in viremic individuals, the triple bNAb combination reported here might still perform well in individuals with low levels of replicating/reactivating virus—that is, what is seen during ATI, as reported by Mendoza et al.^8^ with a double bNAb combination. Indeed, such a strategy, testing a triple bNAb combination during ATI, is currently underway in an ongoing study (NCT03721510). Furthermore, in contrast to ARV medicines that must be continued indefinitely, bNAbs might have a significant advantage. Given their ability to target and mediate elimination of infected cells^27^ and to harness host immune responses^28,29^, a potential effect on reducing the size of the latent reservoir during bNAb therapy has been suggested^27,30^.

Furthermore, combination bNAb therapy will likely be necessary for HIV prevention strategies, to provide broad coverage against the diversity of globally circulating viral strains. As recently reported in the Antibody-Mediated Prevention (AMP) study, the single bNAb VRC01 did not prevent overall HIV acquisition more effectively than placebo but was effective in preventing infection against VRC01-sensitive HIV isolates, suggesting that bNAb prophylaxis can be effective if the viral coverage is broad enough^31^. Here as well, further optimization of bNAbs and bNAb regimens is necessary, and their role for prevention strategies, specifically with long-acting ARVs as a promising alternative, will need to be determined. Thus, combinations of ARVs and bNAbs, taking advantage of the synergistic effects of the single agents, are under active exploration (NCT03739996).

The present study has several limitations. The sample size for part 2 is small, with only three individuals available for complete virological analysis after triple bNAb therapy. The participants were not pre-screened for pre-existing bNAb resistance and/or selected based on viral susceptibility. Furthermore, participants, including participant 693–1969 who demonstrated the strongest virological response, were not tested for plasma ARV drug levels to confirm ART naivety.

In summary, the results presented here will help to advance understanding of the capabilities, the limitations and the future role of bNAb combinations in HIV prevention and care. PGDM1400 could be included in future bNAb cocktails for passive immunization strategies, either for prevention or for therapeutic approaches given its complementary epitope specificity. Our data contribute to efforts to define a threshold bNAb(s) titer(s) that is required for viral control and that will inform dosing requirements and/or bNAb half-life engineering strategies for future monoclonal therapeutics.

## Methods

### Study design

This study evaluated the safety, pharmacokinetics and antiviral activity of PGDM1400, PGT121 and VRC07-523LS bNAbs. Part 1 of the study was a single-center, randomized, double-blind, dose-escalation, placebo-controlled trial of PGDM1400 alone (part 1, group 1, arm 1) or in combination with PGT121 (part 1, group 2, arm 2) in HIV-uninfected adults at the Beth Israel Deaconess Medical Center (BIDMC) in Boston, Massachusetts (Table 1). Part 2 of the study was a multi-center, open-label, non-randomized trial of PGDM1400, PGT121 and VRC07-523LS (part 2, group 3a, arm 1) or PGDM1400 and PGT121 (part 2, group 3B, arm 2) in viremic adults with HIV not on ART at three sites: BIDMC; Orlando Immunology Center (OIC) in Orlando, Florida; and Houston AIDS Research Team (HART), McGovern Medical School at The University of Texas Health Science Center at Houston in Houston, Texas (Table 1). The protocol was approved by the BIDMC institutional review board (IRB), the OIC IRB and the HART Committee for the Protection of Human Subjects. The study was registered at ClinicalTrials.gov (NCT03205917) In part 1, we evaluated three intravenous doses (3 mg kg^−1^, 10 mg kg^−1^ and 30 mg kg^−1^) of PGDM1400 given once or three intravenous doses (3 mg kg^−1^, 10 mg kg^−1^ and 30 mg kg^−1^) of PGDM1400 and three doses (3 mg kg^−1^, 10 mg kg^−1^ and 30 mg kg^−1^) of PGT121 given once (Extended Data Fig. 1). Each participant in part 1 received either PGDM1400 or PGDM1400 and PGT121 versus placebo at a ratio of 3:1 within each subgroup. In part 2, participants in group 3A received a single intravenous dose of PGDM1400, PGT121 and VRC07-523LS at 20 mg kg^−1^ each or PGDM1400 and PGT121 at 30 mgkg^−1^ each in group 3B. There was no placebo or blinding for part 2.

### Study participants

Participants were eligible for the study across groups if they did not have any clinically significant acute or chronic medical condition (besides HIV), such as chronic hepatitis B, active hepatitis C, significant psychiatric disorder, alcohol or substance use disorder or chronic kidney or liver disease and if they had a body mass index >18 kg m^−2^ and <35 kg m^−2^. Sexually active participants had to be willing to use contraception for 3 months after investigational product (IP) administration and could not be pregnant or breastfeeding. Participants were eligible for group 1 and group 2 if they were also 18–50 years of age and at low risk for HIV infection and willing to maintain low-risk behavior.

Participants with HIV (group 3) were eligible if they were 18–65 years of age, had CD4 ≥ 300 cells per μl, had no history of AIDS-defining illness within the previous 5 years and were not on ART for >6 months with detectable HIV-1 RNA levels between 1,000 and 100,000 copies per ml and (after appropriate counseling) were willing to defer ART treatment for at least 56 days after administration of the IP. All participants gave written informed consent and successfully completed an assessment of understanding before the initiation of study procedures.

### Randomization and masking

In part 1, eligible participants were enrolled first into the lowest dose subgroup of PGDM1400 alone (group 1A), and enrollment into the lowest PGDM1400 and PGT121 combination dose subgroup (group 2A) occurred only after the Protocol Safety Review Team (PSRT) reviewed the safety data through day 14 after administration of PGDM1400 alone and approved dose escalation. This staggered dose escalation was continued for each dose group. Participants in each subgroup were identified by a unique study identification number. Participants were randomized according to the randomization schedule prepared by the statisticians at the Data Coordinating Center (DCC, Emmes Company) before the start of the study. Participants were automatically assigned a specific allocation number as they were enrolled into the data entry system. An unblinding list (Pharmacy List) was provided to the unblinded site pharmacist by the DCC. Study staff (investigator and clinical personnel monitoring the safety and laboratory assay results) and participants were blinded with respect to the allocation of the IP. A site pharmacist was unblinded for the purposes of preparing the IP. Blinded participants were informed about their assignment (product or placebo) at study completion, once the data were locked. As the bNAbs and placebo (saline) looked identical in the infusion bag, no masking was required. In part 1, the four participants in each dose level subgroup (3 mg kg^−1^, 10 mg kg^−1^ or 30 mg kg^−1^) in group 1 and (3 + 3 mg kg^−1^, 10 + 10 mg kg^−1^ or 30 + 30 mg kg^−1^) in group 2 were randomized at a ratio of three antibody recipients to one placebo recipient, respectively (total of nine antibody recipients and three placebo recipients per group). At each dose level in part 1, IP administration was separated by at least 24 hours for each of the first three participants. Randomization in part 1 ensured that at least two participants received active product and were observed for at least 24 hours before administration to additional participants. IP administration was also separated by at least 24 hours for each of the first three participants in part 2, group 3A, who received the triple bNAb combination.

### IPs

PGDM1400 is a recombinant, fully human monoclonal antibody of the IgG1 isotype that binds to the HIV envelope. PGDM1400 was formulated in a 20 mM acetate, 9% sucrose, 0.008% polysorbate 80, pH 5.2 formulation buffer at a concentration of 50 mg ml^−1^. Each 10-ml vial contained 6 ml of PGDM1400.

PGT121 is a recombinant, fully human monoclonal antibody of the IgG1 isotype that binds to the HIV envelope. PGT121 was formulated in a 20 mM acetate, 9% sucrose, 0.008% polysorbate 80, pH 5.2 formulation buffer at a concentration of 50 mg ml^−1^. Each 10-ml vial contained 6 ml of PGT121.

VRC07-523LS is a recombinant, fully human monoclonal antibody of the IgG1 isotype that binds to the HIV envelope. VRC07-523LS was formulated at a concentration of 100 ± 10 mg ml^−1^ in a buffer composed of 50 mM histidine, 50 mM sodium chloride, 5% sucrose and 2.5% sorbitol at pH 6.8. Each vial contains 6.25 ± 0.1 ml or 2.25 ± 0.1 ml of VRC07-523LS filled in a standard 10-ml or 3-ml glass vial, respectively.

Placebo was 0.9% sodium chloride for injection (United States Pharmacopeia (USP)), in partial addition or flexible bags.

Participants received the IP via intravenous infusion over approximately 60 minutes per antibody/placebo.

### Safety assessments

Local and systemic reactogenicity safety data were collected for 3 days after IP administration (see Supplementary Information for Protocol and Schedule of Procedures). Data on unsolicited adverse events (AEs) were collected until 56 days after IP administration. Potential immune-mediated diseases (pIMDs) were considered AEs of special interest because they could potentially be caused by immune responses to the IP; pIMDs included both autoimmune diseases and also other inflammatory and/or neurologic disorders that may or may not have an autoimmune etiology. Data on pIMDs and serious adverse events (SAEs) were collected through study day 168. Blood samples for serum chemistry and hematology were collected at 12 time points throughout the study, whereas urine samples for pregnancy testing were collected at six time points, and urinalysis was collected after the screening time point only when clinically indicated. Blood samples for HIV-1 RNA levels and CD4^+^ T cell count were collected throughout the study for participants with HIV. Medical monitoring was provided by a PSRT and an independent Safety Monitoring Committee (SMC). Local and systemic AEs were graded by the NIAID Division of AIDS (DAIDS) Table for Grading the Severity of Adult and Pediatric Adverse Events, version 2.1, July 2017. For the first 24 hours after IP infusion or injection, any infusion-related reactions, including cytokine release syndrome, were graded by the National Cancer Institute Common Terminology Criteria for Adverse Events, version 5.0 (27 November 2017). Peripheral blood was collected to determine PGDM1400, PGT121 and VRC07-523LS serum levels, HIV sequencing and immunogenicity, among other research assessments outlined in the Study Protocol (Supplementary Information).

### ART counseling

Participants with HIV who were not on ART received ART counseling upon entering the study and 8 weeks after administration of the IP. Participants who had not initiated or made plans to initiate ART by the final study visit received ART counseling again at their final study visit.

### HIV-1 RNA levels and CD4^+^ T cell measurement

Plasma HIV-1 RNA levels were measured at BIDMC using the Roche COBAS AmpliPrep/COBAS TaqMan HIV-1 Test, version 2.0 (lower limit of quantification (LLoQ) = 23 RNA copies per ml), until it was replaced with the Hologic Aptima HIV-1 Quant Assay (LLoQ = 32 RNA copies per ml). HIV-1 VL was measured at OIC and HART using the Abbott Real-Time HIV-1 assay (LLoQ = 40 RNA copies per ml). VL assays were performed at LabCorp or at BIDMC. CD4^+^ T cell counts were measured using a clinical flow cytometry assay performed at LabCorp or at BIDMC.

### Determination of bNAb serum levels

(1) BAMA: PGT121, PGDM1400 and VRC07-523LS levels were determined on a Bio-Plex 200 system (Bio-Rad) that measures the ability of each monoclonal antibody to bind to their specific anti-idiotype antibody captured on fluorescent magnetic microspheres (MagPlex, Luminex), using a customized and standardized HIV-1 assay^32–35^. PGT121, PGDM1400 and VRC07-523LS concentrations were representative of three separate assays where each sample was run in duplicate. The standard curve consisted of PGT121 IgG monoclonal antibody, PGT121+PGDM1400 IgG monoclonal antibodies or PGT121+PGDM1400+VRC07-523LS IgG monoclonal antibodies, titrated in assay diluent, depending on subject group. The negative controls included an IgG1 monoclonal antibody (with specificity for an irrelevant antigen) and blank microspheres (uncoupled). Samples with bNAb concentrations below the LLoQ at a dilution of 1:50 were designated as the LLoQ value for plotting purposes. Samples with bNAb concentrations above the LLoQ at a 1:50 dilution were further tested at various dilution factors to obtain median fluorescent intensities (MFIs) in the linear range of the standard curve, and the resulting concentrations from the standard curve were averaged. Samples that were positive at or greater than the limit of detection (LOD) with an MFI that was three-fold over the pre-infusion MFI with a bNAb concentration less than the LLoQ were called positive. Samples with observed concentrations less than the LLoQ were called negative.

Limits of quantification for analytes by HIV serostatus

**Table:** 

Analyte	HIV serostatus	Group	LLoQ (µg ml^−1^)
PGDM1400	HIV-Uninfected	1A, 1B, 1C, 2A, 2B, 2C	0.240
	HIV-Infected	3A	0.107
		3B	0.270
PGT121	HIV-Uninfected	2A, 2B, 2C	0.500
	HIV-Infected	3A	0.310
		3B	0.710
VRC07-523LS	HIV-Infected	3A	0.088

(2) TZM-bl neutralization assay: Neutralizing antibodies against HIV-1 were measured as a function of reduction in Tat-regulated luciferase (Luc) reporter gene expression in TZM-bl cells^36–39^. For participants in groups 1, 2 and 3B, neutralization titers were measured in pre-infusion and post-infusion immune sera against both viruses 6545.v4.c1 and THRO4156.18 (PGDM1400 sensitive and PGT121 resistant) and viruses X2088_c9 and CNE30 (PGDM1400 resistant and PGT121 sensitive). For participants in group 3A (triple bnAb combination), serum samples were tested using viruses 6540.v4.c1 (PGDM1400 sensitive, PGT121 and VRC07-523LS resistant), CH505TF.N334S.N160A.N280D.1 (PGT121 sensitive and VRC07-523LS and PGDM1400 resistant) and CAP220.2.00_A8_5B (VRC07-523LS sensitive and PGDM1400 and PGT121 resistant). MuLV was used as a negative control virus for all participant samples. Titers ranged from a minimum of 1:20 to a maximum of 1:1,562,500, with values outside of this range considered censored. The median IC_50_ titer of bNAbs against the specific indicator viruses used the clinical drug products tested at a primary concentration of 10 μg ml^−1^ with five-fold dilution series and were included in each individual assay setup. The estimated serum concentration for each individual bNAb was calculated as the serum ID_50_ titer × bNAb IC_50_ titer (μg ml^−1^).

### HIV-1 *env* gene sequencing and production of pseudoviruses

SGA assays were performed by isolating HIV-1 RNA and reverse transcribing to viral cDNA^40^. First-round polymerse chain reaction (PCR) was carried out with Platinum PCR Mix High-Fidelity (Invitrogen) together with HIV B primers listed in Supplementary Table 10. Amplicons from cDNA dilutions resulting in less than 30% positive wells were considered to result from amplification of a single cDNA amplification according to the Poisson distribution and were processed for sequencing. For each sample, 10–30 sequences were analyzed. Selected viral sequences that were isolated from the plasma of each participant by SGA were used to generate CMV-promoter-based pseudoviruses^37^.

### Endpoints

The primary endpoints for safety and tolerability were as follows: (1) proportion of participants with moderate or greater reactogenicity (for example, solicited AEs) for 3 days after intravenous infusion of PGDM1400 alone, a combination of PGDM1400 and PGT121 bNAbs and a combination of PGDM1400 and PGT121 and VRC07-523LS; (2) proportion of participants with moderate or greater and/or PGDM1400- and PGT121- and VRC07-523LS bNAb-related unsolicited AEs, including safety laboratory (biochemical and hematological) parameters after intravenous infusion of PGDM1400 and/or PGT121 and/or VRC07-523LS for the first 56 days after administration of the IP; and (3) proportion of participants with PGDM1400- and/or PGT121- and/or VRC07-523LS-related SAEs throughout the study period. The primary endpoints, for pharmacokinetics, were elimination *t*_1/2_, clearance (CL/F), volume of distribution (Vz/F), area under the concentration decay curve (AUC) and effect of HIV RNA levels on PGDM1400 and/or PGT121 and/or VRC07-523LS disposition (elimination *t*_1/2_), CL/F, Vz/F and total exposure. The primary endpoint for antiviral activity among viremic participants with HIV was the change in plasma HIV-1 RNA levels from baseline (mean of pre-entry and entry values). The secondary endpoints were change in CD4^+^ T cell count and frequency compared to baseline as measured by single-platform flow cytometry and development of HIV-1 sequence variations in epitopes known to result in reduced PGDM1400 and/or PGT121 and/or VRC07-523LS neutralization susceptibility. The primary endpoints for safety, tolerability and pharmacokinetics were changed in Protocol Version 4.0 to include the VRC07-523LS monoclonal antibody for the subgroup 3A (see Supplementary Information for a summary of all protocol changes).

### Sample size and statistical analysis

(1) The sample size for safety and tolerability analysis was 30–66 participants according to the dose-escalation design used to characterize the safety profile of one intravenous infusion of PGDM1400 monoclonal antibody ± PGT121 monoclonal antibody, at one of three dose levels. For life-threatening AEs related to active product: if none of the nine (maximum 18) participants in either group 1 or group 2 who receive the active product experience such reactions, then the exact 95% upper confidence bound for the rate of these AEs in the population is 33.6% (or 18.5% if *n* = 18). This was an exploratory proof-of-concept trial; the analysis was descriptive; and no formal null hypothesis was tested. The frequency of moderate or greater reactogenicity events was determined and compared among groups. The frequency of SAEs judged possibly, probably or related to the IP was determined. All AEs were analyzed and grouped by seriousness, severity and relationship to the IP (as judged by the investigators). An interim safety analysis of group data was carried out after each dose escalation according to the study schema without unblinding the study to investigators or participants. At the end of the study, a full analysis was prepared. Missing data were excluded from the statistical analysis. (2) The sample size for pharmacokinetic analysis was three per dose subgroup, sufficient for the planned analyses based on previous experience with PGT121 pharmacokinetics^18^. The data were fit to standard two-compartment population models using the Stochastic Approximation Expectation–Maximization (SAEM) estimation method in Monolix (version 2019R1, Lixoft SAS, 2019). Population (non-linear mixed effects) pharmacokinetic (popPK) models were fit separately by analyte and HIV infection status. Fixed effects were used to model the population-level pharmacokinetic parameters, and random effects were used to model the individual-level variability. The AUC was estimated by calculating the integral of the predicted concentration–time curve from the first infusion time to infinity. Additionally, peak concentration (C_max_) was computed as the maximum observed concentration. Summary descriptive results of pharmacokinetic parameters, including AUC, C_max_, *t*_1/2_ and CL/F results, were reported by bNAb and dose cohort. For each analyte, a Spearman correlation test was conducted to test for correlation among elimination *t*_1/2_, CL/F, Vz/F and dose- and weight-adjusted AUC with log_10_ VL at baseline (null hypothesis: *ρ* = 0; α = 0.05) using mid-ranks for tied scores and the approximate distribution^41^. Correlation between pharmacokinetic and reported safety and pharmacodynamic outcomes were also explored parameters to examine exposure–effect relationships. The concordance correlation coefficient (CCC)^42^ was used to assess the concordance between the log_10_ concentrations from the binding and neutralizing antibody assays. (3) The sample size for virologic analysis was 6–18 participants across groups 3A and 3B. No placebo participants were enrolled in part 2 as per study design. For each participant, VL difference-from-baseline was defined as the difference in day 7 log_10_ plasma HIV-1 RNA levels from baseline (mean, on log_10_ scale, of screening and day 0 levels). Based on a simulation study, power to reject the null hypothesis was 80% when the responder group has a difference-from-baseline VL drop of approximately 1.8 logs for a nominal α level of 0.05. As the study under-enrolled for group 3, the virologic outcome was not formally analyzed.

### Sequence analyses

*Env* gene sequences were extracted and codon-aligned using the webtool Gene Cutter on the Los Alamos HIV database (https://www.hiv.lanl.gov/content/sequence/GENE_CUTTER/cutter.html). Alignments were further refined manually. The bNAb resistance mutations (Fig. 3 and Extended Data Figs. 3–5) were identified using signature sites defined in Bricault et al.^15^ and comparison of baseline and rebound *Env* sequences at these sites together with matched bNAb neutralization data. For some *Env*s, canonical resistance signatures were not found despite bNAb resistance, and putative mutations underlying such resistance were identified by manual inspection of resistant/sensitive sequences together with information on proximity to bNAb epitopes. Highlighter plots (Fig. 3, center) were generated using the Highlighter webtool on the Los Alamos HIV database (https://www.hiv.lanl.gov/content/sequence/HIGHLIGHT/highlighter_top.html). The phylogenetic tree for all participant viruses combined (Fig. 4) was inferred using *Env* nucleotide alignments using the IQ-TREE algorithm as implemented on the Los Alamos HIV Database (https://www.hiv.lanl.gov/content/sequence/IQTREE/iqtree.html) using the default GTR model with site-wise rates and maximum likelihood optimization. Recombination analyses were performed using RAPR on the Los Alamos HIV database (https://www.hiv.lanl.gov/content/sequence/RAP2017/rap.html); additional details are in the legend for Extended Data Fig. 6. Sequence logos (Extended Data Figs. 3–5) were obtained from the web tool AnalyzeAlign in the Los Alamos HIV database (https://www.hiv.lanl.gov/content/sequence/ANALYZEALIGN/analyze_align.html). Recombination analyses (Extended Data Fig. 6) were conducted using the LANL tool RAPR (https://www.hiv.lanl.gov/content/sequence/RAP2017/rap.html)^43^. Sequences are available at GenBank (see Supplementary Table 11 for accession numbers).

### Estimation of single bNAb and bNAb combination ID_80_ titers

For individual bNAbs, ID_80_ titers were calculated as (serum concentration of bNAb) / IC_80_. For baseline ID_80_ titers, day 7 bNAb concentrations were used. This was done to avoid the initially high bNAb concentrations in the serum immediately after infusion that rapidly decay as serum bNAbs seed tissues; this phase typically lasts 7 days. For rebound ID_80_ titers, the bNAb concentrations at the last HIV RNA level nadir time point were used, because this time point is the likely to be close to the time when rebound viruses start increasing in frequency. For bNAb combinations, ID_80_ titers were estimated as the factor by which the serum at a given time point with its composition of bNAbs will need to be diluted to give a predicted 80% neutralization in the pseudovirus neutralization assay. The fraction neutralization afforded by a given concentration profile for bNAbs in the serum was calculated using the Bliss–Hill model^44^ using serum concentration of bNAbs and individual bNAb IC_50_ and IC_80_ titers for each pseudovirus. Neutralizing activity (for example, IC_80_) of bNAb combinations against global heterologous viruses (Extended Data Fig. 8) was predicted using the Bliss–Hill model as implemented in the webtool CombiNAber (https://www.hiv.lanl.gov/content/sequence/COMBINABER/combinaber.html) using data on individual bNAbs as available in CATNAP^9^.

### Comparison to other studies

Individual bNAb concentrations and IC_50_ and IC_80_ titers for each baseline and rebound viruses were obtained from previous studies for 3BNC117+10-1074 (ref. ^4^), 3BNC117 (ref. ^3^) and VRC01 (ref. ^1^) therapy in viremic patients. For each study, only those participants who showed a clear decline in the HIV RNA levels upon bNAb infusion were used; these are shown in Extended Data Fig. 7. Individual bNAb and combination ID_80_ titers were calculated as mentioned above.

### Statistics

All group-based comparisons (Fig. 5 and Extended Data Fig. 7) were analyzed statistically using the Wilcoxon rank-sum test. Depending on the null hypothesis, one-sided or two-sided *P* values were obtained and are mentioned in the figure legends. RAPR, the web tool that was used for recombination analysis, employs the Wald–Wolfowitz Runs Test statistic, as described in Song et al.^43^.

### Important changes to methods after trial commencement

Protocol Version 4.0 added the VRC07-523LS monoclonal antibody to the study design in combination with PGDM1400 monoclonal antibody and PGT121 monoclonal antibody at 20 mg kg^−1^ each in viremic individuals with HIV not on ART (group 3A). These changes were made to allow safety, pharmacokinetic and antiviral activity evaluation of a triple bNAb combination, and primary, secondary and exploratory endpoints were updated accordingly. During this change, the number of participants in groups 3A and 3B was decreased from six (maximum 18) to three (maximum 9), thus changing the group 3 total from 12 (maximum 36) to six (maximum 18) and overall study total from 36 (maximum 84) to 30 (maximum 66). These changes were made to facilitate recruitment and with the approval of the SMC, the PSRT and the IRB.

### Reporting Summary

Further information on research design is available in the Nature Research Reporting Summary linked to this article.

## Online content

Any methods, additional references, Nature Research reporting summaries, source data, extended data, supplementary information, acknowledgements, peer review information; details of author contributions and competing interests; and statements of data and code availability are available at 10.1038/s41591-022-01815-1.

## Supplementary information

Supplementary Information Supplementary Tables 1–11, CONSORT 2010 checklist and protocol versions

Reporting Summary

## Data Availability

All viral sequences identified in this study are publicly available via GenBank (see Supplementary Table 11 for GenBank accession numbers). Comprehensive data on HIV genetic sequences and immunological epitopes used for analysis in this study are publicly available via Los Alamos National Laboratory (https://www.hiv.lanl.gov/content/index). Additional requests for access to the study data can be submitted to D.H.B. (dbarouch@bidmc.harvard.edu). Data containing protected health information or that may identify a participant are restricted, and, therefore, additional data requests must be reviewed before release.
