# Supplementary material for: Safety and antiviral activity of triple combination broadly neutralizing monoclonal antibody therapy against HIV-1: a phase 1 clinical trial
Source: Nat Med. 2022 May 12;28(6):1288–96. doi: 10.1038/s41591-022-01815-1 (PMC9205771; doi:10.1038/s41591-022-01815-1)
Supplement: Supplementary file 1 — Supplementary Tables 1–11, CONSORT 2010 checklist and protocol versions [file 41591_2022_1815_MOESM1_ESM.pdf]

nature portfolio

https://doi.org/10.1038/s41591-022-01815-1

Supplementary information

Safety and antiviral activity of triple combination broadly neutralizing monoclonal antibody therapy against HIV-1: a phase 1 clinical trial

In the format provided by the authors and unedited

Supplementary Information

Reaction	Max. Severity	Groups 1-2 Placebo	Groups 1A-1C PGDM1400			Groups 2A-2C PGDM1400 + PGT121			Group 3A PGDM1400 + PGT121 + VRC07- 523LS	Group 3B PGDM1400 + PGT121
		HIV uninfected							HIV infected	
		Placebo (N=6)	3 mg/kg IV (N=3)	10 mg/kg IV (N=3)	30 mg/kg IV (N=3)	3 mg/kg IV each (N=3)	10 mg/kg IV each (N=3)	30 mg/kg IV each (N=3)	20 mg/kg IV each (N=4)	30 mg/kg IV each (N=1)
Any Symptom (Local or Systemic)	Grade 1	3 (50.0)	1 (33.3)	1 (33.3)	0	2 (66.7)	2 (66.7)	2 (66.7)	0	0
	Grade 2	0	1 (33.3)	1 (33.3)	0	1 (33.3)	0	0	1 (25.0)	0
Any LocalSymptom	Grade 1	3 (50.0)	0	1 (33.3)	0	3 (100.0)	2 (66.7)	1 (33.3)	0	0
	Grade 2	0	0	0	0	0	0	0	0	0
Pain	Grade 1	0	0	1 (33.3)	0	0	1 (33.3)	1 (33.3)	0	0
	Grade 2	0	0	0	0	0	0	0	0	0
Tenderness	Grade 1	1 (16.7)	0	1 (33.3)	0	3 (100.0)	2 (66.7)	1 (33.3)	0	0
	Grade 2	0	0	0	0	0	0	0	0	0
Pruritus	Grade 1	1 (16.7)	0	0	0	0	0	0	0	0
	Grade 2	0	0	0	0	0	0	0	0	0
Erythema/SkinDiscoloration	Grade 1	1 (16.7)	0	0	0	0	0	0	0	0
	Grade 2	0	0	0	0	0	0	0	0	0
Swelling/ Hardening	Grade 1	1 (16.7)	0	0	0	0	0	1 (33.3)	0	0
	Grade 2	0	0	0	0	0	0	0	0	0
Any SystemicSymptom	Grade 1	0	1 (33.3)	0	0	0	0	2 (66.7)	0	0
	Grade 2	0	1 (33.3)	1 (33.3)	0	1 (33.3)	0	0	1 (25.0)	0
Chills	Grade 1	0	1 (33.3)	0	0	0	0	0	0	0
	Grade 2	0	0	0	0	0	0	0	0	0
Malaise	Grade 1	0	0	1 (33.3)	0	0	0	0	0	0
	Grade 2	0	1 (33.3)	0	0	0	0	0	0	0
Myalgia	Grade 1	0	0	0	0	0	0	1 (33.3)	0	0
	Grade 2	0	1 (33.3)	0	0	1 (33.3)	0	0	0	0
Arthralgia	Grade 1	0	0	0	0	0	0	0	0	0
	Grade 2	0	1 (33.3)	0	0	0	0	0	0	0
Headache	Grade 1	0	2 (66.7)	0	0	0	0	1 (33.3)	0	0
	Grade 2	0	0	1 (33.3)	0	0	0	0	1 (25.0)	0
Nausea	Grade 1	0	0	1 (33.3)	0	0	0	0	1 (25.0)	0
	Grade 2	0	0	0	0	0	0	0	0	0
Vomiting	Grade 1	0	0	0	0	0	0	0	0	0
	Grade 2	0	0	0	0	0	0	0	0	0
Maximum Temperature*	Grade 1	0	0	0	0	0	0	0	0	0
	Grade 2	0	0	0	0	0	0	0	0	0

* Temperatures from Physical Exam (PXV) and Reactogenicity Log Summary (RXA) tables (within 3 days of administration)
* Mild (>=38.0˚C), Moderate (>=38.6˚C), Severe (>=39.3˚C), Potentially Life-Threatening (>=40.0˚C)

Supplemental Table 1. Grade 1 or Greater Solicited Symptoms by Maximum Reported Severity

		Groups 1-2 Placebo	Groups 1A-1C PGDM1400			Groups 2A-2C PGDM1400 + PGT121			Group 3A PGDM1400 + PGT121 + VRC07- 523LS	Group 3B PGDM1400 + PGT121
		HIV uninfected							HIV infected	
	Max. Severity	Placebo (N=6)	3 mg/kg IV (N=3)	10 mg/kg IV (N=3)	30 mg/kg IV (N=3)	3 mg/kg IV each (N=3)	10 mg/kg IV each (N=3)	30 mg/kg IV each (N=3)	20 mg/kg IV each (N=4)	30 mg/kg IV each (N=1)
**Any unsolicited AE, n(%)**	Grade 1	2 (33.3)	3 (100)	1 (33.3)	1 (33.3)	1 (33.3)	0	1 (33.3)	2 (50.0)	0
	Grade 2	2 (33.3)	0	0	0	0	0	1 (33.3)	1 (25.0)	0
	Grade 3	0	0	0	0	1 (33.3)	0	0	0	0
**Infections and infestations, n(%)**	Grade 1	1 (16.7)	1 (33.3)	1 (33.3)	1 (33.3)	0	0	0	0	0
	Grade 2	0	0	0	0	0	0	1 (33.3)	1 (25.0)	0
Upper respiratory tract infection	Grade 1	1 (16.7)	1 (33.3)	1 (33.3)	1 (33.3)	0	0	0	0	0
ß-hem streptococcal infection	Grade 1	0	0	1 (33.3)	0	0	0	0	0	0
Genital herpes	Grade 2	0	0	0	0	0	0	0	1 (25.0)	0
Gingivitis	Grade 2	0	0	0	0	0	0	1 (33.3)	0	0
**Investigations, n(%)**	Grade 1	1 (16.7)	1 (33.3)	0	0	0	0	0	0	0
	Grade 2	1 (16.7)	0	0	0	0	0	0	0	0
	Grade 3	0	0	0	0	1 (33.3)	0	0	0	0
Blood creatine phosphokinase increased	Grade 2	1 (16.7)	0	0	0	0	0	0	0	0
	Grade 3	0	0	0	0	1 (33.3)	0	0	0	0
Platelet count decreased	Grade 1	1 (16.7)	0	0	0	0	0	0	0	0
White blood cells urine positive	Grade 1	0	1 (33.3)	0	0	0	0	0	0	0
**Gastrointestinal disorders, n(%)**	Grade 1	0	0	0	0	0	0	0	2 (50.0)	0
Abdominal pain lower	Grade 1	0	0	0	0	0	0	0	1 (25.0)	0
Abdominal pain upper	Grade 1	0	0	0	0	0	0	0	1 (25.0)	0
Diarrhea	Grade 1	0	0	0	0	0	0	0	1 (25.0)	0
**Injury, poisoning and procedural complications, n(%)**	Grade 1	0	0	1 (33.3)	0	0	0	0	0	0
	Grade 2	1 (16.7)	0	0	0	0	0	0	0	0
Contusion	Grade 1	0	0	1 (33.3)	0	0	0	0	0	0
Ligament sprain	Grade 2	1 (16.7)	0	0	0	0	0	0	0	0
**Blood and lymphatic system disorders, n(%)**	Grade 1	0	0	0	0	0	0	1 (33.3)	0	0
Neutropenia	Grade 1	0	0	0	0	0	0	1 (33.3)	0	0
**Ear and labyrinth disorders, n(%)**	Grade 1	0	0	0	0	1 (33.3)	0	0	0	0
Tinnitus	Grade 1	0	0	0	0	1 (33.3)	0	0	0	0
**General disorders and administration site conditions, n(%)**	Grade 1	1 (16.7)	0	0	0	0	0	0	0	0
Fatigue	Grade 1	1 (16.7)	0	0	0	0	0	0	0	0
**Renal and urinary disorders, n(%)**	Grade 1	0	1 (33.3)	0	0	0	0	0	0	0
Proteinuria	Grade 1	0	1 (33.3)	0	0	0	0	0	0	0
**Respiratory, thoracic and mediastinal disorders, n(%)**	Grade 1	0	1 (33.3)	0	0	0	0	0	0	0
Oropharyngeal pain	Grade 1	0	1 (33.3)	0	0	0	0	0	0	0
**Surgical and medical procedures, n (%)**	Grade 2	1 (16.7)	0	0	0	0	0	0	0	0
Prophylaxis against HIV infection	Grade 2	1 (16.7)	0	0	0	0	0	0	0	0

N = Total number of participants in the safety analysis population within each group

n = Number of participants who experienced at least one event (participants with >1 reported event is counted only once in each row)

% = Percentage of participants in each category relative to the total number within each group

Supplemental Table 2. Unsolicited AEs Through Study Day 56, By Group

		Groups 1-2 Placebo	Groups 1A-1C PGDM1400			Groups 2A-2C PGDM1400 + PGT121			Group 3A PGDM1400 + PGT121 + VRC07- 523LS	Group 3B PGDM1400 + PGT121
		HIV uninfected							HIV infected	
	Maximum Severity	Placebo (N=6)	3 mg/kg IV (N=3)	10 mg/kg IV (N=3)	30 mg/kg IV (N=3)	3 mg/kg IV each (N=3)	10 mg/kg IV each (N=3)	30 mg/kg IV each (N=3)	20 mg/kg IV each (N=4)	30 mg/kg IV each (N=1)
Any related[1], unsolicited AE, n (%)	Grade 1	0	1 (33.3)	0	0	0	0	0	0	0
Respiratory, thoracic and mediastinal disorders, n (%)	Grade 1	0	1 (33.3)	0	0	0	0	0	0	0
Oropharyngeal pain	Grade 1	0	1 (33.3)	0	0	0	0	0	0	0

1 "Related" AEs include those reported as possibly, probably, or definitely related to IP administrationN = Total number of participants in the safety analysis population within each group

n = Number of participants who experienced at least one event (participants with >1 reported event are counted only once in each row)

% = Percentage of participants in each category relative to the total number within each group

Supplemental Table 3. Related, Unsolicited AEs, by MedDRA SOC, PT, and Maximum Reported Severity Through Study Day 56

Groups 3 A and 3B								
		PGDM1400 + PGT121 + VRC07-523LS 20 mg/kg IV each				PGDM1400 + PGT121 30 mg/kg IV each		
		CD4 (%)		CD4 (cells/µL)				
**Study Day***	**N**	**Median (IQR)**	**Range**	**Median (IQR)**	**Range**	**N**	**CD4 (%)**	**CD4 (cells/µL)**
Screening	4	29.6 (25.2 - 36.6)	24.7 - 39.7	568 (505 - 649)	469 - 701	1	20.9	355
0	4	29.3 (25.9 - 34.1)	25.5 - 36.0	529 (505 - 561)	500 - 575	1	21.8	392
7	4	28.9 (23.9 - 33.7)	20.9 - 36.5	640 (570 - 737)	554 - 780	1	29.5	413
14	3	30.0 (26.1 - 36.0)	26.1 - 36.0	684 (679 - 930)	679 - 930	1	29.7	475
28	3	26.8 (25.1 - 36.9)	25.1 - 36.9	590 (443 - 602)	443 - 602	1	29.7	505
56	3	25.3 (20.2 - 32.1)	20.2 - 32.1	557 (263 - 578)	263 - 578	1	23.7	474
168	3	22.2 (19.6 - 42.8)	19.6 - 42.8	488 (294 - 685)	294 - 685	1	33.7	573

* Includes Scheduled Visits Only
For Group 3B, IQR and range were not applicable/displayed due to a sample size of 1.

Supplemental Table 4. CD4 counts in participants with HIV (Group 3A and B)

Parameter	Description	Estimate	95% CI	% RSE
**Fixed Effects**				
CL	Clearance	0.191	(0.177, 0.206)	3.799
V_c	Central volume	2.577	(2.232, 2.922)	6.826
Q	Inter-compartmental clearance	0.243	(0.113, 0.373)	27.265
V_p	Peripheral volume	1.736	(1.438, 2.034)	8.770
**Standard Deviation of the Random Effects**				
ω*CL*	SD, clearance	0.156	(0.104, 0.208)	17.054
ωV_c	SD, central volume	0.282	(0.188, 0.377)	17.116
ωQ	SD, Inter-compartmental clearance	1.072	(0.637, 1.508)	20.723
ωV_p	SD, peripheral volume	0.311	(0.16, 0.462)	24.822
**Correlation**				
$\rho V_c Cl$	Correlation between random effects for *Vc* and *CL*	0.781	(0.592, 0.971)	12.372
$\rho V_p Q$	Correlation between random effects for *Vp* and *Q*	0.855	(0.66, 1.05)	11.638
**Error Model Parameters**				
σ (*constant*)	SE, additive	0.227	(0.142, 0.313)	19.263
σ (*proportional*)	SE, proportional	0.114	(0.102, 0.127)	5.716

Note:
RSE: relative standard error; CI: confidence interval; SD: standard deviation.

Supplemental Table 5. PGDM1400 HIV-uninfected model: parameter estimates, corresponding 95% CIs of PK parameters, and % RSEs from a two-compartment population-level model.

	PGDM1400			PGT121			VRC07-523LS		
**Parameter**	**Estimate**	**95% CI**	**% RSE**	**Estimate**	**95% CI**	**% RSE**	**Estimate**	**95% CI**	**% RSE**
**Fixed Effects**									
CL	0.254	(0.207, 0.3)	9.370	0.421	(0.341, 0.501)	9.706	0.163	(0.126, 0.199)	11.419
V_c	2.054	(1.676, 2.431)	9.385	2.581	(2.362, 2.799)	4.314	2.040	(1.518, 2.562)	13.048
Q	0.107	(0.053, 0.161)	25.692	0.442	(0.284, 0.6)	18.235	0.410	(0.277, 0.544)	16.610
V_p	0.995	(0.835, 1.155)	8.195	3.146	(2.379, 3.914)	12.448	4.012	(3.549, 4.476)	5.897
**Standard Deviation of the Random Effects**									
ω*CL*	0.204	(0.075, 0.333)	32.340	0.206	(0.072, 0.34)	33.243	0.213	(0.05, 0.377)	39.026
ωV_c	0.201	(0.07, 0.333)	33.290	0.208	(0.033, 0.384)	42.934	0.238	(0.049, 0.428)	40.600
**Error Model Parameters**									
σ (*proportional*)	0.142	(0.116, 0.168)	9.258	0.197	(0.16, 0.234)	9.685	0.173	(0.138, 0.208)	10.410
RSE: relative standard error									

Supplemental Table 6. PGDM1400, PGT121 and VRC07-523LS HIV-Infected model: parameter estimates, corresponding 95% CIs of PK parameters, and % RSEs from a two-compartment population-level model.

	Distribution half-life			Elimination half-life		
	Median (days)	Min	Max	Median (days)	Min	Max
**HIV uninfected**						
PGDM1400	2.2	0.7	6.8	19.4	15.3	24.7
PGT121	1.1	1.0	1.9	20.2	15.8	27.8
**HIV infected**						
PGDM1400	3.1	1.9	4.9	11.0	10.1	18.7
PGT121	1.6	1.5	1.9	11.8	10.4	20.5
VRC07-523LS	1.9	1.6	2.3	29.3	27.4	37.9

Supplemental Table 7. PGDM1400, PGT121 and VRC07-523LS median, minimum, and maximum individual half-life estimates in participants without and with HIV, respectively.

Parameter	Median [Min, Max] Group 1 (n = 9)	Group 2 (n = 9)	P-value
CL	0.19 [0.17, 0.21]	0.20 [0.14, 0.27]	0.6048
Vz	5.72 [4.79, 6.35]	5.62 [3.42, 8.04]	0.6665
AUC_{ADJ}	398.07 [263.76, 507.73]	362.40 [301.49, 476.98]	0.7304
Elimination half-life	20.77 [17.76, 24.74]	17.43 [15.29, 24.09]	0.0503

Note:
ADJ: dose- and weight-adjusted.

Supplemental Table 8. Comparisons of PGDM1400 PK parameters by group among participants without HIV. Group 1 (pooled subgroups 1A, 1B, and 1C) participants received PGDM1400 alone. Group 2 (pooled subgroups 2A, 2B, and 2C) received PGDM1400 co-administered with PGT121. P-values were calculated using the two-sided Wilcoxon rank-sum test. Placebo participants were excluded.

Visit Number	Scheduled Day	Actual Day	Result	Viral Load (RNA copies/ml)
**693-1969 (PGDM1400+PGT121+VRC07-523LS, 20 mg/kg IV each)**				
01	Screening	-19	Detected	8850
02	0	0	Detected	2770
02A	1	1	Detected	3100
02B	2	2	Detected	2910
02C	3	3	Detected	1390
03	7	7	Detected	230
04	10	10	Detected	50
05	14	15	Detected	<40
06	21	21	Detected	50
07	28	28	Detected	<40
08	42	42	Not detected	
09	56	56	Detected	<40
10	70	71	Not detected	
11	84	85	Detected	410
12	112	116	N/A	N/A
13	140	140	Detected	1660
14	168	169	Not detected	
**693-2290 (PGDM1400 + PGT121 + VRC07-523LS, 20 mg/kg IV each)**				
01	Screening	-27	Detected	23160
02	0	0	Detected	15720
02A	1	1	Detected	13120
02B	2	2	Detected	13970
02C	3	3	Detected	6220
03	7	7	Detected	1050
07	28	29	Detected	150
**693-2215 (PGDM1400 + PGT121 + VRC07-523LS, 20 mg/kg IV each)**				
01	Screening	-19	Detected	9140
02	0	0	Detected	4340
02A	1	1	Detected	3670
02B	2	2	Detected	3470
02C	3	4	Detected	660
03	7	7	Detected	290
04	10	10	Detected	70
05	14	14	Detected	100
06	21	21	Detected	90
07	28	30	Detected	320
08	42	43	Detected	2600
09	56	60	Detected	2080
10	70	73	Detected	230
11	84	91	Detected	3260
12	112	113	Detected	2850
13	140	140	Detected	3330
14	168	168	Detected	3440

Visit Number	Scheduled Day	Actual Day	Result	Viral Load (RNA copies/ml)
**693-7989 (PGDM1400 + PGT121 + VRC07-523LS, 20 mg/kg IV each)**				
01	Screening	-20	Detected	83410
02	0	0	Detected	163130
02A	1	1	Detected	30890
02B	2	2	Detected	59510
02C	3	3	Detected	15860
03	7	8	Detected	520
04	10	10	Detected	530
05	14	14	Detected	570
06	21	21	Detected	3840
08	42	42	Detected	64300
09	56	53	Detected	48650
10	70	66	Detected	60
11	84	84	Not detected	
12	112	109	Detected	<40
13	140	143	Detected	<40
14	168	168	Detected	<40
**693-7312 (PGDM1400 + PGT121, 30 mg/kg IV each)**				
01	Screening	-18	Detected	74020
02	0	0	Detected	34720
02A	1	1	Detected	26200
02B	2	2	Detected	12370
02C	3	3	Detected	3960
03	7	6	Detected	510
04	10	10	Detected	730
05	14	14	Detected	2230
06	21	16	Detected	1500
07	28	30	Detected	11310
08	42	45	Detected	9410
09	56	56	Detected	6420
10	70	73	Detected	5200
11	84	87	Detected	13370
12	112	108	Detected	1400
13	140	143	Detected	<40
14	168	153	Not detected	

Supplemental Table 9. HIV RNA levels (Participants with HIV)

**SGA for HIV B**		
PTL-98	HIV B env out F	TAGAGCCCTGGAAGCATCCAGGAAG
PTL-99	HIV B env out R	TTGCTACTTGTGATTGCTCCATGT
PTL-100	HIV B env inn F	TTAGGCATCTCCTATGGCAGGAAGAAG
PTL-101	HIV B env inn R	GTCTCGAGATACTGCTCCCACCC

Supplemental Table 10: PCR Primers

BankIt2560767 693-7989_V2_A2.3_Env	ON014844
BankIt2560767 693-7989_V2_B4.4_Env	ON014845
BankIt2560767 693-7989_V2_B4.5_Env	ON014846
BankIt2560767 693-7989_V2_B5.3_Env	ON014847
BankIt2560767 693-7989_V2_B8.2_Env	ON014848
BankIt2560767 693-7989_V2_C1.4_Env	ON014849
BankIt2560767 693-7989_V2_C4.2_Env	ON014850
BankIt2560767 693-7989_V2_C4.4.1_Env	ON014851
BankIt2560767 693-7989_V2_C4.4.2_Env	ON014852
BankIt2560767 693-7989_V2_C10.1_Env	ON014853
BankIt2560767 693-7989_V2_C11.3_Env	ON014854
BankIt2560767 693-7989_V2_D4.4_Env	ON014855
BankIt2560767 693-7989_V2_D10.17_Env	ON014856
BankIt2560767 693-7989_V2_E10.4_Env	ON014857
BankIt2560767 693-7989_V2_F1.2_Env	ON014858
BankIt2560767 693-7989_V2_F7.3_Env	ON014859
BankIt2560767 693-7989_V2_F9.4_Env	ON014860
BankIt2560767 693-7989_V9_A4.1_Env	ON014861
BankIt2560767 693-7989_V9_A7.1_Env	ON014862
BankIt2560767 693-7989_V9_A8.1_Env	ON014863
BankIt2560767 693-7989_V9_A11.1_Env	ON014864
BankIt2560767 693-7989_V9_B1.1_Env	ON014865
BankIt2560767 693-7989_V9_B2.2_Env	ON014866
BankIt2560767 693-7989_V9_B8.14_Env	ON014867
BankIt2560767 693-7989_V9_C3.1_Env	ON014868
BankIt2560767 693-7989_V9_D9.1_Env	ON014869
BankIt2560767 693-7989_V9_D8.1_Env	ON014870
BankIt2560767 693-1969_V2_A2.3_Env	ON014871
BankIt2560767 693-1969_V2_A7.1_Env	ON014872
BankIt2560767 693-1969_V2_A8.1_Env	ON014873
BankIt2560767 693-1969_V2_A9.1_Env	ON014874
BankIt2560767 693-1969_V2_A11.1_Env	ON014875
BankIt2560767 693-1969_V2_C3.11_Env	ON014876
BankIt2560767 693-1969_V2_C9.3_Env	ON014877
BankIt2560767 693-1969_V2_C10.3_Env	ON014878
BankIt2560767 693-1969_V2_C11.8_Env	ON014879
BankIt2560767 693-1969_V2_D1.2_Env	ON014880
BankIt2560767 693-1969_V2_D2.3_Env	ON014881
BankIt2560767 693-1969_V2_D7.8_Env	ON014882
BankIt2560767 693-1969_V2_D8.3_Env	ON014883
BankIt2560767 693-1969_V11_A4.2_Env	ON014884

BankIt2560767 693-1969_V11_A7.1_Env	ON014885
BankIt2560767 693-1969_V11_A11.4_Env	ON014886
BankIt2560767 693-1969_V11_B1.7_Env	ON014887
BankIt2560767 693-1969_V11_B3.1_Env	ON014888
BankIt2560767 693-1969_V11_B5.2_Env	ON014889
BankIt2560767 693-1969_V11_B11.2_Env	ON014890
BankIt2560767 693-1969_V11_B12.1_Env	ON014891
BankIt2560767 693-1969_V11_C4.1_Env	ON014892
BankIt2560767 693-1969_V11_C8.1_Env	ON014893
BankIt2560767 693-1969_V11_C11.4_Env	ON014894
BankIt2560767 693-2215_V2_C1.2_Env	ON014895
BankIt2560767 693-2215_V2_A6.3_Env	ON014896
BankIt2560767 693-2215_V2_A8.2_Env	ON014897
BankIt2560767 693-2215_V9_G10.2_Env	ON014898
BankIt2560767 693-2215_V9_C11.1_Env	ON014899
BankIt2560767 693-2215_V2_D11.9_Env	ON014900
BankIt2560767 693-2215_V9_B1.1_Env	ON014901
BankIt2560767 693-2215_V2_F4.4_Env	ON014902
BankIt2560767 693-2215_V2_G10.3_Env	ON014903
BankIt2560767 693-2215_V2_F8.3_Env	ON014904
BankIt2560767 693-2215_V2_D7.1_Env	ON014905
BankIt2560767 693-2215_V9_A6.1_Env	ON014906
BankIt2560767 693-2215_V2_C2.4_Env	ON014907
BankIt2560767 693-2215_V9_D2.4_Env	ON014908
BankIt2560767 693-2215_V9_B9.1_Env	ON014909
BankIt2560767 693-2215_V9_G4.3_Env	ON014910
BankIt2560767 693-2215_V9_A7.1_Env	ON014911
BankIt2560767 693-2215_V9_D7.3_Env	ON014912
BankIt2560767 693-2215_V2_D7.2_Env	ON014913
BankIt2560767 693-2215_V9_B4.3_Env	ON014914
BankIt2560767 693-2290_V2_A2.3_Env	ON014915
BankIt2560767 693-2290_V2_A2.7_Env	ON014916
BankIt2560767 693-2290_V2_A8.6_Env	ON014917
BankIt2560767 693-2290_V2_A9.5_Env	ON014918
BankIt2560767 693-2290_V2_A11.1_Env	ON014919
BankIt2560767 693-2290_V2_A11.5_Env	ON014920
BankIt2560767 693-2290_V2_A12.10_Env	ON014921
BankIt2560767 693-2290_V2_B1.1_Env	ON014922
BankIt2560767 693-2290_V2_B11.5_Env	ON014923
BankIt2560767 693-2290_V2_B12.3_Env	ON014924
BankIt2560767 693-2290_V2_C2.9_Env	ON014925
BankIt2560767 693-7312_V2_A3.3_Env	ON014926

BankIt2560767 693-7312_V2_E3.3_Env	ON014927
BankIt2560767 693-7312_V2_A2.2_Env	ON014928
BankIt2560767 693-7312_V7_F9.1_Env	ON014929
BankIt2560767 693-7312_V7_D8.8_Env	ON014930
BankIt2560767 693-7312_V7_E8.4_Env	ON014931
BankIt2560767 693-7312_V7_E4.2_Env	ON014932
BankIt2560767 693-7312_V2_E4.3_Env	ON014933

Supplemental Table 11: GenBank Accession Numbers

CONSORT 2010 checklist of information to include when reporting a randomised trial*

Section/Topic	Item No	Checklist item	Reported on page No
**Title and abstract**			
	1a	Identification as a randomised trial in the title	1
	1b	Structured summary of trial design, methods, results, and conclusions (for specific guidance see CONSORT for abstracts)	3
**Introduction**			
Background and objectives	2a	Scientific background and explanation of rationale	4
	2b	Specific objectives or hypotheses	5
**Methods**			
Trial design	3a	Description of trial design (such as parallel, factorial) including allocation ratio	5, 21
	3b	Important changes to methods after trial commencement (such as eligibility criteria), with reasons	28
Participants	4a	Eligibility criteria for participants	21,22
	4b	Settings and locations where the data were collected	21,22
Interventions	5	The interventions for each group with sufficient details to allow replication, including how and when they were actually administered	22,23
Outcomes	6a	Completely defined pre-specified primary and secondary outcome measures, including how and when they were assessed	27,28
	6b	Any changes to trial outcomes after the trial commenced, with reasons	31
Sample size	7a	How sample size was determined	28
	7b	When applicable, explanation of any interim analyses and stopping guidelines	
Randomisation:			
Sequence generation	8a	Method used to generate the random allocation sequence	22
	8b	Type of randomisation; details of any restriction (such as blocking and block size)	22
Allocation concealment mechanism	9	Mechanism used to implement the random allocation sequence (such as sequentially numbered containers), describing any steps taken to conceal the sequence until interventions were assigned	22
Implementation	10	Who generated the random allocation sequence, who enrolled participants, and who assigned participants to interventions	22
Blinding	11a	If done, who was blinded after assignment to interventions (for example, participants, care providers, those	22

		assessing outcomes) and how	
	11b	If relevant, description of the similarity of interventions	
Statistical methods	12a	Statistical methods used to compare groups for primary and secondary outcomes	28, 31
	12b	Methods for additional analyses, such as subgroup analyses and adjusted analyses	28
**Results**			
Participant flow (a diagram is strongly recommended)	13a	For each group, the numbers of participants who were randomly assigned, received intended treatment, and were analysed for the primary outcome	5
	13b	For each group, losses and exclusions after randomisation, together with reasons	5,7
Recruitment	14a	Dates defining the periods of recruitment and follow-up	5,6
	14b	Why the trial ended or was stopped	
Baseline data	15	A table showing baseline demographic and clinical characteristics for each group	Table 1
Numbers analysed	16	For each group, number of participants (denominator) included in each analysis and whether the analysis was by original assigned groups	5-7
Outcomes and estimation	17a	For each primary and secondary outcome, results for each group, and the estimated effect size and its precision (such as 95% confidence interval)	5-7
	17b	For binary outcomes, presentation of both absolute and relative effect sizes is recommended	
Ancillary analyses	18	Results of any other analyses performed, including subgroup analyses and adjusted analyses, distinguishing pre-specified from exploratory	8-12
Harms	19	All important harms or unintended effects in each group (for specific guidance see CONSORT for harms)	N/A
**Discussion**			
Limitations	20	Trial limitations, addressing sources of potential bias, imprecision, and, if relevant, multiplicity of analyses	15
Generalisability	21	Generalisability (external validity, applicability) of the trial findings	12-15
Interpretation	22	Interpretation consistent with results, balancing benefits and harms, and considering other relevant evidence	12-15
**Other information**			
Registration	23	Registration number and name of trial registry	3
Protocol	24	Where the full trial protocol can be accessed, if available	Supplemental Information
Funding	25	Sources of funding and other support (such as supply of drugs), role of funders	15-16

*We strongly recommend reading this statement in conjunction with the CONSORT 2010 Explanation and Elaboration for important clarifications on all the items. If relevant, we also recommend reading CONSORT extensions for cluster randomised trials, non-inferiority and equivalence trials, non-pharmacological treatments, herbal interventions, and pragmatic trials. Additional extensions are forthcoming: for those and for up to date references relevant to this checklist, see www.consort-statement.org.

**Protocol Title:**	A Phase 1 Randomized Placebo-controlled Clinical Trial of the Safety, Pharmacokinetics and Antiviral Activity of PGDM1400 and PGT121 Monoclonal Antibodies in HIV-uninfected and HIV-infected Adults
**Protocol Number:**	IAVI T002
**Regulatory Investigational Product Number**	TBD
**ClinicalTrials.gov Registry Number**	TBD
**Phase:**	Phase 1
**Sponsor:**	International AIDS Vaccine Initiative (IAVI)<br>125 Broad Street, 9th Floor<br>New York, New York 10004<br>USA
**Sponsor Status**	Not for-Profit Organization
**Date of Protocol Version:**	12 MAY 2017<br>1.0<br>IND Submission

THE CONFIDENTIAL INFORMATION IN THIS DOCUMENT IS PROVIDED TO YOU AS AN INVESTIGATOR, POTENTIAL INVESTIGATOR, OR CONSULTANT, FOR REVIEW BY YOU, YOUR STAFF, AND APPLICABLE INSTITUTIONAL REVIEW BOARDS (IRBS) AND/OR INDEPENDENT ETHICS COMMITTEES (IECS). IT IS UNDERSTOOD THAT THE INFORMATION WILL NOT BE DISCLOSED TO OTHERS, EXCEPT TO THE EXTENT NECESSARY TO OBTAIN ETHICAL AND REGULATORY APPROVAL FROM THE RESPECTIVE COMMITTEE'S AGENCIES AND INFORMED CONSENT FROM THOSE PERSONS TO WHOM THE INVESTIGATIONAL PRODUCT MAY BE ADMINISTERED.

PROTOCOL SYNOPSIS

**TITLE**	A Phase 1 Randomized Placebo-controlled Clinical Trial of the Safety, Pharmacokinetics and Antiviral Activity of PGDM1400 and PGT121 Monoclonal Antibodies in HIV-uninfected and HIV-infected Adults
**PROTOCOL NUMBER**	IAVI T002
**CLINICAL TRIAL PHASE**	Phase 1
**IND SPONSOR**	International AIDS Vaccine Initiative (IAVI)<br>125 Broad Street, 9th Floor<br>New York, New York 10004,<br>USA
**SPONSOR STATUS**	Not for Profit Organization
**SAMPLE SIZE**	36-84
**STUDY POPULATION**	1. HIV-uninfected males or females aged 18-50 years old who are willing to maintain low risk behavior for HIV infection; principal exclusion criteria include confirmed HIV-infection, pregnancy or lactation, significant acute or chronic disease and clinically significant laboratory abnormalities (Groups 1 and 2, see below for full inclusion and exclusion criteria).<br>2. HIV-infected males or females aged 18-65 years old, not on antiretroviral therapy (ART) with HIV-1 viral load between 1000 and 100,000 copies/ml, CD4 cell count ≥ 300 cells/µl; principal exclusion criteria include significant acute or chronic medical condition other than HIV infection, and clinically significant laboratory abnormalities (Group 3, see below for full inclusion and exclusion criteria).
**STUDY DESIGN**	Double blind, randomized, placebo-controlled study for Groups 1 and 2. Open label study for Group 3. Single intravenous (IV) infusion of PGDM1400 mAb alone @ 3, 10 or 30 mg/kg, or a combination of PGDM1400 mAb and PGT121 mAb, each @ 3, 10 or 30 mg/kg, or placebo. See study design table below for details.
**STUDY DURATION**	Up to 32 weeks per participant, screening up to 42 (HIV-infected) or up to 56 (HIV-uninfected) days before single IV infusion of investigational product on day 0, and 24 weeks of follow up.
**INVESTIGATIONAL PRODUCT DESCRIPTION**	**PGDM1400 mAb**: PGDM1400 mAb is a recombinant, fully human monoclonal antibody (mAb) of the IgG1 isotype that binds to the HIV envelope. The concentration of the product is 50 mg/ml. Vials will contain 6 ml of product @ 50 mg/ml = 300 mg.<br>**PGT121 mAb**: PGT121 mAb is a recombinant, fully human monoclonal antibody (mAb) of the IgG1 isotype that binds to the

	HIV envelope. The concentration of the product is 50 mg/ml. Vials will contain 6 ml of product @ 50 mg/ml = 300 mg.<br>**Placebo:** 0.9% Sodium Chloride for injection, USP
**OBJECTIVES**	**Primary Objectives**<br>• To evaluate the safety and tolerability of IV infusion of PGDM1400 mAb alone, and a combination of PGDM1400 mAb and PGT121 mAb in HIV-uninfected adults and HIV-infected adults<br>• To evaluate the pharmacokinetic (PK) profile of IV infusion of PGDM1400 mAb alone, and a combination of PGDM1400 mAb and PGT121 mAb in HIV-uninfected adults and HIV-infected adults<br>• To evaluate the antiviral activity of IV infusion of PGDM1400 mAb alone, and a combination of PGDM1400 mAb and PGT121 mAb in HIV-infected adults not on ART<br><br>**Secondary Objectives**<br>• To determine if PGDM1400 mAb alone, and in combination with PGT121 mAb induces anti-PGDM1400 and/or anti-PGT121 antibodies<br>• To determine the effect of PGDM1400 mAb alone and in combination with PGT121 mAb on CD4+ T cell counts in HIV-infected adults<br>• To determine the effect of PGDM1400 mAb alone and in combination with PGT121 mAb on viral escape in viremic HIV-infected adults not on ART<br><br>**Exploratory Objectives**<br>• To determine if PGDM1400 ± PGT121 mAb has any impact on the host immune responses (i.e., HIV-specific cellular and humoral immune response).<br>• To determine the effect of PGDM1400 ± PGT121 mAb on the size of the latent HIV reservoir in HIV-infected adults.<br>• To determine PGDM1400 mAb ± PGT121 mAb levels in mucosal secretions in HIV-uninfected and HIV-infected adults.<br>• To measure in vitro neutralization of HIV isolates with participant's serum post PGDM1400 ± PGT121 mAb IV infusion
**ENDPOINTS**	**Primary Endpoints**<br>*Safety and Tolerability*<br>1. Proportion of participants with moderate or greater reactogenicity (e.g., solicited adverse events) for 3 days following IV infusion of PGDM1400 mAb alone, and a combination of PGDM1400 mAb and PGT121 mAb.<br>2. Proportion of participants with adverse events (AEs), including safety laboratory (biochemical, hematological) parameters, during the first 56 days following IV infusion of PGDM1400

mAb alone and a combination of PGDM1400 mAb and PGT121 mAb, that are moderate or greater, and/or related to PGDM1400 mAb or PGT121 mAb.

3. Proportion of participants with serious adverse events (SAEs) throughout the study period following IV infusion of PGDM1400 mAb alone and a combination of PGDM1400 mAb and PGT121 mAb, that are related to PGDM1400 mAb or PGT121 mAb.

Pharmacokinetics

Pharmacokinetics following IV infusion of PGDM1400 mAb alone or a combination of PGDM1400 mAb and PGT121 mAb in HIV-uninfected and HIV-infected adults:

- Elimination half-life (t1/2)
- Clearance (CL/F)
- Volume of distribution (Vz/F)
- Area under the concentration decay curve (AUC)
- Impact of viral load and/or ART on PGDM1400 mAb and PGT121 mAb disposition (elimination half-life (t1/2), clearance (CL/F), volume of distribution (Vz/F), total exposure)

Antiviral activity

Antiviral activity following IV infusion of PGDM1400 mAb alone or in combination with PGT121 mAb in viremic HIV-infected adults not on ART:

1. Change in plasma HIV-1 RNA levels from baseline (mean of pre-entry and entry values)

Secondary Endpoints

Anti-PGDM1400 and anti-PGT121 antibodies

1. Serum anti-PGDM1400 antibody titers
2. Serum anti-PGT121 antibody titers

CD4+ T cell count

We will calculate the following endpoint to determine if IV infusion of PGDM1400 mAb alone or in combination with PGT121 mAb has any impact on CD4+ T cell counts in HIV-infected adults:

1. Change in CD4+ T cell count and frequency compared to baseline as measured by single platform flow cytometry.

HIV genotyping/phenotyping of circulating virus for evaluation of PGDM1400 mAb and/or PGT121 mAb -induced escape mutations

We will compare plasma virus genotype and phenotypic activity before and after IV infusion of PGDM1400 mAb alone or in combination with PGT121 mAb to determine the effect of PGDM1400 mAb and/or PGT121 mAb on viral escape mutations in viremic HIV-infected adults not on ART.

1. Genotypic analysis: Development of sequence variations in

epitopes known to result in reduced PGDM1400 mAb and/or PGT121 mAb neutralization susceptibility or known to cause resistance to antiretroviral drugs.
2. Phenotypic analysis: Changes in viral susceptibility to PGDM1400 mAb and/or PGT121 mAb neutralization.

Exploratory Endpoints
Additional assessments may include but are not limited to the following: HIV-specific IgG/IgA binding responses by ELISA, HIV-specific cellular immune responses by ELISPOT, HIV-specific antibody function by ADCC, ADCP, and ADCVI assays, PGDM1400 mAb and/or PGT121 mAb levels in mucosal secretions, changes in total HIV-1 DNA and 2-long terminal repeat (LTR) circular HIV-1 DNA in resting or total CD4 T cells and in vitro neutralization of HIV isolates with participant's serum post IV infusion of PGDM1400 mAb ± PGT121 mAb.

STUDY DESIGN TABLE

	Group	Participants	Sub-Group	Regimen	N	Dose (mg/kg)
Part 1 – MTD	1	HIV-uninfected participants	1A	PGDM1400/Placebo	3/1 (6/2 if DLT)	3 IV
			1B	PGDM1400/Placebo	3/1 (6/2 if DLT)	10 IV
			1C	PGDM1400/Placebo	3/1 (6/2 if DLT)	30 IV
				Total Group 1	9/3 = 12 (max 18/6 = 24 if DLT)	
	2	HIV-uninfected participants	2A	PGDM1400 + PGT121/Placebo	3/1 (6/2 if DLT)	3 + 3 IV
			2B	PGDM1400 + PGT121/Placebo	3/1 (6/2 if DLT)	10 + 10 IV
			2C	PGDM1400 + PGT121/Placebo	3/1 (6/2 if DLT)	30 + 30 IV
				Total Group 2	9/3 = 12 (max 18/6 = 24 if DLT)	
				Total Groups 1 and 2	18/6 = 24 (max 36/12 = 48 if DLT)	
Safety Monitoring Committee						
Part 2 – antiviral effect	3	HIV-infected off ART (VL $1x10^3$ – $1x10^5$ copies/ml)	3A	PGDM1400	6 (max 18)	MTD IV
			3B	PGDM1400+PGT121	6 (max 18)	MTD IV
				Total Group 3	12 (max 36)	
				Total entire study	36 (max 84)	

DLT, dose limiting toxicity; MTD, maximum tolerated dose

<table>
<tr><td>SAFETY MONITORING</td><td>Safety will continually be monitored by the Investigators, the Sponsor’s Medical Monitor and a Protocol Safety Review Team (PSRT). Safety data will be reviewed by an independent Safety Monitoring Committee (SMC).

- Sentinel participants in Groups 1 and 2
For each dose Subgroup in Groups 1 and 2, the 1st 2 participants will be sentinel participants for whom investigational product infusion will be separated by at least 24 hours contingent on review of safety information prior to infusion of the next sentinel participant. Both sentinel participants are observed for at least 24 hours before IV infusion of investigational product to the 3rd and subsequent participants.

- Dose limiting toxicity in Groups 1 and 2
Dose limiting toxicity (DLT) will be defined as 1) any Grade 3 or greater adverse event considered possibly, probably or definitely related to investigational product or 2) any Grade 3 or greater reactogenicity or 3) any SAE considered possibly, probably or definitely related to investigational product.

- Infusion related reactions
Infusion related reactions, including cytokine release syndrome, will be graded using the NCI Common Terminology Criteria for Adverse Events (CTCAE) version 4.03 (June 14, 2010). All other adverse events will be graded using the Division of AIDS (DAIDS) Table for Grading the Severity of Adult and Pediatric Adverse Events version 2.0 (November 2014).</td></tr>
<tr><td>DOSE ESCALATION IN GROUPS 1 AND 2</td><td>Part 1 of this study is a dose-escalation trial in HIV-uninfected adults (Groups 1 and 2) to establish the MTD of PGDM1400 mAb and a combination of PGDM1400 mAb and PGT121 mAb.

- PSRT review for dose escalation
The Protocol Safety Review Team (PSRT) will review safety data through day 14 post-investigational product infusion for all participants in each dose Subgroup for PGDM1400 mAb alone (Group 1) prior to allowing enrolment of participants into the next higher dose Subgroup in Group 1 or the same dose level PGDM1400 mAb and PGT121 mAb combination dose Subgroup (Group 2).
o If no DLT occurs in the initial 4 participants of a dose Subgroup (e.g., 1A), the study can proceed with enrolment of the next dose Subgroup for PGDM1400 mAb alone (e.g., 1B), and the same dose level PGDM1400 mAb and PGT121 mAb combination dose Subgroup (e.g., 2A).
o If 1 DLT occurs in the initial 4 participants of a dose Subgroup (e.g., 1A), 4 additional participants will be enrolled in the same dose Subgroup.
▪ If no additional DLTs occur within 14 days of infusion in</td></tr>
</table>

	the 8 total participants, the study can proceed with enrolment of the next dose Subgroup for PGDM1400 mAb alone (e.g., 1B), and the same dose level PGDM1400 mAb and PGT121 mAb combination dose Subgroup (e.g., 2A).<br>▪ In Subgroups receiving PGDM1400 alone (1A, 1B, 1C), if 2 or more DLTs accumulate in a dose Subgroup (e.g., 1B) that are the same, similar, or in the same System Organ Class, infusion will be halted and the next lower dose level will be declared the maximum tolerated dose (MTD) within this Group (e.g., 3 mg/kg if the DLTs occurred in dose Subgroup 1B @ 10 mg/kg) , and the same dose level PGDM1400 mAb and PGT121 mAb combination dose Subgroup (2A in this example). Group 2 will not proceed with escalation beyond the MTDs of PGDM1400 alone and PGT121 alone.<br>▪ If no DLT occurs in the final dose Subgroup (1C) after 14 days of follow-up, the MTD for PGDM1400 alone will be the highest dose given (30mg/kg).<br>▪ In Subgroups receiving PGDM1400 and PGT121 (2A, 2B, 2C), if 2 or more DLTs occur in a dose Subgroup (e.g., 2B) that are the same, similar, or in the same System Organ Class, infusion will be halted and the next lower dose level will be declared the MTD for the combination of PGDM1400 and PGT121.<br>▪ If no DLT occurs in the final dose Subgroup (2C) after 14 days of follow-up , MTD for PGDM1400 and PGT121 will be the highest dose given (30mg/kg each).<br><br>- *SMC review to determine MTD of PGDM1400 alone*<br>Following IV infusion of investigational product in the last participant in Group 1, an independent Safety Monitoring Committee (SMC) will review safety data through day 14 post-investigational product infusion for all participants to confirm MTD of PGDM1400 alone, and determine whether, and at what dose, Group 3A can initiate enrolment.<br><br>- *SMC review to determine MTD of the combination of PGDM1400 and PGT121*<br>Following IV infusion of investigational product in the last participant in Group 2, the SMC will review safety data through day 14 post-investigational product infusion for all participants to confirm MTD of the combination of PGDM1400 and PGT121, and determine whether Group 3B can initiate enrollment.
**ANTIVIRAL ACTIVITY IN GROUP 3**	Part 2 of this study will establish the antiviral effect of PGDM1400 mAb alone or the combination of PGDM1400 mAb plus PGT121 mAb in HIV infected adults not on ART (Group 3).<br><br>- *MTD determines dose*

	Group 3 will start with the MTD of PGDM1400 as determined by the SMC; e.g., if the MTD for PGDM1400 is 30mg/kg then Subgroup 3A will receive 30mg/kg and Subgroup 3B will receive PGDM1400 mAb + PGT121 mAb at a dose that combines the MTD for PGDM1400 mAb and the MTD for PGT121 mAb, respectively.
**PAUSE RULES**	The study will be paused for a safety review by the investigators and the independent SMC if:<br>1. 1 or more participants experience a Serious Adverse Event that is judged possibly, probably or definitely related to the investigational product.<br>2. There is a participant death regardless of relationship to the investigational product.<br>3. If 2 or more participants experience Grade 3 adverse events in the same System Organ Class that are considered possibly, probably, or definitely related to investigational product.<br>4. Any Grade 4 adverse event that is considered possibly, probably or definitely related to investigational product.
**EVALUATION FOR INTERCURRENT HIV INFECTION:**	Participants in Groups 1 and 2 (HIV-uninfected) will be tested for HIV according to the Schedule of Procedures. Test results will be interpreted according to a pre-determined diagnostic algorithm. HIV testing at additional time points may be performed upon the request of the participant and Principal Investigator or designee as medical or social circumstances warrant.
**INCLUSION CRITERIA**	**Inclusion criteria for all participants:**<br>1. Willing to comply with the requirements of the protocol and available for follow-up for the planned duration of the study.<br>2. In the opinion of the Principal Investigator or designee and based on Assessment of Informed Consent Understanding results, has understood the information provided and potential impact and/or risks linked to IV infusion and participation in the trial; written informed consent will be obtained from the participant before any study-related procedures are performed.<br>3. All heterosexually active female participants must commit to use an effective method of contraception for 3 months following investigational product administration, including:<br>a. Condoms (male or female) with or without spermicide<br>b. Diaphragm or cervical cap with spermicide<br>c. Intrauterine device, or contraceptive implant<br>d. Hormonal contraception<br>e. Successful vasectomy in the male partner (considered successful if a woman reports that a male partner has [1] documentation of azoospermia by microscopy (< 1 year ago), or [2] a vasectomy more than 2 years ago with no resultant pregnancy despite sexual activity post-vasectomy)<br>f. Not be of reproductive potential, such as having undergone

	hysterectomy, bilateral oophorectomy, or tubal ligation, postmenopausal (> 45 years of age with amenorrhea for at least 2 years, or any age with amenorrhea for at least 6 months and a serum follicle stimulating hormone [FSH] level > 40 IU/L); surgically sterile: no additional contraception required.<br>Women, who are not heterosexually active at screening, must agree to utilize an effective method of contraception if they become hetero-sexually active, as outlined above.<br>4. All sexually active males, regardless of reproductive potential, must be willing to consistently use an effective method of contraception (such as consistent male condoms with male and/or female partners) from the day of investigational product administration until at least 3 months following investigational product administration to avoid exposure of partners to investigational product in ejaculate, and to prevent conception with female partners.<br>5. All female participants must be willing to undergo urine pregnancy tests at time points indicated in the Schedule of Procedures and must test negative prior to investigational product administration.<br>6. A female participant must agree not to donate eggs (ova, oocytes) for the purpose of assisted reproduction until 3 months after investigational product administration. A man must agree not to donate sperm until 3 months after investigational product administration.<br>7. Willing to forgo donations of blood and/or any other tissues, including bone marrow, during the study and, for those HIV-uninfected participants who test HIV-positive due to investigational product administration, until the anti-HIV antibody titers become undetectable.<br>**Specific inclusion criteria for HIV-uninfected participants (Groups 1 and 2):**<br>8. At least 18 years of age on the day of screening and has not reached his or her 51st birthday on the day of signing the Informed Consent Document.<br>9. Willing to undergo HIV testing, risk reduction counselling and receive HIV test results.<br>10. Low risk for HIV infection and willing to maintain low-risk behavior for the duration of the trial.<br>11. Healthy male or female, as assessed by a medical history, physical exam, and laboratory tests.<br>**Specific inclusion criteria for HIV-infected participants (Group 3):**<br>12. At least 18 years of age on the day of screening and has not reached his or her 66th birthday on the day of signing the Informed Consent Document.<br>13. Confirmed HIV-1 infection (HIV Ab+ or HIV RNA+) by documentation in the medical records or in-clinic HIV testing;<br>14. CD4 ≥ 300 cells/μl.

	15. Not receiving cART, and (after appropriate counselling) willing to defer cART treatment for at least 56 days after administration of investigational product.<br>16. HIV-1 viral load between 1000–100,000 copies/ml, confirmed at screening.
**EXCLUSION CRITERIA**	**Exclusion criteria for all participants:**<br>1. Any clinically significant acute or chronic medical condition, other than HIV infection, that is considered progressive or in the opinion of the investigator makes the participant unsuitable for participation in the study.<br>2. If female, pregnant, lactating or planning a pregnancy during the period of screening through completion of the study.<br>3. In the past 6 months a history of alcohol or substance use, including marijuana, judged by the Investigator to potentially interfere with participant study compliance.<br>4. Bleeding disorder that was diagnosed by a physician (e.g., factor deficiency, coagulopathy or platelet disorder that requires special precautions). Note: A participant who states that he or she has easy bruising or bleeding, but does not have a formal diagnosis and has intramuscular injections and blood draws without any adverse experience, is eligible.<br>5. History of a splenectomy.<br>6. Receipt of live attenuated vaccine within the previous 30 days or planned receipt within 30 days after administration of investigational product; or receipt of other vaccine within the previous 14 days or planned receipt within 14 days after infusion with investigational product (exception is live attenuated influenza vaccine within 14 days).<br>7. Receipt of blood transfusion or blood-derived products within the previous 3 months.<br>8. Participation in another clinical trial of an investigational product currently, within the previous 3 months or expected participation during this study.<br>9. Prior receipt of an investigational HIV vaccine candidate, monoclonal antibody or polyclonal immunoglobulin (note: receipt of placebo in a previous HIV vaccine or monoclonal antibody trial will not exclude a participant from participation if documentation is available and the Medical Monitor gives approval).<br>10. History of severe local or systemic reactogenicity to injections or IV infusion (e.g., anaphylaxis, respiratory difficulties, angioedema);<br>11. Psychiatric condition that compromises safety of the participant and precludes compliance with the protocol. Specifically excluded are persons with psychoses within the past 3 years, ongoing risk for suicide, or history of suicide attempt or gesture within the past 3 years.<br>12. If, in the opinion of the Principal Investigator, it is not in the best interest of the participant to participate in the trial.

13. Seizure disorder: a participant who has had a seizure in the last 3 years is excluded. (Not excluded: a participant with a history of seizures who has neither required medications nor had a seizure for 3 years.)
14. Body mass index ≥ 35 or ≤ 18.0.
15. Infectious disease: chronic hepatitis B infection (HbsAg), current hepatitis C infection (HCV Ab positive and HCV RNA positive) or interferon-alfa treatment for chronic hepatitis C infection in the past year, or active syphilis.
16. A history of malignancy within the past 5 years (prior to screening) or ongoing malignancy;
17. Active, serious infections (other than HIV-1 infection) requiring parenteral antibiotic, antiviral or antifungal therapy within 30 days prior to enrollment.

Specific exclusion criteria for HIV-uninfected participants (Groups 1 and 2):

18. Confirmed HIV-1 or HIV-2 infection.
19. Any clinically relevant abnormality on history or examination including history of immunodeficiency or autoimmune disease; use of systemic corticosteroids, immunosuppressive, anticancer, or other medications considered significant by the investigator within the previous 6 months.

    The following exceptions are permitted and will not exclude study participation: use of corticosteroid nasal spray for rhinitis, topical corticosteroids for an acute uncomplicated dermatitis; or a short course (duration of 10 days or less, or a single injection) of corticosteroid for a non-chronic condition (based on investigator clinical judgment) at least 6 weeks prior to enrollment in this study.
20. Any of the following abnormal laboratory parameters listed below:

Hematology

- Hemoglobin < 10.5 g/dL in females; hemoglobin <11.0 g/dL in males
- Absolute Neutrophil Count (ANC): ≤ 1000/mm3
- Absolute Lymphocyte Count (ALC): < 650/mm3
- Platelets: < 125,000 mm3 or ≥ 550,000/mm3

Coagulation

- aPTT: >1.25 x ULN
- INR: ≥1.1 x ULN

Chemistry

- Sodium ≤ 135 mEq/L or ≥ 146 mEq/L
- Potassium ≤ 3.4 mEq/L or ≥ 5.6 mEq/L
- Creatinine ≥ 1.1 x ULN
- AST ≥ 1.25 x ULN
- ALT ≥ 1.25 x ULN
- Total bilirubin ≥ 1.25 x ULN
- Alkaline phosphatase ≥ 1.25 x ULN
- Albumin ≤ 3.0 g/dL or ≤ 30 g/L

- Creatine kinase ≥ 3.0 x ULN
- C-reactive protein > 10 mg/L
- C3 complement < 82 mg/dL
- C4 complement < 14 mg/dL

Urinalysis

Any of the following abnormal findings if consistent with clinically significant disease:

- o Protein = greater than trace on dipstick confirmed by microscopic urinalysis outside institutional range.
- o Blood = greater than trace on dipstick confirmed by >3 RBCs/hpf on microscopic urinalysis (not due to menses).

Specific exclusion criteria for HIV-infected participants who are not on ART (Group 3):

21. Any clinically relevant abnormality on history or examination including history of immunodeficiency or autoimmune disease, other than HIV; use of systemic corticosteroids, immunosuppressive, anticancer, or other medications considered significant by the investigator within the previous 6 months.

The following exceptions are permitted and will not exclude study participation: use of corticosteroid nasal spray for rhinitis, topical corticosteroids for an acute uncomplicated dermatitis; or a short course (duration of 10 days or less, or a single injection) of corticosteroid for a non-chronic condition (based on investigator clinical judgment) at least 6 weeks prior to enrollment in this study.

22. Any of the following abnormal laboratory parameters listed below:

Hematology

- Hemoglobin < 10.0 g/dL
- Absolute Neutrophil Count (ANC): < 1000 cells/mm3
- Platelets: < 100,000 cells/mm3

Coagulation

- aPTT: > 1.25 x ULN
- INR: ≥ 1.1 x ULN

Chemistry

- Estimated Glomerular filtration rate (GFR) < 80 mL/min according to the Cockcroft Gault formula for creatinine clearance
  - o Male: (140 – age in years) x (wt in kg) = CLcr (mL/min) / 72 x (serum creatinine in mg/dL)
  - o Female: (140 – age in years) x (wt in kg) x 0.85 = CLcr (mL/min) / 72 x (serum creatinine in mg/dL)
- AST ≥ 2.5 x ULN
- ALT ≥ 2.5 x ULN
- Total bilirubin ≥ 1.6 x ULN
- Alkaline phosphatase ≥ 5 x ULN

Urinalysis

Any of the following abnormal findings if consistent with

	clinically significant disease:<br>o Protein = greater than 1+ on dipstick confirmed by microscopic urinalysis outside institutional range.<br>o Blood = greater than 1+ on dipstick confirmed by > 10 RBCs/hpf on microscopic urinalysis (not due to menses).<br>o Leukocytes = greater than 1+ on dipstick confirmed by > 10 WBC/hpf on microscopic urinalysis.

TABLE OF CONTENTS

PROTOCOL SYNOPSIS **2**

TABLE OF CONTENTS **14**

ABBREVIATIONS **17**

CONTACT INFORMATION **18**

1.0 SIGNATURE PAGE **19**

2.0 INTRODUCTION AND BACKGROUND INFORMATION **20**

2.1 Study Rationale 20
2.2 Clinical experience with PGDM1400 and PGT121 21

3.0 STUDY OBJECTIVES **22**

3.1 Primary Objectives 22
3.2 Secondary Objectives 22
3.3 Exploratory Objectives 22

4.0 STUDY ENDPOINTS **22**

4.1 Primary Endpoints 22
4.2 Secondary Endpoints 23
4.3 Exploratory Endpoints 23

5.0 STUDY DESIGN **24**

5.1 Definition of Dose Limiting Toxicity- Groups 1 and 2 24
5.2 Definition of Maximum Tolerated Dose- Groups 1 and 2 24
5.3 Dose Escalation and Determination of Maximum Tolerated Dose – Groups 1 and 2 24
5.4 Determination of Antiviral Effect – Group 3 26
5.5 Duration of the Study 26
5.6 Study Population 26
5.7 Inclusion Criteria 27
5.8 Exclusion Criteria 28
5.9 Recruitment of Participants 31

6.0 STUDY VISITS **32**

6.1 Screening Period 32
6.2 IV infusion of Investigational Product Visit 32
6.3 Post-IV infusion of Investigational Product Visits 33
6.4 Additional Follow-up Visits 34
6.5 Unscheduled Visits 34
6.6 Final Study Visit or Early Termination Visit 34

7.0 STUDY PROCEDURES **35**

7.1 Informed Consent Process 35
7.2 Medical History and Physical Examination 35
7.3 HIV Testing and HIV-test Counselling (Groups 1 and 2) 36
7.4 HIV Risk Reduction Counselling 36
7.5 Family Planning Counselling 36
7.6 ART Counselling (Group 3) 36
7.7 Specimens 36

7.8 Reimbursement....37
7.9 Randomization and Blinding....37
7.10 Un-blinding Procedure for Individual Participants....37
7.11 Assessment of PGDM1400 mAb and/or PGT121 mAb related HIV sero-positivity....38

8.0 INVESTIGATIONAL PRODUCT....38

8.1 Description....38
8.2 Shipment and Storage....39
8.3 Preparation of investigational product....39
8.4 Administration of investigational product....39
8.5 Accountability and Disposal of investigational product....39

9.0 ASSESSMENTS....40

9.1 Safety Assessments....40
9.1.1 Local reactogenicity....40
9.1.2 Systemic reactogenicity....40
9.1.3 Vital Signs....40
9.1.4 Other Adverse Events....41
9.1.5 Concomitant Medications....41
9.1.6 Routine laboratory parameters....41
9.1.7 Specific screening tests:....42
9.2 Virologic Assessments....42
9.3 Exploratory Immunogenicity Assessments....42
9.3.1 Antibody Responses....42
9.3.2 Cellular Responses....43
9.3.3 PBMC, Serum and Plasma Storage....43
9.4 Other Assessments....43
9.4.1 HIV Antibody Testing (Groups 1 and 2)....43
9.4.2 Pharmacokinetics....43
9.4.3 HLA Typing....44
9.4.5 Pregnancy Test....44
9.4.6 HIV Risk Assessment (Group 1 and 2)....44
9.4.7 Social Impact Assessment....44

10.0 ADVERSE EVENTS....44

10.1 Definition....44
10.2 Assessment of Severity of Adverse Events....44
10.3 Relationship to Investigational Product....45
10.4 Serious Adverse Events....46
10.5 Reporting Potential Immune-Mediated Diseases....47
10.6 Clinical Management of Adverse Events....48
10.7 Pregnancy....48
10.8 Intercurrent HIV Infection (Group 1 and 2)....49

11.0 MANAGEMENT OF HIV ISSUES DURING AND FOLLOWING STUDY....49

11.1 HIV Testing – Groups 1 and 2....49
11.2 Social Discrimination as a Result of investigational product-related antibodies....49
11.3 HIV infection – Group 1 and 2....49
11.3.1 Counselling....50
11.3.2 Referral for Support/Care....50

12.0 WITHDRAWAL FROM STUDY....50

12.1 Deferral of IV infusion of investigational product....50

12.2 Withdrawal from the Study (Early Termination) 51
13.0 DATA HANDLING 51
13.1 Data Collection and Record Keeping at the Study Site 51
13.2 Data Entry at the Study Site 51
13.3 Data Analysis 52
14.0 STATISTICAL CONSIDERATIONS 52
14.1 Safety and Tolerability Analysis 52
14.2 Pharmacokinetic Analysis 53
14.3. Virologic Analysis for group 3 54
14.5 Secondary and Exploratory Immunologic and Virologic Analyses 55
15.0 QUALITY CONTROL AND QUALITY ASSURANCE 56
16.0 DATA AND BIOLOGICAL MATERIAL 56
17.0 ADMINISTRATIVE STRUCTURE 56
17.1 Protocol Safety Review Team 56
17.2 Safety Monitoring Committee (SMC) 57
17.2.1 Content of Interim Safety Review 57
17.2.2 SMC Review of Group 1 and 2 data prior to starting Group 3 57
17.3 Criteria for Pausing the Study 58
17.4 Study Supervision 59
17.5 Study Monitoring 59
17.6 Investigator's Records 59
18.0 INDEMNITY 59
19.0 PUBLICATION 60
20.0 ETHICAL CONSIDERATIONS 60
APPENDIX A: SCHEDULE OF PROCEDURES – GROUP 1 (A, B, C) 61
APPENDIX B: SCHEDULE OF PROCEDURES – AND GROUP 2 (A, B, C) 63
APPENDIX C: SCHEDULE OF PROCEDURES – GROUP 3 (A, B) 65
APPENDIX D: LOW RISK CRITERIA 68
APPENDIX E REFERENCES 70
APPENDIX F CTCAE TABLE 73
APPENDIX G: DAIDS ADVERSE EVENT SEVERITY ASSESSMENT TABLE 84

ABBREVIATIONS

Abbreviation	Term
**AE**	Adverse Event
**AIDS**	Acquired Immunodeficiency Syndrome
**ALT**	Alanine-Aminotransferase
**ART**	Antiretroviral Therapy
**AST**	Aspartate-Aminotransferase
**CFC**	Cytokine Flow Cytometry
**CMI**	Cell Mediated Immunity
**CRF**	Case Report Form
**CTL**	Cytotoxic T Lymphocyte
**DCC**	Data Coordinating Center
**DLT**	Dose Limiting Toxicity
**DNA**	Deoxyribonucleic Acid
**ELISA**	Enzyme Linked Immunosorbent Assay
**GCP**	Good Clinical Practice
**GFR**	Glomerular Filtration Rate
**HIV**	Human Immunodeficiency Virus
**HLA**	Human Leukocyte Antigen
**HSV**	Herpes Simplex Virus
**IAVI**	International AIDS Vaccine Initiative
**ICH**	International Council for Harmonization of Technical Requirements for Pharmaceuticals for Human Use
**IP**	Investigational Product
**IND**	Investigational New Drug Application
**IV**	Intravenous
**Kg**	Kilogram
**mAb**	Monoclonal Antibody
**mg**	Milligram
**MTD**	Maximum Tolerated Dose
**NHP**	Non Human Primate
**PCR**	Polymerase Chain Reaction
**PBMC**	Peripheral Blood Mononuclear Cells
**PK**	Pharmacokinetic
**RPR**	Rapid Plasma Reagin
**SAE**	Serious Adverse Event
**SIV**	Simian Immunodeficiency Virus
**SOP**	Standard Operating Procedure
**SOM**	Study Operations Manual
**SMC**	Safety Monitoring Committee
**STD**	Sexually Transmitted Disease
**TPHA**	Treponema Pallidum Hemagglutination

CONTACT INFORMATION

Detailed contact information provided in the Study Operation Manual (SOM)

Sponsor Contact:	
Frances Priddy MD MPH<br>Chief Medical Officer and Executive Director<br>International AIDS Vaccine Initiative<br>125 Broad Street, 9th Floor<br>New York, New York 10004	Phone: +1-212-328-7461<br>Mobile: +1-646-287-8943<br>Fax: +1-608-203-5501<br>E-mail: fpriddy@iavi.org
**Clinical Research Center Contacts:**	
Boris D. Juelg MD PhD<br>Center for Virology and Vaccine Research<br>Clinical Trials Unit<br>Beth Israel Deaconess Medical Center<br>E / CLS – 10th Floor, Room 1046<br>330 Brookline Avenue<br>Boston, Massachusetts 02215	Phone: +1-857-268-7088<br>Mobile: +1-617-401-6725<br>Fax: +1-617-735-4566<br>E-mail: bjulg@bidmc.harvard.edu

.

1.0 SIGNATURE PAGE

The signatures below constitute the approval of this protocol and the appendices and provide the necessary assurances that this study will be conducted in compliance with the protocol, Good Clinical Practices (GCP) and the applicable regulatory requirement(s).

Sponsor:

Signed: See electronic signature manifest Date:

Frances Priddy MD MPH
Chief Medical Officer and Executive Director, Medical Affairs, IAVI

Principal Investigator:

Signed: Date:

Name (please print):

Name of institution (please print):

2.0 INTRODUCTION AND BACKGROUND INFORMATION

In 2015, 36.7 million people globally were living with HIV, 2.1 million people became newly infected with HIV, and 1.1 million people died from AIDS-related illnesses (UNAIDS 2016). More than 78 million people have become infected with HIV and 39 million people have died since the beginning of the AIDS epidemic (UNAIDS 2016). One reason that such high rates of AIDS-related deaths continue to occur globally – despite the advent of drugs that are highly effective at suppressing HIV replication – is that by June 2016, 18.2 million people (two in four people living with HIV) were accessing antiretroviral therapy (ART) (UNAIDS 2016). Another reason for continued AIDS-related mortality is that ART does not cure HIV infection and must be maintained for a lifetime (Barouch and Deeks 2014). Even in the United States (US), only 30% of the 1.2 million people living with HIV have suppressed HIV to undetectable levels, likely because 1) not everyone is aware of their HIV-positive status, 2) not everyone is accessing ART, and 3) of the challenge of maintaining adequate continuity of ART over many years (CDC 2014).

2.1 Study Rationale

This is a Phase 1 study to evaluate the safety, tolerability, pharmacokinetics and anti-viral efficacy of the PGDM1400 and PGT121 mAbs for HIV prevention and therapy. PGDM1400 mAb and PGT121 mAb are recombinant human IgG1 monoclonal antibodies that target a V1V2 (PGDM1400) and a V3 glycan-dependent (PGT121) epitope region of the HIV envelope protein (Jardine, Julien et al. 2013, Sok, Doores et al. 2014). PGDM1400 and PGT121 mAbs were chosen for this study because of their potency, their ability to neutralize a wide array of cross-clade HIV viruses in a complementary pattern, and their proven antiviral activity in animals studies e.g., their capacity to robustly prevent and treat simian-human immunodeficiency virus (SHIV) in rhesus monkeys.

Some HIV-infected individuals develop HIV specific antibodies capable of neutralizing a broad range of HIV virus strains (Simek, Rida et al. 2009, Walker, Phogat et al. 2009). By selecting and cloning B-cells from such HIV-infected individuals, monoclonal antibodies have been identified which have potent and broadly neutralizing activity against a wide range of HIV virus strains (Walker LM 2010). New developments in high throughput single-cell BCR-amplification and novel soluble Env baits have led to the isolation of monoclonal antibodies with extraordinary potency and breadth (Walker, Huber et al. 2011).

These HIV-specific antibodies target the HIV envelope (Env) and can prevent SHIV infection in rhesus monkeys and have been shown to reduce HIV RNA levels in humans temporarily (Hessell, Poignard et al. 2009, Hessell, Rakasz et al. 2009, Moldt, Rakasz et al. 2012, Barouch, Whitney et al. 2013, Caskey, Klein et al. 2015, Caskey, Schoofs et al. 2017). These broadly neutralizing antibodies (bnAbs) may be effective for prevention of HIV infection when administered passively (Haynes and McElrath 2013, Burton and Mascola 2015). It is likely that bnAbs targeting different HIV epitopes will need to be used in combination to prevent development of resistance and to adequately cover all global HIV strains.

PGDM1400 can neutralize a wide array of HIV-1 viruses *in vitro* and can treat and prevent simian-human immunodeficiency virus (SHIV) in the NHP model (Sok, van Gils et al. 2014) and (Julg et al, unpublished data).
PGDM1400 is a novel monoclonal antibody that targets the V1V2 loop on the HIV Env spike, distinct from VRC01 and 3BNC117 which both target the CD4 binding site, and PGT121 and 10-1074 which target the V3 loop.

PGDM1400 mAb and PGT121 mAb were selected for development because of the following critical attributes:

1. PGDM1400 neutralizes 83% of global viral isolates at a median IC50 of 0.003 µg/mL (Sok et al 2014) and is therefore 10 to 100-fold more potent than the previous best-in-class CD4bs antibodies VRC01, VRC07, and 3BNC117 (Scheid, Mouquet et al. 2011, Walker, Huber et al. 2011, Sok, van Gils et al. 2014)
2. PGDM1400 protects against SHIV acquisition in monkeys at substantially lower dose levels compared to VRC01 and 3BNC117 (unpublished data)
3. PGT121 mAb has superior therapeutic efficacy in SHIV-infected monkeys compared to VRC01, 3BNC117, and 10-1074 (Barouch, Whitney et al. 2013) (and unpublished data).
4. PGT121 mAb may have a higher bar to escape in vivo as compared to other V3 glycan and CD4bs antibodies as a result of making multiple glycan contacts (Sok, van Gils et al. 2014).
5. PGDM1400 and PGT121 are complementary in the coverage of global viral isolates and when combined neutralize 98-99% of global HIV-1 viruses tested and at unparalleled potency with a median IC50 of 0.007 µg/ml (Sok, van Gils et al. 2014).

The potency and breadth of PGDM1400 mAb and PGT121 mAb, both alone and in combination with other bNAbs, raise the possibility that combinations may be effective for HIV prophylaxis at low doses and against global viruses. An antibody that is effective at low doses may eventually be given subcutaneously, which would reduce the cost. It is these features that make PGDM1400 mAb and PGT121 mAb particularly well-suited for preventing and/or treating HIV in the developing world, where it is critical that a public health intervention be low cost, easy to deliver, and effective in diverse settings.

2.2 Clinical experience with PGDM1400 and PGT121

There is no previous clinical experience with PGDM1400 mAb, and limited experience with PGT121 mAb. The safety, pharmacokinetics and antiviral effects of PGT121 mAb alone are being evaluated in the ongoing T001 study (IND 126807, NCT02960581). Several other HIV monoclonal antibodies are currently in clinical development as passive HIV immunoprophylaxis, or as potential therapeutics (10-1074, 3BNC117, VRC01, VRC01LS). Published data from phase 1 studies shows acceptable preliminary safety and tolerability profiles for these products, and similar anti-viral effects and pharmacokinetics (Caskey, Klein et al. 2015, Ledgerwood, Coates et al. 2015, Lynch, Boritz et al. 2015, Bar, Sneller et al. 2016, Scheid, Horwitz et al. 2016, Schoofs, Klein et al. 2016, Caskey, Schoofs et al. 2017). A comprehensive summary of phase 1 studies of HIV monoclonal antibodies can be found in the Investigator's Brochure.

3.0 STUDY OBJECTIVES

3.1 Primary Objectives

- To evaluate the safety and tolerability of IV infusion of PGDM1400 mAb alone, and a combination of PGDM1400 mAb and PGT121 mAb, in HIV-uninfected adults and HIV-infected adults
- To evaluate the pharmacokinetic (PK) profile of IV infusion of PGDM1400 mAb alone, and a combination of PGDM1400 mAb and PGT121 mAb, in HIV-uninfected adults and HIV-infected adults
- To evaluate the antiviral activity of IV infusion of PGDM1400 mAb alone, and a combination of PGDM1400 and PGT121 mAb, in HIV-infected adults not on ART

3.2 Secondary Objectives

- To determine if PGDM1400 mAb alone, and in combination with PGT121 mAb, induces anti-PGDM1400 and/or anti-PGT121 antibodies
- To determine the effect of PGDM1400 mAb alone, and in combination with PGT121 mAb, on CD4+ T cell counts in HIV-infected adults
- To determine the effect of PGDM1400 mAb alone, and in combination with PGT121 mAb, on viral escape in viremic HIV-infected adults not on ART

3.3 Exploratory Objectives

- To determine if PGDM1400 ± PGT121 mAb has any impact on the host immune responses (i.e., HIV-specific cellular and humoral immune response).
- To determine the effect of PGDM1400 ± PGT121 mAb on the size of the latent HIV reservoir in HIV-infected adults.
- To determine PGDM1400 mAb ± PGT121 mAb levels in mucosal secretions in HIV-uninfected and HIV-infected adults.
- To measure in vitro neutralization of HIV isolates with participant’s serum post PGDM1400 ± PGT121 mAb IV infusion.

4.0 STUDY ENDPOINTS

4.1 Primary Endpoints

Safety and Tolerability

1. Proportion of participants with moderate or greater reactogenicity (e.g., solicited adverse events) for 3 days following IV infusion of PGDM1400 mAb alone, and a combination of PGDM1400 mAb and PGT121 mAb.
2. Proportion of participants with adverse events (AEs), including safety laboratory (biochemical, hematological) parameters, during the first 56 days following IV infusion of PGDM1400 mAb alone and a combination of PGDM1400 mAb and PGT121 mAb, that are moderate or greater, and/or related to PGDM1400 mAb or PGT121 mAb.
3. Proportion of participants with serious adverse events (SAEs) throughout the study period following IV infusion of PGDM1400 mAb alone and a combination of PGDM1400 mAb and PGT121 mAb, that are related to PGDM1400 mAb or PGT121 mAb.

Pharmacokinetics
Pharmacokinetics following IV infusion of PGDM1400 mAb alone or a combination of PGDM1400 mAb and PGT121 mAb in HIV-uninfected and HIV-infected adults:
1. Elimination half-life (t1/2)
2. Clearance (CL/F)
3. Volume of distribution (Vz/F)
4. Area under the concentration decay curve (AUC)
5. Impact of viral load and/or ART on PGDM1400 mAb and PGT121 mAb disposition (elimination half-life (t1/2), clearance (CL/F), volume of distribution (Vz/F), total exposure)

Antiviral activity
Antiviral activity following IV infusion of PGDM1400 mAb alone or in combination with PGT121 mAb in viremic HIV-infected adults not on ART:
1. Change in plasma HIV-1 RNA levels from baseline (mean of pre-entry and entry values)

4.2 Secondary Endpoints

Anti-PGDM1400 and anti-PGT121 antibodies
1. Serum anti-PGDM1400 antibody titers
2. Serum anti-PGT121 antibody titers

CD4+ T cell count
We will calculate the following endpoint to determine if IV infusion of PGDM1400 mAb alone or in combination with PGT121 mAb has any impact on CD4+ T cell counts in HIV-infected adults:
1. Change in CD4+ T cell count and frequency compared to baseline as measured by single platform flow cytometry

HIV genotyping/phenotyping of circulating virus for evaluation of PGDM1400 mAb and/or PGT121 mAb -induced escape mutations
We will compare plasma virus genotype and phenotypic activity before and after IV infusion of PGDM1400 mAb alone or in combination with PGT121 mAb to determine if PGDM1400 mAb and/or PGT121 mAb induce viral escape mutations have developed in viremic HIV-infected adults not on ART.
1. Genotypic analysis: Development of sequence variations in epitopes known to result in reduced PGDM1400 mAb and/or PGT121 mAb neutralization susceptibility or known to cause resistance to antiretroviral drugs.
2. Phenotypic analysis: Changes in viral susceptibility to PGDM1400 mAb and/or PGT121 mAb neutralization.

4.3 Exploratory Endpoints

Additional assessments may include but are not limited to the following: HIV-specific IgG/IgA binding responses by ELISA, HIV-specific cellular immune responses by ELISPOT, HIV-specific antibody function by ADCC, ADCP, and ADCVI assays, PGDM1400 mAb and/or PGT121 mAb levels in mucosal secretions, changes in total HIV-1 DNA and 2-long terminal repeat (LTR) circular HIV-1 DNA in resting or total CD4 T cells and in vitro neutralization of HIV isolates with participant's serum post IV infusion of PGDM1400 mAb ± PGT121 mAb.

5.0 STUDY DESIGN

The study is a double-blind, randomized, placebo-controlled study for Groups 1 and 2, and open label for Group 3 who will not receive placebo. A single intravenous (IV) infusion of PGDM1400 mAb alone at 3, 10 or 30 mg/kg, or a combination of PGDM1400 mAb and PGT121 mAb at 3, 10 or 30 mg/kg each, or placebo will be administered to participants. See study Table 5.0.1 for details.

Table 5.0.1 Study Design Table

	Group	Participants	Sub-Group	Regimen	N	Dose (mg/kg)
Part 1 – MTD	1	HIV-uninfected participants	1A	PGDM1400/Placebo	3/1 (6/2 if DLT)	3 IV
			1B	PGDM1400/Placebo	3/1 (6/2 if DLT)	10 IV
			1C	PGDM1400/Placebo	3/1 (6/2 if DLT)	30 IV
				Total Group 1	9/3 = 12 (max 18/6 = 24 if DLT)	
	2	HIV-uninfected participants	2A	PGDM1400 + PGT121/Placebo	3/1 (6/2 if DLT)	3 + 3 IV
			2B	PGDM1400 + PGT121/Placebo	3/1 (6/2 if DLT)	10 + 10 IV
			2C	PGDM1400 + PGT121/Placebo	3/1 (6/2 if DLT)	30 + 30 IV
				Total Group 2	9/3 = 12 (max 18/6 = 24 if DLT)	
				Total Groups 1 and 2	18/6 = 24 (max 36/12 = 48 if DLT)	
Safety Monitoring Committee						
Part 2 – antiviral effect	3	HIV-infected off ART (VL 1x10^3 – 1x10^5 copies/ml)	3A	PGDM1400	6 (max 18)	MTD IV
			3B	PGDM1400 + PGT121	6 (max 18)	MTD IV
				Total Group 3	12 (max 36)	
				Total entire study	36 (max 84)	

DLT, dose limiting toxicity; MTD, maximum tolerated dose

5.1 Definition of Dose Limiting Toxicity- Groups 1 and 2

Dose limiting toxicity (DLT) will be defined as 1) any Grade 3 or greater adverse event considered possibly, probably or definitely related to investigational product or 2) any Grade 3 or greater reactogenicity or 3) any SAE considered possibly, probably or definitely related to investigational product.

5.2 Definition of Maximum Tolerated Dose- Groups 1 and 2

If 2 or more DLTs occur in a dose Subgroup (e.g., 1B) that are the same, similar, or in the same System Organ Class, infusion will be halted and the next lower dose level will be declared the maximum tolerated dose (MTD) within this Group. If no DLT occurs in the final dose Subgroup (1C and 2C), MTD will be the highest dose given (30mg/kg) after 14 days of follow-up.

5.3 Dose Escalation and Determination of Maximum Tolerated Dose – Groups 1 and 2

Part 1 of this study is a dose-escalation trial in HIV-uninfected adults (Groups 1 and 2) to establish the MTD of PGDM1400 mAb alone and a combination of PGDM1400 mAb and

PGT121 mAb. There will be sentinel participants in each dose Subgroup, and safety data will be reviewed to determine if dose escalation can proceed.

Sentinel participants in Groups 1 and 2

For each dose Subgroup in Groups 1 and 2, the 1st 2 participants will be sentinel participants for whom investigational product infusion will be separated by at least 24 hours contingent on review of safety information prior to infusion of the next sentinel participant. If no reactogenicity or adverse events that meet the DLT criteria occur within 24 hours after IV infusion of the 1st participant, the 2nd participant may be infused with investigational product. If no events meeting the DLT criteria occur within 24 hours after the 2nd participant is infused, then the remaining participants in that dose Subgroup will be infused. If events meeting the DLT criteria do occur for the first 2 participants in a dose Subgroup, the data will be reviewed by the Safety Monitoring Committee (SMC) to determine whether further infusions may proceed.

Staggered dose escalation design whereby PGDM1400 alone and PGT121 alone safety data are reviewed before combined infusion of PGDM1400 and PGT121 at the same dose

The combination Groups, starting with PGDM1400 3 mg/kg + PGT121 3 mg/kg can only start when 1) the PSRT has reviewed the safety data through day 14 post investigational product infusion from PGDM1400 alone at 3 mg/kg and has approved dose escalation to 10 mg/kg AND 2) the MTD of PGT121 as determined in the T001 study is 3 mg/kg or higher. Dosing levels of PGDM1400 mAb and PGT121 in the combination Subgroups can never exceed the MTDs for PGDM1400 and PGT121 as determined in the PGDM1400 alone Group 1 in this study, and the PGT121 alone Group in the T001 study.

PSRT review for dose escalation

The Protocol Safety Review Team (PSRT) will review safety data through day 14 post-investigational product infusion for all participants in each dose Subgroup for PGDM1400 mAb alone (Group 1) prior to allowing enrolment of participants into the next higher dose Subgroup in Group 1 or the same dose level PGDM1400 mAb and PGT121 mAb combination dose Subgroup (Group 2).

- If no DLT occurs in the initial 4 participants of a dose Subgroup (e.g., 1A), the study can proceed with enrolment of the next dose Subgroup for PGDM1400 mAb alone (e.g., 1B), and the same dose level PGDM1400 mAb and PGT121 mAb combination dose Subgroup (e.g., 2A).
- If 1 DLT occurs in the initial 4 participants of a dose Subgroup (e.g., 1A), 4 additional participants will be enrolled in the same dose Subgroup.
    - If no additional DLTs occur within 14 days of infusion in the 8 total participants, the study can proceed with enrolment of the next dose Subgroup for PGDM1400 mAb alone (e.g., 1B), and the same dose level PGDM1400 mAb and PGT121 mAb combination dose Subgroup (e.g., 2A).
    - If 2 or more DLTs accumulate in a dose Subgroup (e.g., 1B) that are the same, similar, or in the same System Organ Class, infusion will be halted and the next lower dose level will be declared the maximum tolerated dose (MTD) within this Group.
    - If no DLT occurs in the final dose Subgroup (e.g.,1C), the MTD for PGDM1400 alone will be the highest dose given (30mg/kg) after 14 days of follow-up.

- Dosing levels of PGDM1400 mAb and PGT121 in the combination Subgroups can never exceed the MTDs for PGDM1400 and PGT121 as determined in the PGDM1400 alone Group 1 in this study, and the PGT121 alone Group in the T001 study.

SMC review to determine MTD of PGDM1400 alone
Following IV infusion of of investigational product in the last participant in Group 1 , an independent Safety Monitoring Committee (SMC) will review safety data through day 14 post-investigational product infusion for all participants to confirm MTD of PGDM1400 alone, and determine whether, and at what dose, Group 3A can initiate enrolment.

SMC review to determine MTD of the combination of PGDM1400 and PGT121
Following IV infusion of investigational product in the last participant in Group 2, the SMC will review safety data through day 14 post-investigational product infusion for all participants to confirm MTD of the combination of PGDM1400 and PGT121, and determine whether Group 3B can initiate enrollment.

5.4 Determination of Antiviral Effect – Group 3

Part 2 of this study will establish the antiviral effect of PGDM1400 mAb alone or the combination of PGDM1400 mAb plus PGT121 mAb in HIV infected adults not on ART (Group 3).

MTD determines dose
Group 3 will start with the MTD of PGDM1400 as determined by the SMC; e.g., if the MTD for PGDM1400 is 30mg/kg then Subgroup 3A will receive 30mg/kg and Subgroup 3B will receive PGDM1400 mAb + PGT121 mAb at a dose that combines the MTD for PGDM1400 mAb and the MTD for PGT121 mAb (as determined in the ongoing T001 study IND 126807, NCT02960581).

5.5 Duration of the Study

Up to 32 weeks per participant, screening up to 42 (HIV-infected) or up to 56 (HIV-uninfected) days before single IV infusion of investigational product on day 0, and 24 weeks of follow up.

It will take approximately 11 months to enroll the entire study. The exact duration depends on the recruitment rate and how many participants will be required per dose Subgroup as specified in sections 5.2 and 5.3.

5.6 Study Population

The study population consists of HIV-uninfected male or female adults (Group 1 and 2) and HIV-infected males and female adults (Group 3) who meet the detailed inclusion and exclusion criteria listed below, and who in the opinion of the investigator or designee, understand the study and provide written informed consent.

Approximately 36-84 participants (72 active product recipients, 12 placebo recipients) who meet all eligibility criteria will be included in the study. An over-enrollment of up to 5% (up to 5 participants total) will be permitted in the study to facilitate rapid enrollment.

5.7 Inclusion Criteria

Inclusion criteria for all participants:

1. Willing to comply with the requirements of the protocol and available for follow-up for the planned duration of the study.

2. In the opinion of the Principal Investigator or designee and based on Assessment of Informed Consent Understanding results, has understood the information provided and potential impact and/or risks linked to IV infusion and participation in the trial; written informed consent will be obtained from the participant before any study-related procedures are performed.

3. All heterosexually active female participants must commit to use an effective method of contraception for 3 months following investigational product administration, including:

   a. Condoms (male or female) with or without spermicide

   b. Diaphragm or cervical cap with spermicide

   c. Intrauterine device, or contraceptive implant

   d. Hormonal contraception

   e. Successful vasectomy in the male partner (considered successful if a woman reports that a male partner has [1] documentation of azoospermia by microscopy (< 1 year ago), or [2] a vasectomy more than 2 years ago with no resultant pregnancy despite sexual activity post-vasectomy)

   f. Not be of reproductive potential, such as having undergone hysterectomy, bilateral oophorectomy, or tubal ligation, postmenopausal (> 45 years of age with amenorrhea for at least 2 years, or any age with amenorrhea for at least 6 months and a serum follicle stimulating hormone [FSH] level > 40 IU/L); surgically sterile: no additional contraception required.

   g. Women, who are not heterosexually active at screening, must agree to utilize an effective method of contraception if they become hetero-sexually active, as outlined above.

4. All sexually active males, regardless of reproductive potential, must be willing to consistently use an effective method of contraception (such as consistent male condoms with male and/or female partners) from the day of investigational product administration until at least 3 months following investigational product administration to avoid exposure of partners to investigational product in ejaculate, and to prevent conception with female partners.

5. All female participants must be willing to undergo urine pregnancy tests at time points indicated in the Schedule of Procedures and must test negative prior to investigational product administration.

6. A female participant must agree not to donate eggs (ova, oocytes) for the purpose of assisted reproduction until 3 months after investigational product administration. A man must agree not to donate sperm until 3 months after investigational product administration.

7. Willing to forgo donations of blood and/or any other tissues, including bone marrow, during the study and, for those HIV-uninfected participants who test HIV-positive due to investigational product administration, until the anti-HIV antibody titers become undetectable.

Specific inclusion criteria for HIV-uninfected participants (Groups 1 and 2):

8. At least 18 years of age on the day of screening and has not reached his or her 51st birthday on the day of signing the Informed Consent Document.

9. Willing to undergo HIV testing, risk reduction counselling and receive HIV test results.

10. Low risk for HIV infection and willing to maintain low-risk behavior for the duration of the trial.

11. Healthy male or female, as assessed by a medical history, physical exam, and laboratory tests.

Specific inclusion criteria for HIV-infected participants (Groups 3):

12. At least 18 years of age on the day of screening and has not reached his or her 66th birthday on the day of signing the Informed Consent Document.

13. Confirmed HIV-1 infection (HIV Ab+ or HIV RNA+) by documentation in the medical records or in-clinic HIV testing;

14. CD4 ≥ 300 cells/μl.

15. Not receiving cART, and (after appropriate counselling) willing to defer cART treatment for at least 56 days after administration of investigational product.

16. HIV-1 viral load between 1000–100,000 copies/ml, confirmed at screening.

5.8 Exclusion Criteria

Exclusion criteria for all participants:

1. Any clinically significant acute or chronic medical condition, other than HIV infection, that is considered progressive or in the opinion of the investigator makes the participant unsuitable for participation in the study.

2. If female, pregnant, lactating or planning a pregnancy during the period of screening through completion of the study.

3. In the past 6 months a history of alcohol or substance use, including marijuana, judged by the Investigator to potentially interfere with participant study compliance.

4. Bleeding disorder that was diagnosed by a physician (e.g., factor deficiency, coagulopathy or platelet disorder that requires special precautions). Note: A participant who states that he or she has easy bruising or bleeding, but does not have a formal diagnosis and has intramuscular injections and blood draws without any adverse experience, is eligible.

5. History of a splenectomy.

6. Receipt of live attenuated vaccine within the previous 30 days or planned receipt within 30 days after administration of investigational product; or receipt of other vaccine within the previous 14 days or planned receipt within 14 days after infusion with investigational product (exception is live attenuated influenza vaccine within 14 days).

7. Receipt of blood transfusion or blood-derived products within the previous 3 months.

8. Participation in another clinical trial of an investigational product currently, within the previous 3 months or expected participation during this study.

9. Prior receipt of an investigational HIV vaccine candidate, monoclonal antibody or polyclonal immunoglobulin (note: receipt of placebo in a previous HIV vaccine or monoclonal antibody trial will not exclude a participant from participation if documentation is available and the Medical Monitor gives approval).

10. History of severe local or systemic reactogenicity to injections or IV infusion (e.g., anaphylaxis, respiratory difficulties, angioedema);

11. Psychiatric condition that compromises safety of the participant and precludes compliance with the protocol. Specifically excluded are persons with psychoses within the past 3 years, ongoing risk for suicide, or history of suicide attempt or gesture within the past 3 years.

12. If, in the opinion of the Principal Investigator, it is not in the best interest of the participant to participate in the trial.

13. Seizure disorder: a participant who has had a seizure in the last 3 years is excluded. (Not excluded: a participant with a history of seizures who has neither required medications nor had a seizure for 3 years.)

14. Body mass index ≥ 35 or ≤ 18.0.

15. Infectious disease: chronic hepatitis B infection (HbsAg), current hepatitis C infection (HCV Ab positive and HCV RNA positive) or interferon-alfa treatment for chronic hepatitis C infection in the past year, or active syphilis.

16. A history of malignancy within the past 5 years (prior to screening) or ongoing malignancy;

17. Active, serious infections (other than HIV-1 infection) requiring parenteral antibiotic, antiviral or antifungal therapy within 30 days prior to enrollment.

Specific exclusion criteria for HIV-uninfected participants (Group 1 and 2):

18. Confirmed HIV-1 or HIV-2 infection.

19. Any clinically relevant abnormality on history or examination including history of immunodeficiency or autoimmune disease; use of systemic corticosteroids, immunosuppressive, anticancer, or other medications considered significant by the investigator within the previous 6 months.

    The following exceptions are permitted and will not exclude study participation: use of corticosteroid nasal spray for rhinitis, topical corticosteroids for an acute uncomplicated dermatitis; or a short course (duration of 10 days or less, or a single injection) of corticosteroid for a non-chronic condition (based on investigator clinical judgment) at least 6 weeks prior to enrollment in this study.

20. Any of the following abnormal laboratory parameters listed below:

    <u>Hematology</u>
    Hemoglobin < 10.5 g/dL in females; hemoglobin <11.0 g/dL in males
    Absolute Neutrophil Count (ANC): ≤ 1000/mm3
    Absolute Lymphocyte Count (ALC): < 650/mm3
    Platelets: < 125,000 mm3 or ≥ 550,000/mm3
    <u>Coagulation</u>
    aPTT: >1.25 x ULN
    INR: ≥1.1 x ULN
    <u>Chemistry</u>
    - Sodium ≤ 135 mEq/L or ≥ 146 mEq/L
    - Potassium ≤ 3.4 mEq/L or ≥ 5.6 mEq/L
    - Creatinine ≥ 1.1 x ULN
    - AST ≥ 1.25 x ULN
    - ALT ≥ 1.25 x ULN
    - Total bilirubin ≥ 1.25 x ULN
    - Alkaline phosphatase ≥ 1.25 x ULN
    - Albumin ≤ 3.0 g/dL or ≤ 30 g/L
    - Creatine kinase ≥ 3.0 x ULN
    - C-reactive protein > 10 mg/L
    - C3 complement < 82 mg/dL
    - C4 complement < 14 mg/dL

    <u>Urinalysis</u>
    Any of the following abnormal findings if consistent with clinically significant disease:
    - Protein = greater than trace on dipstick confirmed by microscopic urinalysis outside institutional range.
    - Blood = greater than trace on dipstick confirmed by >3 RBCs/hpf on microscopic urinalysis (not due to menses).

Specific exclusion criteria for HIV-infected participants who are not on ART (Group 3):

21. Any clinically relevant abnormality on history or examination including history of immunodeficiency or autoimmune disease, other than HIV; use of systemic

corticosteroids, immunosuppressive, anticancer, or other medications considered significant by the investigator within the previous 6 months.

The following exceptions are permitted and will not exclude study participation: use of corticosteroid nasal spray for rhinitis, topical corticosteroids for an acute uncomplicated dermatitis; or a short course (duration of 10 days or less, or a single injection) of corticosteroid for a non-chronic condition (based on investigator clinical judgment) at least 6 weeks prior to enrollment in this study.

22. Any of the following abnormal laboratory parameters listed below:

<u>Hematology</u>
- Hemoglobin < 10.0 g/dL
- Absolute Neutrophil Count (ANC): < 1000 cells/mm3
- Platelets: < 100,000 cells/mm3

<u>Coagulation</u>
- aPTT: > 1.25 x ULN
- INR: ≥ 1.1 x ULN

<u>Chemistry</u>
- Estimated Glomerular filtration rate (GFR) < 80 mL/min according to the Cockcroft Gault formula for creatinine clearance
    - Male: (140 – age in years) x (wt in kg) = CLcr (mL/min) / 72 x (serum creatinine in mg/dL)
    - Female: (140 – age in years) x (wt in kg) x 0.85 = CLcr (mL/min) / 72 x (serum creatinine in mg/dL)
- AST ≥ 2.5 x ULN
- ALT ≥ 2.5 x ULN
- Total bilirubin ≥ 1.6 x ULN
- Alkaline phosphatase ≥ 5 x ULN

<u>Urinalysis</u>
Any of the following abnormal findings if consistent with clinically significant disease:
- Protein = greater than 1+ on dipstick confirmed by microscopic urinalysis outside institutional range.
- Blood = greater than 1+ on dipstick confirmed by > 10 RBCs/hpf on microscopic urinalysis (not due to menses).
- Leukocytes = greater than 1+ on dipstick confirmed by > 10 WBC/hpf on microscopic urinalysis.

5.9 Recruitment of Participants

Adult male and female participants may be recruited through in-clinic referrals, information presented to community organizations, hospitals, colleges, other institutions and/or advertisements to the general public or from existing cohorts. The information distributed will contain contact details of the trial site.

6.0 STUDY VISITS

6.1 Screening Period

During Screening, study staff will perform the following procedures:

- Provide and/or review the Informed Consent Document and answer any questions about the study prior to obtaining written informed consent.
- Complete Assessment of Informed Consent Understanding (AOU). Please refer to the Study Operations Manual (SOM)

If the participant agrees to participate, passes the AOU and provides written informed consent, study staff will:

- Conduct HIV test counselling, HIV testing, and HIV risk reduction counselling, as applicable
- Conduct family planning counselling, refer for pregnancy prevention counselling if necessary
- Administer HIV risk assessment (Group 1 and 2)
- Conduct ART counselling (Group 3)
- Perform a comprehensive medical history
- Collect concomitant medication information
- Perform a general physical examination (Refer to Section 7.2)
- Collect specimens for all tests as indicated in the Schedule of Procedures in Appendices A, B and C (for details see Analytical Plan (AP)).

When available, the screening laboratory tests will be reviewed by the trial physician. Screening laboratory test(s) may be repeated once at the discretion of the principal investigator or designee to investigate any isolated abnormalities.

If the screening visit occurs outside the allowable screening window, all screening procedures must be repeated except the comprehensive medical history may be replaced by an interim medical history and the Participant Information Sheet of the Informed Consent Document should be reviewed.

If a participant has signed the Consent Form but does not meet the eligibility criteria, the records must be kept at the site.

6.2 IV infusion of Investigational Product Visit

Prior to the infusion of investigational product, study staff will:

- Answer any questions the participant may have about the study
- Review the Informed Consent Document with the participant
- Review screening safety laboratory data
- Conduct HIV test counselling, and HIV risk reduction counselling , as applicable
- Conduct ART counselling (Group 3)
- Conduct family planning counselling as per site specific procedures and ensure compliance with respective pregnancy prevention method, and discuss male condom use with all male participants

- Review interim medical history
- Collect concomitant medication information
- Weigh participant and record vital signs
- Perform a symptom-directed physical examination (Refer to Section 7.2)
- Assess at baseline local and systemic signs and symptoms (this includes an examination of IV infusion site)
- Collect specimens for all tests as indicated in the Schedule of Procedures see Appendices A, B and C (for details see AP).
- Obtain pregnancy test results prior to infusion of investigational product.
- Assign an allocation number to the participant according to the instructions specified in the Study Operations Manual.

If a participant has an abnormal laboratory value that is known, at the time of infusion, follow the specified guidelines (Section 12.0).

At the time of infusion of investigational product and after IV infusion of investigational product, study staff will:

- Administer the investigational product as specified in Section 8.4, Administration of Investigational Product and according to the instructions specified in the SOM.
- Observe participant closely during the infusion of investigational product and for at least 30 minutes after IV infusion of investigational product has ended for any acute reactogenicity. At the end of the observation period study staff will:
    - Record vital signs (pulse, respiratory rate, blood pressure and temperature)
    - Assess any local and systemic reactogenicity
    - Assess any other adverse events
- Every hour after IV infusion of investigational product, starting hour 1 through 6, the study staff will:
    - Record vital signs (pulse, respiratory rate, blood pressure and temperature)
    - Assess any local and systemic reactogenicity
    - Assess any other adverse events
    - Collect PK samples according to the Schedule of Procedures

6.3 Post-IV infusion of Investigational Product Visits

The participant will be asked to return to the clinic for post-investigational product infusion visits as indicated in the schedule of procedures (see appendices A, B and C) for an assessment by clinic staff. The participant will be asked to maintain a Memory Aid to track any local and systemic reactogenicity the participant experiences, including temperature, from the day of investigational product infusion for the next 3 days (for a total of 4 days including day of investigational product infusion). Study staff will review the Memory Aid with the participant and determine the severity of the reactions through discussion with the participant.

The following procedures will be conducted at these visits:

- Review interim medical history
- Collect concomitant medication information
- Perform a symptom-directed physical examination if any signs or symptoms are present

- Assess vital signs (pulse, respiratory rate, blood pressure and temperature)
- Assess any adverse events and local and systemic reactogenicity (Days 1, 2, 3) including reviewing the Memory Aid.
- Collect specimens for all tests as indicated in the Schedule of Procedures (Appendices A, B and C and AP).

6.4 Additional Follow-up Visits

Assessments and procedures will be performed according to the Schedule of Procedures (Appendices A, B and C).

6.5 Unscheduled Visits

Unscheduled Visits/Contacts are visits/contacts that are not described in the Schedule of Procedures (Appendices A, B and C). Unscheduled visits may occur any time during the study:

- For administrative reasons, e.g., the participant may have questions for study staff or may need to re-schedule a follow-up visit.
- To obtain laboratory test results from a previous visit.
- For other reasons as requested by the participant or site investigator.

All unscheduled visits will be documented in the participants' study records on applicable source documents and entered into the Case Report Form (CRF).

6.6 Final Study Visit or Early Termination Visit

Assessments and procedures will be performed according to the Schedule of Procedures (Appendices A, B and C).

7.0 STUDY PROCEDURES

7.1 Informed Consent Process

A Master Informed Consent Document consisting of a Participant Information Sheet and a Consent Form is provided by the Sponsor to the trial site. This document is made site-specific and translated (if necessary), submitted and approved by the Institutional Review Board (IRB). The Master and site specific Informed Consent Documents are separate documents and should not be part of the protocol.

Participant Information Sheet
A qualified member of the study staff will conduct the informed consent process by reviewing the Participant Information Sheet and document in the clinic notes.

Consent Form
The participant's consent to participate must be obtained by him/her signing and dating the Consent Form. The person obtaining consent will also sign.

The signed and dated Informed Consent Document must remain at the study site. A copy of the signed/marked and dated Informed Consent Document will be offered to the participant to take home. Those participants who do not wish to take a copy will be required to document that they declined to do so.

7.2 Medical History and Physical Examination

Medical History
At screening, a comprehensive medical history will be collected including previous IV infusions and reaction to IV infusion, history of sexually transmitted infection (STI) and pregnancy prevention practices. At subsequent visits, an interim medical history will be performed.

Physical Examination

General Physical Examination
A general physical examination includes examination of head/ears/eyes/nose and throat, skin, respiratory, cardiovascular, abdominal, limited neurological and musculoskeletal and external ano-genital systems (for HIV-infected participants only) at the time points indicated in the Schedule of Procedures (see Appendices A, B and C).

Symptom-Directed Physical Examination
A symptom-directed physical examination is a targeted examination based on the participant's history or observation. If deemed necessary, this examination should be done at the time points indicated in the schedule of procedures (see Appendices A, B and C).

Measuring Height and Weight
Includes measuring the height and weight at the time points indicated in the Schedule of Procedures (see Appendices A, B and C).

Vital Signs

Vital signs including pulse, respiratory rate, blood pressure and temperature are measured and recorded at the time points indicated in the Schedule of Procedures (see Appendices A, B and C)

7.3 HIV Testing and HIV-test Counselling (Groups 1 and 2)

Study staff will perform pre-HIV test counselling prior to collecting blood for an HIV test, and post-HIV test counselling when HIV test results are available. This is referred to as HIV-test counselling, and done according to the CDC guidelines. For more information on HIV testing and HIV-test counselling, see Section 11.0. A screening questionnaire and other tools may be used.

7.4 HIV Risk Reduction Counselling

HIV risk reduction counselling will be provided to all participants as outlined by site-specific SOPs.

Study staff will provide HIV risk reduction counselling based on reported individual risk and provide free condoms, as appropriate, at every visit. Group 1 and 2 will receive HIV risk reduction counselling and for Groups 3, HIV risk reduction counselling will be conducted as secondary prevention to reduce onward transmission.

7.5 Family Planning Counselling

Study staff will counsel participants about the importance of preventing pregnancies and of using condoms, as well as other effective family planning methods until at least 3 months following investigational product administration, as appropriate. Participants may be referred for family planning services as necessary according to site-specific SOPs as detailed in the SOM. Pregnancy prevention methods chosen and compliance will be documented.

7.6 ART Counselling (Group 3)

HIV-infected participants who are not on ART will receive ART counselling upon entering the study and 8 weeks after infusion of investigational product. Participants who have not initiated or made plans to initiate ART by the final study visit will receive ART counselling again at their final study visit.

7.7 Specimens

Approximately 50 ml of blood will be collected from participants in Group 1 and 2, and approximately 150 ml of blood will be collected from participants in Group 3 at the screening visit. At later visits, approximately 8.5 ml to 175 ml of blood will be collected, depending on study procedures and Group assignment (see Appendices A, B and C), usually from the antecubital fossa.

Optional collection of rectal and/or cervical mucosal secretions will be obtained using a rectal sponge (or comparable swab) or cervical Softcup (or comparable cervical fluid collection cup) for those participants that consent.

All specimens will be handled according to the procedures specified in the AP and relevant SOPs if applicable.

In the event of an abnormal laboratory value, participants may be asked to have an additional sample collected at the discretion of the Principal Investigator or designee.

7.8 Reimbursement

Participants will be reimbursed for their time, effort and for costs to cover their travel expenses to the study site and any inconvenience caused due to study participation. Site specific-reimbursement amounts will be documented in the site-specific Participant Information Sheet, and approved by the Institutional Review Board.

7.9 Randomization and Blinding

Participants will be identified by a unique study identification number.

Participants will be randomized according to the randomization schedule prepared by the statisticians at the Data Coordinating Center (DCC) prior to the start of the study. Participants will be automatically assigned a specific allocation number as they are enrolled into the data entry system. An unblinding list (Pharmacy List) will be provided to the unblinded site pharmacist by the DCC.

This a double blind, randomized, placebo-controlled study for Groups 1 and 2, and an open label study for Group 3. For Groups 1 and 2, study staff (investigator and clinical personnel monitoring the safety and laboratory assay results) and participants will be blinded with respect to the allocation of Investigational Product (PGDM1400 mAb alone or PGDM1400 and PGT121 in combination or placebo). A site pharmacist will be unblinded for the purposes of preparing investigational product.

A participant will be considered enrolled once he/she has been assigned an allocation number.

Blinded participants will be informed about their assignment (active product/placebo) at study completion, once the database is locked. Should a study participant be unblinded during the study, the study participant will be followed up until the end of the study according to the Schedule of Procedures (Appendices A and B).

7.10 Un-blinding Procedure for Individual Participants

Un-blinding of an individual participant may be indicated in the event of a medical emergency if the clinical management of the participant would be altered by knowledge of the treatment assignment.

The un-blinded information should be restricted to a small Group of individuals involved in clinical management/medical treatment of the participant (e.g., treating physician) and the blind must be maintained for those responsible for the study assessments.

The reasons for un-blinding should be documented and the IAVI Chief Medical Officer, the Medical Monitor and the DCC should be notified as soon as possible. The procedures and contact numbers for un-blinding are outlined in the SOM.

7.11 Assessment of PGDM1400 mAb and/or PGT121 mAb related HIV sero-positivity

It is possible that PGDM1400 and/or PGT121 or an immune response to PGDM1400 and/or PGT121 could cause a positive result on a diagnostic HIV antigen/antibody test. A Group 1 or 2 participant who tests HIV antigen and/or HIV antibody positive at the end of the study will have additional testing to distinguish actual HIV infection from investigational product-related responses. The participant will be informed of his/her positive HIV antigen and/or HIV antibody test result and offered continuing follow-up until the HIV antigen/antibody test becomes negative.

8.0 INVESTIGATIONAL PRODUCT

8.1 Description

PGDM1400 and PGT121 active products are formulated in a 20 mM Acetate, 9% Sucrose, 0.008% polysorbate 80, pH 5.2 formulation buffer at a concentration of 50 mg/mL. Each 10 ml vial contains 6 ml of PGDM1400 or PGT121.

A summary of the Investigational Products and example volumes needed for administration for each dose level are shown in Table 8.1-1.

Table 8.1-1 Investigational Products

Active Product / Placebo	Dosage level	Total volume in investigational product container	Total Active Product or placebo volume to be injected into a 100 mL saline IV bag (for an 88 kg body weight**)	Total volume to be Infused (for an 88 kg body weight**)
PGDM1400 (50 mg/mL)	3 mg/kg	6 mL per vial	5.3 mL	105.3 mL
	10 mg/kg		17.6 mL	117.6 mL
	30 mg/kg		52.8 mL	152.8 mL
PGT121 (50 mg/mL)	3 mg/kg	6 mL per vial	5.3 mL	105.3 mL
	10 mg/kg		17.6 mL	117.6 mL
	30 mg/kg		52.8 mL	152.8 mL
Placebo: 0.9% Sodium Chloride for Injection USP (Saline)*	3 mg/kg matching***	NA	5.3 mL***	105.3 mL***
	10 mg/kg matching***		17.6 mL***	117.6 mL***
	30 mg/kg matching***		52.8 mL***	152.8 mL***

* The Placebo provided will be a commercially-available 0.9% sodium chloride for injection USP partial addition IV bag.
** The actual volume to be injected will be based on the dose Subgroup and the weight of the participant at the time of investigational product administration. The example included here is the average weight of an adult male in the US (88kg) (http://www.cdc.gov/nchs/data/series/sr_11/sr11_252.pdf)
*** For placebo IV infusions: saline from an additional IV bag will be injected into the saline IV bag intended for administration, to match the volume used for a PGDM1400 ± PGT121 mAb injection

in the same dose Subgroup, to prevent unblinding. Placebo recipients in Group 2 will receive 2 sequential administrations of placebo to mimic administration of PGDM1400 followed by PGT121 to maintain blinding. See SOM for details.

8.2 Shipment and Storage

Authorization to ship the PGDM1400 and PGT121 to the site will be provided in writing by the Sponsor, upon confirmation that all required critical documents for shipment authorization are completed. PGDM1400 and PGT121 will be shipped maintaining the required storage conditions and stored in a secure location in the clinical site's pharmacy.

PGDM1400 and PGT121 vials will be stored at - 20°C ± 5°C. Each vial will be labelled with the name of the product, lot number, concentration, fill volume, storage temperature, date of manufacture, name and location of the manufacturer and a US cautionary statement. Several such vials will be packaged in a box. Each box will be labelled with similar information as the vial label, including an address and contact information for the manufacturer.

0.9% Sodium Chloride for Injection USP, in partial-addition bags, will be used as the placebo and diluent for PGDM1400 and PGT121. It will be stored at room temperature.

8.3 Preparation of investigational product

Detailed instructions will be provided to the site pharmacist in the SOM for preparing each of the investigational products. The site pharmacist will not be blinded, but the study physician/designee administering the investigational product will be blinded. Infusion of the investigational product(s) should be completed within 4 hours of the PGDM1400 or PGT121 being injected into the saline IV bag. Example calculations for the volumes needed for IV infusion are illustrated in Table 8.1-1. Procedures for handling used and partially used vials of PGDM1400 and PGT121 will be provided in the SOM. Syringes or other components in direct contact with PGDM1400 or PGT121 will be disposed of properly in a biohazard container and incinerated or autoclaved as per site procedure.

8.4 Administration of investigational product

Investigational product will be administered at the enrollment visit. The investigational product will be injected into a 0.9% sodium chloride for injection USP partial addition bag. The participant will receive the investigational product via IV infusion. If more than one investigational product will be administered, these will be administered sequentially in separate saline IV bags. Participants will receive each infusion over approximately 60 minutes, allowing for clinician discretion. Further information on the IV infusion of the investigational product is supplied in the SOM and other study documents.

8.5 Accountability and Disposal of investigational product

All used PGDM1400 and PGT121 vials will be handled according to instructions in the SOM. Throughout the study, the investigational product accountability forms including receipt and dispensing of vials will be kept and monitored. The vial label for the used vial(s) will be removed and retained for accountability purposes; the used vial(s) can be discarded as per site procedures.

At the end of the study, the unused PGDM1400 and PGT121 vials will be reconciled according to Sponsor instructions.

Further information on accountability and disposal of PGDM1400 and PGT121 is supplied in the SOM.

9.0 ASSESSMENTS

9.1 Safety Assessments

Data on local and systemic reactogenicity (i.e., solicited AEs) will be collected by structured interview and medical examination. Data on other adverse events will be collected with open-ended questions. All data will be recorded on the appropriate source documents and entered into the study database. Participants will be given a Memory Aid, which is a tool to assist with collecting reactogenicity data.

Local and systemic reactogenicity events will be assessed by study staff prior to and during IV infusion of investigational product, at approximately 30 minutes after investigational product infusion, at 1 hour after investigational product infusion and subsequently every hour for the first 6 hours post-investigational product infusion. Study staff will review the Memory Aid with the participant, and determine the severity of the reactions on days 1-3 through discussion with the participant.

9.1.1 Local reactogenicity

The presence of local reactogenicity will be assessed at the time points specified in the Schedule of Procedures (Appendices A, B and C).
Pain, tenderness, erythema/skin discoloration, swelling/hardening or pruritus will be assessed and graded using Appendix G, DAIDS Adverse Event Severity Assessment Table, as a guideline.

9.1.2 Systemic reactogenicity

The presence of systemic reactogenicity will be assessed at the time points specified in the Schedule of Procedures (Appendices A, B and C).
Fever, chills, headache, nausea, vomiting, malaise, myalgia and arthralgia will be assessed and graded using the Appendix G DAIDS Adverse Event Severity Assessment Table as a guideline.
For the first 24 hours after investigational product infusion, any infusion related reactions, including cytokine release syndrome, should be graded using the Common Terminology Criteria for Adverse Events (CTCAE) v4.03: June 14, 2010 (Appendix F).

9.1.3 Vital Signs

At the infusion visit, vital signs (pulse, respiratory rate, blood pressure and temperature) will be measured by study staff prior to investigational product infusion, at approximately 30 minutes post investigational product infusion and hourly until 6 hours after IV infusion. For the other study visits vital signs will be assessed at the time points specified in the Schedule of Procedures (Appendices A, B and C).

9.1.4 Other Adverse Events

Other adverse events (AEs) will be collected through 56 days after investigational product infusion in all participants. Serious Adverse Events (SAEs) will be collected throughout the entire study period. Potential Immune Mediated Diseases (pIMDs), as defined in Section 10.5, will be collected throughout the study period, using the SAE reporting process. Open-ended questions will be asked at time points according to the Schedule of Procedures (Appendices A, B and C). All adverse events during the first 24 hours after the infusion will be graded using Appendix G, DAIDS Adverse Event Severity Assessment Table, as a guideline and will be assessed for relatedness to the investigational product. For more information regarding adverse events refer to Section 10.0, Adverse Events.

9.1.5 Concomitant Medications

Concomitant receipt of Investigational Products is prohibited during the study.

Contraceptive use and use of medication at study entry will be documented. (See DCF instructions).

During the study, information regarding concomitant medications and reasons for their use will be solicited from the study participants for 56 days. Ongoing concomitant medications will be recorded until end of study.

9.1.6 Routine laboratory parameters

Table 9.1.6-1 shows the laboratory parameters that will be measured routinely. The samples for these tests will be collected at the time points indicated in the Schedule of Procedures (Appendices A, B and C).

Table 9.1.6-1: Laboratory Parameters

Laboratory Parameter	Test
Hematology and Coagulation	Hemoglobin, hematocrit, leukocytes, platelets, absolute neutrophil count (ANC), absolute lymphocyte count (ALC), activate partial thromboplastin time (aPTT), international normalized ratio (INR)
Clinical Chemistry	Sodium, potassium, creatinine, aspartate aminotransferase (AST), alanine aminotransferase (ALT), total bilirubin, alkaline phosphatase<br>Groups 1 and 2 only: albumin, creatine kinase, C-reactive protein, C3 complement, C4 complement
Urinalysis	Dipstick test for protein, blood glucose, ketones, esterase (leukocytes) and nitrite. If clinically significant abnormalities (e.g., blood, protein, leukocytes) are found on dipstick test, then further test(s) will be performed (e.g., microscopy, culture)
T cell panel (Group 3)	CD4 T cell count and frequency by single platform flow cytometry

9.1.7 Specific screening tests:

Participants will be screened to exclude the following diseases:

- Hepatitis B: positive for hepatitis B surface antigen (HBsAg)
- Hepatitis C: positive for hepatitis C RNA (HCV antibody test, followed by HCV RNA test if HCV antibody positive)
- Active syphilis: confirmed diagnosis.

A negative Hepatitis B and Hepatitis C result can be documented from the medical record only if the result is from a test administered less than 6 months ago.

9.1.8 Monitoring for anti-PGDM1400 and anti-PGT121 antibodies:

Participants will be evaluated for the development of antibodies to PGDM1400 and PGT121 mAb (anti-drug antibodies, ADA) by ELISA according to the Schedule of Procedures (Appendices A, B and C).

9.2 Virologic Assessments

Table 9.2-1 shows the virologic parameters that will be measured routinely. The samples for these tests will be collected at the time points indicated in the Schedule of Procedures (Appendix C).

Table 9.2-1: Virologic Assessment Table

Virologic Parameter	Test
Antiviral Activity	Plasma HIV RNA levels
Anti-reservoir activity	Cell-associated HIV-1 RNA levels in resting CD4 T cells; total HIV-1 DNA and 2-long terminal repeat (LTR) HIV-1 DNA circles in resting or total CD4 T cells; quantitative viral outgrowth assay (qVOA)
Other	Genotyping of plasma HIV RNA for evaluation of PGDM1400 and PGT121-induced escape mutations and resistance to antiretroviral drugs; phenotyping of plasma HIV RNA for neutralization susceptibility to PGDM1400 and PGT121 in-vitro

9.3 Exploratory Immunogenicity Assessments

Humoral immune response assays will include, but are not limited to Env-specific Ab-binding assays, virus neutralization assay, and assays for Ab functionality. Cellular immune response assays will include, but are not limited to IFNγ ELISPOT assay, ICS, and multiparameter flow cytometry. Exploratory assessments on mucosal samples will include, but are not limited to characterization of Env-specific binding Abs. Priority assays are listed below.

9.3.1 Antibody Responses

- Env-specific binding Abs (titers and breadth).
- Env-specific nAbs (titers and breadth).

- Env-specific functional Abs (e.g. phagocytosis score and breadth).
- Env-specific binding Ab isotypes (IgA, IgG1-4) (titers and breadth).

9.3.2 Cellular Responses

- IFNγ peripheral blood mononuclear cell (PBMC) responders to peptide pools and subpools of Potential T-cell epitopes, PTE Env/Gag/Pol peptides.
- $CD4^+$ and $CD8^+$ T-cell functionality (% cells producing e.g., IFNγ, IL-2, IL-4, TNFα).
- T-cell development with emphasis on follicular helper T-cells and memory differentiation.

9.3.3 PBMC, Serum and Plasma Storage

Samples of cryopreserved PBMC, plasma and serum will be stored as indicated in the Schedule of Procedures in Appendices A, B and C (for details see Analytical Plan (AP)) and, if the participant consents, may be used for the purposes of standardization, quality control and for future assays related to HIV prevention or treatment research and development. These samples will be archived and the testing laboratories will be blinded to the participant's identity.

9.4 Other Assessments

9.4.1 HIV Antibody Testing (Groups 1 and 2)

All HIV-uninfected participants (Group 1 and 2) will be tested for HIV antibodies as indicated in the Schedule of Procedures (Appendix A and B) or as needed, if medical or social circumstances arise. All participants will receive HIV risk reduction counselling and pre- and post-HIV-test counselling, as specified in Section 7.3 Counselling.

9.4.2 Pharmacokinetics

Blood draws for pharmacokinetics will be done on the day of investigational product infusion immediately before starting IV infusion(s) of investigational product, at the end of the investigational product infusion(s), and 3, 6 and 24 hours after the investigational product infusions. Thereafter, pharmacokinetic draws will be done as indicated in the Schedule of Procedures (Appendices A, B and C).

PGDM1400 and PGT121 mAb pharmacokinetic analyses will be performed using, but not limited to, standard non-compartmental analysis methods to estimate elimination half-life (t1/2), clearance (CL/F), volume of distribution (Vz/F), Area under the concentration decay curve (AUC), impact of viral load and/or ART on PGDM1400 and PGT121 mAb disposition (elimination half-life (t1/2), clearance (CL/F), volume of distribution (Vz/F) and total exposure. PGDM1400 ± PGT121 accumulation will also be examined in rectal and cervical mucosal secretions collected with rectal sponges (or comparable swab) or cervical Softcups (or comparable cervical fluid collection cup) in study participants who specifically consented for these procedures. Descriptive results will be reported for the pharmacokinetic parameters by dose Subgroup.

Exploratory analysis using population analysis methods simultaneously combining all pharmacokinetic data across all doses and treatment Subgroups and Groups will be performed for quantitative characterization of differences in PGDM1400 and PGT121 mAb disposition by dose, participant Subgroup or Group or disease state.

9.4.3 HLA Typing

Samples for HLA typing will be collected as specified in the Schedule of Procedures in Appendices A, B and C (for details see Analytical Plan (AP)) and may be analyzed as warranted.

9.4.5 Pregnancy Test

A urine pregnancy test for all female participants will be performed by measurement of human chorionic gonadotrophin (βhCG) at time points indicated in the Schedule of Procedures (Appendices A, B and C). The results of the pregnancy test must be negative prior to IV infusion of investigational product. See section 10.7 for description of pregnancy after infusion of investigational product.

9.4.6 HIV Risk Assessment (Group 1 and 2)

Study staff will assess participants for their past and current risk of acquiring HIV at time points indicated in Schedule of Procedures (Appendix A and B).

9.4.7 Social Impact Assessment

A brief assessment of the impact of participation in the study will be administered to participants at their final study visit.

10.0 ADVERSE EVENTS

10.1 Definition

An adverse event (AE) is any untoward medical occurrence in a participant administered an Investigational Product and which does not necessarily have a causal relationship with the Investigational Product. An AE can therefore be any unfavourable or unintended sign (including an abnormal laboratory finding), symptom, or disease, temporally associated with the use of Investigational Product whether or not related to the Investigational Product.

Assessment of severity of all AEs, including and seriousness of AEs, is ultimately the responsibility of the Principal Investigator of each site. Refer to the DIVISION OF AIDS (DAIDS) TABLE FOR GRADING THE SEVERITY OF ADULT AND PEDIATRIC ADVERSE EVENTS Version 2.0, November 2014 and the National Cancer Institute Common Terminology Criteria for Adverse Events (CTCAE) Version 4.03: June 14, 2010 for additional guidance.

10.2 Assessment of Severity of Adverse Events

The following general criteria should be used in assessing adverse events as mild, moderate, severe or very severe at the time of evaluation:

Grade 1 (Mild): Symptoms causing no or minimal interference with usual social & functional activities

Grade 2 (Moderate): Symptoms causing greater than minimal interference with usual social & functional activities

Grade 3 (Severe): Symptoms causing inability to perform usual social & functional activities

Grade 4 (Very Severe): Symptoms causing inability to perform basic self-care functions OR Medical or operative intervention indicated to prevent permanent impairment, persistent disability, or death

Guidelines for assessing the severity of specific adverse events and laboratory abnormalities are listed in Appendix G, DAIDS Adverse Event Severity Assessment Table.

10.3 Relationship to Investigational Product

Assessment of relationship of an AE or SAE to Investigational Product is the responsibility of the Principal Investigator or designee. All medically indicated and available diagnostic methods (e.g., laboratory, blood smear, culture, X-ray, etc.) should be used to assess the nature and cause of the AE/SAE. Best clinical and scientific judgment should be used to assess relationship of AE/SAEs to the investigational product and/or other cause.

The following should be considered:

- Presence/absence of a clear temporal (time) sequence between administration of the investigational product and the onset of AE/SAE
- Presence/absence of another cause that could more likely explain the AE/SAE (concurrent disease, concomitant medication, environmental or toxic factors)
- Whether or not the AE/SAE follows a known response pattern associated with the investigational product

The relationship assessment should be reported as one of the following:

Not Related: clearly explained by another cause (concurrent disease, concomitant medication, environmental or toxic factors, etc.).

Unlikely: more likely explained by another cause (concurrent disease, concomitant medication, environmental or toxic factors, etc.).

Possibly: equally likely explained by another cause but the possibility of the investigational product relationship cannot be ruled out (e.g., reasonably well temporally related and/or follows a known investigational product response pattern but equally well explained by another cause).

Probably: more likely explained by the investigational product (e.g., reasonably well temporally related and/or follows a known investigational product response pattern and less likely explained by another cause).

Definitely: clearly related and most likely explained by the investigational product.

For the purpose of expedited safety reporting, all possibly, probably or definitely related SAEs are considered investigational product-related SAEs.

10.4 Serious Adverse Events

An adverse event is reported as a "Serious Adverse Event" if it meets any of the following criteria (as per International Conference on Harmonisation [ICH] Good Clinical Practice [GCP] Guidelines):

- Results in death
- Is life threatening
- Results in persistent or significant disability/incapacity
- Requires in-participant hospitalization or prolongs existing hospitalization
- Is a congenital anomaly/birth defect or spontaneous abortion
- Any other important medical condition that requires medical or surgical intervention to prevent permanent impairment of a body function or structure

Elective surgery for pre-existing condition that did not increase in severity or frequency is not considered an SAE.

Serious Adverse Events (SAEs) should be reported within 24 hours of the site becoming aware of the event, and sent to the Sponsor as described in the SOM.

To discuss investigational product-related SAEs or any urgent medical questions related to the SAE, the site investigator should contact one of the IAVI Medical Monitors directly (see Contact List in the SOM).

The IAVI SAE Report Form should be completed with all the available information at the time of reporting and sent to the Sponsor as described in the SOM. The minimum data required in reporting an SAE are the study identification number, date of birth, gender, event description (in as much detail as is known at the time), onset date of event (if available), reason event is classified as serious, reporting source (name of Principal Investigator or designee), and relationship to the investigational product as assessed by the investigator.

The Principal Investigator or designee is required to prepare a detailed written report with follow up until resolution or until it is judged by the Principal Investigator or designee to have stabilized.

The Principal Investigator or designee must notify the local IRB/IEC of all SAEs as appropriate. In case of investigational product-related SAEs, the Sponsor will notify responsible regulatory authorities, Safety Monitoring Committee (SMC), and other study sites where the same investigational product is being tested.

More details on SAE definitions and reporting requirements are provided in the SOM.

<u>Serious Event Prior to Investigational Product Administration</u>

If a serious event occurs in the period between the participant signing the Informed Consent Form and receiving the IV infusion of investigational product, the event will be reported using the SAE form and following the same procedures for SAE reporting, as indicated in Section 10.4. The timing of the event will be indicated by using the relevant checkbox on the SAE form.

10.5 Reporting Potential Immune-Mediated Diseases

Potential immune-mediated diseases (pIMDs) are a subset of AEs that include both clearly autoimmune diseases and also other inflammatory and/or neurologic disorders that may or may not have an autoimmune etiology. These events are of special interest since they could potentially be caused by immune responses to the investigational product. The investigator/designee should report such adverse events within the same time limits (following confirmation of an AE as a pIMD; see last paragraph of this section below), and using the same CRF pages, as utilized for SAEs. The investigator or his/her designee will evaluate the occurrence of pIMDs at every visit/contact during the study. IAVI will also expect investigators/designee to provide additional information about pIMD events. AEs to be reported and documented as pIMDs include:

Neuroinflammatory disorders: optic neuritis, cranial nerve disorders (including Bell's palsy), multiple sclerosis, demyelinating disease, transverse myelitis, Guillain-Barré syndrome, myasthenia gravis, encephalitis, neuritis.

Musculoskeletal disorders: systemic lupus erythematosus, cutaneous lupus, Sjögren's syndrome, scleroderma, dermatomyositis, polymyositis, myopathy, rheumatoid arthritis and juvenile rheumatoid arthritis, polymyalgia rheumatica or temporal arteritis, reactive arthritis, psoriatic arthropathy, ankylosing spondylitis, undifferentiated spondyloarthropathy.

Gastrointestinal disorders: Crohn's disease, ulcerative colitis or proctitis, celiac disease.

Metabolic diseases: autoimmune thyroiditis, Grave's or Basedow's disease, Hashimoto thyroiditis, insulin-dependent diabetes mellitus (IDDM), Addison's disease.

Skin disorders: psoriasis, vitiligo, Raynaud's phenomenon, erythema nodosum, autoimmune bullous skin diseases.

Others: autoimmune hemolytic anemia, thrombocytopenia, antiphospholipid syndrome, *vasculitis, pernicious anemia, autoimmune hepatitis, primary biliary cirrhosis, primary sclerosing cholangitis, autoimmune glomerulonephritis, autoimmune uveitis, autoimmune myocarditis/cardiomyopathy, sarcoidosis, Stevens-Johnson syndrome, Behçet's syndrome.

Infusion site reactions: Grade 3 or 4 infusion site reactions lasting more than 2 days.

*Vasculitis: Vasculitis, Diffuse vasculitis, leucocytoclastic vasculitis, polyarteritis nodosa, microscopic polyangiitis, Wegener's granulomatosis, anti-neutrophil cytoplasmic antibody positive vasculitis, Henoch-schönlein purpura, allergic granulomatous angiitis (Churg-Strauss disease), Kawasaki disease, Takayasu's arteritis, temporal arteritis (giant cell arteritis), renal vasculitis.

Medical judgement should be exercised in deciding whether other disorders/diseases have an autoimmune origin and should also be reported as described above, and this judgement is the investigator's prerogative. Whenever sufficient data exist to substantiate any of the diagnoses in the above list, the event must be reported as a pIMD. While the intent of pIMD reporting is to be inclusive, isolated nonspecific symptoms, which might (or might not) represent the above diagnoses, should be captured as AEs but not reported as pIMDs until the diagnosis can be defended.

10.6 Clinical Management of Adverse Events

Adverse events (AEs) will be managed by the clinical study team who will assess, provide first line of care as appropriate and refer to health care and treatment facilities as warranted. If any treatment/medical care is required as a result of the harm caused by the investigational product or study procedures, this will be provided free of charge.

If a participant has an AE and/or abnormal laboratory value that is known at the time of IV infusion of investigational product, the specifications of Section 12.0 will be followed.

Participants will be followed until the AE resolves or stabilizes or up to the end of the study, whichever comes last. If at the end of the study, an AE (including clinically significant laboratory abnormality) that is considered possibly, probably or definitely related to the investigational product is unresolved, follow-up will continue until resolution if possible and/or the participant will be referred.

If a participant from Group 3 experiences a significant decrease in CD4 cell count (e.g., – 20% of baseline, or decline to <200 cells/µL) during the course of the trial, participants will be monitored closely until their CD4 count returns to baseline or until the participant initiates ART. Participants whose CD4 cell counts decrease to <200 cells/µL will be promptly informed and will be referred to their primary HIV care provider. Appropriate prophylaxis against opportunistic infections will be instituted according to accepted U.S. HIV treatment guidelines.

10.7 Pregnancy

Although not considered an AE, if a female participant becomes pregnant during the study, it is the responsibility of the Principal Investigator or designee to report the pregnancy promptly to IAVI using the designated forms. The participant will be followed for safety until the end of pregnancy or study completion, whichever occurs last. If possible, approximately 2–4 weeks after delivery, the baby will be examined by a physician to assess its health status and the results will be reported to the Sponsor. The baby will be examined again by a Physician around age 1, and the results will be reported to the Sponsor.

Complications of pregnancy that meet criteria for SAEs, specified in Section 10.4 of this Protocol (e.g., hospitalization for eclampsia, spontaneous abortion, etc.) should be reported as SAEs.

10.8 Intercurrent HIV Infection (Group 1 and 2)

HIV infection cannot be directly caused by the investigational product. If a participant acquires HIV through exposure in the community, at any time after the IV infusion of investigational product, the participant should be offered referral to appropriate care and treatment facilities. The participant will continue to be followed in the study for safety assessments.

Intercurrent HIV infection in study participants, although not considered an SAE, must be reported promptly to IAVI using the designated forms. However, medical conditions associated with the HIV infection that meet criteria for being serious specified in the Section 10.4 of this Protocol (e.g., sepsis, *Pneumocystis jiroveci [carinii] pneumonia*, etc.) should be reported as SAEs using the SAE Report Form.

11.0 MANAGEMENT OF HIV ISSUES DURING AND FOLLOWING STUDY

11.1 HIV Testing – Groups 1 and 2

Group 1 and 2 participants will be tested for HIV antibodies as indicated in the Schedule of Procedures (Appendix A and B) or as needed, if medical or social circumstances arise. All participants will receive HIV risk reduction counselling and pre- and post-HIV-test counselling, as specified in Section 11.3.1, Counselling (Group 1 and 2).

It is possible that the Investigational Product(s) or an immune response to the Investigational Product(s) could cause a positive result on a diagnostic HIV antibody test. An investigational product recipient who falsely tests HIV positive with a diagnostic HIV antibody test at the end of the study will be informed of his/her positive test result and offered continuing follow-up until the test becomes negative.

If a participant acquires HIV through exposure in the community, at any time after the administration of investigational product, the participant will be offered referral to appropriate care and treatment facilities. The participant will continue to be followed in the study for safety assessments.

Should a participant require HIV testing outside of the study for personal reasons, it is recommended that the participant contact the study staff first. HIV testing can be done at the study site and then processed at an independent laboratory as above. Written evidence of HIV status (HIV-infected or HIV-uninfected) will be provided upon request.

11.2 Social Discrimination as a Result of investigational product-related antibodies

In order to minimize the possibility of social discrimination in participants (if any) who test positive on a diagnostic HIV antibody test due to investigational product-related antibodies, appropriate diagnostic HIV testing and certification will be provided both during and after the study as needed.

11.3 HIV infection – Group 1 and 2

Group 1 and 2 participants who are diagnosed with HIV infection at screening or during the study (intercurrent HIV-infection) will be provided the following:

11.3.1 Counselling

The participant will be counselled by the study investigators or designated counsellors. The counselling process will assist the participant with the following issues:

- Psychological and social implications of HIV infection
- Who to inform and what to say
- Implications for sexual partners
- Implications for child-bearing
- Avoidance of transmission to others in future
- Mandatory reporting to the state, in some instances

11.3.2 Referral for Support/Care

Participants will be referred to a participant support center or institution of his/her choice for a full discussion of the clinical aspects of HIV infection. Referral will be made to a designated physician or center

12.0 WITHDRAWAL FROM STUDY

12.1 Deferral of IV infusion of investigational product

An IV infusion of investigational product may be temporarily deferred if the participant is clinically ill at the time of the administration of investigational product visit and/or presents with fever ($\geq$ 100.4 °F) at the time of the administration of investigational product. A participant must be clinically well and afebrile for a minimum of a 24-hour consecutive period prior to administration of investigational product.

Any planned or unplanned deferral of infusion of investigational product will be discussed with the Sponsor. Participants will be deferred from infusion of investigational product for any of the following reasons:

- Pregnancy
- A disease or condition or adverse event that may develop, regardless of relationship to Investigational Product, if the Principal Investigator or designee is of the opinion that administration of investigational product will jeopardize the safety of the participant
- Participant's request to defer infusion

The following events require resolution and/or review of clinical history by the Principal Investigator or designee and consultation with the Medical Monitor, prior to administration of investigational product:

- Any abnormal laboratory value, as outlined in section 5.7, Exclusion Criteria, Hematology, Chemistry, Urinalysis that is known at the time of infusion and have not resolved.
- Receipt of inactivated/killed/subunit vaccines (non-HIV) or immunoglobulin within the previous 14 days. Receipt of live attenuated vaccines within the previous 30 days.
- Participating in another clinical study of an Investigational Product

12.2 Withdrawal from the Study (Early Termination)

Participants may be withdrawn from the study permanently for the following reasons:

1. Participants may withdraw from the study at any time if they wish, for any reason
2. The Principal Investigator or designee has reason to believe that the participant is not complying with the protocol
3. If the Sponsor decides to terminate or suspend the study

If a participant withdraws or is withdrawn from the study, all termination visit procedures will be performed according to the Schedule of Procedures (Appendices A, B and C) where possible. Every effort will be made to determine and document the reason for withdrawal.

13.0 DATA HANDLING

13.1 Data Collection and Record Keeping at the Study Site

Data Collection: All study data will be collected by the clinical study staff using designated source documents and entered onto the appropriate electronic CRFs (eCRFs). Access to eCRFs will be provided via an electronic data entry system hosted by the Data Coordination Center. All study data must be verifiable to the source documentation. A file will be held for each participant at the clinic(s) containing all the source documents. Source documentation will be available for review to ensure that the collected data are consistent with the CRFs.

All CRFs and laboratory reports will be reviewed by the clinical team, who will ensure that they are accurate and complete.

Source documents and other supporting documents will be kept in a secure location. Standard GCP practices will be followed to ensure accurate, reliable and consistent data collection.

Source documents include but are not limited to:

- Signed Informed Consent Documents
- Progress notes
- Data collection forms
- Documentation of any existing conditions or past conditions relevant to eligibility
- Printed laboratory results
- Print out of the generated enrollment confirmation
- All Adverse Events
- Concomitant medications
- Local and systemic reactogenicity events

13.2 Data Entry at the Study Site

The data collected at the site will be recorded onto the eCRFs by the study staff and entered into a database. To provide for real time assessment of safety, data should be entered as soon as reasonably feasible after a visit occurs.

13.3 Data Analysis

The Sponsor, PIs and Product Developers will agree on how data will be analyzed and presented prior to unblinding of the study.

The DCC will conduct the data analysis and will provide interim safety and final study reports for the Sponsor, Principal Investigators, the PSRT and SMC and the regulatory authorities, as appropriate.

14.0 STATISTICAL CONSIDERATIONS

14.1 Safety and Tolerability Analysis

14.1.1 Sample Size

The sample size for safety and tolerability analysis will be 36-84 participants according to the dose escalation design used to characterize the safety profile of one IV infusion of PGDM1400 ± PGT121 mAb, at one of three dose levels.

14.1.2 Null Hypothesis

As this is an exploratory proof of concept trial and analysis will be descriptive, no formal null hypothesis will be tested.

14.1.3 Statistical Power and Analysis and Dose Escalation Rules

The frequency (percentage) of moderate or greater local and systemic reactogenicity events (along with 95% confidence intervals) will be determined for each active Group and placebo.

The frequency of SAEs judged possibly, probably or definitely related to the investigational product will be determined.

All AEs will be analyzed and grouped by seriousness, severity and relationship to the investigational product (as judged by the investigator).

For life-threatening adverse events related to investigational product: if none of the 18 (max 36) participants receiving active product experience such reactions, then the exact 95 % upper confidence bound for the rate of these adverse events in the population is 18.5% (or 9.7% if n=36).

All AEs will be analysed and grouped by seriousness, severity and relationship to the investigational product (as judged by the investigator).

For life-threatening adverse events related to active product: if none of the 9 (max 18) participants in either Group 1 or Group 2 who receive the active product experience such reactions then the exact 95% upper confidence bound for the rate of these adverse events in the population is 33.6% (or 18.5% if n=18).

An interim analysis of Subgroup and Group data will be carried out according to the study schema (Table 5.3.1) without unblinding the study to investigators or participants. At the end of the study, a full analysis will be prepared.

Based on previous experience with IAVI Phase 1 investigational product studies, it is expected that the amount of missing, unused or spurious data will be insignificant. Unused and spurious data will be listed separately and excluded from the statistical analysis. Missing data will be considered missing completely at random and excluded from the statistical analysis.

14.2 Pharmacokinetic Analysis

14.2.1 Sample Size

The sample size for pharmacokinetic analysis will be 3 per dose Subgroup, to provide sufficient information for the planned analyses.

14.2.2 Null Hypothesis

As this is an exploratory proof of concept trial and analysis will be descriptive, no formal null hypothesis will be tested.

14.2.3 Statistical Power and Analysis

Disposition of PGDM1400 mAb and PGT121 mAb will be evaluated in this study. Based on the PK profile of other human monoclonal antibodies, it is expected that the half-life of PGDM1400 mAb and PGT121 mAb will be 14 to 21 days. Previously published data indicate that the pharmacokinetics of VRC01, 3BNC117 and 10-1074 are fairly similar across phase 1 studies (Table).

Table 14.2.3-1: Key parameters of select Antibodies for the Prevention and Treatment of HIV Infection

	**3BNC117 (Caskey, Klein et al. 2015)**	**VRC01 (Ledgerwood, Coates et al. 2015, Lynch, Boritz et al. 2015)**	**10-1074 (Caskey, Schoofs et al. 2017)**
**Binding site on HIV ENV spike**	CD4 binding site	CD4 binding site	V3 loop
**Human safety data available**	Yes, safe and well tolerated	Yes, safe and well tolerated	Yes, safe and well tolerated
**PK HIV-uninfected, terminal half life (days)**	17	15	24
**PK HIV-infected & viremic, terminal half life (days)**	9	12 (i.v.), 11 (s.c.)	12.8
**Antiviral effect (average or range $\log_{10}$ decrease HIV RNA)**	1.48	1.1-1.8	1.52

Commonly reported PK parameters will be calculated using, but not limited to, standard non-compartmental slope/height/area/moment (SHAM) analysis methods. Summary descriptive results of PK parameters, including AUC, Cmax, T1/2, and clearance results will be reported by dose cohort. Dose normalized plots of PK parameters will be presented. Correlation between PK and reported safety and pharmacodynamic

outcomes will also be explored parameters in order to examine exposure-effect relationships.

A more powerful exploratory analysis to quantitatively determine the dose, participant and disease impact on PGDM1400 mAb and PGT121 mAb pharmacokinetics, and correlate exposure with response, while correctly accounting for variance based on population intrinsic factors such as weight and gender will be performed. Using the proposed population analysis approach we will be able to simultaneously examine the magnitude and the rate of change to PGDM1400 and PGT121 disposition driven by HIV-1 RNA levels, and also examine the magnitude and the rate of decline in log copies/ml of HIV-1 RNA plasma levels from baseline.

The frequency and levels of anti-PGDM1400 antibodies and anti-PGT121 antibodies will be calculated and tabulated.

14.3. Virologic Analysis for Group 3

14.3.1 Sample Size

The sample size for virologic analysis in Groups 3A and 3B will be 12-36 participants according to the design described below.

14.3.2 Null Hypothesis

The null hypothesis is that there is a mixture of responders and non-responders with mixture probability 0.5 such that the difference-from-baseline is greater than -0.9 logs HIV RNA viral load in the responder group and 0 logs HIV RNA viral load in the non-responder group.

14.3.3 Statistical Power and Analysis

The virologic analysis described in this section relates to Subgroups 3A and 3B of the study design, in which antiviral activity of PGDM1400 mAb alone and a combination of PGDM1400 mAb + PGT121 mAb is measured in HIV-infected participants off ART with plasma HIV RNA levels of 1×10^3 – $1x\ 10^5$ copies/ml. This section assumes that Part 1 of the study has successfully demonstrated that there are safe dose levels of PGDM1400 mAb, and PGDM1400 mab + PGT121 mAb. No placebo participants are enrolled as part of this design.

The primary efficacy outcome for this analysis is defined as change in log10 viral load between Day 0 (day of infusion) and Day 7. The minimum clinically significant value for this outcome is defined as a difference of –0.9 log10.

Groups 3A: this Group will enroll participants sequentially. After administering PGDM1400 at the MTD, as defined in part 1 of this study, the day 7 post-infusion viral load measurement will be compared to the baseline viral load measurement. If the difference in viral load at 7 days post-infusion is greater than a 0.5 log10 drop, the participant is categorized as a responder; otherwise, the participant is categorized as a non-responder. Enrollment will continue until at least 6 responders or 18 total participants have been enrolled whichever occurs first.

Group 3B: this Group will enroll participants sequentially. After administering PGDM1400 mAb and PGT121 mAb at the MTD, as defined in part 1 of this study, the day 7 post-infusion viral load measurement will be compared to the baseline viral load

measurement. If the difference in viral load at 7 days post-infusion is greater than a 0.5 log10 drop, the participant is categorized as a responder; otherwise, the participant is categorized as a non-responder. Enrollment will continue until at least 6 responders or 18 total participants have been enrolled whichever occurs first.

Antiviral activity in Subgroup 3A and 3B will be tested in the responder group only using a one-sided Wilcoxon signed rank test against the null hypothesis of a "shift" parameter of -0.9 $\log_{10}$. Based on a simulation study outlined in the SAP, the power to reject the null hypothesis is 80% when the responder group has a difference-from-baseline viral load drop of approximately 1.8 logs for a nominal alpha level of 0.05. Since the responder group is defined by the outcome it is not surprising that the Type I error is greater than the nominal alpha level which was deemed an acceptable trade-off between sample size and power to detect the desired effect under the alternative described above. Notably, the Type I error drops below the nominal error rate when the true shift in viral load is in the responder group is greater than -0.5 logs.

For the analysis of sample size and power, log10 viral load differences from baseline for each participant were simulated from a normal distribution, with a standard deviation of 0.5. This value was chosen by examining a study of the antiretroviral drug raltegravir, which demonstrated a mean estimated standard deviation of the change of baseline of 0.47(Andrade, Rosenkranz et al. 2013). This is a conservative estimate, as the variability of viral loads near the lower range might be expected to also be lower.

The statistical test performed will be the Signed-ranktest, which will incorporate the “shift” parameter of –0.9 log10 (the minimum clinically significant difference selected for this study). An evaluation of potential harm (increased viral load) will also be performed with the Signed ranktest; this test will examine the null hypothesis of no change in viral load (a shift of 0.0 log10 following investigational product administration) against the one-sided alternative hypothesis that the viral load is increased following investigational product administration. Each efficacy test will be performed at the level $\alpha = 0.05$. Each test for harm will be performed at level $2\alpha = 0.10$, in order to provide additional sensitivity to detect potential harm.

14.5 Secondary and Exploratory Immunologic and Virologic Analyses

14.5.1 Sample Size

The sample size for secondary immunologic and/or virologic analysis will be the 12-36 HIV infected participants in part 2 of the study.

14.5.2 Null Hypothesis

No formal hypothesis on immunologic or virologic responses will be tested, with the exception of the change in HIV viral load described in Section 14.3.

14.5.3 Statistical Power and Analysis

Descriptive statistics (actual values and changes from reference) will be calculated for continuous immunologic and virologic parameters at all time points. Graphical representations of changes in parameters will be made as applicable. Differences between Subgroups and/or Groups at a specific time point will be tested for exploratory purposes by a 2-sample t-test if the data appear to be normally distributed (after transformation if necessary). If not, the non-parametric Wilcoxon rank sum test will be used. If portions of the measurements are censored below the assay quantification limit,

the Gehan-Wilcoxon test will be employed. All statistical tests will be two-sided and will be considered statistically significant if $p < 0.05$.

Frequency tabulations will be calculated for discrete (qualitative) immunologic and virologic parameters at all time points. Significant differences between Subgroups and/or Groups will be determined by a 2-sided Fisher's exact test.

Interim immunologic and virologic analyses of grouped data may be performed without unblinding the study to investigators or participants.

15.0 QUALITY CONTROL AND QUALITY ASSURANCE

To ensure the quality and reliability of the data collected and generated and the ethical conduct of this study, a Study Operations Manual (SOM) will be developed. All deviations will be reported and investigated. The SOM describes reporting and deviation documentation requirements and procedures.

Regular monitoring will be performed according to ICH-GCP as indicated in Section 17.5.

An independent audit of the study and study sites may be performed by the Sponsor or designee to establish the status of applicable quality systems. Inspection by regulatory authorities may also occur.

By signing the protocol, the Principal Investigators agree to facilitate study related monitoring, audits, IRB/IEC review and regulatory inspection(s) and direct access to source documents. Such information will be treated as strictly confidential and under no circumstances be made publicly available.

16.0 DATA AND BIOLOGICAL MATERIAL

All data and biological material collected through the study shall be managed in accordance with the Clinical Trial Agreement (CTA). Distribution and use of these data will be conducted by agreement of all parties.

The computerized raw data generated will be held by the DCC on behalf of the Sponsor. The study sites will also hold the final data files and tables generated for the purpose of analysis.

17.0 ADMINISTRATIVE STRUCTURE

The Principal Investigator will be responsible for all aspects of the study at the study site.

17.1 Protocol Safety Review Team

A PSRT will be formed to monitor the clinical safety data. During the administration of investigational productphase of the trial, the PSRT will review the clinical safety data on a weekly basis via electronic distribution of reports. An ad hoc PSRT review meeting will occur if any of the members of the PSRT requests a special review to discuss a specific safety issue or as specified in the Study Operations Manual. After the administration of

investigational product phase the PSRT will review the clinical safety data at least monthly.

The PSRT will consist of the IAVI Medical Monitor(s), and the PI or designee from each clinical team. The study chair or an IAVI Medical Monitor may be the PSRT chair. *Ex officio* members will include the IAVI Chief Medical Officer and an unblinded IAVI Medical Monitor. Additional PSRT participants may include the following, as needed:

- Co-investigators and trial site senior clinical research nursing staff
- Laboratory directors
- Data management, study statistician and regulatory staff

The PSRT membership and procedures are detailed in the PSRT charter.

17.2 Safety Monitoring Committee (SMC)

The SMC will consist of independent clinicians/scientists/statisticians/ethicists who are not involved in the study. Investigators responsible for the clinical care of participants or representative of the Sponsor may not be a member of the SMC. Details of membership, chair and co-chair and responsibilities are outlined in the SMC charter.

Principal Investigator(s) or designee and/or a Sponsor representative may be asked to join an open session of the SMC meeting to provide information on study conduct, present data or to respond to questions.

Safety data will be reviewed by the SMC at pre-specified time points and at an ad-hoc basis.

17.2.1 Content of Interim Safety Review

The SMC will be asked to review the following blinded data:

- Summary of reactogenicity (i.e., solicited adverse events)
- All adverse events judged by the Principal Investigator or designee to be possibly, probably or definitely related to investigational product
- All laboratory results confirmed on retest and judged by the Principal Investigator or designee to be clinically significant
- All SAEs and pIMDs

An unblinded presentation of all above noted events may also be made available for the SMC for their review if required by any member of the SMC.

17.2.2 SMC Review of Group 1 and 2 data prior to starting Group 3

Following IV infusion of investigational product of the last participant in Groups 1 and/or 2, the Safety Monitoring Committee (SMC) will review safety data through the day 14 post-IV infusion visit for all participants to confirm MTD in each Group, and determine whether, and at what dose level, Groups 3A and 3B can initiate enrollment. See section 5.3 for additional details.

17.3 Criteria for Pausing the Study

Enrollment and administration of investigational product will be stopped and a safety review conducted by the SMC for any of the following criteria:

- One or more participants experience an SAE that is judged possibly, probably or definitely related to investigational product.
- There is a participant death, regardless of relationship to the investigational product.
- Two or more participants experience Grade 3 adverse events in the same category System Organ Class that are considered possibly, probably or definitely related to investigational product or
- Any Grade 4 adverse event that is considered possibly, probably or definitely related to investigational product.

Table 17.3-1: AE notification and safety pause/AE review rules

Event and relationship to study product	Severity	Occurrence	Site PI action	PSRT or SMC action
SAE, possibly, probably or definitely related	Any	Any	Phone, email or fax forms to sponsor within 24 hours	Study pause within 24 hours, refer to SMC for review
SAE, probably not or not related	Death	Any	Phone, email or fax forms to sponsor within 24 hours	Study pause within 24 hours, refer to SMC for review
AE, possibly, probably or definitely related	Grade 4	Any	Phone, email or fax notification to sponsor within 24 hours	Study pause within 24 hours, refer to SMC for review
AE¶, possibly, probably or definitely related	Grade 3*	First	Phone, email or fax notification to sponsor within 24 hours	PSRT review within 2 business days to consider pause
AE¶, possibly, probably or definitely related	Grade 3*	Second‡	Phone, email or fax notification to sponsor within 24 hours	Study pause within 24 hours, refer to SMC for review

¶Does not include the following reactogenicity symptoms (fever, fatigue/malaise, myalgia, arthralgia, chills, headache, nausea, vomiting).

*If no evidence of disease is present other than an abnormal laboratory value, the test must be repeated with a new blood sample at least one time within 72 hours after the investigator becoming aware of the abnormal laboratory value. When signs and symptoms are present, repeat test will not be needed.

‡PSRT will determine whether the reported related AE (Grade 3) is a second occurrence of a previously reported AE (Grade 3).

The Sponsor will request a review by the SMC, (or the SMC chair if other SMC members cannot be convened), to be held within 2 business days of the Sponsor learning of the event. The individual participant(s)/or study may be unblinded at the discretion of the SMC. Following this review, the SMC will make a recommendation regarding the continuation or suspension of the administration of the investigational product or the trial and communicate this decision immediately to the Sponsor. The Sponsor then will inform the Principal Investigators without delay.

Additional *ad hoc* review may be specifically requested by the Sponsor, the Principal Investigator(s) or by the SMC.

17.4 Study Supervision

The SMC, the IAVI Chief Medical Officer (CMO) and the IAVI Medical Monitor(s) have access to progress report(s) of this study. Close cooperation will be necessary to track study progress, respond to queries about proper study implementation and management, address issues in a timely manner, and assure consistent documentation, and share information effectively. Rates of accrual, retention, and other parameters relevant to the site's performance will be regularly and closely monitored by the study team.

17.5 Study Monitoring

On-and/or off-site monitoring will ensure that the study is conducted in compliance with human subjects' protection and other research regulations and guidelines, recorded and reported in accordance with the protocol, is consistent with SOPs, GCP, applicable regulatory requirements and locally accepted practices. The monitor will confirm the quality and accuracy of data at the site by validation of CRFs against the source documents, such as clinical records. The investigators, as well as participants through consenting to the study, agree that the monitor may inspect study facilities and source records (e.g., informed consent forms, clinic and laboratory records, other source documents), as well as observe the performance of study procedures (in accordance with site IRB requirements). Such information will be treated as strictly confidential and will under no circumstances be made publicly available.

The monitoring will adhere to GCP guidelines. The Principal Investigator will permit inspection of the facilities and all study-related documentation by authorized representatives of IAVI, and Government and Regulatory Authorities responsible for this study.

17.6 Investigator's Records

Study records include administrative documentation—e.g., reports and correspondence relating to the study—as well as documentation related to each participant screened and/or enrolled in the study—including informed consent forms, case report forms, and all other source documents. The investigator will maintain and store, in a secure manner, complete, accurate, and current study records for a minimum of 2 years after marketing application approval or the study is discontinued and applicable national and local health authorities are notified. IAVI will notify the Principal Investigator of these events.

18.0 INDEMNITY

The Sponsor and Institution are responsible to have appropriate liability insurance. For research-related injuries and/or medical problems determined to result from receiving the investigational product, treatment including necessary emergency treatment and proper follow-up care will be made available to the participant free of charge at the expense of the Sponsor.

19.0 PUBLICATION

A primary manuscript describing safety, anti-viral effect and immune responses in this trial will be prepared promptly after the data analysis is available.

Authors will be representatives of each trial site, the data management and statistical analysis center, the laboratories, the product developer and the sponsor, participant to the generally accepted criteria of contributions to the design and conduct of the study, the analysis of data and writing of the manuscript. Precedence will be given to authors from the site enrolling the greatest number of participants. Manuscripts will be reviewed by representatives of each participating group as specified in the CTA**.**

20.0 ETHICAL CONSIDERATIONS

The Principal Investigator will ensure that the study is conducted in compliance with the protocol, SOPs in accordance with guidelines formulated by the ICH for GCP in clinical studies, the ethical principles that have their origins in the Declaration of Helsinki and applicable local standards and regulatory requirements.

APPENDIX A: SCHEDULE OF PROCEDURES – GROUP 1 (A, B, C)

**Study Month**		**0**							**1**		**2**		**3**	**4**	**5**	**6**
**Study Week**		**0**				**1**	**2**	**3**	**4**	**6**	**8**	**10**	**12**	**16**	**20**	**24**
**Study Day**	**Scr**	**0**	**1**	**2**	**3**	**7**	**14**	**21**	**28**	**42**	**56**	**70**	**84**	**112**	**140**	**168/ET^**
**Visit Windows (Days)**	**-56**	**0**	**0**	**0**	**0**	**± 1**	**± 2**	**± 2**	**± 2**	**± 3**	**± 3**	**±3**	**±7**	**±7**	**±7**	**±7**
**INVESTIGATIONAL PRODUCT**																
Investigational Product		**X**														
**CONSENT/ASSESSMENTS/COUNSELLING**																
Informed Consent	**X**															
Assessment of Understanding	**X**															
HIV Risk Assessment	**X**															**X**
HIV Risk Reduction Counselling	**X**	**X**							**X**		**X**		**X**	**X**	**X**	**X**
HIV-test Counselling	**X**	**X**							**X**							**X**
Family Planning Counselling	**X**	**X**														
Social Impact Assessment																**X**
**CLINICAL SAFETY ASSESSMENTS**																
Comprehensive Medical History	**X**															
Interim Medical History		**X**	**X**	**X**	**X**	**X**	**X**	**X**	**X**	**X**	**X**					
Concomitant Medications	**X**	**X**	**X**	**X**	**X**	**X**	**X**	**X**	**X**	**X**	**X**					
General Physical Exam	**X**															**X**
Directed Physical Exam		**X**	**X**	**X**	**X**	**X**	**X**	**X**	**X**	**X**	**X**	**X**	**X**	**X**	**X**	
Weight	**X**	**X**														**X**
Height	**X**															
Vital Signs	**X**	**X***	**X**	**X**	**X**	**X**	**X**	**X**	**X**	**X**	**X**	**X**	**X**	**X**	**X**	**X**
Local & Systemic Reactogenicity		**X***	**X**	**X**	**X**											
Adverse Events		**X**	**X**	**X**	**X**	**X**	**X**	**X**	**X**	**X**	**X**					
Serious Adverse Events and pIMD	**X**	**X**	**X**	**X**	**X**	**X**	**X**	**X**	**X**	**X**	**X**	**X**	**X**	**X**	**X**	**X**

Study Month		0							1		2		3	4	5	6
**Study Week**		**0**				**1**	**2**	**3**	**4**	**6**	**8**	**10**	**12**	**16**	**20**	**24**
**Study Day**	**Scr**	**0**	**1**	**2**	**3**	**7**	**14**	**21**	**28**	**42**	**56**	**70**	**84**	**112**	**140**	**168/ET^**
**Visit Windows (Days)**	**-56**	**0**	**0**	**0**	**0**	**± 1**	**± 2**	**± 2**	**± 2**	**± 3**	**± 3**	**±3**	**±7**	**±7**	**±7**	**±7**
**CLINICAL LABORATORY TESTS**																
Hematology and Coagulation	X	X#	X		X	X	X		X		X		X	X	X	X
Clinical Chemistry	X	X#	X		X	X	X		X		X		X	X	X	X
Urine Dipstick	X	X#	X		X	X	X		X		X		X	X	X	X
Urine Pregnancy test	X	X#							X		X		X			X
Active Syphilis	X															
Hepatitis B	X															
Hepatitis C	X															
HIV screen (4th generation Ag/Ab test)	X															
Blinded HIV diagnostic testing		X#							X							X
**RESEARCH LABORATORY TESTS**																
Anti PGDM1400 and anti-PGT121 Antibodies (ADA)		X#							X		X		X			X
Humoral Assays**		X#			X	X	X		X		X		X			X
Cellular Assays**		X#					X		X		X		X			X
HLA typing		X#														
**PHARMACOKINETICS PGDM1400 ± PGT121 ELISA**		X#	X	X	X	X	X	X	X	X	X	X	X	X	X	X
**MUCOSAL SAMPLING**		X~	X			X	X									
**PLASMA/SERUM STORAGE**		X	X	X	X	X	X	X	X		X		X			X
**PBMC STORAGE**		X									X		X			X

Day 0 baseline sample collections must be done before infusion of investigational product. Additional day 0 pharmacokinetics sample collection will be done as outlined in Protocol section 9.4.2.
^ Early Termination (ET): Procedures to be performed at ET are the same as last visit procedures
* At baseline, approximately 30 minutes after IP administration and then every hour until 6 hours after IV infusion. Local and systemic reactogenicity will be assessed by clinic staff at visits on study days 1, 2 and 3. Local and Systemic reactogenicity will also be assessed by the participant using the Memory Aid on study days 1, 2, and 3.
** See Laboratory Analytical Plan for details
~ Cervico-vaginal and/or rectal mucosal sampling (optional) on Day 0 must be done prior to IV infusion of IP.

APPENDIX B: SCHEDULE OF PROCEDURES – AND GROUP 2 (A, B, C)

**Study Month**		**0**							**1**		**2**		**3**	**4**	**5**	**6**
**Study Week**		**0**				**1**	**2**	**3**	**4**	**6**	**8**	**10**	**12**	**16**	**20**	**24**
**Study Day**	**Scr**	**0**	**1**	**2**	**3**	**7**	**14**	**21**	**28**	**42**	**56**	**70**	**84**	**112**	**140**	**168/ET^**
**Visit Windows (Days)**	**-56**	**0**	**0**	**0**	**0**	**± 1**	**± 2**	**± 2**	**± 2**	**± 3**	**± 3**	**±3**	**±7**	**±7**	**±7**	**±7**
**INVESTIGATIONAL PRODUCT**																
Investigational Product		**X**														
**CONSENT/ASSESSMENTS/COUNSELLING**																
Informed Consent	**X**															
Assessment of Understanding	**X**															
HIV Risk Assessment	**X**															**X**
HIV Risk Reduction Counselling	**X**	**X**							**X**		**X**		**X**	**X**	**X**	**X**
HIV-test Counselling	**X**	**X**							**X**							**X**
Family Planning Counselling	**X**	**X**														
Social Impact Assessment																**X**
**CLINICAL SAFETY ASSESSMENTS**																
Comprehensive Medical History	**X**															
Interim Medical History		**X**	**X**	**X**	**X**	**X**	**X**	**X**	**X**	**X**	**X**					
Concomitant Medications	**X**	**X**	**X**	**X**	**X**	**X**	**X**	**X**	**X**	**X**	**X**					
General Physical Exam	**X**															**X**
Directed Physical Exam		**X**	**X**	**X**	**X**	**X**	**X**	**X**	**X**	**X**	**X**	**X**	**X**	**X**	**X**	
Weight	**X**	**X**														**X**
Height	**X**															
Vital Signs	**X**	**X***	**X**	**X**	**X**	**X**	**X**	**X**	**X**	**X**	**X**	**X**	**X**	**X**	**X**	**X**
Local & Systemic Reactogenicity		**X***	**X**	**X**	**X**											
Adverse Events		**X**	**X**	**X**	**X**	**X**	**X**	**X**	**X**	**X**	**X**					
Serious Adverse Events and pIMD	**X**	**X**	**X**	**X**	**X**	**X**	**X**	**X**	**X**	**X**	**X**	**X**	**X**	**X**	**X**	**X**
**CLINICAL LABORATORY TESTS**																
Hematology and Coagulation	**X**	**X#**	**X**		**X**	**X**	**X**		**X**		**X**		**X**	**X**	**X**	**X**

**Study Month**		**0**							**1**		**2**		**3**	**4**	**5**	**6**
**Study Week**		**0**				**1**	**2**	**3**	**4**	**6**	**8**	**10**	**12**	**16**	**20**	**24**
**Study Day**	**Scr**	**0**	**1**	**2**	**3**	**7**	**14**	**21**	**28**	**42**	**56**	**70**	**84**	**112**	**140**	**168/ET[^]**
**Visit Windows (Days)**	**-56**	**0**	**0**	**0**	**0**	**± 1**	**± 2**	**± 2**	**± 2**	**± 3**	**± 3**	**±3**	**±7**	**±7**	**±7**	**±7**
Clinical Chemistry	X	X[#]	X		X	X	X		X		X		X	X	X	X
Urine Dipstick	X	X[#]	X		X	X	X		X		X		X	X	X	X
Urine Pregnancy test	X	X[#]							X		X		X			X
Active Syphilis	X															
Hepatitis B	X															
Hepatitis C	X															
HIV screen (4th generation Ag/Ab test)	X															
Blinded HIV diagnostic testing		X[#]							X							X
**RESEARCH LABORATORY TESTS**																
Anti PGDM1400 and anti-PGT121 Antibodies (ADA)		X[#]							X		X		X			X
Humoral Assays**		X[#]			X	X	X		X		X		X			X
Cellular Assays**		X[#]					X		X		X		X			X
HLA typing		X[#]														
**PHARMACOKINETICS PGDM1400 ± PGT121 ELISA**		X[#]	X	X	X	X	X	X	X	X	X	X	X	X	X	X
**MUCOSAL SAMPLING**		X[~]	X			X	X									
**PLASMA/SERUM STORAGE**		X	X	X	X	X	X	X	X		X		X			X
**PBMCs STORAGE**		X									X		X			X

Day 0 baseline sample collections must be done before infusion of investigational product. Additional day 0 pharmacokinetics sample collection will be done as outlined in Protocol section 9.4.2.
^ Early Termination (ET): Procedures to be performed at ET are the same as last visit procedures
* At baseline, approximately 30 minutes after IP administration and then every hour until 6 hours after IV infusion. Local and systemic reactogenicity will be assessed by clinic staff at visits on study days 1, 2 and 3. Local and Systemic reactogenicity will also be assessed by the participant using the Memory Aid on study days 1, 2, and 3.
** See Laboratory Analytical Plan for details
~ Cervico-vaginal and/or rectal mucosal sampling (optional) on Day 0 must be done prior to IV infusion of IP.

APPENDIX C: SCHEDULE OF PROCEDURES – GROUP 3 (A, B)

Study Month		0								1		2		3	4	5	6
**Study Week**		**0**				**1**		**2**	**3**	**4**	**6**	**8**	**10**	**12**	**16**	**20**	**24**
**Study Day**	**Scr**	**0**	**1**	**2**	**3**	**7**	**10**	**14**	**21**	**28**	**42**	**56**	**70**	**84**	**112**	**140**	**168/ET**
**Visit Windows (Days)**	**-42**	**0**	**0**	**0**	**0**	**± 1**	**0**	**± 2**	**± 2**	**± 2**	**± 3**	**± 3**	**±3**	**±7**	**±7**	**±7**	**±7**
**INVESTIGATIONAL PRODUCT**																	
Investigational Product		X															
**CONSENT/ASSESSMENTS/COUNSELLING**																	
Informed Consent	X																
Assessment of Understanding	X																
HIV Risk Reduction Counselling	X	X								X		X		X	X	X	X
ART counselling	X	X										X					X
Family Planning Counselling	X	X															
Social Impact Assessment																	X
**CLINICAL SAFETY ASSESSMENTS**																	
Comprehensive Medical History	X																
Interim Medical History		X	X	X	X	X	X	X	X	X	X	X					
Concomitant Medications	X	X	X	X	X	X	X	X	X	X	X	X					
General Physical Exam	X																X
Directed Physical Exam		X	X	X	X	X	X	X	X	X	X	X	X	X	X	X	
Weight	X	X															X
Height	X																
Vital Signs	X	X*	X	X	X	X	X	X	X	X	X	X	X	X	X	X	X
Local & Systemic Reactogenicity		X*	X	X	X												
Adverse Events		X	X	X	X	X	X	X	X	X	X	X					
Serious Adverse Events and pIMD	X	X	X	X	X	X	X	X	X	X	X	X	X	X	X	X	X
**CLINICAL LABORATORY TESTS**																	
Hematology and Coagulation	X	X#	X		X	X		X		X		X		X	X	X	X
CD4	X	X#				X		X		X		X					X

Study Month		0								1		2		3	4	5	6
**Study Week**		**0**				**1**		**2**	**3**	**4**	**6**	**8**	**10**	**12**	**16**	**20**	**24**
**Study Day**	**Scr**	**0**	**1**	**2**	**3**	**7**	**10**	**14**	**21**	**28**	**42**	**56**	**70**	**84**	**112**	**140**	**168/ET**
**Visit Windows (Days)**	**-42**	**0**	**0**	**0**	**0**	**± 1**	**0**	**± 2**	**± 2**	**± 2**	**± 3**	**± 3**	**±3**	**±7**	**±7**	**±7**	**±7**
Clinical Chemistry	X	X#	X		X	X		X		X		X		X	X	X	X
Urine Dipstick	X	X#	X		X	X		X		X		X		X	X	X	X
Urine Pregnancy test	X	X#								X		X		X			X
Active Syphilis	X																
Hepatitis B	X																
Hepatitis C	X																
HIV 4th generation Ag/Ab test***	X																
HIV Viral Load	X	X#	X	X	X	X	X	X	X	X	X	X	X	X	X	X	X
**RESEARCH LABORATORY TESTS**	-	-	-	-	-	-	-	-	-	-	-	-	-	-	-	-	-
Anti PGDM1400 and anti-PGT121 Antibodies (ADA)		X#								X		X		X			X
HIV phenotypic testing for PGDM1400 ± PGT121 susceptibility	X									X							X
HIV SGA sequencing	X									X							X
HIV genotypic testing for ART resistance	X									X				X			X
HIV reservoir size assessment	X							X						X			
Humoral Assays**		X#			X	X		X		X		X		X			X
Cellular Assays**		X#						X		X		X		X			X
HLA typing		X#															
**PHARMACOKINETICS PGDM1400 ± PGT121 ELISA**	X	X#	X	X	X	X		X	X	X	X	X	X	X	X	X	X
**MUCOSAL SAMPLING**		X~	X			X		X									
**PLASMA/SERUM STORAGE**	X	X	X	X	X	X	X	X	X	X		X		X			X
**PBMCs STORAGE**		X										X		X			X

Day 0 baseline sample collections must be done before infusion of investigational product. Additional day 0 pharmacokinetics sample collection will be done as outlined in Protocol section 9.4.2.
^ Early Termination (ET): Procedures to be performed at ET are the same as last visit procedures

* At baseline, approximately 30 minutes after IP administration and then every hour until 6 hours after IV infusion. Local and systemic reactogenicity will be assessed by clinic staff at visits on study days 1, 2 and 3. Local and Systemic reactogenicity will also be assessed by the participant using the Memory Aid on study days 1, 2, and 3.
** See Laboratory Analytical Plan for details
*** Confirmed HIV-1 infection (HIV Ab+ or HIV RNA+) by documentation in the medical records or in-clinic HIV testing;
~ Cervico-vaginal and/or rectal mucosal sampling (optional) on Day 0 must be done prior to IV infusion of IP.

APPENDIX D: LOW RISK CRITERIA

Low risk will be defined as:

1. **SEXUAL BEHAVIORS**

In the **last 12 months** did not:

- Have oral, vaginal or anal intercourse with an HIV-infected partner, or partner who uses injection drugs.
- Gave or receive money, drugs, gifts, or services in exchange for oral, vaginal or anal sex

  AND

  In the **last 6 months** has abstained from penile/anal or penile/vaginal intercourse

  OR

  In the **last 6 months**:

- Had 4 or fewer partners of the opposite birth sex for vaginal and/or anal intercourse, OR

Is MSM (person born male with partner(s) born male) who, in the **last 12 months**:

- Had 2 or fewer MSM partners for anal intercourse and had no unprotected anal sex with MSM, OR
- Had unprotected anal intercourse with only 1 MSM partner, within a monogamous relationship lasting at least 12 months (during which neither partner had any other partners). If the monogamous relationship ended, the participant may then have had protected anal intercourse with 1 other MSM partner (total 2 or fewer partners in the last 12 months).

Is a transgender person, regardless of the point on the transition spectrum, having sex with men (born male) and/or other transgender persons, who in the last 12 months:

- Had 2 or fewer partners for anal or vaginal intercourse, and had no unprotected anal or vaginal sex, OR
- Had unprotected anal or vaginal intercourse sex with 1 partner only within a monogamous relationship lasting at least 12 months (during which neither partner had any other partners). If the monogamous relationship ended, may then have had protected anal or vaginal sex with one other partner (total 2 or fewer partners in the last 12 months).

  AND

  Uses or intends to use condoms in situations which may include penile/anal or penile/vaginal intercourse with new partners of unknown HIV status, occasional partners, partners outside a primary relationship, and/or partners known to have other partners.

2. **NON-SEXUAL BEHAVIORS**

   In the **last 12 months** did not:

- Inject drugs or other substances without a prescription

- Use cocaine, methamphetamine, or excessive alcohol, which in the investigator's judgement, rendered the participant at greater than low risk for acquiring HIV infection

  The investigator's judgement should consider local epidemiologic information about HIV prevalence in the area and community networks.

  A participant is NOT appropriate for inclusion if he/she:

  Acquired an STI (i.e., new infection) in the last 12 months:

- Syphilis
- Gonorrhea
- Non-gonococcal urethritis
- HSV-2
- Chlamydia
- Pelvic inflammatory disease (PID)
- Trichomonas
- Mucopurulent cervicitis
- Epididymitis
- Proctitis
- Lymphogranuloma venereum
- Chancroid
- Hepatitis B

APPENDIX E REFERENCES

Andrade, A., S. L. Rosenkranz, A. R. Cillo, D. Lu, E. S. Daar, J. M. Jacobson, M. Lederman, E. P. Acosta, T. Campbell, J. Feinberg, C. Flexner, J. W. Mellors, D. R. Kuritzkes and A. C. T. G. A. Team (2013). "Three distinct phases of HIV-1 RNA decay in treatment-naive patients receiving raltegravir-based antiretroviral therapy: ACTG A5248." J Infect Dis **208**(6): 884-891.

Bar, K. J., M. C. Sneller, L. J. Harrison, J. S. Justement, E. T. Overton, M. E. Petrone, D. B. Salantes, C. A. Seamon, B. Scheinfeld, R. W. Kwan, G. H. Learn, M. A. Proschan, E. F. Kreider, J. Blazkova, M. Bardsley, E. W. Refsland, M. Messer, K. E. Clarridge, N. B. Tustin, P. J. Madden, K. Oden, S. J. O'Dell, B. Jarocki, A. R. Shiakolas, R. L. Tressler, N. A. Doria-Rose, R. T. Bailer, J. E. Ledgerwood, E. V. Capparelli, R. M. Lynch, B. S. Graham, S. Moir, R. A. Koup, J. R. Mascola, J. A. Hoxie, A. S. Fauci, P. Tebas and T. W. Chun (2016). "Effect of HIV Antibody VRC01 on Viral Rebound after Treatment Interruption." N Engl J Med **375**(21): 2037-2050.

Barouch, D. H. and S. G. Deeks (2014). "Immunologic strategies for HIV-1 remission and eradication." Science **345**(6193): 169-174.

Barouch, D. H., J. B. Whitney, B. Moldt, F. Klein, T. Y. Oliveira, J. Liu, K. E. Stephenson, H. W. Chang, K. Shekhar, S. Gupta, J. P. Nkolola, M. S. Seaman, K. M. Smith, E. N. Borducchi, C. Cabral, J. Y. Smith, S. Blackmore, S. Sanisetty, J. R. Perry, M. Beck, M. G. Lewis, W. Rinaldi, A. K. Chakraborty, P. Poignard, M. C. Nussenzweig and D. R. Burton (2013). "Therapeutic efficacy of potent neutralizing HIV-1-specific monoclonal antibodies in SHIV-infected rhesus monkeys." Nature **503**(7475): 224-228.

Burton, D. R. and J. R. Mascola (2015). "Antibody responses to envelope glycoproteins in HIV-1 infection." Nat Immunol **16**(6): 571-576.

Caskey, M., F. Klein, J. C. Lorenzi, M. S. Seaman, A. P. West, Jr., N. Buckley, G. Kremer, L. Nogueira, M. Braunschweig, J. F. Scheid, J. A. Horwitz, I. Shimeliovich, S. Ben-Avraham, M. Witmer-Pack, M. Platten, C. Lehmann, L. A. Burke, T. Hawthorne, R. J. Gorelick, B. D. Walker, T. Keler, R. M. Gulick, G. Fatkenheuer, S. J. Schlesinger and M. C. Nussenzweig (2015). "Viraemia suppressed in HIV-1-infected humans by broadly neutralizing antibody 3BNC117." Nature **522**(7557): 487-491.

Caskey, M., T. Schoofs, H. Gruell, A. Settler, T. Karagounis, E. F. Kreider, B. Murrell, N. Pfeifer, L. Nogueira, T. Y. Oliveira, G. H. Learn, Y. Z. Cohen, C. Lehmann, D. Gillor, I. Shimeliovich, C. Unson-O'Brien, D. Weiland, A. Robles, T. Kummerle, C. Wyen, R. Levin, M. Witmer-Pack, K. Eren, C. Ignacio, S. Kiss, A. P. West, Jr., H. Mouquet, B. S. Zingman, R. M. Gulick, T. Keler, P. J. Bjorkman, M. S. Seaman, B. H. Hahn, G. Fatkenheuer, S. J. Schlesinger, M. C. Nussenzweig and F. Klein (2017). "Antibody 10-1074 suppresses viremia in HIV-1-infected individuals." Nat Med.

CDC (2014). "CDC. Vital Signs: HIV Diagnosis, Care, and Treatment Among Persons Living with HIV-United States 2011." MMWR **4**(63): 1-6.

Haynes, B. F. and M. J. McElrath (2013). "Progress in HIV-1 vaccine development." Curr Opin HIV AIDS **8**(4): 326-332.

Hessell, A. J., P. Poignard, M. Hunter, L. Hangartner, D. M. Tehrani, W. K. Bleeker, P. W. Parren, P. A. Marx and D. R. Burton (2009). "Effective, low-titer antibody protection against low-dose repeated mucosal SHIV challenge in macaques." Nat Med **15**(8): 951-954.

Hessell, A. J., E. G. Rakasz, P. Poignard, L. Hangartner, G. Landucci, D. N. Forthal, W. C. Koff, D. I. Watkins and D. R. Burton (2009). "Broadly neutralizing human anti-HIV antibody 2G12 is effective in protection against mucosal SHIV challenge even at low serum neutralizing titers." PLoS Pathog **5**(5): e1000433.

Jardine, J., J. P. Julien, S. Menis, T. Ota, O. Kalyuzhniy, A. McGuire, D. Sok, P. S. Huang, S. MacPherson, M. Jones, T. Nieusma, J. Mathison, D. Baker, A. B. Ward, D. R. Burton, L. Stamatatos, D. Nemazee, I. A. Wilson and W. R. Schief (2013). "Rational HIV immunogen design to target specific germline B cell receptors." Science **340**(6133): 711-716.

Ledgerwood, J. E., E. E. Coates, G. Yamshchikov, J. G. Saunders, L. Holman, M. E. Enama, A. DeZure, R. M. Lynch, I. Gordon, S. Plummer, C. S. Hendel, A. Pegu, M. Conan-Cibotti, S. Sitar, R. T. Bailer, S. Narpala, A. McDermott, M. Louder, S. O'Dell, S. Mohan, J. P. Pandey, R. M. Schwartz, Z. Hu, R. A. Koup, E. Capparelli, J. R. Mascola, B. S. Graham and V. R. C. S. Team (2015). "Safety, pharmacokinetics and neutralization of the broadly neutralizing HIV-1 human monoclonal antibody VRC01 in healthy adults." Clin Exp Immunol.

Lynch, R. M., E. Boritz, E. E. Coates, A. DeZure, P. Madden, P. Costner, M. E. Enama, S. Plummer, L. Holman, C. S. Hendel, I. Gordon, J. Casazza, M. Conan-Cibotti, S. A. Migueles, R. Tressler, R. T. Bailer, A. McDermott, S. Narpala, S. O'Dell, G. Wolf, J. D. Lifson, B. A. Freemire, R. J. Gorelick, J. P. Pandey, S. Mohan, N. Chomont, R. Fromentin, T. W. Chun, A. S. Fauci, R. M. Schwartz, R. A. Koup, D. C. Douek, Z. Hu, E. Capparelli, B. S. Graham, J. R. Mascola, J. E. Ledgerwood and V. R. C. S. Team (2015). "Virologic effects of broadly neutralizing antibody VRC01 administration during chronic HIV-1 infection." Sci Transl Med **7**(319): 319ra206.

Moldt, B., E. G. Rakasz, N. Schultz, P. Y. Chan-Hui, K. Swiderek, K. L. Weisgrau, S. M. Piaskowski, Z. Bergman, D. I. Watkins, P. Poignard and D. R. Burton (2012). "Highly potent HIV-specific antibody neutralization in vitro translates into effective protection against mucosal SHIV challenge in vivo." Proc Natl Acad Sci U S A **109**(46): 18921-18925.

Scheid, J. F., J. A. Horwitz, Y. Bar-On, E. F. Kreider, C. L. Lu, J. C. Lorenzi, A. Feldmann, M. Braunschweig, L. Nogueira, T. Oliveira, I. Shimeliovich, R. Patel, L. Burke, Y. Z. Cohen, S. Hadrigan, A. Settler, M. Witmer-Pack, A. P. West, Jr., B. Juelg, T. Keler, T. Hawthorne, B. Zingman, R. M. Gulick, N. Pfeifer, G. H. Learn, M. S. Seaman, P. J. Bjorkman, F. Klein, S. J. Schlesinger, B. D. Walker, B. H. Hahn and M. C. Nussenzweig (2016). "HIV-1 antibody 3BNC117 suppresses viral rebound in humans during treatment interruption." Nature **535**(7613): 556-560.

Scheid, J. F., H. Mouquet, B. Ueberheide, R. Diskin, F. Klein, T. Y. Oliveira, J. Pietzsch, D. Fenyo, A. Abadir, K. Velinzon, A. Hurley, S. Myung, F. Boulad, P. Poignard, D. R. Burton, F. Pereyra, D. D. Ho, B. D. Walker, M. S. Seaman, P. J. Bjorkman, B. T. Chait and M. C. Nussenzweig (2011). "Sequence and structural convergence of broad and potent HIV antibodies that mimic CD4 binding." Science **333**(6049): 1633-1637.

Schoofs, T., F. Klein, M. Braunschweig, E. F. Kreider, A. Feldmann, L. Nogueira, T. Oliveira, J. C. Lorenzi, E. H. Parrish, G. H. Learn, A. P. West, Jr., P. J. Bjorkman, S. J. Schlesinger, M. S. Seaman, J. Czartoski, M. J. McElrath, N. Pfeifer, B. H. Hahn, M. Caskey and M. C. Nussenzweig (2016). "HIV-1 therapy with monoclonal antibody 3BNC117 elicits host immune responses against HIV-1." Science **352**(6288): 997-1001.

Simek, M. D., W. Rida, F. H. Priddy, P. Pung, E. Carrow, D. S. Laufer, J. K. Lehrman, M. Boaz, T. Tarragona-Fiol, G. Miiro, J. Birungi, A. Pozniak, D. A. McPhee, O. Manigart, E. Karita, A. Inwoley, W. Jaoko, J. Dehovitz, L. G. Bekker, P. Pitisuttithum, R. Paris, L. M. Walker, P. Poignard, T. Wrin, P. E. Fast, D. R. Burton and W. C. Koff (2009). "Human immunodeficiency virus type 1 elite neutralizers: individuals with broad and potent neutralizing activity identified by using a high-throughput neutralization assay together with an analytical selection algorithm." J Virol **83**(14): 7337-7348.

Sok, D., K. J. Doores, B. Briney, K. M. Le, K. L. Saye-Francisco, A. Ramos, D. W. Kulp, J. P. Julien, S. Menis, L. Wickramasinghe, M. S. Seaman, W. R. Schief, I. A. Wilson, P. Poignard and D. R. Burton (2014). "Promiscuous glycan site recognition by antibodies to the high-mannose patch of gp120 broadens neutralization of HIV." Sci Transl Med **6**(236): 236ra263.

Sok, D., M. J. van Gils, M. Pauthner, J. P. Julien, K. L. Saye-Francisco, J. Hsueh, B. Briney, J. H. Lee, K. M. Le, P. S. Lee, Y. Hua, M. S. Seaman, J. P. Moore, A. B. Ward, I. A. Wilson, R. W. Sanders and D. R. Burton (2014). "Recombinant HIV envelope trimer selects for quaternary-dependent antibodies targeting the trimer apex." Proc Natl Acad Sci U S A **111**(49): 17624-17629.

UNAIDS (2016). "UNAIDS Fact Sheet November 2016."

Walker LM, B. D. (2010). "Rational antibody-based HIV-1 vaccine design: current approaches and future directions. ." Curr Opin Immunol. **22**(3): 358-366.

Walker, L. M., M. Huber, K. J. Doores, E. Falkowska, R. Pejchal, J. P. Julien, S. K. Wang, A. Ramos, P. Y. Chan-Hui, M. Moyle, J. L. Mitcham, P. W. Hammond, O. A. Olsen, P. Phung, S. Fling, C. H. Wong, S. Phogat, T. Wrin, M. D. Simek, G. P. I. Protocol, W. C. Koff, I. A. Wilson, D. R. Burton and P. Poignard (2011). "Broad neutralization coverage of HIV by multiple highly potent antibodies." Nature **477**(7365): 466-470.

Walker, L. M., S. K. Phogat, P. Y. Chan-Hui, D. Wagner, P. Phung, J. L. Goss, T. Wrin, M. D. Simek, S. Fling, J. L. Mitcham, J. K. Lehrman, F. H. Priddy, O. A. Olsen, S. M. Frey, P. W. Hammond, G. P. I. Protocol, S. Kaminsky, T. Zamb, M. Moyle, W. C. Koff, P. Poignard and D. R. Burton (2009). "Broad and potent neutralizing antibodies from an African donor reveal a new HIV-1 vaccine target." Science **326**(5950): 285-289.

APPENDIX F CTCAE TABLE

CTCAE4.03 Relevant For T002
Common Terminology Criteria for Adverse Events (CTCAE)
Version 4.0 Published: May 28, 2009 (v4.03: June 14, 2010)

U.S. DEPARTMENT OF HEALTH AND HUMAN SERVICES National Institutes of Health National Cancer Institute

Quick Reference

The NCI Common Terminology Criteria for Adverse Events is a descriptive terminology which can be utilized for Adverse Event (AE) reporting. A grading (severity) scale is provided for each AE term.

Components and Organization

SOC

System Organ Class, the highest level of the MedDRA hierarchy, is identified by anatomical or physiological system, etiology, or purpose (e.g., SOC Investigations for laboratory test results). CTCAE terms are grouped by MedDRA Primary SOCs. Within each SOC, AEs are listed and accompanied by descriptions of severity (Grade).

CTCAE Terms

An Adverse Event (AE) is any unfavorable and unintended sign (including an abnormal laboratory finding), symptom, or disease temporally associated with the use of a medical treatment or procedure that may or may not be considered related to the medical treatment or procedure. An AE is a term that is a unique representation of a specific event used for medical documentation and scientific analyses. Each CTCAE v4.0 term is a MedDRA LLT (Lowest Level Term).

Definitions

A brief definition is provided to clarify the meaning of each AE term.

Grades

Grade refers to the severity of the AE. The CTCAE displays Grades 1 through 5 with unique clinical descriptions of severity for each AE based on this general guideline:

Grade 1	Mild; asymptomatic or mild symptoms; clinical or diagnostic observations only; intervention not indicated.
Grade 2	Moderate; minimal, local or noninvasive intervention indicated; limiting age-appropriate instrumental ADL*.
Grade 3	Severe or medically significant but not immediately life-threatening; hospitalization or prolongation of hospitalization indicated; disabling; limiting self care ADL**.

Grade 4	Life-threatening consequences; urgent intervention indicated.
Grade 5	Death related to AE.

A Semi-colon indicates ‘or’ within the description of the grade.

A single dash (-) indicates a grade is not available.

Not all Grades are appropriate for all AEs. Therefore, some AEs are listed with fewer than five options for Grade selection.

Grade 5

Grade 5 (Death) is not appropriate for some AEs and therefore is not an option.

Activities of Daily Living (ADL)

*Instrumental ADL refer to preparing meals, shopping for groceries or clothes, using the telephone, managing money, etc.

**Self care ADL refer to bathing, dressing and undressing, feeding self, using the toilet, taking medications, and not bedridden.

† CTCAE v4.0 incorporates certain elements of the MedDRA terminology. For further details on MedDRA refer to the MedDRA MSSO Web site (http://www.meddramsso.com).

MedDRA v12.0 Code	CTCAE v4.0 SOC	CTCAE v4.0 Term	Grade 1	Grade 2	Grade 3	Grade 4	Grade 5	CTCAE v4.0 AE Term Definition
10001718	Immune system disorders	Allergic reaction	Transient flushing or rash, drug fever <38 degrees C (<100.4 degrees F); intervention not indicated	Intervention or infusion interruption indicated; responds promptly to symptomatic treatment (e.g., antihistamines, NSAIDS, narcotics); prophylactic medications indicated for <=24 hrs	Prolonged (e.g., not rapidly responsive to symptomatic medication and/or brief interruption of infusion); recurrence of symptoms following initial improvement; hospitalization indicated for clinical sequelae (e.g., renal impairment, pulmonary infiltrates)	Life-threatening consequences; urgent intervention indicated	Death	A disorder characterized by an adverse local or general response from exposure to an allergen.
10002218	Immune system disorders	Anaphylaxis	-	-	Symptomatic bronchospasm, with or without urticaria; parenteral intervention indicated; allergy-related edema/angioedema; hypotension	Life-threatening consequences; urgent intervention indicated	Death	A disorder characterized by an acute inflammatory reaction resulting from the release of histamine and histamine-like substances from mast cells, causing a hypersensitivity immune response. Clinically, it presents with breathing difficulty, dizziness, hypotension, cyanosis and loss of consciousness and may lead to death.

MedDRA v12.0 Code	CTCAE v4.0 SOC	CTCAE v4.0 Term	Grade 1	Grade 2	Grade 3	Grade 4	Grade 5	CTCAE v4.0 AE Term Definition
10003239	Musculoskeletal and connective tissue disorders	Arthralgia	Mild pain	Moderate pain; limiting instrumental ADL	Severe pain; limiting self care ADL	-	-	A disorder characterized by a sensation of marked discomfort in a joint.
10008531	General disorders and administration site conditions	Chills	Mild sensation of cold; shivering; chattering of teeth	Moderate tremor of the entire body; narcotics indicated	Severe or prolonged, not responsive to narcotics	-	-	A disorder characterized by a sensation of cold that often marks a physiologic response to sweating after a fever.
10052015	Immune system disorders	Cytokine release syndrome	Mild reaction; infusion interruption not indicated; intervention not indicated	Therapy or infusion interruption indicated but responds promptly to symptomatic treatment (e.g., antihistamines, NSAIDS, narcotics, IV fluids); prophylactic medications indicated for <=24 hrs	Prolonged (e.g., not rapidly responsive to symptomatic medication and/or brief interruption of infusion); recurrence of symptoms following initial improvement; hospitalization indicated for clinical sequelae (e.g., renal impairment, pulmonary infiltrates)	Life-threatening consequences; pressor or ventilatory support indicated	Death	A disorder characterized by nausea, headache, tachycardia, hypotension, rash, and shortness of breath; it is caused by the release of cytokines from the cells.
10013573	Nervous system disorders	Dizziness	Mild unsteadiness or sensation of movement	Moderate unsteadiness or sensation of movement; limiting instrumental ADL	Severe unsteadiness or sensation of movement; limiting self care ADL	-	-	A disorder characterized by a disturbing sensation of lightheadedness, unsteadiness, giddiness, spinning or rocking.
10013963	Respiratory, thoracic and mediastinal disorders	Dyspnea	Shortness of breath with moderate exertion	Shortness of breath with minimal exertion; limiting instrumental ADL	Shortness of breath at rest; limiting self care ADL	Life-threatening consequences; urgent intervention indicated	Death	A disorder characterized by an uncomfortable sensation of difficulty breathing.

MedDRA v12.0 Code	CTCAE v4.0 SOC	CTCAE v4.0 Term	Grade 1	Grade 2	Grade 3	Grade 4	Grade 5	CTCAE v4.0 AE Term Definition
10015218	Skin and subcutaneous tissue disorders	Erythema multiforme	Target lesions covering <10% BSA and not associated with skin tenderness	Target lesions covering 10 - 30% BSA and associated with skin tenderness	Target lesions covering >30% BSA and associated with oral or genital erosions	Target lesions covering >30% BSA; associated with fluid or electrolyte abnormalities; ICU care or burn unit indicated	Death	A disorder characterized by target lesions (a pink-red ring around a pale center).
10016558	General disorders and administration site conditions	Fever	38.0 - 39.0 degrees C (100.4 - 102.2 degrees F)	>39.0 - 40.0 degrees C (102.3 - 104.0 degrees F)	>40.0 degrees C (>104.0 degrees F) for <=24 hrs	>40.0 degrees C (>104.0 degrees F) for >24 hrs	Death	A disorder characterized by elevation of the body's temperature above the upper limit of normal.
10016825	Vascular disorders	Flushing	Asymptomatic; clinical or diagnostic observations only; intervention not indicated	Moderate symptoms; medical intervention indicated; limiting instrumental ADL	Symptomatic, associated with hypotension and/or tachycardia; limiting self care ADL	-	-	A disorder characterized by episodic reddening of the face.
10019211	Nervous system disorders	Headache	Mild pain	Moderate pain; limiting instrumental ADL	Severe pain; limiting self care ADL	-	-	A disorder characterized by a sensation of marked discomfort in various parts of the head, not confined to the area of distribution of any nerve.

MedDRA v12.0 Code	CTCAE v4.0 SOC	CTCAE v4.0 Term	Grade 1	Grade 2	Grade 3	Grade 4	Grade 5	CTCAE v4.0 AE Term Definition
10020772	Vascular disorders	Hypertension	Prehypertension (systolic BP 120 - 139 mm Hg or diastolic BP 80 - 89 mm Hg)	Stage 1 hypertension (systolic BP 140 - 159 mm Hg or diastolic BP 90 - 99 mm Hg); medical intervention indicated; recurrent or persistent (>=24 hrs); symptomatic increase by >20 mm Hg (diastolic) or to >140/90 mm Hg if previously WNL; monotherapy indicated	Stage 2 hypertension (systolic BP >=160 mm Hg or diastolic BP >=100 mm Hg); medical intervention indicated; more than one drug or more intensive therapy than previously used indicated	Life-threatening consequences (e.g., malignant hypertension, transient or permanent neurologic deficit, hypertensive crisis); urgent intervention indicated	Death	A disorder characterized by a pathological increase in blood pressure; a repeatedly elevation in the blood pressure exceeding 140 over 90 mm Hg.
10021097	Vascular disorders	Hypotension	Asymptomatic, intervention not indicated	Non-urgent medical intervention indicated	Medical intervention or hospitalization indicated	Life-threatening and urgent intervention indicated	Death	A disorder characterized by a blood pressure that is below the normal expected for an individual in a given environment.
10051792	General disorders and administration site conditions	Infusion related reaction	Mild transient reaction; infusion interruption not indicated; intervention not indicated	Therapy or infusion interruption indicated but responds promptly to symptomatic treatment (e.g., antihistamines, NSAIDS, narcotics, IV fluids); prophylactic medications indicated for <=24 hrs	Prolonged (e.g., not rapidly responsive to symptomatic medication and/or brief interruption of infusion); recurrence of symptoms following initial improvement; hospitalization indicated for clinical sequelae	Life-threatening consequences; urgent intervention indicated	Death	A disorder characterized by adverse reaction to the infusion of pharmacological or biological substances.

MedDRA v12.0 Code	CTCAE v4.0 SOC	CTCAE v4.0 Term	Grade 1	Grade 2	Grade 3	Grade 4	Grade 5	CTCAE v4.0 AE Term Definition
10064774	General disorders and administration site conditions	Infusion site extravasation	-	Erythema with associated symptoms (e.g., edema, pain, induration, phlebitis)	Ulceration or necrosis; severe tissue damage; operative intervention indicated	Life-threatening consequences; urgent intervention indicated	Death	A disorder characterized by leakage of a pharmacologic or a biologic substance from the infusion site into the surrounding tissue. Signs and symptoms include induration, erythema, swelling, burning sensation and marked discomfort at the infusion site.
10022095	General disorders and administration site conditions	Injection site reaction	Tenderness with or without associated symptoms (e.g., warmth, erythema, itching)	Pain; lipodystrophy; edema; phlebitis	Ulceration or necrosis; severe tissue damage; operative intervention indicated	Life-threatening consequences; urgent intervention indicated	Death	A disorder characterized by an intense adverse reaction (usually immunologic) developing at the site of an injection.
10025482	General disorders and administration site conditions	Malaise	Uneasiness or lack of well being	Uneasiness or lack of well being; limiting instrumental ADL	-	-	-	A disorder characterized by a feeling of general discomfort or uneasiness, an out-of-sorts feeling.
10028411	Musculoskeletal and connective tissue disorders	Myalgia	Mild pain	Moderate pain; limiting instrumental ADL	Severe pain; limiting self care ADL	-	-	A disorder characterized by marked discomfort sensation originating from a muscle or group of muscles.

MedDRA v12.0 Code	CTCAE v4.0 SOC	CTCAE v4.0 Term	Grade 1	Grade 2	Grade 3	Grade 4	Grade 5	CTCAE v4.0 AE Term Definition
10028813	Gastrointestinal disorders	Nausea	Loss of appetite without alteration in eating habits	Oral intake decreased without significant weight loss, dehydration or malnutrition	Inadequate oral caloric or fluid intake; tube feeding, TPN, or hospitalization indicated	-	-	A disorder characterized by a queasy sensation and/or the urge to vomit.
10033371	General disorders and administration site conditions	Pain	Mild pain	Moderate pain; limiting instrumental ADL	Severe pain; limiting self care ADL	-	-	A disorder characterized by the sensation of marked discomfort, distress or agony.
10033557	Cardiac disorders	Palpitations	Mild symptoms; intervention not indicated	Intervention indicated	-	-	-	A disorder characterized by an unpleasant sensation of irregular and/or forceful beating of the heart.
10037087	Skin and subcutaneous tissue disorders	Pruritus	Mild or localized; topical intervention indicated	Intense or widespread; intermittent; skin changes from scratching (e.g., edema, papulation, excoriations, lichenification, oozing/crusts); oral intervention indicated; limiting instrumental ADL	Intense or widespread; constant; limiting self care ADL or sleep; oral corticosteroid or immunosuppressive therapy indicated	-	-	A disorder characterized by an intense itching sensation.

MedDRA v12.0 Code	CTCAE v4.0 SOC	CTCAE v4.0 Term	Grade 1	Grade 2	Grade 3	Grade 4	Grade 5	CTCAE v4.0 AE Term Definition
10037868	Skin and subcutaneous tissue disorders	Rash maculo-papular	Macules/papules covering <10% BSA with or without symptoms (e.g., pruritus, burning, tightness)	Macules/papules covering 10 - 30% BSA with or without symptoms (e.g., pruritus, burning, tightness); limiting instrumental ADL	Macules/papules covering >30% BSA with or without associated symptoms; limiting self care ADL	-	-	A disorder characterized by the presence of macules (flat) and papules (elevated). Also known as morbillform rash, it is one of the most common cutaneous adverse events, frequently affecting the upper trunk, spreading centripetally and associated with pruritis.

MedDRA v12.0 Code	CTCAE v4.0 SOC	CTCAE v4.0 Term	Grade 1	Grade 2	Grade 3	Grade 4	Grade 5	CTCAE v4.0 AE Term Definition
10040400	Immune system disorders	Serum sickness	Asymptomatic; clinical or diagnostic observations only; intervention not indicated	Moderate arthralgia; fever, rash, urticaria, antihistamines indicated	Severe arthralgia or arthritis; extensive rash; steroids or IV fluids indicated	Life-threatening consequences; pressor or ventilatory support indicated	Death	A disorder characterized by a delayed-type hypersensitivity reaction to foreign proteins derived from an animal serum. It occurs approximately six to twenty-one days following the administration of the foreign antigen. Symptoms include fever, arthralgias, myalgias, skin eruptions, lymphadenopathy, chest marked discomfort and dyspnea.
10051837	Skin and subcutaneous tissue disorders	Skin induration	Mild induration, able to move skin parallel to plane (sliding) and perpendicular to skin (pinching up)	Moderate induration, able to slide skin, unable to pinch skin; limiting instrumental ADL	Severe induration, unable to slide or pinch skin; limiting joint movement or orifice (e.g., mouth, anus); limiting self care ADL	Generalized; associated with signs or symptoms of impaired breathing or feeding	Death	A disorder characterized by an area of hardness in the skin.
10042241	Respiratory, thoracic and mediastinal disorders	Stridor	-	-	Respiratory distress limiting self care ADL; medical intervention indicated	Life-threatening airway compromise; urgent intervention indicated (e.g., tracheotomy or intubation)	Death	A disorder characterized by a high pitched breathing sound due to laryngeal or upper airway obstruction.

MedDRA v12.0 Code	CTCAE v4.0 SOC	CTCAE v4.0 Term	Grade 1	Grade 2	Grade 3	Grade 4	Grade 5	CTCAE v4.0 AE Term Definition
10046735	Skin and subcutaneous tissue disorders	Urticaria	Urticarial lesions covering <10% BSA; topical intervention indicated	Urticarial lesions covering 10 - 30% BSA; oral intervention indicated	Urticarial lesions covering >30% BSA; IV intervention indicated	-	-	A disorder characterized by an itchy skin eruption characterized by wheals with pale interiors and well-defined red margins.
10047700	Gastrointestinal disorders	Vomiting	1 - 2 episodes (separated by 5 minutes) in 24 hrs	3 - 5 episodes (separated by 5 minutes) in 24 hrs	>=6 episodes (separated by 5 minutes) in 24 hrs; tube feeding, TPN or hospitalization indicated	Life-threatening consequences; urgent intervention indicated	Death	A disorder characterized by the reflexive act of ejecting the contents of the stomach through the mouth.

APPENDIX G: DAIDS ADVERSE EVENT SEVERITY ASSESSMENT TABLE

Division of AIDS (DAIDS) Table for Grading the Severity of Adult and Pediatric Adverse Events

Version 2.0
November 2014

Division of AIDS
National Institute of Allergy and Infectious Diseases
National Institutes of Health
US Department of Health and Human Services

Table of Contents

Glossary and Acronyms 1
Introduction 3
Instructions for Use 4
Major Clinical Conditions 7
- Cardiovascular 7
- Dermatologic 9
- Endocrine and Metabolic 10
- Gastrointestinal 11
- Musculoskeletal 13
- Neurologic 14
- Pregnancy, Puerperium, and Perinatal 16
- Psychiatric 17
- Respiratory 18
- Sensory 19
- Systemic 20
- Urinary 22

Site Reactions to Injections and Infusions 23
Laboratory Values 24
- Chemistries 24
- Hematology 27
- Urinalysis 29

Appendix A. Total Bilirubin Table for Term and Preterm Neonates 30

Glossary and Acronyms

AE	Adverse event; Any unfavorable and unintended sign (including an abnormal laboratory finding), symptom, or disease temporally associated with the use of a medical treatment or procedure regardless of whether it is considered related to the medical treatment or procedure.
ALT (SGPT)	Alanine aminotransferase (serum glutamic pyruvic transaminase)
ANC	Absolute neutrophil count
AST (SGOT)	Aspartate aminotransferase (serum glutamic-oxaloacetic transaminase)
AV	Atrioventricular
Basic Self-care Functions	Adult<br>Activities such as bathing, dressing, toileting, transfer or movement, continence, and feeding.<br><br>Young Children<br>Activities that are age and culturally appropriate, such as feeding one's self with culturally appropriate eating implements.
BMI z-score	Body mass index z- score; A body reference norm. Specifically, the number of standard deviations a participant's BMI differs from the average BMI for their age, sex, and ethnicity.
BMD t-score	Bone mineral density t-score; The number of standard deviations above or below the mean bone mineral density of a healthy 30 year old adult of the same sex and ethnicity as the participant.
BMD z-score	Bone mineral density z-score; The number of standard deviations a participant's BMD differs from the average BMD for their age, sex, and ethnicity.
BPAP	Bilevel positive airway pressure; A mode used during noninvasive positive pressure ventilation.
Chemical Pregnancy	A pregnancy in which a positive pregnancy test is followed by a negative pregnancy test without evidence of a clinical pregnancy loss.
CNS	Central nervous system
CPAP	Continuous positive airway pressure
DAERS	DAIDS Adverse Experience Reporting System; An internet-based system developed for clinical research sites to report Expedited Adverse Events (EAEs) to DAIDS. It facilitates timely EAE report submission and serves as a centralized location for accessing and processing EAE information for reporting purposes.
Disability	A substantial disruption of a person's ability to conduct normal life functions.
ECG	Electrocardiogram
eGFR	Estimated glomerular filtration rate
Hospitalization	Does not include the following hospital admissions: under 24 hours, unrelated to an adverse event (e.g., for labor and delivery, cosmetic surgery, social or administrative for temporary placement [for lack of a place to sleep]), protocol-specified, and for diagnosis or therapy of a condition that existed before the receipt of a study agent and which has not increased in severity or frequency.
INR	International normalized ratio

GLOSSARY AND ACRONYMS

Intervention	Medical, surgical, or other procedures recommended or provided by a healthcare professional for the treatment of an adverse event.
IV	Intravenous
IVIG	Intravenous immune globulin
LDL	Low density lipoprotein
LLN	Lower limit of normal
Life-threatening AE	Any adverse event that places the participant, in the view of the investigator, at immediate risk of death from the reaction when it occurred (i.e., it does not include a reaction that would have caused death if it had occurred in a more severe form).
NA	Not applicable
Participant ID	The identification number assigned to a study participant which is used to track study-related documentation, including any reported AEs.
PR Interval	The interval between the beginning of the P wave and the beginning of the QRS complex of an electrocardiogram that represents the time between the beginning of the contraction of the atria and the beginning of the contraction of the ventricles.
PT	Prothrombin time
PTT	Partial thromboplastin time
QTc Interval	The measure of time between the onset of ventricular depolarization and completion of ventricular repolarization corrected for ventricular rate.
RBC	Red blood cell
SI	Standard international unit
ULN	Upper limit of normal
Usual Social & Functional Activities	Activities which adults and children perform on a routine basis and those which are part of regular activities of daily living, for example:<br><br><u>Adults</u><br>Adaptive tasks and desirable activities, such as going to work, shopping, cooking, use of transportation, or pursuing a hobby.<br><br><u>Young Children</u><br>Activities that are age and culturally appropriate, such as social interactions, play activities, or learning tasks.
WBC	White blood cell
WHO	World Health Organization
WNL	Within normal limits

INTRODUCTION

The Division of AIDS (DAIDS) oversees clinical trials throughout the world which it sponsors and supports. The clinical trials evaluate the safety and efficacy of therapeutic products, vaccines, and other preventive modalities. Adverse event (AE) data collected during these clinical trials form the basis for subsequent safety and efficacy analyses of pharmaceutical products and medical devices. Incorrect and inconsistent AE severity grading can lead to inaccurate data analyses and interpretation, which in turn can impact the safety and well-being of clinical trial participants and future patients using pharmaceutical products.

The DAIDS AE grading table is a shared tool for assessing the severity of AEs (including clinical and laboratory abnormalities) in participants enrolled in clinical trials. Over the years as scientific knowledge and experience have expanded, revisions to the DAIDS AE grading table have become necessary.

The *Division of AIDS (DAIDS) Table for Grading the Severity of Adult and Pediatric Adverse Events, Version 2.0* replaces the grading table published in 2004 and updated in 2009. In version 2.0, AEs not previously included, but which now are deemed medically important events, are included while other AEs have been removed. Some AE severity grading descriptions have been revised to more appropriately reflect the presentation of these events in clinical settings and their impact on clinical trials. For example, DAIDS performed an extensive literature search and reviews of select DAIDS clinical trial data in revising certain hematology parameters (i.e., hemoglobin, white cell counts, and absolute neutrophil counts). DAIDS also took into consideration the U.S. Food and Drug Administration's guidance regarding the use of local laboratory reference values and ethnic differences among certain healthy adolescent and adult populations in defining parameter limits. Finally, the revised DAIDS AE grading table also contains an updated glossary and acronyms section, an expanded instructions for use section, and an appendix that provides more age-specific information for an AE of concern to DAIDS.

DAIDS is grateful to the DAIDS Grading Table Working Group, numerous government and non-government affiliated medical subject matter experts and reviewers who were instrumental in the revision of the DAIDS AE grading table.

INSTRUCTIONS FOR USE

General Considerations
The *Division of AIDS* (*DAIDS) Table for Grading the Severity of Adult and Pediatric Adverse Events, Version 2.0* consists of parameters, or AEs, with severity grading guidance that are to be used in DAIDS clinical trials for safety data reporting to maintain accuracy and consistency in the evaluation of AEs. The term "severe" is not the same as the term "serious" in classifying AEs. The severity of a specific event describes its intensity, and it is the intensity which is graded. Seriousness, which is not graded, relates to an outcome of an AE and is a regulatory definition.

Clinical sites are encouraged to report parameters in the DAIDS AE grading table as they are written to maintain data consistency across clinical trials. However, since some parameters can be reported with more specificity, clinical sites are encouraged to report parameters that convey additional clinical information. For example, diarrhea could be reported as neonatal diarrhea; seizures, as febrile seizures; and pain, as jaw pain.

The DAIDS AE grading table provides an AE severity grading scale ranging from grades 1 to 5 with descriptions for each AE based on the following general guidelines:

- Grade 1 indicates a mild event
- Grade 2 indicates a moderate event
- Grade 3 indicates a severe event
- Grade 4 indicates a potentially life-threatening event
- Grade 5 indicates death (*Note*: This grade is not specifically listed on each page of the grading table).

Other points to consider include:

- Use parameters defined by age and sex values as applicable.
- Male and female sex are defined as sex at birth.
- Unless noted, laboratory values are for term neonates. Preterm neonates should be assessed using local laboratory normal ranges.
- Where applicable, Standard International (SI) units are included in italics.

Selecting and Reporting a Primary AE Term
When selecting a primary AE term to report, sites should select the term that best describes what occurred to the participant. For example, a participant may present with itching, urticaria, flushing, angioedema of the face, and dyspnea. If the underlying diagnosis is determined to be an acute allergic reaction, sites should report "Acute Allergic Reaction" as the primary AE term.

Primary AE terms should be reported using the DAIDS Adverse Experience Reporting System (DAERS) only if they meet expedited reporting criteria. However, all primary AE terms should be reported using protocol-specific case report forms (CRFs). Because the reported information is stored in different databases (i.e., safety and clinical), sites should report primary AE terms using the same terminology for data consistency.

Instructions for Use

When reporting using DAERS, other clinically significant events associated with a primary AE term that more fully describe the nature, severity, or complications of the primary AE term should be entered in the “Other Events” section. However, the severity grade for these events must be lower than or equal to the severity grade of the primary AE term. In the example above, dyspnea and angioedema of the face may be entered in the “Other Events” section, because they are more descriptive and provide additional information on the severity of the acute allergic reaction. However, their severity grades must be lower than or equal to the severity grade of the primary AE term of “Acute Allergic Reaction”.

Differences exist in the reporting and recording of information (e.g., signs and symptoms, clinically significant events) in DAERS and CRFs. Therefore, sites should refer to their protocols and CRF requirements for further instructions.

Grading Adult and Pediatric AEs
When a single parameter is not appropriate for grading an AE in both adult and pediatric populations, separate parameters with specified age ranges are provided. If no distinction between adult and pediatric populations has been made, the listed parameter should be used for grading an AE in both populations.

Reporting Pregnancy Outcomes
In the *Pregnancy, Puerperium, and Perinatal* section, all parameters are pregnancy outcomes and should be reported using the mother's participant ID. If an infant is not enrolled in the same study as the mother, any identified birth defects should be reported using the mother's participant ID. However, if an infant is enrolled in the same study as the mother or in another study, any identified birth defects should be reported using the infant's participant ID. Sites should refer to the applicable network standards for reporting abnormal pregnancy outcomes on the CRFs.

Determining Severity Grade for Parameters between Grades
If the severity of an AE could fall in either one of two grades (i.e., the severity of an AE could be either grade 2 or grade 3), sites should select the higher of the two grades.

Laboratory Values
General. An asymptomatic, abnormal laboratory finding without an accompanying AE should not be reported to DAIDS in an expedited timeframe unless it meets protocol-specific reporting requirements. Sites should refer to the applicable network standards for reporting abnormal laboratory findings on the CRFs.

Values below Grade 1. Any laboratory value that is between the ULN and grade 1 (for high values) or the LLN and grade 1 (for low values) should not be graded or reported as an AE. Sites should consult the *Manual for Expedited Reporting of Adverse Events to DAIDS, Version 2.0* and their protocol when making an assessment of the need to report an AE.

Overlap of Local Laboratory Normal Values with Grading Table Ranges. When local laboratory normal values fall within grading table laboratory ranges, the severity grading is based on the ranges in the grading table unless there is a protocol-specific grading criterion for the laboratory

INSTRUCTIONS FOR USE

value. For example, “Magnesium, Low" has a grade 1 range of 1.2 to < 1.4 mEq/L, while a particular laboratory’s normal range for magnesium may be 1.3 to 2.8 mEq/L. If a study participant’s magnesium laboratory value is 1.3 mEq/L, the laboratory value should be graded as grade 1.

Appendix Usage
Appendix A takes priority over the main grading table in all assessments of total bilirubin for term and preterm neonates.

Using Addenda 1-3: Grading Tables Used in Microbicide Studies
In protocols involving topical application of products to the female and male genital tracts or rectum, strong consideration should be given to using Addenda 1-3 (see below) as the primary grading tables for these areas. Although these grading tables are used specifically in microbicide studies, they may be used in other protocols as adjuncts to the main grading table (i.e., the *Division of AIDS (AIDS) Table for Grading the Severity of Adult and Pediatric Adverse Events, Version 2.0*). It should be clearly stated in a protocol which addendum is being used as the primary grading table (and thus takes precedence over the main grading table) and which addendum is being used in a complementary fashion.

- Addendum 1 – Female Genital Grading Table for Use in Microbicide Studies – PDF
- Addendum 2 – Male Genital Grading Table for Use in Microbicide Studies – PDF
- Addendum 3 – Rectal Grading Table for Use in Microbicide Studies – PDF

Estimating Severity Grade for Parameters Not Identified in the Grading Table
The functional table below should be used to grade the severity of an AE that is not specifically identified in the grading table. In addition, all deaths related to an AE are to be classified as grade 5.

PARAMETER	GRADE 1 MILD	GRADE 2 MODERATE	GRADE 3 SEVERE	GRADE 4 POTENTIALLY LIFE-THREATENING
**Clinical** adverse event **NOT** identified elsewhere in the grading table	Mild symptoms causing no or minimal interference with usual social & functional activities with intervention not indicated	Moderate symptoms causing greater than minimal interference with usual social & functional activities with intervention indicated	Severe symptoms causing inability to perform usual social & functional activities with intervention or hospitalization indicated	Potentially life-threatening symptoms causing inability to perform basic self-care functions with intervention indicated to prevent permanent impairment, persistent disability, or death

Major Clinical Conditions
Cardiovascular

PARAMETER	GRADE 1 MILD	GRADE 2 MODERATE	GRADE 3 SEVERE	GRADE 4 POTENTIALLY LIFE-THREATENING
**Arrhythmia** (by ECG or physical examination) *Specify type, if applicable*	No symptoms AND No intervention indicated	No symptoms AND Non-urgent intervention indicated	Non-life-threatening symptoms AND Non-urgent intervention indicated	Life-threatening arrhythmia OR Urgent intervention indicated
**Blood Pressure Abnormalities**[1] ***Hypertension*** *(with the lowest reading taken after repeat testing during a visit)* *≥ 18 years of age*	140 to < 160 mmHg systolic OR 90 to < 100 mmHg diastolic	≥ 160 to < 180 mmHg systolic OR ≥ 100 to < 110 mmHg diastolic	≥ 180 mmHg systolic OR ≥ 110 mmHg diastolic	Life-threatening consequences in a participant not previously diagnosed with hypertension (e.g., malignant hypertension) OR Hospitalization indicated
< 18 years of age	> 120/80 mmHg	≥ 95th to < 99th percentile + 5 mmHg adjusted for age, height, and gender (systolic and/or diastolic)	≥ 99th percentile + 5 mmHg adjusted for age, height, and gender (systolic and/or diastolic)	Life-threatening consequences in a participant not previously diagnosed with hypertension (e.g., malignant hypertension) OR Hospitalization indicated
Hypotension	No symptoms	Symptoms corrected with oral fluid replacement	Symptoms AND IV fluids indicated	Shock requiring use of vasopressors or mechanical assistance to maintain blood pressure
**Cardiac Ischemia or Infarction** *Report only one*	NA	NA	New symptoms with ischemia (stable angina) OR New testing consistent with ischemia	Unstable angina OR Acute myocardial infarction
**Heart Failure**	No symptoms AND Laboratory or cardiac imaging abnormalities	Symptoms with mild to moderate activity or exertion	Symptoms at rest or with minimal activity or exertion (e.g., hypoxemia) OR Intervention indicated (e.g., oxygen)	Life-threatening consequences OR Urgent intervention indicated (e.g., vasoactive medications, ventricular assist device, heart transplant)
**Hemorrhage** (with significant acute blood loss)	NA	Symptoms AND No transfusion indicated	Symptoms AND Transfusion of ≤ 2 units packed RBCs indicated	Life-threatening hypotension OR Transfusion of > 2 units packed RBCs (for children, packed RBCs > 10 cc/kg) indicated

[1] Blood pressure norms for children < 18 years of age can be found in: Expert Panel on Integrated Guidelines for Cardiovascular Health and Risk Reduction in Children and Adolescents. *Pediatrics* 2011;128;S213; originally published online November 14, 2011; DOI: 10.1542/peds.2009-2107C.

CARDIOVASCULAR

PARAMETER	GRADE 1 MILD	GRADE 2 MODERATE	GRADE 3 SEVERE	GRADE 4 POTENTIALLY LIFE-THREATENING
**Prolonged PR Interval or AV Block**<br>*Report only one*<br>*> 16 years of age*	PR interval 0.21 to < 0.25 seconds	PR interval ≥ 0.25 seconds OR Type I 2nd degree AV block	Type II 2nd degree AV block OR Ventricular pause ≥ 3.0 seconds	Complete AV block
≤ 16 years of age	1st degree AV block (PR interval > normal for age and rate)	Type I 2nd degree AV block	Type II 2nd degree AV block OR Ventricular pause ≥ 3.0 seconds	Complete AV block
**Prolonged QTc Interval**[2]	0.45 to 0.47 seconds	> 0.47 to 0.50 seconds	> 0.50 seconds OR ≥ 0.06 seconds above baseline	Life-threatening consequences (e.g., Torsade de pointes, other associated serious ventricular dysrhythmia)
**Thrombosis or Embolism**<br>*Report only one*	NA	Symptoms AND No intervention indicated	Symptoms AND Intervention indicated	Life-threatening embolic event (e.g., pulmonary embolism, thrombus)

[2] As per Bazett's formula.

Dermatologic

PARAMETER	GRADE 1 MILD	GRADE 2 MODERATE	GRADE 3 SEVERE	GRADE 4 POTENTIALLY LIFE-THREATENING
**Alopecia** (scalp only)	Detectable by study participant, caregiver, or physician AND Causing no or minimal interference with usual social & functional activities	Obvious on visual inspection AND Causing greater than minimal interference with usual social & functional activities	NA	NA
**Bruising**	Localized to one area	Localized to more than one area	Generalized	NA
**Cellulitis**	NA	Non-parenteral treatment indicated (e.g., oral antibiotics, antifungals, antivirals)	IV treatment indicated (e.g., IV antibiotics, antifungals, antivirals)	Life-threatening consequences (e.g., sepsis, tissue necrosis)
**Hyperpigmentation**	Slight or localized causing no or minimal interference with usual social & functional activities	Marked or generalized causing greater than minimal interference with usual social & functional activities	NA	NA
**Hypopigmentation**	Slight or localized causing no or minimal interference with usual social & functional activities	Marked or generalized causing greater than minimal interference with usual social & functional activities	NA	NA
**Petechiae**	Localized to one area	Localized to more than one area	Generalized	NA
**Pruritus**[3] (without skin lesions)	Itching causing no or minimal interference with usual social & functional activities	Itching causing greater than minimal interference with usual social & functional activities	Itching causing inability to perform usual social & functional activities	NA
**Rash** *Specify type, if applicable*	Localized rash	Diffuse rash OR Target lesions	Diffuse rash AND Vesicles or limited number of bullae or superficial ulcerations of mucous membrane limited to one site	Extensive or generalized bullous lesions OR Ulceration of mucous membrane involving two or more distinct mucosal sites OR Stevens-Johnson syndrome OR Toxic epidermal necrolysis

[3] For pruritus associated with injections or infusions, see the *Site Reactions to Injections and Infusions* section (page 23).

ENDOCRINE AND METABOLIC

PARAMETER	GRADE 1 MILD	GRADE 2 MODERATE	GRADE 3 SEVERE	GRADE 4 POTENTIALLY LIFE-THREATENING
**Diabetes Mellitus**	Controlled without medication	Controlled with medication OR Modification of current medication regimen	Uncontrolled despite treatment modification OR Hospitalization for immediate glucose control indicated	Life-threatening consequences (e.g., ketoacidosis, hyperosmolar non-ketotic coma, end organ failure)
**Gynecomastia**	Detectable by study participant, caregiver, or physician AND Causing no or minimal interference with usual social & functional activities	Obvious on visual inspection AND Causing pain with greater than minimal interference with usual social & functional activities	Disfiguring changes AND Symptoms requiring intervention or causing inability to perform usual social & functional activities	NA
**Hyperthyroidism**	No symptoms AND Abnormal laboratory value	Symptoms causing greater than minimal interference with usual social & functional activities OR Thyroid suppression therapy indicated	Symptoms causing inability to perform usual social & functional activities OR Uncontrolled despite treatment modification	Life-threatening consequences (e.g., thyroid storm)
**Hypothyroidism**	No symptoms AND Abnormal laboratory value	Symptoms causing greater than minimal interference with usual social & functional activities OR Thyroid replacement therapy indicated	Symptoms causing inability to perform usual social & functional activities OR Uncontrolled despite treatment modification	Life-threatening consequences (e.g., myxedema coma)
**Lipoatrophy**[4]	Detectable by study participant, caregiver, or physician AND Causing no or minimal interference with usual social & functional activities	Obvious on visual inspection AND Causing greater than minimal interference with usual social & functional activities	Disfiguring changes	NA
**Lipohypertrophy**[5]	Detectable by study participant, caregiver, or physician AND Causing no or minimal interference with usual social & functional activities	Obvious on visual inspection AND Causing greater than minimal interference with usual social & functional activities	Disfiguring changes	NA

[4] Definition: A disorder characterized by fat loss in the face, extremities, and buttocks.
[5] Definition: A disorder characterized by abnormal fat accumulation on the back of the neck, breasts, and abdomen.

GASTROINTESTINAL

PARAMETER	GRADE 1 MILD	GRADE 2 MODERATE	GRADE 3 SEVERE	GRADE 4 POTENTIALLY LIFE-THREATENING
**Anorexia**	Loss of appetite without decreased oral intake	Loss of appetite associated with decreased oral intake without significant weight loss	Loss of appetite associated with significant weight loss	Life-threatening consequences OR Aggressive intervention indicated (e.g., tube feeding, total parenteral nutrition)
**Ascites**	No symptoms	Symptoms AND Intervention indicated (e.g., diuretics, therapeutic paracentesis)	Symptoms recur or persist despite intervention	Life-threatening consequences
**Bloating or Distension**<br>*Report only one*	Symptoms causing no or minimal interference with usual social & functional activities	Symptoms causing greater than minimal interference with usual social & functional activities	Symptoms causing inability to perform usual social & functional activities	NA
**Cholecystitis**	NA	Symptoms AND Medical intervention indicated	Radiologic, endoscopic, or operative intervention indicated	Life-threatening consequences (e.g., sepsis, perforation)
**Constipation**	NA	Persistent constipation requiring regular use of dietary modifications, laxatives, or enemas	Obstipation with manual evacuation indicated	Life-threatening consequences (e.g., obstruction)
**Diarrhea**<br>*≥ 1 year of age*	Transient or intermittent episodes of unformed stools OR Increase of ≤ 3 stools over baseline per 24-hour period	Persistent episodes of unformed to watery stools OR Increase of 4 to 6 stools over baseline per 24-hour period	Increase of ≥ 7 stools per 24-hour period OR IV fluid replacement indicated	Life-threatening consequences (e.g., hypotensive shock)
< 1 year of age	Liquid stools (more unformed than usual) but usual number of stools	Liquid stools with increased number of stools OR Mild dehydration	Liquid stools with moderate dehydration	Life-threatening consequences (e.g., liquid stools resulting in severe dehydration, hypotensive shock)
**Dysphagia or Odynophagia**<br>*Report only one and specify location*	Symptoms but able to eat usual diet	Symptoms causing altered dietary intake with no intervention indicated	Symptoms causing severely altered dietary intake with intervention indicated	Life-threatening reduction in oral intake
**Gastrointestinal Bleeding**	Not requiring intervention other than iron supplement	Endoscopic intervention indicated	Transfusion indicated	Life-threatening consequences (e.g., hypotensive shock)

GASTROINTESTINAL

PARAMETER	GRADE 1 MILD	GRADE 2 MODERATE	GRADE 3 SEVERE	GRADE 4 POTENTIALLY LIFE-THREATENING
**Mucositis or Stomatitis** *Report only one and specify location*	Mucosal erythema	Patchy pseudomembranes or ulcerations	Confluent pseudomembranes or ulcerations OR Mucosal bleeding with minor trauma	Life-threatening consequences (e.g., aspiration, choking) OR Tissue necrosis OR Diffuse spontaneous mucosal bleeding
**Nausea**	Transient (< 24 hours) or intermittent AND No or minimal interference with oral intake	Persistent nausea resulting in decreased oral intake for 24 to 48 hours	Persistent nausea resulting in minimal oral intake for > 48 hours OR Rehydration indicated (e.g., IV fluids)	Life-threatening consequences (e.g., hypotensive shock)
**Pancreatitis**	NA	Symptoms with hospitalization not indicated	Symptoms with hospitalization indicated	Life-threatening consequences (e.g., circulatory failure, hemorrhage, sepsis)
**Perforation** (colon or rectum)	NA	NA	Intervention indicated	Life-threatening consequences
**Proctitis**	Rectal discomfort with no intervention indicated	Symptoms causing greater than minimal interference with usual social & functional activities OR Medical intervention indicated	Symptoms causing inability to perform usual social & functional activities OR Operative intervention indicated	Life-threatening consequences (e.g., perforation)
**Rectal Discharge**	Visible discharge	Discharge requiring the use of pads	NA	NA
**Vomiting**	Transient or intermittent AND No or minimal interference with oral intake	Frequent episodes with no or mild dehydration	Persistent vomiting resulting in orthostatic hypotension OR Aggressive rehydration indicated (e.g., IV fluids)	Life-threatening consequences (e.g., hypotensive shock)

MUSCULOSKELETAL

PARAMETER	GRADE 1 MILD	GRADE 2 MODERATE	GRADE 3 SEVERE	GRADE 4 POTENTIALLY LIFE-THREATENING
**Arthralgia**	Joint pain causing no or minimal interference with usual social & functional activities	Joint pain causing greater than minimal interference with usual social & functional activities	Joint pain causing inability to perform usual social & functional activities	Disabling joint pain causing inability to perform basic self-care functions
**Arthritis**	Stiffness or joint swelling causing no or minimal interference with usual social & functional activities	Stiffness or joint swelling causing greater than minimal interference with usual social & functional activities	Stiffness or joint swelling causing inability to perform usual social & functional activities	Disabling joint stiffness or swelling causing inability to perform basic self-care functions
**Myalgia** (generalized)	Muscle pain causing no or minimal interference with usual social & functional activities	Muscle pain causing greater than minimal interference with usual social & functional activities	Muscle pain causing inability to perform usual social & functional activities	Disabling muscle pain causing inability to perform basic self-care functions
**Osteonecrosis**	NA	No symptoms but with radiographic findings AND No operative intervention indicated	Bone pain with radiographic findings OR Operative intervention indicated	Disabling bone pain with radiographic findings causing inability to perform basic self-care functions
**Osteopenia**[6] *≥ 30 years of age*	BMD t-score -2.5 to -1	NA	NA	NA
< 30 years of age	BMD z-score -2 to -1	NA	NA	NA
**Osteoporosis**[6] *≥ 30 years of age*	NA	BMD t-score < -2.5	Pathologic fracture (e.g., compression fracture causing loss of vertebral height)	Pathologic fracture causing life-threatening consequences
< 30 years of age	NA	BMD z-score < -2	Pathologic fracture (e.g., compression fracture causing loss of vertebral height)	Pathologic fracture causing life-threatening consequences

[6] BMD t and z scores can be found in: Kanis JA on behalf of the World Health Organization Scientific Group (2007). Assessment of osteoporosis at the primary health-care level. Technical Report. World Health Organization Collaborating Centre for Metabolic Bone Diseases, University of Sheffield, UK. 2007: Printed by the University of Sheffield.

NEUROLOGIC

PARAMETER	GRADE 1 MILD	GRADE 2 MODERATE	GRADE 3 SEVERE	GRADE 4 POTENTIALLY LIFE-THREATENING
**Acute CNS Ischemia**	NA	NA	Transient ischemic attack	Cerebral vascular accident (e.g., stroke with neurological deficit)
**Altered Mental Status** (for Dementia, see *Cognitive, Behavioral, or Attentional Disturbance* below)	Changes causing no or minimal interference with usual social & functional activities	Mild lethargy or somnolence causing greater than minimal interference with usual social & functional activities	Confusion, memory impairment, lethargy, or somnolence causing inability to perform usual social & functional activities	Delirium OR Obtundation OR Coma
**Ataxia**	Symptoms causing no or minimal interference with usual social & functional activities OR No symptoms with ataxia detected on examination	Symptoms causing greater than minimal interference with usual social & functional activities	Symptoms causing inability to perform usual social & functional activities	Disabling symptoms causing inability to perform basic self-care functions
**Cognitive, Behavioral, or Attentional Disturbance** (includes dementia and attention deficit disorder)<br>*Specify type, if applicable*	Disability causing no or minimal interference with usual social & functional activities OR Specialized resources not indicated	Disability causing greater than minimal interference with usual social & functional activities OR Specialized resources on part-time basis indicated	Disability causing inability to perform usual social & functional activities OR Specialized resources on a full-time basis indicated	Disability causing inability to perform basic self-care functions OR Institutionalization indicated
**Developmental Delay** *< 18 years of age*<br>*Specify type, if applicable*	Mild developmental delay, either motor or cognitive, as determined by comparison with a developmental screening tool appropriate for the setting	Moderate developmental delay, either motor or cognitive, as determined by comparison with a developmental screening tool appropriate for the setting	Severe developmental delay, either motor or cognitive, as determined by comparison with a developmental screening tool appropriate for the setting	Developmental regression, either motor or cognitive, as determined by comparison with a developmental screening tool appropriate for the setting
**Headache**	Symptoms causing no or minimal interference with usual social & functional activities	Symptoms causing greater than minimal interference with usual social & functional activities	Symptoms causing inability to perform usual social & functional activities	Symptoms causing inability to perform basic self-care functions OR Hospitalization indicated OR Headache with significant impairment of alertness or other neurologic function

NEUROLOGIC

PARAMETER	GRADE 1 MILD	GRADE 2 MODERATE	GRADE 3 SEVERE	GRADE 4 POTENTIALLY LIFE-THREATENING
**Neuromuscular Weakness** (includes myopathy and neuropathy)<br>*Specify type, if applicable*	Minimal muscle weakness causing no or minimal interference with usual social & functional activities OR No symptoms with decreased strength on examination	Muscle weakness causing greater than minimal interference with usual social & functional activities	Muscle weakness causing inability to perform usual social & functional activities	Disabling muscle weakness causing inability to perform basic self-care functions OR Respiratory muscle weakness impairing ventilation
**Neurosensory Alteration** (includes paresthesia and painful neuropathy)<br>*Specify type, if applicable*	Minimal paresthesia causing no or minimal interference with usual social & functional activities OR No symptoms with sensory alteration on examination	Sensory alteration or paresthesia causing greater than minimal interference with usual social & functional activities	Sensory alteration or paresthesia causing inability to perform usual social & functional activities	Disabling sensory alteration or paresthesia causing inability to perform basic self-care functions
**Seizures**<br>***New Onset Seizure***<br>*≥ 18 years of age*	NA	NA	1 to 3 seizures	Prolonged and repetitive seizures (e.g., status epilepticus) OR Difficult to control (e.g., refractory epilepsy)
< 18 years of age (includes new or pre-existing febrile seizures)	Seizure lasting < 5 minutes with < 24 hours postictal state	Seizure lasting 5 to < 20 minutes with < 24 hours postictal state	Seizure lasting ≥ 20 minutes OR > 24 hours postictal state	Prolonged and repetitive seizures (e.g., status epilepticus) OR Difficult to control (e.g., refractory epilepsy)
Pre-existing Seizure	NA	Increased frequency from previous level of control without change in seizure character	Change in seizure character either in duration or quality (e.g., severity or focality)	Prolonged and repetitive seizures (e.g., status epilepticus) OR Difficult to control (e.g., refractory epilepsy)
**Syncope**	Near syncope without loss of consciousness (e.g., pre-syncope)	Loss of consciousness with no intervention indicated	Loss of consciousness AND Hospitalization or intervention required	NA

PREGNANCY, PUERPERIUM, AND PERINATAL

PARAMETER	GRADE 1<br>MILD	GRADE 2<br>MODERATE	GRADE 3<br>SEVERE	GRADE 4<br>POTENTIALLY LIFE-THREATENING
**Fetal Death or Stillbirth** (report using mother's participant ID)<br>*Report only one*	NA	NA	Fetal loss occurring at ≥ 20 weeks gestation	NA
**Preterm Delivery**[7] (report using mother's participant [illegible]	Delivery at 34 to < 37 weeks gestational age	Delivery at 28 to < 34 weeks gestational age	Delivery at 24 to < 28 weeks gestational age	Delivery at < 24 weeks gestational age
**Spontaneous Abortion or Miscarriage**[8] (report using mother's participant ID)<br>*Report only one*	Chemical pregnancy	Uncomplicated spontaneous abortion or miscarriage	Complicated spontaneous abortion or miscarriage	NA

[7] Definition: A delivery of a live-born neonate occurring at ≥ 20 to < 37 weeks gestational age.
[8] Definition: A clinically recognized pregnancy occurring at < 20 weeks gestational age.

PSYCHIATRIC

PARAMETER	GRADE 1 MILD	GRADE 2 MODERATE	GRADE 3 SEVERE	GRADE 4 POTENTIALLY LIFE-THREATENING
**Insomnia**	Mild difficulty falling asleep, staying asleep, or waking up early	Moderate difficulty falling asleep, staying asleep, or waking up early	Severe difficulty falling asleep, staying asleep, or waking up early	NA
**Psychiatric Disorders** (includes anxiety, depression, mania, and psychosis)<br>*Specify disorder*	Symptoms with intervention not indicated OR Behavior causing no or minimal interference with usual social & functional activities	Symptoms with intervention indicated OR Behavior causing greater than minimal interference with usual social & functional activities	Symptoms with hospitalization indicated OR Behavior causing inability to perform usual social & functional activities	Threatens harm to self or others OR Acute psychosis OR Behavior causing inability to perform basic self-care functions
**Suicidal Ideation or Attempt**<br>*Report only one*	Preoccupied with thoughts of death AND No wish to kill oneself	Preoccupied with thoughts of death AND Wish to kill oneself with no specific plan or intent	Thoughts of killing oneself with partial or complete plans but no attempt to do so OR Hospitalization indicated	Suicide attempted

RESPIRATORY

PARAMETER	GRADE 1 MILD	GRADE 2 MODERATE	GRADE 3 SEVERE	GRADE 4 POTENTIALLY LIFE-THREATENING
**Acute Bronchospasm**	Forced expiratory volume in 1 second or peak flow reduced to ≥ 70 to < 80% OR Mild symptoms with intervention not indicated	Forced expiratory volume in 1 second or peak flow 50 to < 70% OR Symptoms with intervention indicated OR Symptoms causing greater than minimal interference with usual social & functional activities	Forced expiratory volume in 1 second or peak flow 25 to < 50% OR Symptoms causing inability to perform usual social & functional activities	Forced expiratory volume in 1 second or peak flow < 25% OR Life-threatening respiratory or Hemodynamic compromise OR Intubation
**Dyspnea or Respiratory Distress** *Report only one*	Dyspnea on exertion with no or minimal interference with usual social & functional activities OR Wheezing OR Minimal increase in respiratory rate for age	Dyspnea on exertion causing greater than minimal interference with usual social & functional activities OR Nasal flaring OR Intercostal retractions OR Pulse oximetry 90 to < 95%	Dyspnea at rest causing inability to perform usual social & functional activities OR Pulse oximetry < 90%	Respiratory failure with ventilator support indicated (e.g., CPAP, BPAP, intubation)

SENSORY

PARAMETER	GRADE 1 MILD	GRADE 2 MODERATE	GRADE 3 SEVERE	GRADE 4 POTENTIALLY LIFE-THREATENING
**Hearing Loss**<br>*≥ 12 years of age*	NA	Hearing aid or intervention not indicated	Hearing aid or intervention indicated	Profound bilateral hearing loss (> 80 dB at 2 kHz and above) OR Non-serviceable hearing (i.e., >50 dB audiogram and <50% speech discrimination)
< 12 years of age (based on a 1, 2, 3, 4, 6 and 8 kHz audiogram)	> 20 dB hearing loss at ≤ 4 kHz	> 20 dB hearing loss at > 4 kHz	> 20 dB hearing loss at ≥ 3 kHz in one ear with additional speech language related services indicated (where available) OR Hearing loss sufficient to indicate therapeutic intervention, including hearing aids	Audiologic indication for cochlear implant and additional speech-language related services indicated (where available)
**Tinnitus**	Symptoms causing no or minimal interference with usual social & functional activities with intervention not indicated	Symptoms causing greater than minimal interference with usual social & functional activities with intervention indicated	Symptoms causing inability to perform usual social & functional activities	NA
**Uveitis**	No symptoms AND Detectable on examination	Anterior uveitis with symptoms OR Medicamylasal intervention indicated	Posterior or pan-uveitis OR Operative intervention indicated	Disabling visual loss in affected eye(s)
**Vertigo**	Vertigo causing no or minimal interference with usual social & functional activities	Vertigo causing greater than minimal interference with usual social & functional activities	Vertigo causing inability to perform usual social & functional activities	Disabling vertigo causing inability to perform basic self-care functions
**Visual Changes** (assessed from baseline)	Visual changes causing no or minimal interference with usual social & functional activities	Visual changes causing greater than minimal interference with usual social & functional activities	Visual changes causing inability to perform usual social & functional activities	Disabling visual loss in affected eye(s)

SYSTEMIC

PARAMETER	GRADE 1 MILD	GRADE 2 MODERATE	GRADE 3 SEVERE	GRADE 4 POTENTIALLY LIFE-THREATENING
**Acute Allergic Reaction**	Localized urticaria (wheals) with no medical intervention indicated	Localized urticaria with intervention indicated OR Mild angioedema with no intervention indicated	Generalized urticaria OR Angioedema with intervention indicated OR Symptoms of mild bronchospasm	Acute anaphylaxis OR Life-threatening bronchospasm OR Laryngeal edema
**Chills**	Symptoms causing no or minimal interference with usual social & functional activities	Symptoms causing greater than minimal interference with usual social & functional activities	Symptoms causing inability to perform usual social & functional activities	NA
**Cytokine Release Syndrome**[9]	Mild signs and symptoms AND Therapy (i.e., antibody infusion) interruption not indicated	Therapy (i.e., antibody infusion) interruption indicated AND Responds promptly to symptomatic treatment OR Prophylactic medications indicated for ≤ 24 hours	Prolonged severe signs and symptoms OR Recurrence of symptoms following initial improvement	Life-threatening consequences (e.g., requiring pressor or ventilator support)
**Fatigue or Malaise** *Report only one*	Symptoms causing no or minimal interference with usual social & functional activities	Symptoms causing greater than minimal interference with usual social & functional activities	Symptoms causing inability to perform usual social & functional activities	Incapacitating symptoms of fatigue or malaise causing inability to perform basic self-care functions
**Fever** (non-axillary temperatures only)	38.0 to < 38.6°C or 100.4 to < 101.5°F	≥ 38.6 to < 39.3°C or ≥101.5 to < 102.7°F	≥ 39.3 to < 40.0°C or ≥ 102.7 to < 104.0°F	≥ 40.0°C or ≥ 104.0°F
**Pain**[10] (not associated with study agent injections and not specified elsewhere) *Specify location*	Pain causing no or minimal interference with usual social & functional activities	Pain causing greater than minimal interference with usual social & functional activities	Pain causing inability to perform usual social & functional activities	Disabling pain causing inability to perform basic self-care functions OR Hospitalization indicated
**Serum Sickness**[11]	Mild signs and symptoms	Moderate signs and symptoms AND Intervention indicated (e.g., antihistamines)	Severe signs and symptoms AND Higher level intervention indicated (e.g., steroids or IV fluids)	Life-threatening consequences (e.g., requiring pressor or ventilator support)

[9] Definition: A disorder characterized by nausea, headache, tachycardia, hypotension, rash, and/or shortness of breath.
[10] For pain associated with injections or infusions, see the *Site Reactions to Injections and Infusions* section (page 23).
[11] Definition: A disorder characterized by fever, arthralgia, myalgia, skin eruptions, lymphadenopathy, marked discomfort, and/or dyspnea.

SYSTEMIC

PARAMETER	GRADE 1 MILD	GRADE 2 MODERATE	GRADE 3 SEVERE	GRADE 4 POTENTIALLY LIFE-THREATENING
**Underweight**[12] *> 5 to 19 years of age*	NA	WHO BMI z-score < -2 to ≤ -3	WHO BMI z-score < -3	WHO BMI z-score < -3 with life-threatening consequences
2 to 5 years of age	NA	WHO Weight-for-height z-score < -2 to ≤-3	WHO Weight-for-height z-score < -3	WHO Weight-for-height z-score < -3 with life-threatening consequences
< 2 years of age	NA	WHO Weight-for-length z-score < -2 to ≤-3	WHO Weight-for-length z-score < -3	WHO Weight-for-length z-score < -3 with life-threatening consequences
**Weight Loss** (excludes postpartum weight loss)	NA	5 to < 9% loss in body weight from baseline	≥ 9 to < 20% loss in body weight from baseline	≥ 20% loss in body weight from baseline OR Aggressive intervention indicated (e.g., tube feeding, total parenteral nutrition)

[12] WHO reference tables may be accesed by clicking the desired age range or by accessing the following URLs: http://www.who.int/growthref/who2007 bmi for age/en/ for participants > 5 to 19 years of age and http://www.who.int/childgrowth/standards/chart catalogue/en/ for those < 5 years of age.

URINARY

PARAMETER	GRADE 1 MILD	GRADE 2 MODERATE	GRADE 3 SEVERE	GRADE 4 POTENTIALLY LIFE-THREATENING
**Urinary Tract Obstruction**	NA	Signs or symptoms of urinary tract obstruction without hydronephrosis or renal dysfunction	Signs or symptoms of urinary tract obstruction with hydronephrosis or renal dysfunction	Obstruction causing life-threatening consequences

SITE REACTIONS TO INJECTIONS AND INFUSIONS

PARAMETER	GRADE 1<br>MILD	GRADE 2<br>MODERATE	GRADE 3<br>SEVERE	GRADE 4<br>POTENTIALLY LIFE-THREATENING
**Injection Site Pain or Tenderness**<br>*Report only one*	Pain or tenderness causing no or minimal limitation of use of limb	Pain or tenderness causing greater than minimal limitation of use of limb	Pain or tenderness causing inability to perform usual social & functional activities	Pain or tenderness causing inability to perform basic self-care function OR Hospitalization indicated
**Injection Site Erythema or Redness**[13]<br>*Report only one*<br>*> 15 years of age*	2.5 to < 5 cm in diameter OR 6.25 to < 25 cm^2 surface area AND<br>Symptoms causing no or minimal interference with usual social & functional activities	≥ 5 to < 10 cm in diameter OR ≥ 25 to < 100 cm^2 surface area OR Symptoms causing greater than minimal interference with usual social & functional activities	≥ 10 cm in diameter OR ≥ 100 cm^2 surface area OR Ulceration OR Secondary infection OR Phlebitis OR Sterile abscess OR Drainage OR<br>Symptoms causing inability to perform usual social & functional activities	Potentially life-threatening consequences (e.g., abscess, exfoliative dermatitis, necrosis involving dermis or deeper tissue)
≤15 years of age	≤ 2.5 cm in diameter	> 2.5 cm in diameter with < 50% surface area of the extremity segment involved (e.g., upper arm or thigh)	≥ 50% surface area of the extremity segment involved (e.g., upper arm or thigh) OR Ulceration OR Secondary infection OR Phlebitis OR Sterile abscess OR Drainage	Potentially life-threatening consequences (e.g., abscess, exfoliative dermatitis, necrosis involving dermis or deeper tissue)
**Injection Site Induration or Swelling**<br>*Report only one*<br>*> 15 years of age*	Same as for **Injection Site Erythema or Redness,** *> 15 years of age*	Same as for **Injection Site Erythema or Redness,** *> 15 years of age*	Same as for **Injection Site Erythema or Redness,** *> 15 years of age*	Same as for **Injection Site Erythema or Redness,** *> 15 years of age*
≤ 15 years of age	Same as for **Injection Site Erythema or Redness,** *≤ 15 years of age*	Same as for **Injection Site Erythema or Redness,** *≤ 15 years of age*	Same as for **Injection Site Erythema or Redness,** *≤ 15 years of age*	Same as for **Injection Site Erythema or Redness,** *≤ 15 years of age*
**Injection Site Pruritus**	Itching localized to the injection site that is relieved spontaneously or in < 48 hours of treatment	Itching beyond the injection site that is not generalized OR<br>Itching localized to the injection site requiring ≥ 48 hours treatment	Generalized itching causing inability to perform usual social & functional activities	NA

[13] Injection Site Erythema or Redness should be evaluated and graded using the greatest single diameter or measured surface area.

Laboratory Values
Chemistries

PARAMETER	GRADE 1 MILD	GRADE 2 MODERATE	GRADE 3 SEVERE	GRADE 4 POTENTIALLY LIFE-THREATENING
**Acidosis**	NA	pH ≥ 7.3 to < LLN	pH < 7.3 without life-threatening consequences	pH < 7.3 with life-threatening consequences
**Albumin, Low** (g/dL; *g/L*)	3.0 to < LLN<br>*30 to < LLN*	≥ 2.0 to < 3.0<br>*≥ 20 to < 30*	< 2.0<br>*< 20*	NA
**Alkaline Phosphatase, High**	1.25 to < 2.5 x ULN	2.5 to < 5.0 x ULN	5.0 to < 10.0 x ULN	≥ 10.0 x ULN
**Alkalosis**	NA	pH > ULN to ≤ 7.5	pH > 7.5 without life-threatening consequences	pH > 7.5 with life-threatening consequences
**ALT or SGPT, High**<br>*Report only one*	1.25 to < 2.5 x ULN	2.5 to < 5.0 x ULN	5.0 to < 10.0 x ULN	≥ 10.0 x ULN
**Amylase (Pancreatic) or Amylase (Total), High**<br>*Report only one*	1.1 to < 1.5 x ULN	1.5 to < 3.0 x ULN	3.0 to < 5.0 x ULN	≥ 5.0 x ULN
**AST or SGOT, High**<br>*Report only one*	1.25 to < 2.5 x ULN	2.5 to < 5.0 x ULN	5.0 to < 10.0 x ULN	≥ 10.0 x ULN
**Bicarbonate, Low** (mEq/L; *mmol/L*)	16.0 to < LLN<br>*16.0 to < LLN*	11.0 to < 16.0<br>*11.0 to < 16.0*	8.0 to < 11.0<br>*8.0 to < 11.0*	< 8.0<br>*< 8.0*
**Bilirubin**<br>***Direct Bilirubin[14], High***<br>*> 28 days of age*	NA	NA	> ULN	> ULN with life-threatening consequences (e.g., signs and symptoms of liver failure)
≤ 28 days of age	ULN to ≤ 1 mg/dL	> 1 to ≤ 1.5 mg/dL	> 1.5 to ≤ 2 mg/dL	> 2 mg/dL
Total Bilirubin, High<br>*> 28 days of age*	1.1 to < 1.6 x ULN	1.6 to < 2.6 x ULN	2.6 to < 5.0 x ULN	≥ 5.0 x ULN
≤ 28 days of age	See Appendix A. Total Bilirubin for Term and Preterm Neonates	See Appendix A. Total Bilirubin for Term and Preterm Neonates	See Appendix A. Total Bilirubin for Term and Preterm Neonates	See Appendix A. Total Bilirubin for Term and Preterm Neonates
**Calcium, High** (mg/dL; *mmol/L*)<br>*≥ 7 days of age*	10.6 to < 11.5<br>*2.65* to *< 2.88*	11.5 to < 12.5<br>*2.88* to *< 3.13*	12.5 to < 13.5<br>*3.13* to *< 3.38*	≥ 13.5<br>*≥ 3.38*
< 7 days of age	11.5 to < 12.4<br>*2.88* to *< 3.10*	12.4 to < 12.9<br>*3.10* to *< 3.23*	12.9 to < 13.5<br>*3.23* to *< 3.38*	≥ 13.5<br>*≥ 3.38*

[14] Direct bilirubin > 1.5 mg/dL in a participant < 28 days of age should be graded as grade 2, if < 10% of the total bilirubin.

CHEMISTRIES

PARAMETER	GRADE 1 MILD	GRADE 2 MODERATE	GRADE 3 SEVERE	GRADE 4 POTENTIALLY LIFE-THREATENING
**Calcium (Ionized), High** (mg/dL; *mmol/L*)	> ULN to < 6.0<br>*> ULN to < 1.5*	6.0 to < 6.4<br>*1.5 to < 1.6*	6.4 to < 7.2<br>*1.6 to < 1.8*	≥ 7.2<br>*≥ 1.8*
**Calcium, Low** (mg/dL; *mmol/L*)<br>*≥ 7 days of age*	7.8 to < 8.4<br>*1.95* to *< 2.10*	7.0 to < 7.8<br>*1.75* to *< 1.95*	6.1 to < 7.0<br>*1.53* to *< 1.75*	< 6.1<br>*< 1.53*
< 7 days of age	6.5 to < 7.5<br>*1.63* to *< 1.88*	6.0 to < 6.5<br>*1.50* to *< 1.63*	5.50 to < 6.0<br>*1.38* to *< 1.50*	< 5.50<br>*< 1.38*
**Calcium (Ionized), Low** (mg/dL; *mmol/L*)	< LLN to 4.0<br>*< LLN to 1.0*	3.6 to < 4.0<br>*0.9 to < 1.0*	3.2 to < 3.6<br>*0.8 to < 0.9*	< 3.2<br>*< 0.8*
**Cardiac Troponin I, High**	NA	NA	NA	Levels consistent with myocardial infarction or unstable angina as defined by the local laboratory
**Creatine Kinase, High**	3 to < 6 x ULN	6 to < 10 x ULN	10 to < 20 x ULN	≥ 20 x ULN
**Creatinine, High**	1.1 to 1.3 x ULN	> 1.3 to 1.8 x ULN OR Increase of > 0.3 mg/dL above baseline	> 1.8 to < 3.5 x ULN OR Increase of 1.5 to < 2.0 x above baseline	≥ 3.5 x ULN OR Increase of ≥ 2.0 x above baseline
**Creatinine Clearance[15] or eGFR, Low**<br>*Report only one*	NA	< 90 to 60 ml/min or ml/min/1.73 m^2 OR 10 to < 30% decrease from baseline	< 60 to 30 ml/min or ml/min/1.73 m^2 OR ≥ 30 to < 50% decrease from baseline	< 30 ml/min or ml/min/1.73 m2 OR ≥ 50% decrease from baseline or dialysis needed
**Glucose** (mg/dL; *mmol/L*)<br>***Fasting, High***	110 to 125<br>*6.11 to < 6.95*	> 125 to 250<br>*6.95 to < 13.89*	> 250 to 500<br>*13.89 to < 27.75*	> 500<br>*≥ 27.75*
Nonfasting, High	116 to 160<br>*6.44 to < 8.89*	> 160 to 250<br>*8.89 to < 13.89*	> 250 to 500<br>*13.89 to < 27.75*	> 500<br>*≥ 27.75*
**Glucose, Low** (mg/dL; *mmol/L*)<br>*≥ 1 month of age*	55 to 64<br>*3.05 to 3.55*	40 to < 55<br>*2.22 to < 3.05*	30 to < 40<br>*1.67 to < 2.22*	< 30<br>*< 1.67*
< 1 month of age	50 to 54<br>*2.78 to 3.00*	40 to < 50<br>*2.22 to < 2.78*	30 to < 40<br>*1.67 to < 2.22*	< 30<br>*< 1.67*
**Lactate, High**	ULN to < 2.0 x ULN without acidosis	≥ 2.0 x ULN without acidosis	Increased lactate with pH < 7.3 without life-threatening consequences	Increased lactate with pH < 7.3 with life-threatening consequences

15 Use the applicable formula (i.e., Cockroft-Gault in mL/min or Schwatrz in mL/min/1.73m^2).

CHEMISTRIES

PARAMETER	GRADE 1 MILD	GRADE 2 MODERATE	GRADE 3 SEVERE	GRADE 4 POTENTIALLY LIFE-THREATENING
**Lipase, High**	1.1 to < 1.5 x ULN	1.5 to < 3.0 x ULN	3.0 to < 5.0 x ULN	≥ 5.0 x ULN
**Lipid Disorders** (mg/dL; *mmol/L*)<br>***Cholesterol, Fasting, High***<br>*≥18 years of age*	200 to < 240<br>*5.18 to < 6.19*	240 to < 300<br>*6.19 to < 7.77*	≥ 300<br>*≥ 7.77*	NA
< 18 years of age	170 to < 200<br>*4.40 to < 5.15*	200 to < 300<br>*5.15 to < 7.77*	≥ 300<br>*≥ 7.77*	NA
LDL, Fasting, High<br>*≥18 years of age*	130 to < 160<br>*3.37 to < 4.12*	160 to < 190<br>*4.12 to < 4.90*	≥ 190<br>*≥ 4.90*	NA
> 2 to < 18 years of age	110 to < 130<br>*2.85 to < 3.34*	130 to < 190<br>*3.34 to < 4.90*	≥ 190<br>*≥ 4.90*	NA
Triglycerides, Fasting, High	150 to 300<br>*1.71 to 3.42*	>300 to 500<br>*>3.42 to 5.7*	>500 to < 1,000<br>*>5.7 to 11.4*	> 1,000<br>*> 11.4*
**Magnesium[16], Low** (mEq/L; *mmol/L*)	1.2 to < 1.4<br>*0.60 to < 0.70*	0.9 to < 1.2<br>*0.45 to < 0.60*	0.6 to < 0.9<br>*0.30 to < 0.45*	< 0.6<br>*< 0.30*
**Phosphate, Low** (mg/dL;<br>*> 14 years of age*	2.0 to < LLN<br>*0.81 to < LLN*	1.4 to < 2.0<br>*0.65 to < 0.81*	1.0 to < 1.4<br>*0.32 to < 0.65*	< 1.0<br>*< 0.32*
1 to 14 years of age	3.0 to < 3.5<br>*0.97 to < 1.13*	2.5 to < 3.0<br>*0.81 to < 0.97*	1.5 to < 2.5<br>*0.48 to < 0.81*	< 1.5<br>*< 0.48*
< 1 year of age	3.5 to < 4.5<br>*1.13 to < 1.45*	2.5 to < 3.5<br>*0.81 to < 1.13*	1.5 to < 2.5<br>*0.48 to < 0.81*	< 1.5<br>*< 0.48*
**Potassium, High** (mEq/L; *mmol/L*)	5.6 to < 6.0<br>*5.6 to < 6.0*	6.0 to < 6.5<br>*6.0 to < 6.5*	6.5 to < 7.0<br>*6.5 to < 7.0*	≥ 7.0<br>*≥ 7.0*
**Potassium, Low** (mEq/L; *mmol/L*)	3.0 to < 3.4<br>*3.0 to < 3.4*	2.5 to < 3.0<br>*2.5 to < 3.0*	2.0 to < 2.5<br>*2.0 to < 2.5*	< 2.0<br>*< 2.0*
**Sodium, High** (mEq/L; *mmol/L*)	146 to < 150<br>*146 to < 150*	150 to < 154<br>*150 to < 154*	154 to < 160<br>*154 to < 160*	≥ 160<br>*≥ 160*
**Sodium, Low** (mEq/L; *mmol/L*)	130 to < 135<br>*130 to < 135*	125 to < 130<br>*125 to < 135*	121 to < 125<br>*121 to < 125*	≤ 120<br>*≤ 120*
**Uric Acid, High** (mg/dL; *mmol/L*)	7.5 to < 10.0<br>*0.45 to < 0.59*	10.0 to < 12.0<br>*0.59 to < 0.71*	12.0 to < 15.0<br>*0.71 to < 0.89*	≥ 15.0<br>*≥ 0.89*

[16] To convert a magnesium value from mg/dL to mmol/L, laboratories should multiply by 0.4114.

HEMATOLOGY

PARAMETER	GRADE 1<br>MILD	GRADE 2<br>MODERATE	GRADE 3<br>SEVERE	GRADE 4<br>POTENTIALLY LIFE-THREATENING
**Absolute CD4+ Count, Low**<br>(cell/mm^3; *cells/L*)<br>*> 5 years of age*<br>*(not HIV infected)*	300 to < 400<br>*300 to < 400*	200 to < 300<br>*200 to < 300*	100 to < 200<br>*100 to < 200*	< 100<br>*< 100*
**Absolute Lymphocyte Count, Low**<br>(cell/mm^3; *cells/L*)<br>*> 5 years of age*<br>*(not HIV infected)*	600 to < 650<br>*0.600 x 10^9 to*<br>*< 0.650 x 10^9*	500 to < 600<br>*0.500 x 10^9 to*<br>*< 0.600 x 10^9*	350 to < 500<br>*0.350 x 10^9 to*<br>*< 0.500 x 10^9*	< 350<br>*< 0.350 x 10^9*
**Absolute Neutrophil Count (ANC), Low**<br>(cells/mm^3; *cells/L*)<br>*> 7 days of age*	800 to 1,000<br>*0.800 x 10^9 to 1.000 x 10^9*	600 to 799<br>*0.600 x 10^9 to 0.799 x 10^9*	400 to 599<br>*0.400 x 10^9 to 0.599 x 10^9*	< 400<br>*< 0.400 x 10^9*
2 to 7 days of age	1,250 to 1,500<br>*1.250 x 10^9 to 1.500 x 10^9*	1,000 to 1,249<br>*1.000 x 10^9 to 1.249 x 10^9*	750 to 999<br>*0.750 x 10^9 to 0.999 x 10^9*	< 750<br>*< 0.750 x 10^9*
≤ 1 day of age	4,000 to 5,000<br>*4.000 x 10^9 to*<br>*5.000 x 10_9*	3,000 to 3,999<br>*3.000 x 10^9 to 3.999 x 10^9*	1,500 to 2,999<br>*1.500 x 10^9 to 2.999 x 10^9*	< 1,500<br>*< 1.500 x 10^9*
**Fibrinogen, Decreased**<br>(mg/dL; *g/L*)	100 to < 200<br>*1.00 to < 2.00*<br>OR<br>0.75 to < 1.00<br>x LLN	75 to < 100<br>*0.75 to < 1.00*<br>OR<br>≥ 0.50 to < 0.75<br>x LLN	50 to < 75<br>*0.50 to < 0.75*<br>OR<br>0.25 to < 0.50<br>x LLN	< 50<br>*< 0.50*<br>OR<br>< 0.25 x LLN<br>OR Associated with gross bleeding
**Hemoglobin[17], Low**<br>(g/dL; *mmol/L*)[18]<br>*≥ 13 years of age*<br>*(male only)*	10.0 to 10.9<br>*6.19 to 6.76*	9.0 to < 10.0<br>*5.57 to < 6.19*	7.0 to < 9.0<br>*4.34 to < 5.57*	< 7.0<br>*< 4.34*
≥ 13 years of age<br>*(female only)*	9.5 to 10.4<br>*5.88 to 6.48*	8.5 to < 9.5<br>*5.25 to < 5.88*	6.5 to < 8.5<br>*4.03 to < 5.25*	< 6.5<br>*< 4.03*

[17] Male and female sex are defined as sex at birth.
[18] The conversion factor used to convert g/dL to mmol/L is 0.6206 and is the most commonly used conversion factor. For grading hemoglobin results obtained by an analytic method with a conversion factor other than 0.6206, the result must be converted to g/dL using the appropriate conversion factor for the particular laboratory.

HEMATOLOGY

PARAMETER	GRADE 1 MILD	GRADE 2 MODERATE	GRADE 3 SEVERE	GRADE 4 POTENTIALLY LIFE-THREATENING
57 days of age to < 13 years of age (male and female)	9.5 to 10.4<br>*5.88 to 6.48*	8.5 to < 9.5<br>*5.25 to < 5.88*	6.5 to < 8.5<br>*4.03 to < 5.25*	< 6.5<br>*< 4.03*
36 to 56 days of age (male and female)	8.5 to 9.6<br>*5.26 to 5.99*	7.0 to < 8.5<br>*4.32 to < 5.26*	6.0 to < 7.0<br>*3.72 to < 4.32*	< 6.0<br>*< 3.72*
22 to 35 days of age (male and female)	9.5 to 11.0<br>*5.88 to 6.86*	8.0 to < 9.5<br>*4.94 to < 5.88*	6.7 to < 8.0<br>*4.15 to < 4.94*	< 6.7<br>*< 4.15*
8 to ≤ 21 days of age (male and female)	11.0 to 13.0<br>*6.81 to 8.10*	9.0 to < 11.0<br>*5.57 to < 6.81*	8.0 to < 9.0<br>*4.96 to < 5.57*	< 8.0<br>*< 4.96*
≤ 7 days of age (male and female)	13.0 to 14.0<br>*8.05 to 8.72*	10.0 to < 13.0<br>*6.19 to < 8.05*	9.0 to < 10.0<br>*5.59 to < 6.19*	< 9.0<br>*< 5.59*
**INR, High** (not on anticoagulation therapy)	1.1 to < 1.5 x ULN	1.5 to < 2.0 x ULN	2.0 to < 3.0 x ULN	≥ 3.0 x ULN
**Methemoglobin** (% hemoglobin)	5.0 to < 10.0%	10.0 to < 15.0%	15.0 to < 20.0%	≥ 20.0%
**PTT, High** (not on anticoagulation therapy)	1.1 to < 1.66 x ULN	1.66 to < 2.33 x ULN	2.33 to < 3.00 x ULN	≥ 3.00 x ULN
**Platelets, Decreased** (cells/mm^3; *cells/L*)	100,000 to < 124,999<br>*100.000 x 10^9 to < 124.999 x 10^9*	50,000 to < 100,000<br>*50.000 x 10^9 to < 100.000 x 10^9*	25,000 to < 50,000<br>*25.000 x 10^9 to < 50.000 x 10^9*	< 25,000<br>*< 25.000 x 10^9*
**PT, High** (not on anticoagulation therapy	1.1 to < 1.25 x ULN	1.25 to < 1.50 x ULN	1.50 to < 3.00 x ULN	≥ 3.00 x ULN
**WBC, Decreased** (cells/mm^3; *cells/L*)<br>*> 7 days of age*	2,000 to 2,499<br>*2.000 x 10^9 to 2.499 x 10^9*	1,500 to 1,999<br>*1.500 x 10^9 to 1.999 x 10^9*	1,000 to 1,499<br>*1.000 x 10^9 to 1.499 x 10^9*	< 1,000<br>*< 1.000 x 10^9*
≤ 7 days of age	5,500 to 6,999<br>*5.500 x 10^9 to 6.999 x 10^9*	4,000 to 5,499<br>*4.000 x 10^9 to 5.499 x 10^9*	2,500 to 3,999<br>*2.500 x 10^9 to 3.999 x 10^9*	< 2,500<br>*< 2.500 x 10^9*

URINALYSIS

PARAMETER	GRADE 1 MILD	GRADE 2 MODERATE	GRADE 3 SEVERE	GRADE 4 POTENTIALLY LIFE-THREATENING
**Glycosuria** (random collection tested by dipstick)	Trace to 1+ or ≤ 250 mg	2+ or > 250 to < 500 mg	> 2+ or > 500 mg	NA
**Hematuria** (not to be reported based on dipstick findings or on blood believed to be of menstrual origin)	6 to < 10 RBCs per high power field	≥ 10 RBCs per high power field	Gross, with or without clots OR With RBC casts OR Intervention indicated	Life-threatening consequences
**Proteinuria** (random collection tested by dipstick)	1+	2+	3+ or higher	NA

International AIDS Vaccine Initiative

DOCUMENT NUMBER:
DOCUMENT TITLE:
DOCUMENT NOTES:

Document Information

Revision:	Vault:
Status:	Document Type:

Date Information

Effective Date:	Expiration Date:
Release Date:	Next Review Date:

Control Information

Author:	Owner:
Previous Number:	Change Number:

Signature Manifest

Document Number: TMF-02-0248 **Revision:** 0

Title: Protocol PGT121/PGDM1400

All dates and times are in Eastern Time Zone.

T002 Protocol

Change Request Approval

Name/Signature	Title	Date	Meaning/Reason
Dani Vooijs (DVOOIJS)			
Katherine Crisafi (KCRISAFI)			
Michele Fong Lim (MFONGLIM)	Director Quality Systems	10 Feb 2017, 10:36:32 AM	Approved

CMO Approval

Name/Signature	Title	Date	Meaning/Reason
Frances Priddy (FPRIDDY)	Chief Medical Officer	12 May 2017, 02:18:23 PM	Approved

QA Final Release

Name/Signature	Title	Date	Meaning/Reason
Dani Vooijs (DVOOIJS)			
Michele Fong Lim (MFONGLIM)			
Katherine Crisafi (KCRISAFI)	Director Laboratory QA	13 May 2017, 03:25:16 AM	Approved

Notify

Name/Signature	Title	Date	Meaning/Reason
Lisa Sunner (LSUNNER)		13 May 2017, 03:25:16 AM	Email Sent

**Protocol Title:**	A Phase 1 Randomized Placebo-controlled Clinical Trial of the Safety, Pharmacokinetics and Antiviral Activity of PGDM1400 and PGT121 Monoclonal Antibodies in HIV-uninfected and HIV-infected Adults
**Protocol Number:**	IAVI T002
**Regulatory Investigational Product Number**	134270
**ClinicalTrials.gov Registry Number**	TBD
**Phase:**	Phase 1
**Sponsor:**	International AIDS Vaccine Initiative (IAVI)<br>125 Broad Street, 9th Floor<br>New York, New York 10004<br>USA
**Sponsor Status**	Not for-Profit Organization
**Date of Protocol Version:**	29 JUNE 2017<br>2.0<br><br>12 MAY 2017<br>1.0

THE CONFIDENTIAL INFORMATION IN THIS DOCUMENT IS PROVIDED TO YOU AS AN INVESTIGATOR, POTENTIAL INVESTIGATOR, OR CONSULTANT, FOR REVIEW BY YOU, YOUR STAFF, AND APPLICABLE INSTITUTIONAL REVIEW BOARDS (IRBS) AND/OR INDEPENDENT ETHICS COMMITTEES (IECS). IT IS UNDERSTOOD THAT THE INFORMATION WILL NOT BE DISCLOSED TO OTHERS, EXCEPT TO THE EXTENT NECESSARY TO OBTAIN ETHICAL AND REGULATORY APPROVAL FROM THE RESPECTIVE COMMITTEE'S AGENCIES AND INFORMED CONSENT FROM THOSE PERSONS TO WHOM THE INVESTIGATIONAL PRODUCT MAY BE ADMINISTERED.

PROTOCOL SYNOPSIS

**TITLE**	A Phase 1 Randomized Placebo-controlled Clinical Trial of the Safety, Pharmacokinetics and Antiviral Activity of PGDM1400 and PGT121 Monoclonal Antibodies in HIV-uninfected and HIV-infected Adults
**PROTOCOL NUMBER**	IAVI T002
**CLINICAL TRIAL PHASE**	Phase 1
**IND SPONSOR**	International AIDS Vaccine Initiative (IAVI)<br>125 Broad Street, 9th Floor<br>New York, New York 10004,<br>USA
**SPONSOR STATUS**	Not for Profit Organization
**SAMPLE SIZE**	36-84
**STUDY POPULATION**	1. HIV-uninfected males or females aged 18-50 years old who are willing to maintain low risk behavior for HIV infection; principal exclusion criteria include confirmed HIV-infection, pregnancy or lactation, significant acute or chronic disease and clinically significant laboratory abnormalities (Groups 1 and 2, see below for full inclusion and exclusion criteria).<br>2. HIV-infected males or females aged 18-65 years old, not on antiretroviral therapy (ART) with HIV-1 viral load between 1000 and 100,000 copies/ml, CD4 cell count ≥ 300 cells/µl; principal exclusion criteria include significant acute or chronic medical condition other than HIV infection, and clinically significant laboratory abnormalities (Group 3, see below for full inclusion and exclusion criteria).
**STUDY DESIGN**	Double blind, randomized, placebo-controlled study for Groups 1 and 2. Open label study for Group 3. Single intravenous (IV) infusion of PGDM1400 mAb alone @ 3, 10 or 30 mg/kg, or a combination of PGDM1400 mAb and PGT121 mAb, each @ 3, 10 or 30 mg/kg, or placebo. See study design table below for details.
**STUDY DURATION**	Up to 32 weeks per participant, screening up to 42 (HIV-infected) or up to 56 (HIV-uninfected) days before single IV infusion of investigational product on day 0, and 24 weeks of follow up.
**INVESTIGATIONAL PRODUCT DESCRIPTION**	**PGDM1400 mAb**: PGDM1400 mAb is a recombinant, fully human monoclonal antibody (mAb) of the IgG1 isotype that binds to the HIV envelope. The concentration of the product is 50 mg/ml. Vials will contain 6 ml of product @ 50 mg/ml = 300 mg.<br>**PGT121 mAb**: PGT121 mAb is a recombinant, fully human monoclonal antibody (mAb) of the IgG1 isotype that binds to the

	HIV envelope. The concentration of the product is 50 mg/ml. Vials will contain 6 ml of product @ 50 mg/ml = 300 mg.<br>**Placebo:** 0.9% Sodium Chloride for injection, USP
**OBJECTIVES**	**Primary Objectives**<br>• To evaluate the safety and tolerability of IV infusion of PGDM1400 mAb alone, and a combination of PGDM1400 mAb and PGT121 mAb in HIV-uninfected adults and HIV-infected adults<br>• To evaluate the pharmacokinetic (PK) profile of IV infusion of PGDM1400 mAb alone, and a combination of PGDM1400 mAb and PGT121 mAb in HIV-uninfected adults and HIV-infected adults<br>• To evaluate the antiviral activity of IV infusion of PGDM1400 mAb alone, and a combination of PGDM1400 mAb and PGT121 mAb in HIV-infected adults not on ART<br><br>**Secondary Objectives**<br>• To determine if PGDM1400 mAb alone, and in combination with PGT121 mAb induces anti-PGDM1400 and/or anti-PGT121 antibodies<br>• To determine the effect of PGDM1400 mAb alone and in combination with PGT121 mAb on CD4+ T cell counts in HIV-infected adults<br>• To determine the effect of PGDM1400 mAb alone and in combination with PGT121 mAb on viral escape in viremic HIV-infected adults not on ART<br><br>**Exploratory Objectives**<br>• To determine if PGDM1400 ± PGT121 mAb has any impact on the host immune responses (i.e., HIV-specific cellular and humoral immune response).<br>• To determine the effect of PGDM1400 ± PGT121 mAb on the size of the latent HIV reservoir in HIV-infected adults.<br>• To determine PGDM1400 mAb ± PGT121 mAb levels in mucosal secretions in HIV-uninfected and HIV-infected adults.<br>• To measure in vitro neutralization of HIV isolates with participant's serum post PGDM1400 ± PGT121 mAb IV infusion
**ENDPOINTS**	**Primary Endpoints**<br>*Safety and Tolerability*<br>1. Proportion of participants with moderate or greater reactogenicity (e.g., solicited adverse events) for 3 days following IV infusion of PGDM1400 mAb alone, and a combination of PGDM1400 mAb and PGT121 mAb.<br>2. Proportion of participants with adverse events (AEs), including safety laboratory (biochemical, hematological) parameters, during the first 56 days following IV infusion of PGDM1400

mAb alone and a combination of PGDM1400 mAb and PGT121 mAb, that are moderate or greater, and/or related to PGDM1400 mAb or PGT121 mAb.

3. Proportion of participants with serious adverse events (SAEs) throughout the study period following IV infusion of PGDM1400 mAb alone and a combination of PGDM1400 mAb and PGT121 mAb, that are related to PGDM1400 mAb or PGT121 mAb.

Pharmacokinetics

Pharmacokinetics following IV infusion of PGDM1400 mAb alone or a combination of PGDM1400 mAb and PGT121 mAb in HIV-uninfected and HIV-infected adults:

- Elimination half-life (t1/2)
- Clearance (CL/F)
- Volume of distribution (Vz/F)
- Area under the concentration decay curve (AUC)
- Impact of viral load and/or ART on PGDM1400 mAb and PGT121 mAb disposition (elimination half-life (t1/2), clearance (CL/F), volume of distribution (Vz/F), total exposure)

Antiviral activity

Antiviral activity following IV infusion of PGDM1400 mAb alone or in combination with PGT121 mAb in viremic HIV-infected adults not on ART:

1. Change in plasma HIV-1 RNA levels from baseline (mean of pre-entry and entry values)

Secondary Endpoints

Anti-PGDM1400 and anti-PGT121 antibodies

1. Serum anti-PGDM1400 antibody titers
2. Serum anti-PGT121 antibody titers

CD4+ T cell count

We will calculate the following endpoint to determine if IV infusion of PGDM1400 mAb alone or in combination with PGT121 mAb has any impact on CD4+ T cell counts in HIV-infected adults:

1. Change in CD4+ T cell count and frequency compared to baseline as measured by single platform flow cytometry.

HIV genotyping/phenotyping of circulating virus for evaluation of PGDM1400 mAb and/or PGT121 mAb -induced escape mutations

We will compare plasma virus genotype and phenotypic activity before and after IV infusion of PGDM1400 mAb alone or in combination with PGT121 mAb to determine the effect of PGDM1400 mAb and/or PGT121 mAb on viral escape mutations in viremic HIV-infected adults not on ART.

1. Genotypic analysis: Development of sequence variations in

	epitopes known to result in reduced PGDM1400 mAb and/or PGT121 mAb neutralization susceptibility or known to cause resistance to antiretroviral drugs.<br>2. Phenotypic analysis: Changes in viral susceptibility to PGDM1400 mAb and/or PGT121 mAb neutralization.<br><br>**Exploratory Endpoints**<br>Additional assessments may include but are not limited to the following: HIV-specific IgG/IgA binding responses by ELISA, HIV-specific cellular immune responses by ELISPOT, HIV-specific antibody function by ADCC, ADCP, and ADCVI assays, PGDM1400 mAb and/or PGT121 mAb levels in mucosal secretions, changes in total HIV-1 DNA and 2-long terminal repeat (LTR) circular HIV-1 DNA in resting or total CD4 T cells and in vitro neutralization of HIV isolates with participant's serum post IV infusion of PGDM1400 mAb ± PGT121 mAb.

STUDY DESIGN TABLE

	Group	Participants	Sub-Group	Regimen	N	Dose (mg/kg)
Part 1 – MTD	1	HIV-uninfected participants	1A	PGDM1400/Placebo	3/1 (6/2 if DLT)	3 IV
			1B	PGDM1400/Placebo	3/1 (6/2 if DLT)	10 IV
			1C	PGDM1400/Placebo	3/1 (6/2 if DLT)	30 IV
				Total Group 1	9/3 = 12 (max 18/6 = 24 if DLT)	
	2	HIV-uninfected participants	2A	PGDM1400 + PGT121/Placebo	3/1 (6/2 if DLT)	3 + 3 IV
			2B	PGDM1400 + PGT121/Placebo	3/1 (6/2 if DLT)	10 + 10 IV
			2C	PGDM1400 + PGT121/Placebo	3/1 (6/2 if DLT)	30 + 30 IV
				Total Group 2	9/3 = 12 (max 18/6 = 24 if DLT)	
				Total Groups 1 and 2	18/6 = 24 (max 36/12 = 48 if DLT)	
Safety Monitoring Committee						
Part 2 – antiviral effect	3	HIV-infected off ART (VL $1x10^3$ – $1x10^5$ copies/ml)	3A	PGDM1400	6 (max 18)	MTD IV
			3B	PGDM1400+PGT121	6 (max 18)	MTD IV
				Total Group 3	12 (max 36)	
				Total entire study	36 (max 84)	

DLT, dose limiting toxicity; MTD, maximum tolerated dose

<table>
<tr><td>SAFETY MONITORING</td><td>Safety will continually be monitored by the Investigators, the Sponsor's Medical Monitor and a Protocol Safety Review Team (PSRT). Safety data will be reviewed by an independent Safety Monitoring Committee (SMC).

- Sentinel participants in Groups 1 and 2
For each dose Subgroup in Groups 1 and 2, the 1st 2 participants will be sentinel participants for whom investigational product infusion will be separated by at least 24 hours contingent on review of safety information prior to infusion of the next sentinel participant. Both sentinel participants are observed for at least 24 hours before IV infusion of investigational product to the 3rd and subsequent participants.

- Dose limiting toxicity in Groups 1 and 2
Dose limiting toxicity (DLT) will be defined as 1) any Grade 3 or greater adverse event considered possibly, probably or definitely related to investigational product or 2) any Grade 3 or greater reactogenicity or 3) any SAE considered possibly, probably or definitely related to investigational product.

- Infusion related reactions
Infusion related reactions, including cytokine release syndrome, will be graded using the NCI Common Terminology Criteria for Adverse Events (CTCAE) version 4.03 (June 14, 2010). All other adverse events will be graded using the Division of AIDS (DAIDS) Table for Grading the Severity of Adult and Pediatric Adverse Events version 2.0 (November 2014).</td></tr>
<tr><td>DOSE ESCALATION IN GROUPS 1 AND 2</td><td>Part 1 of this study is a dose-escalation trial in HIV-uninfected adults (Groups 1 and 2) to establish the MTD of PGDM1400 mAb and a combination of PGDM1400 mAb and PGT121 mAb.

- PSRT review for dose escalation
The Protocol Safety Review Team (PSRT) will review safety data through day 14 post-investigational product infusion for all participants in each dose Subgroup for PGDM1400 mAb alone (Group 1) prior to allowing enrolment of participants into the next higher dose Subgroup in Group 1 or the same dose level PGDM1400 mAb and PGT121 mAb combination dose Subgroup (Group 2).
o If no DLT occurs in the initial 4 participants of a dose Subgroup (e.g., 1A), the study can proceed with enrolment of the next dose Subgroup for PGDM1400 mAb alone (e.g., 1B), and the same dose level PGDM1400 mAb and PGT121 mAb combination dose Subgroup (e.g., 2A).
o If 1 DLT occurs in the initial 4 participants of a dose Subgroup (e.g., 1A), 4 additional participants will be enrolled in the same dose Subgroup.
▪ If no additional DLTs occur within 14 days of infusion in</td></tr>
</table>

	the 8 total participants, the study can proceed with enrolment of the next dose Subgroup for PGDM1400 mAb alone (e.g., 1B), and the same dose level PGDM1400 mAb and PGT121 mAb combination dose Subgroup (e.g., 2A).<br>▪ In Subgroups receiving PGDM1400 alone (1A, 1B, 1C), if 2 or more DLTs accumulate in a dose Subgroup (e.g., 1B) that are the same, similar, or in the same System Organ Class, infusion will be halted and the next lower dose level will be declared the maximum tolerated dose (MTD) within this Group (e.g., 3 mg/kg if the DLTs occurred in dose Subgroup 1B @ 10 mg/kg) , and the same dose level PGDM1400 mAb and PGT121 mAb combination dose Subgroup (2A in this example). Group 2 will not proceed with escalation beyond the MTDs of PGDM1400 alone and PGT121 alone.<br>▪ If no DLT occurs in the final dose Subgroup (1C) after 14 days of follow-up, the MTD for PGDM1400 alone will be the highest dose given (30mg/kg).<br>▪ In Subgroups receiving PGDM1400 and PGT121 (2A, 2B, 2C), if 2 or more DLTs occur in a dose Subgroup (e.g., 2B) that are the same, similar, or in the same System Organ Class, infusion will be halted and the next lower dose level will be declared the MTD for the combination of PGDM1400 and PGT121.<br>▪ If no DLT occurs in the final dose Subgroup (2C) after 14 days of follow-up , MTD for PGDM1400 and PGT121 will be the highest dose given (30mg/kg each).<br><br>- *SMC review to determine MTD of PGDM1400 alone*<br>Following IV infusion of investigational product in the last participant in Group 1, an independent Safety Monitoring Committee (SMC) will review safety data through day 14 post-investigational product infusion for all participants to confirm MTD of PGDM1400 alone, and determine whether, and at what dose, Group 3A can initiate enrolment.<br><br>- *SMC review to determine MTD of the combination of PGDM1400 and PGT121*<br>Following IV infusion of investigational product in the last participant in Group 2, the SMC will review safety data through day 14 post-investigational product infusion for all participants to confirm MTD of the combination of PGDM1400 and PGT121, and determine whether Group 3B can initiate enrollment.
**ANTIVIRAL ACTIVITY IN GROUP 3**	Part 2 of this study will establish the antiviral effect of PGDM1400 mAb alone or the combination of PGDM1400 mAb plus PGT121 mAb in HIV infected adults not on ART (Group 3).<br><br>- *MTD determines dose*

	Group 3 will start with the MTD of PGDM1400 as determined by the SMC; e.g., if the MTD for PGDM1400 is 30mg/kg then Subgroup 3A will receive 30mg/kg and Subgroup 3B will receive PGDM1400 mAb + PGT121 mAb at a dose that combines the MTD for PGDM1400 mAb and the MTD for PGT121 mAb, respectively.
**PAUSE RULES**	The study will be paused for a safety review by the investigators and the independent SMC if:<br>1. 1 or more participants experience a Serious Adverse Event that is judged possibly, probably or definitely related to the investigational product.<br>2. There is a participant death regardless of relationship to the investigational product.<br>3. If 2 or more participants experience Grade 3 adverse events in the same System Organ Class that are considered possibly, probably, or definitely related to investigational product.<br>4. Any Grade 4 adverse event that is considered possibly, probably or definitely related to investigational product.
**EVALUATION FOR INTERCURRENT HIV INFECTION:**	Participants in Groups 1 and 2 (HIV-uninfected) will be tested for HIV according to the Schedule of Procedures. Test results will be interpreted according to a pre-determined diagnostic algorithm. HIV testing at additional time points may be performed upon the request of the participant and Principal Investigator or designee as medical or social circumstances warrant.
**INCLUSION CRITERIA**	**Inclusion criteria for all participants:**<br>1. Willing to comply with the requirements of the protocol and available for follow-up for the planned duration of the study.<br>2. In the opinion of the Principal Investigator or designee and based on Assessment of Informed Consent Understanding results, has understood the information provided and potential impact and/or risks linked to IV infusion and participation in the trial; written informed consent will be obtained from the participant before any study-related procedures are performed.<br>3. All heterosexually active female participants must commit to use an effective method of contraception for 3 months following investigational product administration, including:<br>a. Condoms (male or female) with or without spermicide<br>b. Diaphragm or cervical cap with spermicide<br>c. Intrauterine device, or contraceptive implant<br>d. Hormonal contraception<br>e. Successful vasectomy in the male partner (considered successful if a woman reports that a male partner has [1] documentation of azoospermia by microscopy (< 1 year ago), or [2] a vasectomy more than 2 years ago with no resultant pregnancy despite sexual activity post-vasectomy)<br>f. Not be of reproductive potential, such as having undergone

hysterectomy, bilateral oophorectomy, or tubal ligation, postmenopausal (> 45 years of age with amenorrhea for at least 2 years, or any age with amenorrhea for at least 6 months and a serum follicle stimulating hormone [FSH] level > 40 IU/L); surgically sterile: no additional contraception required.

Women, who are not heterosexually active at screening, must agree to utilize an effective method of contraception if they become hetero-sexually active, as outlined above.

4. All sexually active males, regardless of reproductive potential, must be willing to consistently use an effective method of contraception (such as consistent male condoms with male and/or female partners) from the day of investigational product administration until at least 3 months following investigational product administration to avoid exposure of partners to investigational product in ejaculate, and to prevent conception with female partners.
5. All female participants must be willing to undergo urine pregnancy tests at time points indicated in the Schedule of Procedures and must test negative prior to investigational product administration.
6. A female participant must agree not to donate eggs (ova, oocytes) for the purpose of assisted reproduction until 3 months after investigational product administration. A man must agree not to donate sperm until 3 months after investigational product administration.
7. Willing to forgo donations of blood and/or any other tissues, including bone marrow, during the study and, for those HIV-uninfected participants who test HIV-positive due to investigational product administration, until the anti-HIV antibody titers become undetectable.

Specific inclusion criteria for HIV-uninfected participants (Groups 1 and 2):

8. At least 18 years of age on the day of screening and has not reached his or her 51st birthday on the day of signing the Informed Consent Document.
9. Willing to undergo HIV testing, risk reduction counselling and receive HIV test results.
10. Low risk for HIV infection and willing to maintain low-risk behavior for the duration of the trial.
11. Healthy male or female, as assessed by a medical history, physical exam, and laboratory tests.

Specific inclusion criteria for HIV-infected participants (Group 3):

12. At least 18 years of age on the day of screening and has not reached his or her 66th birthday on the day of signing the Informed Consent Document.
13. Confirmed HIV-1 infection (HIV Ab+ or HIV RNA+) by documentation in the medical records or in-clinic HIV testing;
14. CD4 ≥ 300 cells/μl.

	15. Not receiving cART, and (after appropriate counselling) willing to defer cART treatment for at least 56 days after administration of investigational product.<br>16. HIV-1 viral load between 1000–100,000 copies/ml, confirmed at screening.
**EXCLUSION CRITERIA**	**Exclusion criteria for all participants:**<br>1. Any clinically significant acute or chronic medical condition, other than HIV infection, that is considered progressive or in the opinion of the investigator makes the participant unsuitable for participation in the study.<br>2. If female, pregnant, lactating or planning a pregnancy during the period of screening through completion of the study.<br>3. In the past 6 months a history of alcohol or substance use, including marijuana, judged by the Investigator to potentially interfere with participant study compliance.<br>4. Bleeding disorder that was diagnosed by a physician (e.g., factor deficiency, coagulopathy or platelet disorder that requires special precautions). Note: A participant who states that he or she has easy bruising or bleeding, but does not have a formal diagnosis and has intramuscular injections and blood draws without any adverse experience, is eligible.<br>5. History of a splenectomy.<br>6. Receipt of live attenuated vaccine within the previous 30 days or planned receipt within 30 days after administration of investigational product; or receipt of other vaccine within the previous 14 days or planned receipt within 14 days after infusion with investigational product (exception is live attenuated influenza vaccine within 14 days).<br>7. Receipt of blood transfusion or blood-derived products within the previous 3 months.<br>8. Participation in another clinical trial of an investigational product currently, within the previous 3 months or expected participation during this study.<br>9. Prior receipt of an investigational HIV vaccine candidate, monoclonal antibody or polyclonal immunoglobulin (note: receipt of placebo in a previous HIV vaccine or monoclonal antibody trial will not exclude a participant from participation if documentation is available and the Medical Monitor gives approval).<br>10. History of severe local or systemic reactogenicity to injections or IV infusion (e.g., anaphylaxis, respiratory difficulties, angioedema);<br>11. Psychiatric condition that compromises safety of the participant and precludes compliance with the protocol. Specifically excluded are persons with psychoses within the past 3 years, ongoing risk for suicide, or history of suicide attempt or gesture within the past 3 years.<br>12. If, in the opinion of the Principal Investigator, it is not in the best interest of the participant to participate in the trial.

13. Seizure disorder: a participant who has had a seizure in the last 3 years is excluded. (Not excluded: a participant with a history of seizures who has neither required medications nor had a seizure for 3 years.)
14. Body mass index ≥ 35 or ≤ 18.0.
15. Infectious disease: chronic hepatitis B infection (HbsAg), current hepatitis C infection (HCV Ab positive and HCV RNA positive) or interferon-alfa treatment for chronic hepatitis C infection in the past year, or active syphilis.
16. A history of malignancy within the past 5 years (prior to screening) or ongoing malignancy;
17. Active, serious infections (other than HIV-1 infection) requiring parenteral antibiotic, antiviral or antifungal therapy within 30 days prior to enrollment.

Specific exclusion criteria for HIV-uninfected participants (Groups 1 and 2):

18. Confirmed HIV-1 or HIV-2 infection.
19. Any clinically relevant abnormality on history or examination including history of immunodeficiency or autoimmune disease; use of systemic corticosteroids, immunosuppressive, anticancer, or other medications considered significant by the investigator within the previous 6 months.

    The following exceptions are permitted and will not exclude study participation: use of corticosteroid nasal spray for rhinitis, topical corticosteroids for an acute uncomplicated dermatitis; or a short course (duration of 10 days or less, or a single injection) of corticosteroid for a non-chronic condition (based on investigator clinical judgment) at least 6 weeks prior to enrollment in this study.
20. Any of the following abnormal laboratory parameters listed below:

Hematology

- Hemoglobin < 10.5 g/dL in females; hemoglobin <11.0 g/dL in males
- Absolute Neutrophil Count (ANC): ≤ 1000/mm3
- Absolute Lymphocyte Count (ALC): < 650/mm3
- Platelets: < 125,000 mm3 or ≥ 550,000/mm3

Coagulation

- aPTT: >1.25 x ULN
- INR: ≥1.1 x ULN

Chemistry

- Sodium ≤ 135 mEq/L or ≥ 146 mEq/L
- Potassium ≤ 3.4 mEq/L or ≥ 5.6 mEq/L
- Creatinine ≥ 1.1 x ULN
- AST ≥ 1.25 x ULN
- ALT ≥ 1.25 x ULN
- Total bilirubin ≥ 1.25 x ULN
- Alkaline phosphatase ≥ 1.25 x ULN
- Albumin ≤ 3.0 g/dL or ≤ 30 g/L

- Creatine kinase ≥ 3.0 x ULN
- C-reactive protein > 10 mg/L
- C3 complement < 82 mg/dL
- C4 complement < 14 mg/dL

Urinalysis

Any of the following abnormal findings if consistent with clinically significant disease:

- o Protein = greater than trace on dipstick confirmed by microscopic urinalysis outside institutional range.
- o Blood = greater than trace on dipstick confirmed by >3 RBCs/hpf on microscopic urinalysis (not due to menses).

Specific exclusion criteria for HIV-infected participants who are not on ART (Group 3):

21. Any clinically relevant abnormality on history or examination including history of immunodeficiency or autoimmune disease, other than HIV; use of systemic corticosteroids, immunosuppressive, anticancer, or other medications considered significant by the investigator within the previous 6 months.

The following exceptions are permitted and will not exclude study participation: use of corticosteroid nasal spray for rhinitis, topical corticosteroids for an acute uncomplicated dermatitis; or a short course (duration of 10 days or less, or a single injection) of corticosteroid for a non-chronic condition (based on investigator clinical judgment) at least 6 weeks prior to enrollment in this study.

22. Any of the following abnormal laboratory parameters listed below:

Hematology

- Hemoglobin < 10.0 g/dL
- Absolute Neutrophil Count (ANC): < 1000 cells/mm3
- Platelets: < 100,000 cells/mm3

Coagulation

- aPTT: > 1.25 x ULN
- INR: ≥ 1.1 x ULN

Chemistry

- Estimated Glomerular filtration rate (GFR) < 80 mL/min according to the Cockcroft Gault formula for creatinine clearance
    - o Male: (140 – age in years) x (wt in kg) = CLcr (mL/min) / 72 x (serum creatinine in mg/dL)
    - o Female: (140 – age in years) x (wt in kg) x 0.85 = CLcr (mL/min) / 72 x (serum creatinine in mg/dL)
- AST ≥ 2.5 x ULN
- ALT ≥ 2.5 x ULN
- Total bilirubin ≥ 1.6 x ULN
- Alkaline phosphatase ≥ 5 x ULN

Urinalysis

Any of the following abnormal findings if consistent with

	clinically significant disease:<br>o Protein = greater than 1+ on dipstick confirmed by microscopic urinalysis outside institutional range.<br>o Blood = greater than 1+ on dipstick confirmed by > 10 RBCs/hpf on microscopic urinalysis (not due to menses).<br>o Leukocytes = greater than 1+ on dipstick confirmed by > 10 WBC/hpf on microscopic urinalysis.

TABLE OF CONTENTS

PROTOCOL SYNOPSIS **2**
TABLE OF CONTENTS **14**
ABBREVIATIONS **18**
CONTACT INFORMATION **19**
1.0 SIGNATURE PAGE **20**
2.0 INTRODUCTION AND BACKGROUND INFORMATION **21**
2.1 Study Rationale 21
2.2 Clinical experience with PGDM1400 and PGT121 22
3.0 STUDY OBJECTIVES **23**
3.1 Primary Objectives 23
3.2 Secondary Objectives 23
3.3 Exploratory Objectives 23
4.0 STUDY ENDPOINTS **23**
4.1 Primary Endpoints 23
4.2 Secondary Endpoints 24
4.3 Exploratory Endpoints 24
5.0 STUDY DESIGN **25**
5.1 Definition of Dose Limiting Toxicity- Groups 1 and 2 25
5.2 Definition of Maximum Tolerated Dose- Groups 1 and 2 25
5.3 Dose Escalation and Determination of Maximum Tolerated Dose – Groups 1 and 2 25
5.4 Determination of Antiviral Effect – Group 3 27
5.5 Duration of the Study 27
5.6 Study Population 27
5.7 Inclusion Criteria 28
5.8 Exclusion Criteria 29
5.9 Recruitment of Participants 32
6.0 STUDY VISITS **33**
6.1 Screening Period 33
6.2 IV infusion of Investigational Product Visit 33
6.3 Post-IV infusion of Investigational Product Visits 34
6.4 Additional Follow-up Visits 35
6.5 Unscheduled Visits 35
6.6 Final Study Visit or Early Termination Visit 35
7.0 STUDY PROCEDURES **36**
7.1 Informed Consent Process 36
7.2 Medical History and Physical Examination 36
7.3 HIV Testing and HIV-test Counselling (Groups 1 and 2) 37
7.4 HIV Risk Reduction Counselling 37
7.5 Family Planning Counselling 37
7.6 ART Counselling (Group 3) 37
7.7 Specimens 37

7.8 Reimbursement 38
7.9 Randomization and Blinding 38
7.10 Un-blinding Procedure for Individual Participants 38
7.11 Assessment of PGDM1400 mAb and/or PGT121 mAb related HIV sero-positivity 39

8.0 INVESTIGATIONAL PRODUCT 39

8.1 Description 39
8.2 Shipment and Storage 40
8.3 Preparation of investigational product 40
8.4 Administration of investigational product 40
8.5 Accountability and Disposal of investigational product 40

9.0 ASSESSMENTS 41

9.1 Safety Assessments 41
9.1.1 Local reactogenicity 41
9.1.2 Systemic reactogenicity 41
9.1.3 Vital Signs 41
9.1.4 Other Adverse Events 42
9.1.5 Concomitant Medications 42
9.1.6 Routine laboratory parameters 42
9.1.7 Specific screening tests: 43
9.2 Virologic Assessments 43
9.3 Exploratory Immunogenicity Assessments 43
9.3.1 Antibody Responses 43
9.3.2 Cellular Responses 44
9.3.3 PBMC, Serum and Plasma Storage 44
9.4 Other Assessments 44
9.4.1 HIV Antibody Testing (Groups 1 and 2) 44
9.4.2 Pharmacokinetics 44
9.4.3 HLA Typing 45
9.4.5 Pregnancy Test 45
9.4.6 HIV Risk Assessment (Group 1 and 2) 45
9.4.7 Social Impact Assessment 45

10.0 ADVERSE EVENTS 45

10.1 Definition 45
10.2 Assessment of Severity of Adverse Events 45
10.3 Relationship to Investigational Product 46
10.4 Serious Adverse Events 47
10.5 Reporting Potential Immune-Mediated Diseases 48
10.6 Clinical Management of Adverse Events 49
10.7 Clinical Management of Infusion-Related Reactions and Stopping Criteria 49
10.8 Pregnancy 51
10.9 Intercurrent HIV Infection (Group 1 and 2) 51

11.0 MANAGEMENT OF HIV ISSUES DURING AND FOLLOWING STUDY 51

11.1 HIV Testing – Groups 1 and 2 51
11.2 Social Discrimination as a Result of investigational product-related antibodies 52
11.3 HIV infection – Group 1 and 2 52
11.3.1 Counselling 52
11.3.2 Referral for Support/Care 52

12.0 WITHDRAWAL FROM STUDY 52

12.1 Deferral of IV infusion of investigational product 52
12.2 Withdrawal from the Study (Early Termination) 53

13.0 DATA HANDLING 53

13.1 Data Collection and Record Keeping at the Study Site 53
13.2 Data Entry at the Study Site 54
13.3 Data Analysis 54

14.0 STATISTICAL CONSIDERATIONS 54

14.1 Safety and Tolerability Analysis 54
14.2 Pharmacokinetic Analysis 55
14.3. Virologic Analysis for Group 3 56
14.5 Secondary and Exploratory Immunologic and Virologic Analyses 57

15.0 QUALITY CONTROL AND QUALITY ASSURANCE 58

16.0 DATA AND BIOLOGICAL MATERIAL 58

17.0 ADMINISTRATIVE STRUCTURE 59

17.1 Protocol Safety Review Team 59
17.2 Safety Monitoring Committee (SMC) 59
17.2.1 Content of Interim Safety Review 59
17.2.2 SMC Review of Group 1 and 2 data prior to starting Group 3 60
17.3 Criteria for Pausing the Study 60
17.4 Study Supervision 61
17.5 Study Monitoring 62
17.6 Investigator's Records 62

18.0 INDEMNITY 62

19.0 PUBLICATION 62

20.0 ETHICAL CONSIDERATIONS 63

APPENDIX A: SCHEDULE OF PROCEDURES – GROUP 1 (A, B, C) 64

APPENDIX B: SCHEDULE OF PROCEDURES – AND GROUP 2 (A, B, C) 66

APPENDIX C: SCHEDULE OF PROCEDURES – GROUP 3 (A, B) 68

APPENDIX D: LOW RISK CRITERIA 71

APPENDIX E REFERENCES 73

APPENDIX F CTCAE TABLE 76

APPENDIX G: DAIDS ADVERSE EVENT SEVERITY ASSESSMENT TABLE 87

Introduction 90
Major Clinical Conditions Cardiovascular 94
Cardiovascular 96
Dermatologic 97
Endocrine and Metabolic 98
Gastrointestinal 99
Gastrointestinal 100
Musculoskeletal 101
Neurologic 102
Neurologic 103
Pregnancy, Puerperium, and Perinatal 103

Psychiatric 104
Respiratory 105
Sensory 106
Systemic 107
Systemic 108
Urinary 109
Site Reactions to Injections and Infusions 110
Laboratory Values Chemistries 111

ABBREVIATIONS

Abbreviation	Term
**AE**	Adverse Event
**AIDS**	Acquired Immunodeficiency Syndrome
**ALT**	Alanine-Aminotransferase
**ART**	Antiretroviral Therapy
**AST**	Aspartate-Aminotransferase
**CFC**	Cytokine Flow Cytometry
**CMI**	Cell Mediated Immunity
**CRF**	Case Report Form
**CTL**	Cytotoxic T Lymphocyte
**DCC**	Data Coordinating Center
**DLT**	Dose Limiting Toxicity
**DNA**	Deoxyribonucleic Acid
**ELISA**	Enzyme Linked Immunosorbent Assay
**GCP**	Good Clinical Practice
**GFR**	Glomerular Filtration Rate
**HIV**	Human Immunodeficiency Virus
**HLA**	Human Leukocyte Antigen
**HSV**	Herpes Simplex Virus
**IAVI**	International AIDS Vaccine Initiative
**ICH**	International Council for Harmonization of Technical Requirements for Pharmaceuticals for Human Use
**IP**	Investigational Product
**IND**	Investigational New Drug Application
**IV**	Intravenous
**Kg**	Kilogram
**mAb**	Monoclonal Antibody
**mg**	Milligram
**MTD**	Maximum Tolerated Dose
**NHP**	Non Human Primate
**PCR**	Polymerase Chain Reaction
**PBMC**	Peripheral Blood Mononuclear Cells
**PK**	Pharmacokinetic
**RPR**	Rapid Plasma Reagin
**SAE**	Serious Adverse Event
**SIV**	Simian Immunodeficiency Virus
**SOP**	Standard Operating Procedure
**SOM**	Study Operations Manual
**SMC**	Safety Monitoring Committee
**STD**	Sexually Transmitted Disease
**TPHA**	Treponema Pallidum Hemagglutination

CONTACT INFORMATION

Detailed contact information provided in the Study Operation Manual (SOM)

**Sponsor Contact:**	
Frances Priddy MD MPH<br>Chief Medical Officer and Executive Director<br>International AIDS Vaccine Initiative<br>125 Broad Street, 9th Floor<br>New York, New York 10004	Phone: +1-212-328-7461<br>Mobile: +1-646-287-8943<br>Fax: +1-608-203-5501<br>E-mail: fpriddy@iavi.org
**Clinical Research Center Contacts:**	
Boris D. Juelg MD PhD<br>Center for Virology and Vaccine Research<br>Clinical Trials Unit<br>Beth Israel Deaconess Medical Center<br>E / CLS – 10th Floor, Room 1046<br>330 Brookline Avenue<br>Boston, Massachusetts 02215	Phone: +1-857-268-7088<br>Mobile: +1-617-401-6725<br>Fax: +1-617-735-4566<br>E-mail: bjulg@bidmc.harvard.edu

.

1.0 SIGNATURE PAGE

The signatures below constitute the approval of this protocol and the appendices and provide the necessary assurances that this study will be conducted in compliance with the protocol, Good Clinical Practices (GCP) and the applicable regulatory requirement(s).

Sponsor:

Signed: See electronic signature manifest Date:

Frances Priddy MD MPH
Chief Medical Officer and Executive Director, Medical Affairs, IAVI

Principal Investigator:

Signed: Date:

Name (please print):

Name of institution (please print):

2.0 INTRODUCTION AND BACKGROUND INFORMATION

In 2015, 36.7 million people globally were living with HIV, 2.1 million people became newly infected with HIV, and 1.1 million people died from AIDS-related illnesses (UNAIDS 2016). More than 78 million people have become infected with HIV and 39 million people have died since the beginning of the AIDS epidemic (UNAIDS 2016). One reason that such high rates of AIDS-related deaths continue to occur globally – despite the advent of drugs that are highly effective at suppressing HIV replication – is that by June 2016, 18.2 million people (two in four people living with HIV) were accessing antiretroviral therapy (ART) (UNAIDS 2016). Another reason for continued AIDS-related mortality is that ART does not cure HIV infection and must be maintained for a lifetime (Barouch and Deeks 2014). Even in the United States (US), only 30% of the 1.2 million people living with HIV have suppressed HIV to undetectable levels, likely because 1) not everyone is aware of their HIV-positive status, 2) not everyone is accessing ART, and 3) of the challenge of maintaining adequate continuity of ART over many years (CDC 2014).

2.1 Study Rationale

This is a Phase 1 study to evaluate the safety, tolerability, pharmacokinetics and anti-viral efficacy of the PGDM1400 and PGT121 mAbs for HIV prevention and therapy. PGDM1400 mAb and PGT121 mAb are recombinant human IgG1 monoclonal antibodies that target a V1V2 (PGDM1400) and a V3 glycan-dependent (PGT121) epitope region of the HIV envelope protein (Jardine, Julien et al. 2013, Sok, Doores et al. 2014). PGDM1400 and PGT121 mAbs were chosen for this study because of their potency, their ability to neutralize a wide array of cross-clade HIV viruses in a complementary pattern, and their proven antiviral activity in animals studies e.g., their capacity to robustly prevent and treat simian-human immunodeficiency virus (SHIV) in rhesus monkeys.

Some HIV-infected individuals develop HIV specific antibodies capable of neutralizing a broad range of HIV virus strains (Simek, Rida et al. 2009, Walker, Phogat et al. 2009). By selecting and cloning B-cells from such HIV-infected individuals, monoclonal antibodies have been identified which have potent and broadly neutralizing activity against a wide range of HIV virus strains (Walker LM 2010). New developments in high throughput single-cell BCR-amplification and novel soluble Env baits have led to the isolation of monoclonal antibodies with extraordinary potency and breadth (Walker, Huber et al. 2011).

These HIV-specific antibodies target the HIV envelope (Env) and can prevent SHIV infection in rhesus monkeys and have been shown to reduce HIV RNA levels in humans temporarily (Hessell, Poignard et al. 2009, Hessell, Rakasz et al. 2009, Moldt, Rakasz et al. 2012, Barouch, Whitney et al. 2013, Caskey, Klein et al. 2015, Caskey, Schoofs et al. 2017). These broadly neutralizing antibodies (bnAbs) may be effective for prevention of HIV infection when administered passively (Haynes and McElrath 2013, Burton and Mascola 2015). It is likely that bnAbs targeting different HIV epitopes will need to be used in combination to prevent development of resistance and to adequately cover all global HIV strains.

PGDM1400 can neutralize a wide array of HIV-1 viruses *in vitro* and can treat and prevent simian-human immunodeficiency virus (SHIV) in the NHP model (Sok, van Gils et al. 2014) and (Julg et al, unpublished data).
PGDM1400 is a novel monoclonal antibody that targets the V1V2 loop on the HIV Env spike, distinct from VRC01 and 3BNC117 which both target the CD4 binding site, and PGT121 and 10-1074 which target the V3 loop.

PGDM1400 mAb and PGT121 mAb were selected for development because of the following critical attributes:

1. PGDM1400 neutralizes 83% of global viral isolates at a median IC50 of 0.003 µg/mL (Sok et al 2014) and is therefore 10 to 100-fold more potent than the previous best-in-class CD4bs antibodies VRC01, VRC07, and 3BNC117 (Scheid, Mouquet et al. 2011, Walker, Huber et al. 2011, Sok, van Gils et al. 2014)
2. PGDM1400 protects against SHIV acquisition in monkeys at substantially lower dose levels compared to VRC01 and 3BNC117 (unpublished data)
3. PGT121 mAb has superior therapeutic efficacy in SHIV-infected monkeys compared to VRC01, 3BNC117, and 10-1074 (Barouch, Whitney et al. 2013) (and unpublished data). Specifically, SHIV-infected monkeys with low baseline viral load achieved and maintained undetectable viral loads for up to 100 days after administration of PGT121 (Barouch, Whitney et al. 2013) (and unpublished data).
4. PGT121 mAb may have a higher bar to escape in vivo as compared to other V3 glycan and CD4bs antibodies as a result of making multiple glycan contacts (Sok, van Gils et al. 2014).
5. PGDM1400 and PGT121 are complementary in the coverage of global viral isolates and when combined neutralize 98-99% of global HIV-1 viruses tested and at unparalleled potency with a median IC50 of 0.007 µg/ml (Sok, van Gils et al. 2014).

The potency and breadth of PGDM1400 mAb and PGT121 mAb, both alone and in combination with other bNAbs, raise the possibility that combinations may be effective for HIV prophylaxis at low doses and against global viruses. An antibody that is effective at low doses may eventually be given subcutaneously, which would reduce the cost. It is these features that make PGDM1400 mAb and PGT121 mAb particularly well-suited for preventing and/or treating HIV in the developing world, where it is critical that a public health intervention be low cost, easy to deliver, and effective in diverse settings.

2.2 Clinical experience with PGDM1400 and PGT121

There is no previous clinical experience with PGDM1400 mAb, and limited experience with PGT121 mAb. The safety, pharmacokinetics and antiviral effects of PGT121 mAb alone are being evaluated in the ongoing T001 study (IND 126807, NCT02960581). Several other HIV monoclonal antibodies are currently in clinical development as passive HIV immunoprophylaxis, or as potential therapeutics (10-1074, 3BNC117, VRC01, VRC01LS). Published data from phase 1 studies shows acceptable preliminary safety and tolerability profiles for these products, and similar anti-viral effects and pharmacokinetics (Caskey, Klein et al. 2015, Ledgerwood, Coates et al. 2015, Lynch, Boritz et al. 2015, Bar, Sneller et al. 2016, Scheid, Horwitz et al. 2016, Schoofs, Klein et al. 2016, Caskey, Schoofs et al. 2017). A comprehensive summary of phase 1 studies of HIV monoclonal antibodies can be found in the Investigator's Brochure.

3.0 STUDY OBJECTIVES

3.1 Primary Objectives

- To evaluate the safety and tolerability of IV infusion of PGDM1400 mAb alone, and a combination of PGDM1400 mAb and PGT121 mAb, in HIV-uninfected adults and HIV-infected adults
- To evaluate the pharmacokinetic (PK) profile of IV infusion of PGDM1400 mAb alone, and a combination of PGDM1400 mAb and PGT121 mAb, in HIV-uninfected adults and HIV-infected adults
- To evaluate the antiviral activity of IV infusion of PGDM1400 mAb alone, and a combination of PGDM1400 and PGT121 mAb, in HIV-infected adults not on ART

3.2 Secondary Objectives

- To determine if PGDM1400 mAb alone, and in combination with PGT121 mAb, induces anti-PGDM1400 and/or anti-PGT121 antibodies
- To determine the effect of PGDM1400 mAb alone, and in combination with PGT121 mAb, on CD4+ T cell counts in HIV-infected adults
- To determine the effect of PGDM1400 mAb alone, and in combination with PGT121 mAb, on viral escape in viremic HIV-infected adults not on ART

3.3 Exploratory Objectives

- To determine if PGDM1400 ± PGT121 mAb has any impact on the host immune responses (i.e., HIV-specific cellular and humoral immune response).
- To determine the effect of PGDM1400 ± PGT121 mAb on the size of the latent HIV reservoir in HIV-infected adults.
- To determine PGDM1400 mAb ± PGT121 mAb levels in mucosal secretions in HIV-uninfected and HIV-infected adults.
- To measure in vitro neutralization of HIV isolates with participant's serum post PGDM1400 ± PGT121 mAb IV infusion.

4.0 STUDY ENDPOINTS

4.1 Primary Endpoints

Safety and Tolerability

1. Proportion of participants with moderate or greater reactogenicity (e.g., solicited adverse events) for 3 days following IV infusion of PGDM1400 mAb alone, and a combination of PGDM1400 mAb and PGT121 mAb.
2. Proportion of participants with adverse events (AEs), including safety laboratory (biochemical, hematological) parameters, during the first 56 days following IV infusion of PGDM1400 mAb alone and a combination of PGDM1400 mAb and PGT121 mAb, that are moderate or greater, and/or related to PGDM1400 mAb or PGT121 mAb.
3. Proportion of participants with serious adverse events (SAEs) throughout the study period following IV infusion of PGDM1400 mAb alone and a combination of PGDM1400 mAb and PGT121 mAb, that are related to PGDM1400 mAb or PGT121 mAb.

Pharmacokinetics
Pharmacokinetics following IV infusion of PGDM1400 mAb alone or a combination of PGDM1400 mAb and PGT121 mAb in HIV-uninfected and HIV-infected adults:
1. Elimination half-life (t1/2)
2. Clearance (CL/F)
3. Volume of distribution (Vz/F)
4. Area under the concentration decay curve (AUC)
5. Impact of viral load and/or ART on PGDM1400 mAb and PGT121 mAb disposition (elimination half-life (t1/2), clearance (CL/F), volume of distribution (Vz/F), total exposure)

Antiviral activity
Antiviral activity following IV infusion of PGDM1400 mAb alone or in combination with PGT121 mAb in viremic HIV-infected adults not on ART:
1. Change in plasma HIV-1 RNA levels from baseline (mean of pre-entry and entry values)

4.2 Secondary Endpoints

Anti-PGDM1400 and anti-PGT121 antibodies
1. Serum anti-PGDM1400 antibody titers
2. Serum anti-PGT121 antibody titers

CD4+ T cell count
We will calculate the following endpoint to determine if IV infusion of PGDM1400 mAb alone or in combination with PGT121 mAb has any impact on CD4+ T cell counts in HIV-infected adults:
1. Change in CD4+ T cell count and frequency compared to baseline as measured by single platform flow cytometry

HIV genotyping/phenotyping of circulating virus for evaluation of PGDM1400 mAb and/or PGT121 mAb -induced escape mutations
We will compare plasma virus genotype and phenotypic activity before and after IV infusion of PGDM1400 mAb alone or in combination with PGT121 mAb to determine if PGDM1400 mAb and/or PGT121 mAb induce viral escape mutations have developed in viremic HIV-infected adults not on ART.
1. Genotypic analysis: Development of sequence variations in epitopes known to result in reduced PGDM1400 mAb and/or PGT121 mAb neutralization susceptibility or known to cause resistance to antiretroviral drugs.
2. Phenotypic analysis: Changes in viral susceptibility to PGDM1400 mAb and/or PGT121 mAb neutralization.

4.3 Exploratory Endpoints

Additional assessments may include but are not limited to the following: HIV-specific IgG/IgA binding responses by ELISA, HIV-specific cellular immune responses by ELISPOT, HIV-specific antibody function by ADCC, ADCP, and ADCVI assays, PGDM1400 mAb and/or PGT121 mAb levels in mucosal secretions, changes in total HIV-1 DNA and 2-long terminal repeat (LTR) circular HIV-1 DNA in resting or total CD4 T cells and in vitro neutralization of HIV isolates with participant's serum post IV infusion of PGDM1400 mAb ± PGT121 mAb.

5.0 STUDY DESIGN

The study is a double-blind, randomized, placebo-controlled study for Groups 1 and 2, and open label for Group 3 who will not receive placebo. A single intravenous (IV) infusion of PGDM1400 mAb alone at 3, 10 or 30 mg/kg, or a combination of PGDM1400 mAb and PGT121 mAb at 3, 10 or 30 mg/kg each, or placebo will be administered to participants. See study Table 5.0.1 for details.

Table 5.0.1 Study Design Table

	Group	Participants	Sub-Group	Regimen	N	Dose (mg/kg)
Part 1 – MTD	1	HIV-uninfected participants	1A	PGDM1400/Placebo	3/1 (6/2 if DLT)	3 IV
			1B	PGDM1400/Placebo	3/1 (6/2 if DLT)	10 IV
			1C	PGDM1400/Placebo	3/1 (6/2 if DLT)	30 IV
				Total Group 1	9/3 = 12 (max 18/6 = 24 if DLT)	
	2	HIV-uninfected participants	2A	PGDM1400 + PGT121/Placebo	3/1 (6/2 if DLT)	3 + 3 IV
			2B	PGDM1400 + PGT121/Placebo	3/1 (6/2 if DLT)	10 + 10 IV
			2C	PGDM1400 + PGT121/Placebo	3/1 (6/2 if DLT)	30 + 30 IV
				Total Group 2	9/3 = 12 (max 18/6 = 24 if DLT)	
				Total Groups 1 and 2	18/6 = 24 (max 36/12 = 48 if DLT)	
Safety Monitoring Committee						
Part 2 – antiviral effect	3	HIV-infected off ART (VL $1x10^3$ – $1x10^5$ copies/ml)	3A	PGDM1400	6 (max 18)	MTD IV
			3B	PGDM1400 + PGT121	6 (max 18)	MTD IV
				Total Group 3	12 (max 36)	
				Total entire study	36 (max 84)	

DLT, dose limiting toxicity; MTD, maximum tolerated dose

5.1 Definition of Dose Limiting Toxicity- Groups 1 and 2

Dose limiting toxicity (DLT) will be defined as 1) any Grade 3 or greater adverse event considered possibly, probably or definitely related to investigational product or 2) any Grade 3 or greater reactogenicity or 3) any SAE considered possibly, probably or definitely related to investigational product.

5.2 Definition of Maximum Tolerated Dose- Groups 1 and 2

If 2 or more DLTs occur in a dose Subgroup (e.g., 1B) that are the same, similar, or in the same System Organ Class, infusion will be halted and the next lower dose level will be declared the maximum tolerated dose (MTD) within this Group. If no DLT occurs in the final dose Subgroup (1C and 2C), MTD will be the highest dose given (30mg/kg) after 14 days of follow-up.

5.3 Dose Escalation and Determination of Maximum Tolerated Dose – Groups 1 and 2

Part 1 of this study is a dose-escalation trial in HIV-uninfected adults (Groups 1 and 2) to establish the MTD of PGDM1400 mAb alone and a combination of PGDM1400 mAb and

PGT121 mAb. There will be sentinel participants in each dose Subgroup, and safety data will be reviewed to determine if dose escalation can proceed.

Sentinel participants in Groups 1 and 2

For each dose Subgroup in Groups 1 and 2, the 1st 2 participants will be sentinel participants for whom investigational product infusion will be separated by at least 24 hours contingent on review of safety information prior to infusion of the next sentinel participant. If no reactogenicity or adverse events that meet the DLT criteria occur within 24 hours after IV infusion of the 1st participant, the 2nd participant may be infused with investigational product. If no events meeting the DLT criteria occur within 24 hours after the 2nd participant is infused, then the remaining participants in that dose Subgroup will be infused. If events meeting the DLT criteria do occur for the first 2 participants in a dose Subgroup, the data will be reviewed by the Safety Monitoring Committee (SMC) to determine whether further infusions may proceed.

Staggered dose escalation design whereby PGDM1400 alone and PGT121 alone safety data are reviewed before combined infusion of PGDM1400 and PGT121 at the same dose

The combination Groups, starting with PGDM1400 3 mg/kg + PGT121 3 mg/kg can only start when 1) the PSRT has reviewed the safety data through day 14 post investigational product infusion from PGDM1400 alone at 3 mg/kg and has approved dose escalation to 10 mg/kg AND 2) the MTD of PGT121 as determined in the T001 study is 3 mg/kg or higher. Dosing levels of PGDM1400 mAb and PGT121 in the combination Subgroups can never exceed the MTDs for PGDM1400 and PGT121 as determined in the PGDM1400 alone Group 1 in this study, and the PGT121 alone Group in the T001 study.

PSRT review for dose escalation

The Protocol Safety Review Team (PSRT) will review safety data through day 14 post-investigational product infusion for all participants in each dose Subgroup for PGDM1400 mAb alone (Group 1) prior to allowing enrolment of participants into the next higher dose Subgroup in Group 1 or the same dose level PGDM1400 mAb and PGT121 mAb combination dose Subgroup (Group 2).

- If no DLT occurs in the initial 4 participants of a dose Subgroup (e.g., 1A), the study can proceed with enrolment of the next dose Subgroup for PGDM1400 mAb alone (e.g., 1B), and the same dose level PGDM1400 mAb and PGT121 mAb combination dose Subgroup (e.g., 2A).
- If 1 DLT occurs in the initial 4 participants of a dose Subgroup (e.g., 1A), 4 additional participants will be enrolled in the same dose Subgroup.
    - If no additional DLTs occur within 14 days of infusion in the 8 total participants, the study can proceed with enrolment of the next dose Subgroup for PGDM1400 mAb alone (e.g., 1B), and the same dose level PGDM1400 mAb and PGT121 mAb combination dose Subgroup (e.g., 2A).
    - If 2 or more DLTs accumulate in a dose Subgroup (e.g., 1B) that are the same, similar, or in the same System Organ Class, infusion will be halted and the next lower dose level will be declared the maximum tolerated dose (MTD) within this Group.
    - If no DLT occurs in the final dose Subgroup (e.g.,1C), the MTD for PGDM1400 alone will be the highest dose given (30mg/kg) after 14 days of follow-up.

- Dosing levels of PGDM1400 mAb and PGT121 in the combination Subgroups can never exceed the MTDs for PGDM1400 and PGT121 as determined in the PGDM1400 alone Group 1 in this study, and the PGT121 alone Group in the T001 study.

SMC review to determine MTD of PGDM1400 alone
Following IV infusion of of investigational product in the last participant in Group 1 , an independent Safety Monitoring Committee (SMC) will review safety data through day 14 post-investigational product infusion for all participants to confirm MTD of PGDM1400 alone, and determine whether, and at what dose, Group 3A can initiate enrolment.

SMC review to determine MTD of the combination of PGDM1400 and PGT121
Following IV infusion of investigational product in the last participant in Group 2, the SMC will review safety data through day 14 post-investigational product infusion for all participants to confirm MTD of the combination of PGDM1400 and PGT121, and determine whether Group 3B can initiate enrollment.

5.4 Determination of Antiviral Effect – Group 3

Part 2 of this study will establish the antiviral effect of PGDM1400 mAb alone or the combination of PGDM1400 mAb plus PGT121 mAb in HIV infected adults not on ART (Group 3).

MTD determines dose
Group 3 will start with the MTD of PGDM1400 as determined by the SMC; e.g., if the MTD for PGDM1400 is 30mg/kg then Subgroup 3A will receive 30mg/kg and Subgroup 3B will receive PGDM1400 mAb + PGT121 mAb at a dose that combines the MTD for PGDM1400 mAb and the MTD for PGT121 mAb (as determined in the ongoing T001 study IND 126807, NCT02960581).

5.5 Duration of the Study

Up to 32 weeks per participant, screening up to 42 (HIV-infected) or up to 56 (HIV-uninfected) days before single IV infusion of investigational product on day 0, and 24 weeks of follow up.

It will take approximately 11 months to enroll the entire study. The exact duration depends on the recruitment rate and how many participants will be required per dose Subgroup as specified in sections 5.3 and 5.4.

5.6 Study Population

The study population consists of HIV-uninfected male or female adults (Group 1 and 2) and HIV-infected males and female adults (Group 3) who meet the detailed inclusion and exclusion criteria listed below, and who in the opinion of the investigator or designee, understand the study and provide written informed consent.

Approximately 36-84 participants (72 active product recipients, 12 placebo recipients) who meet all eligibility criteria will be included in the study. An over-enrollment of up to 5% (up to 5 participants total) will be permitted in the study to facilitate rapid enrollment.

5.7 Inclusion Criteria

Inclusion criteria for all participants:

1. Willing to comply with the requirements of the protocol and available for follow-up for the planned duration of the study.

2. In the opinion of the Principal Investigator or designee and based on Assessment of Informed Consent Understanding results, has understood the information provided and potential impact and/or risks linked to IV infusion and participation in the trial; written informed consent will be obtained from the participant before any study-related procedures are performed.

3. All heterosexually active female participants must commit to use an effective method of contraception for 3 months following investigational product administration, including:

   a. Condoms (male or female) with or without spermicide

   b. Diaphragm or cervical cap with spermicide

   c. Intrauterine device, or contraceptive implant

   d. Hormonal contraception

   e. Successful vasectomy in the male partner (considered successful if a woman reports that a male partner has [1] documentation of azoospermia by microscopy (< 1 year ago), or [2] a vasectomy more than 2 years ago with no resultant pregnancy despite sexual activity post-vasectomy)

   f. Not be of reproductive potential, such as having undergone hysterectomy, bilateral oophorectomy, or tubal ligation, postmenopausal (> 45 years of age with amenorrhea for at least 2 years, or any age with amenorrhea for at least 6 months and a serum follicle stimulating hormone [FSH] level > 40 IU/L); surgically sterile: no additional contraception required.

   g. Women, who are not heterosexually active at screening, must agree to utilize an effective method of contraception if they become hetero-sexually active, as outlined above.

4. All sexually active males, regardless of reproductive potential, must be willing to consistently use an effective method of contraception (such as consistent male condoms with male and/or female partners) from the day of investigational product administration until at least 3 months following investigational product administration to avoid exposure of partners to investigational product in ejaculate, and to prevent conception with female partners.

5. All female participants must be willing to undergo urine pregnancy tests at time points indicated in the Schedule of Procedures and must test negative prior to investigational product administration.

6. A female participant must agree not to donate eggs (ova, oocytes) for the purpose of assisted reproduction until 3 months after investigational product administration. A man must agree not to donate sperm until 3 months after investigational product administration.

7. Willing to forgo donations of blood and/or any other tissues, including bone marrow, during the study and, for those HIV-uninfected participants who test HIV-positive due to investigational product administration, until the anti-HIV antibody titers become undetectable.

Specific inclusion criteria for HIV-uninfected participants (Groups 1 and 2):

8. At least 18 years of age on the day of screening and has not reached his or her 51st birthday on the day of signing the Informed Consent Document.

9. Willing to undergo HIV testing, risk reduction counselling and receive HIV test results.

10. Low risk for HIV infection and willing to maintain low-risk behavior for the duration of the trial.

11. Healthy male or female, as assessed by a medical history, physical exam, and laboratory tests.

Specific inclusion criteria for HIV-infected participants (Groups 3):

12. At least 18 years of age on the day of screening and has not reached his or her 66th birthday on the day of signing the Informed Consent Document.

13. Confirmed HIV-1 infection (HIV Ab+ or HIV RNA+) by documentation in the medical records or in-clinic HIV testing;

14. CD4 ≥ 300 cells/µl.

15. Not receiving cART, and (after appropriate counselling) willing to defer cART treatment for at least 56 days after administration of investigational product.

16. HIV-1 viral load between 1000–100,000 copies/ml, confirmed at screening.

5.8 Exclusion Criteria

Exclusion criteria for all participants:

1. Any clinically significant acute or chronic medical condition, other than HIV infection, that is considered progressive or in the opinion of the investigator makes the participant unsuitable for participation in the study.

2. If female, pregnant, lactating or planning a pregnancy during the period of screening through completion of the study.

3. In the past 6 months a history of alcohol or substance use, including marijuana, judged by the Investigator to potentially interfere with participant study compliance.

4. Bleeding disorder that was diagnosed by a physician (e.g., factor deficiency, coagulopathy or platelet disorder that requires special precautions). Note: A participant who states that he or she has easy bruising or bleeding, but does not have a formal diagnosis and has intramuscular injections and blood draws without any adverse experience, is eligible.

5. History of a splenectomy.

6. Receipt of live attenuated vaccine within the previous 30 days or planned receipt within 30 days after administration of investigational product; or receipt of other vaccine within the previous 14 days or planned receipt within 14 days after infusion with investigational product (exception is live attenuated influenza vaccine within 14 days).

7. Receipt of blood transfusion or blood-derived products within the previous 3 months.

8. Participation in another clinical trial of an investigational product currently, within the previous 3 months or expected participation during this study.

9. Prior receipt of an investigational HIV vaccine candidate, monoclonal antibody or polyclonal immunoglobulin (note: receipt of placebo in a previous HIV vaccine or monoclonal antibody trial will not exclude a participant from participation if documentation is available and the Medical Monitor gives approval).

10. History of severe local or systemic reactogenicity to injections or IV infusion (e.g., anaphylaxis, respiratory difficulties, angioedema);

11. Psychiatric condition that compromises safety of the participant and precludes compliance with the protocol. Specifically excluded are persons with psychoses within the past 3 years, ongoing risk for suicide, or history of suicide attempt or gesture within the past 3 years.

12. If, in the opinion of the Principal Investigator, it is not in the best interest of the participant to participate in the trial.

13. Seizure disorder: a participant who has had a seizure in the last 3 years is excluded. (Not excluded: a participant with a history of seizures who has neither required medications nor had a seizure for 3 years.)

14. Body mass index ≥ 35 or ≤ 18.0.

15. Infectious disease: chronic hepatitis B infection (HbsAg), current hepatitis C infection (HCV Ab positive and HCV RNA positive) or interferon-alfa treatment for chronic hepatitis C infection in the past year, or active syphilis.

16. A history of malignancy within the past 5 years (prior to screening) or ongoing malignancy;

17. Active, serious infections (other than HIV-1 infection) requiring parenteral antibiotic, antiviral or antifungal therapy within 30 days prior to enrollment.

Specific exclusion criteria for HIV-uninfected participants (Group 1 and 2):

18. Confirmed HIV-1 or HIV-2 infection.

19. Any clinically relevant abnormality on history or examination including history of immunodeficiency or autoimmune disease; use of systemic corticosteroids, immunosuppressive, anticancer, or other medications considered significant by the investigator within the previous 6 months.

    The following exceptions are permitted and will not exclude study participation: use of corticosteroid nasal spray for rhinitis, topical corticosteroids for an acute uncomplicated dermatitis; or a short course (duration of 10 days or less, or a single injection) of corticosteroid for a non-chronic condition (based on investigator clinical judgment) at least 6 weeks prior to enrollment in this study.

20. Any of the following abnormal laboratory parameters listed below:

    <u>Hematology</u>
    Hemoglobin < 10.5 g/dL in females; hemoglobin <11.0 g/dL in males
    Absolute Neutrophil Count (ANC): ≤ 1000/mm3
    Absolute Lymphocyte Count (ALC): < 650/mm3
    Platelets: < 125,000 mm3 or ≥ 550,000/mm3
    <u>Coagulation</u>
    aPTT: >1.25 x ULN
    INR: ≥1.1 x ULN
    <u>Chemistry</u>
    - Sodium ≤ 135 mEq/L or ≥ 146 mEq/L
    - Potassium ≤ 3.4 mEq/L or ≥ 5.6 mEq/L
    - Creatinine ≥ 1.1 x ULN
    - AST ≥ 1.25 x ULN
    - ALT ≥ 1.25 x ULN
    - Total bilirubin ≥ 1.25 x ULN
    - Alkaline phosphatase ≥ 1.25 x ULN
    - Albumin ≤ 3.0 g/dL or ≤ 30 g/L
    - Creatine kinase ≥ 3.0 x ULN
    - C-reactive protein > 10 mg/L
    - C3 complement < 82 mg/dL
    - C4 complement < 14 mg/dL

    <u>Urinalysis</u>
    Any of the following abnormal findings if consistent with clinically significant disease:
    - Protein = greater than trace on dipstick confirmed by microscopic urinalysis outside institutional range.
    - Blood = greater than trace on dipstick confirmed by >3 RBCs/hpf on microscopic urinalysis (not due to menses).

Specific exclusion criteria for HIV-infected participants who are not on ART (Group 3):

21. Any clinically relevant abnormality on history or examination including history of immunodeficiency or autoimmune disease, other than HIV; use of systemic

corticosteroids, immunosuppressive, anticancer, or other medications considered significant by the investigator within the previous 6 months.

The following exceptions are permitted and will not exclude study participation: use of corticosteroid nasal spray for rhinitis, topical corticosteroids for an acute uncomplicated dermatitis; or a short course (duration of 10 days or less, or a single injection) of corticosteroid for a non-chronic condition (based on investigator clinical judgment) at least 6 weeks prior to enrollment in this study.

22. Any of the following abnormal laboratory parameters listed below:

<u>Hematology</u>
- Hemoglobin < 10.0 g/dL
- Absolute Neutrophil Count (ANC): < 1000 cells/mm3
- Platelets: < 100,000 cells/mm3

<u>Coagulation</u>
- aPTT: > 1.25 x ULN
- INR: ≥ 1.1 x ULN

<u>Chemistry</u>
- Estimated Glomerular filtration rate (GFR) < 80 mL/min according to the Cockcroft Gault formula for creatinine clearance
  - Male: (140 – age in years) x (wt in kg) = CLcr (mL/min) / 72 x (serum creatinine in mg/dL)
  - Female: (140 – age in years) x (wt in kg) x 0.85 = CLcr (mL/min) / 72 x (serum creatinine in mg/dL)
- AST ≥ 2.5 x ULN
- ALT ≥ 2.5 x ULN
- Total bilirubin ≥ 1.6 x ULN
- Alkaline phosphatase ≥ 5 x ULN

<u>Urinalysis</u>

Any of the following abnormal findings if consistent with clinically significant disease:
- Protein = greater than 1+ on dipstick confirmed by microscopic urinalysis outside institutional range.
- Blood = greater than 1+ on dipstick confirmed by > 10 RBCs/hpf on microscopic urinalysis (not due to menses).
- Leukocytes = greater than 1+ on dipstick confirmed by > 10 WBC/hpf on microscopic urinalysis.

5.9 Recruitment of Participants

Adult male and female participants may be recruited through in-clinic referrals, information presented to community organizations, hospitals, colleges, other institutions and/or advertisements to the general public or from existing cohorts. The information distributed will contain contact details of the trial site.

6.0 STUDY VISITS

6.1 Screening Period

During Screening, study staff will perform the following procedures:

- Provide and/or review the Informed Consent Document and answer any questions about the study prior to obtaining written informed consent.
- Complete Assessment of Informed Consent Understanding (AOU). Please refer to the Study Operations Manual (SOM)

If the participant agrees to participate, passes the AOU and provides written informed consent, study staff will:

- Conduct HIV test counselling, HIV testing, and HIV risk reduction counselling, as applicable
- Conduct family planning counselling, refer for pregnancy prevention counselling if necessary
- Administer HIV risk assessment (Group 1 and 2)
- Conduct ART counselling (Group 3)
- Perform a comprehensive medical history
- Collect concomitant medication information
- Perform a general physical examination (Refer to Section 7.2)
- Collect specimens for all tests as indicated in the Schedule of Procedures in Appendices A, B and C (for details see Analytical Plan (AP)).

When available, the screening laboratory tests will be reviewed by the trial physician. Screening laboratory test(s) may be repeated once at the discretion of the principal investigator or designee to investigate any isolated abnormalities.

If the screening visit occurs outside the allowable screening window, all screening procedures must be repeated except the comprehensive medical history may be replaced by an interim medical history and the Participant Information Sheet of the Informed Consent Document should be reviewed.

If a participant has signed the Consent Form but does not meet the eligibility criteria, the records must be kept at the site.

6.2 IV infusion of Investigational Product Visit

Prior to the infusion of investigational product, study staff will:

- Answer any questions the participant may have about the study
- Review the Informed Consent Document with the participant
- Review screening safety laboratory data
- Conduct HIV test counselling, and HIV risk reduction counselling , as applicable
- Conduct ART counselling (Group 3)
- Conduct family planning counselling as per site specific procedures and ensure compliance with respective pregnancy prevention method, and discuss male condom use with all male participants

- Review interim medical history
- Collect concomitant medication information
- Weigh participant and record vital signs
- Perform a symptom-directed physical examination (Refer to Section 7.2)
- Assess at baseline local and systemic signs and symptoms (this includes an examination of IV infusion site)
- Collect specimens for all tests as indicated in the Schedule of Procedures see Appendices A, B and C (for details see AP).
- Obtain pregnancy test results prior to infusion of investigational product.
- Assign an allocation number to the participant according to the instructions specified in the Study Operations Manual.

If a participant has an abnormal laboratory value that is known, at the time of infusion, follow the specified guidelines (Section 12.0).

At the time of infusion of investigational product and after IV infusion of investigational product, study staff will:

- Administer the investigational product as specified in Section 8.4, Administration of Investigational Product and according to the instructions specified in the SOM.
- Observe participant closely during the infusion of investigational product and for at least 30 minutes after IV infusion of investigational product has ended for any acute reactogenicity.
- Every 30 minutes after IV infusion of investigational product, for the 1st 4 hours, and every hour thereafter through 6 hours post-infusion, the study staff will:
  - Record vital signs (pulse, respiratory rate, blood pressure and temperature)
  - Assess any local and systemic reactogenicity
  - Assess any other adverse events
- Collect PK samples according to the Schedule of Procedures

6.3 Post-IV infusion of Investigational Product Visits

The participant will be asked to return to the clinic for post-investigational product infusion visits as indicated in the schedule of procedures (see appendices A, B and C) for an assessment by clinic staff. The participant will be asked to maintain a Memory Aid to track any local and systemic reactogenicity the participant experiences, including temperature, from the day of investigational product infusion for the next 3 days (for a total of 4 days including day of investigational product infusion). Participants will receive a thermometer to take their oral temperature at home once a day. Study staff will review the Memory Aid with the participant and determine the severity of the reactions through discussion with the participant.

The following procedures will be conducted at these visits:

- Review interim medical history
- Collect concomitant medication information
- Perform a symptom-directed physical examination if any signs or symptoms are present
- Assess vital signs (pulse, respiratory rate, blood pressure and temperature)

- Assess any adverse events and local and systemic reactogenicity (Days 1, 2, 3) including reviewing the Memory Aid.
- Collect specimens for all tests as indicated in the Schedule of Procedures (Appendices A, B and C and AP).

6.4 Additional Follow-up Visits

Assessments and procedures will be performed according to the Schedule of Procedures (Appendices A, B and C).

6.5 Unscheduled Visits

Unscheduled Visits/Contacts are visits/contacts that are not described in the Schedule of Procedures (Appendices A, B and C). Unscheduled visits may occur any time during the study:

- For administrative reasons, e.g., the participant may have questions for study staff or may need to re-schedule a follow-up visit.
- To obtain laboratory test results from a previous visit.
- For other reasons as requested by the participant or site investigator.

All unscheduled visits will be documented in the participants' study records on applicable source documents and entered into the Case Report Form (CRF).

6.6 Final Study Visit or Early Termination Visit

Assessments and procedures will be performed according to the Schedule of Procedures (Appendices A, B and C).

7.0 STUDY PROCEDURES

7.1 Informed Consent Process

A Master Informed Consent Document consisting of a Participant Information Sheet and a Consent Form is provided by the Sponsor to the trial site. This document is made site-specific and translated (if necessary), submitted and approved by the Institutional Review Board (IRB). The Master and site specific Informed Consent Documents are separate documents and should not be part of the protocol.

Participant Information Sheet
A qualified member of the study staff will conduct the informed consent process by reviewing the Participant Information Sheet and document in the clinic notes.

Consent Form
The participant's consent to participate must be obtained by him/her signing and dating the Consent Form. The person obtaining consent will also sign.

The signed and dated Informed Consent Document must remain at the study site. A copy of the signed/marked and dated Informed Consent Document will be offered to the participant to take home. Those participants who do not wish to take a copy will be required to document that they declined to do so.

7.2 Medical History and Physical Examination

Medical History
At screening, a comprehensive medical history will be collected including previous IV infusions and reaction to IV infusion, history of sexually transmitted infection (STI) and pregnancy prevention practices. At subsequent visits, an interim medical history will be performed.

Physical Examination

General Physical Examination
A general physical examination includes examination of head/ears/eyes/nose and throat, skin, respiratory, cardiovascular, abdominal, limited neurological and musculoskeletal and external ano-genital systems (for HIV-infected participants only) at the time points indicated in the Schedule of Procedures (see Appendices A, B and C).

Symptom-Directed Physical Examination
A symptom-directed physical examination is a targeted examination based on the participant's history or observation. If deemed necessary, this examination should be done at the time points indicated in the schedule of procedures (see Appendices A, B and C).

Measuring Height and Weight
Includes measuring the height and weight at the time points indicated in the Schedule of Procedures (see Appendices A, B and C).

Vital Signs

Vital signs including pulse, respiratory rate, blood pressure and temperature are measured and recorded at the time points indicated in the Schedule of Procedures (see Appendices A, B and C)

7.3 HIV Testing and HIV-test Counselling (Groups 1 and 2)

Study staff will perform pre-HIV test counselling prior to collecting blood for an HIV test, and post-HIV test counselling when HIV test results are available. This is referred to as HIV-test counselling, and done according to the CDC guidelines. For more information on HIV testing and HIV-test counselling, see Section 11.0. A screening questionnaire and other tools may be used.

7.4 HIV Risk Reduction Counselling

HIV risk reduction counselling will be provided to all participants as outlined by site-specific SOPs.

Study staff will provide HIV risk reduction counselling based on reported individual risk and provide free condoms, as appropriate, at every visit. Group 1 and 2 will receive HIV risk reduction counselling and for Groups 3, HIV risk reduction counselling will be conducted as secondary prevention to reduce onward transmission.

7.5 Family Planning Counselling

Study staff will counsel participants about the importance of preventing pregnancies and of using condoms, as well as other effective family planning methods until at least 3 months following investigational product administration, as appropriate. Participants may be referred for family planning services as necessary according to site-specific SOPs as detailed in the SOM. Pregnancy prevention methods chosen and compliance will be documented.

7.6 ART Counselling (Group 3)

HIV-infected participants who are not on ART will receive ART counselling upon entering the study and 8 weeks after infusion of investigational product. Participants who have not initiated or made plans to initiate ART by the final study visit will receive ART counselling again at their final study visit.

HIV-infected participants who are not on ART who achieve an undetectable HIV RNA level after IV infusion of PGDM1400 ± PGT121 may be asked to postpone initiation of ART until HIV RNA becomes detectable (see section 2.1.3.).

7.7 Specimens

Approximately 50 ml of blood will be collected from participants in Group 1 and 2, and approximately 150 ml of blood will be collected from participants in Group 3 at the screening visit. At later visits, approximately 8.5 ml to 175 ml of blood will be collected, depending on study procedures and Group assignment (see Appendices A, B and C), usually from the antecubital fossa.

Optional collection of rectal and/or cervical mucosal secretions will be obtained using a rectal sponge (or comparable swab) or cervical Softcup (or comparable cervical fluid collection cup) for those participants that consent.

All specimens will be handled according to the procedures specified in the AP and relevant SOPs if applicable.

In the event of an abnormal laboratory value, participants may be asked to have an additional sample collected at the discretion of the Principal Investigator or designee.

7.8 Reimbursement

Participants will be reimbursed for their time, effort and for costs to cover their travel expenses to the study site and any inconvenience caused due to study participation. Site specific-reimbursement amounts will be documented in the site-specific Participant Information Sheet, and approved by the Institutional Review Board.

7.9 Randomization and Blinding

Participants will be identified by a unique study identification number.

Participants will be randomized according to the randomization schedule prepared by the statisticians at the Data Coordinating Center (DCC) prior to the start of the study. Participants will be automatically assigned a specific allocation number as they are enrolled into the data entry system. An unblinding list (Pharmacy List) will be provided to the unblinded site pharmacist by the DCC.

This a double blind, randomized, placebo-controlled study for Groups 1 and 2, and an open label study for Group 3. For Groups 1 and 2, study staff (investigator and clinical personnel monitoring the safety and laboratory assay results) and participants will be blinded with respect to the allocation of Investigational Product (PGDM1400 mAb alone or PGDM1400 and PGT121 in combination or placebo). A site pharmacist will be unblinded for the purposes of preparing investigational product.

A participant will be considered enrolled once he/she has been assigned an allocation number.

Blinded participants will be informed about their assignment (active product/placebo) at study completion, once the database is locked. Should a study participant be unblinded during the study, the study participant will be followed up until the end of the study according to the Schedule of Procedures (Appendices A and B).

7.10 Un-blinding Procedure for Individual Participants

Un-blinding of an individual participant may be indicated in the event of a medical emergency if the clinical management of the participant would be altered by knowledge of the treatment assignment.

The un-blinded information should be restricted to a small Group of individuals involved in clinical management/medical treatment of the participant (e.g., treating physician) and the blind must be maintained for those responsible for the study assessments.

The reasons for un-blinding should be documented and the IAVI Chief Medical Officer, the Medical Monitor and the DCC should be notified as soon as possible. The procedures and contact numbers for un-blinding are outlined in the SOM.

7.11 Assessment of PGDM1400 mAb and/or PGT121 mAb related HIV sero-positivity

It is possible that PGDM1400 and/or PGT121 or an immune response to PGDM1400 and/or PGT121 could cause a positive result on a diagnostic HIV antigen/antibody test. A Group 1 or 2 participant who tests HIV antigen and/or HIV antibody positive at the end of the study will have additional testing to distinguish actual HIV infection from investigational product-related responses. The participant will be informed of his/her positive HIV antigen and/or HIV antibody test result and offered continuing follow-up until the HIV antigen/antibody test becomes negative.

8.0 INVESTIGATIONAL PRODUCT

8.1 Description

PGDM1400 and PGT121 active products are formulated in a 20 mM Acetate, 9% Sucrose, 0.008% polysorbate 80, pH 5.2 formulation buffer at a concentration of 50 mg/mL. Each 10 ml vial contains 6 ml of PGDM1400 or PGT121.

A summary of the Investigational Products and example volumes needed for administration for each dose level are shown in Table 8.1-1.

Table 8.1-1 Investigational Products

Active Product / Placebo	Dosage level	Total volume in investigational product container	Total Active Product or placebo volume to be injected into a 100 mL saline IV bag (for an 88 kg body weight**)	Total volume to be Infused (for an 88 kg body weight**)
PGDM1400 (50 mg/mL)	3 mg/kg	6 mL per vial	5.3 mL	105.3 mL
	10 mg/kg		17.6 mL	117.6 mL
	30 mg/kg		52.8 mL	152.8 mL
PGT121 (50 mg/mL)	3 mg/kg	6 mL per vial	5.3 mL	105.3 mL
	10 mg/kg		17.6 mL	117.6 mL
	30 mg/kg		52.8 mL	152.8 mL
Placebo: 0.9% Sodium Chloride for Injection USP (Saline)*	3 mg/kg matching***	NA	5.3 mL***	105.3 mL***
	10 mg/kg matching***		17.6 mL***	117.6 mL***
	30 mg/kg matching***		52.8 mL***	152.8 mL***

* The Placebo provided will be a commercially-available 0.9% sodium chloride for injection USP partial addition IV bag.

** The actual volume to be injected will be based on the dose Subgroup and the weight of the participant at the time of investigational product administration. The example included here is the average weight of an adult male in the US (88kg) (http://www.cdc.gov/nchs/data/series/sr_11/sr11_252.pdf)

*** For placebo IV infusions: saline from an additional IV bag will be injected into the saline IV bag intended for administration, to match the volume used for a PGDM1400 ± PGT121 mAb injection

in the same dose Subgroup, to prevent unblinding. Placebo recipients in Group 2 will receive 2 sequential administrations of placebo to mimic administration of PGDM1400 followed by PGT121 to maintain blinding. See SOM for details.

8.2 Shipment and Storage

Authorization to ship the PGDM1400 and PGT121 to the site will be provided in writing by the Sponsor, upon confirmation that all required critical documents for shipment authorization are completed. PGDM1400 and PGT121 will be shipped maintaining the required storage conditions and stored in a secure location in the clinical site's pharmacy.

PGDM1400 and PGT121 vials will be stored at - 20°C ± 5°C. Each vial will be labelled with the name of the product, lot number, concentration, fill volume, storage temperature, date of manufacture, name and location of the manufacturer and a US cautionary statement. Several such vials will be packaged in a box. Each box will be labelled with similar information as the vial label, including an address and contact information for the manufacturer.

0.9% Sodium Chloride for Injection USP, in partial-addition bags, will be used as the placebo and diluent for PGDM1400 and PGT121. It will be stored at room temperature.

8.3 Preparation of investigational product

Detailed instructions will be provided to the site pharmacist in the SOM for preparing each of the investigational products. The site pharmacist will not be blinded, but the study physician/designee administering the investigational product will be blinded. Infusion of the investigational product(s) should be completed within 4 hours of the PGDM1400 or PGT121 being injected into the saline IV bag. Example calculations for the volumes needed for IV infusion are illustrated in Table 8.1-1. Procedures for handling used and partially used vials of PGDM1400 and PGT121 will be provided in the SOM. Syringes or other components in direct contact with PGDM1400 or PGT121 will be disposed of properly in a biohazard container and incinerated or autoclaved as per site procedure.

8.4 Administration of investigational product

Investigational product will be administered at the enrollment visit. The investigational product will be injected into a 0.9% sodium chloride for injection USP partial addition bag. The participant will receive the investigational product via IV infusion. If more than one investigational product will be administered, these will be administered sequentially in separate saline IV bags. Participants will receive each infusion over approximately 60 minutes, allowing for clinician discretion. Further information on the IV infusion of the investigational product is supplied in the SOM and other study documents.

8.5 Accountability and Disposal of investigational product

All used PGDM1400 and PGT121 vials will be handled according to instructions in the SOM. Throughout the study, the investigational product accountability forms including receipt and dispensing of vials will be kept and monitored. The vial label for the used vial(s) will be removed and retained for accountability purposes; the used vial(s) can be discarded as per site procedures.

At the end of the study, the unused PGDM1400 and PGT121 vials will be reconciled according to Sponsor instructions.

Further information on accountability and disposal of PGDM1400 and PGT121 is supplied in the SOM.

9.0 ASSESSMENTS

9.1 Safety Assessments

Data on local and systemic reactogenicity (i.e., solicited AEs) will be collected by structured interview and medical examination. Data on other adverse events will be collected with open-ended questions. All data will be recorded on the appropriate source documents and entered into the study database. Participants will be given a Memory Aid, which is a tool to assist with collecting reactogenicity data.

Local and systemic reactogenicity events will be assessed by study staff prior to and during IV infusion of investigational product, approximately every 30 minutes after investigational product infusion for the first 4 hours after investigational product infusion and subsequently every hour for the first 6 hours post-investigational product infusion. Study staff will review the Memory Aid with the participant, and determine the severity of the reactions on days 1-3 through discussion with the participant.

9.1.1 Local reactogenicity

The presence of local reactogenicity will be assessed at the time points specified in the Schedule of Procedures (Appendices A, B and C).
Pain, tenderness, erythema/skin discoloration, swelling/hardening or pruritus will be assessed and graded using Appendix G, DAIDS Adverse Event Severity Assessment Table, as a guideline.

9.1.2 Systemic reactogenicity

The presence of systemic reactogenicity will be assessed at the time points specified in the Schedule of Procedures (Appendices A, B and C).
Fever, chills, headache, nausea, vomiting, malaise, myalgia and arthralgia will be assessed and graded using the Appendix G DAIDS Adverse Event Severity Assessment Table as a guideline.
For the first 24 hours after investigational product infusion, any infusion related reactions, including cytokine release syndrome, should be graded using the Common Terminology Criteria for Adverse Events (CTCAE) v4.03: June 14, 2010 (Appendix F).

9.1.3 Vital Signs

At the infusion visit, vital signs (pulse, respiratory rate, blood pressure and temperature) will be measured by study staff prior to investigational product infusion, approximately every 30 minutes for the first 4 hours post investigational product infusion and then hourly until 6 hours after IV infusion. For the other study visits vital signs will be assessed at the time points specified in the Schedule of Procedures (Appendices A, B and C).

9.1.4 Other Adverse Events

Other adverse events (AEs) will be collected through 56 days after investigational product infusion in all participants. Serious Adverse Events (SAEs) will be collected throughout the entire study period. Potential Immune Mediated Diseases (pIMDs), as defined in Section 10.5, will be collected throughout the study period, using the SAE reporting process. Open-ended questions will be asked at time points according to the Schedule of Procedures (Appendices A, B and C). All adverse events during the first 24 hours after the infusion will be graded using Appendix G, DAIDS Adverse Event Severity Assessment Table, as a guideline and will be assessed for relatedness to the investigational product. For more information regarding adverse events refer to Section 10.0, Adverse Events.

9.1.5 Concomitant Medications

Concomitant receipt of Investigational Products is prohibited during the study.

Contraceptive use and use of medication at study entry will be documented. (See DCF instructions).

During the study, information regarding concomitant medications and reasons for their use will be solicited from the study participants for 56 days. Ongoing concomitant medications will be recorded until end of study.

9.1.6 Routine laboratory parameters

Table 9.1.6-1 shows the laboratory parameters that will be measured routinely. The samples for these tests will be collected at the time points indicated in the Schedule of Procedures (Appendices A, B and C).

Table 9.1.6-1: Laboratory Parameters

Laboratory Parameter	Test
Hematology and Coagulation	Hemoglobin, hematocrit, leukocytes, platelets, absolute neutrophil count (ANC), absolute lymphocyte count (ALC), activate partial thromboplastin time (aPTT), international normalized ratio (INR)
Clinical Chemistry	Sodium, potassium, creatinine, aspartate aminotransferase (AST), alanine aminotransferase (ALT), total bilirubin, alkaline phosphatase<br>Groups 1 and 2 only: albumin, creatine kinase, C-reactive protein, C3 complement, C4 complement
Urinalysis	Dipstick test for protein, blood glucose, ketones, esterase (leukocytes) and nitrite. If clinically significant abnormalities (e.g., blood, protein, leukocytes) are found on dipstick test, then further test(s) will be performed (e.g., microscopy, culture)
T cell panel (Group 3)	CD4 T cell count and frequency by single platform flow cytometry

9.1.7 Specific screening tests:

Participants will be screened to exclude the following diseases:

- Hepatitis B: positive for hepatitis B surface antigen (HBsAg)
- Hepatitis C: positive for hepatitis C RNA (HCV antibody test, followed by HCV RNA test if HCV antibody positive)
- Active syphilis: confirmed diagnosis.

A negative Hepatitis B and Hepatitis C result can be documented from the medical record only if the result is from a test administered less than 6 months ago.

9.1.8 Monitoring for anti-PGDM1400 and anti-PGT121 antibodies:

Participants will be evaluated for the development of antibodies to PGDM1400 and PGT121 mAb (anti-drug antibodies, ADA) by ELISA according to the Schedule of Procedures (Appendices A, B and C).

9.2 Virologic Assessments

Table 9.2-1 shows the virologic parameters that will be measured routinely. The samples for these tests will be collected at the time points indicated in the Schedule of Procedures (Appendix C).

Table 9.2-1: Virologic Assessment Table

Virologic Parameter	Test
Antiviral Activity	Plasma HIV RNA levels
Anti-reservoir activity	Cell-associated HIV-1 RNA levels in resting CD4 T cells; total HIV-1 DNA and 2-long terminal repeat (LTR) HIV-1 DNA circles in resting or total CD4 T cells; quantitative viral outgrowth assay (qVOA)
Other	Genotyping of plasma HIV RNA for evaluation of PGDM1400 and PGT121-induced escape mutations and resistance to antiretroviral drugs; phenotyping of plasma HIV RNA for neutralization susceptibility to PGDM1400 and PGT121 in-vitro

9.3 Exploratory Immunogenicity Assessments

Humoral immune response assays will include, but are not limited to Env-specific Ab-binding assays, virus neutralization assay, and assays for Ab functionality. Cellular immune response assays will include, but are not limited to IFNγ ELISPOT assay, ICS, and multiparameter flow cytometry. Exploratory assessments on mucosal samples will include, but are not limited to characterization of Env-specific binding Abs. Priority assays are listed below.

9.3.1 Antibody Responses

- Env-specific binding Abs (titers and breadth).
- Env-specific nAbs (titers and breadth).

- Env-specific functional Abs (e.g. phagocytosis score and breadth).
- Env-specific binding Ab isotypes (IgA, IgG1-4) (titers and breadth).

9.3.2 Cellular Responses

- IFNγ peripheral blood mononuclear cell (PBMC) responders to peptide pools and subpools of Potential T-cell epitopes, PTE Env/Gag/Pol peptides.
- $CD4^{+}$ and $CD8^{+}$ T-cell functionality (% cells producing e.g., IFNγ, IL-2, IL-4, TNFα).
- T-cell development with emphasis on follicular helper T-cells and memory differentiation.

9.3.3 PBMC, Serum and Plasma Storage

Samples of cryopreserved PBMC, plasma and serum will be stored as indicated in the Schedule of Procedures in Appendices A, B and C (for details see Analytical Plan (AP)) and, if the participant consents, may be used for the purposes of standardization, quality control and for future assays related to HIV prevention or treatment research and development. These samples will be archived and the testing laboratories will be blinded to the participant's identity.

9.4 Other Assessments

9.4.1 HIV Antibody Testing (Groups 1 and 2)

All HIV-uninfected participants (Group 1 and 2) will be tested for HIV antibodies as indicated in the Schedule of Procedures (Appendix A and B) or as needed, if medical or social circumstances arise. All participants will receive HIV risk reduction counselling and pre- and post-HIV-test counselling, as specified in Section 7.3 Counselling.

9.4.2 Pharmacokinetics

Blood draws for pharmacokinetics will be done on the day of investigational product infusion immediately before starting IV infusion(s) of investigational product, at the end of the investigational product infusion(s), and 3, 6 and 24 hours after the investigational product infusions. Thereafter, pharmacokinetic draws will be done as indicated in the Schedule of Procedures (Appendices A, B and C).

PGDM1400 and PGT121 mAb pharmacokinetic analyses will be performed using, but not limited to, standard non-compartmental analysis methods to estimate elimination half-life (t1/2), clearance (CL/F), volume of distribution (Vz/F), Area under the concentration decay curve (AUC), impact of viral load and/or ART on PGDM1400 and PGT121 mAb disposition (elimination half-life (t1/2), clearance (CL/F), volume of distribution (Vz/F) and total exposure. PGDM1400 ± PGT121 accumulation will also be examined in rectal and cervical mucosal secretions collected with rectal sponges (or comparable swab) or cervical Softcups (or comparable cervical fluid collection cup) in study participants who specifically consented for these procedures. Descriptive results will be reported for the pharmacokinetic parameters by dose Subgroup.

Exploratory analysis using population analysis methods simultaneously combining all pharmacokinetic data across all doses and treatment Subgroups and Groups will be performed for quantitative characterization of differences in PGDM1400 and PGT121 mAb disposition by dose, participant Subgroup or Group or disease state.

9.4.3 HLA Typing

Samples for HLA typing will be collected as specified in the Schedule of Procedures in Appendices A, B and C (for details see Analytical Plan (AP)) and may be analyzed as warranted.

9.4.5 Pregnancy Test

A urine pregnancy test for all female participants will be performed by measurement of human chorionic gonadotrophin (βhCG) at time points indicated in the Schedule of Procedures (Appendices A, B and C). The results of the pregnancy test must be negative prior to IV infusion of investigational product. See section 10.7 for description of pregnancy after infusion of investigational product.

9.4.6 HIV Risk Assessment (Group 1 and 2)

Study staff will assess participants for their past and current risk of acquiring HIV at time points indicated in Schedule of Procedures (Appendix A and B).

9.4.7 Social Impact Assessment

A brief assessment of the impact of participation in the study will be administered to participants at their final study visit.

10.0 ADVERSE EVENTS

10.1 Definition

An adverse event (AE) is any untoward medical occurrence in a participant administered an Investigational Product and which does not necessarily have a causal relationship with the Investigational Product. An AE can therefore be any unfavourable or unintended sign (including an abnormal laboratory finding), symptom, or disease, temporally associated with the use of Investigational Product whether or not related to the Investigational Product.

Assessment of severity of all AEs, including and seriousness of AEs, is ultimately the responsibility of the Principal Investigator of each site. Refer to the DIVISION OF AIDS (DAIDS) TABLE FOR GRADING THE SEVERITY OF ADULT AND PEDIATRIC ADVERSE EVENTS Version 2.0, November 2014 and the National Cancer Institute Common Terminology Criteria for Adverse Events (CTCAE) Version 4.03: June 14, 2010 for additional guidance.

10.2 Assessment of Severity of Adverse Events

The following general criteria should be used in assessing adverse events as mild, moderate, severe or very severe at the time of evaluation:

Grade 1 (Mild): Symptoms causing no or minimal interference with usual social & functional activities

Grade 2 (Moderate): Symptoms causing greater than minimal interference with usual social & functional activities

Grade 3 (Severe): Symptoms causing inability to perform usual social & functional activities

Grade 4 (Very Severe): Symptoms causing inability to perform basic self-care functions OR Medical or operative intervention indicated to prevent permanent impairment, persistent disability, or death

Guidelines for assessing the severity of specific adverse events and laboratory abnormalities are listed in Appendix G, DAIDS Adverse Event Severity Assessment Table.

10.3 Relationship to Investigational Product

Assessment of relationship of an AE or SAE to Investigational Product is the responsibility of the Principal Investigator or designee. All medically indicated and available diagnostic methods (e.g., laboratory, blood smear, culture, X-ray, etc.) should be used to assess the nature and cause of the AE/SAE. Best clinical and scientific judgment should be used to assess relationship of AE/SAEs to the investigational product and/or other cause.

The following should be considered:

- Presence/absence of a clear temporal (time) sequence between administration of the investigational product and the onset of AE/SAE
- Presence/absence of another cause that could more likely explain the AE/SAE (concurrent disease, concomitant medication, environmental or toxic factors)
- Whether or not the AE/SAE follows a known response pattern associated with the investigational product

The relationship assessment should be reported as one of the following:

Not Related: clearly explained by another cause (concurrent disease, concomitant medication, environmental or toxic factors, etc.).

Unlikely: more likely explained by another cause (concurrent disease, concomitant medication, environmental or toxic factors, etc.).

Possibly: equally likely explained by another cause but the possibility of the investigational product relationship cannot be ruled out (e.g., reasonably well temporally related and/or follows a known investigational product response pattern but equally well explained by another cause).

Probably: more likely explained by the investigational product (e.g., reasonably well temporally related and/or follows a known investigational product response pattern and less likely explained by another cause).

Definitely: clearly related and most likely explained by the investigational product.

For the purpose of expedited safety reporting, all possibly, probably or definitely related SAEs are considered investigational product-related SAEs.

10.4 Serious Adverse Events

An adverse event is reported as a "Serious Adverse Event" if it meets any of the following criteria (as per International Conference on Harmonisation [ICH] Good Clinical Practice [GCP] Guidelines):

- Results in death
- Is life threatening
- Results in persistent or significant disability/incapacity
- Requires in-participant hospitalization or prolongs existing hospitalization
- Is a congenital anomaly/birth defect or spontaneous abortion
- Any other important medical condition that requires medical or surgical intervention to prevent permanent impairment of a body function or structure

Elective surgery for pre-existing condition that did not increase in severity or frequency is not considered an SAE.

Serious Adverse Events (SAEs) should be reported within 24 hours of the site becoming aware of the event, and sent to the Sponsor as described in the SOM.

To discuss investigational product-related SAEs or any urgent medical questions related to the SAE, the site investigator should contact one of the IAVI Medical Monitors directly (see Contact List in the SOM).

The IAVI SAE Report Form should be completed with all the available information at the time of reporting and sent to the Sponsor as described in the SOM. The minimum data required in reporting an SAE are the study identification number, date of birth, gender, event description (in as much detail as is known at the time), onset date of event (if available), reason event is classified as serious, reporting source (name of Principal Investigator or designee), and relationship to the investigational product as assessed by the investigator.

The Principal Investigator or designee is required to prepare a detailed written report with follow up until resolution or until it is judged by the Principal Investigator or designee to have stabilized.

The Principal Investigator or designee must notify the local IRB/IEC of all SAEs as appropriate. In case of investigational product-related SAEs, the Sponsor will notify responsible regulatory authorities, Safety Monitoring Committee (SMC), and other study sites where the same investigational product is being tested.

More details on SAE definitions and reporting requirements are provided in the SOM.

<u>Serious Event Prior to Investigational Product Administration</u>

If a serious event occurs in the period between the participant signing the Informed Consent Form and receiving the IV infusion of investigational product, the event will be reported using the SAE form and following the same procedures for SAE reporting, as indicated in Section 10.4. The timing of the event will be indicated by using the relevant checkbox on the SAE form.

10.5 Reporting Potential Immune-Mediated Diseases

Potential immune-mediated diseases (pIMDs) are a subset of AEs that include both clearly autoimmune diseases and also other inflammatory and/or neurologic disorders that may or may not have an autoimmune etiology. These events are of special interest since they could potentially be caused by immune responses to the investigational product. The investigator/designee should report such adverse events within the same time limits (following confirmation of an AE as a pIMD; see last paragraph of this section below), and using the same CRF pages, as utilized for SAEs. The investigator or his/her designee will evaluate the occurrence of pIMDs at every visit/contact during the study. IAVI will also expect investigators/designee to provide additional information about pIMD events. AEs to be reported and documented as pIMDs include:

<u>Neuroinflammatory disorders</u>: optic neuritis, cranial nerve disorders (including Bell's palsy), multiple sclerosis, demyelinating disease, transverse myelitis, Guillain-Barré syndrome, myasthenia gravis, encephalitis, neuritis.

<u>Musculoskeletal disorder</u>s: systemic lupus erythematosus, cutaneous lupus, Sjögren's syndrome, scleroderma, dermatomyositis, polymyositis, myopathy, rheumatoid arthritis and juvenile rheumatoid arthritis, polymyalgia rheumatica or temporal arteritis, reactive arthritis, psoriatic arthropathy, ankylosing spondylitis, undifferentiated spondyloarthropathy.

<u>Gastrointestinal disorders</u>: Crohn's disease, ulcerative colitis or proctitis, celiac disease.

<u>Metabolic diseases</u>: autoimmune thyroiditis, Grave's or Basedow's disease, Hashimoto thyroiditis, insulin-dependent diabetes mellitus (IDDM), Addison's disease.

<u>Skin disorders</u>: psoriasis, vitiligo, Raynaud's phenomenon, erythema nodosum, autoimmune bullous skin diseases.

<u>Others:</u> autoimmune hemolytic anemia, thrombocytopenia, antiphospholipid syndrome, *vasculitis, pernicious anemia, autoimmune hepatitis, primary biliary cirrhosis, primary sclerosing cholangitis, autoimmune glomerulonephritis, autoimmune uveitis, autoimmune myocarditis/cardiomyopathy, sarcoidosis, Stevens-Johnson syndrome, Behçet's syndrome.

<u>Infusion site reactions:</u> Grade 3 or 4 infusion site reactions lasting more than 2 days.

<u>*Vasculitis:</u> Vasculitis, Diffuse vasculitis, leucocytoclastic vasculitis, polyarteritis nodosa, microscopic polyangiitis, Wegener's granulomatosis, anti-neutrophil cytoplasmic antibody positive vasculitis, Henoch-schönlein purpura, allergic granulomatous angiitis (Churg-Strauss disease), Kawasaki disease, Takayasu's arteritis, temporal arteritis (giant cell arteritis), renal vasculitis.

Medical judgement should be exercised in deciding whether other disorders/diseases have an autoimmune origin and should also be reported as described above, and this judgement is the investigator's prerogative. Whenever sufficient data exist to substantiate any of the diagnoses in the above list, the event must be reported as a pIMD. While the intent of pIMD reporting is to be inclusive, isolated nonspecific symptoms, which might (or might not) represent the above diagnoses, should be captured as AEs but not reported as pIMDs until the diagnosis can be defended.

10.6 Clinical Management of Adverse Events

Adverse events (AEs) will be managed by the clinical study team who will assess, provide first line of care as appropriate and refer to health care and treatment facilities as warranted. If any treatment/medical care is required as a result of the harm caused by the investigational product or study procedures, this will be provided free of charge.

If a participant has an AE and/or abnormal laboratory value that is known at the time of IV infusion of investigational product, the specifications of Section 12.0 will be followed.

Participants will be followed until the AE resolves or stabilizes or up to the end of the study, whichever comes last. If at the end of the study, an AE (including clinically significant laboratory abnormality) that is considered possibly, probably or definitely related to the investigational product is unresolved, follow-up will continue until resolution if possible and/or the participant will be referred.

If a participant from Group 3 experiences a significant decrease in CD4 cell count (e.g., – 20% of baseline, or decline to <200 cells/µL) during the course of the trial, participants will be monitored closely until their CD4 count returns to baseline or until the participant initiates ART. Participants whose CD4 cell counts decrease to <200 cells/µL will be promptly informed and will be referred to their primary HIV care provider. Appropriate prophylaxis against opportunistic infections will be instituted according to accepted U.S. HIV treatment guidelines.

10.7 Clinical Management of Infusion-Related Reactions and Stopping Criteria

Infusion-related reactions can be local or systemic. Depending on the severity of the reaction, administration of IP can continue, be paused or stopped. Use clinical judgement and follow site-specific SOPs for anaphylaxis / infusion-related reactions. At a minimum, the following medications should be available for immediate use during the infusion: 25 mg IV diphenhydramine x 1, 125 mg IV methylprednisolone x 1, epinephrine (1:1000) 0.5 mg/ 0.5 mL IV x 1, albuterol inhaler, and albuterol nebulizer. These medications should be included with the prescription for IP, if necessary (some sites may stock these medications on site already), so that they are available at the bedside to be administered consistent with site SOPs for anaphylaxis / infusion-related reactions.

For infusion related reactions, including cytokine release syndrome, use the Common Terminology Criteria for Adverse Events (CTCAE), Version 4.03 June 2010 grading scale.

Infusion stopping criteria:

Grade	
1	Monitor participant closely, intervention not indicated
2	Stop and re-start if clinically acceptable
3	Stop, do not re-start
4	Stop, do not re-start

Mild reaction (Grade 1)

- Inform medical staff
- Monitor volunteer for signs of systemic reaction. If there is no sign of systemic reaction continue the infusion of IP at the same rate
- Continue to monitor volunteer, manage volunteer according to local guidelines and SOPs
- Document the events
- Do not continue the infusion of any IP (1st or 2nd) if there is any concern
- If infusion of 1st IP is not continued, do not proceed with infusion of 2nd IP

Systemic reaction (Grade 2)

- Stop administration of IP at the catheter level, i.e., as close to the arm as possible to prevent any more IP from entering the body. Remember that there is IP in the intravenous line downstream from the IP IV saline bag (removing the IP IV saline bag and replacing with a 0.9% saline bag or another IV fluid on the 1st intravenous line will result in additional administration of the several ml of IP that is in the line).
- Inform medical staff
- Monitor volunteer and continue to monitor until the volunteer is stable
- Ensure that 0.9% saline is running on the 2nd intravenous line
- The Principal Investigator, or study physician on call, must decide whether to restart or stop the administration of IP
  - If the volunteer improves discuss restarting infusion of IP, and at what rate, with Principal Investigator, or study physician on call.
  - If the volunteer does not improve or condition becomes worse, commence basic resuscitation according to site guidelines and SOPs and administer intravenous methylprednisolone and intravenous diphenhydramine. Replace the intravenous line connected to the 1st intravenous catheter (these lines contain IP).
- Document the events

Cytokine Release Syndrome or anaphylactic or anaphylactoid reaction (Grade 3 or 4)

- Stop administration of IP at the catheter level, i.e., as close to the arm as possible to prevent any more IP from entering the body. Replace the intravenous line connected to the 1st intravenous catheter (these lines contain IP).
- Call for assistance
- Monitor volunteer and continue to monitor until the volunteer is stable
- Ensure that 0.9% saline is running on the 2nd intravenous line
- Commence management of anaphylaxis according to site guidelines and SOPs
- Document the events

10.8 Pregnancy

Although not considered an AE, if a female participant becomes pregnant during the study, it is the responsibility of the Principal Investigator or designee to report the pregnancy promptly to IAVI using the designated forms. The participant will be followed for safety until the end of pregnancy or study completion, whichever occurs last. If possible, approximately 2–4 weeks after delivery, the baby will be examined by a physician to assess its health status and the results will be reported to the Sponsor. The baby will be examined again by a Physician around age 1, and the results will be reported to the Sponsor.

Complications of pregnancy that meet criteria for SAEs, specified in Section 10.4 of this Protocol (e.g., hospitalization for eclampsia, spontaneous abortion, etc.) should be reported as SAEs.

10.9 Intercurrent HIV Infection (Group 1 and 2)

HIV infection cannot be directly caused by the investigational product. If a participant acquires HIV through exposure in the community, at any time after the IV infusion of investigational product, the participant should be offered referral to appropriate care and treatment facilities. The participant will continue to be followed in the study for safety assessments.

Intercurrent HIV infection in study participants, although not considered an SAE, must be reported promptly to IAVI using the designated forms. However, medical conditions associated with the HIV infection that meet criteria for being serious specified in the Section 10.4 of this Protocol (e.g., sepsis, *Pneumocystis jiroveci [carinii] pneumonia*, etc.) should be reported as SAEs using the SAE Report Form.

11.0 MANAGEMENT OF HIV ISSUES DURING AND FOLLOWING STUDY

11.1 HIV Testing – Groups 1 and 2

Group 1 and 2 participants will be tested for HIV antibodies as indicated in the Schedule of Procedures (Appendix A and B) or as needed, if medical or social circumstances arise. All participants will receive HIV risk reduction counselling and pre- and post-HIV-test counselling, as specified in Section 11.3.1, Counselling (Group 1 and 2).

It is possible that the Investigational Product(s) or an immune response to the Investigational Product(s) could cause a positive result on a diagnostic HIV antibody test. An investigational product recipient who falsely tests HIV positive with a diagnostic HIV antibody test at the end of the study will be informed of his/her positive test result and offered continuing follow-up until the test becomes negative.

If a participant acquires HIV through exposure in the community, at any time after the administration of investigational product, the participant will be offered referral to appropriate care and treatment facilities. The participant will continue to be followed in the study for safety assessments.

Should a participant require HIV testing outside of the study for personal reasons, it is recommended that the participant contact the study staff first. HIV testing can be done

at the study site and then processed at an independent laboratory as above. Written evidence of HIV status (HIV-infected or HIV-uninfected) will be provided upon request.

11.2 Social Discrimination as a Result of investigational product-related antibodies

In order to minimize the possibility of social discrimination in participants (if any) who test positive on a diagnostic HIV antibody test due to investigational product-related antibodies, appropriate diagnostic HIV testing and certification will be provided both during and after the study as needed.

11.3 HIV infection – Group 1 and 2

Group 1 and 2 participants who are diagnosed with HIV infection at screening or during the study (intercurrent HIV-infection) will be provided the following:

11.3.1 Counselling

The participant will be counselled by the study investigators or designated counsellors. The counselling process will assist the participant with the following issues:

- Psychological and social implications of HIV infection
- Who to inform and what to say
- Implications for sexual partners
- Implications for child-bearing
- Avoidance of transmission to others in future
- Mandatory reporting to the state, in some instances

11.3.2 Referral for Support/Care

Participants will be referred to a participant support center or institution of his/her choice for a full discussion of the clinical aspects of HIV infection. Referral will be made to a designated physician or center

12.0 WITHDRAWAL FROM STUDY

12.1 Deferral of IV infusion of investigational product

An IV infusion of investigational product may be temporarily deferred if the participant is clinically ill at the time of the administration of investigational product visit and/or presents with fever ($\geq$ 100.4 °F) at the time of the administration of investigational product. A participant must be clinically well and afebrile for a minimum of a 24-hour consecutive period prior to administration of investigational product.

Any planned or unplanned deferral of infusion of investigational product will be discussed with the Sponsor. Participants will be deferred from infusion of investigational product for any of the following reasons:

- Pregnancy
- A disease or condition or adverse event that may develop, regardless of relationship to Investigational Product, if the Principal Investigator or designee is of the opinion that administration of investigational product will jeopardize the safety of the participant

- Participant's request to defer infusion

The following events require resolution and/or review of clinical history by the Principal Investigator or designee and consultation with the Medical Monitor, prior to administration of investigational product:

- Any abnormal laboratory value, as outlined in section 5.7, Exclusion Criteria, Hematology, Chemistry, Urinalysis that is known at the time of infusion and have not resolved.
- Receipt of inactivated/killed/subunit vaccines (non-HIV) or immunoglobulin within the previous 14 days. Receipt of live attenuated vaccines within the previous 30 days.
- Participating in another clinical study of an Investigational Product

12.2 Withdrawal from the Study (Early Termination)

Participants may be withdrawn from the study permanently for the following reasons:

1. Participants may withdraw from the study at any time if they wish, for any reason
2. The Principal Investigator or designee has reason to believe that the participant is not complying with the protocol
3. If the Sponsor decides to terminate or suspend the study

If a participant withdraws or is withdrawn from the study, all termination visit procedures will be performed according to the Schedule of Procedures (Appendices A, B and C) where possible. Every effort will be made to determine and document the reason for withdrawal.

13.0 DATA HANDLING

13.1 Data Collection and Record Keeping at the Study Site

Data Collection: All study data will be collected by the clinical study staff using designated source documents and entered onto the appropriate electronic CRFs (eCRFs). Access to eCRFs will be provided via an electronic data entry system hosted by the Data Coordination Center. All study data must be verifiable to the source documentation. A file will be held for each participant at the clinic(s) containing all the source documents. Source documentation will be available for review to ensure that the collected data are consistent with the CRFs.

All CRFs and laboratory reports will be reviewed by the clinical team, who will ensure that they are accurate and complete.

Source documents and other supporting documents will be kept in a secure location. Standard GCP practices will be followed to ensure accurate, reliable and consistent data collection.

Source documents include but are not limited to:

- Signed Informed Consent Documents
- Progress notes
- Data collection forms
- Documentation of any existing conditions or past conditions relevant to eligibility

- Printed laboratory results
- Print out of the generated enrollment confirmation
- All Adverse Events
- Concomitant medications
- Local and systemic reactogenicity events

13.2 Data Entry at the Study Site

The data collected at the site will be recorded onto the eCRFs by the study staff and entered into a database. To provide for real time assessment of safety, data should be entered as soon as reasonably feasible after a visit occurs.

13.3 Data Analysis

The Sponsor, PIs and Product Developers will agree on how data will be analyzed and presented prior to unblinding of the study.

The DCC will conduct the data analysis and will provide interim safety and final study reports for the Sponsor, Principal Investigators, the PSRT and SMC and the regulatory authorities, as appropriate.

14.0 STATISTICAL CONSIDERATIONS

14.1 Safety and Tolerability Analysis

14.1.1 Sample Size

The sample size for safety and tolerability analysis will be 36-84 participants according to the dose escalation design used to characterize the safety profile of one IV infusion of PGDM1400 ± PGT121 mAb, at one of three dose levels.

14.1.2 Null Hypothesis

As this is an exploratory proof of concept trial and analysis will be descriptive, no formal null hypothesis will be tested.

14.1.3 Statistical Power and Analysis and Dose Escalation Rules

The frequency (percentage) of moderate or greater local and systemic reactogenicity events (along with 95% confidence intervals) will be determined for each active Group and placebo.

The frequency of SAEs judged possibly, probably or definitely related to the investigational product will be determined.

All AEs will be analyzed and grouped by seriousness, severity and relationship to the investigational product (as judged by the investigator).

For life-threatening adverse events related to investigational product: if none of the 18 (max 36) participants receiving active product experience such reactions, then the exact 95 % upper confidence bound for the rate of these adverse events in the population is 18.5% (or 9.7% if n=36).

All AEs will be analysed and grouped by seriousness, severity and relationship to the investigational product (as judged by the investigator).

For life-threatening adverse events related to active product: if none of the 9 (max 18) participants in either Group 1 or Group 2 who receive the active product experience such reactions then the exact 95% upper confidence bound for the rate of these adverse events in the population is 33.6% (or 18.5% if n=18).

An interim analysis of Subgroup and Group data will be carried out according to the study schema (Table 5.3.1) without unblinding the study to investigators or participants. At the end of the study, a full analysis will be prepared.

Based on previous experience with IAVI Phase 1 investigational product studies, it is expected that the amount of missing, unused or spurious data will be insignificant. Unused and spurious data will be listed separately and excluded from the statistical analysis. Missing data will be considered missing completely at random and excluded from the statistical analysis.

14.2 Pharmacokinetic Analysis

14.2.1 Sample Size

The sample size for pharmacokinetic analysis will be 3 per dose Subgroup, to provide sufficient information for the planned analyses.

14.2.2 Null Hypothesis

As this is an exploratory proof of concept trial and analysis will be descriptive, no formal null hypothesis will be tested.

14.2.3 Statistical Power and Analysis

Disposition of PGDM1400 mAb and PGT121 mAb will be evaluated in this study. Based on the PK profile of other human monoclonal antibodies, it is expected that the half-life of PGDM1400 mAb and PGT121 mAb will be 14 to 21 days. Previously published data indicate that the pharmacokinetics of VRC01, 3BNC117 and 10-1074 are fairly similar across phase 1 studies (Table).

Table 14.2.3-1: Key parameters of select Antibodies for the Prevention and Treatment of HIV Infection

	**3BNC117 (Caskey, Klein et al. 2015)**	**VRC01 (Ledgerwood, Coates et al. 2015, Lynch, Boritz et al. 2015)**	**10-1074 (Caskey, Schoofs et al. 2017)**
**Binding site on HIV ENV spike**	CD4 binding site	CD4 binding site	V3 loop
**Human safety data available**	Yes, safe and well tolerated	Yes, safe and well tolerated	Yes, safe and well tolerated
**PK HIV-uninfected, terminal half life (days)**	17	15	24
**PK HIV-infected & viremic, terminal half life (days)**	9	12 (i.v.), 11 (s.c.)	12.8

**Antiviral effect (average or range log_{10} decrease HIV RNA)**	1.48	1.1-1.8	1.52

Commonly reported PK parameters will be calculated using, but not limited to, standard non-compartmental slope/height/area/moment (SHAM) analysis methods. Summary descriptive results of PK parameters, including AUC, Cmax, T1/2, and clearance results will be reported by dose cohort. Dose normalized plots of PK parameters will be presented. Correlation between PK and reported safety and pharmacodynamic outcomes will also be explored parameters in order to examine exposure-effect relationships.

A more powerful exploratory analysis to quantitatively determine the dose, participant and disease impact on PGDM1400 mAb and PGT121 mAb pharmacokinetics, and correlate exposure with response, while correctly accounting for variance based on population intrinsic factors such as weight and gender will be performed. Using the proposed population analysis approach we will be able to simultaneously examine the magnitude and the rate of change to PGDM1400 and PGT121 disposition driven by HIV-1 RNA levels, and also examine the magnitude and the rate of decline in log copies/ml of HIV-1 RNA plasma levels from baseline.

The frequency and levels of anti-PGDM1400 antibodies and anti-PGT121 antibodies will be calculated and tabulated.

14.3. Virologic Analysis for Group 3

14.3.1 Sample Size

The sample size for virologic analysis in Groups 3A and 3B will be 12-36 participants according to the design described below.

14.3.2 Null Hypothesis

The null hypothesis is that there is a mixture of responders and non-responders with mixture probability 0.5 such that the difference-from-baseline is greater than -0.9 logs HIV RNA viral load in the responder group and 0 logs HIV RNA viral load in the non-responder group.

14.3.3 Statistical Power and Analysis

The virologic analysis described in this section relates to Subgroups 3A and 3B of the study design, in which antiviral activity of PGDM1400 mAb alone and a combination of PGDM1400 mAb + PGT121 mAb is measured in HIV-infected participants off ART with plasma HIV RNA levels of 1×10^3 – 1×10^5 copies/ml. This section assumes that Part 1 of the study has successfully demonstrated that there are safe dose levels of PGDM1400 mAb, and PGDM1400 mab + PGT121 mAb. No placebo participants are enrolled as part of this design.

The primary efficacy outcome for this analysis is defined as change in log10 viral load between Day 0 (day of infusion) and Day 7. The minimum clinically significant value for this outcome is defined as a difference of –0.9 log10.

Groups 3A: this Group will enroll participants sequentially. After administering PGDM1400 at the MTD, as defined in part 1 of this study, the day 7 post-infusion viral load measurement will be compared to the baseline viral load measurement. If the difference in viral load at 7 days post-infusion is greater than a 0.5 log10 drop, the participant is categorized as a responder; otherwise, the participant is categorized as a non-responder. Enrollment will continue until at least 6 responders or 18 total participants have been enrolled whichever occurs first.

Group 3B: this Group will enroll participants sequentially. After administering PGDM1400 mAb and PGT121 mAb at the MTD, as defined in part 1 of this study, the day 7 post-infusion viral load measurement will be compared to the baseline viral load measurement. If the difference in viral load at 7 days post-infusion is greater than a 0.5 log10 drop, the participant is categorized as a responder; otherwise, the participant is categorized as a non-responder. Enrollment will continue until at least 6 responders or 18 total participants have been enrolled whichever occurs first.

Antiviral activity in Subgroup 3A and 3B will be tested in the responder group only using a one-sided Wilcoxon signed rank test against the null hypothesis of a "shift" parameter of -0.9 $\log_{10}$. Based on a simulation study outlined in the SAP, the power to reject the null hypothesis is 80% when the responder group has a difference-from-baseline viral load drop of approximately 1.8 logs for a nominal alpha level of 0.05. Since the responder group is defined by the outcome it is not surprising that the Type I error is greater than the nominal alpha level which was deemed an acceptable trade-off between sample size and power to detect the desired effect under the alternative described above. Notably, the Type I error drops below the nominal error rate when the true shift in viral load is in the responder group is greater than -0.5 logs.

For the analysis of sample size and power, log10 viral load differences from baseline for each participant were simulated from a normal distribution, with a standard deviation of 0.5. This value was chosen by examining a study of the antiretroviral drug raltegravir, which demonstrated a mean estimated standard deviation of the change of baseline of 0.47(Andrade, Rosenkranz et al. 2013). This is a conservative estimate, as the variability of viral loads near the lower range might be expected to also be lower.

The statistical test performed will be the Signed-ranktest, which will incorporate the “shift” parameter of –0.9 log10 (the minimum clinically significant difference selected for this study). An evaluation of potential harm (increased viral load) will also be performed with the Signed ranktest; this test will examine the null hypothesis of no change in viral load (a shift of 0.0 log10 following investigational product administration) against the one-sided alternative hypothesis that the viral load is increased following investigational product administration. Each efficacy test will be performed at the level α = 0.05. Each test for harm will be performed at level 2α = 0.10, in order to provide additional sensitivity to detect potential harm.

14.5 Secondary and Exploratory Immunologic and Virologic Analyses

14.5.1 Sample Size

The sample size for secondary immunologic and/or virologic analysis will be the 12-36 HIV infected participants in part 2 of the study.

14.5.2 Null Hypothesis

No formal hypothesis on immunologic or virologic responses will be tested, with the exception of the change in HIV viral load described in Section 14.3.

14.5.3 Statistical Power and Analysis

Descriptive statistics (actual values and changes from reference) will be calculated for continuous immunologic and virologic parameters at all time points. Graphical representations of changes in parameters will be made as applicable. Differences between Subgroups and/or Groups at a specific time point will be tested for exploratory purposes by a 2-sample t-test if the data appear to be normally distributed (after transformation if necessary). If not, the non-parametric Wilcoxon rank sum test will be used. If portions of the measurements are censored below the assay quantification limit, the Gehan-Wilcoxon test will be employed. All statistical tests will be two-sided and will be considered statistically significant if $p < 0.05$.

Frequency tabulations will be calculated for discrete (qualitative) immunologic and virologic parameters at all time points. Significant differences between Subgroups and/or Groups will be determined by a 2-sided Fisher's exact test.

Interim immunologic and virologic analyses of grouped data may be performed without unblinding the study to investigators or participants.

15.0 QUALITY CONTROL AND QUALITY ASSURANCE

To ensure the quality and reliability of the data collected and generated and the ethical conduct of this study, a Study Operations Manual (SOM) will be developed. All deviations will be reported and investigated. The SOM describes reporting and deviation documentation requirements and procedures.

Regular monitoring will be performed according to ICH-GCP as indicated in Section 17.5.

An independent audit of the study and study sites may be performed by the Sponsor or designee to establish the status of applicable quality systems. Inspection by regulatory authorities may also occur.

By signing the protocol, the Principal Investigators agree to facilitate study related monitoring, audits, IRB/IEC review and regulatory inspection(s) and direct access to source documents. Such information will be treated as strictly confidential and under no circumstances be made publicly available.

16.0 DATA AND BIOLOGICAL MATERIAL

All data and biological material collected through the study shall be managed in accordance with the Clinical Trial Agreement (CTA). Distribution and use of these data will be conducted by agreement of all parties.

The computerized raw data generated will be held by the DCC on behalf of the Sponsor. The study sites will also hold the final data files and tables generated for the purpose of analysis.

17.0 ADMINISTRATIVE STRUCTURE

The Principal Investigator will be responsible for all aspects of the study at the study site.

17.1 Protocol Safety Review Team

A PSRT will be formed to monitor the clinical safety data. During the administration of investigational productphase of the trial, the PSRT will review the clinical safety data on a weekly basis via electronic distribution of reports. An ad hoc PSRT review meeting will occur if any of the members of the PSRT requests a special review to discuss a specific safety issue or as specified in the Study Operations Manual. After the administration of investigational product phase the PSRT will review the clinical safety data at least monthly.

The PSRT will consist of the IAVI Medical Monitor(s), and the PI or designee from each clinical team. The study chair or an IAVI Medical Monitor may be the PSRT chair. *Ex officio* members will include the IAVI Chief Medical Officer and an unblinded IAVI Medical Monitor. Additional PSRT participants may include the following, as needed:

- Co-investigators and trial site senior clinical research nursing staff
- Laboratory directors
- Data management, study statistician and regulatory staff

The PSRT membership and procedures are detailed in the PSRT charter.

17.2 Safety Monitoring Committee (SMC)

The SMC will consist of independent clinicians/scientists/statisticians/ethicists who are not involved in the study. Investigators responsible for the clinical care of participants or representative of the Sponsor may not be a member of the SMC. Details of membership, chair and co-chair and responsibilities are outlined in the SMC charter.

Principal Investigator(s) or designee and/or a Sponsor representative may be asked to join an open session of the SMC meeting to provide information on study conduct, present data or to respond to questions.

Safety data will be reviewed by the SMC at pre-specified time points and at an ad-hoc basis.

17.2.1 Content of Interim Safety Review

The SMC will be asked to review the following blinded data:

- Summary of reactogenicity (i.e., solicited adverse events)
- All adverse events judged by the Principal Investigator or designee to be possibly, probably or definitely related to investigational product
- All laboratory results confirmed on retest and judged by the Principal Investigator or designee to be clinically significant
- All SAEs and pIMDs

An unblinded presentation of all above noted events may also be made available for the SMC for their review if required by any member of the SMC.

17.2.2 SMC Review of Group 1 and 2 data prior to starting Group 3

Following IV infusion of investigational product of the last participant in Groups 1 and/or 2, the Safety Monitoring Committee (SMC) will review safety data through the day 14 post-IV infusion visit for all participants to confirm MTD in each Group, and determine whether, and at what dose level, Groups 3A and 3B can initiate enrollment. See section 5.3 for additional details.

17.3 Criteria for Pausing the Study

Enrollment and administration of investigational product will be stopped and a safety review conducted by the SMC for any of the following criteria:

- One or more participants experience an SAE that is judged possibly, probably or definitely related to investigational product.
- There is a participant death, regardless of relationship to the investigational product.
- Two or more participants experience Grade 3 adverse events in the same category System Organ Class that are considered possibly, probably or definitely related to investigational product or
- Any Grade 4 adverse event that is considered possibly, probably or definitely related to investigational product.

Table 17.3-1: AE notification and safety pause/AE review rules

Event and relationship to study product	Severity	Occurrence	Site PI action	PSRT or SMC action
SAE, possibly, probably or definitely related	Any	Any	Phone, email or fax forms to sponsor within 24 hours	Study pause within 24 hours, refer to SMC for review
SAE, probably not or not related	Death	Any	Phone, email or fax forms to sponsor within 24 hours	Study pause within 24 hours, refer to SMC for review
AE, possibly, probably or definitely related	Grade 4	Any	Phone, email or fax notification to sponsor within 24 hours	Study pause within 24 hours, refer to SMC for review
AE¶, possibly, probably or definitely related	Grade 3*	First	Phone, email or fax notification to sponsor within 24 hours	PSRT review within 2 business days to consider pause
AE¶, possibly, probably or definitely related	Grade 3*	Second‡	Phone, email or fax notification to sponsor within 24 hours	Study pause within 24 hours, refer to SMC for review

¶Does not include the following reactogenicity symptoms (fever, fatigue/malaise, myalgia, arthralgia, chills, headache, nausea, vomiting).

*If no evidence of disease is present other than an abnormal laboratory value, the test must be repeated with a new blood sample at least one time within 72 hours after the investigator becoming aware of the abnormal laboratory value. When signs and symptoms are present, repeat test will not be needed.

‡PSRT will determine whether the reported related AE (Grade 3) is a second occurrence of a previously reported AE (Grade 3).

The Sponsor will request a review by the SMC, (or the SMC chair if other SMC members cannot be convened), to be held within 2 business days of the Sponsor learning of the event. The individual participant(s)/or study may be unblinded at the discretion of the SMC. Following this review, the SMC will make a recommendation regarding the continuation or suspension of the administration of the investigational product or the trial and communicate this decision immediately to the Sponsor. The Sponsor then will inform the Principal Investigators without delay.

Additional *ad hoc* review may be specifically requested by the Sponsor, the Principal Investigator(s) or by the SMC.

17.4 Study Supervision

The SMC, the IAVI Chief Medical Officer (CMO) and the IAVI Medical Monitor(s) have access to progress report(s) of this study. Close cooperation will be necessary to track study progress, respond to queries about proper study implementation and management, address issues in a timely manner, and assure consistent documentation, and share information effectively. Rates of accrual, retention, and other parameters relevant to the site's performance will be regularly and closely monitored by the study team.

17.5 Study Monitoring

On-and/or off-site monitoring will ensure that the study is conducted in compliance with human subjects' protection and other research regulations and guidelines, recorded and reported in accordance with the protocol, is consistent with SOPs, GCP, applicable regulatory requirements and locally accepted practices. The monitor will confirm the quality and accuracy of data at the site by validation of CRFs against the source documents, such as clinical records. The investigators, as well as participants through consenting to the study, agree that the monitor may inspect study facilities and source records (e.g., informed consent forms, clinic and laboratory records, other source documents), as well as observe the performance of study procedures (in accordance with site IRB requirements). Such information will be treated as strictly confidential and will under no circumstances be made publicly available.

The monitoring will adhere to GCP guidelines. The Principal Investigator will permit inspection of the facilities and all study-related documentation by authorized representatives of IAVI, and Government and Regulatory Authorities responsible for this study.

17.6 Investigator's Records

Study records include administrative documentation—e.g., reports and correspondence relating to the study—as well as documentation related to each participant screened and/or enrolled in the study—including informed consent forms, case report forms, and all other source documents. The investigator will maintain and store, in a secure manner, complete, accurate, and current study records for a minimum of 2 years after marketing application approval or the study is discontinued and applicable national and local health authorities are notified. IAVI will notify the Principal Investigator of these events.

18.0 INDEMNITY

The Sponsor and Institution are responsible to have appropriate liability insurance. For research-related injuries and/or medical problems determined to result from receiving the investigational product, treatment including necessary emergency treatment and proper follow-up care will be made available to the participant free of charge at the expense of the Sponsor.

19.0 PUBLICATION

A primary manuscript describing safety, anti-viral effect and immune responses in this trial will be prepared promptly after the data analysis is available.

Authors will be representatives of each trial site, the data management and statistical analysis center, the laboratories, the product developer and the sponsor, participant to the generally accepted criteria of contributions to the design and conduct of the study, the analysis of data and writing of the manuscript. Precedence will be given to authors from the site enrolling the greatest number of participants. Manuscripts will be reviewed by representatives of each participating group as specified in the CTA.

20.0 ETHICAL CONSIDERATIONS

The Principal Investigator will ensure that the study is conducted in compliance with the protocol, SOPs in accordance with guidelines formulated by the ICH for GCP in clinical studies, the ethical principles that have their origins in the Declaration of Helsinki and applicable local standards and regulatory requirements.

APPENDIX A: SCHEDULE OF PROCEDURES – GROUP 1 (A, B, C)

Study Month		0							1		2		3	4	5	6
**Study Week**		**0**				**1**	**2**	**3**	**4**	**6**	**8**	**10**	**12**	**16**	**20**	**24**
**Study Day**	**Scr**	**0**	**1**	**2**	**3**	**7**	**14**	**21**	**28**	**42**	**56**	**70**	**84**	**112**	**140**	**168/ET^**
**Visit Windows (Days)**	**-56**	**0**	**0**	**0**	**0**	**± 1**	**± 2**	**± 2**	**± 2**	**± 3**	**± 3**	**±3**	**±7**	**±7**	**±7**	**±7**
**INVESTIGATIONAL PRODUCT**																
Investigational Product		X														
**CONSENT/ASSESSMENTS/COUNSELLING**																
Informed Consent	X															
Assessment of Understanding	X															
HIV Risk Assessment	X															X
HIV Risk Reduction Counselling	X	X							X		X		X	X	X	X
HIV-test Counselling	X	X							X							X
Family Planning Counselling	X	X														
Social Impact Assessment																X
**CLINICAL SAFETY ASSESSMENTS**																
Comprehensive Medical History	X															
Interim Medical History		X	X	X	X	X	X	X	X	X	X					
Concomitant Medications	X	X	X	X	X	X	X	X	X	X	X					
General Physical Exam	X															X
Directed Physical Exam		X	X	X	X	X	X	X	X	X	X	X	X	X	X	
Weight	X	X														X
Height	X															
Vital Signs	X	X*	X	X	X	X	X	X	X	X	X	X	X	X	X	X
Local & Systemic Reactogenicity		X*	X	X	X											
Adverse Events		X	X	X	X	X	X	X	X	X	X					
Serious Adverse Events and pIMD	X	X	X	X	X	X	X	X	X	X	X	X	X	X	X	X

**Study Month**		**0**							**1**		**2**		**3**	**4**	**5**	**6**
**Study Week**		**0**				**1**	**2**	**3**	**4**	**6**	**8**	**10**	**12**	**16**	**20**	**24**
**Study Day**	**Scr**	**0**	**1**	**2**	**3**	**7**	**14**	**21**	**28**	**42**	**56**	**70**	**84**	**112**	**140**	**168/ET[^]**
**Visit Windows (Days)**	**-56**	**0**	**0**	**0**	**0**	**± 1**	**± 2**	**± 2**	**± 2**	**± 3**	**± 3**	**±3**	**±7**	**±7**	**±7**	**±7**
**CLINICAL LABORATORY TESTS**																
Hematology and Coagulation	X	X[#]	X		X	X	X		X		X		X	X	X	X
Clinical Chemistry	X	X[#]	X		X	X	X		X		X		X	X	X	X
Urine Dipstick	X	X[#]	X		X	X	X		X		X		X	X	X	X
Urine Pregnancy test	X	X[#]							X		X		X			X
Active Syphilis	X															
Hepatitis B	X															
Hepatitis C	X															
HIV screen (4th generation Ag/Ab test)	X															
Blinded HIV diagnostic testing		X[#]							X							X
**RESEARCH LABORATORY TESTS**																
Anti PGDM1400 and anti-PGT121 Antibodies (ADA)		X[#]							X		X		X			X
Humoral Assays**		X[#]			X	X	X		X		X		X			X
Cellular Assays**		X[#]					X		X		X		X			X
HLA typing		X[#]														
**PHARMACOKINETICS PGDM1400 ± PGT121 ELISA**		X[#]	X	X	X	X	X	X	X	X	X	X	X	X	X	X
**MUCOSAL SAMPLING**		X[~]	X			X	X									
**PLASMA/SERUM STORAGE**		X	X	X	X	X	X	X	X		X		X			X
**PBMC STORAGE**		X									X		X			X

Day 0 baseline sample collections must be done before infusion of investigational product. Additional day 0 pharmacokinetics sample collection will be done as outlined in Protocol section 9.4.2.
^ Early Termination (ET): Procedures to be performed at ET are the same as last visit procedures
* At baseline, approximately every 30 minutes for the first 4 hours after IP administration, and then every hour until 6 hours after IV infusion. Local and systemic reactogenicity will be assessed by clinic staff at visits on study days 1, 2 and 3. Local and Systemic reactogenicity will also be assessed by the participant using the Memory Aid on study days 1, 2, and 3.
** See Laboratory Analytical Plan for details
~ Cervico-vaginal and/or rectal mucosal sampling (optional) on Day 0 must be done prior to IV infusion of IP.

APPENDIX B: SCHEDULE OF PROCEDURES – AND GROUP 2 (A, B, C)

**Study Month**		**0**							**1**		**2**		**3**	**4**	**5**	**6**
**Study Week**		**0**				**1**	**2**	**3**	**4**	**6**	**8**	**10**	**12**	**16**	**20**	**24**
**Study Day**	**Scr**	**0**	**1**	**2**	**3**	**7**	**14**	**21**	**28**	**42**	**56**	**70**	**84**	**112**	**140**	**168/ET^**
**Visit Windows (Days)**	**-56**	**0**	**0**	**0**	**0**	**± 1**	**± 2**	**± 2**	**± 2**	**± 3**	**± 3**	**±3**	**±7**	**±7**	**±7**	**±7**
**INVESTIGATIONAL PRODUCT**																
Investigational Product		**X**														
**CONSENT/ASSESSMENTS/COUNSELLING**																
Informed Consent	**X**															
Assessment of Understanding	**X**															
HIV Risk Assessment	**X**															**X**
HIV Risk Reduction Counselling	**X**	**X**							**X**		**X**		**X**	**X**	**X**	**X**
HIV-test Counselling	**X**	**X**							**X**							**X**
Family Planning Counselling	**X**	**X**														
Social Impact Assessment																**X**
**CLINICAL SAFETY ASSESSMENTS**																
Comprehensive Medical History	**X**															
Interim Medical History		**X**	**X**	**X**	**X**	**X**	**X**	**X**	**X**	**X**	**X**					
Concomitant Medications	**X**	**X**	**X**	**X**	**X**	**X**	**X**	**X**	**X**	**X**	**X**					
General Physical Exam	**X**															**X**
Directed Physical Exam		**X**	**X**	**X**	**X**	**X**	**X**	**X**	**X**	**X**	**X**	**X**	**X**	**X**	**X**	
Weight	**X**	**X**														**X**
Height	**X**															
Vital Signs	**X**	**X***	**X**	**X**	**X**	**X**	**X**	**X**	**X**	**X**	**X**	**X**	**X**	**X**	**X**	**X**
Local & Systemic Reactogenicity		**X***	**X**	**X**	**X**											
Adverse Events		**X**	**X**	**X**	**X**	**X**	**X**	**X**	**X**	**X**	**X**					
Serious Adverse Events and pIMD	**X**	**X**	**X**	**X**	**X**	**X**	**X**	**X**	**X**	**X**	**X**	**X**	**X**	**X**	**X**	**X**
**CLINICAL LABORATORY TESTS**																
Hematology and Coagulation	**X**	**X#**	**X**		**X**	**X**	**X**		**X**		**X**		**X**	**X**	**X**	**X**

**Study Month**		**0**							**1**		**2**		**3**	**4**	**5**	**6**
**Study Week**		**0**				**1**	**2**	**3**	**4**	**6**	**8**	**10**	**12**	**16**	**20**	**24**
**Study Day**	**Scr**	**0**	**1**	**2**	**3**	**7**	**14**	**21**	**28**	**42**	**56**	**70**	**84**	**112**	**140**	**168/ET^**
**Visit Windows (Days)**	**-56**	**0**	**0**	**0**	**0**	**± 1**	**± 2**	**± 2**	**± 2**	**± 3**	**± 3**	**±3**	**±7**	**±7**	**±7**	**±7**
Clinical Chemistry	**X**	**X#**	**X**		**X**	**X**	**X**		**X**		**X**		**X**	**X**	**X**	**X**
Urine Dipstick	**X**	**X#**	**X**		**X**	**X**	**X**		**X**		**X**		**X**	**X**	**X**	**X**
Urine Pregnancy test	**X**	**X#**							**X**		**X**		**X**			**X**
Active Syphilis	**X**															
Hepatitis B	**X**															
Hepatitis C	**X**															
HIV screen (4th generation Ag/Ab test)	**X**															
Blinded HIV diagnostic testing		**X#**							**X**							**X**
**RESEARCH LABORATORY TESTS**																
Anti PGDM1400 and anti-PGT121 Antibodies (ADA)		**X#**							**X**		**X**		**X**			**X**
Humoral Assays**		**X#**			**X**	**X**	**X**		**X**		**X**		**X**			**X**
Cellular Assays**		**X#**					**X**		**X**		**X**		**X**			**X**
HLA typing		**X#**														
**PHARMACOKINETICS PGDM1400 ± PGT121 ELISA**		**X#**	**X**	**X**	**X**	**X**	**X**	**X**	**X**	**X**	**X**	**X**	**X**	**X**	**X**	**X**
**MUCOSAL SAMPLING**		**X~**	**X**			**X**	**X**									
**PLASMA/SERUM STORAGE**		**X**	**X**	**X**	**X**	**X**	**X**	**X**	**X**		**X**		**X**			**X**
**PBMCs STORAGE**		**X**									**X**		**X**			**X**

Day 0 baseline sample collections must be done before infusion of investigational product. Additional day 0 pharmacokinetics sample collection will be done as outlined in Protocol section 9.4.2.
^ Early Termination (ET): Procedures to be performed at ET are the same as last visit procedures
* At baseline, approximately every 30 minutes for the first 4 hours after IP administration and then every hour until 6 hours after IV infusion. Local and systemic reactogenicity will be assessed by clinic staff at visits on study days 1, 2 and 3. Local and Systemic reactogenicity will also be assessed by the participant using the Memory Aid on study days 1, 2, and 3.
** See Laboratory Analytical Plan for details
~ Cervico-vaginal and/or rectal mucosal sampling (optional) on Day 0 must be done prior to IV infusion of IP.

APPENDIX C: SCHEDULE OF PROCEDURES – GROUP 3 (A, B)

**Study Month**		**0**								**1**		**2**		**3**	**4**	**5**	**6**
**Study Week**		**0**				**1**		**2**	**3**	**4**	**6**	**8**	**10**	**12**	**16**	**20**	**24**
**Study Day**	**Scr**	**0**	**1**	**2**	**3**	**7**	**10**	**14**	**21**	**28**	**42**	**56**	**70**	**84**	**112**	**140**	**168/ET**
**Visit Windows (Days)**	**-42**	**0**	**0**	**0**	**0**	**± 1**	**0**	**± 2**	**± 2**	**± 2**	**± 3**	**± 3**	**±3**	**±7**	**±7**	**±7**	**±7**
**INVESTIGATIONAL PRODUCT**																	
Investigational Product		**X**															
**CONSENT/ASSESSMENTS/COUNSELLING**																	
Informed Consent	**X**																
Assessment of Understanding	**X**																
HIV Risk Reduction Counselling	**X**	**X**								**X**		**X**		**X**	**X**	**X**	**X**
ART counselling	**X**	**X**										**X**					**X**
Family Planning Counselling	**X**	**X**															
Social Impact Assessment																	**X**
**CLINICAL SAFETY ASSESSMENTS**																	
Comprehensive Medical History	**X**																
Interim Medical History		**X**	**X**	**X**	**X**	**X**	**X**	**X**	**X**	**X**	**X**	**X**					
Concomitant Medications	**X**	**X**	**X**	**X**	**X**	**X**	**X**	**X**	**X**	**X**	**X**	**X**					
General Physical Exam	**X**																**X**
Directed Physical Exam		**X**	**X**	**X**	**X**	**X**	**X**	**X**	**X**	**X**	**X**	**X**	**X**	**X**	**X**	**X**	
Weight	**X**	**X**															**X**
Height	**X**																
Vital Signs	**X**	**X***	**X**	**X**	**X**	**X**	**X**	**X**	**X**	**X**	**X**	**X**	**X**	**X**	**X**	**X**	**X**
Local & Systemic Reactogenicity		**X***	**X**	**X**	**X**												
Adverse Events		**X**	**X**	**X**	**X**	**X**	**X**	**X**	**X**	**X**	**X**	**X**					
Serious Adverse Events and pIMD	**X**	**X**	**X**	**X**	**X**	**X**	**X**	**X**	**X**	**X**	**X**	**X**	**X**	**X**	**X**	**X**	**X**
**CLINICAL LABORATORY TESTS**																	
Hematology and Coagulation	**X**	**X#**	**X**		**X**	**X**		**X**		**X**		**X**		**X**	**X**	**X**	**X**
CD4	**X**	**X#**				**X**		**X**		**X**		**X**					**X**

Study Month		0								1		2		3	4	5	6
**Study Week**		**0**				**1**		**2**	**3**	**4**	**6**	**8**	**10**	**12**	**16**	**20**	**24**
**Study Day**	**Scr**	**0**	**1**	**2**	**3**	**7**	**10**	**14**	**21**	**28**	**42**	**56**	**70**	**84**	**112**	**140**	**168/ET**
**Visit Windows (Days)**	**-42**	**0**	**0**	**0**	**0**	**± 1**	**0**	**± 2**	**± 2**	**± 2**	**± 3**	**± 3**	**±3**	**±7**	**±7**	**±7**	**±7**
Clinical Chemistry	X	X#	X		X	X		X		X		X		X	X	X	X
Urine Dipstick	X	X#	X		X	X		X		X		X		X	X	X	X
Urine Pregnancy test	X	X#								X		X		X			X
Active Syphilis	X																
Hepatitis B	X																
Hepatitis C	X																
HIV 4th generation Ag/Ab test***	X																
HIV Viral Load	X	X#	X	X	X	X	X	X	X	X	X	X	X	X	X	X	X
**RESEARCH LABORATORY TESTS**	-	-	-	-	-	-	-	-	-	-	-	-	-	-	-	-	-
Anti PGDM1400 and anti-PGT121 Antibodies (ADA)		X#								X		X		X			X
HIV phenotypic testing for PGDM1400 ± PGT121 susceptibility	X									X							X
HIV SGA sequencing	X									X							X
HIV genotypic testing for ART resistance	X									X				X			X
HIV reservoir size assessment	X							X						X			
Humoral Assays**		X#			X	X		X		X		X		X			X
Cellular Assays**		X#						X		X		X		X			X
HLA typing		X#															
**PHARMACOKINETICS PGDM1400 ± PGT121 ELISA**	X	X#	X	X	X	X		X	X	X	X	X	X	X	X	X	X
**MUCOSAL SAMPLING**		X~	X			X		X									
**PLASMA/SERUM STORAGE**	X	X	X	X	X	X	X	X	X	X		X		X			X
**PBMCs STORAGE**		X										X		X			X

Day 0 baseline sample collections must be done before infusion of investigational product. Additional day 0 pharmacokinetics sample collection will be done as outlined in Protocol section 9.4.2.

^ Early Termination (ET): Procedures to be performed at ET are the same as last visit procedures

* At baseline, approximately every 30 minutes for the first 4 hours after IP administration and then every hour until 6 hours after IV infusion. Local and systemic reactogenicity will be assessed by clinic staff at visits on study days 1, 2 and 3. Local and Systemic reactogenicity will also be assessed by the participant using the Memory Aid on study days 1, 2, and 3.
** See Laboratory Analytical Plan for details
*** Confirmed HIV-1 infection (HIV Ab+ or HIV RNA+) by documentation in the medical records or in-clinic HIV testing;
~ Cervico-vaginal and/or rectal mucosal sampling (optional) on Day 0 must be done prior to IV infusion of IP.

APPENDIX D: LOW RISK CRITERIA

Low risk will be defined as:

1. **SEXUAL BEHAVIORS**

In the **last 12 months** did not:

- Have oral, vaginal or anal intercourse with an HIV-infected partner, or partner who uses injection drugs.
- Gave or receive money, drugs, gifts, or services in exchange for oral, vaginal or anal sex

  AND

  In the **last 6 months** has abstained from penile/anal or penile/vaginal intercourse

  OR

  In the **last 6 months**:

- Had 4 or fewer partners of the opposite birth sex for vaginal and/or anal intercourse, OR

Is MSM (person born male with partner(s) born male) who, in the **last 12 months**:

- Had 2 or fewer MSM partners for anal intercourse and had no unprotected anal sex with MSM, OR
- Had unprotected anal intercourse with only 1 MSM partner, within a monogamous relationship lasting at least 12 months (during which neither partner had any other partners). If the monogamous relationship ended, the participant may then have had protected anal intercourse with 1 other MSM partner (total 2 or fewer partners in the last 12 months).

Is a transgender person, regardless of the point on the transition spectrum, having sex with men (born male) and/or other transgender persons, who in the last 12 months:

- Had 2 or fewer partners for anal or vaginal intercourse, and had no unprotected anal or vaginal sex, OR
- Had unprotected anal or vaginal intercourse sex with 1 partner only within a monogamous relationship lasting at least 12 months (during which neither partner had any other partners). If the monogamous relationship ended, may then have had protected anal or vaginal sex with one other partner (total 2 or fewer partners in the last 12 months).

  AND

  Uses or intends to use condoms in situations which may include penile/anal or penile/vaginal intercourse with new partners of unknown HIV status, occasional partners, partners outside a primary relationship, and/or partners known to have other partners.

2. **NON-SEXUAL BEHAVIORS**

   In the **last 12 months** did not:

- Inject drugs or other substances without a prescription

- Use cocaine, methamphetamine, or excessive alcohol, which in the investigator's judgement, rendered the participant at greater than low risk for acquiring HIV infection

  The investigator's judgement should consider local epidemiologic information about HIV prevalence in the area and community networks.

  A participant is NOT appropriate for inclusion if he/she:

  Acquired an STI (i.e., new infection) in the last 12 months:

- Syphilis
- Gonorrhea
- Non-gonococcal urethritis
- HSV-2
- Chlamydia
- Pelvic inflammatory disease (PID)
- Trichomonas
- Mucopurulent cervicitis
- Epididymitis
- Proctitis
- Lymphogranuloma venereum
- Chancroid
- Hepatitis B

APPENDIX E REFERENCES

Andrade, A., S. L. Rosenkranz, A. R. Cillo, D. Lu, E. S. Daar, J. M. Jacobson, M. Lederman, E. P. Acosta, T. Campbell, J. Feinberg, C. Flexner, J. W. Mellors, D. R. Kuritzkes and A. C. T. G. A. Team (2013). "Three distinct phases of HIV-1 RNA decay in treatment-naive patients receiving raltegravir-based antiretroviral therapy: ACTG A5248." J Infect Dis **208**(6): 884-891.

Bar, K. J., M. C. Sneller, L. J. Harrison, J. S. Justement, E. T. Overton, M. E. Petrone, D. B. Salantes, C. A. Seamon, B. Scheinfeld, R. W. Kwan, G. H. Learn, M. A. Proschan, E. F. Kreider, J. Blazkova, M. Bardsley, E. W. Refsland, M. Messer, K. E. Clarridge, N. B. Tustin, P. J. Madden, K. Oden, S. J. O'Dell, B. Jarocki, A. R. Shiakolas, R. L. Tressler, N. A. Doria-Rose, R. T. Bailer, J. E. Ledgerwood, E. V. Capparelli, R. M. Lynch, B. S. Graham, S. Moir, R. A. Koup, J. R. Mascola, J. A. Hoxie, A. S. Fauci, P. Tebas and T. W. Chun (2016). "Effect of HIV Antibody VRC01 on Viral Rebound after Treatment Interruption." N Engl J Med **375**(21): 2037-2050.

Barouch, D. H. and S. G. Deeks (2014). "Immunologic strategies for HIV-1 remission and eradication." Science **345**(6193): 169-174.

Barouch, D. H., J. B. Whitney, B. Moldt, F. Klein, T. Y. Oliveira, J. Liu, K. E. Stephenson, H. W. Chang, K. Shekhar, S. Gupta, J. P. Nkolola, M. S. Seaman, K. M. Smith, E. N. Borducchi, C. Cabral, J. Y. Smith, S. Blackmore, S. Sanisetty, J. R. Perry, M. Beck, M. G. Lewis, W. Rinaldi, A. K. Chakraborty, P. Poignard, M. C. Nussenzweig and D. R. Burton (2013). "Therapeutic efficacy of potent neutralizing HIV-1-specific monoclonal antibodies in SHIV-infected rhesus monkeys." Nature **503**(7475): 224-228.

Burton, D. R. and J. R. Mascola (2015). "Antibody responses to envelope glycoproteins in HIV-1 infection." Nat Immunol **16**(6): 571-576.

Caskey, M., F. Klein, J. C. Lorenzi, M. S. Seaman, A. P. West, Jr., N. Buckley, G. Kremer, L. Nogueira, M. Braunschweig, J. F. Scheid, J. A. Horwitz, I. Shimeliovich, S. Ben-Avraham, M. Witmer-Pack, M. Platten, C. Lehmann, L. A. Burke, T. Hawthorne, R. J. Gorelick, B. D. Walker, T. Keler, R. M. Gulick, G. Fatkenheuer, S. J. Schlesinger and M. C. Nussenzweig (2015). "Viraemia suppressed in HIV-1-infected humans by broadly neutralizing antibody 3BNC117." Nature **522**(7557): 487-491.

Caskey, M., T. Schoofs, H. Gruell, A. Settler, T. Karagounis, E. F. Kreider, B. Murrell, N. Pfeifer, L. Nogueira, T. Y. Oliveira, G. H. Learn, Y. Z. Cohen, C. Lehmann, D. Gillor, I. Shimeliovich, C. Unson-O'Brien, D. Weiland, A. Robles, T. Kummerle, C. Wyen, R. Levin, M. Witmer-Pack, K. Eren, C. Ignacio, S. Kiss, A. P. West, Jr., H. Mouquet, B. S. Zingman, R. M. Gulick, T. Keler, P. J. Bjorkman, M. S. Seaman, B. H. Hahn, G. Fatkenheuer, S. J. Schlesinger, M. C. Nussenzweig and F. Klein (2017). "Antibody 10-1074 suppresses viremia in HIV-1-infected individuals." Nat Med.

CDC (2014). "CDC. Vital Signs: HIV Diagnosis, Care, and Treatment Among Persons Living with HIV- United States 2011." MMWR **4**(63): 1-6.

Haynes, B. F. and M. J. McElrath (2013). "Progress in HIV-1 vaccine development." Curr Opin HIV AIDS **8**(4): 326-332.

Hessell, A. J., P. Poignard, M. Hunter, L. Hangartner, D. M. Tehrani, W. K. Bleeker, P. W. Parren, P. A. Marx and D. R. Burton (2009). "Effective, low-titer antibody protection against low-dose repeated mucosal SHIV challenge in macaques." Nat Med **15**(8): 951-954.

Hessell, A. J., E. G. Rakasz, P. Poignard, L. Hangartner, G. Landucci, D. N. Forthal, W. C. Koff, D. I. Watkins and D. R. Burton (2009). "Broadly neutralizing human anti-HIV antibody 2G12 is effective in protection against mucosal SHIV challenge even at low serum neutralizing titers." PLoS Pathog **5**(5): e1000433.

Jardine, J., J. P. Julien, S. Menis, T. Ota, O. Kalyuzhniy, A. McGuire, D. Sok, P. S. Huang, S. MacPherson, M. Jones, T. Nieusma, J. Mathison, D. Baker, A. B. Ward, D. R. Burton, L. Stamatatos, D. Nemazee, I. A. Wilson and W. R. Schief (2013). "Rational HIV immunogen design to target specific germline B cell receptors." Science **340**(6133): 711-716.

Ledgerwood, J. E., E. E. Coates, G. Yamshchikov, J. G. Saunders, L. Holman, M. E. Enama, A. DeZure, R. M. Lynch, I. Gordon, S. Plummer, C. S. Hendel, A. Pegu, M. Conan-Cibotti, S. Sitar, R. T. Bailer, S. Narpala, A. McDermott, M. Louder, S. O'Dell, S. Mohan, J. P. Pandey, R. M. Schwartz, Z. Hu, R. A. Koup, E. Capparelli, J. R. Mascola, B. S. Graham and V. R. C. S. Team (2015). "Safety, pharmacokinetics and neutralization of the broadly neutralizing HIV-1 human monoclonal antibody VRC01 in healthy adults." Clin Exp Immunol.

Lynch, R. M., E. Boritz, E. E. Coates, A. DeZure, P. Madden, P. Costner, M. E. Enama, S. Plummer, L. Holman, C. S. Hendel, I. Gordon, J. Casazza, M. Conan-Cibotti, S. A. Migueles, R. Tressler, R. T. Bailer, A. McDermott, S. Narpala, S. O'Dell, G. Wolf, J. D. Lifson, B. A. Freemire, R. J. Gorelick, J. P. Pandey, S. Mohan, N. Chomont, R. Fromentin, T. W. Chun, A. S. Fauci, R. M. Schwartz, R. A. Koup, D. C. Douek, Z. Hu, E. Capparelli, B. S. Graham, J. R. Mascola, J. E. Ledgerwood and V. R. C. S. Team (2015). "Virologic effects of broadly neutralizing antibody VRC01 administration during chronic HIV-1 infection." Sci Transl Med **7**(319): 319ra206.

Moldt, B., E. G. Rakasz, N. Schultz, P. Y. Chan-Hui, K. Swiderek, K. L. Weisgrau, S. M. Piaskowski, Z. Bergman, D. I. Watkins, P. Poignard and D. R. Burton (2012). "Highly potent HIV-specific antibody neutralization in vitro translates into effective protection against mucosal SHIV challenge in vivo." Proc Natl Acad Sci U S A **109**(46): 18921-18925.

Scheid, J. F., J. A. Horwitz, Y. Bar-On, E. F. Kreider, C. L. Lu, J. C. Lorenzi, A. Feldmann, M. Braunschweig, L. Nogueira, T. Oliveira, I. Shimeliovich, R. Patel, L. Burke, Y. Z. Cohen, S. Hadrigan, A. Settler, M. Witmer-Pack, A. P. West, Jr., B. Juelg, T. Keler, T. Hawthorne, B. Zingman, R. M. Gulick, N. Pfeifer, G. H. Learn, M. S. Seaman, P. J. Bjorkman, F. Klein, S. J. Schlesinger, B. D. Walker, B. H. Hahn and M. C. Nussenzweig (2016). "HIV-1 antibody 3BNC117 suppresses viral rebound in humans during treatment interruption." Nature **535**(7613): 556-560.

Scheid, J. F., H. Mouquet, B. Ueberheide, R. Diskin, F. Klein, T. Y. Oliveira, J. Pietzsch, D. Fenyo, A. Abadir, K. Velinzon, A. Hurley, S. Myung, F. Boulad, P. Poignard, D. R. Burton, F. Pereyra, D. D. Ho, B. D. Walker, M. S. Seaman, P. J. Bjorkman, B. T. Chait and M. C. Nussenzweig (2011). "Sequence and structural convergence of broad and potent HIV antibodies that mimic CD4 binding." Science **333**(6049): 1633-1637.

Schoofs, T., F. Klein, M. Braunschweig, E. F. Kreider, A. Feldmann, L. Nogueira, T. Oliveira, J. C. Lorenzi, E. H. Parrish, G. H. Learn, A. P. West, Jr., P. J. Bjorkman, S. J. Schlesinger, M. S. Seaman, J. Czartoski, M. J. McElrath, N. Pfeifer, B. H. Hahn, M. Caskey and M. C. Nussenzweig (2016). "HIV-1 therapy with monoclonal antibody 3BNC117 elicits host immune responses against HIV-1." Science **352**(6288): 997-1001.

Simek, M. D., W. Rida, F. H. Priddy, P. Pung, E. Carrow, D. S. Laufer, J. K. Lehrman, M. Boaz, T. Tarragona-Fiol, G. Miiro, J. Birungi, A. Pozniak, D. A. McPhee, O. Manigart, E. Karita, A. Inwoley, W. Jaoko, J. Dehovitz, L. G. Bekker, P. Pitisuttithum, R. Paris, L. M. Walker, P. Poignard, T. Wrin, P. E. Fast, D. R. Burton and W. C. Koff (2009). "Human immunodeficiency virus type 1 elite neutralizers: individuals with broad and potent neutralizing activity identified by using a high-throughput neutralization assay together with an analytical selection algorithm." J Virol **83**(14): 7337-7348.

Sok, D., K. J. Doores, B. Briney, K. M. Le, K. L. Saye-Francisco, A. Ramos, D. W. Kulp, J. P. Julien, S. Menis, L. Wickramasinghe, M. S. Seaman, W. R. Schief, I. A. Wilson, P. Poignard and D. R. Burton (2014). "Promiscuous glycan site recognition by antibodies to the high-mannose patch of gp120 broadens neutralization of HIV." Sci Transl Med **6**(236): 236ra263.

Sok, D., M. J. van Gils, M. Pauthner, J. P. Julien, K. L. Saye-Francisco, J. Hsueh, B. Briney, J. H. Lee, K. M. Le, P. S. Lee, Y. Hua, M. S. Seaman, J. P. Moore, A. B. Ward, I. A. Wilson, R. W. Sanders and D. R. Burton (2014). "Recombinant HIV envelope trimer selects for quaternary-dependent antibodies targeting the trimer apex." Proc Natl Acad Sci U S A **111**(49): 17624-17629.

UNAIDS (2016). "UNAIDS Fact Sheet November 2016."

Walker LM, B. D. (2010). "Rational antibody-based HIV-1 vaccine design: current approaches and future directions. ." Curr Opin Immunol. **22**(3): 358-366.

Walker, L. M., M. Huber, K. J. Doores, E. Falkowska, R. Pejchal, J. P. Julien, S. K. Wang, A. Ramos, P. Y. Chan-Hui, M. Moyle, J. L. Mitcham, P. W. Hammond, O. A. Olsen, P. Phung, S. Fling, C. H. Wong, S. Phogat, T. Wrin, M. D. Simek, G. P. I. Protocol, W. C. Koff, I. A. Wilson, D. R. Burton and P. Poignard (2011). "Broad neutralization coverage of HIV by multiple highly potent antibodies." Nature **477**(7365): 466-470.

Walker, L. M., S. K. Phogat, P. Y. Chan-Hui, D. Wagner, P. Phung, J. L. Goss, T. Wrin, M. D. Simek, S. Fling, J. L. Mitcham, J. K. Lehrman, F. H. Priddy, O. A. Olsen, S. M. Frey, P. W. Hammond, G. P. I. Protocol, S. Kaminsky, T. Zamb, M. Moyle, W. C. Koff, P. Poignard and D. R. Burton (2009). "Broad and potent neutralizing antibodies from an African donor reveal a new HIV-1 vaccine target." Science **326**(5950): 285-289.

APPENDIX F CTCAE TABLE

CTCAE4.03 Relevant For T002
Common Terminology Criteria for Adverse Events (CTCAE)
Version 4.0 Published: May 28, 2009 (v4.03: June 14, 2010)

U.S. DEPARTMENT OF HEALTH AND HUMAN SERVICES National Institutes of Health National Cancer Institute

Quick Reference

The NCI Common Terminology Criteria for Adverse Events is a descriptive terminology which can be utilized for Adverse Event (AE) reporting. A grading (severity) scale is provided for each AE term.

Components and Organization

SOC

System Organ Class, the highest level of the MedDRA hierarchy, is identified by anatomical or physiological system, etiology, or purpose (e.g., SOC Investigations for laboratory test results). CTCAE terms are grouped by MedDRA Primary SOCs. Within each SOC, AEs are listed and accompanied by descriptions of severity (Grade).

CTCAE Terms

An Adverse Event (AE) is any unfavorable and unintended sign (including an abnormal laboratory finding), symptom, or disease temporally associated with the use of a medical treatment or procedure that may or may not be considered related to the medical treatment or procedure. An AE is a term that is a unique representation of a specific event used for medical documentation and scientific analyses. Each CTCAE v4.0 term is a MedDRA LLT (Lowest Level Term).

Definitions

A brief definition is provided to clarify the meaning of each AE term.

Grades

Grade refers to the severity of the AE. The CTCAE displays Grades 1 through 5 with unique clinical descriptions of severity for each AE based on this general guideline:

Grade 1	Mild; asymptomatic or mild symptoms; clinical or diagnostic observations only; intervention not indicated.
Grade 2	Moderate; minimal, local or noninvasive intervention indicated; limiting age-appropriate instrumental ADL*.
Grade 3	Severe or medically significant but not immediately life-threatening; hospitalization or prolongation of hospitalization indicated; disabling; limiting self care ADL**.

Grade 4	Life-threatening consequences; urgent intervention indicated.
Grade 5	Death related to AE.

A Semi-colon indicates ‘or’ within the description of the grade.

A single dash (-) indicates a grade is not available.

Not all Grades are appropriate for all AEs. Therefore, some AEs are listed with fewer than five options for Grade selection.

Grade 5

Grade 5 (Death) is not appropriate for some AEs and therefore is not an option.

Activities of Daily Living (ADL)

*Instrumental ADL refer to preparing meals, shopping for groceries or clothes, using the telephone, managing money, etc.

**Self care ADL refer to bathing, dressing and undressing, feeding self, using the toilet, taking medications, and not bedridden.

† CTCAE v4.0 incorporates certain elements of the MedDRA terminology. For further details on MedDRA refer to the MedDRA MSSO Web site (http://www.meddramsso.com).

MedDRA v12.0 Code	CTCAE v4.0 SOC	CTCAE v4.0 Term	Grade 1	Grade 2	Grade 3	Grade 4	Grade 5	CTCAE v4.0 AE Term Definition
10001718	Immune system disorders	Allergic reaction	Transient flushing or rash, drug fever <38 degrees C (<100.4 degrees F); intervention not indicated	Intervention or infusion interruption indicated; responds promptly to symptomatic treatment (e.g., antihistamines, NSAIDS, narcotics); prophylactic medications indicated for <=24 hrs	Prolonged (e.g., not rapidly responsive to symptomatic medication and/or brief interruption of infusion); recurrence of symptoms following initial improvement; hospitalization indicated for clinical sequelae (e.g., renal impairment, pulmonary infiltrates)	Life-threatening consequences; urgent intervention indicated	Death	A disorder characterized by an adverse local or general response from exposure to an allergen.
10002218	Immune system disorders	Anaphylaxis	-	-	Symptomatic bronchospasm, with or without urticaria; parenteral intervention indicated; allergy-related edema/angioedema; hypotension	Life-threatening consequences; urgent intervention indicated	Death	A disorder characterized by an acute inflammatory reaction resulting from the release of histamine and histamine-like substances from mast cells, causing a hypersensitivity immune response. Clinically, it presents with breathing difficulty, dizziness, hypotension, cyanosis and loss of consciousness and may lead to death.

MedDRA v12.0 Code	CTCAE v4.0 SOC	CTCAE v4.0 Term	Grade 1	Grade 2	Grade 3	Grade 4	Grade 5	CTCAE v4.0 AE Term Definition
10003239	Musculoskeletal and connective tissue disorders	Arthralgia	Mild pain	Moderate pain; limiting instrumental ADL	Severe pain; limiting self care ADL	-	-	A disorder characterized by a sensation of marked discomfort in a joint.
10008531	General disorders and administration site conditions	Chills	Mild sensation of cold; shivering; chattering of teeth	Moderate tremor of the entire body; narcotics indicated	Severe or prolonged, not responsive to narcotics	-	-	A disorder characterized by a sensation of cold that often marks a physiologic response to sweating after a fever.
10052015	Immune system disorders	Cytokine release syndrome	Mild reaction; infusion interruption not indicated; intervention not indicated	Therapy or infusion interruption indicated but responds promptly to symptomatic treatment (e.g., antihistamines, NSAIDS, narcotics, IV fluids); prophylactic medications indicated for <=24 hrs	Prolonged (e.g., not rapidly responsive to symptomatic medication and/or brief interruption of infusion); recurrence of symptoms following initial improvement; hospitalization indicated for clinical sequelae (e.g., renal impairment, pulmonary infiltrates)	Life-threatening consequences; pressor or ventilatory support indicated	Death	A disorder characterized by nausea, headache, tachycardia, hypotension, rash, and shortness of breath; it is caused by the release of cytokines from the cells.
10013573	Nervous system disorders	Dizziness	Mild unsteadiness or sensation of movement	Moderate unsteadiness or sensation of movement; limiting instrumental ADL	Severe unsteadiness or sensation of movement; limiting self care ADL	-	-	A disorder characterized by a disturbing sensation of lightheadedness, unsteadiness, giddiness, spinning or rocking.
10013963	Respiratory, thoracic and mediastinal disorders	Dyspnea	Shortness of breath with moderate exertion	Shortness of breath with minimal exertion; limiting instrumental ADL	Shortness of breath at rest; limiting self care ADL	Life-threatening consequences; urgent intervention indicated	Death	A disorder characterized by an uncomfortable sensation of difficulty breathing.

MedDRA v12.0 Code	CTCAE v4.0 SOC	CTCAE v4.0 Term	Grade 1	Grade 2	Grade 3	Grade 4	Grade 5	CTCAE v4.0 AE Term Definition
10015218	Skin and subcutaneous tissue disorders	Erythema multiforme	Target lesions covering <10% BSA and not associated with skin tenderness	Target lesions covering 10 - 30% BSA and associated with skin tenderness	Target lesions covering >30% BSA and associated with oral or genital erosions	Target lesions covering >30% BSA; associated with fluid or electrolyte abnormalities; ICU care or burn unit indicated	Death	A disorder characterized by target lesions (a pink-red ring around a pale center).
10016558	General disorders and administration site conditions	Fever	38.0 - 39.0 degrees C (100.4 - 102.2 degrees F)	>39.0 - 40.0 degrees C (102.3 - 104.0 degrees F)	>40.0 degrees C (>104.0 degrees F) for <=24 hrs	>40.0 degrees C (>104.0 degrees F) for >24 hrs	Death	A disorder characterized by elevation of the body's temperature above the upper limit of normal.
10016825	Vascular disorders	Flushing	Asymptomatic; clinical or diagnostic observations only; intervention not indicated	Moderate symptoms; medical intervention indicated; limiting instrumental ADL	Symptomatic, associated with hypotension and/or tachycardia; limiting self care ADL	-	-	A disorder characterized by episodic reddening of the face.
10019211	Nervous system disorders	Headache	Mild pain	Moderate pain; limiting instrumental ADL	Severe pain; limiting self care ADL	-	-	A disorder characterized by a sensation of marked discomfort in various parts of the head, not confined to the area of distribution of any nerve.

MedDRA v12.0 Code	CTCAE v4.0 SOC	CTCAE v4.0 Term	Grade 1	Grade 2	Grade 3	Grade 4	Grade 5	CTCAE v4.0 AE Term Definition
10020772	Vascular disorders	Hypertension	Prehypertension (systolic BP 120 - 139 mm Hg or diastolic BP 80 - 89 mm Hg)	Stage 1 hypertension (systolic BP 140 - 159 mm Hg or diastolic BP 90 - 99 mm Hg); medical intervention indicated; recurrent or persistent (>=24 hrs); symptomatic increase by >20 mm Hg (diastolic) or to >140/90 mm Hg if previously WNL; monotherapy indicated	Stage 2 hypertension (systolic BP >=160 mm Hg or diastolic BP >=100 mm Hg); medical intervention indicated; more than one drug or more intensive therapy than previously used indicated	Life-threatening consequences (e.g., malignant hypertension, transient or permanent neurologic deficit, hypertensive crisis); urgent intervention indicated	Death	A disorder characterized by a pathological increase in blood pressure; a repeatedly elevation in the blood pressure exceeding 140 over 90 mm Hg.
10021097	Vascular disorders	Hypotension	Asymptomatic, intervention not indicated	Non-urgent medical intervention indicated	Medical intervention or hospitalization indicated	Life-threatening and urgent intervention indicated	Death	A disorder characterized by a blood pressure that is below the normal expected for an individual in a given environment.
10051792	General disorders and administration site conditions	Infusion related reaction	Mild transient reaction; infusion interruption not indicated; intervention not indicated	Therapy or infusion interruption indicated but responds promptly to symptomatic treatment (e.g., antihistamines, NSAIDS, narcotics, IV fluids); prophylactic medications indicated for <=24 hrs	Prolonged (e.g., not rapidly responsive to symptomatic medication and/or brief interruption of infusion); recurrence of symptoms following initial improvement; hospitalization indicated for clinical sequelae	Life-threatening consequences; urgent intervention indicated	Death	A disorder characterized by adverse reaction to the infusion of pharmacological or biological substances.

MedDRA v12.0 Code	CTCAE v4.0 SOC	CTCAE v4.0 Term	Grade 1	Grade 2	Grade 3	Grade 4	Grade 5	CTCAE v4.0 AE Term Definition
10064774	General disorders and administration site conditions	Infusion site extravasation	-	Erythema with associated symptoms (e.g., edema, pain, induration, phlebitis)	Ulceration or necrosis; severe tissue damage; operative intervention indicated	Life-threatening consequences; urgent intervention indicated	Death	A disorder characterized by leakage of a pharmacologic or a biologic substance from the infusion site into the surrounding tissue. Signs and symptoms include induration, erythema, swelling, burning sensation and marked discomfort at the infusion site.
10022095	General disorders and administration site conditions	Injection site reaction	Tenderness with or without associated symptoms (e.g., warmth, erythema, itching)	Pain; lipodystrophy; edema; phlebitis	Ulceration or necrosis; severe tissue damage; operative intervention indicated	Life-threatening consequences; urgent intervention indicated	Death	A disorder characterized by an intense adverse reaction (usually immunologic) developing at the site of an injection.
10025482	General disorders and administration site conditions	Malaise	Uneasiness or lack of well being	Uneasiness or lack of well being; limiting instrumental ADL	-	-	-	A disorder characterized by a feeling of general discomfort or uneasiness, an out-of-sorts feeling.
10028411	Musculoskeletal and connective tissue disorders	Myalgia	Mild pain	Moderate pain; limiting instrumental ADL	Severe pain; limiting self care ADL	-	-	A disorder characterized by marked discomfort sensation originating from a muscle or group of muscles.

MedDRA v12.0 Code	CTCAE v4.0 SOC	CTCAE v4.0 Term	Grade 1	Grade 2	Grade 3	Grade 4	Grade 5	CTCAE v4.0 AE Term Definition
10028813	Gastrointestinal disorders	Nausea	Loss of appetite without alteration in eating habits	Oral intake decreased without significant weight loss, dehydration or malnutrition	Inadequate oral caloric or fluid intake; tube feeding, TPN, or hospitalization indicated	-	-	A disorder characterized by a queasy sensation and/or the urge to vomit.
10033371	General disorders and administration site conditions	Pain	Mild pain	Moderate pain; limiting instrumental ADL	Severe pain; limiting self care ADL	-	-	A disorder characterized by the sensation of marked discomfort, distress or agony.
10033557	Cardiac disorders	Palpitations	Mild symptoms; intervention not indicated	Intervention indicated	-	-	-	A disorder characterized by an unpleasant sensation of irregular and/or forceful beating of the heart.
10037087	Skin and subcutaneous tissue disorders	Pruritus	Mild or localized; topical intervention indicated	Intense or widespread; intermittent; skin changes from scratching (e.g., edema, papulation, excoriations, lichenification, oozing/crusts); oral intervention indicated; limiting instrumental ADL	Intense or widespread; constant; limiting self care ADL or sleep; oral corticosteroid or immunosuppressive therapy indicated	-	-	A disorder characterized by an intense itching sensation.

MedDRA v12.0 Code	CTCAE v4.0 SOC	CTCAE v4.0 Term	Grade 1	Grade 2	Grade 3	Grade 4	Grade 5	CTCAE v4.0 AE Term Definition
10037868	Skin and subcutaneous tissue disorders	Rash maculo-papular	Macules/papules covering <10% BSA with or without symptoms (e.g., pruritus, burning, tightness)	Macules/papules covering 10 - 30% BSA with or without symptoms (e.g., pruritus, burning, tightness); limiting instrumental ADL	Macules/papules covering >30% BSA with or without associated symptoms; limiting self care ADL	-	-	A disorder characterized by the presence of macules (flat) and papules (elevated). Also known as morbillform rash, it is one of the most common cutaneous adverse events, frequently affecting the upper trunk, spreading centripetally and associated with pruritis.

MedDRA v12.0 Code	CTCAE v4.0 SOC	CTCAE v4.0 Term	Grade 1	Grade 2	Grade 3	Grade 4	Grade 5	CTCAE v4.0 AE Term Definition
10040400	Immune system disorders	Serum sickness	Asymptomatic; clinical or diagnostic observations only; intervention not indicated	Moderate arthralgia; fever, rash, urticaria, antihistamines indicated	Severe arthralgia or arthritis; extensive rash; steroids or IV fluids indicated	Life-threatening consequences; pressor or ventilatory support indicated	Death	A disorder characterized by a delayed-type hypersensitivity reaction to foreign proteins derived from an animal serum. It occurs approximately six to twenty-one days following the administration of the foreign antigen. Symptoms include fever, arthralgias, myalgias, skin eruptions, lymphadenopathy, chest marked discomfort and dyspnea.
10051837	Skin and subcutaneous tissue disorders	Skin induration	Mild induration, able to move skin parallel to plane (sliding) and perpendicular to skin (pinching up)	Moderate induration, able to slide skin, unable to pinch skin; limiting instrumental ADL	Severe induration, unable to slide or pinch skin; limiting joint movement or orifice (e.g., mouth, anus); limiting self care ADL	Generalized; associated with signs or symptoms of impaired breathing or feeding	Death	A disorder characterized by an area of hardness in the skin.
10042241	Respiratory, thoracic and mediastinal disorders	Stridor	-	-	Respiratory distress limiting self care ADL; medical intervention indicated	Life-threatening airway compromise; urgent intervention indicated (e.g., tracheotomy or intubation)	Death	A disorder characterized by a high pitched breathing sound due to laryngeal or upper airway obstruction.

MedDRA v12.0 Code	CTCAE v4.0 SOC	CTCAE v4.0 Term	Grade 1	Grade 2	Grade 3	Grade 4	Grade 5	CTCAE v4.0 AE Term Definition
10046735	Skin and subcutaneous tissue disorders	Urticaria	Urticarial lesions covering <10% BSA; topical intervention indicated	Urticarial lesions covering 10 - 30% BSA; oral intervention indicated	Urticarial lesions covering >30% BSA; IV intervention indicated	-	-	A disorder characterized by an itchy skin eruption characterized by wheals with pale interiors and well-defined red margins.
10047700	Gastrointestinal disorders	Vomiting	1 - 2 episodes (separated by 5 minutes) in 24 hrs	3 - 5 episodes (separated by 5 minutes) in 24 hrs	>=6 episodes (separated by 5 minutes) in 24 hrs; tube feeding, TPN or hospitalization indicated	Life-threatening consequences; urgent intervention indicated	Death	A disorder characterized by the reflexive act of ejecting the contents of the stomach through the mouth.

APPENDIX G: DAIDS ADVERSE EVENT SEVERITY ASSESSMENT TABLE

Division of AIDS (DAIDS) Table for Grading the Severity of Adult and Pediatric Adverse Events

Version 2.0
November 2014

Division of AIDS
National Institute of Allergy and Infectious Diseases
National Institutes of Health
US Department of Health and Human Services

TABLE OF CONTENTS

Glossary and Acronyms 1
Introduction 3
Instructions for Use 4
Major Clinical Conditions 7

Cardiovascular 7
Dermatologic 9
Endocrine and Metabolic 10
Gastrointestinal 11
Musculoskeletal 13
Neurologic 14
Pregnancy, Puerperium, and Perinatal 16
Psychiatric 17
Respiratory 18
Sensory 19
Systemic 20
Urinary 22
Site Reactions to Injections and Infusions 23
Laboratory Values 24
Chemistries 24
Hematology 27
Urinalysis 29
Appendix A. Total Bilirubin Table for Term and Preterm Neonates 30

Glossary and Acronyms

AE	Adverse event; Any unfavorable and unintended sign (including an abnormal laboratory finding), symptom, or disease temporally associated with the use of a medical treatment or procedure regardless of whether it is considered related to the medical treatment or procedure.
ALT (SGPT)	Alanine aminotransferase (serum glutamic pyruvic transaminase)
ANC	Absolute neutrophil count

AST (SGOT)	Aspartate aminotransferase (serum glutamic-oxaloacetic transaminase)
AV	Atrioventricular
Basic Self-care Functions	Adult<br>Activities such as bathing, dressing, toileting, transfer or movement, continence, and feeding.<br><br>Young Children<br>Activities that are age and culturally appropriate, such as feeding one's self with culturally appropriate eating implements.
BMI z-score	Body mass index z- score; A body reference norm. Specifically, the number of standard deviations a participant's BMI differs from the average BMI for their age, sex, and ethnicity.
BMD t-score	Bone mineral density t-score; The number of standard deviations above or below the mean bone mineral density of a healthy 30 year old adult of the same sex and ethnicity as the participant.
BMD z-score	Bone mineral density z-score; The number of standard deviations a participant's BMD differs from the average BMD for their age, sex, and ethnicity.
BPAP	Bilevel positive airway pressure; A mode used during noninvasive positive pressure ventilation.
Chemical Pregnancy	A pregnancy in which a positive pregnancy test is followed by a negative pregnancy test without evidence of a clinical pregnancy loss.
CNS	Central nervous system
CPAP	Continuous positive airway pressure
DAERS	DAIDS Adverse Experience Reporting System; An internet-based system developed for clinical research sites to report Expedited Adverse Events (EAEs) to DAIDS. It facilitates timely EAE report submission and serves as a centralized location for accessing and processing EAE information for reporting purposes.
Disability	A substantial disruption of a person's ability to conduct normal life functions.
ECG	Electrocardiogram
eGFR	Estimated glomerular filtration rate
Hospitalization	Does not include the following hospital admissions: under 24 hours, unrelated to an adverse event (e.g., for labor and delivery, cosmetic surgery, social or administrative for temporary placement [for lack of a place to sleep]), protocol-specified, and for diagnosis or therapy of a condition that existed before the receipt of a study agent and which has not increased in severity or frequency.
INR	International normalized ratio

GLOSSARY AND ACRONYMS

Intervention	Medical, surgical, or other procedures recommended or provided by a healthcare professional for the treatment of an adverse event.
IV	Intravenous
IVIG	Intravenous immune globulin
LDL	Low density lipoprotein
LLN	Lower limit of normal

Life-threatening AE	Any adverse event that places the participant, in the view of the investigator, at immediate risk of death from the reaction when it occurred (i.e., it does not include a reaction that would have caused death if it had occurred in a more severe form).
NA	Not applicable
Participant ID	The identification number assigned to a study participant which is used to track study-related documentation, including any reported AEs.
PR Interval	The interval between the beginning of the P wave and the beginning of the QRS complex of an electrocardiogram that represents the time between the beginning of the contraction of the atria and the beginning of the contraction of the ventricles.
PT	Prothrombin time
PTT	Partial thromboplastin time
QTc Interval	The measure of time between the onset of ventricular depolarization and completion of ventricular repolarization corrected for ventricular rate.
RBC	Red blood cell
SI	Standard international unit
ULN	Upper limit of normal
Usual Social & Functional Activities	Activities which adults and children perform on a routine basis and those which are part of regular activities of daily living, for example:<br><br>Adults<br>Adaptive tasks and desirable activities, such as going to work, shopping, cooking, use of transportation, or pursuing a hobby.<br><br>Young Children<br>Activities that are age and culturally appropriate, such as social interactions, play activities, or learning tasks.
WBC	White blood cell
WHO	World Health Organization
WNL	Within normal limits

INTRODUCTION

The Division of AIDS (DAIDS) oversees clinical trials throughout the world which it sponsors and supports. The clinical trials evaluate the safety and efficacy of therapeutic products, vaccines, and other preventive modalities. Adverse event (AE) data collected during these clinical trials form the basis for subsequent safety and efficacy analyses of pharmaceutical products and medical devices. Incorrect and inconsistent AE severity grading can lead to inaccurate data analyses and interpretation, which in turn can impact the safety and well-being of clinical trial participants and future patients using pharmaceutical products.

The DAIDS AE grading table is a shared tool for assessing the severity of AEs (including clinical and laboratory abnormalities) in participants enrolled in clinical trials. Over the years as scientific knowledge and experience have expanded, revisions to the DAIDS AE grading table have become necessary.

The *Division of AIDS (DAIDS) Table for Grading the Severity of Adult and Pediatric Adverse Events, Version 2.0* replaces the grading table published in 2004 and updated in 2009. In version 2.0, AEs not previously included, but which now are deemed medically important events, are included while other AEs have been removed. Some AE severity grading descriptions have been revised to more appropriately reflect the presentation of these events in clinical settings and their impact on clinical trials. For example, DAIDS performed an extensive literature search and reviews of select DAIDS clinical trial data in revising certain hematology parameters (i.e., hemoglobin, white cell counts, and absolute neutrophil counts). DAIDS also took into consideration the U.S. Food and Drug Administration's guidance regarding the use of local laboratory reference values and ethnic differences among certain healthy adolescent and adult populations in defining parameter limits. Finally, the revised DAIDS AE grading table also contains an updated glossary and acronyms section, an expanded instructions for use section, and an appendix that provides more age-specific information for an AE of concern to DAIDS.

DAIDS is grateful to the DAIDS Grading Table Working Group, numerous government and non-government affiliated medical subject matter experts and reviewers who were instrumental in the revision of the DAIDS AE grading table.

Instructions for Use

General Considerations

The *Division of AIDS (DAIDS) Table for Grading the Severity of Adult and Pediatric Adverse Events, Version 2.0* consists of parameters, or AEs, with severity grading guidance that are to be used in DAIDS clinical trials for safety data reporting to maintain accuracy and consistency in the evaluation of AEs. The term "severe" is not the same as the term "serious" in classifying

AEs. The severity of a specific event describes its intensity, and it is the intensity which is graded. Seriousness, which is not graded, relates to an outcome of an AE and is a regulatory definition.

Clinical sites are encouraged to report parameters in the DAIDS AE grading table as they are written to maintain data consistency across clinical trials. However, since some parameters can be reported with more specificity, clinical sites are encouraged to report parameters that convey additional clinical information. For example, diarrhea could be reported as neonatal diarrhea; seizures, as febrile seizures; and pain, as jaw pain.

The DAIDS AE grading table provides an AE severity grading scale ranging from grades 1 to 5 with descriptions for each AE based on the following general guidelines:

- Grade 1 indicates a mild event
- Grade 2 indicates a moderate event
- Grade 3 indicates a severe event
- Grade 4 indicates a potentially life-threatening event
- Grade 5 indicates death (*Note*: This grade is not specifically listed on each page of the grading table).

Other points to consider include:

- Use parameters defined by age and sex values as applicable.
- Male and female sex are defined as sex at birth.
- Unless noted, laboratory values are for term neonates. Preterm neonates should be assessed using local laboratory normal ranges.
- Where applicable, Standard International (SI) units are included in italics.

Selecting and Reporting a Primary AE Term
When selecting a primary AE term to report, sites should select the term that best describes what occurred to the participant. For example, a participant may present with itching, urticaria, flushing, angioedema of the face, and dyspnea. If the underlying diagnosis is determined to be an acute allergic reaction, sites should report "Acute Allergic Reaction" as the primary AE term.

Primary AE terms should be reported using the DAIDS Adverse Experience Reporting System (DAERS) only if they meet expedited reporting criteria. However, all primary AE terms should be reported using protocol-specific case report forms (CRFs). Because the reported information is stored in different databases (i.e., safety and clinical), sites should report primary AE terms using the same terminology for data consistency.

INSTRUCTIONS FOR USE

When reporting using DAERS, other clinically significant events associated with a primary AE term that more fully describe the nature, severity, or complications of the primary AE term should be entered in the "Other Events" section. However, the severity grade for these events must be lower than or equal to the severity grade of the primary AE term. In the example above, dyspnea and angioedema of the face may be entered in the "Other Events" section, because they are more descriptive and provide additional information on the severity of the acute allergic

reaction. However, their severity grades must be lower than or equal to the severity grade of the primary AE term of "Acute Allergic Reaction".

Differences exist in the reporting and recording of information (e.g., signs and symptoms, clinically significant events) in DAERS and CRFs. Therefore, sites should refer to their protocols and CRF requirements for further instructions.

Grading Adult and Pediatric AEs
When a single parameter is not appropriate for grading an AE in both adult and pediatric populations, separate parameters with specified age ranges are provided. If no distinction between adult and pediatric populations has been made, the listed parameter should be used for grading an AE in both populations.

Reporting Pregnancy Outcomes
In the *Pregnancy, Puerperium, and Perinatal* section, all parameters are pregnancy outcomes and should be reported using the mother's participant ID. If an infant is not enrolled in the same study as the mother, any identified birth defects should be reported using the mother's participant ID. However, if an infant is enrolled in the same study as the mother or in another study, any identified birth defects should be reported using the infant's participant ID. Sites should refer to the applicable network standards for reporting abnormal pregnancy outcomes on the CRFs.

Determining Severity Grade for Parameters between Grades
If the severity of an AE could fall in either one of two grades (i.e., the severity of an AE could be either grade 2 or grade 3), sites should select the higher of the two grades.

Laboratory Values
General. An asymptomatic, abnormal laboratory finding without an accompanying AE should not be reported to DAIDS in an expedited timeframe unless it meets protocol-specific reporting requirements. Sites should refer to the applicable network standards for reporting abnormal laboratory findings on the CRFs.

Values below Grade 1. Any laboratory value that is between the ULN and grade 1 (for high values) or the LLN and grade 1 (for low values) should not be graded or reported as an AE. Sites should consult the *Manual for Expedited Reporting of Adverse Events to DAIDS, Version 2.0* and their protocol when making an assessment of the need to report an AE.

Overlap of Local Laboratory Normal Values with Grading Table Ranges. When local laboratory normal values fall within grading table laboratory ranges, the severity grading is based on the ranges in the grading table unless there is a protocol-specific grading criterion for the laboratory

INSTRUCTIONS FOR USE

value. For example, "Magnesium, Low" has a grade 1 range of 1.2 to < 1.4 mEq/L, while a particular laboratory's normal range for magnesium may be 1.3 to 2.8 mEq/L. If a study participant's magnesium laboratory value is 1.3 mEq/L, the laboratory value should be graded as grade 1.

Appendix Usage

MAJOR CLINICAL CONDITIONS
CARDIOVASCULAR

Appendix A takes priority over the main grading table in all assessments of total bilirubin for term and preterm neonates.

Using Addenda 1-3: Grading Tables Used in Microbicide Studies
In protocols involving topical application of products to the female and male genital tracts or rectum, strong consideration should be given to using Addenda 1-3 (see below) as the primary grading tables for these areas. Although these grading tables are used specifically in microbicide studies, they may be used in other protocols as adjuncts to the main grading table (i.e., the *Division of AIDS (AIDS) Table for Grading the Severity of Adult and Pediatric Adverse Events, Version 2.0*). It should be clearly stated in a protocol which addendum is being used as the primary grading table (and thus takes precedence over the main grading table) and which addendum is being used in a complementary fashion.

- Addendum 1 – Female Genital Grading Table for Use in Microbicide Studies – PDF
- Addendum 2 – Male Genital Grading Table for Use in Microbicide Studies – PDF
- Addendum 3 – Rectal Grading Table for Use in Microbicide Studies – PDF

Estimating Severity Grade for Parameters Not Identified in the Grading Table
The functional table below should be used to grade the severity of an AE that is not specifically identified in the grading table. In addition, all deaths related to an AE are to be classified as grade 5.

PARAMETER	GRADE 1 MILD	GRADE 2 MODERATE	GRADE 3 SEVERE	GRADE 4 POTENTIALLY LIFE-THREATENING
**Clinical** adverse event **NOT** identified elsewhere in the grading table	Mild symptoms causing no or minimal interference with usual social & functional activities with intervention not indicated	Moderate symptoms causing greater than minimal interference with usual social & functional activities with intervention indicated	Severe symptoms causing inability to perform usual social & functional activities with intervention or hospitalization indicated	Potentially life-threatening symptoms causing inability to perform basic self-care functions with intervention indicated to prevent permanent impairment, persistent disability, or death

PARAMETER	GRADE 1 MILD	GRADE 2 MODERATE	GRADE 3 SEVERE	GRADE 4 POTENTIALLY LIFE-THREATENING
**Arrhythmia** (by ECG or physical examination)<br>*Specify type, if applicable*	No symptoms AND No intervention indicated	No symptoms AND Non-urgent intervention indicated	Non-life-threatening symptoms AND Non-urgent intervention indicated	Life-threatening arrhythmia OR Urgent intervention indicated
**Blood Pressure Abnormalities**[1]<br>***Hypertension*** *(with the lowest reading taken after repeat testing during a visit)*<br>*≥ 18 years of age*	140 to < 160 mmHg systolic OR 90 to < 100 mmHg diastolic	≥ 160 to < 180 mmHg systolic OR ≥ 100 to < 110 mmHg diastolic	≥ 180 mmHg systolic OR ≥ 110 mmHg diastolic	Life-threatening consequences in a participant not previously diagnosed with hypertension (e.g., malignant hypertension) OR Hospitalization indicated
< 18 years of age	> 120/80 mmHg	≥ 95th to < 99th percentile + 5 mmHg adjusted for age, height, and gender (systolic and/or diastolic)	≥ 99th percentile + 5 mmHg adjusted for age, height, and gender (systolic and/or diastolic)	Life-threatening consequences in a participant not previously diagnosed with hypertension (e.g., malignant hypertension) OR Hospitalization indicated
Hypotension	No symptoms	Symptoms corrected with oral fluid replacement	Symptoms AND IV fluids indicated	Shock requiring use of vasopressors or mechanical assistance to maintain blood pressure
**Cardiac Ischemia or Infarction**<br>*Report only one*	NA	NA	New symptoms with ischemia (stable angina) OR New testing consistent with ischemia	Unstable angina OR Acute myocardial infarction
**Heart Failure**	No symptoms AND Laboratory or cardiac imaging abnormalities	Symptoms with mild to moderate activity or exertion	Symptoms at rest or with minimal activity or exertion (e.g., hypoxemia) OR Intervention indicated (e.g., oxygen)	Life-threatening consequences OR Urgent intervention indicated (e.g., vasoactive medications, ventricular assist device, heart transplant)
**Hemorrhage** (with significant acute blood loss)	NA	Symptoms AND No transfusion indicated	Symptoms AND Transfusion of ≤ 2 units packed RBCs indicated	Life-threatening hypotension OR Transfusion of > 2 units packed RBCs (for children, packed RBCs > 10 cc/kg) indicated

[1] Blood pressure norms for children < 18 years of age can be found in: Expert Panel on Integrated Guidelines for Cardiovascular Health and Risk Reduction in Children and Adolescents. *Pediatrics* 2011;128;S213; originally published online November 14, 2011; DOI: 10.1542/peds.2009-2107C.

CARDIOVASCULAR

PARAMETER	GRADE 1 MILD	GRADE 2 MODERATE	GRADE 3 SEVERE	GRADE 4 POTENTIALLY LIFE-THREATENING
**Prolonged PR Interval or AV Block** *Report only one* *> 16 years of age*	PR interval 0.21 to < 0.25 seconds	PR interval ≥ 0.25 seconds OR Type I 2nd degree AV block	Type II 2nd degree AV block OR Ventricular pause ≥ 3.0 seconds	Complete AV block
≤ 16 years of age	1st degree AV block (PR interval > normal for age and rate)	Type I 2nd degree AV block	Type II 2nd degree AV block OR Ventricular pause ≥ 3.0 seconds	Complete AV block
**Prolonged QTc Interval**[2]	0.45 to 0.47 seconds	> 0.47 to 0.50 seconds	> 0.50 seconds OR ≥ 0.06 seconds above baseline	Life-threatening consequences (e.g., Torsade de pointes, other associated serious ventricular dysrhythmia)
**Thrombosis or Embolism** *Report only one*	NA	Symptoms AND No intervention indicated	Symptoms AND Intervention indicated	Life-threatening embolic event (e.g., pulmonary embolism, thrombus)

[2] As per Bazett's formula.

DERMATOLOGIC

PARAMETER	GRADE 1 MILD	GRADE 2 MODERATE	GRADE 3 SEVERE	GRADE 4 POTENTIALLY LIFE-THREATENING
**Alopecia** (scalp only)	Detectable by study participant, caregiver, or physician AND Causing no or minimal interference with usual social & functional activities	Obvious on visual inspection AND Causing greater than minimal interference with usual social & functional activities	NA	NA
**Bruising**	Localized to one area	Localized to more than one area	Generalized	NA
**Cellulitis**	NA	Non-parenteral treatment indicated (e.g., oral antibiotics, antifungals, antivirals)	IV treatment indicated (e.g., IV antibiotics, antifungals, antivirals)	Life-threatening consequences (e.g., sepsis, tissue necrosis)
**Hyperpigmentation**	Slight or localized causing no or minimal interference with usual social & functional activities	Marked or generalized causing greater than minimal interference with usual social & functional activities	NA	NA
**Hypopigmentation**	Slight or localized causing no or minimal interference with usual social & functional activities	Marked or generalized causing greater than minimal interference with usual social & functional activities	NA	NA
**Petechiae**	Localized to one area	Localized to more than one area	Generalized	NA
**Pruritus**[3] (without skin lesions)	Itching causing no or minimal interference with usual social & functional activities	Itching causing greater than minimal interference with usual social & functional activities	Itching causing inability to perform usual social & functional activities	NA
**Rash** *Specify type, if applicable*	Localized rash	Diffuse rash OR Target lesions	Diffuse rash AND Vesicles or limited number of bullae or superficial ulcerations of mucous membrane limited to one site	Extensive or generalized bullous lesions OR Ulceration of mucous membrane involving two or more distinct mucosal sites OR Stevens-Johnson syndrome OR Toxic epidermal necrolysis

[3] For pruritus associated with injections or infusions, see the *Site Reactions to Injections and Infusions* section (page 23).

Endocrine and Metabolic

PARAMETER	GRADE 1 MILD	GRADE 2 MODERATE	GRADE 3 SEVERE	GRADE 4 POTENTIALLY LIFE-THREATENING
**Diabetes Mellitus**	Controlled without medication	Controlled with medication OR Modification of current medication regimen	Uncontrolled despite treatment modification OR Hospitalization for immediate glucose control indicated	Life-threatening consequences (e.g., ketoacidosis, hyperosmolar non-ketotic coma, end organ failure)
**Gynecomastia**	Detectable by study participant, caregiver, or physician AND Causing no or minimal interference with usual social & functional activities	Obvious on visual inspection AND Causing pain with greater than minimal interference with usual social & functional activities	Disfiguring changes AND Symptoms requiring intervention or causing inability to perform usual social & functional activities	NA
**Hyperthyroidism**	No symptoms AND Abnormal laboratory value	Symptoms causing greater than minimal interference with usual social & functional activities OR Thyroid suppression therapy indicated	Symptoms causing inability to perform usual social & functional activities OR Uncontrolled despite treatment modification	Life-threatening consequences (e.g., thyroid storm)
**Hypothyroidism**	No symptoms AND Abnormal laboratory value	Symptoms causing greater than minimal interference with usual social & functional activities OR Thyroid replacement therapy indicated	Symptoms causing inability to perform usual social & functional activities OR Uncontrolled despite treatment modification	Life-threatening consequences (e.g., myxedema coma)
**Lipoatrophy**[4]	Detectable by study participant, caregiver, or physician AND Causing no or minimal interference with usual social & functional activities	Obvious on visual inspection AND Causing greater than minimal interference with usual social & functional activities	Disfiguring changes	NA
**Lipohypertrophy**[5]	Detectable by study participant, caregiver, or physician AND Causing no or minimal interference with usual social & functional activities	Obvious on visual inspection AND Causing greater than minimal interference with usual social & functional activities	Disfiguring changes	NA

[4] Definition: A disorder characterized by fat loss in the face, extremities, and buttocks.
[5] Definition: A disorder characterized by abnormal fat accumulation on the back of the neck, breasts, and abdomen.

GASTROINTESTINAL

PARAMETER	GRADE 1 MILD	GRADE 2 MODERATE	GRADE 3 SEVERE	GRADE 4 POTENTIALLY LIFE-THREATENING
**Anorexia**	Loss of appetite without decreased oral intake	Loss of appetite associated with decreased oral intake without significant weight loss	Loss of appetite associated with significant weight loss	Life-threatening consequences OR Aggressive intervention indicated (e.g., tube feeding, total parenteral nutrition)
**Ascites**	No symptoms	Symptoms AND Intervention indicated (e.g., diuretics, therapeutic paracentesis)	Symptoms recur or persist despite intervention	Life-threatening consequences
**Bloating or Distension** *Report only one*	Symptoms causing no or minimal interference with usual social & functional activities	Symptoms causing greater than minimal interference with usual social & functional activities	Symptoms causing inability to perform usual social & functional activities	NA
**Cholecystitis**	NA	Symptoms AND Medical intervention indicated	Radiologic, endoscopic, or operative intervention indicated	Life-threatening consequences (e.g., sepsis, perforation)
**Constipation**	NA	Persistent constipation requiring regular use of dietary modifications, laxatives, or enemas	Obstipation with manual evacuation indicated	Life-threatening consequences (e.g., obstruction)
**Diarrhea** *≥ 1 year of age*	Transient or intermittent episodes of unformed stools OR Increase of ≤ 3 stools over baseline per 24-hour period	Persistent episodes of unformed to watery stools OR Increase of 4 to 6 stools over baseline per 24-hour period	Increase of ≥ 7 stools per 24-hour period OR IV fluid replacement indicated	Life-threatening consequences (e.g., hypotensive shock)
< 1 year of age	Liquid stools (more unformed than usual) but usual number of stools	Liquid stools with increased number of stools OR Mild dehydration	Liquid stools with moderate dehydration	Life-threatening consequences (e.g., liquid stools resulting in severe dehydration, hypotensive shock)
**Dysphagia or Odynophagia** *Report only one and specify location*	Symptoms but able to eat usual diet	Symptoms causing altered dietary intake with no intervention indicated	Symptoms causing severely altered dietary intake with intervention indicated	Life-threatening reduction in oral intake
**Gastrointestinal Bleeding**	Not requiring intervention other than iron supplement	Endoscopic intervention indicated	Transfusion indicated	Life-threatening consequences (e.g., hypotensive shock)

GASTROINTESTINAL

PARAMETER	GRADE 1 MILD	GRADE 2 MODERATE	GRADE 3 SEVERE	GRADE 4 POTENTIALLY LIFE-THREATENING
**Mucositis or Stomatitis** *Report only one and specify location*	Mucosal erythema	Patchy pseudomembranes or ulcerations	Confluent pseudomembranes or ulcerations OR Mucosal bleeding with minor trauma	Life-threatening consequences (e.g., aspiration, choking) OR Tissue necrosis OR Diffuse spontaneous mucosal bleeding
**Nausea**	Transient (< 24 hours) or intermittent AND No or minimal interference with oral intake	Persistent nausea resulting in decreased oral intake for 24 to 48 hours	Persistent nausea resulting in minimal oral intake for > 48 hours OR Rehydration indicated (e.g., IV fluids)	Life-threatening consequences (e.g., hypotensive shock)
**Pancreatitis**	NA	Symptoms with hospitalization not indicated	Symptoms with hospitalization indicated	Life-threatening consequences (e.g., circulatory failure, hemorrhage, sepsis)
**Perforation** (colon or rectum)	NA	NA	Intervention indicated	Life-threatening consequences
**Proctitis**	Rectal discomfort with no intervention indicated	Symptoms causing greater than minimal interference with usual social & functional activities OR Medical intervention indicated	Symptoms causing inability to perform usual social & functional activities OR Operative intervention indicated	Life-threatening consequences (e.g., perforation)
**Rectal Discharge**	Visible discharge	Discharge requiring the use of pads	NA	NA
**Vomiting**	Transient or intermittent AND No or minimal interference with oral intake	Frequent episodes with no or mild dehydration	Persistent vomiting resulting in orthostatic hypotension OR Aggressive rehydration indicated (e.g., IV fluids)	Life-threatening consequences (e.g., hypotensive shock)

MUSCULOSKELETAL

PARAMETER	GRADE 1 MILD	GRADE 2 MODERATE	GRADE 3 SEVERE	GRADE 4 POTENTIALLY LIFE-THREATENING
**Arthralgia**	Joint pain causing no or minimal interference with usual social & functional activities	Joint pain causing greater than minimal interference with usual social & functional activities	Joint pain causing inability to perform usual social & functional activities	Disabling joint pain causing inability to perform basic self-care functions
**Arthritis**	Stiffness or joint swelling causing no or minimal interference with usual social & functional activities	Stiffness or joint swelling causing greater than minimal interference with usual social & functional activities	Stiffness or joint swelling causing inability to perform usual social & functional activities	Disabling joint stiffness or swelling causing inability to perform basic self-care functions
**Myalgia** (generalized)	Muscle pain causing no or minimal interference with usual social & functional activities	Muscle pain causing greater than minimal interference with usual social & functional activities	Muscle pain causing inability to perform usual social & functional activities	Disabling muscle pain causing inability to perform basic self-care functions
**Osteonecrosis**	NA	No symptoms but with radiographic findings AND No operative intervention indicated	Bone pain with radiographic findings OR Operative intervention indicated	Disabling bone pain with radiographic findings causing inability to perform basic self-care functions
**Osteopenia**[6] *≥ 30 years of age*	BMD t-score -2.5 to -1	NA	NA	NA
< 30 years of age	BMD z-score -2 to -1	NA	NA	NA
**Osteoporosis**[6] *≥ 30 years of age*	NA	BMD t-score < -2.5	Pathologic fracture (e.g., compression fracture causing loss of vertebral height)	Pathologic fracture causing life-threatening consequences
< 30 years of age	NA	BMD z-score < -2	Pathologic fracture (e.g., compression fracture causing loss of vertebral height)	Pathologic fracture causing life-threatening consequences

[6] BMD t and z scores can be found in: Kanis JA on behalf of the World Health Organization Scientific Group (2007). Assessment of osteoporosis at the primary health-care level. Technical Report. World Health Organization Collaborating Centre for Metabolic Bone Diseases, University of Sheffield, UK. 2007: Printed by the University of Sheffield.

NEUROLOGIC

PARAMETER	GRADE 1 MILD	GRADE 2 MODERATE	GRADE 3 SEVERE	GRADE 4 POTENTIALLY LIFE-THREATENING
**Acute CNS Ischemia**	NA	NA	Transient ischemic attack	Cerebral vascular accident (e.g., stroke with neurological deficit)
**Altered Mental Status** (for Dementia, see *Cognitive, Behavioral, or Attentional Disturbance* below)	Changes causing no or minimal interference with usual social & functional activities	Mild lethargy or somnolence causing greater than minimal interference with usual social & functional activities	Confusion, memory impairment, lethargy, or somnolence causing inability to perform usual social & functional activities	Delirium OR Obtundation OR Coma
**Ataxia**	Symptoms causing no or minimal interference with usual social & functional activities OR No symptoms with ataxia detected on examination	Symptoms causing greater than minimal interference with usual social & functional activities	Symptoms causing inability to perform usual social & functional activities	Disabling symptoms causing inability to perform basic self-care functions
**Cognitive, Behavioral, or Attentional Disturbance** (includes dementia and attention deficit disorder)<br>*Specify type, if applicable*	Disability causing no or minimal interference with usual social & functional activities OR Specialized resources not indicated	Disability causing greater than minimal interference with usual social & functional activities OR Specialized resources on part-time basis indicated	Disability causing inability to perform usual social & functional activities OR Specialized resources on a full-time basis indicated	Disability causing inability to perform basic self-care functions OR Institutionalization indicated
**Developmental Delay**<br>*< 18 years of age*<br>*Specify type, if applicable*	Mild developmental delay, either motor or cognitive, as determined by comparison with a developmental screening tool appropriate for the setting	Moderate developmental delay, either motor or cognitive, as determined by comparison with a developmental screening tool appropriate for the setting	Severe developmental delay, either motor or cognitive, as determined by comparison with a developmental screening tool appropriate for the setting	Developmental regression, either motor or cognitive, as determined by comparison with a developmental screening tool appropriate for the setting
**Headache**	Symptoms causing no or minimal interference with usual social & functional activities	Symptoms causing greater than minimal interference with usual social & functional activities	Symptoms causing inability to perform usual social & functional activities	Symptoms causing inability to perform basic self-care functions OR Hospitalization indicated OR Headache with significant impairment of alertness or other neurologic function

NEUROLOGIC

PARAMETER	GRADE 1 MILD	GRADE 2 MODERATE	GRADE 3 SEVERE	GRADE 4 POTENTIALLY LIFE-THREATENING
**Neuromuscular Weakness** (includes myopathy and neuropathy) *Specify type, if applicable*	Minimal muscle weakness causing no or minimal interference with usual social & functional activities OR No symptoms with decreased strength on examination	Muscle weakness causing greater than minimal interference with usual social & functional activities	Muscle weakness causing inability to perform usual social & functional activities	Disabling muscle weakness causing inability to perform basic self-care functions OR Respiratory muscle weakness impairing ventilation
**Neurosensory Alteration** (includes paresthesia and painful neuropathy) *Specify type, if applicable*	Minimal paresthesia causing no or minimal interference with usual social & functional activities OR No symptoms with sensory alteration on examination	Sensory alteration or paresthesia causing greater than minimal interference with usual social & functional activities	Sensory alteration or paresthesia causing inability to perform usual social & functional activities	Disabling sensory alteration or paresthesia causing inability to perform basic self-care functions
**Seizures** ***New Onset Seizure*** *≥ 18 years of age*	NA	NA	1 to 3 seizures	Prolonged and repetitive seizures (e.g., status epilepticus) OR Difficult to control (e.g., refractory epilepsy)
< 18 years of age (includes new or pre-existing febrile seizures)	Seizure lasting < 5 minutes with < 24 hours postictal state	Seizure lasting 5 to < 20 minutes with < 24 hours postictal state	Seizure lasting ≥ 20 minutes OR > 24 hours postictal state	Prolonged and repetitive seizures (e.g., status epilepticus) OR Difficult to control (e.g., refractory epilepsy)
Pre-existing Seizure	NA	Increased frequency from previous level of control without change in seizure character	Change in seizure character either in duration or quality (e.g., severity or focality)	Prolonged and repetitive seizures (e.g., status epilepticus) OR Difficult to control (e.g., refractory epilepsy)
**Syncope**	Near syncope without loss of consciousness (e.g., pre-syncope)	Loss of consciousness with no intervention indicated	Loss of consciousness AND Hospitalization or intervention required	NA

PREGNANCY, PUERPERIUM, AND PERINATAL

PARAMETER	GRADE 1 MILD	GRADE 2 MODERATE	GRADE 3 SEVERE	GRADE 4 POTENTIALLY LIFE-THREATENING
**Fetal Death or Stillbirth** (report using mother's participant ID)<br>*Report only one*	NA	NA	Fetal loss occurring at ≥ 20 weeks gestation	NA
**Preterm Delivery**[7] (report using mother's participant [illegible]	Delivery at 34 to < 37 weeks gestational age	Delivery at 28 to < 34 weeks gestational age	Delivery at 24 to < 28 weeks gestational age	Delivery at < 24 weeks gestational age
**Spontaneous Abortion or Miscarriage**[8] (report using mother's participant ID)<br>*Report only one*	Chemical pregnancy	Uncomplicated spontaneous abortion or miscarriage	Complicated spontaneous abortion or miscarriage	NA

[7] Definition: A delivery of a live-born neonate occurring at ≥ 20 to < 37 weeks gestational age.
[8] Definition: A clinically recognized pregnancy occurring at < 20 weeks gestational

PSYCHIATRIC

PARAMETER	GRADE 1 MILD	GRADE 2 MODERATE	GRADE 3 SEVERE	GRADE 4 POTENTIALLY LIFE-THREATENING
**Insomnia**	Mild difficulty falling asleep, staying asleep, or waking up early	Moderate difficulty falling asleep, staying asleep, or waking up early	Severe difficulty falling asleep, staying asleep, or waking up early	NA
**Psychiatric Disorders** (includes anxiety, depression, mania, and psychosis)<br>*Specify disorder*	Symptoms with intervention not indicated OR Behavior causing no or minimal interference with usual social & functional activities	Symptoms with intervention indicated OR Behavior causing greater than minimal interference with usual social & functional activities	Symptoms with hospitalization indicated OR Behavior causing inability to perform usual social & functional activities	Threatens harm to self or others OR Acute psychosis OR Behavior causing inability to perform basic self-care functions
**Suicidal Ideation or Attempt**<br>*Report only one*	Preoccupied with thoughts of death AND No wish to kill oneself	Preoccupied with thoughts of death AND Wish to kill oneself with no specific plan or intent	Thoughts of killing oneself with partial or complete plans but no attempt to do so OR Hospitalization indicated	Suicide attempted

RESPIRATORY

PARAMETER	GRADE 1 MILD	GRADE 2 MODERATE	GRADE 3 SEVERE	GRADE 4 POTENTIALLY LIFE-THREATENING
**Acute Bronchospasm**	Forced expiratory volume in 1 second or peak flow reduced to ≥ 70 to < 80% OR Mild symptoms with intervention not indicated	Forced expiratory volume in 1 second or peak flow 50 to < 70% OR Symptoms with intervention indicated OR Symptoms causing greater than minimal interference with usual social & functional activities	Forced expiratory volume in 1 second or peak flow 25 to < 50% OR Symptoms causing inability to perform usual social & functional activities	Forced expiratory volume in 1 second or peak flow < 25% OR Life-threatening respiratory or Hemodynamic compromise OR Intubation
**Dyspnea or Respiratory Distress**<br>*Report only one*	Dyspnea on exertion with no or minimal interference with usual social & functional activities OR Wheezing OR Minimal increase in respiratory rate for age	Dyspnea on exertion causing greater than minimal interference with usual social & functional activities OR Nasal flaring OR Intercostal retractions OR Pulse oximetry 90 to < 95%	Dyspnea at rest causing inability to perform usual social & functional activities OR Pulse oximetry < 90%	Respiratory failure with ventilator support indicated (e.g., CPAP, BPAP, intubation)

SENSORY

PARAMETER	GRADE 1 MILD	GRADE 2 MODERATE	GRADE 3 SEVERE	GRADE 4 POTENTIALLY LIFE-THREATENING
**Hearing Loss** *≥ 12 years of age*	NA	Hearing aid or intervention not indicated	Hearing aid or intervention indicated	Profound bilateral hearing loss (> 80 dB at 2 kHz and above) OR Non-serviceable hearing (i.e., >50 dB audiogram and <50% speech discrimination)
< 12 years of age (based on a 1, 2, 3, 4, 6 and 8 kHz audiogram)	> 20 dB hearing loss at ≤ 4 kHz	> 20 dB hearing loss at > 4 kHz	> 20 dB hearing loss at ≥ 3 kHz in one ear with additional speech language related services indicated (where available) OR Hearing loss sufficient to indicate therapeutic intervention, including hearing aids	Audiologic indication for cochlear implant and additional speech-language related services indicated (where available)
**Tinnitus**	Symptoms causing no or minimal interference with usual social & functional activities with intervention not indicated	Symptoms causing greater than minimal interference with usual social & functional activities with intervention indicated	Symptoms causing inability to perform usual social & functional activities	NA
**Uveitis**	No symptoms AND Detectable on examination	Anterior uveitis with symptoms OR Medicamylasal intervention indicated	Posterior or pan-uveitis OR Operative intervention indicated	Disabling visual loss in affected eye(s)
**Vertigo**	Vertigo causing no or minimal interference with usual social & functional activities	Vertigo causing greater than minimal interference with usual social & functional activities	Vertigo causing inability to perform usual social & functional activities	Disabling vertigo causing inability to perform basic self-care functions
**Visual Changes** (assessed from baseline)	Visual changes causing no or minimal interference with usual social & functional activities	Visual changes causing greater than minimal interference with usual social & functional activities	Visual changes causing inability to perform usual social & functional activities	Disabling visual loss in affected eye(s)

SYSTEMIC

PARAMETER	GRADE 1 MILD	GRADE 2 MODERATE	GRADE 3 SEVERE	GRADE 4 POTENTIALLY LIFE-THREATENING
**Acute Allergic Reaction**	Localized urticaria (wheals) with no medical intervention indicated	Localized urticaria with intervention indicated OR Mild angioedema with no intervention indicated	Generalized urticaria OR Angioedema with intervention indicated OR Symptoms of mild bronchospasm	Acute anaphylaxis OR Life-threatening bronchospasm OR Laryngeal edema
**Chills**	Symptoms causing no or minimal interference with usual social & functional activities	Symptoms causing greater than minimal interference with usual social & functional activities	Symptoms causing inability to perform usual social & functional activities	NA
**Cytokine Release Syndrome**[9]	Mild signs and symptoms AND Therapy (i.e., antibody infusion) interruption not indicated	Therapy (i.e., antibody infusion) interruption indicated AND Responds promptly to symptomatic treatment OR Prophylactic medications indicated for ≤ 24 hours	Prolonged severe signs and symptoms OR Recurrence of symptoms following initial improvement	Life-threatening consequences (e.g., requiring pressor or ventilator support)
**Fatigue or Malaise** *Report only one*	Symptoms causing no or minimal interference with usual social & functional activities	Symptoms causing greater than minimal interference with usual social & functional activities	Symptoms causing inability to perform usual social & functional activities	Incapacitating symptoms of fatigue or malaise causing inability to perform basic self-care functions
**Fever** (non-axillary temperatures only)	38.0 to < 38.6°C or 100.4 to < 101.5°F	≥ 38.6 to < 39.3°C or ≥101.5 to < 102.7°F	≥ 39.3 to < 40.0°C or ≥ 102.7 to < 104.0°F	≥ 40.0°C or ≥ 104.0°F
**Pain**[10] (not associated with study agent injections and not specified elsewhere) *Specify location*	Pain causing no or minimal interference with usual social & functional activities	Pain causing greater than minimal interference with usual social & functional activities	Pain causing inability to perform usual social & functional activities	Disabling pain causing inability to perform basic self-care functions OR Hospitalization indicated
**Serum Sickness**[11]	Mild signs and symptoms	Moderate signs and symptoms AND Intervention indicated (e.g., antihistamines)	Severe signs and symptoms AND Higher level intervention indicated (e.g., steroids or IV fluids)	Life-threatening consequences (e.g., requiring pressor or ventilator support)

9 Definition: A disorder characterized by nausea, headache, tachycardia, hypotension, rash, and/or shortness of breath.
10 For pain associated with injections or infusions, see the *Site Reactions to Injections and Infusions* section (page 23).
11 Definition: A disorder characterized by fever, arthralgia, myalgia, skin eruptions, lymphadenopathy, marked discomfort, and/or dyspnea.

SYSTEMIC

PARAMETER	GRADE 1 MILD	GRADE 2 MODERATE	GRADE 3 SEVERE	GRADE 4 POTENTIALLY LIFE-THREATENING
**Underweight**[12]<br>*> 5 to 19 years of age*	NA	WHO BMI z-score < -2 to ≤ -3	WHO BMI z-score < -3	WHO BMI z-score < -3 with life-threatening consequences
2 to 5 years of age	NA	WHO Weight-for-height z-score < -2 to ≤-3	WHO Weight-for-height z-score < -3	WHO Weight-for-height z-score < -3 with life-threatening consequences
< 2 years of age	NA	WHO Weight-for-length z-score < -2 to ≤-3	WHO Weight-for-length z-score < -3	WHO Weight-for-length z-score < -3 with life-threatening consequences
**Weight Loss** (excludes postpartum weight loss)	NA	5 to < 9% loss in body weight from baseline	≥ 9 to < 20% loss in body weight from baseline	≥ 20% loss in body weight from baseline OR Aggressive intervention indicated (e.g., tube feeding, total parenteral nutrition)

[12] WHO reference tables may be accesed by clicking the desired age range or by accessing the following URLs: http://www.who.int/growthref/who2007_bmi_for_age/en/ for participants > 5 to 19 years of age and http://www.who.int/childgrowth/standards/chart_catalogue/en/ for those < 5 years of age.

URINARY

PARAMETER	GRADE 1 MILD	GRADE 2 MODERATE	GRADE 3 SEVERE	GRADE 4 POTENTIALLY LIFE-THREATENING
**Urinary Tract Obstruction**	NA	Signs or symptoms of urinary tract obstruction without hydronephrosis or renal dysfunction	Signs or symptoms of urinary tract obstruction with hydronephrosis or renal dysfunction	Obstruction causing life-threatening consequences

SITE REACTIONS TO INJECTIONS AND INFUSIONS

PARAMETER	GRADE 1 MILD	GRADE 2 MODERATE	GRADE 3 SEVERE	GRADE 4 POTENTIALLY LIFE-THREATENING
**Injection Site Pain or Tenderness** *Report only one*	Pain or tenderness causing no or minimal limitation of use of limb	Pain or tenderness causing greater than minimal limitation of use of limb	Pain or tenderness causing inability to perform usual social & functional activities	Pain or tenderness causing inability to perform basic self-care function OR Hospitalization indicated
**Injection Site Erythema or Redness**[13] *Report only one* *> 15 years of age*	2.5 to < 5 cm in diameter OR 6.25 to < 25 cm^2 surface area AND Symptoms causing no or minimal interference with usual social & functional activities	≥ 5 to < 10 cm in diameter OR ≥ 25 to < 100 cm^2 surface area OR Symptoms causing greater than minimal interference with usual social & functional activities	≥ 10 cm in diameter OR ≥ 100 cm^2 surface area OR Ulceration OR Secondary infection OR Phlebitis OR Sterile abscess OR Drainage OR Symptoms causing inability to perform usual social & functional activities	Potentially life-threatening consequences (e.g., abscess, exfoliative dermatitis, necrosis involving dermis or deeper tissue)
≤15 years of age	≤ 2.5 cm in diameter	> 2.5 cm in diameter with < 50% surface area of the extremity segment involved (e.g., upper arm or thigh)	≥ 50% surface area of the extremity segment involved (e.g., upper arm or thigh) OR Ulceration OR Secondary infection OR Phlebitis OR Sterile abscess OR Drainage	Potentially life-threatening consequences (e.g., abscess, exfoliative dermatitis, necrosis involving dermis or deeper tissue)
**Injection Site Induration or Swelling** *Report only one* *> 15 years of age*	Same as for **Injection Site Erythema or Redness,** *> 15 years of age*	Same as for **Injection Site Erythema or Redness,** *> 15 years of age*	Same as for **Injection Site Erythema or Redness,** *> 15 years of age*	Same as for **Injection Site Erythema or Redness,** *> 15 years of age*
≤ 15 years of age	Same as for **Injection Site Erythema or Redness,** *≤ 15 years of age*	Same as for **Injection Site Erythema or Redness,** *≤ 15 years of age*	Same as for **Injection Site Erythema or Redness,** *≤ 15 years of age*	Same as for **Injection Site Erythema or Redness,** *≤ 15 years of age*
**Injection Site Pruritus**	Itching localized to the injection site that is relieved spontaneously or in < 48 hours of treatment	Itching beyond the injection site that is not generalized OR Itching localized to the injection site requiring ≥ 48 hours treatment	Generalized itching causing inability to perform usual social & functional activities	NA

[13] Injection Site Erythema or Redness should be evaluated and graded using the greatest single diameter or measured surface area.

LABORATORY VALUES CHEMISTRIES

PARAMETER	GRADE 1 MILD	GRADE 2 MODERATE	GRADE 3 SEVERE	GRADE 4 POTENTIALLY LIFE-THREATENING
**Acidosis**	NA	pH ≥ 7.3 to < LLN	pH < 7.3 without life-threatening consequences	pH < 7.3 with life-threatening consequences
**Albumin, Low**<br>(g/dL; *g/L*)	3.0 to < LLN<br>*30 to < LLN*	≥ 2.0 to < 3.0<br>*≥ 20 to < 30*	< 2.0<br>*< 20*	NA
**Alkaline Phosphatase, High**	1.25 to < 2.5 x ULN	2.5 to < 5.0 x ULN	5.0 to < 10.0 x ULN	≥ 10.0 x ULN
**Alkalosis**	NA	pH > ULN to ≤ 7.5	pH > 7.5 without life-threatening consequences	pH > 7.5 with life-threatening consequences
**ALT or SGPT, High**<br>*Report only one*	1.25 to < 2.5 x ULN	2.5 to < 5.0 x ULN	5.0 to < 10.0 x ULN	≥ 10.0 x ULN
**Amylase (Pancreatic) or Amylase (Total), High**<br>*Report only one*	1.1 to < 1.5 x ULN	1.5 to < 3.0 x ULN	3.0 to < 5.0 x ULN	≥ 5.0 x ULN
**AST or SGOT, High**<br>*Report only one*	1.25 to < 2.5 x ULN	2.5 to < 5.0 x ULN	5.0 to < 10.0 x ULN	≥ 10.0 x ULN
**Bicarbonate, Low**<br>(mEq/L; *mmol/L*)	16.0 to < LLN<br>*16.0 to < LLN*	11.0 to < 16.0<br>*11.0 to < 16.0*	8.0 to < 11.0<br>*8.0 to < 11.0*	< 8.0<br>*< 8.0*
**Bilirubin**<br>***Direct Bilirubin[14], High***<br>*> 28 days of age*	NA	NA	> ULN	> ULN with life-threatening consequences (e.g., signs and symptoms of liver failure)
≤ 28 days of age	ULN to ≤ 1 mg/dL	> 1 to ≤ 1.5 mg/dL	> 1.5 to ≤ 2 mg/dL	> 2 mg/dL
Total Bilirubin, High<br>*> 28 days of age*	1.1 to < 1.6 x ULN	1.6 to < 2.6 x ULN	2.6 to < 5.0 x ULN	≥ 5.0 x ULN
≤ 28 days of age	See Appendix A. Total Bilirubin for Term and Preterm Neonates	See Appendix A. Total Bilirubin for Term and Preterm Neonates	See Appendix A. Total Bilirubin for Term and Preterm Neonates	See Appendix A. Total Bilirubin for Term and Preterm Neonates
**Calcium, High**<br>(mg/dL; *mmol/L*)<br>*≥ 7 days of age*	10.6 to < 11.5<br>*2.65* to < *2.88*	11.5 to < 12.5<br>*2.88* to < *3.13*	12.5 to < 13.5<br>*3.13* to < *3.38*	≥ 13.5<br>*≥ 3.38*
< 7 days of age	11.5 to < 12.4<br>*2.88* to < *3.10*	12.4 to < 12.9<br>*3.10* to < *3.23*	12.9 to < 13.5<br>*3.23* to < *3.38*	≥ 13.5<br>*≥ 3.38*

[14] Direct bilirubin > 1.5 mg/dL in a participant < 28 days of age should be graded as grade 2, if < 10% of the total bilirubin.

CHEMISTRIES

PARAMETER	GRADE 1 MILD	GRADE 2 MODERATE	GRADE 3 SEVERE	GRADE 4 POTENTIALLY LIFE-THREATENING
**Calcium (Ionized), High** (mg/dL; *mmol/L*)	> ULN to < 6.0<br>*> ULN to < 1.5*	6.0 to < 6.4<br>*1.5 to < 1.6*	6.4 to < 7.2<br>*1.6 to < 1.8*	≥ 7.2<br>*≥ 1.8*
**Calcium, Low** (mg/dL; *mmol/L*)<br>*≥ 7 days of age*	7.8 to < 8.4<br>*1.95* to *< 2.10*	7.0 to < 7.8<br>*1.75* to *< 1.95*	6.1 to < 7.0<br>*1.53* to *< 1.75*	< 6.1<br>*< 1.53*
< 7 days of age	6.5 to < 7.5<br>*1.63* to *< 1.88*	6.0 to < 6.5<br>*1.50* to *< 1.63*	5.50 to < 6.0<br>*1.38* to *< 1.50*	< 5.50<br>*< 1.38*
**Calcium (Ionized), Low** (mg/dL; *mmol/L*)	< LLN to 4.0<br>*< LLN to 1.0*	3.6 to < 4.0<br>*0.9 to < 1.0*	3.2 to < 3.6<br>*0.8 to < 0.9*	< 3.2<br>*< 0.8*
**Cardiac Troponin I, High**	NA	NA	NA	Levels consistent with myocardial infarction or unstable angina as defined by the local laboratory
**Creatine Kinase, High**	3 to < 6 x ULN	6 to < 10 x ULN	10 to < 20 x ULN	≥ 20 x ULN
**Creatinine, High**	1.1 to 1.3 x ULN	> 1.3 to 1.8 x ULN OR Increase of > 0.3 mg/dL above baseline	> 1.8 to < 3.5 x ULN OR Increase of 1.5 to < 2.0 x above baseline	≥ 3.5 x ULN OR Increase of ≥ 2.0 x above baseline
**Creatinine Clearance[15] or eGFR, Low**<br>*Report only one*	NA	< 90 to 60 ml/min or ml/min/1.73 m^2 OR 10 to < 30% decrease from baseline	< 60 to 30 ml/min or ml/min/1.73 m^2 OR ≥ 30 to < 50% decrease from baseline	< 30 ml/min or ml/min/1.73 m^2 OR ≥ 50% decrease from baseline or dialysis needed
**Glucose** (mg/dL; *mmol/L*)<br>***Fasting, High***	110 to 125<br>*6.11 to < 6.95*	> 125 to 250<br>*6.95 to < 13.89*	> 250 to 500<br>*13.89 to < 27.75*	> 500<br>*≥ 27.75*
Nonfasting, High	116 to 160<br>*6.44 to < 8.89*	> 160 to 250<br>*8.89 to < 13.89*	> 250 to 500<br>*13.89 to < 27.75*	> 500<br>*≥ 27.75*
**Glucose, Low** (mg/dL; *mmol/L*)<br>*≥ 1 month of age*	55 to 64<br>*3.05 to 3.55*	40 to < 55<br>*2.22 to < 3.05*	30 to < 40<br>*1.67 to < 2.22*	< 30<br>*< 1.67*
< 1 month of age	50 to 54<br>*2.78 to 3.00*	40 to < 50<br>*2.22 to < 2.78*	30 to < 40<br>*1.67 to < 2.22*	< 30<br>*< 1.67*

**Lactate, High**	ULN to < 2.0 x ULN without acidosis	≥ 2.0 x ULN without acidosis	Increased lactate with pH < 7.3 without life-threatening consequences	Increased lactate with pH < 7.3 with life-threatening consequences

[15] Use the applicable formula (i.e., Cockroft-Gault in mL/min or Schwatrz in mL/min/1.73m^2).

CHEMISTRIES

PARAMETER	GRADE 1 MILD	GRADE 2 MODERATE	GRADE 3 SEVERE	GRADE 4 POTENTIALLY LIFE-THREATENING
**Lipase, High**	1.1 to < 1.5 x ULN	1.5 to < 3.0 x ULN	3.0 to < 5.0 x ULN	≥ 5.0 x ULN
**Lipid Disorders** (mg/dL; *mmol/L*) ***Cholesterol, Fasting, High*** *≥18 years of age*	200 to < 240 *5.18 to < 6.19*	240 to < 300 *6.19 to < 7.77*	≥ 300 *≥ 7.77*	NA
< 18 years of age	170 to < 200 *4.40 to < 5.15*	200 to < 300 *5.15 to < 7.77*	≥ 300 *≥ 7.77*	NA
LDL, Fasting, High *≥18 years of age*	130 to < 160 *3.37 to < 4.12*	160 to < 190 *4.12 to < 4.90*	≥ 190 *≥ 4.90*	NA
> 2 to < 18 years of age	110 to < 130 *2.85 to < 3.34*	130 to < 190 *3.34 to < 4.90*	≥ 190 *≥ 4.90*	NA
Triglycerides, Fasting, High	150 to 300 *1.71 to 3.42*	>300 to 500 *>3.42 to 5.7*	>500 to < 1,000 *>5.7 to 11.4*	> 1,000 *> 11.4*
**Magnesium[16], Low** (mEq/L; *mmol/L*)	1.2 to < 1.4 *0.60 to < 0.70*	0.9 to < 1.2 *0.45 to < 0.60*	0.6 to < 0.9 *0.30 to < 0.45*	< 0.6 *< 0.30*
**Phosphate, Low** (mg/dL; *> 14 years of age*	2.0 to < LLN *0.81 to < LLN*	1.4 to < 2.0 *0.65 to < 0.81*	1.0 to < 1.4 *0.32 to < 0.65*	< 1.0 *< 0.32*
1 to 14 years of age	3.0 to < 3.5 *0.97 to < 1.13*	2.5 to < 3.0 *0.81 to < 0.97*	1.5 to < 2.5 *0.48 to < 0.81*	< 1.5 *< 0.48*
< 1 year of age	3.5 to < 4.5 *1.13 to < 1.45*	2.5 to < 3.5 *0.81 to < 1.13*	1.5 to < 2.5 *0.48 to < 0.81*	< 1.5 *< 0.48*
**Potassium, High** (mEq/L; *mmol/L*)	5.6 to < 6.0 *5.6 to < 6.0*	6.0 to < 6.5 *6.0 to < 6.5*	6.5 to < 7.0 *6.5 to < 7.0*	≥ 7.0 *≥ 7.0*
**Potassium, Low** (mEq/L; *mmol/L*)	3.0 to < 3.4 *3.0 to < 3.4*	2.5 to < 3.0 *2.5 to < 3.0*	2.0 to < 2.5 *2.0 to < 2.5*	< 2.0 *< 2.0*
**Sodium, High** (mEq/L; *mmol/L*)	146 to < 150 *146 to < 150*	150 to < 154 *150 to < 154*	154 to < 160 *154 to < 160*	≥ 160 *≥ 160*
**Sodium, Low** (mEq/L; *mmol/L*)	130 to < 135 *130 to < 135*	125 to < 130 *125 to < 135*	121 to < 125 *121 to < 125*	≤ 120 *≤ 120*
**Uric Acid, High**	7.5 to < 10.0	10.0 to < 12.0	12.0 to < 15.0	≥ 15.0

(mg/dL; *mmol/L*)	*0.45 to < 0.59*	*0.59 to < 0.71*	*0.71 to < 0.89*	≥ *0.89*

[16] To convert a magnesium value from mg/dL to mmol/L, laboratories should multiply by 0.4114.

HEMATOLOGY

PARAMETER	GRADE 1 MILD	GRADE 2 MODERATE	GRADE 3 SEVERE	GRADE 4 POTENTIALLY LIFE-THREATENING
**Absolute CD4+ Count, Low**<br>(cell/mm^3; *cells/L*)<br>*> 5 years of age*<br>*(not HIV infected)*	300 to < 400<br>*300 to < 400*	200 to < 300<br>*200 to < 300*	100 to < 200<br>*100 to < 200*	< 100<br>*< 100*
**Absolute Lymphocyte Count, Low**<br>(cell/mm^3; *cells/L*)<br>*> 5 years of age*<br>*(not HIV infected)*	600 to < 650<br>*0.600 x 10^9 to*<br>*< 0.650 x 10^9*	500 to < 600<br>*0.500 x 10^9 to*<br>*< 0.600 x 10^9*	350 to < 500<br>*0.350 x 10^9 to*<br>*< 0.500 x 10^9*	< 350<br>*< 0.350 x 10^9*
**Absolute Neutrophil Count (ANC), Low**<br>(cells/mm^3; *cells/L*)<br>*> 7 days of age*	800 to 1,000<br>*0.800 x 10^9 to 1.000 x 10^9*	600 to 799<br>*0.600 x 10^9 to 0.799 x 10^9*	400 to 599<br>*0.400 x 10^9 to 0.599 x 10^9*	< 400<br>*< 0.400 x 10^9*
2 to 7 days of age	1,250 to 1,500<br>*1.250 x 10^9 to 1.500 x 10^9*	1,000 to 1,249<br>*1.000 x 10^9 to 1.249 x 10^9*	750 to 999<br>*0.750 x 10^9 to 0.999 x 10^9*	< 750<br>*< 0.750 x 10^9*
≤ *1 day of age*	4,000 to 5,000<br>*4.000 x 10^9 to*<br>*5.000 x 10^9*	3,000 to 3,999<br>*3.000 x 10^9 to 3.999 x 10^9*	1,500 to 2,999<br>*1.500 x 10^9 to 2.999 x 10^9*	< 1,500<br>*< 1.500 x 10^9*
**Fibrinogen, Decreased**<br>(mg/dL; *g/L*)	100 to < 200<br>*1.00 to < 2.00*<br>OR<br>0.75 to < 1.00 x LLN	75 to < 100<br>*0.75 to < 1.00*<br>OR<br>≥ 0.50 to < 0.75 x LLN	50 to < 75<br>*0.50 to < 0.75*<br>OR<br>0.25 to < 0.50 x LLN	< 50<br>*< 0.50*<br>OR<br>< 0.25 x LLN<br>OR Associated with gross bleeding
**Hemoglobin[17], Low**<br>(g/dL; *mmol/L*)[18]<br>≥ *13 years of age*<br>*(male only)*	10.0 to 10.9<br>*6.19 to 6.76*	9.0 to < 10.0<br>*5.57 to < 6.19*	7.0 to < 9.0<br>*4.34 to < 5.57*	< 7.0<br>*< 4.34*
≥ *13 years of age*<br>*(female only)*	9.5 to 10.4<br>*5.88 to 6.48*	8.5 to < 9.5<br>*5.25 to < 5.88*	6.5 to < 8.5<br>*4.03 to < 5.25*	< 6.5<br>*< 4.03*

[17] Male and female sex are defined as sex at birth.
[18] The conversion factor used to convert g/dL to mmol/L is 0.6206 and is the most commonly used conversion factor. For grading hemoglobin results obtained by an analytic method with a conversion factor other than 0.6206, the result must be converted to g/dL using the appropriate conversion factor for the particular laboratory.

HEMATOLOGY

PARAMETER	GRADE 1 MILD	GRADE 2 MODERATE	GRADE 3 SEVERE	GRADE 4 POTENTIALLY LIFE-THREATENING
57 days of age to < 13 years of age (male and female)	9.5 to 10.4<br>*5.88 to 6.48*	8.5 to < 9.5<br>*5.25 to < 5.88*	6.5 to < 8.5<br>*4.03 to < 5.25*	< 6.5<br>*< 4.03*
36 to 56 days of age (male and female)	8.5 to 9.6<br>*5.26 to 5.99*	7.0 to < 8.5<br>*4.32 to < 5.26*	6.0 to < 7.0<br>*3.72 to < 4.32*	< 6.0<br>*< 3.72*
22 to 35 days of age (male and female)	9.5 to 11.0<br>*5.88 to 6.86*	8.0 to < 9.5<br>*4.94 to < 5.88*	6.7 to < 8.0<br>*4.15 to < 4.94*	< 6.7<br>*< 4.15*
8 to ≤ 21 days of age (male and female)	11.0 to 13.0<br>*6.81 to 8.10*	9.0 to < 11.0<br>*5.57 to < 6.81*	8.0 to < 9.0<br>*4.96 to < 5.57*	< 8.0<br>*< 4.96*
≤ 7 days of age (male and female)	13.0 to 14.0<br>*8.05 to 8.72*	10.0 to < 13.0<br>*6.19 to < 8.05*	9.0 to < 10.0<br>*5.59 to < 6.19*	< 9.0<br>*< 5.59*
**INR, High** (not on anticoagulation therapy)	1.1 to < 1.5 x ULN	1.5 to < 2.0 x ULN	2.0 to < 3.0 x ULN	≥ 3.0 x ULN
**Methemoglobin** (% hemoglobin)	5.0 to < 10.0%	10.0 to < 15.0%	15.0 to < 20.0%	≥ 20.0%
**PTT, High** (not on anticoagulation therapy)	1.1 to < 1.66 x ULN	1.66 to < 2.33 x ULN	2.33 to < 3.00 x ULN	≥ 3.00 x ULN
**Platelets, Decreased** (cells/mm^3; *cells/L*)	100,000 to < 124,999<br>*100.000 x 10^9 to < 124.999 x 10^9*	50,000 to < 100,000<br>*50.000 x 10^9 to < 100.000 x 10^9*	25,000 to < 50,000<br>*25.000 x 10^9 to < 50.000 x 10^9*	< 25,000<br>*< 25.000 x 10^9*
**PT, High** (not on anticoagulation therapy	1.1 to < 1.25 x ULN	1.25 to < 1.50 x ULN	1.50 to < 3.00 x ULN	≥ 3.00 x ULN
**WBC, Decreased** (cells/mm^3; *cells/L*)<br>*> 7 days of age*	2,000 to 2,499<br>*2.000 x 10^9 to 2.499 x 10^9*	1,500 to 1,999<br>*1.500 x 10^9 to 1.999 x 10^9*	1,000 to 1,499<br>*1.000 x 10^9 to 1.499 x 10^9*	< 1,000<br>*< 1.000 x 10^9*
≤ 7 days of age	5,500 to 6,999<br>*5.500 x 10^9 to 6.999 x 10^9*	4,000 to 5,499<br>*4.000 x 10^9 to 5.499 x 10^9*	2,500 to 3,999<br>*2.500 x 10^9 to 3.999 x 10^9*	< 2,500<br>*< 2.500 x 10^9*

URINALYSIS

PARAMETER	GRADE 1 MILD	GRADE 2 MODERATE	GRADE 3 SEVERE	GRADE 4 POTENTIALLY LIFE-THREATENING
**Glycosuria** (random collection tested by dipstick)	Trace to 1+ or ≤ 250 mg	2+ or > 250 to < 500 mg	> 2+ or > 500 mg	NA
**Hematuria** (not to be reported based on dipstick findings or on blood believed to be of menstrual origin)	6 to < 10 RBCs per high power field	≥ 10 RBCs per high power field	Gross, with or without clots OR With RBC casts OR Intervention indicated	Life-threatening consequences
**Proteinuria** (random collection tested by dipstick)	1+	2+	3+ or higher	NA

International AIDS Vaccine Initiative

DOCUMENT NUMBER:
DOCUMENT TITLE:
DOCUMENT NOTES:

Document Information

Revision:	Vault:
Status:	Document Type:

Date Information

Effective Date:	Expiration Date:
Release Date:	Next Review Date:

Control Information

Author:	Owner:
Previous Number:	Change Number:

Signature Manifest

Document Number: TMF-02-0248 **Revision:** 1

Title: Protocol PGT121/PGDM1400

All dates and times are in Eastern Time Zone.

T002 Protocol

Change Request Approval

Name/Signature	Title	Date	Meaning/Reason
Carl Verlinde (CVERLINDE)			
Dani Vooijs (DVOOIJS)			
Jeniffer Kigera (JKIGERA)			
Lisa Sunner (LSUNNER)			
Harriet Park (HPARK)	Director Clincal Operations	29 Jun 2017, 03:23:26 PM	Approved

CMO Approval

Name/Signature	Title	Date	Meaning/Reason
Frances Priddy (FPRIDDY)	Chief Medical Officer	30 Jun 2017, 10:33:27 AM	Approved

QA Final Release

Name/Signature	Title	Date	Meaning/Reason
Jeniffer Kigera (JKIGERA)			
Lisa Sunner (LSUNNER)			
Carl Verlinde (CVERLINDE)			
Dani Vooijs (DVOOIJS)			
Harriet Park (HPARK)	Director Clincal Operations	30 Jun 2017, 10:40:00 AM	Approved

Notify

Name/Signature	Title	Date	Meaning/Reason
Lisa Sunner (LSUNNER)		30 Jun 2017, 10:40:01 AM	Email Sent

**Protocol Title:**	A Phase 1 Randomized Placebo-controlled Clinical Trial of the Safety, Pharmacokinetics and Antiviral Activity of PGDM1400 and PGT121 Monoclonal Antibodies in HIV-uninfected and HIV-infected Adults
**Protocol Number:**	IAVI T002
**Regulatory Investigational Product Number**	134270
**ClinicalTrials.gov Registry Number**	TBD
**Phase:**	Phase 1
**Sponsor:**	International AIDS Vaccine Initiative (IAVI)<br>125 Broad Street, 9th Floor<br>New York, New York 10004<br>USA
**Sponsor Status**	Not for-Profit Organization
**Date of Protocol Version:**	07 AUGUST 2017<br>3.0<br><br>29 JUNE 2017<br>2.0<br><br>12 MAY 2017<br>1.0

THE CONFIDENTIAL INFORMATION IN THIS DOCUMENT IS PROVIDED TO YOU AS AN INVESTIGATOR, POTENTIAL INVESTIGATOR, OR CONSULTANT, FOR REVIEW BY YOU, YOUR STAFF, AND APPLICABLE INSTITUTIONAL REVIEW BOARDS (IRBS) AND/OR INDEPENDENT ETHICS COMMITTEES (IECS). IT IS UNDERSTOOD THAT THE INFORMATION WILL NOT BE DISCLOSED TO OTHERS, EXCEPT TO THE EXTENT NECESSARY TO OBTAIN ETHICAL AND REGULATORY APPROVAL FROM THE RESPECTIVE COMMITTEE'S AGENCIES AND INFORMED CONSENT FROM THOSE PERSONS TO WHOM THE INVESTIGATIONAL PRODUCT MAY BE ADMINISTERED.

PROTOCOL SYNOPSIS

**TITLE**	A Phase 1 Randomized Placebo-controlled Clinical Trial of the Safety, Pharmacokinetics and Antiviral Activity of PGDM1400 and PGT121 Monoclonal Antibodies in HIV-uninfected and HIV-infected Adults
**PROTOCOL NUMBER**	IAVI T002
**CLINICAL TRIAL PHASE**	Phase 1
**IND SPONSOR**	International AIDS Vaccine Initiative (IAVI)<br>125 Broad Street, 9th Floor<br>New York, New York 10004,<br>USA
**SPONSOR STATUS**	Not for Profit Organization
**SAMPLE SIZE**	36-84
**STUDY POPULATION**	1. HIV-uninfected males or females aged 18-50 years old who are willing to maintain low risk behavior for HIV infection; principal exclusion criteria include confirmed HIV-infection, pregnancy or lactation, significant acute or chronic disease and clinically significant laboratory abnormalities (Groups 1 and 2, see below for full inclusion and exclusion criteria).<br>2. HIV-infected males or females aged 18-65 years old, not on antiretroviral therapy (ART) with HIV-1 viral load between 1000 and 100,000 copies/ml, CD4 cell count ≥ 300 cells/µl; principal exclusion criteria include significant acute or chronic medical condition other than HIV infection, and clinically significant laboratory abnormalities (Group 3, see below for full inclusion and exclusion criteria).
**STUDY DESIGN**	Double blind, randomized, placebo-controlled study for Groups 1 and 2. Open label study for Group 3. Single intravenous (IV) infusion of PGDM1400 mAb alone @ 3, 10 or 30 mg/kg, or a combination of PGDM1400 mAb and PGT121 mAb, each @ 3, 10 or 30 mg/kg, or placebo. See study design table below for details.
**STUDY DURATION**	Up to 32 weeks per participant, screening up to 42 (HIV-infected) or up to 56 (HIV-uninfected) days before single IV infusion of investigational product on day 0, and 24 weeks of follow up.
**INVESTIGATIONAL PRODUCT DESCRIPTION**	**PGDM1400 mAb**: PGDM1400 mAb is a recombinant, fully human monoclonal antibody (mAb) of the IgG1 isotype that binds to the HIV envelope. The concentration of the product is 50 mg/ml. Vials will contain 6 ml of product @ 50 mg/ml = 300 mg.<br>**PGT121 mAb**: PGT121 mAb is a recombinant, fully human monoclonal antibody (mAb) of the IgG1 isotype that binds to the

	HIV envelope. The concentration of the product is 50 mg/ml. Vials will contain 6 ml of product @ 50 mg/ml = 300 mg.<br>**Placebo:** 0.9% Sodium Chloride for injection, USP
**OBJECTIVES**	**Primary Objectives**<br>• To evaluate the safety and tolerability of IV infusion of PGDM1400 mAb alone, and a combination of PGDM1400 mAb and PGT121 mAb in HIV-uninfected adults and HIV-infected adults<br>• To evaluate the pharmacokinetic (PK) profile of IV infusion of PGDM1400 mAb alone, and a combination of PGDM1400 mAb and PGT121 mAb in HIV-uninfected adults and HIV-infected adults<br>• To evaluate the antiviral activity of IV infusion of PGDM1400 mAb alone, and a combination of PGDM1400 mAb and PGT121 mAb in HIV-infected adults not on ART<br><br>**Secondary Objectives**<br>• To determine if PGDM1400 mAb alone, and in combination with PGT121 mAb induces anti-PGDM1400 and/or anti-PGT121 antibodies<br>• To determine the effect of PGDM1400 mAb alone and in combination with PGT121 mAb on CD4+ T cell counts in HIV-infected adults<br>• To determine the effect of PGDM1400 mAb alone and in combination with PGT121 mAb on viral escape in viremic HIV-infected adults not on ART<br><br>**Exploratory Objectives**<br>• To determine if PGDM1400 ± PGT121 mAb has any impact on the host immune responses (i.e., HIV-specific cellular and humoral immune response).<br>• To determine the effect of PGDM1400 ± PGT121 mAb on the size of the latent HIV reservoir in HIV-infected adults.<br>• To determine PGDM1400 mAb ± PGT121 mAb levels in mucosal secretions in HIV-uninfected and HIV-infected adults.<br>• To measure in vitro neutralization of HIV isolates with participant's serum post PGDM1400 ± PGT121 mAb IV infusion
**ENDPOINTS**	**Primary Endpoints**<br>*Safety and Tolerability*<br>1. Proportion of participants with moderate or greater reactogenicity (e.g., solicited adverse events) for 3 days following IV infusion of PGDM1400 mAb alone, and a combination of PGDM1400 mAb and PGT121 mAb.<br>2. Proportion of participants with adverse events (AEs), including safety laboratory (biochemical, hematological) parameters, during the first 56 days following IV infusion of PGDM1400

mAb alone and a combination of PGDM1400 mAb and PGT121 mAb, that are moderate or greater, and/or related to PGDM1400 mAb or PGT121 mAb.
3. Proportion of participants with serious adverse events (SAEs) throughout the study period following IV infusion of PGDM1400 mAb alone and a combination of PGDM1400 mAb and PGT121 mAb, that are related to PGDM1400 mAb or PGT121 mAb.

Pharmacokinetics
Pharmacokinetics following IV infusion of PGDM1400 mAb alone or a combination of PGDM1400 mAb and PGT121 mAb in HIV-uninfected and HIV-infected adults:
- Elimination half-life (t1/2)
- Clearance (CL/F)
- Volume of distribution (Vz/F)
- Area under the concentration decay curve (AUC)
- Impact of viral load and/or ART on PGDM1400 mAb and PGT121 mAb disposition (elimination half-life (t1/2), clearance (CL/F), volume of distribution (Vz/F), total exposure)

Antiviral activity
Antiviral activity following IV infusion of PGDM1400 mAb alone or in combination with PGT121 mAb in viremic HIV-infected adults not on ART:
1. Change in plasma HIV-1 RNA levels from baseline (mean of pre-entry and entry values)

Secondary Endpoints
Anti-PGDM1400 and anti-PGT121 antibodies
1. Serum anti-PGDM1400 antibody titers
2. Serum anti-PGT121 antibody titers

CD4+ T cell count
We will calculate the following endpoint to determine if IV infusion of PGDM1400 mAb alone or in combination with PGT121 mAb has any impact on CD4+ T cell counts in HIV-infected adults:
1. Change in CD4+ T cell count and frequency compared to baseline as measured by single platform flow cytometry.

HIV genotyping/phenotyping of circulating virus for evaluation of PGDM1400 mAb and/or PGT121 mAb -induced escape mutations
We will compare plasma virus genotype and phenotypic activity before and after IV infusion of PGDM1400 mAb alone or in combination with PGT121 mAb to determine the effect of PGDM1400 mAb and/or PGT121 mAb on viral escape mutations in viremic HIV-infected adults not on ART.
1. Genotypic analysis: Development of sequence variations in

epitopes known to result in reduced PGDM1400 mAb and/or PGT121 mAb neutralization susceptibility or known to cause resistance to antiretroviral drugs.
2. Phenotypic analysis: Changes in viral susceptibility to PGDM1400 mAb and/or PGT121 mAb neutralization.

Exploratory Endpoints
Additional assessments may include but are not limited to the following: HIV-specific IgG/IgA binding responses by ELISA, HIV-specific cellular immune responses by ELISPOT, HIV-specific antibody function by ADCC, ADCP, and ADCVI assays, PGDM1400 mAb and/or PGT121 mAb levels in mucosal secretions, changes in total HIV-1 DNA and 2-long terminal repeat (LTR) circular HIV-1 DNA in resting or total CD4 T cells and in vitro neutralization of HIV isolates with participant's serum post IV infusion of PGDM1400 mAb ± PGT121 mAb.

STUDY DESIGN TABLE

	Group	Participants	Sub-Group	Regimen	N	Dose (mg/kg)
Part 1 – MTD	1	HIV-uninfected participants	1A	PGDM1400/Placebo	3/1 (6/2 if DLT)	3 IV
			1B	PGDM1400/Placebo	3/1 (6/2 if DLT)	10 IV
			1C	PGDM1400/Placebo	3/1 (6/2 if DLT)	30 IV
				Total Group 1	9/3 = 12 (max 18/6 = 24 if DLT)	
	2	HIV-uninfected participants	2A	PGDM1400 + PGT121/Placebo	3/1 (6/2 if DLT)	3 + 3 IV
			2B	PGDM1400 + PGT121/Placebo	3/1 (6/2 if DLT)	10 + 10 IV
			2C	PGDM1400 + PGT121/Placebo	3/1 (6/2 if DLT)	30 + 30 IV
				Total Group 2	9/3 = 12 (max 18/6 = 24 if DLT)	
				Total Groups 1 and 2	18/6 = 24 (max 36/12 = 48 if DLT)	
Safety Monitoring Committee						
Part 2 – antiviral effect	3	HIV-infected off ART (VL $1x10^3$ – $1x10^5$ copies/ml)	3A	PGDM1400	6 (max 18)	MTD IV
			3B	PGDM1400+PGT121	6 (max 18)	MTD IV
				Total Group 3	12 (max 36)	
				Total entire study	36 (max 84)	

DLT, dose limiting toxicity; MTD, maximum tolerated dose

**SAFETY MONITORING**	Safety will continually be monitored by the Investigators, the Sponsor's Medical Monitor and a Protocol Safety Review Team (PSRT). Safety data will be reviewed by an independent Safety Monitoring Committee (SMC).<br><br>- *Sentinel participants in Groups 1 and 2*<br>For each dose Subgroup in Groups 1 and 2, the 1st 2 participants will be sentinel participants for whom investigational product infusion will be separated by at least 24 hours contingent on review of safety information prior to infusion of the next sentinel participant. Both sentinel participants are observed for at least 24 hours before IV infusion of investigational product to the 3rd and subsequent participants.<br><br>- *Dose limiting toxicity in Groups 1 and 2*<br>Dose limiting toxicity (DLT) will be defined as 1) any Grade 3 or greater adverse event considered possibly, probably or definitely related to investigational product or 2) any Grade 3 or greater reactogenicity or 3) any SAE considered possibly, probably or definitely related to investigational product.<br><br>- *Infusion related reactions*<br>Infusion related reactions, including cytokine release syndrome, will be graded using the NCI Common Terminology Criteria for Adverse Events (CTCAE) version 4.03 (June 14, 2010). All other adverse events will be graded using the Division of AIDS (DAIDS) Table for Grading the Severity of Adult and Pediatric Adverse Events version 2.0 (November 2014).
**DOSE ESCALATION IN GROUPS 1 AND 2**	Part 1 of this study is a dose-escalation trial in HIV-uninfected adults (Groups 1 and 2) to establish the MTD of PGDM1400 mAb and a combination of PGDM1400 mAb and PGT121 mAb.<br><br>- *PSRT review for dose escalation*<br>The Protocol Safety Review Team (PSRT) will review safety data through day 14 post-investigational product infusion for all participants in each dose Subgroup for PGDM1400 mAb alone (Group 1) prior to allowing enrolment of participants into the next higher dose Subgroup in Group 1 or the same dose level PGDM1400 mAb and PGT121 mAb combination dose Subgroup (Group 2).<br>o If no DLT occurs in the initial 4 participants of a dose Subgroup (e.g., 1A), the study can proceed with enrolment of the next dose Subgroup for PGDM1400 mAb alone (e.g., 1B), and the same dose level PGDM1400 mAb and PGT121 mAb combination dose Subgroup (e.g., 2A).<br>o If 1 DLT occurs in the initial 4 participants of a dose Subgroup (e.g., 1A), 4 additional participants will be enrolled in the same dose Subgroup.<br>▪ If no additional DLTs occur within 14 days of infusion in

	the 8 total participants, the study can proceed with enrolment of the next dose Subgroup for PGDM1400 mAb alone (e.g., 1B), and the same dose level PGDM1400 mAb and PGT121 mAb combination dose Subgroup (e.g., 2A).<br>▪ In Subgroups receiving PGDM1400 alone (1A, 1B, 1C), if 2 or more DLTs accumulate in a dose Subgroup (e.g., 1B) that are the same, similar, or in the same System Organ Class, infusion will be halted and the next lower dose level will be declared the maximum tolerated dose (MTD) within this Group (e.g., 3 mg/kg if the DLTs occurred in dose Subgroup 1B @ 10 mg/kg) , and the same dose level PGDM1400 mAb and PGT121 mAb combination dose Subgroup (2A in this example). Group 2 will not proceed with escalation beyond the MTDs of PGDM1400 alone and PGT121 alone.<br>▪ If no DLT occurs in the final dose Subgroup (1C) after 14 days of follow-up, the MTD for PGDM1400 alone will be the highest dose given (30mg/kg).<br>▪ In Subgroups receiving PGDM1400 and PGT121 (2A, 2B, 2C), if 2 or more DLTs occur in a dose Subgroup (e.g., 2B) that are the same, similar, or in the same System Organ Class, infusion will be halted and the next lower dose level will be declared the MTD for the combination of PGDM1400 and PGT121.<br>▪ If no DLT occurs in the final dose Subgroup (2C) after 14 days of follow-up , MTD for PGDM1400 and PGT121 will be the highest dose given (30mg/kg each).<br><br>- *SMC review to determine MTD of PGDM1400 alone*<br>Following IV infusion of investigational product in the last participant in Group 1, an independent Safety Monitoring Committee (SMC) will review safety data through day 14 post-investigational product infusion for all participants to confirm MTD of PGDM1400 alone, and determine whether, and at what dose, Group 3A can initiate enrolment.<br><br>- *SMC review to determine MTD of the combination of PGDM1400 and PGT121*<br>Following IV infusion of investigational product in the last participant in Group 2, the SMC will review safety data through day 14 post-investigational product infusion for all participants to confirm MTD of the combination of PGDM1400 and PGT121, and determine whether Group 3B can initiate enrollment.
**ANTIVIRAL ACTIVITY IN GROUP 3**	Part 2 of this study will establish the antiviral effect of PGDM1400 mAb alone or the combination of PGDM1400 mAb plus PGT121 mAb in HIV infected adults not on ART (Group 3).<br><br>- *MTD determines dose*

	Group 3 will start with the MTD of PGDM1400 as determined by the SMC; e.g., if the MTD for PGDM1400 is 30mg/kg then Subgroup 3A will receive 30mg/kg and Subgroup 3B will receive PGDM1400 mAb + PGT121 mAb at a dose that combines the MTD for PGDM1400 mAb and the MTD for PGT121 mAb, respectively.
**PAUSE RULES**	The study will be paused for a safety review by the investigators and the independent SMC if:<br>1. 1 or more participants experience a Serious Adverse Event that is judged possibly, probably or definitely related to the investigational product.<br>2. There is a participant death regardless of relationship to the investigational product.<br>3. If 2 or more participants experience Grade 3 adverse events in the same System Organ Class that are considered possibly, probably, or definitely related to investigational product.<br>4. Any Grade 4 adverse event that is considered possibly, probably or definitely related to investigational product.
**EVALUATION FOR INTERCURRENT HIV INFECTION:**	Participants in Groups 1 and 2 (HIV-uninfected) will be tested for HIV according to the Schedule of Procedures. Test results will be interpreted according to a pre-determined diagnostic algorithm. HIV testing at additional time points may be performed upon the request of the participant and Principal Investigator or designee as medical or social circumstances warrant.
**INCLUSION CRITERIA**	**Inclusion criteria for all participants:**<br>1. Willing to comply with the requirements of the protocol and available for follow-up for the planned duration of the study.<br>2. In the opinion of the Principal Investigator or designee and based on Assessment of Informed Consent Understanding results, has understood the information provided and potential impact and/or risks linked to IV infusion and participation in the trial; written informed consent will be obtained from the participant before any study-related procedures are performed.<br>3. All heterosexually active female participants must commit to use an effective method of contraception for 3 months following investigational product administration, including:<br>a. Condoms (male or female) with or without spermicide<br>b. Diaphragm or cervical cap with spermicide<br>c. Intrauterine device, or contraceptive implant<br>d. Hormonal contraception<br>e. Successful vasectomy in the male partner (considered successful if a woman reports that a male partner has [1] documentation of azoospermia by microscopy (< 1 year ago), or [2] a vasectomy more than 2 years ago with no resultant pregnancy despite sexual activity post-vasectomy)<br>f. Not be of reproductive potential, such as having undergone

hysterectomy, bilateral oophorectomy, or tubal ligation, postmenopausal (> 45 years of age with amenorrhea for at least 2 years, or any age with amenorrhea for at least 6 months and a serum follicle stimulating hormone [FSH] level > 40 IU/L); surgically sterile: no additional contraception required.

Women, who are not heterosexually active at screening, must agree to utilize an effective method of contraception if they become hetero-sexually active, as outlined above.

4. All sexually active males, regardless of reproductive potential, must be willing to consistently use an effective method of contraception (such as consistent male condoms with male and/or female partners) from the day of investigational product administration until at least 3 months following investigational product administration to avoid exposure of partners to investigational product in ejaculate, and to prevent conception with female partners.
5. All female participants must be willing to undergo urine pregnancy tests at time points indicated in the Schedule of Procedures and must test negative prior to investigational product administration.
6. A female participant must agree not to donate eggs (ova, oocytes) for the purpose of assisted reproduction until 3 months after investigational product administration. A man must agree not to donate sperm until 3 months after investigational product administration.
7. Willing to forgo donations of blood and/or any other tissues, including bone marrow, during the study and, for those HIV-uninfected participants who test HIV-positive due to investigational product administration, until the anti-HIV antibody titers become undetectable.

Specific inclusion criteria for HIV-uninfected participants (Groups 1 and 2):

8. At least 18 years of age on the day of screening and has not reached his or her 51st birthday on the day of signing the Informed Consent Document.
9. Willing to undergo HIV testing, risk reduction counselling and receive HIV test results.
10. Low risk for HIV infection and willing to maintain low-risk behavior for the duration of the trial.
11. Healthy male or female, as assessed by a medical history, physical exam, and laboratory tests.

Specific inclusion criteria for HIV-infected participants (Group 3):

12. At least 18 years of age on the day of screening and has not reached his or her 66th birthday on the day of signing the Informed Consent Document.
13. Confirmed HIV-1 infection (HIV Ab+ or HIV RNA+) by documentation in the medical records or in-clinic HIV testing;
14. CD4 ≥ 300 cells/μl.

	15. Not receiving cART, and (after appropriate counselling) willing to defer cART treatment for at least 56 days after administration of investigational product.<br>16. HIV-1 viral load between 1000–100,000 copies/ml, confirmed at screening.<br>17. Under care of an HIV healthcare provider
**EXCLUSION CRITERIA**	**Exclusion criteria for all participants:**<br>1. Any clinically significant acute or chronic medical condition, other than HIV infection, that is considered progressive or in the opinion of the investigator makes the participant unsuitable for participation in the study.<br>2. If female, pregnant, lactating or planning a pregnancy during the period of screening through completion of the study.<br>3. In the past 6 months a history of alcohol or substance use, including marijuana, judged by the Investigator to potentially interfere with participant study compliance.<br>4. Bleeding disorder that was diagnosed by a physician (e.g., factor deficiency, coagulopathy or platelet disorder that requires special precautions). Note: A participant who states that he or she has easy bruising or bleeding, but does not have a formal diagnosis and has intramuscular injections and blood draws without any adverse experience, is eligible.<br>5. History of a splenectomy.<br>6. Receipt of live attenuated vaccine within the previous 30 days or planned receipt within 30 days after administration of investigational product; or receipt of other vaccine within the previous 14 days or planned receipt within 14 days after infusion with investigational product (exception is live attenuated influenza vaccine within 14 days).<br>7. Receipt of blood transfusion or blood-derived products within the previous 3 months.<br>8. Participation in another clinical trial of an investigational product currently, within the previous 3 months or expected participation during this study.<br>9. Prior receipt of an investigational HIV vaccine candidate, monoclonal antibody or polyclonal immunoglobulin (note: receipt of placebo in a previous HIV vaccine or monoclonal antibody trial will not exclude a participant from participation if documentation is available and the Medical Monitor gives approval).<br>10. History of severe local or systemic reactogenicity to injections or IV infusion (e.g., anaphylaxis, respiratory difficulties, angioedema);<br>11. Psychiatric condition that compromises safety of the participant and precludes compliance with the protocol. Specifically excluded are persons with psychoses within the past 3 years, ongoing risk for suicide, or history of suicide attempt or gesture within the past 3 years.<br>12. If, in the opinion of the Principal Investigator, it is not in the best

interest of the participant to participate in the trial.

13. Seizure disorder: a participant who has had a seizure in the last 3 years is excluded. (Not excluded: a participant with a history of seizures who has neither required medications nor had a seizure for 3 years.)
14. Body mass index ≥ 35 or ≤ 18.0.
15. Infectious disease: chronic hepatitis B infection (HbsAg), current hepatitis C infection (HCV Ab positive and HCV RNA positive) or interferon-alfa treatment for chronic hepatitis C infection in the past year, chlamydia, gonorrhea, or active syphilis.
16. A history of malignancy within the past 5 years (prior to screening) or ongoing malignancy;
17. Active, serious infections (other than HIV-1 infection) requiring parenteral antibiotic, antiviral or antifungal therapy within 30 days prior to enrollment.

Specific exclusion criteria for HIV-uninfected participants (Groups 1 and 2):

18. Confirmed HIV-1 or HIV-2 infection.
19. Any clinically relevant abnormality on history or examination including history of immunodeficiency or autoimmune disease; use of systemic corticosteroids, immunosuppressive, anticancer, or other medications considered significant by the investigator within the previous 6 months.

    The following exceptions are permitted and will not exclude study participation: use of corticosteroid nasal spray for rhinitis, topical corticosteroids for an acute uncomplicated dermatitis; or a short course (duration of 10 days or less, or a single injection) of corticosteroid for a non-chronic condition (based on investigator clinical judgment) at least 6 weeks prior to enrollment in this study.
20. Any of the following abnormal laboratory parameters listed below:

Hematology

- Hemoglobin < 10.5 g/dL in females; hemoglobin <11.0 g/dL in males
- Absolute Neutrophil Count (ANC): ≤ 1000/mm3
- Absolute Lymphocyte Count (ALC): < 650/mm3
- Platelets: < 125,000 mm3 or ≥ 550,000/mm3

Coagulation

- aPTT: >1.25 x ULN
- INR: ≥1.1 x ULN

Chemistry

- Sodium ≤ 135 mEq/L or ≥ 146 mEq/L
- Potassium ≤ 3.4 mEq/L or ≥ 5.6 mEq/L
- Creatinine ≥ 1.1 x ULN
- AST ≥ 1.25 x ULN
- ALT ≥ 1.25 x ULN
- Total bilirubin ≥ 1.25 x ULN

- Alkaline phosphatase ≥ 1.25 x ULN
- Albumin ≤ 3.0 g/dL or ≤ 30 g/L
- Creatine kinase ≥ 3.0 x ULN
- C-reactive protein > 10 mg/L
- C3 complement < 82 mg/dL
- C4 complement < 14 mg/dL

Urinalysis

Any of the following abnormal findings if consistent with clinically significant disease:

- Protein = greater than trace on dipstick confirmed by microscopic urinalysis outside institutional range.
- Blood = greater than trace on dipstick confirmed by >3 RBCs/hpf on microscopic urinalysis (not due to menses).

Specific exclusion criteria for HIV-infected participants who are not on ART (Group 3):

21. Any clinically relevant abnormality on history or examination including history of immunodeficiency or autoimmune disease, other than HIV; use of systemic corticosteroids, immunosuppressive, anticancer, or other medications considered significant by the investigator within the previous 6 months.

The following exceptions are permitted and will not exclude study participation: use of corticosteroid nasal spray for rhinitis, topical corticosteroids for an acute uncomplicated dermatitis; or a short course (duration of 10 days or less, or a single injection) of corticosteroid for a non-chronic condition (based on investigator clinical judgment) at least 6 weeks prior to enrollment in this study.

22. Any of the following abnormal laboratory parameters listed below:

Hematology
- Hemoglobin < 10.0 g/dL
- Absolute Neutrophil Count (ANC): < 1000 cells/mm3
- Platelets: < 100,000 cells/mm3

Coagulation
- aPTT: > 1.25 x ULN
- INR: ≥ 1.1 x ULN

Chemistry
- Estimated Glomerular filtration rate (GFR) < 80 mL/min according to the Cockcroft Gault formula for creatinine clearance
  - Male: (140 – age in years) x (wt in kg) = CLcr (mL/min) / 72 x (serum creatinine in mg/dL)
  - Female: (140 – age in years) x (wt in kg) x 0.85 = CLcr (mL/min) / 72 x (serum creatinine in mg/dL)
- AST ≥ 2.5 x ULN
- ALT ≥ 2.5 x ULN
- Total bilirubin ≥ 1.6 x ULN
- Alkaline phosphatase ≥ 5 x ULN

	Urinalysis<br>Any of the following abnormal findings if consistent with clinically significant disease:<br>o Protein = greater than 1+ on dipstick confirmed by microscopic urinalysis outside institutional range.<br>o Blood = greater than 1+ on dipstick confirmed by > 10 RBCs/hpf on microscopic urinalysis (not due to menses).<br>o Leukocytes = greater than 1+ on dipstick confirmed by > 10 WBC/hpf on microscopic urinalysis.

TABLE OF CONTENTS

PROTOCOL SYNOPSIS **2**

TABLE OF CONTENTS **14**

ABBREVIATIONS **18**

CONTACT INFORMATION **19**

1.0 SIGNATURE PAGE **20**

2.0 INTRODUCTION AND BACKGROUND INFORMATION **21**

2.1 Study Rationale 21
2.2 Clinical experience with PGDM1400 and PGT121 22

3.0 STUDY OBJECTIVES **23**

3.1 Primary Objectives 23
3.2 Secondary Objectives 23
3.3 Exploratory Objectives 23

4.0 STUDY ENDPOINTS **23**

4.1 Primary Endpoints 23
4.2 Secondary Endpoints 24
4.3 Exploratory Endpoints 24

5.0 STUDY DESIGN **25**

5.1 Definition of Dose Limiting Toxicity- Groups 1 and 2 25
5.2 Definition of Maximum Tolerated Dose- Groups 1 and 2 25
5.3 Dose Escalation and Determination of Maximum Tolerated Dose – Groups 1 and 2 25
5.4 Determination of Antiviral Effect – Group 3 27
5.5 Duration of the Study 27
5.6 Study Population 27
5.7 Inclusion Criteria 28
5.8 Exclusion Criteria 29
5.9 Recruitment of Participants 32

6.0 STUDY VISITS **33**

6.1 Screening Period 33
6.2 IV infusion of Investigational Product Visit 33
6.3 Post-IV infusion of Investigational Product Visits 34
6.4 Additional Follow-up Visits 35
6.5 Unscheduled Visits 35
6.6 Final Study Visit or Early Termination Visit 35

7.0 STUDY PROCEDURES **36**

7.1 Informed Consent Process 36
7.2 Medical History and Physical Examination 36
7.3 HIV Testing and HIV-test Counselling (Groups 1 and 2) 37
7.4 HIV Risk Reduction Counselling 37
7.5 Family Planning Counselling 37
7.6 ART Counselling (Group 3) 37
7.7 Specimens 38

7.8 Reimbursement ... 38
7.9 Randomization and Blinding ... 38
7.10 Un-blinding Procedure for Individual Participants ... 39
7.11 Assessment of PGDM1400 mAb and/or PGT121 mAb related HIV sero-positivity ... 39

8.0 INVESTIGATIONAL PRODUCT ... 39

8.1 Description ... 39
8.2 Shipment and Storage ... 40
8.3 Preparation of investigational product ... 40
8.4 Administration of investigational product ... 40
8.5 Accountability and Disposal of investigational product ... 41

9.0 ASSESSMENTS ... 41

9.1 Safety Assessments ... 41
9.1.1 Local reactogenicity ... 41
9.1.2 Systemic reactogenicity ... 41
9.1.3 Vital Signs ... 42
9.1.4 Other Adverse Events ... 42
9.1.5 Concomitant Medications ... 42
9.1.6 Routine laboratory parameters ... 42
9.1.7 Specific screening tests: ... 43
9.2 Virologic Assessments ... 43
9.3 Exploratory Immunogenicity Assessments ... 44
9.3.1 Antibody Responses ... 44
9.3.2 Cellular Responses ... 44
9.3.3 PBMC, Serum and Plasma Storage ... 44
9.4 Other Assessments ... 44
9.4.1 HIV Antibody Testing (Groups 1 and 2) ... 44
9.4.2 Pharmacokinetics ... 44
9.4.3 HLA Typing ... 45
9.4.5 Pregnancy Test ... 45
9.4.6 HIV Risk Assessment (Group 1 and 2) ... 45
9.4.7 Social Impact Assessment ... 45

10.0 ADVERSE EVENTS ... 45

10.1 Definition ... 45
10.2 Assessment of Severity of Adverse Events ... 46
10.3 Relationship to Investigational Product ... 46
10.4 Serious Adverse Events ... 47
10.5 Reporting Potential Immune-Mediated Diseases ... 48
10.6 Clinical Management of Adverse Events ... 49
10.7 Clinical Management of Infusion-Related Reactions and Stopping Criteria ... 50
10.8 Pregnancy ... 51
10.9 Intercurrent HIV Infection (Group 1 and 2) ... 51

11.0 MANAGEMENT OF HIV ISSUES DURING AND FOLLOWING STUDY ... 51

11.1 HIV Testing – Groups 1 and 2 ... 51
11.2 Social Discrimination as a Result of investigational product-related antibodies ... 52
11.3 HIV infection – Group 1 and 2 ... 52
11.3.1 Counselling ... 52
11.3.2 Referral for Support/Care ... 52

12.0 WITHDRAWAL FROM STUDY ... 53

12.1 Deferral of IV infusion of investigational product 53
12.2 Withdrawal from the Study (Early Termination) 53

13.0 DATA HANDLING 54

13.1 Data Collection and Record Keeping at the Study Site 54
13.2 Data Entry at the Study Site 54
13.3 Data Analysis 54

14.0 STATISTICAL CONSIDERATIONS 54

14.1 Safety and Tolerability Analysis 54
14.2 Pharmacokinetic Analysis 55
14.3. Virologic Analysis for Group 3 57
14.5 Secondary and Exploratory Immunologic and Virologic Analyses 58

15.0 QUALITY CONTROL AND QUALITY ASSURANCE 58

16.0 DATA AND BIOLOGICAL MATERIAL 59

17.0 ADMINISTRATIVE STRUCTURE 59

17.1 Protocol Safety Review Team 59
17.2 Safety Monitoring Committee (SMC) 60
17.2.1 Content of Interim Safety Review 60
17.2.2 SMC Review of Group 1 and 2 data prior to starting Group 3 60
17.3 Criteria for Pausing the Study 60
17.4 Study Supervision 61
17.5 Study Monitoring 62
17.6 Investigator's Records 62

18.0 INDEMNITY 62

19.0 PUBLICATION 62

20.0 ETHICAL CONSIDERATIONS 63

APPENDIX A: SCHEDULE OF PROCEDURES – GROUP 1 (A, B, C) 64

APPENDIX B: SCHEDULE OF PROCEDURES – AND GROUP 2 (A, B, C) 66

APPENDIX C: SCHEDULE OF PROCEDURES – GROUP 3 (A, B) 68

APPENDIX D: LOW RISK CRITERIA 71

APPENDIX E REFERENCES 73

APPENDIX F CTCAE TABLE 76

APPENDIX G: DAIDS ADVERSE EVENT SEVERITY ASSESSMENT TABLE 87

Introduction 91
Major Clinical Conditions Cardiovascular 95
Cardiovascular 96
Dermatologic 97
Endocrine and Metabolic 98
Gastrointestinal 99
Gastrointestinal 100
Musculoskeletal 101
Neurologic 102
Neurologic 103
Pregnancy, Puerperium, and Perinatal 103

Psychiatric ... 104
Respiratory ... 105
Sensory ... 106
Systemic ... 107
Systemic ... 108
Urinary ... 108
Site Reactions to Injections and Infusions ... 109
Laboratory Values Chemistries ... 110

ABBREVIATIONS

Abbreviation	Term
**AE**	Adverse Event
**AIDS**	Acquired Immunodeficiency Syndrome
**ALT**	Alanine-Aminotransferase
**ART**	Antiretroviral Therapy
**AST**	Aspartate-Aminotransferase
**CFC**	Cytokine Flow Cytometry
**CMI**	Cell Mediated Immunity
**CRF**	Case Report Form
**CTL**	Cytotoxic T Lymphocyte
**DCC**	Data Coordinating Center
**DLT**	Dose Limiting Toxicity
**DNA**	Deoxyribonucleic Acid
**ELISA**	Enzyme Linked Immunosorbent Assay
**GCP**	Good Clinical Practice
**GFR**	Glomerular Filtration Rate
**HIV**	Human Immunodeficiency Virus
**HLA**	Human Leukocyte Antigen
**HSV**	Herpes Simplex Virus
**IAVI**	International AIDS Vaccine Initiative
**ICH**	International Council for Harmonization of Technical Requirements for Pharmaceuticals for Human Use
**IP**	Investigational Product
**IND**	Investigational New Drug Application
**IV**	Intravenous
**Kg**	Kilogram
**mAb**	Monoclonal Antibody
**mg**	Milligram
**MTD**	Maximum Tolerated Dose
**NHP**	Non Human Primate
**PCR**	Polymerase Chain Reaction
**PBMC**	Peripheral Blood Mononuclear Cells
**PK**	Pharmacokinetic
**RPR**	Rapid Plasma Reagin
**SAE**	Serious Adverse Event
**SIV**	Simian Immunodeficiency Virus
**SOP**	Standard Operating Procedure
**SOM**	Study Operations Manual
**SMC**	Safety Monitoring Committee
**STD**	Sexually Transmitted Disease
**TPHA**	Treponema Pallidum Hemagglutination

CONTACT INFORMATION

Detailed contact information provided in the Study Operation Manual (SOM)

**Sponsor Contact:**	
Frances Priddy MD MPH<br>Chief Medical Officer and Executive Director<br>International AIDS Vaccine Initiative<br>125 Broad Street, 9th Floor<br>New York, New York 10004	Phone: +1-212-328-7461<br>Mobile: +1-646-287-8943<br>Fax: +1-608-203-5501<br>E-mail: fpriddy@iavi.org
**Clinical Research Center Contacts:**	
Boris D. Juelg MD PhD<br>Center for Virology and Vaccine Research<br>Clinical Trials Unit<br>Beth Israel Deaconess Medical Center<br>E / CLS – 10th Floor, Room 1046<br>330 Brookline Avenue<br>Boston, Massachusetts 02215	Phone: +1-857-268-7088<br>Mobile: +1-617-401-6725<br>Fax: +1-617-735-4566<br>E-mail: bjulg@bidmc.harvard.edu

.

1.0 SIGNATURE PAGE

The signatures below constitute the approval of this protocol and the appendices and provide the necessary assurances that this study will be conducted in compliance with the protocol, Good Clinical Practices (GCP) and the applicable regulatory requirement(s).

Sponsor:

Signed: See electronic signature manifest Date:

Frances Priddy MD MPH
Chief Medical Officer and Executive Director, Medical Affairs, IAVI

Principal Investigator:

Signed: Date:

Name (please print):

Name of institution (please print):

2.0 INTRODUCTION AND BACKGROUND INFORMATION

In 2015, 36.7 million people globally were living with HIV, 2.1 million people became newly infected with HIV, and 1.1 million people died from AIDS-related illnesses (UNAIDS 2016). More than 78 million people have become infected with HIV and 39 million people have died since the beginning of the AIDS epidemic (UNAIDS 2016). One reason that such high rates of AIDS-related deaths continue to occur globally – despite the advent of drugs that are highly effective at suppressing HIV replication – is that by June 2016, 18.2 million people (two in four people living with HIV) were accessing antiretroviral therapy (ART) (UNAIDS 2016). Another reason for continued AIDS-related mortality is that ART does not cure HIV infection and must be maintained for a lifetime (Barouch and Deeks 2014). Even in the United States (US), only 30% of the 1.2 million people living with HIV have suppressed HIV to undetectable levels, likely because 1) not everyone is aware of their HIV-positive status, 2) not everyone is accessing ART, and 3) of the challenge of maintaining adequate continuity of ART over many years (CDC 2014).

2.1 Study Rationale

This is a Phase 1 study to evaluate the safety, tolerability, pharmacokinetics and anti-viral efficacy of the PGDM1400 and PGT121 mAbs for HIV prevention and therapy. PGDM1400 mAb and PGT121 mAb are recombinant human IgG1 monoclonal antibodies that target a V1V2 (PGDM1400) and a V3 glycan-dependent (PGT121) epitope region of the HIV envelope protein (Jardine, Julien et al. 2013, Sok, Doores et al. 2014). PGDM1400 and PGT121 mAbs were chosen for this study because of their potency, their ability to neutralize a wide array of cross-clade HIV viruses in a complementary pattern, and their proven antiviral activity in animals studies e.g., their capacity to robustly prevent and treat simian-human immunodeficiency virus (SHIV) in rhesus monkeys.

Some HIV-infected individuals develop HIV specific antibodies capable of neutralizing a broad range of HIV virus strains (Simek, Rida et al. 2009, Walker, Phogat et al. 2009). By selecting and cloning B-cells from such HIV-infected individuals, monoclonal antibodies have been identified which have potent and broadly neutralizing activity against a wide range of HIV virus strains (Walker LM 2010). New developments in high throughput single-cell BCR-amplification and novel soluble Env baits have led to the isolation of monoclonal antibodies with extraordinary potency and breadth (Walker, Huber et al. 2011).

These HIV-specific antibodies target the HIV envelope (Env) and can prevent SHIV infection in rhesus monkeys and have been shown to reduce HIV RNA levels in humans temporarily (Hessell, Poignard et al. 2009, Hessell, Rakasz et al. 2009, Moldt, Rakasz et al. 2012, Barouch, Whitney et al. 2013, Caskey, Klein et al. 2015, Caskey, Schoofs et al. 2017). These broadly neutralizing antibodies (bnAbs) may be effective for prevention of HIV infection when administered passively (Haynes and McElrath 2013, Burton and Mascola 2015). It is likely that bnAbs targeting different HIV epitopes will need to be used in combination to prevent development of resistance and to adequately cover all global HIV strains.

PGDM1400 can neutralize a wide array of HIV-1 viruses *in vitro* and can treat and prevent simian-human immunodeficiency virus (SHIV) in the NHP model (Sok, van Gils et al. 2014) and (Julg et al, unpublished data).
PGDM1400 is a novel monoclonal antibody that targets the V1V2 loop on the HIV Env spike, distinct from VRC01 and 3BNC117 which both target the CD4 binding site, and PGT121 and 10-1074 which target the V3 loop.

PGDM1400 mAb and PGT121 mAb were selected for development because of the following critical attributes:

1. PGDM1400 neutralizes 83% of global viral isolates at a median IC50 of 0.003 µg/mL (Sok et al 2014) and is therefore 10 to 100-fold more potent than the previous best-in-class CD4bs antibodies VRC01, VRC07, and 3BNC117 (Scheid, Mouquet et al. 2011, Walker, Huber et al. 2011, Sok, van Gils et al. 2014)
2. PGDM1400 protects against SHIV acquisition in monkeys at substantially lower dose levels compared to VRC01 and 3BNC117 (unpublished data)
3. PGT121 mAb has superior therapeutic efficacy in SHIV-infected monkeys compared to VRC01, 3BNC117, and 10-1074 (Barouch, Whitney et al. 2013) (and unpublished data). Specifically, SHIV-infected monkeys with low baseline viral load achieved and maintained undetectable viral loads for up to 100 days after administration of PGT121 (Barouch, Whitney et al. 2013) (and unpublished data).
4. PGT121 mAb may have a higher bar to escape in vivo as compared to other V3 glycan and CD4bs antibodies as a result of making multiple glycan contacts (Sok, van Gils et al. 2014).
5. PGDM1400 and PGT121 are complementary in the coverage of global viral isolates and when combined neutralize 98-99% of global HIV-1 viruses tested and at unparalleled potency with a median IC50 of 0.007 µg/ml (Sok, van Gils et al. 2014).

The potency and breadth of PGDM1400 mAb and PGT121 mAb, both alone and in combination with other bNAbs, raise the possibility that combinations may be effective for HIV prophylaxis at low doses and against global viruses. An antibody that is effective at low doses may eventually be given subcutaneously, which would reduce the cost. It is these features that make PGDM1400 mAb and PGT121 mAb particularly well-suited for preventing and/or treating HIV in the developing world, where it is critical that a public health intervention be low cost, easy to deliver, and effective in diverse settings.

2.2 Clinical experience with PGDM1400 and PGT121

There is no previous clinical experience with PGDM1400 mAb, and limited experience with PGT121 mAb. The safety, pharmacokinetics and antiviral effects of PGT121 mAb alone are being evaluated in the ongoing T001 study (IND 126807, NCT02960581). Several other HIV monoclonal antibodies are currently in clinical development as passive HIV immunoprophylaxis, or as potential therapeutics (10-1074, 3BNC117, VRC01, VRC01LS). Published data from phase 1 studies shows acceptable preliminary safety and tolerability profiles for these products, and similar anti-viral effects and pharmacokinetics (Caskey, Klein et al. 2015, Ledgerwood, Coates et al. 2015, Lynch, Boritz et al. 2015, Bar, Sneller et al. 2016, Scheid, Horwitz et al. 2016, Schoofs, Klein et al. 2016, Caskey, Schoofs et al. 2017). A comprehensive summary of phase 1 studies of HIV monoclonal antibodies can be found in the Investigator's Brochure.

3.0 STUDY OBJECTIVES

3.1 Primary Objectives

- To evaluate the safety and tolerability of IV infusion of PGDM1400 mAb alone, and a combination of PGDM1400 mAb and PGT121 mAb, in HIV-uninfected adults and HIV-infected adults
- To evaluate the pharmacokinetic (PK) profile of IV infusion of PGDM1400 mAb alone, and a combination of PGDM1400 mAb and PGT121 mAb, in HIV-uninfected adults and HIV-infected adults
- To evaluate the antiviral activity of IV infusion of PGDM1400 mAb alone, and a combination of PGDM1400 and PGT121 mAb, in HIV-infected adults not on ART

3.2 Secondary Objectives

- To determine if PGDM1400 mAb alone, and in combination with PGT121 mAb, induces anti-PGDM1400 and/or anti-PGT121 antibodies
- To determine the effect of PGDM1400 mAb alone, and in combination with PGT121 mAb, on CD4+ T cell counts in HIV-infected adults
- To determine the effect of PGDM1400 mAb alone, and in combination with PGT121 mAb, on viral escape in viremic HIV-infected adults not on ART

3.3 Exploratory Objectives

- To determine if PGDM1400 ± PGT121 mAb has any impact on the host immune responses (i.e., HIV-specific cellular and humoral immune response).
- To determine the effect of PGDM1400 ± PGT121 mAb on the size of the latent HIV reservoir in HIV-infected adults.
- To determine PGDM1400 mAb ± PGT121 mAb levels in mucosal secretions in HIV-uninfected and HIV-infected adults.
- To measure in vitro neutralization of HIV isolates with participant’s serum post PGDM1400 ± PGT121 mAb IV infusion.

4.0 STUDY ENDPOINTS

4.1 Primary Endpoints

Safety and Tolerability

1. Proportion of participants with moderate or greater reactogenicity (e.g., solicited adverse events) for 3 days following IV infusion of PGDM1400 mAb alone, and a combination of PGDM1400 mAb and PGT121 mAb.
2. Proportion of participants with adverse events (AEs), including safety laboratory (biochemical, hematological) parameters, during the first 56 days following IV infusion of PGDM1400 mAb alone and a combination of PGDM1400 mAb and PGT121 mAb, that are moderate or greater, and/or related to PGDM1400 mAb or PGT121 mAb.
3. Proportion of participants with serious adverse events (SAEs) throughout the study period following IV infusion of PGDM1400 mAb alone and a combination of PGDM1400 mAb and PGT121 mAb, that are related to PGDM1400 mAb or PGT121 mAb.

Pharmacokinetics

Pharmacokinetics following IV infusion of PGDM1400 mAb alone or a combination of PGDM1400 mAb and PGT121 mAb in HIV-uninfected and HIV-infected adults:

1. Elimination half-life (t1/2)
2. Clearance (CL/F)
3. Volume of distribution (Vz/F)
4. Area under the concentration decay curve (AUC)
5. Impact of viral load and/or ART on PGDM1400 mAb and PGT121 mAb disposition (elimination half-life (t1/2), clearance (CL/F), volume of distribution (Vz/F), total exposure)

Antiviral activity

Antiviral activity following IV infusion of PGDM1400 mAb alone or in combination with PGT121 mAb in viremic HIV-infected adults not on ART:

1. Change in plasma HIV-1 RNA levels from baseline (mean of pre-entry and entry values)

4.2 Secondary Endpoints

Anti-PGDM1400 and anti-PGT121 antibodies

1. Serum anti-PGDM1400 antibody titers
2. Serum anti-PGT121 antibody titers

CD4+ T cell count

We will calculate the following endpoint to determine if IV infusion of PGDM1400 mAb alone or in combination with PGT121 mAb has any impact on CD4+ T cell counts in HIV-infected adults:

1. Change in CD4+ T cell count and frequency compared to baseline as measured by single platform flow cytometry

HIV genotyping/phenotyping of circulating virus for evaluation of PGDM1400 mAb and/or PGT121 mAb -induced escape mutations

We will compare plasma virus genotype and phenotypic activity before and after IV infusion of PGDM1400 mAb alone or in combination with PGT121 mAb to determine if PGDM1400 mAb and/or PGT121 mAb induce viral escape mutations have developed in viremic HIV-infected adults not on ART.

1. Genotypic analysis: Development of sequence variations in epitopes known to result in reduced PGDM1400 mAb and/or PGT121 mAb neutralization susceptibility or known to cause resistance to antiretroviral drugs.
2. Phenotypic analysis: Changes in viral susceptibility to PGDM1400 mAb and/or PGT121 mAb neutralization.

4.3 Exploratory Endpoints

Additional assessments may include but are not limited to the following: HIV-specific IgG/IgA binding responses by ELISA, HIV-specific cellular immune responses by ELISPOT, HIV-specific antibody function by ADCC, ADCP, and ADCVI assays, PGDM1400 mAb and/or PGT121 mAb levels in mucosal secretions, changes in total HIV-1 DNA and 2-long terminal repeat (LTR) circular HIV-1 DNA in resting or total CD4 T cells and in vitro neutralization of HIV isolates with participant’s serum post IV infusion of PGDM1400 mAb ± PGT121 mAb.

5.0 STUDY DESIGN

The study is a double-blind, randomized, placebo-controlled study for Groups 1 and 2, and open label for Group 3 who will not receive placebo. A single intravenous (IV) infusion of PGDM1400 mAb alone at 3, 10 or 30 mg/kg, or a combination of PGDM1400 mAb and PGT121 mAb at 3, 10 or 30 mg/kg each, or placebo will be administered to participants. See study Table 5.0.1 for details.

Table 5.0.1 Study Design Table

	Group	Participants	Sub-Group	Regimen	N	Dose (mg/kg)
Part 1 – MTD	1	HIV-uninfected participants	1A	PGDM1400/Placebo	3/1 (6/2 if DLT)	3 IV
			1B	PGDM1400/Placebo	3/1 (6/2 if DLT)	10 IV
			1C	PGDM1400/Placebo	3/1 (6/2 if DLT)	30 IV
				Total Group 1	9/3 = 12 (max 18/6 = 24 if DLT)	
	2	HIV-uninfected participants	2A	PGDM1400 + PGT121/Placebo	3/1 (6/2 if DLT)	3 + 3 IV
			2B	PGDM1400 + PGT121/Placebo	3/1 (6/2 if DLT)	10 + 10 IV
			2C	PGDM1400 + PGT121/Placebo	3/1 (6/2 if DLT)	30 + 30 IV
				Total Group 2	9/3 = 12 (max 18/6 = 24 if DLT)	
				Total Groups 1 and 2	18/6 = 24 (max 36/12 = 48 if DLT)	
Safety Monitoring Committee						
Part 2 – antiviral effect	3	HIV-infected off ART (VL 1x10³ – 1x10⁵ copies/ml)	3A	PGDM1400	6 (max 18)	MTD IV
			3B	PGDM1400 + PGT121	6 (max 18)	MTD IV
				Total Group 3	12 (max 36)	
				Total entire study	36 (max 84)	

DLT, dose limiting toxicity; MTD, maximum tolerated dose

5.1 Definition of Dose Limiting Toxicity- Groups 1 and 2

Dose limiting toxicity (DLT) will be defined as 1) any Grade 3 or greater adverse event considered possibly, probably or definitely related to investigational product or 2) any Grade 3 or greater reactogenicity or 3) any SAE considered possibly, probably or definitely related to investigational product.

5.2 Definition of Maximum Tolerated Dose- Groups 1 and 2

If 2 or more DLTs occur in a dose Subgroup (e.g., 1B) that are the same, similar, or in the same System Organ Class, infusion will be halted and the next lower dose level will be declared the maximum tolerated dose (MTD) within this Group. If no DLT occurs in the final dose Subgroup (1C and 2C), MTD will be the highest dose given (30mg/kg) after 14 days of follow-up.

5.3 Dose Escalation and Determination of Maximum Tolerated Dose – Groups 1 and 2

Part 1 of this study is a dose-escalation trial in HIV-uninfected adults (Groups 1 and 2) to establish the MTD of PGDM1400 mAb alone and a combination of PGDM1400 mAb and

PGT121 mAb. There will be sentinel participants in each dose Subgroup, and safety data will be reviewed to determine if dose escalation can proceed.

Sentinel participants in Groups 1 and 2

For each dose Subgroup in Groups 1 and 2, the 1st 2 participants will be sentinel participants for whom investigational product infusion will be separated by at least 24 hours contingent on review of safety information prior to infusion of the next sentinel participant. If no reactogenicity or adverse events that meet the DLT criteria occur within 24 hours after IV infusion of the 1st participant, the 2nd participant may be infused with investigational product. If no events meeting the DLT criteria occur within 24 hours after the 2nd participant is infused, then the remaining participants in that dose Subgroup will be infused. If events meeting the DLT criteria do occur for the first 2 participants in a dose Subgroup, the data will be reviewed by the Safety Monitoring Committee (SMC) to determine whether further infusions may proceed.

Staggered dose escalation design whereby PGDM1400 alone and PGT121 alone safety data are reviewed before combined infusion of PGDM1400 and PGT121 at the same dose

The combination Groups, starting with PGDM1400 3 mg/kg + PGT121 3 mg/kg can only start when 1) the PSRT has reviewed the safety data through day 14 post investigational product infusion from PGDM1400 alone at 3 mg/kg and has approved dose escalation to 10 mg/kg AND 2) the MTD of PGT121 as determined in the T001 study is 3 mg/kg or higher. Dosing levels of PGDM1400 mAb and PGT121 in the combination Subgroups can never exceed the MTDs for PGDM1400 and PGT121 as determined in the PGDM1400 alone Group 1 in this study, and the PGT121 alone Group in the T001 study.

PSRT review for dose escalation

The Protocol Safety Review Team (PSRT) will review safety data through day 14 post-investigational product infusion for all participants in each dose Subgroup for PGDM1400 mAb alone (Group 1) prior to allowing enrolment of participants into the next higher dose Subgroup in Group 1 or the same dose level PGDM1400 mAb and PGT121 mAb combination dose Subgroup (Group 2).

- If no DLT occurs in the initial 4 participants of a dose Subgroup (e.g., 1A), the study can proceed with enrolment of the next dose Subgroup for PGDM1400 mAb alone (e.g., 1B), and the same dose level PGDM1400 mAb and PGT121 mAb combination dose Subgroup (e.g., 2A).
- If 1 DLT occurs in the initial 4 participants of a dose Subgroup (e.g., 1A), 4 additional participants will be enrolled in the same dose Subgroup.
    - If no additional DLTs occur within 14 days of infusion in the 8 total participants, the study can proceed with enrolment of the next dose Subgroup for PGDM1400 mAb alone (e.g., 1B), and the same dose level PGDM1400 mAb and PGT121 mAb combination dose Subgroup (e.g., 2A).
    - If 2 or more DLTs accumulate in a dose Subgroup (e.g., 1B) that are the same, similar, or in the same System Organ Class, infusion will be halted and the next lower dose level will be declared the maximum tolerated dose (MTD) within this Group.
    - If no DLT occurs in the final dose Subgroup (e.g.,1C), the MTD for PGDM1400 alone will be the highest dose given (30mg/kg) after 14 days of follow-up.

- Dosing levels of PGDM1400 mAb and PGT121 in the combination Subgroups can never exceed the MTDs for PGDM1400 and PGT121 as determined in the PGDM1400 alone Group 1 in this study, and the PGT121 alone Group in the T001 study.

SMC review to determine MTD of PGDM1400 alone
Following IV infusion of of investigational product in the last participant in Group 1 , an independent Safety Monitoring Committee (SMC) will review safety data through day 14 post-investigational product infusion for all participants to confirm MTD of PGDM1400 alone, and determine whether, and at what dose, Group 3A can initiate enrolment.

SMC review to determine MTD of the combination of PGDM1400 and PGT121
Following IV infusion of investigational product in the last participant in Group 2, the SMC will review safety data through day 14 post-investigational product infusion for all participants to confirm MTD of the combination of PGDM1400 and PGT121, and determine whether Group 3B can initiate enrollment.

5.4 Determination of Antiviral Effect – Group 3

Part 2 of this study will establish the antiviral effect of PGDM1400 mAb alone or the combination of PGDM1400 mAb plus PGT121 mAb in HIV infected adults not on ART (Group 3).

MTD determines dose
Group 3 will start with the MTD of PGDM1400 as determined by the SMC; e.g., if the MTD for PGDM1400 is 30mg/kg then Subgroup 3A will receive 30mg/kg and Subgroup 3B will receive PGDM1400 mAb + PGT121 mAb at a dose that combines the MTD for PGDM1400 mAb and the MTD for PGT121 mAb (as determined in the ongoing T001 study IND 126807, NCT02960581).

5.5 Duration of the Study

Up to 32 weeks per participant, screening up to 42 (HIV-infected) or up to 56 (HIV-uninfected) days before single IV infusion of investigational product on day 0, and 24 weeks of follow up.

It will take approximately 11 months to enroll the entire study. The exact duration depends on the recruitment rate and how many participants will be required per dose Subgroup as specified in sections 5.3 and 5.4.

5.6 Study Population

The study population consists of HIV-uninfected male or female adults (Group 1 and 2) and HIV-infected males and female adults (Group 3) who meet the detailed inclusion and exclusion criteria listed below, and who in the opinion of the investigator or designee, understand the study and provide written informed consent.

Approximately 36-84 participants (72 active product recipients, 12 placebo recipients) who meet all eligibility criteria will be included in the study. An over-enrollment of up to 5% (up to 5 participants total) will be permitted in the study to facilitate rapid enrollment.

5.7 Inclusion Criteria

Inclusion criteria for all participants:

1. Willing to comply with the requirements of the protocol and available for follow-up for the planned duration of the study.

2. In the opinion of the Principal Investigator or designee and based on Assessment of Informed Consent Understanding results, has understood the information provided and potential impact and/or risks linked to IV infusion and participation in the trial; written informed consent will be obtained from the participant before any study-related procedures are performed.

3. All heterosexually active female participants must commit to use an effective method of contraception for 3 months following investigational product administration, including:

   a. Condoms (male or female) with or without spermicide

   b. Diaphragm or cervical cap with spermicide

   c. Intrauterine device, or contraceptive implant

   d. Hormonal contraception

   e. Successful vasectomy in the male partner (considered successful if a woman reports that a male partner has [1] documentation of azoospermia by microscopy (< 1 year ago), or [2] a vasectomy more than 2 years ago with no resultant pregnancy despite sexual activity post-vasectomy)

   f. Not be of reproductive potential, such as having undergone hysterectomy, bilateral oophorectomy, or tubal ligation, postmenopausal (> 45 years of age with amenorrhea for at least 2 years, or any age with amenorrhea for at least 6 months and a serum follicle stimulating hormone [FSH] level > 40 IU/L); surgically sterile: no additional contraception required.

   g. Women, who are not heterosexually active at screening, must agree to utilize an effective method of contraception if they become hetero-sexually active, as outlined above.

4. All sexually active males, regardless of reproductive potential, must be willing to consistently use an effective method of contraception (such as consistent male condoms with male and/or female partners) from the day of investigational product administration until at least 3 months following investigational product administration to avoid exposure of partners to investigational product in ejaculate, and to prevent conception with female partners.

5. All female participants must be willing to undergo urine pregnancy tests at time points indicated in the Schedule of Procedures and must test negative prior to investigational product administration.

6. A female participant must agree not to donate eggs (ova, oocytes) for the purpose of assisted reproduction until 3 months after investigational product administration. A man must agree not to donate sperm until 3 months after investigational product administration.

7. Willing to forgo donations of blood and/or any other tissues, including bone marrow, during the study and, for those HIV-uninfected participants who test HIV-positive due to investigational product administration, until the anti-HIV antibody titers become undetectable.

Specific inclusion criteria for HIV-uninfected participants (Groups 1 and 2):

8. At least 18 years of age on the day of screening and has not reached his or her 51st birthday on the day of signing the Informed Consent Document.

9. Willing to undergo HIV testing, risk reduction counselling and receive HIV test results.

10. Low risk for HIV infection and willing to maintain low-risk behavior for the duration of the trial.

11. Healthy male or female, as assessed by a medical history, physical exam, and laboratory tests.

Specific inclusion criteria for HIV-infected participants (Groups 3):

12. At least 18 years of age on the day of screening and has not reached his or her 66th birthday on the day of signing the Informed Consent Document.

13. Confirmed HIV-1 infection (HIV Ab+ or HIV RNA+) by documentation in the medical records or in-clinic HIV testing;

14. CD4 ≥ 300 cells/μl.

15. Not receiving cART, and (after appropriate counselling) willing to defer cART treatment for at least 56 days after administration of investigational product.

16. HIV-1 viral load between 1000–100,000 copies/ml, confirmed at screening.

17. Under care of an HIV healthcare provider

5.8 Exclusion Criteria

Exclusion criteria for all participants:

1. Any clinically significant acute or chronic medical condition, other than HIV infection, that is considered progressive or in the opinion of the investigator makes the participant unsuitable for participation in the study.

2. If female, pregnant, lactating or planning a pregnancy during the period of screening through completion of the study.

3. In the past 6 months a history of alcohol or substance use, including marijuana, judged by the Investigator to potentially interfere with participant study compliance.

4. Bleeding disorder that was diagnosed by a physician (e.g., factor deficiency, coagulopathy or platelet disorder that requires special precautions). Note: A participant who states that he or she has easy bruising or bleeding, but does not have a formal diagnosis and has intramuscular injections and blood draws without any adverse experience, is eligible.

5. History of a splenectomy.

6. Receipt of live attenuated vaccine within the previous 30 days or planned receipt within 30 days after administration of investigational product; or receipt of other vaccine within the previous 14 days or planned receipt within 14 days after infusion with investigational product (exception is live attenuated influenza vaccine within 14 days).

7. Receipt of blood transfusion or blood-derived products within the previous 3 months.

8. Participation in another clinical trial of an investigational product currently, within the previous 3 months or expected participation during this study.

9. Prior receipt of an investigational HIV vaccine candidate, monoclonal antibody or polyclonal immunoglobulin (note: receipt of placebo in a previous HIV vaccine or monoclonal antibody trial will not exclude a participant from participation if documentation is available and the Medical Monitor gives approval).

10. History of severe local or systemic reactogenicity to injections or IV infusion (e.g., anaphylaxis, respiratory difficulties, angioedema);

11. Psychiatric condition that compromises safety of the participant and precludes compliance with the protocol. Specifically excluded are persons with psychoses within the past 3 years, ongoing risk for suicide, or history of suicide attempt or gesture within the past 3 years.

12. If, in the opinion of the Principal Investigator, it is not in the best interest of the participant to participate in the trial.

13. Seizure disorder: a participant who has had a seizure in the last 3 years is excluded. (Not excluded: a participant with a history of seizures who has neither required medications nor had a seizure for 3 years.)

14. Body mass index ≥ 35 or ≤ 18.0.

15. Infectious disease: chronic hepatitis B infection (HbsAg), current hepatitis C infection (HCV Ab positive and HCV RNA positive) or interferon-alfa treatment for chronic hepatitis C infection in the past year, chlamydia, gonorrhea, or active syphilis.

16. A history of malignancy within the past 5 years (prior to screening) or ongoing malignancy;

17. Active, serious infections (other than HIV-1 infection) requiring parenteral antibiotic, antiviral or antifungal therapy within 30 days prior to enrollment.

Specific exclusion criteria for HIV-uninfected participants (Group 1 and 2):

18. Confirmed HIV-1 or HIV-2 infection.

19. Any clinically relevant abnormality on history or examination including history of immunodeficiency or autoimmune disease; use of systemic corticosteroids, immunosuppressive, anticancer, or other medications considered significant by the investigator within the previous 6 months.

    The following exceptions are permitted and will not exclude study participation: use of corticosteroid nasal spray for rhinitis, topical corticosteroids for an acute uncomplicated dermatitis; or a short course (duration of 10 days or less, or a single injection) of corticosteroid for a non-chronic condition (based on investigator clinical judgment) at least 6 weeks prior to enrollment in this study.

20. Any of the following abnormal laboratory parameters listed below:

    Hematology
    Hemoglobin < 10.5 g/dL in females; hemoglobin <11.0 g/dL in males
    Absolute Neutrophil Count (ANC): ≤ 1000/mm3
    Absolute Lymphocyte Count (ALC): < 650/mm3
    Platelets: < 125,000 mm3 or ≥ 550,000/mm3
    Coagulation
    aPTT: >1.25 x ULN
    INR: ≥1.1 x ULN
    Chemistry
    - Sodium ≤ 135 mEq/L or ≥ 146 mEq/L
    - Potassium ≤ 3.4 mEq/L or ≥ 5.6 mEq/L
    - Creatinine ≥ 1.1 x ULN
    - AST ≥ 1.25 x ULN
    - ALT ≥ 1.25 x ULN
    - Total bilirubin ≥ 1.25 x ULN
    - Alkaline phosphatase ≥ 1.25 x ULN
    - Albumin ≤ 3.0 g/dL or ≤ 30 g/L
    - Creatine kinase ≥ 3.0 x ULN
    - C-reactive protein > 10 mg/L
    - C3 complement < 82 mg/dL
    - C4 complement < 14 mg/dL

    Urinalysis
    Any of the following abnormal findings if consistent with clinically significant disease:
    - Protein = greater than trace on dipstick confirmed by microscopic urinalysis outside institutional range.
    - Blood = greater than trace on dipstick confirmed by >3 RBCs/hpf on microscopic urinalysis (not due to menses).

Specific exclusion criteria for HIV-infected participants who are not on ART (Group 3):

21. Any clinically relevant abnormality on history or examination including history of immunodeficiency or autoimmune disease, other than HIV; use of systemic corticosteroids, immunosuppressive, anticancer, or other medications considered significant by the investigator within the previous 6 months.

    The following exceptions are permitted and will not exclude study participation: use of corticosteroid nasal spray for rhinitis, topical corticosteroids for an acute uncomplicated dermatitis; or a short course (duration of 10 days or less, or a single injection) of corticosteroid for a non-chronic condition (based on investigator clinical judgment) at least 6 weeks prior to enrollment in this study.

22. Any of the following abnormal laboratory parameters listed below:

    Hematology
    - Hemoglobin < 10.0 g/dL
    - Absolute Neutrophil Count (ANC): < 1000 cells/mm3
    - Platelets: < 100,000 cells/mm3

    Coagulation
    - aPTT: > 1.25 x ULN
    - INR: ≥ 1.1 x ULN

    Chemistry
    - Estimated Glomerular filtration rate (GFR) < 80 mL/min according to the Cockcroft Gault formula for creatinine clearance
        - Male: (140 – age in years) x (wt in kg) = CLcr (mL/min) / 72 x (serum creatinine in mg/dL)
        - Female: (140 – age in years) x (wt in kg) x 0.85 = CLcr (mL/min) / 72 x (serum creatinine in mg/dL)
    - AST ≥ 2.5 x ULN
    - ALT ≥ 2.5 x ULN
    - Total bilirubin ≥ 1.6 x ULN
    - Alkaline phosphatase ≥ 5 x ULN

    Urinalysis

    Any of the following abnormal findings if consistent with clinically significant disease:
    - Protein = greater than 1+ on dipstick confirmed by microscopic urinalysis outside institutional range.
    - Blood = greater than 1+ on dipstick confirmed by > 10 RBCs/hpf on microscopic urinalysis (not due to menses).
    - Leukocytes = greater than 1+ on dipstick confirmed by > 10 WBC/hpf on microscopic urinalysis.

5.9 Recruitment of Participants

Adult male and female participants may be recruited through in-clinic referrals, information presented to community organizations, hospitals, colleges, other institutions and/or advertisements to the general public or from existing cohorts. The information distributed will contain contact details of the trial site.

6.0 STUDY VISITS

6.1 Screening Period

During Screening, study staff will perform the following procedures:

- Provide and/or review the Informed Consent Document and answer any questions about the study prior to obtaining written informed consent.
- Complete Assessment of Informed Consent Understanding (AOU). Please refer to the Study Operations Manual (SOM)

If the participant agrees to participate, passes the AOU and provides written informed consent, study staff will:

- Conduct HIV test counselling, HIV testing, and HIV risk reduction counselling, as applicable
- Conduct family planning counselling, refer for pregnancy prevention counselling if necessary
- Administer HIV risk assessment (Group 1 and 2)
- Conduct ART counselling (Group 3)
- Perform a comprehensive medical history
- Collect concomitant medication information
- Perform a general physical examination (Refer to Section 7.2)
- Collect specimens for all tests as indicated in the Schedule of Procedures in Appendices A, B and C (for details see Analytical Plan (AP)).

When available, the screening laboratory tests will be reviewed by the trial physician. Screening laboratory test(s) may be repeated once at the discretion of the principal investigator or designee to investigate any isolated abnormalities.

If the screening visit occurs outside the allowable screening window, all screening procedures must be repeated except the comprehensive medical history may be replaced by an interim medical history and the Participant Information Sheet of the Informed Consent Document should be reviewed.

If a participant has signed the Consent Form but does not meet the eligibility criteria, the records must be kept at the site.

6.2 IV infusion of Investigational Product Visit

Prior to the infusion of investigational product, study staff will:

- Answer any questions the participant may have about the study
- Review the Informed Consent Document with the participant
- Review screening safety laboratory data
- Conduct HIV test counselling, and HIV risk reduction counselling , as applicable
- Conduct ART counselling (Group 3)
- Conduct family planning counselling as per site specific procedures and ensure compliance with respective pregnancy prevention method, and discuss male condom use with all male participants

- Review interim medical history
- Collect concomitant medication information
- Weigh participant and record vital signs
- Perform a symptom-directed physical examination (Refer to Section 7.2)
- Assess at baseline local and systemic signs and symptoms (this includes an examination of IV infusion site)
- Collect specimens for all tests as indicated in the Schedule of Procedures see Appendices A, B and C (for details see AP).
- Obtain pregnancy test results prior to infusion of investigational product.
- Assign an allocation number to the participant according to the instructions specified in the Study Operations Manual.

If a participant has an abnormal laboratory value that is known, at the time of infusion, follow the specified guidelines (Section 12.0).

At the time of infusion of investigational product and after IV infusion of investigational product, study staff will:

- Administer the investigational product as specified in Section 8.4, Administration of Investigational Product and according to the instructions specified in the SOM.
- Observe participant closely during the infusion of investigational product and for at least 30 minutes after IV infusion of investigational product has ended for any acute reactogenicity.
- Every 30 minutes after IV infusion of investigational product, for the 1st 4 hours, and every hour thereafter through 6 hours post-infusion, the study staff will:
  - Record vital signs (pulse, respiratory rate, blood pressure and temperature)
  - Assess any local and systemic reactogenicity
  - Assess any other adverse events
- Collect PK samples according to the Schedule of Procedures

6.3 Post-IV infusion of Investigational Product Visits

The participant will be asked to return to the clinic for post-investigational product infusion visits as indicated in the schedule of procedures (see appendices A, B and C) for an assessment by clinic staff. The participant will be asked to maintain a Memory Aid to track any local and systemic reactogenicity the participant experiences, including temperature, from the day of investigational product infusion for the next 3 days (for a total of 4 days including day of investigational product infusion). Participants will receive a thermometer to take their oral temperature at home once a day. Study staff will review the Memory Aid with the participant and determine the severity of the reactions through discussion with the participant.

The following procedures will be conducted at these visits:

- Review interim medical history
- Collect concomitant medication information
- Perform a symptom-directed physical examination if any signs or symptoms are present
- Assess vital signs (pulse, respiratory rate, blood pressure and temperature)

- Assess any adverse events and local and systemic reactogenicity (Days 1, 2, 3) including reviewing the Memory Aid.
- Collect specimens for all tests as indicated in the Schedule of Procedures (Appendices A, B and C and AP).

6.4 Additional Follow-up Visits

Assessments and procedures will be performed according to the Schedule of Procedures (Appendices A, B and C).

6.5 Unscheduled Visits

Unscheduled Visits/Contacts are visits/contacts that are not described in the Schedule of Procedures (Appendices A, B and C). Unscheduled visits may occur any time during the study:

- For administrative reasons, e.g., the participant may have questions for study staff or may need to re-schedule a follow-up visit.
- To obtain laboratory test results from a previous visit.
- For other reasons as requested by the participant or site investigator.

All unscheduled visits will be documented in the participants' study records on applicable source documents and entered into the Case Report Form (CRF).

6.6 Final Study Visit or Early Termination Visit

Assessments and procedures will be performed according to the Schedule of Procedures (Appendices A, B and C).

7.0 STUDY PROCEDURES

7.1 Informed Consent Process

A Master Informed Consent Document consisting of a Participant Information Sheet and a Consent Form is provided by the Sponsor to the trial site. This document is made site-specific and translated (if necessary), submitted and approved by the Institutional Review Board (IRB). The Master and site specific Informed Consent Documents are separate documents and should not be part of the protocol.

Participant Information Sheet
A qualified member of the study staff will conduct the informed consent process by reviewing the Participant Information Sheet and document in the clinic notes.

Consent Form
The participant's consent to participate must be obtained by him/her signing and dating the Consent Form. The person obtaining consent will also sign.

The signed and dated Informed Consent Document must remain at the study site. A copy of the signed/marked and dated Informed Consent Document will be offered to the participant to take home. Those participants who do not wish to take a copy will be required to document that they declined to do so.

7.2 Medical History and Physical Examination

Medical History
At screening, a comprehensive medical history will be collected including previous IV infusions and reaction to IV infusion, history of sexually transmitted infection (STI) and pregnancy prevention practices. At subsequent visits, an interim medical history will be performed.

Physical Examination

General Physical Examination
A general physical examination includes examination of head/ears/eyes/nose and throat, skin, respiratory, cardiovascular, abdominal, limited neurological and musculoskeletal and external ano-genital systems (for HIV-infected participants only) at the time points indicated in the Schedule of Procedures (see Appendices A, B and C).

Symptom-Directed Physical Examination
A symptom-directed physical examination is a targeted examination based on the participant's history or observation. If deemed necessary, this examination should be done at the time points indicated in the schedule of procedures (see Appendices A, B and C).

Measuring Height and Weight
Includes measuring the height and weight at the time points indicated in the Schedule of Procedures (see Appendices A, B and C).

Vital Signs

Vital signs including pulse, respiratory rate, blood pressure and temperature are measured and recorded at the time points indicated in the Schedule of Procedures (see Appendices A, B and C)

7.3 HIV Testing and HIV-test Counselling (Groups 1 and 2)

Study staff will perform pre-HIV test counselling prior to collecting blood for an HIV test, and post-HIV test counselling when HIV test results are available. This is referred to as HIV-test counselling, and done according to the CDC guidelines. For more information on HIV testing and HIV-test counselling, see Section 11.0. A screening questionnaire and other tools may be used.

7.4 HIV Risk Reduction Counselling

HIV risk reduction counselling will be provided to all participants as outlined by site-specific SOPs.

Study staff will provide HIV risk reduction counselling based on reported individual risk and provide free condoms, as appropriate, at every visit. Group 1 and 2 will receive HIV risk reduction counselling and for Groups 3, HIV risk reduction counselling will be conducted as secondary prevention to reduce onward transmission.

7.5 Family Planning Counselling

Study staff will counsel participants about the importance of preventing pregnancies and of using condoms, as well as other effective family planning methods until at least 3 months following investigational product administration, as appropriate. Participants may be referred for family planning services as necessary according to site-specific SOPs as detailed in the SOM. Pregnancy prevention methods chosen and compliance will be documented.

7.6 ART Counselling (Group 3)

All HIV-infected participants will receive ART counselling upon entering the study and will be asked to postpone ART initiation for 56 days following infusion of investigational product. Upon completion of the 56 days, all HIV-infected participants will receive ART counselling again and will be asked to initiate ART with their HIV healthcare provider. At all counselling sessions, participants will be advised to discuss this study regarding delaying or starting ART with their HIV healthcare provider. Participants who have not initiated or made plans to initiate ART by the final study visit (day 168) will receive ART counselling again.

HIV-infected participants who are not on ART who achieve an undetectable HIV RNA level after IV infusion of PGDM1400 ± PGT121 and who decide against ART initiation at day 56 despite ART counselling (as above), will be followed until HIV RNA becomes detectable or until the final study visit at which point these participants will be counseled again to initiate ART. HIV RNA in these participants will be assessed at every study visit, (see schedule of procedures, appendix C) and, if needed, participants will be approached for additional HIV RNA level assessments in between planned study visits in order to track individuals HIV RNA levels as needed.

7.7 Specimens

Approximately 50 ml of blood will be collected from participants in Group 1 and 2, and approximately 150 ml of blood will be collected from participants in Group 3 at the screening visit. At later visits, approximately 8.5 ml to 175 ml of blood will be collected, depending on study procedures and Group assignment (see Appendices A, B and C), usually from the antecubital fossa.

Optional collection of rectal and/or cervical mucosal secretions will be obtained using a rectal sponge (or comparable swab) or cervical Softcup (or comparable cervical fluid collection cup) for those participants that consent.

All specimens will be handled according to the procedures specified in the AP and relevant SOPs if applicable.

In the event of an abnormal laboratory value, participants may be asked to have an additional sample collected at the discretion of the Principal Investigator or designee.

7.8 Reimbursement

Participants will be reimbursed for their time, effort and for costs to cover their travel expenses to the study site and any inconvenience caused due to study participation. Site specific-reimbursement amounts will be documented in the site-specific Participant Information Sheet, and approved by the Institutional Review Board.

7.9 Randomization and Blinding

Participants will be identified by a unique study identification number.

Participants will be randomized according to the randomization schedule prepared by the statisticians at the Data Coordinating Center (DCC) prior to the start of the study. Participants will be automatically assigned a specific allocation number as they are enrolled into the data entry system. An unblinding list (Pharmacy List) will be provided to the unblinded site pharmacist by the DCC.

This a double blind, randomized, placebo-controlled study for Groups 1 and 2, and an open label study for Group 3. For Groups 1 and 2, study staff (investigator and clinical personnel monitoring the safety and laboratory assay results) and participants will be blinded with respect to the allocation of Investigational Product (PGDM1400 mAb alone or PGDM1400 and PGT121 in combination or placebo). A site pharmacist will be unblinded for the purposes of preparing investigational product.

A participant will be considered enrolled once he/she has been assigned an allocation number.

Blinded participants will be informed about their assignment (active product/placebo) at study completion, once the database is locked. Should a study participant be unblinded during the study, the study participant will be followed up until the end of the study according to the Schedule of Procedures (Appendices A and B).

7.10 Un-blinding Procedure for Individual Participants

Un-blinding of an individual participant may be indicated in the event of a medical emergency if the clinical management of the participant would be altered by knowledge of the treatment assignment.

The un-blinded information should be restricted to a small Group of individuals involved in clinical management/medical treatment of the participant (e.g., treating physician) and the blind must be maintained for those responsible for the study assessments.

The reasons for un-blinding should be documented and the IAVI Chief Medical Officer, the Medical Monitor and the DCC should be notified as soon as possible. The procedures and contact numbers for un-blinding are outlined in the SOM.

7.11 Assessment of PGDM1400 mAb and/or PGT121 mAb related HIV sero-positivity

It is possible that PGDM1400 and/or PGT121 or an immune response to PGDM1400 and/or PGT121 could cause a positive result on a diagnostic HIV antigen/antibody test. A Group 1 or 2 participant who tests HIV antigen and/or HIV antibody positive at the end of the study will have additional testing to distinguish actual HIV infection from investigational product-related responses. The participant will be informed of his/her positive HIV antigen and/or HIV antibody test result and offered continuing follow-up until the HIV antigen/antibody test becomes negative.

8.0 INVESTIGATIONAL PRODUCT

8.1 Description

PGDM1400 and PGT121 active products are formulated in a 20 mM Acetate, 9% Sucrose, 0.008% polysorbate 80, pH 5.2 formulation buffer at a concentration of 50 mg/mL. Each 10 ml vial contains 6 ml of PGDM1400 or PGT121.

A summary of the Investigational Products and example volumes needed for administration for each dose level are shown in Table 8.1-1.

Table 8.1-1 Investigational Products

**Active Product / Placebo**	**Dosage level**	**Total volume in investigational product container**	**Total Active Product or placebo volume to be injected into a 100 mL saline IV bag** (for an 88 kg body weight**)	**Total volume to be Infused** (for an 88 kg body weight**)
PGDM1400 (50 mg/mL)	3 mg/kg	6 mL per vial	5.3 mL	105.3 mL
	10 mg/kg		17.6 mL	117.6 mL
	30 mg/kg		52.8 mL	152.8 mL
PGT121 (50 mg/mL)	3 mg/kg	6 mL per vial	5.3 mL	105.3 mL
	10 mg/kg		17.6 mL	117.6 mL
	30 mg/kg		52.8 mL	152.8 mL

Placebo: 0.9% Sodium Chloride for Injection USP (Saline)*	3 mg/kg matching***	NA	5.3 mL***	105.3 mL***
	10 mg/kg matching***		17.6 mL***	117.6 mL***
	30 mg/kg matching***		52.8 mL***	152.8 mL***

* The Placebo provided will be a commercially-available 0.9% sodium chloride for injection USP partial addition IV bag.
** The actual volume to be injected will be based on the dose Subgroup and the weight of the participant at the time of investigational product administration. The example included here is the average weight of an adult male in the US (88kg) (http://www.cdc.gov/nchs/data/series/sr_11/sr11_252.pdf)
*** For placebo IV infusions: saline from an additional IV bag will be injected into the saline IV bag intended for administration, to match the volume used for a PGDM1400 ± PGT121 mAb injection in the same dose Subgroup, to prevent unblinding. Placebo recipients in Group 2 will receive 2 sequential administrations of placebo to mimic administration of PGDM1400 followed by PGT121 to maintain blinding. See SOM for details.

8.2 Shipment and Storage

Authorization to ship the PGDM1400 and PGT121 to the site will be provided in writing by the Sponsor, upon confirmation that all required critical documents for shipment authorization are completed. PGDM1400 and PGT121 will be shipped maintaining the required storage conditions and stored in a secure location in the clinical site's pharmacy.

PGDM1400 and PGT121 vials will be stored at - 20°C ± 5°C. Each vial will be labelled with the name of the product, lot number, concentration, fill volume, storage temperature, date of manufacture, name and location of the manufacturer and a US cautionary statement. Several such vials will be packaged in a box. Each box will be labelled with similar information as the vial label, including an address and contact information for the manufacturer.

0.9% Sodium Chloride for Injection USP, in partial-addition bags, will be used as the placebo and diluent for PGDM1400 and PGT121. It will be stored at room temperature.

8.3 Preparation of investigational product

Detailed instructions will be provided to the site pharmacist in the SOM for preparing each of the investigational products. The site pharmacist will not be blinded, but the study physician/designee administering the investigational product will be blinded. Infusion of the investigational product(s) should be completed within 4 hours of the PGDM1400 or PGT121 being injected into the saline IV bag. Example calculations for the volumes needed for IV infusion are illustrated in Table 8.1-1. Procedures for handling used and partially used vials of PGDM1400 and PGT121 will be provided in the SOM. Syringes or other components in direct contact with PGDM1400 or PGT121 will be disposed of properly in a biohazard container and incinerated or autoclaved as per site procedure.

8.4 Administration of investigational product

Investigational product will be administered at the enrollment visit. The investigational product will be injected into a 0.9% sodium chloride for injection USP partial addition

bag. The participant will receive the investigational product via IV infusion. If more than one investigational product will be administered, these will be administered sequentially in separate saline IV bags. Participants will receive each infusion over approximately 60 minutes, allowing for clinician discretion. Further information on the IV infusion of the investigational product is supplied in the SOM and other study documents.

8.5 Accountability and Disposal of investigational product

All used PGDM1400 and PGT121 vials will be handled according to instructions in the SOM. Throughout the study, the investigational product accountability forms including receipt and dispensing of vials will be kept and monitored. The vial label for the used vial(s) will be removed and retained for accountability purposes; the used vial(s) can be discarded as per site procedures.

At the end of the study, the unused PGDM1400 and PGT121 vials will be reconciled according to Sponsor instructions.

Further information on accountability and disposal of PGDM1400 and PGT121 is supplied in the SOM.

9.0 ASSESSMENTS

9.1 Safety Assessments

Data on local and systemic reactogenicity (i.e., solicited AEs) will be collected by structured interview and medical examination. Data on other adverse events will be collected with open-ended questions. All data will be recorded on the appropriate source documents and entered into the study database. Participants will be given a Memory Aid, which is a tool to assist with collecting reactogenicity data.

Local and systemic reactogenicity events will be assessed by study staff prior to and during IV infusion of investigational product, approximately every 30 minutes after investigational product infusion for the first 4 hours after investigational product infusion and subsequently every hour for the first 6 hours post-investigational product infusion. Study staff will review the Memory Aid with the participant, and determine the severity of the reactions on days 1-3 through discussion with the participant.

9.1.1 Local reactogenicity

The presence of local reactogenicity will be assessed at the time points specified in the Schedule of Procedures (Appendices A, B and C).
Pain, tenderness, erythema/skin discoloration, swelling/hardening or pruritus will be assessed and graded using Appendix G, DAIDS Adverse Event Severity Assessment Table, as a guideline.

9.1.2 Systemic reactogenicity

The presence of systemic reactogenicity will be assessed at the time points specified in the Schedule of Procedures (Appendices A, B and C).
Fever, chills, headache, nausea, vomiting, malaise, myalgia and arthralgia will be assessed and graded using the Appendix G DAIDS Adverse Event Severity Assessment Table as a guideline.

For the first 24 hours after investigational product infusion, any infusion related reactions, including cytokine release syndrome, should be graded using the Common Terminology Criteria for Adverse Events (CTCAE) v4.03: June 14, 2010 (Appendix F).

9.1.3 Vital Signs

At the infusion visit, vital signs (pulse, respiratory rate, blood pressure and temperature) will be measured by study staff prior to investigational product infusion, approximately every 30 minutes for the first 4 hours post investigational product infusion and then hourly until 6 hours after IV infusion. For the other study visits vital signs will be assessed at the time points specified in the Schedule of Procedures (Appendices A, B and C).

9.1.4 Other Adverse Events

Other adverse events (AEs) will be collected through 56 days after investigational product infusion in all participants. Serious Adverse Events (SAEs) will be collected throughout the entire study period. Potential Immune Mediated Diseases (pIMDs), as defined in Section 10.5, will be collected throughout the study period, using the SAE reporting process. Open-ended questions will be asked at time points according to the Schedule of Procedures (Appendices A, B and C). All adverse events during the first 24 hours after the infusion will be graded using Appendix G, DAIDS Adverse Event Severity Assessment Table, as a guideline and will be assessed for relatedness to the investigational product. For more information regarding adverse events refer to Section 10.0, Adverse Events.

9.1.5 Concomitant Medications

Concomitant receipt of Investigational Products is prohibited during the study.

Contraceptive use and use of medication at study entry will be documented. (See DCF instructions).

During the study, information regarding concomitant medications and reasons for their use will be solicited from the study participants for 56 days. Ongoing concomitant medications will be recorded until end of study.

9.1.6 Routine laboratory parameters

Table 9.1.6-1 shows the laboratory parameters that will be measured routinely. The samples for these tests will be collected at the time points indicated in the Schedule of Procedures (Appendices A, B and C).

Table 9.1.6-1: Laboratory Parameters

Laboratory Parameter	Test
Hematology and Coagulation	Hemoglobin, hematocrit, leukocytes, platelets, absolute neutrophil count (ANC), absolute lymphocyte count (ALC), activate partial thromboplastin time (aPTT), international normalized ratio (INR)

Clinical Chemistry	Sodium, potassium, creatinine, aspartate aminotransferase (AST), alanine aminotransferase (ALT), total bilirubin, alkaline phosphatase<br>Groups 1 and 2 only: albumin, creatine kinase, C-reactive protein, C3 complement, C4 complement
Urinalysis	Dipstick test for protein, blood glucose, ketones, esterase (leukocytes) and nitrite. If clinically significant abnormalities (e.g., blood, protein, leukocytes) are found on dipstick test, then further test(s) will be performed (e.g., microscopy, culture)
T cell panel (Group 3)	CD4 T cell count and frequency by single platform flow cytometry

9.1.7 Specific screening tests:

Participants will be screened to exclude the following diseases:

- Hepatitis B: positive for hepatitis B surface antigen (HBsAg)
- Hepatitis C: positive for hepatitis C RNA (HCV antibody test, followed by HCV RNA test if HCV antibody positive)
- Chlamydia: confirmed diagnosis
- Gonorrhea: confirmed diagnosis
- Active syphilis: confirmed diagnosis.

A negative Hepatitis B and Hepatitis C result can be documented from the medical record only if the result is from a test administered less than 6 months ago.

9.1.8 Monitoring for anti-PGDM1400 and anti-PGT121 antibodies:

Participants will be evaluated for the development of antibodies to PGDM1400 and PGT121 mAb (anti-drug antibodies, ADA) by ELISA according to the Schedule of Procedures (Appendices A, B and C).

9.2 Virologic Assessments

Table 9.2-1 shows the virologic parameters that will be measured routinely. The samples for these tests will be collected at the time points indicated in the Schedule of Procedures (Appendix C).

Table 9.2-1: Virologic Assessment Table

**Virologic Parameter**	**Test**
Antiviral Activity	Plasma HIV RNA levels
Anti-reservoir activity	Cell-associated HIV-1 RNA levels in resting CD4 T cells; total HIV-1 DNA and 2-long terminal repeat (LTR) HIV-1 DNA circles in resting or total CD4 T cells; quantitative viral outgrowth assay (qVOA)
Other	Genotyping of plasma HIV RNA for evaluation of PGDM1400 and PGT121-induced escape mutations and resistance to antiretroviral drugs; phenotyping of plasma HIV RNA for

	neutralization susceptibility to PGDM1400 and PGT121 in-vitro

9.3 Exploratory Immunogenicity Assessments

Humoral immune response assays will include, but are not limited to Env-specific Ab-binding assays, virus neutralization assay, and assays for Ab functionality. Cellular immune response assays will include, but are not limited to IFNγ ELISPOT assay, ICS, and multiparameter flow cytometry. Exploratory assessments on mucosal samples will include, but are not limited to characterization of Env-specific binding Abs. Priority assays are listed below.

9.3.1 Antibody Responses

- Env-specific binding Abs (titers and breadth).
- Env-specific nAbs (titers and breadth).
- Env-specific functional Abs (e.g. phagocytosis score and breadth).
- Env-specific binding Ab isotypes (IgA, IgG1-4) (titers and breadth).

9.3.2 Cellular Responses

- IFNγ peripheral blood mononuclear cell (PBMC) responders to peptide pools and subpools of Potential T-cell epitopes, PTE Env/Gag/Pol peptides.
- $CD4^+$ and $CD8^+$ T-cell functionality (% cells producing e.g., IFNγ, IL-2, IL-4, TNFα).
- T-cell development with emphasis on follicular helper T-cells and memory differentiation.

9.3.3 PBMC, Serum and Plasma Storage

Samples of cryopreserved PBMC, plasma and serum will be stored as indicated in the Schedule of Procedures in Appendices A, B and C (for details see Analytical Plan (AP)) and, if the participant consents, may be used for the purposes of standardization, quality control and for future assays related to HIV prevention or treatment research and development. These samples will be archived and the testing laboratories will be blinded to the participant's identity.

9.4 Other Assessments

9.4.1 HIV Antibody Testing (Groups 1 and 2)

All HIV-uninfected participants (Group 1 and 2) will be tested for HIV antibodies as indicated in the Schedule of Procedures (Appendix A and B) or as needed, if medical or social circumstances arise. All participants will receive HIV risk reduction counselling and pre- and post-HIV-test counselling, as specified in Section 7.3 Counselling.

9.4.2 Pharmacokinetics

Blood draws for pharmacokinetics will be done on the day of investigational product infusion immediately before starting IV infusion(s) of investigational product, at the end of the investigational product infusion(s), and 3, 6 and 24

hours after the investigational product infusions. Thereafter, pharmacokinetic draws will be done as indicated in the Schedule of Procedures (Appendices A, B and C).

PGDM1400 and PGT121 mAb pharmacokinetic analyses will be performed using, but not limited to, standard non-compartmental analysis methods to estimate elimination half-life (t1/2), clearance (CL/F), volume of distribution (Vz/F), Area under the concentration decay curve (AUC), impact of viral load and/or ART on PGDM1400 and PGT121 mAb disposition (elimination half-life (t1/2), clearance (CL/F), volume of distribution (Vz/F) and total exposure. PGDM1400 ± PGT121 accumulation will also be examined in rectal and cervical mucosal secretions collected with rectal sponges (or comparable swab) or cervical Softcups (or comparable cervical fluid collection cup) in study participants who specifically consented for these procedures. Descriptive results will be reported for the pharmacokinetic parameters by dose Subgroup.

Exploratory analysis using population analysis methods simultaneously combining all pharmacokinetic data across all doses and treatment Subgroups and Groups will be performed for quantitative characterization of differences in PGDM1400 and PGT121 mAb disposition by dose, participant Subgroup or Group or disease state.

9.4.3 HLA Typing

Samples for HLA typing will be collected as specified in the Schedule of Procedures in Appendices A, B and C (for details see Analytical Plan (AP)) and may be analyzed as warranted.

9.4.5 Pregnancy Test

A urine pregnancy test for all female participants will be performed by measurement of human chorionic gonadotrophin (βhCG) at time points indicated in the Schedule of Procedures (Appendices A, B and C). The results of the pregnancy test must be negative prior to IV infusion of investigational product. See section 10.7 for description of pregnancy after infusion of investigational product.

9.4.6 HIV Risk Assessment (Group 1 and 2)

Study staff will assess participants for their past and current risk of acquiring HIV at time points indicated in Schedule of Procedures (Appendix A and B).

9.4.7 Social Impact Assessment

A brief assessment of the impact of participation in the study will be administered to participants at their final study visit.

10.0 ADVERSE EVENTS

10.1 Definition

An adverse event (AE) is any untoward medical occurrence in a participant administered an Investigational Product and which does not necessarily have a causal relationship with the Investigational Product. An AE can therefore be any unfavourable or

unintended sign (including an abnormal laboratory finding), symptom, or disease, temporally associated with the use of Investigational Product whether or not related to the Investigational Product.

Assessment of severity of all AEs, including and seriousness of AEs, is ultimately the responsibility of the Principal Investigator of each site. Refer to the DIVISION OF AIDS (DAIDS) TABLE FOR GRADING THE SEVERITY OF ADULT AND PEDIATRIC ADVERSE EVENTS Version 2.0, November 2014 and the National Cancer Institute Common Terminology Criteria for Adverse Events (CTCAE) Version 4.03: June 14, 2010 for additional guidance.

10.2 Assessment of Severity of Adverse Events

The following general criteria should be used in assessing adverse events as mild, moderate, severe or very severe at the time of evaluation:

Grade 1 (Mild): Symptoms causing no or minimal interference with usual social & functional activities

Grade 2 (Moderate): Symptoms causing greater than minimal interference with usual social & functional activities

Grade 3 (Severe): Symptoms causing inability to perform usual social & functional activities

Grade 4 (Very Severe): Symptoms causing inability to perform basic self-care functions OR Medical or operative intervention indicated to prevent permanent impairment, persistent disability, or death

Guidelines for assessing the severity of specific adverse events and laboratory abnormalities are listed in Appendix G, DAIDS Adverse Event Severity Assessment Table.

10.3 Relationship to Investigational Product

Assessment of relationship of an AE or SAE to Investigational Product is the responsibility of the Principal Investigator or designee. All medically indicated and available diagnostic methods (e.g., laboratory, blood smear, culture, X-ray, etc.) should be used to assess the nature and cause of the AE/SAE. Best clinical and scientific judgment should be used to assess relationship of AE/SAEs to the investigational product and/or other cause.

The following should be considered:

- Presence/absence of a clear temporal (time) sequence between administration of the investigational product and the onset of AE/SAE
- Presence/absence of another cause that could more likely explain the AE/SAE (concurrent disease, concomitant medication, environmental or toxic factors)
- Whether or not the AE/SAE follows a known response pattern associated with the investigational product

The relationship assessment should be reported as one of the following:

Not Related: clearly explained by another cause (concurrent disease, concomitant medication, environmental or toxic factors, etc.).

Unlikely: more likely explained by another cause (concurrent disease, concomitant medication, environmental or toxic factors, etc.).

Possibly: equally likely explained by another cause but the possibility of the investigational product relationship cannot be ruled out (e.g., reasonably well temporally related and/or follows a known investigational product response pattern but equally well explained by another cause).

Probably: more likely explained by the investigational product (e.g., reasonably well temporally related and/or follows a known investigational product response pattern and less likely explained by another cause).

Definitely: clearly related and most likely explained by the investigational product.

For the purpose of expedited safety reporting, all possibly, probably or definitely related SAEs are considered investigational product-related SAEs.

10.4 Serious Adverse Events

An adverse event is reported as a "Serious Adverse Event" if it meets any of the following criteria (as per International Conference on Harmonisation [ICH] Good Clinical Practice [GCP] Guidelines):

- Results in death
- Is life threatening
- Results in persistent or significant disability/incapacity
- Requires in-participant hospitalization or prolongs existing hospitalization
- Is a congenital anomaly/birth defect or spontaneous abortion
- Any other important medical condition that requires medical or surgical intervention to prevent permanent impairment of a body function or structure

Elective surgery for pre-existing condition that did not increase in severity or frequency is not considered an SAE.

Serious Adverse Events (SAEs) should be reported within 24 hours of the site becoming aware of the event, and sent to the Sponsor as described in the SOM.

To discuss investigational product-related SAEs or any urgent medical questions related to the SAE, the site investigator should contact one of the IAVI Medical Monitors directly (see Contact List in the SOM).

The IAVI SAE Report Form should be completed with all the available information at the time of reporting and sent to the Sponsor as described in the SOM. The minimum data required in reporting an SAE are the study identification number, date of birth, gender, event description (in as much detail as is known at the time), onset date of event (if available), reason event is classified as serious, reporting source (name of Principal Investigator or designee), and relationship to the investigational product as assessed by the investigator.

The Principal Investigator or designee is required to prepare a detailed written report with follow up until resolution or until it is judged by the Principal Investigator or designee to have stabilized.

The Principal Investigator or designee must notify the local IRB/IEC of all SAEs as appropriate. In case of investigational product-related SAEs, the Sponsor will notify responsible regulatory authorities, Safety Monitoring Committee (SMC), and other study sites where the same investigational product is being tested.

More details on SAE definitions and reporting requirements are provided in the SOM.

Serious Event Prior to Investigational Product Administration

If a serious event occurs in the period between the participant signing the Informed Consent Form and receiving the IV infusion of investigational product, the event will be reported using the SAE form and following the same procedures for SAE reporting, as indicated in Section 10.4. The timing of the event will be indicated by using the relevant checkbox on the SAE form.

10.5 Reporting Potential Immune-Mediated Diseases

Potential immune-mediated diseases (pIMDs) are a subset of AEs that include both clearly autoimmune diseases and also other inflammatory and/or neurologic disorders that may or may not have an autoimmune etiology. These events are of special interest since they could potentially be caused by immune responses to the investigational product. The investigator/designee should report such adverse events within the same time limits (following confirmation of an AE as a pIMD; see last paragraph of this section below), and using the same CRF pages, as utilized for SAEs. The investigator or his/her designee will evaluate the occurrence of pIMDs at every visit/contact during the study. IAVI will also expect investigators/designee to provide additional information about pIMD events. AEs to be reported and documented as pIMDs include:

Neuroinflammatory disorders: optic neuritis, cranial nerve disorders (including Bell's palsy), multiple sclerosis, demyelinating disease, transverse myelitis, Guillain-Barré syndrome, myasthenia gravis, encephalitis, neuritis.

Musculoskeletal disorders: systemic lupus erythematosus, cutaneous lupus, Sjögren's syndrome, scleroderma, dermatomyositis, polymyositis, myopathy, rheumatoid arthritis and juvenile rheumatoid arthritis, polymyalgia rheumatica or temporal arteritis, reactive arthritis, psoriatic arthropathy, ankylosing spondylitis, undifferentiated spondyloarthropathy.

Gastrointestinal disorders: Crohn's disease, ulcerative colitis or proctitis, celiac disease.

Metabolic diseases: autoimmune thyroiditis, Grave's or Basedow's disease, Hashimoto thyroiditis, insulin-dependent diabetes mellitus (IDDM), Addison's disease.

Skin disorders: psoriasis, vitiligo, Raynaud's phenomenon, erythema nodosum, autoimmune bullous skin diseases.

Others: autoimmune hemolytic anemia, thrombocytopenia, antiphospholipid syndrome, *vasculitis, pernicious anemia, autoimmune hepatitis, primary biliary cirrhosis, primary sclerosing cholangitis, autoimmune glomerulonephritis, autoimmune uveitis, autoimmune myocarditis/cardiomyopathy, sarcoidosis, Stevens-Johnson syndrome, Behçet's syndrome.

Infusion site reactions: Grade 3 or 4 infusion site reactions lasting more than 2 days.

*Vasculitis: Vasculitis, Diffuse vasculitis, leucocytoclastic vasculitis, polyarteritis nodosa, microscopic polyangiitis, Wegener's granulomatosis, anti-neutrophil cytoplasmic antibody positive vasculitis, Henoch-schönlein purpura, allergic granulomatous angiitis (Churg-Strauss disease), Kawasaki disease, Takayasu's arteritis, temporal arteritis (giant cell arteritis), renal vasculitis.

Medical judgement should be exercised in deciding whether other disorders/diseases have an autoimmune origin and should also be reported as described above, and this judgement is the investigator's prerogative. Whenever sufficient data exist to substantiate any of the diagnoses in the above list, the event must be reported as a pIMD. While the intent of pIMD reporting is to be inclusive, isolated nonspecific symptoms, which might (or might not) represent the above diagnoses, should be captured as AEs but not reported as pIMDs until the diagnosis can be defended.

10.6 Clinical Management of Adverse Events

Adverse events (AEs) will be managed by the clinical study team who will assess, provide first line of care as appropriate and refer to health care and treatment facilities as warranted. If any treatment/medical care is required as a result of the harm caused by the investigational product or study procedures, this will be provided free of charge.

If a participant has an AE and/or abnormal laboratory value that is known at the time of IV infusion of investigational product, the specifications of Section 12.0 will be followed.

Participants will be followed until the AE resolves or stabilizes or up to the end of the study, whichever comes last. If at the end of the study, an AE (including clinically significant laboratory abnormality) that is considered possibly, probably or definitely related to the investigational product is unresolved, follow-up will continue until resolution if possible and/or the participant will be referred.

If a participant from Group 3 experiences a significant decrease in CD4 cell count (e.g., – 20% of baseline, or decline to <200 cells/µL) during the course of the trial, participants will be monitored closely until their CD4 count returns to baseline or until the participant initiates ART. Participants whose CD4 cell counts decrease to <200 cells/µL will be promptly informed and will be referred to their primary HIV care provider. Appropriate prophylaxis against opportunistic infections will be instituted according to accepted U.S. HIV treatment guidelines.

10.7 Clinical Management of Infusion-Related Reactions and Stopping Criteria

Infusion-related reactions can be local or systemic. Depending on the severity of the reaction, administration of IP can continue, be paused or stopped. Use clinical judgement and follow site-specific SOPs for anaphylaxis / infusion-related reactions. At a minimum, the following medications should be available for immediate use during the infusion: 25 mg IV diphenhydramine x 1, 125 mg IV methylprednisolone x 1, epinephrine (1:1000) 0.5 mg/ 0.5 mL IV x 1, albuterol inhaler, and albuterol nebulizer. These medications should be included with the prescription for IP, if necessary (some sites may stock these medications on site already), so that they are available at the bedside to be administered consistent with site SOPs for anaphylaxis / infusion-related reactions.

For infusion related reactions, including cytokine release syndrome, use the Common Terminology Criteria for Adverse Events (CTCAE), Version 4.03 June 2010 grading scale.

Infusion stopping criteria:

Grade	
1	Monitor participant closely, intervention not indicated
2	Stop and re-start if clinically acceptable
3	Stop, do not re-start
4	Stop, do not re-start

Mild reaction (Grade 1)

- Inform medical staff
- Monitor volunteer for signs of systemic reaction. If there is no sign of systemic reaction continue the infusion of IP at the same rate
- Continue to monitor volunteer, manage volunteer according to local guidelines and SOPs
- Document the events
- Do not continue the infusion of any IP (1st or 2nd) if there is any concern
- If infusion of 1st IP is not continued, do not proceed with infusion of 2nd IP

Systemic reaction (Grade 2)

- Stop administration of IP at the catheter level, i.e., as close to the arm as possible to prevent any more IP from entering the body. Remember that there is IP in the intravenous line downstream from the IP IV saline bag (removing the IP IV saline bag and replacing with a 0.9% saline bag or another IV fluid on the 1st intravenous line will result in additional administration of the several ml of IP that is in the line).
- Inform medical staff
- Monitor volunteer and continue to monitor until the volunteer is stable
- Ensure that 0.9% saline is running on the 2nd intravenous line
- The Principal Investigator, or study physician on call, must decide whether to restart or stop the administration of IP
  - If the volunteer improves discuss restarting infusion of IP, and at what rate, with Principal Investigator, or study physician on call.
  - If the volunteer does not improve or condition becomes worse, commence basic resuscitation according to site guidelines and SOPs and administer intravenous

methylprednisolone and intravenous diphenhydramine. Replace the intravenous line connected to the 1st intravenous catheter (these lines contain IP).

- Document the events

Cytokine Release Syndrome or anaphylactic or anaphylactoid reaction (Grade 3 or 4)

- Stop administration of IP at the catheter level, i.e., as close to the arm as possible to prevent any more IP from entering the body. Replace the intravenous line connected to the 1st intravenous catheter (these lines contain IP).
- Call for assistance
- Monitor volunteer and continue to monitor until the volunteer is stable
- Ensure that 0.9% saline is running on the 2nd intravenous line
- Commence management of anaphylaxis according to site guidelines and SOPs
- Document the events

10.8 Pregnancy

Although not considered an AE, if a female participant becomes pregnant during the study, it is the responsibility of the Principal Investigator or designee to report the pregnancy promptly to IAVI using the designated forms. The participant will be followed for safety until the end of pregnancy or study completion, whichever occurs last. If possible, approximately 2–4 weeks after delivery, the baby will be examined by a physician to assess its health status and the results will be reported to the Sponsor. The baby will be examined again by a Physician around age 1, and the results will be reported to the Sponsor.

Complications of pregnancy that meet criteria for SAEs, specified in Section 10.4 of this Protocol (e.g., hospitalization for eclampsia, spontaneous abortion, etc.) should be reported as SAEs.

10.9 Intercurrent HIV Infection (Group 1 and 2)

HIV infection cannot be directly caused by the investigational product. If a participant acquires HIV through exposure in the community, at any time after the IV infusion of investigational product, the participant should be offered referral to appropriate care and treatment facilities. The participant will continue to be followed in the study for safety assessments.

Intercurrent HIV infection in study participants, although not considered an SAE, must be reported promptly to IAVI using the designated forms. However, medical conditions associated with the HIV infection that meet criteria for being serious specified in the Section 10.4 of this Protocol (e.g., sepsis, *Pneumocystis jiroveci [carinii] pneumonia*, etc.) should be reported as SAEs using the SAE Report Form.

11.0 MANAGEMENT OF HIV ISSUES DURING AND FOLLOWING STUDY

11.1 HIV Testing – Groups 1 and 2

Group 1 and 2 participants will be tested for HIV antibodies as indicated in the Schedule of Procedures (Appendix A and B) or as needed, if medical or social circumstances

arise. All participants will receive HIV risk reduction counselling and pre- and post-HIV-test counselling, as specified in Section 11.3.1, Counselling (Group 1 and 2).

It is possible that the Investigational Product(s) or an immune response to the Investigational Product(s) could cause a positive result on a diagnostic HIV antibody test. An investigational product recipient who falsely tests HIV positive with a diagnostic HIV antibody test at the end of the study will be informed of his/her positive test result and offered continuing follow-up until the test becomes negative.

If a participant acquires HIV through exposure in the community, at any time after the administration of investigational product, the participant will be offered referral to appropriate care and treatment facilities. The participant will continue to be followed in the study for safety assessments.

Should a participant require HIV testing outside of the study for personal reasons, it is recommended that the participant contact the study staff first. HIV testing can be done at the study site and then processed at an independent laboratory as above. Written evidence of HIV status (HIV-infected or HIV-uninfected) will be provided upon request.

11.2 Social Discrimination as a Result of investigational product-related antibodies

In order to minimize the possibility of social discrimination in participants (if any) who test positive on a diagnostic HIV antibody test due to investigational product-related antibodies, appropriate diagnostic HIV testing and certification will be provided both during and after the study as needed.

11.3 HIV infection – Group 1 and 2

Group 1 and 2 participants who are diagnosed with HIV infection at screening or during the study (intercurrent HIV-infection) will be provided the following:

11.3.1 Counselling

The participant will be counselled by the study investigators or designated counsellors. The counselling process will assist the participant with the following issues:

- Psychological and social implications of HIV infection
- Who to inform and what to say
- Implications for sexual partners
- Implications for child-bearing
- Avoidance of transmission to others in future
- Mandatory reporting to the state, in some instances

11.3.2 Referral for Support/Care

Participants will be referred to a participant support center or institution of his/her choice for a full discussion of the clinical aspects of HIV infection. Referral will be made to a designated physician or center

12.0 WITHDRAWAL FROM STUDY

12.1 Deferral of IV infusion of investigational product

An IV infusion of investigational product may be temporarily deferred if the participant is clinically ill at the time of the administration of investigational product visit and/or presents with fever (> 100.4 °F; > 38.0 °C) at the time of the administration of investigational product. A participant must be clinically well and afebrile for a minimum of a 24-hour consecutive period prior to administration of investigational product.

Any planned or unplanned deferral of infusion of investigational product will be discussed with the Sponsor. Participants will be deferred from infusion of investigational product for any of the following reasons:

- Pregnancy
- A disease or condition or adverse event that may develop, regardless of relationship to Investigational Product, if the Principal Investigator or designee is of the opinion that administration of investigational product will jeopardize the safety of the participant
- Participant's request to defer infusion

The following events require resolution and/or review of clinical history by the Principal Investigator or designee and consultation with the Medical Monitor, prior to administration of investigational product:

- Any abnormal laboratory value, as outlined in section 5.7, Exclusion Criteria, Hematology, Chemistry, Urinalysis that is known at the time of infusion and have not resolved.
- Receipt of inactivated/killed/subunit vaccines (non-HIV) or immunoglobulin within the previous 14 days. Receipt of live attenuated vaccines within the previous 30 days.
- Participating in another clinical study of an Investigational Product

12.2 Withdrawal from the Study (Early Termination)

Participants may be withdrawn from the study permanently for the following reasons:

1. Participants may withdraw from the study at any time if they wish, for any reason
2. The Principal Investigator or designee has reason to believe that the participant is not complying with the protocol
3. If the Sponsor decides to terminate or suspend the study

If a participant withdraws or is withdrawn from the study, all termination visit procedures will be performed according to the Schedule of Procedures (Appendices A, B and C) where possible. Every effort will be made to determine and document the reason for withdrawal.

13.0 DATA HANDLING

13.1 Data Collection and Record Keeping at the Study Site

Data Collection: All study data will be collected by the clinical study staff using designated source documents and entered onto the appropriate electronic CRFs (eCRFs). Access to eCRFs will be provided via an electronic data entry system hosted by the Data Coordination Center. All study data must be verifiable to the source documentation. A file will be held for each participant at the clinic(s) containing all the source documents. Source documentation will be available for review to ensure that the collected data are consistent with the CRFs.

All CRFs and laboratory reports will be reviewed by the clinical team, who will ensure that they are accurate and complete.

Source documents and other supporting documents will be kept in a secure location. Standard GCP practices will be followed to ensure accurate, reliable and consistent data collection.

Source documents include but are not limited to:

- Signed Informed Consent Documents
- Progress notes
- Data collection forms
- Documentation of any existing conditions or past conditions relevant to eligibility
- Printed laboratory results
- Print out of the generated enrollment confirmation
- All Adverse Events
- Concomitant medications
- Local and systemic reactogenicity events

13.2 Data Entry at the Study Site

The data collected at the site will be recorded onto the eCRFs by the study staff and entered into a database. To provide for real time assessment of safety, data should be entered as soon as reasonably feasible after a visit occurs.

13.3 Data Analysis

The Sponsor, PIs and Product Developers will agree on how data will be analyzed and presented prior to unblinding of the study.

The DCC will conduct the data analysis and will provide interim safety and final study reports for the Sponsor, Principal Investigators, the PSRT and SMC and the regulatory authorities, as appropriate.

14.0 STATISTICAL CONSIDERATIONS

14.1 Safety and Tolerability Analysis

14.1.1 Sample Size

The sample size for safety and tolerability analysis will be 36-84 participants according to the dose escalation design used to characterize the safety profile of one IV infusion of PGDM1400 ± PGT121 mAb, at one of three dose levels.

14.1.2 Null Hypothesis

As this is an exploratory proof of concept trial and analysis will be descriptive, no formal null hypothesis will be tested.

14.1.3 Statistical Power and Analysis and Dose Escalation Rules

The frequency (percentage) of moderate or greater local and systemic reactogenicity events (along with 95% confidence intervals) will be determined for each active Group and placebo.

The frequency of SAEs judged possibly, probably or definitely related to the investigational product will be determined.

All AEs will be analyzed and grouped by seriousness, severity and relationship to the investigational product (as judged by the investigator).

For life-threatening adverse events related to investigational product: if none of the 18 (max 36) participants receiving active product experience such reactions, then the exact 95 % upper confidence bound for the rate of these adverse events in the population is 18.5% (or 9.7% if n=36).

All AEs will be analysed and grouped by seriousness, severity and relationship to the investigational product (as judged by the investigator).

For life-threatening adverse events related to active product: if none of the 9 (max 18) participants in either Group 1 or Group 2 who receive the active product experience such reactions then the exact 95% upper confidence bound for the rate of these adverse events in the population is 33.6% (or 18.5% if n=18).

An interim analysis of Subgroup and Group data will be carried out according to the study schema (Table 5.3.1) without unblinding the study to investigators or participants. At the end of the study, a full analysis will be prepared.

Based on previous experience with IAVI Phase 1 investigational product studies, it is expected that the amount of missing, unused or spurious data will be insignificant. Unused and spurious data will be listed separately and excluded from the statistical analysis. Missing data will be considered missing completely at random and excluded from the statistical analysis.

14.2 Pharmacokinetic Analysis

14.2.1 Sample Size

The sample size for pharmacokinetic analysis will be 3 per dose Subgroup, to provide sufficient information for the planned analyses.

14.2.2 Null Hypothesis

As this is an exploratory proof of concept trial and analysis will be descriptive, no formal null hypothesis will be tested.

14.2.3 Statistical Power and Analysis

Disposition of PGDM1400 mAb and PGT121 mAb will be evaluated in this study. Based on the PK profile of other human monoclonal antibodies, it is expected that the half-life of PGDM1400 mAb and PGT121 mAb will be 14 to 21 days. Previously published data indicate that the pharmacokinetics of VRC01, 3BNC117 and 10-1074 are fairly similar across phase 1 studies (Table).

Table 14.2.3-1: Key parameters of select Antibodies for the Prevention and Treatment of HIV Infection

	3BNC117 (Caskey, Klein et al. 2015)	VRC01 (Ledgerwood, Coates et al. 2015, Lynch, Boritz et al. 2015)	10-1074 (Caskey, Schoofs et al. 2017)
**Binding site on HIV ENV spike**	CD4 binding site	CD4 binding site	V3 loop
**Human safety data available**	Yes, safe and well tolerated	Yes, safe and well tolerated	Yes, safe and well tolerated
**PK HIV-uninfected, terminal half life (days)**	17	15	24
**PK HIV-infected & viremic, terminal half life (days)**	9	12 (i.v.), 11 (s.c.)	12.8
**Antiviral effect (average or range $\log_{10}$ decrease HIV RNA)**	1.48	1.1-1.8	1.52

Commonly reported PK parameters will be calculated using, but not limited to, standard non-compartmental slope/height/area/moment (SHAM) analysis methods. Summary descriptive results of PK parameters, including AUC, Cmax, T1/2, and clearance results will be reported by dose cohort. Dose normalized plots of PK parameters will be presented. Correlation between PK and reported safety and pharmacodynamic outcomes will also be explored parameters in order to examine exposure-effect relationships.

A more powerful exploratory analysis to quantitatively determine the dose, participant and disease impact on PGDM1400 mAb and PGT121 mAb pharmacokinetics, and correlate exposure with response, while correctly accounting for variance based on population intrinsic factors such as weight and gender will be performed. Using the proposed population analysis approach we will be able to simultaneously examine the magnitude and the rate of change to PGDM1400 and PGT121 disposition driven by HIV-1 RNA levels, and also examine the magnitude and the rate of decline in log copies/ml of HIV-1 RNA plasma levels from baseline.

The frequency and levels of anti-PGDM1400 antibodies and anti-PGT121 antibodies will be calculated and tabulated.

14.3. Virologic Analysis for Group 3

14.3.1 Sample Size

The sample size for virologic analysis in Groups 3A and 3B will be 12-36 participants according to the design described below.

14.3.2 Null Hypothesis

The null hypothesis is that there is a mixture of responders and non-responders with mixture probability 0.5 such that the difference-from-baseline is greater than -0.9 logs HIV RNA viral load in the responder group and 0 logs HIV RNA viral load in the non-responder group.

14.3.3 Statistical Power and Analysis

The virologic analysis described in this section relates to Subgroups 3A and 3B of the study design, in which antiviral activity of PGDM1400 mAb alone and a combination of PGDM1400 mAb + PGT121 mAb is measured in HIV-infected participants off ART with plasma HIV RNA levels of 1 x 10^3 – 1x 10^5 copies/ml. This section assumes that Part 1 of the study has successfully demonstrated that there are safe dose levels of PGDM1400 mAb, and PGDM1400 mab + PGT121 mAb. No placebo participants are enrolled as part of this design.

The primary efficacy outcome for this analysis is defined as change in log10 viral load between Day 0 (day of infusion) and Day 7. The minimum clinically significant value for this outcome is defined as a difference of –0.9 log10.

Groups 3A: this Group will enroll participants sequentially. After administering PGDM1400 at the MTD, as defined in part 1 of this study, the day 7 post-infusion viral load measurement will be compared to the baseline viral load measurement. If the difference in viral load at 7 days post-infusion is greater than a 0.5 log10 drop, the participant is categorized as a responder; otherwise, the participant is categorized as a non-responder. Enrollment will continue until at least 6 responders or 18 total participants have been enrolled whichever occurs first.

Group 3B: this Group will enroll participants sequentially. After administering PGDM1400 mAb and PGT121 mAb at the MTD, as defined in part 1 of this study, the day 7 post-infusion viral load measurement will be compared to the baseline viral load measurement. If the difference in viral load at 7 days post-infusion is greater than a 0.5 log10 drop, the participant is categorized as a responder; otherwise, the participant is categorized as a non-responder. Enrollment will continue until at least 6 responders or 18 total participants have been enrolled whichever occurs first.

Antiviral activity in Subgroup 3A and 3B will be tested in the responder group only using a one-sided Wilcoxon signed rank test against the null hypothesis of a "shift" parameter of -0.9 $\log_{10}$. Based on a simulation study outlined in the SAP, the power to reject the null hypothesis is 80% when the responder group has a difference-from-baseline viral load drop of approximately 1.8 logs for a nominal alpha level of 0.05. Since the responder group is defined by the outcome it is not surprising that the Type I error is greater than the nominal alpha level which was deemed an acceptable trade-off between sample size and power to detect the desired effect under the alternative described above. Notably, the Type I error drops below the nominal error rate when the true shift in viral load is in the responder group is greater than -0.5 logs.

For the analysis of sample size and power, log10 viral load differences from baseline for each participant were simulated from a normal distribution, with a standard deviation of 0.5. This value was chosen by examining a study of the antiretroviral drug raltegravir, which demonstrated a mean estimated standard deviation of the change of baseline of 0.47(Andrade, Rosenkranz et al. 2013). This is a conservative estimate, as the variability of viral loads near the lower range might be expected to also be lower.

The statistical test performed will be the Signed-ranktest, which will incorporate the “shift” parameter of –0.9 log10 (the minimum clinically significant difference selected for this study). An evaluation of potential harm (increased viral load) will also be performed with the Signed ranktest; this test will examine the null hypothesis of no change in viral load (a shift of 0.0 log10 following investigational product administration) against the one-sided alternative hypothesis that the viral load is increased following investigational product administration. Each efficacy test will be performed at the level $\alpha = 0.05$. Each test for harm will be performed at level $2\alpha = 0.10$, in order to provide additional sensitivity to detect potential harm.

14.5 Secondary and Exploratory Immunologic and Virologic Analyses

14.5.1 Sample Size

The sample size for secondary immunologic and/or virologic analysis will be the 12-36 HIV infected participants in part 2 of the study.

14.5.2 Null Hypothesis

No formal hypothesis on immunologic or virologic responses will be tested, with the exception of the change in HIV viral load described in Section 14.3.

14.5.3 Statistical Power and Analysis

Descriptive statistics (actual values and changes from reference) will be calculated for continuous immunologic and virologic parameters at all time points. Graphical representations of changes in parameters will be made as applicable. Differences between Subgroups and/or Groups at a specific time point will be tested for exploratory purposes by a 2-sample t-test if the data appear to be normally distributed (after transformation if necessary). If not, the non-parametric Wilcoxon rank sum test will be used. If portions of the measurements are censored below the assay quantification limit, the Gehan-Wilcoxon test will be employed. All statistical tests will be two-sided and will be considered statistically significant if $p < 0.05$.

Frequency tabulations will be calculated for discrete (qualitative) immunologic and virologic parameters at all time points. Significant differences between Subgroups and/or Groups will be determined by a 2-sided Fisher’s exact test.

Interim immunologic and virologic analyses of grouped data may be performed without unblinding the study to investigators or participants.

15.0 QUALITY CONTROL AND QUALITY ASSURANCE

To ensure the quality and reliability of the data collected and generated and the ethical conduct of this study, a Study Operations Manual (SOM) will be developed. All

deviations will be reported and investigated. The SOM describes reporting and deviation documentation requirements and procedures.

Regular monitoring will be performed according to ICH-GCP as indicated in Section 17.5.

An independent audit of the study and study sites may be performed by the Sponsor or designee to establish the status of applicable quality systems. Inspection by regulatory authorities may also occur.

By signing the protocol, the Principal Investigators agree to facilitate study related monitoring, audits, IRB/IEC review and regulatory inspection(s) and direct access to source documents. Such information will be treated as strictly confidential and under no circumstances be made publicly available.

16.0 DATA AND BIOLOGICAL MATERIAL

All data and biological material collected through the study shall be managed in accordance with the Clinical Trial Agreement (CTA). Distribution and use of these data will be conducted by agreement of all parties.

The computerized raw data generated will be held by the DCC on behalf of the Sponsor. The study sites will also hold the final data files and tables generated for the purpose of analysis.

17.0 ADMINISTRATIVE STRUCTURE

The Principal Investigator will be responsible for all aspects of the study at the study site.

17.1 Protocol Safety Review Team

A PSRT will be formed to monitor the clinical safety data. During the administration of investigational productphase of the trial, the PSRT will review the clinical safety data on a weekly basis via electronic distribution of reports. An ad hoc PSRT review meeting will occur if any of the members of the PSRT requests a special review to discuss a specific safety issue or as specified in the Study Operations Manual. After the administration of investigational product phase the PSRT will review the clinical safety data at least monthly.

The PSRT will consist of the IAVI Medical Monitor(s), and the PI or designee from each clinical team. The study chair or an IAVI Medical Monitor may be the PSRT chair. *Ex officio* members will include the IAVI Chief Medical Officer and an unblinded IAVI Medical Monitor. Additional PSRT participants may include the following, as needed:

- Co-investigators and trial site senior clinical research nursing staff
- Laboratory directors
- Data management, study statistician and regulatory staff

The PSRT membership and procedures are detailed in the PSRT charter.

17.2 Safety Monitoring Committee (SMC)

The SMC will consist of independent clinicians/scientists/statisticians/ethicists who are not involved in the study. Investigators responsible for the clinical care of participants or representative of the Sponsor may not be a member of the SMC. Details of membership, chair and co-chair and responsibilities are outlined in the SMC charter.

Principal Investigator(s) or designee and/or a Sponsor representative may be asked to join an open session of the SMC meeting to provide information on study conduct, present data or to respond to questions.

Safety data will be reviewed by the SMC at pre-specified time points and at an ad-hoc basis.

17.2.1 Content of Interim Safety Review

The SMC will be asked to review the following blinded data:

- Summary of reactogenicity (i.e., solicited adverse events)
- All adverse events judged by the Principal Investigator or designee to be possibly, probably or definitely related to investigational product
- All laboratory results confirmed on retest and judged by the Principal Investigator or designee to be clinically significant
- All SAEs and pIMDs

An unblinded presentation of all above noted events may also be made available for the SMC for their review if required by any member of the SMC.

17.2.2 SMC Review of Group 1 and 2 data prior to starting Group 3

Following IV infusion of investigational product of the last participant in Groups 1 and/or 2, the Safety Monitoring Committee (SMC) will review safety data through the day 14 post-IV infusion visit for all participants to confirm MTD in each Group, and determine whether, and at what dose level, Groups 3A and 3B can initiate enrollment. See section 5.3 for additional details.

17.3 Criteria for Pausing the Study

Enrollment and administration of investigational product will be stopped and a safety review conducted by the SMC for any of the following criteria:

- One or more participants experience an SAE that is judged possibly, probably or definitely related to investigational product.
- There is a participant death, regardless of relationship to the investigational product.
- Two or more participants experience Grade 3 adverse events in the same category System Organ Class that are considered possibly, probably or definitely related to investigational product or
- Any Grade 4 adverse event that is considered possibly, probably or definitely related to investigational product.

Table 17.3-1: AE notification and safety pause/AE review rules

Event and relationship to study product	Severity	Occurrence	Site PI action	PSRT or SMC action
SAE, possibly, probably or definitely related	Any	Any	Phone, email or fax forms to sponsor within 24 hours	Study pause within 24 hours, refer to SMC for review
SAE, probably not or not related	Death	Any	Phone, email or fax forms to sponsor within 24 hours	Study pause within 24 hours, refer to SMC for review
AE, possibly, probably or definitely related	Grade 4	Any	Phone, email or fax notification to sponsor within 24 hours	Study pause within 24 hours, refer to SMC for review
AE¶, possibly, probably or definitely related	Grade 3*	First	Phone, email or fax notification to sponsor within 24 hours	PSRT review within 2 business days to consider pause
AE¶, possibly, probably or definitely related	Grade 3*	Second‡	Phone, email or fax notification to sponsor within 24 hours	Study pause within 24 hours, refer to SMC for review

¶Does not include the following reactogenicity symptoms (fever, fatigue/malaise, myalgia, arthralgia, chills, headache, nausea, vomiting).

*If no evidence of disease is present other than an abnormal laboratory value, the test must be repeated with a new blood sample at least one time within 72 hours after the investigator becoming aware of the abnormal laboratory value. When signs and symptoms are present, repeat test will not be needed.

‡PSRT will determine whether the reported related AE (Grade 3) is a second occurrence of a previously reported AE (Grade 3).

The Sponsor will request a review by the SMC, (or the SMC chair if other SMC members cannot be convened), to be held within 2 business days of the Sponsor learning of the event. The individual participant(s)/or study may be unblinded at the discretion of the SMC. Following this review, the SMC will make a recommendation regarding the continuation or suspension of the administration of the investigational product or the trial and communicate this decision immediately to the Sponsor. The Sponsor then will inform the Principal Investigators without delay.

Additional *ad hoc* review may be specifically requested by the Sponsor, the Principal Investigator(s) or by the SMC.

17.4 Study Supervision

The SMC, the IAVI Chief Medical Officer (CMO) and the IAVI Medical Monitor(s) have access to progress report(s) of this study. Close cooperation will be necessary to track study progress, respond to queries about proper study implementation and management, address issues in a timely manner, and assure consistent documentation, and share information effectively. Rates of accrual, retention, and other parameters relevant to the site's performance will be regularly and closely monitored by the study team.

17.5 Study Monitoring

On-and/or off-site monitoring will ensure that the study is conducted in compliance with human subjects' protection and other research regulations and guidelines, recorded and reported in accordance with the protocol, is consistent with SOPs, GCP, applicable regulatory requirements and locally accepted practices. The monitor will confirm the quality and accuracy of data at the site by validation of CRFs against the source documents, such as clinical records. The investigators, as well as participants through consenting to the study, agree that the monitor may inspect study facilities and source records (e.g., informed consent forms, clinic and laboratory records, other source documents), as well as observe the performance of study procedures (in accordance with site IRB requirements). Such information will be treated as strictly confidential and will under no circumstances be made publicly available.

The monitoring will adhere to GCP guidelines. The Principal Investigator will permit inspection of the facilities and all study-related documentation by authorized representatives of IAVI, and Government and Regulatory Authorities responsible for this study.

17.6 Investigator's Records

Study records include administrative documentation—e.g., reports and correspondence relating to the study—as well as documentation related to each participant screened and/or enrolled in the study—including informed consent forms, case report forms, and all other source documents. The investigator will maintain and store, in a secure manner, complete, accurate, and current study records for a minimum of 2 years after marketing application approval or the study is discontinued and applicable national and local health authorities are notified. IAVI will notify the Principal Investigator of these events.

18.0 INDEMNITY

The Sponsor and Institution are responsible to have appropriate liability insurance. For research-related injuries and/or medical problems determined to result from receiving the investigational product, treatment including necessary emergency treatment and proper follow-up care will be made available to the participant free of charge at the expense of the Sponsor.

19.0 PUBLICATION

A primary manuscript describing safety, anti-viral effect and immune responses in this trial will be prepared promptly after the data analysis is available.

Authors will be representatives of each trial site, the data management and statistical analysis center, the laboratories, the product developer and the sponsor, participant to the generally accepted criteria of contributions to the design and conduct of the study, the analysis of data and writing of the manuscript. Precedence will be given to authors from the site enrolling the greatest number of participants. Manuscripts will be reviewed by representatives of each participating group as specified in the CTA.

20.0 ETHICAL CONSIDERATIONS

The Principal Investigator will ensure that the study is conducted in compliance with the protocol, SOPs in accordance with guidelines formulated by the ICH for GCP in clinical studies, the ethical principles that have their origins in the Declaration of Helsinki and applicable local standards and regulatory requirements.

APPENDIX A: SCHEDULE OF PROCEDURES – GROUP 1 (A, B, C)

**Study Month**		**0**							**1**		**2**		**3**	**4**	**5**	**6**
**Study Week**		**0**				**1**	**2**	**3**	**4**	**6**	**8**	**10**	**12**	**16**	**20**	**24**
**Study Day**	**Scr**	**0**	**1**	**2**	**3**	**7**	**14**	**21**	**28**	**42**	**56**	**70**	**84**	**112**	**140**	**168/ET^**
**Visit Windows (Days)**	**-56**	**0**	**0**	**0**	**0**	**± 1**	**± 2**	**± 2**	**± 2**	**± 3**	**± 3**	**±3**	**±7**	**±7**	**±7**	**±7**
**INVESTIGATIONAL PRODUCT**																
Investigational Product		**X**														
**CONSENT/ASSESSMENTS/COUNSELLING**																
Informed Consent	**X**															
Assessment of Understanding	**X**															
HIV Risk Assessment	**X**															**X**
HIV Risk Reduction Counselling	**X**	**X**							**X**		**X**		**X**	**X**	**X**	**X**
HIV-test Counselling	**X**	**X**							**X**							**X**
Family Planning Counselling	**X**	**X**														
Social Impact Assessment																**X**
**CLINICAL SAFETY ASSESSMENTS**																
Comprehensive Medical History	**X**															
Interim Medical History		**X**	**X**	**X**	**X**	**X**	**X**	**X**	**X**	**X**	**X**					
Concomitant Medications	**X**	**X**	**X**	**X**	**X**	**X**	**X**	**X**	**X**	**X**	**X**					
General Physical Exam	**X**															**X**
Directed Physical Exam		**X**	**X**	**X**	**X**	**X**	**X**	**X**	**X**	**X**	**X**	**X**	**X**	**X**	**X**	
Weight	**X**	**X**														**X**
Height	**X**															
Vital Signs	**X**	**X***	**X**	**X**	**X**	**X**	**X**	**X**	**X**	**X**	**X**	**X**	**X**	**X**	**X**	**X**
Local & Systemic Reactogenicity		**X***	**X**	**X**	**X**											
Adverse Events		**X**	**X**	**X**	**X**	**X**	**X**	**X**	**X**	**X**	**X**					
Serious Adverse Events and pIMD	**X**	**X**	**X**	**X**	**X**	**X**	**X**	**X**	**X**	**X**	**X**	**X**	**X**	**X**	**X**	**X**

Study Month		0							1		2		3	4	5	6
**Study Week**		**0**				**1**	**2**	**3**	**4**	**6**	**8**	**10**	**12**	**16**	**20**	**24**
**Study Day**	**Scr**	**0**	**1**	**2**	**3**	**7**	**14**	**21**	**28**	**42**	**56**	**70**	**84**	**112**	**140**	**168/ET^**
**Visit Windows (Days)**	**-56**	**0**	**0**	**0**	**0**	**± 1**	**± 2**	**± 2**	**± 2**	**± 3**	**± 3**	**±3**	**±7**	**±7**	**±7**	**±7**
**CLINICAL LABORATORY TESTS**																
Hematology and Coagulation	X	X#	X		X	X	X		X		X		X	X	X	X
Clinical Chemistry	X	X#	X		X	X	X		X		X		X	X	X	X
Urine Dipstick	X	X#	X		X	X	X		X		X		X	X	X	X
Urine Pregnancy test	X	X#							X		X		X			X
Active Syphilis	X															
Chlamydia, Gonorrhea	X															
Hepatitis B	X															
Hepatitis C	X															
HIV screen (4th generation Ag/Ab test)	X															
Blinded HIV diagnostic testing		X#							X							X
**RESEARCH LABORATORY TESTS**																
Anti PGDM1400 and anti-PGT121 Antibodies (ADA)		X#							X		X		X			X
Humoral Assays**		X#			X	X	X		X		X		X			X
Cellular Assays**		X#					X		X		X		X			X
HLA typing		X#														
**PHARMACOKINETICS PGDM1400 ± PGT121 ELISA**		X#	X	X	X	X	X	X	X	X	X	X	X	X	X	X
**MUCOSAL SAMPLING**		X~	X			X	X									
**PLASMA/SERUM STORAGE**		X	X	X	X	X	X	X	X		X		X			X
**PBMC STORAGE**		X									X		X			X

Day 0 baseline sample collections must be done before infusion of investigational product. Additional day 0 pharmacokinetics sample collection will be done as outlined in Protocol section 9.4.2.
^ Early Termination (ET): Procedures to be performed at ET are the same as last visit procedures
* At baseline, approximately every 30 minutes for the first 4 hours after IP administration, and then every hour until 6 hours after IV infusion. Local and systemic reactogenicity will be assessed by clinic staff at visits on study days 1, 2 and 3. Local and Systemic reactogenicity will also be assessed by the participant using the Memory Aid on study days 1, 2, and 3.
** See Laboratory Analytical Plan for details
~ Cervico-vaginal and/or rectal mucosal sampling (optional) on Day 0 must be done prior to IV infusion of IP.

APPENDIX B: SCHEDULE OF PROCEDURES – AND GROUP 2 (A, B, C)

Study Month		0							1		2		3	4	5	6
**Study Week**		**0**				**1**	**2**	**3**	**4**	**6**	**8**	**10**	**12**	**16**	**20**	**24**
**Study Day**	**Scr**	**0**	**1**	**2**	**3**	**7**	**14**	**21**	**28**	**42**	**56**	**70**	**84**	**112**	**140**	**168/ET^**
**Visit Windows (Days)**	**-56**	**0**	**0**	**0**	**0**	**± 1**	**± 2**	**± 2**	**± 2**	**± 3**	**± 3**	**±3**	**±7**	**±7**	**±7**	**±7**
**INVESTIGATIONAL PRODUCT**																
Investigational Product		X														
**CONSENT/ASSESSMENTS/COUNSELLING**																
Informed Consent	X															
Assessment of Understanding	X															
HIV Risk Assessment	X															X
HIV Risk Reduction Counselling	X	X							X		X		X	X	X	X
HIV-test Counselling	X	X							X							X
Family Planning Counselling	X	X														
Social Impact Assessment																X
**CLINICAL SAFETY ASSESSMENTS**																
Comprehensive Medical History	X															
Interim Medical History		X	X	X	X	X	X	X	X	X	X					
Concomitant Medications	X	X	X	X	X	X	X	X	X	X	X					
General Physical Exam	X															X
Directed Physical Exam		X	X	X	X	X	X	X	X	X	X	X	X	X	X	
Weight	X	X														X
Height	X															
Vital Signs	X	X*	X	X	X	X	X	X	X	X	X	X	X	X	X	X
Local & Systemic Reactogenicity		X*	X	X	X											
Adverse Events		X	X	X	X	X	X	X	X	X	X					
Serious Adverse Events and pIMD	X	X	X	X	X	X	X	X	X	X	X	X	X	X	X	X
**CLINICAL LABORATORY TESTS**																

Study Month		0							1		2		3	4	5	6
**Study Week**		**0**				**1**	**2**	**3**	**4**	**6**	**8**	**10**	**12**	**16**	**20**	**24**
**Study Day**	**Scr**	**0**	**1**	**2**	**3**	**7**	**14**	**21**	**28**	**42**	**56**	**70**	**84**	**112**	**140**	**168/ET^**
**Visit Windows (Days)**	**-56**	**0**	**0**	**0**	**0**	**± 1**	**± 2**	**± 2**	**± 2**	**± 3**	**± 3**	**±3**	**±7**	**±7**	**±7**	**±7**
Hematology and Coagulation	X	X#	X		X	X	X		X		X		X	X	X	X
Clinical Chemistry	X	X#	X		X	X	X		X		X		X	X	X	X
Urine Dipstick	X	X#	X		X	X	X		X		X		X	X	X	X
Urine Pregnancy test	X	X#							X		X		X			X
Active Syphilis	X															
Chlamydia, Gonorrhea	X															
Hepatitis B	X															
Hepatitis C	X															
HIV screen (4th generation Ag/Ab test)	X															
Blinded HIV diagnostic testing		X#							X							X
**RESEARCH LABORATORY TESTS**																
Anti PGDM1400 and anti-PGT121 Antibodies (ADA)		X#							X		X		X			X
Humoral Assays**		X#			X	X	X		X		X		X			X
Cellular Assays**		X#					X		X		X		X			X
HLA typing		X#														
**PHARMACOKINETICS PGDM1400 ± PGT121 ELISA**		X#	X	X	X	X	X	X	X	X	X	X	X	X	X	X
**MUCOSAL SAMPLING**		X~	X			X	X									
**PLASMA/SERUM STORAGE**		X	X	X	X	X	X	X	X		X		X			X
**PBMCs STORAGE**		X									X		X			X

Day 0 baseline sample collections must be done before infusion of investigational product. Additional day 0 pharmacokinetics sample collection will be done as outlined in Protocol section 9.4.2.
^ Early Termination (ET): Procedures to be performed at ET are the same as last visit procedures
* At baseline, approximately every 30 minutes for the first 4 hours after IP administration and then every hour until 6 hours after IV infusion. Local and systemic reactogenicity will be assessed by clinic staff at visits on study days 1, 2 and 3. Local and Systemic reactogenicity will also be assessed by the participant using the Memory Aid on study days 1, 2, and 3.
** See Laboratory Analytical Plan for details
~ Cervico-vaginal and/or rectal mucosal sampling (optional) on Day 0 must be done prior to IV infusion of IP.

APPENDIX C: SCHEDULE OF PROCEDURES – GROUP 3 (A, B)

**Study Month**		**0**								**1**		**2**		**3**	**4**	**5**	**6**
**Study Week**		**0**				**1**		**2**	**3**	**4**	**6**	**8**	**10**	**12**	**16**	**20**	**24**
**Study Day**	**Scr**	**0**	**1**	**2**	**3**	**7**	**10**	**14**	**21**	**28**	**42**	**56**	**70**	**84**	**112**	**140**	**168/ET**
**Visit Windows (Days)**	**-42**	**0**	**0**	**0**	**0**	**± 1**	**0**	**± 2**	**± 2**	**± 2**	**± 3**	**± 3**	**±3**	**±7**	**±7**	**±7**	**±7**
**INVESTIGATIONAL PRODUCT**																	
Investigational Product		**X**															
**CONSENT/ASSESSMENTS/COUNSELLING**																	
Informed Consent	**X**																
Assessment of Understanding	**X**																
HIV Risk Reduction Counselling	**X**	**X**								**X**		**X**		**X**	**X**	**X**	**X**
ART counselling	**X**	**X**										**X**					**X**
Family Planning Counselling	**X**	**X**															
Social Impact Assessment																	**X**
**CLINICAL SAFETY ASSESSMENTS**																	
Comprehensive Medical History	**X**																
Interim Medical History		**X**	**X**	**X**	**X**	**X**	**X**	**X**	**X**	**X**	**X**	**X**					
Concomitant Medications	**X**	**X**	**X**	**X**	**X**	**X**	**X**	**X**	**X**	**X**	**X**	**X**					
General Physical Exam	**X**																**X**
Directed Physical Exam		**X**	**X**	**X**	**X**	**X**	**X**	**X**	**X**	**X**	**X**	**X**	**X**	**X**	**X**	**X**	
Weight	**X**	**X**															**X**
Height	**X**																
Vital Signs	**X**	**X***	**X**	**X**	**X**	**X**	**X**	**X**	**X**	**X**	**X**	**X**	**X**	**X**	**X**	**X**	**X**
Local & Systemic Reactogenicity		**X***	**X**	**X**	**X**												
Adverse Events		**X**	**X**	**X**	**X**	**X**	**X**	**X**	**X**	**X**	**X**	**X**					
Serious Adverse Events and pIMD	**X**	**X**	**X**	**X**	**X**	**X**	**X**	**X**	**X**	**X**	**X**	**X**	**X**	**X**	**X**	**X**	**X**
**CLINICAL LABORATORY TESTS**																	
Hematology and Coagulation	**X**	**X#**	**X**		**X**	**X**		**X**		**X**		**X**		**X**	**X**	**X**	**X**
CD4	**X**	**X#**				**X**		**X**		**X**		**X**					**X**

**Study Month**		**0**								**1**		**2**		**3**	**4**	**5**	**6**
**Study Week**		**0**				**1**		**2**	**3**	**4**	**6**	**8**	**10**	**12**	**16**	**20**	**24**
**Study Day**	**Scr**	**0**	**1**	**2**	**3**	**7**	**10**	**14**	**21**	**28**	**42**	**56**	**70**	**84**	**112**	**140**	**168/ET**
**Visit Windows (Days)**	**-42**	**0**	**0**	**0**	**0**	**± 1**	**0**	**± 2**	**± 2**	**± 2**	**± 3**	**± 3**	**±3**	**±7**	**±7**	**±7**	**±7**
Clinical Chemistry	**X**	**X#**	**X**		**X**	**X**		**X**		**X**		**X**		**X**	**X**	**X**	**X**
Urine Dipstick	**X**	**X#**	**X**		**X**	**X**		**X**		**X**		**X**		**X**	**X**	**X**	**X**
Urine Pregnancy test	**X**	**X#**								**X**		**X**		**X**			**X**
Active Syphilis	**X**																
Chlamydia, Gonorrhea	**X**																
Hepatitis B	**X**																
Hepatitis C	**X**																
HIV 4th generation Ag/Ab test***	**X**																
HIV Viral Load	**X**	**X#**	**X**	**X**	**X**	**X**	**X**	**X**	**X**	**X**	**X**	**X**	**X**	**X**	**X**	**X**	**X**
**RESEARCH LABORATORY TESTS**	-	-	-	-	-	-	-	-	-	-	-	-	-	-	-	-	-
Anti PGDM1400 and anti-PGT121 Antibodies (ADA)		**X#**								**X**		**X**		**X**			**X**
HIV phenotypic testing for PGDM1400 ± PGT121 susceptibility	**X**									**X**							**X**
HIV SGA sequencing	**X**									**X**							**X**
HIV genotypic testing for ART resistance	**X**									**X**				**X**			**X**
HIV reservoir size assessment	**X**							**X**						**X**			
Humoral Assays**		**X#**			**X**	**X**		**X**		**X**		**X**		**X**			**X**
Cellular Assays**		**X#**						**X**		**X**		**X**		**X**			**X**
HLA typing		**X#**															
**PHARMACOKINETICS PGDM1400 ± PGT121 ELISA**	**X**	**X#**	**X**	**X**	**X**	**X**		**X**	**X**	**X**	**X**	**X**	**X**	**X**	**X**	**X**	**X**
**MUCOSAL SAMPLING**		**X~**	**X**			**X**		**X**									
**PLASMA/SERUM STORAGE**	**X**	**X**	**X**	**X**	**X**	**X**	**X**	**X**	**X**	**X**		**X**		**X**			**X**
**PBMCs STORAGE**		**X**										**X**		**X**			**X**

Day 0 baseline sample collections must be done before infusion of investigational product. Additional day 0 pharmacokinetics sample collection will be done as outlined in Protocol section 9.4.2.

^ Early Termination (ET): Procedures to be performed at ET are the same as last visit procedures
* At baseline, approximately every 30 minutes for the first 4 hours after IP administration and then every hour until 6 hours after IV infusion. Local and systemic reactogenicity will be assessed by clinic staff at visits on study days 1, 2 and 3. Local and Systemic reactogenicity will also be assessed by the participant using the Memory Aid on study days 1, 2, and 3.
** See Laboratory Analytical Plan for details
*** Confirmed HIV-1 infection (HIV Ab+ or HIV RNA+) by documentation in the medical records or in-clinic HIV testing;
~ Cervico-vaginal and/or rectal mucosal sampling (optional) on Day 0 must be done prior to IV infusion of IP.

APPENDIX D: LOW RISK CRITERIA

Low risk will be defined as:

1. **SEXUAL BEHAVIORS**

In the **last 12 months** did not:

- Have oral, vaginal or anal intercourse with an HIV-infected partner, or partner who uses injection drugs.
- Gave or receive money, drugs, gifts, or services in exchange for oral, vaginal or anal sex

  AND

  In the **last 6 months** has abstained from penile/anal or penile/vaginal intercourse

  OR

  In the **last 6 months**:

- Had 4 or fewer partners of the opposite birth sex for vaginal and/or anal intercourse, OR

Is MSM (person born male with partner(s) born male) who, in the **last 12 months**:

- Had 2 or fewer MSM partners for anal intercourse and had no unprotected anal sex with MSM, OR
- Had unprotected anal intercourse with only 1 MSM partner, within a monogamous relationship lasting at least 12 months (during which neither partner had any other partners). If the monogamous relationship ended, the participant may then have had protected anal intercourse with 1 other MSM partner (total 2 or fewer partners in the last 12 months).

Is a transgender person, regardless of the point on the transition spectrum, having sex with men (born male) and/or other transgender persons, who in the last 12 months:

- Had 2 or fewer partners for anal or vaginal intercourse, and had no unprotected anal or vaginal sex, OR
- Had unprotected anal or vaginal intercourse sex with 1 partner only within a monogamous relationship lasting at least 12 months (during which neither partner had any other partners). If the monogamous relationship ended, may then have had protected anal or vaginal sex with one other partner (total 2 or fewer partners in the last 12 months).

  AND

  Uses or intends to use condoms in situations which may include penile/anal or penile/vaginal intercourse with new partners of unknown HIV status, occasional partners, partners outside a primary relationship, and/or partners known to have other partners.

2. **NON-SEXUAL BEHAVIORS**

   In the **last 12 months** did not:

- Inject drugs or other substances without a prescription

- Use cocaine, methamphetamine, or excessive alcohol, which in the investigator’s judgement, rendered the participant at greater than low risk for acquiring HIV infection

  The investigator’s judgement should consider local epidemiologic information about HIV prevalence in the area and community networks.

  A participant is NOT appropriate for inclusion if he/she:

  Acquired an STI (i.e., new infection) in the last 12 months:

- Syphilis
- Gonorrhea
- Non-gonococcal urethritis
- HSV-2
- Chlamydia
- Pelvic inflammatory disease (PID)
- Trichomonas
- Mucopurulent cervicitis
- Epididymitis
- Proctitis
- Lymphogranuloma venereum
- Chancroid
- Hepatitis B

APPENDIX E REFERENCES

Andrade, A., S. L. Rosenkranz, A. R. Cillo, D. Lu, E. S. Daar, J. M. Jacobson, M. Lederman, E. P. Acosta, T. Campbell, J. Feinberg, C. Flexner, J. W. Mellors, D. R. Kuritzkes and A. C. T. G. A. Team (2013). "Three distinct phases of HIV-1 RNA decay in treatment-naive patients receiving raltegravir-based antiretroviral therapy: ACTG A5248." J Infect Dis **208**(6): 884-891.

Bar, K. J., M. C. Sneller, L. J. Harrison, J. S. Justement, E. T. Overton, M. E. Petrone, D. B. Salantes, C. A. Seamon, B. Scheinfeld, R. W. Kwan, G. H. Learn, M. A. Proschan, E. F. Kreider, J. Blazkova, M. Bardsley, E. W. Refsland, M. Messer, K. E. Clarridge, N. B. Tustin, P. J. Madden, K. Oden, S. J. O'Dell, B. Jarocki, A. R. Shiakolas, R. L. Tressler, N. A. Doria-Rose, R. T. Bailer, J. E. Ledgerwood, E. V. Capparelli, R. M. Lynch, B. S. Graham, S. Moir, R. A. Koup, J. R. Mascola, J. A. Hoxie, A. S. Fauci, P. Tebas and T. W. Chun (2016). "Effect of HIV Antibody VRC01 on Viral Rebound after Treatment Interruption." N Engl J Med **375**(21): 2037-2050.

Barouch, D. H. and S. G. Deeks (2014). "Immunologic strategies for HIV-1 remission and eradication." Science **345**(6193): 169-174.

Barouch, D. H., J. B. Whitney, B. Moldt, F. Klein, T. Y. Oliveira, J. Liu, K. E. Stephenson, H. W. Chang, K. Shekhar, S. Gupta, J. P. Nkolola, M. S. Seaman, K. M. Smith, E. N. Borducchi, C. Cabral, J. Y. Smith, S. Blackmore, S. Sanisetty, J. R. Perry, M. Beck, M. G. Lewis, W. Rinaldi, A. K. Chakraborty, P. Poignard, M. C. Nussenzweig and D. R. Burton (2013). "Therapeutic efficacy of potent neutralizing HIV-1-specific monoclonal antibodies in SHIV-infected rhesus monkeys." Nature **503**(7475): 224-228.

Burton, D. R. and J. R. Mascola (2015). "Antibody responses to envelope glycoproteins in HIV-1 infection." Nat Immunol **16**(6): 571-576.

Caskey, M., F. Klein, J. C. Lorenzi, M. S. Seaman, A. P. West, Jr., N. Buckley, G. Kremer, L. Nogueira, M. Braunschweig, J. F. Scheid, J. A. Horwitz, I. Shimeliovich, S. Ben-Avraham, M. Witmer-Pack, M. Platten, C. Lehmann, L. A. Burke, T. Hawthorne, R. J. Gorelick, B. D. Walker, T. Keler, R. M. Gulick, G. Fatkenheuer, S. J. Schlesinger and M. C. Nussenzweig (2015). "Viraemia suppressed in HIV-1-infected humans by broadly neutralizing antibody 3BNC117." Nature **522**(7557): 487-491.

Caskey, M., T. Schoofs, H. Gruell, A. Settler, T. Karagounis, E. F. Kreider, B. Murrell, N. Pfeifer, L. Nogueira, T. Y. Oliveira, G. H. Learn, Y. Z. Cohen, C. Lehmann, D. Gillor, I. Shimeliovich, C. Unson-O'Brien, D. Weiland, A. Robles, T. Kummerle, C. Wyen, R. Levin, M. Witmer-Pack, K. Eren, C. Ignacio, S. Kiss, A. P. West, Jr., H. Mouquet, B. S. Zingman, R. M. Gulick, T. Keler, P. J. Bjorkman, M. S. Seaman, B. H. Hahn, G. Fatkenheuer, S. J. Schlesinger, M. C. Nussenzweig and F. Klein (2017). "Antibody 10-1074 suppresses viremia in HIV-1-infected individuals." Nat Med.

CDC (2014). "CDC. Vital Signs: HIV Diagnosis, Care, and Treatment Among Persons Living with HIV- United States 2011." MMWR **4**(63): 1-6.

Haynes, B. F. and M. J. McElrath (2013). "Progress in HIV-1 vaccine development." Curr Opin HIV AIDS **8**(4): 326-332.

Hessell, A. J., P. Poignard, M. Hunter, L. Hangartner, D. M. Tehrani, W. K. Bleeker, P. W. Parren, P. A. Marx and D. R. Burton (2009). "Effective, low-titer antibody protection against low-dose repeated mucosal SHIV challenge in macaques." Nat Med **15**(8): 951-954.

Hessell, A. J., E. G. Rakasz, P. Poignard, L. Hangartner, G. Landucci, D. N. Forthal, W. C. Koff, D. I. Watkins and D. R. Burton (2009). "Broadly neutralizing human anti-HIV antibody 2G12 is effective in protection against mucosal SHIV challenge even at low serum neutralizing titers." PLoS Pathog **5**(5): e1000433.

Jardine, J., J. P. Julien, S. Menis, T. Ota, O. Kalyuzhniy, A. McGuire, D. Sok, P. S. Huang, S. MacPherson, M. Jones, T. Nieusma, J. Mathison, D. Baker, A. B. Ward, D. R. Burton, L. Stamatatos, D. Nemazee, I. A. Wilson and W. R. Schief (2013). "Rational HIV immunogen design to target specific germline B cell receptors." Science **340**(6133): 711-716.

Ledgerwood, J. E., E. E. Coates, G. Yamshchikov, J. G. Saunders, L. Holman, M. E. Enama, A. DeZure, R. M. Lynch, I. Gordon, S. Plummer, C. S. Hendel, A. Pegu, M. Conan-Cibotti, S. Sitar, R. T. Bailer, S. Narpala, A. McDermott, M. Louder, S. O'Dell, S. Mohan, J. P. Pandey, R. M. Schwartz, Z. Hu, R. A. Koup, E. Capparelli, J. R. Mascola, B. S. Graham and V. R. C. S. Team (2015). "Safety, pharmacokinetics and neutralization of the broadly neutralizing HIV-1 human monoclonal antibody VRC01 in healthy adults." Clin Exp Immunol.

Lynch, R. M., E. Boritz, E. E. Coates, A. DeZure, P. Madden, P. Costner, M. E. Enama, S. Plummer, L. Holman, C. S. Hendel, I. Gordon, J. Casazza, M. Conan-Cibotti, S. A. Migueles, R. Tressler, R. T. Bailer, A. McDermott, S. Narpala, S. O'Dell, G. Wolf, J. D. Lifson, B. A. Freemire, R. J. Gorelick, J. P. Pandey, S. Mohan, N. Chomont, R. Fromentin, T. W. Chun, A. S. Fauci, R. M. Schwartz, R. A. Koup, D. C. Douek, Z. Hu, E. Capparelli, B. S. Graham, J. R. Mascola, J. E. Ledgerwood and V. R. C. S. Team (2015). "Virologic effects of broadly neutralizing antibody VRC01 administration during chronic HIV-1 infection." Sci Transl Med **7**(319): 319ra206.

Moldt, B., E. G. Rakasz, N. Schultz, P. Y. Chan-Hui, K. Swiderek, K. L. Weisgrau, S. M. Piaskowski, Z. Bergman, D. I. Watkins, P. Poignard and D. R. Burton (2012). "Highly potent HIV-specific antibody neutralization in vitro translates into effective protection against mucosal SHIV challenge in vivo." Proc Natl Acad Sci U S A **109**(46): 18921-18925.

Scheid, J. F., J. A. Horwitz, Y. Bar-On, E. F. Kreider, C. L. Lu, J. C. Lorenzi, A. Feldmann, M. Braunschweig, L. Nogueira, T. Oliveira, I. Shimeliovich, R. Patel, L. Burke, Y. Z. Cohen, S. Hadrigan, A. Settler, M. Witmer-Pack, A. P. West, Jr., B. Juelg, T. Keler, T. Hawthorne, B. Zingman, R. M. Gulick, N. Pfeifer, G. H. Learn, M. S. Seaman, P. J. Bjorkman, F. Klein, S. J. Schlesinger, B. D. Walker, B. H. Hahn and M. C. Nussenzweig (2016). "HIV-1 antibody 3BNC117 suppresses viral rebound in humans during treatment interruption." Nature **535**(7613): 556-560.

Scheid, J. F., H. Mouquet, B. Ueberheide, R. Diskin, F. Klein, T. Y. Oliveira, J. Pietzsch, D. Fenyo, A. Abadir, K. Velinzon, A. Hurley, S. Myung, F. Boulad, P. Poignard, D. R. Burton, F. Pereyra, D. D. Ho, B. D. Walker, M. S. Seaman, P. J. Bjorkman, B. T. Chait and M. C. Nussenzweig (2011). "Sequence and structural convergence of broad and potent HIV antibodies that mimic CD4 binding." Science **333**(6049): 1633-1637.

Schoofs, T., F. Klein, M. Braunschweig, E. F. Kreider, A. Feldmann, L. Nogueira, T. Oliveira, J. C. Lorenzi, E. H. Parrish, G. H. Learn, A. P. West, Jr., P. J. Bjorkman, S. J. Schlesinger, M. S. Seaman, J. Czartoski, M. J. McElrath, N. Pfeifer, B. H. Hahn, M. Caskey and M. C. Nussenzweig (2016). "HIV-1 therapy with monoclonal antibody 3BNC117 elicits host immune responses against HIV-1." Science **352**(6288): 997-1001.

Simek, M. D., W. Rida, F. H. Priddy, P. Pung, E. Carrow, D. S. Laufer, J. K. Lehrman, M. Boaz, T. Tarragona-Fiol, G. Miiro, J. Birungi, A. Pozniak, D. A. McPhee, O. Manigart, E. Karita, A. Inwoley, W. Jaoko, J. Dehovitz, L. G. Bekker, P. Pitisuttithum, R. Paris, L. M. Walker, P. Poignard, T. Wrin, P. E. Fast, D. R. Burton and W. C. Koff (2009). "Human immunodeficiency virus type 1 elite neutralizers: individuals with broad and potent neutralizing activity identified by using a high-throughput neutralization assay together with an analytical selection algorithm." J Virol **83**(14): 7337-7348.

Sok, D., K. J. Doores, B. Briney, K. M. Le, K. L. Saye-Francisco, A. Ramos, D. W. Kulp, J. P. Julien, S. Menis, L. Wickramasinghe, M. S. Seaman, W. R. Schief, I. A. Wilson, P. Poignard and D. R. Burton (2014). "Promiscuous glycan site recognition by antibodies to the high-mannose patch of gp120 broadens neutralization of HIV." Sci Transl Med **6**(236): 236ra263.

Sok, D., M. J. van Gils, M. Pauthner, J. P. Julien, K. L. Saye-Francisco, J. Hsueh, B. Briney, J. H. Lee, K. M. Le, P. S. Lee, Y. Hua, M. S. Seaman, J. P. Moore, A. B. Ward, I. A. Wilson, R. W. Sanders and D. R. Burton (2014). "Recombinant HIV envelope trimer selects for quaternary-dependent antibodies targeting the trimer apex." Proc Natl Acad Sci U S A **111**(49): 17624-17629.

UNAIDS (2016). "UNAIDS Fact Sheet November 2016."

Walker LM, B. D. (2010). "Rational antibody-based HIV-1 vaccine design: current approaches and future directions. ." Curr Opin Immunol. **22**(3): 358-366.

Walker, L. M., M. Huber, K. J. Doores, E. Falkowska, R. Pejchal, J. P. Julien, S. K. Wang, A. Ramos, P. Y. Chan-Hui, M. Moyle, J. L. Mitcham, P. W. Hammond, O. A. Olsen, P. Phung, S. Fling, C. H. Wong, S. Phogat, T. Wrin, M. D. Simek, G. P. I. Protocol, W. C. Koff, I. A. Wilson, D. R. Burton and P. Poignard (2011). "Broad neutralization coverage of HIV by multiple highly potent antibodies." Nature **477**(7365): 466-470.

Walker, L. M., S. K. Phogat, P. Y. Chan-Hui, D. Wagner, P. Phung, J. L. Goss, T. Wrin, M. D. Simek, S. Fling, J. L. Mitcham, J. K. Lehrman, F. H. Priddy, O. A. Olsen, S. M. Frey, P. W. Hammond, G. P. I. Protocol, S. Kaminsky, T. Zamb, M. Moyle, W. C. Koff, P. Poignard and D. R. Burton (2009). "Broad and potent neutralizing antibodies from an African donor reveal a new HIV-1 vaccine target." Science **326**(5950): 285-289.

APPENDIX F CTCAE TABLE

CTCAE4.03 Relevant For T002
Common Terminology Criteria for Adverse Events (CTCAE)
Version 4.0 Published: May 28, 2009 (v4.03: June 14, 2010)

U.S. DEPARTMENT OF HEALTH AND HUMAN SERVICES National Institutes of Health National Cancer Institute

Quick Reference

The NCI Common Terminology Criteria for Adverse Events is a descriptive terminology which can be utilized for Adverse Event (AE) reporting. A grading (severity) scale is provided for each AE term.

Components and Organization

SOC

System Organ Class, the highest level of the MedDRA hierarchy, is identified by anatomical or physiological system, etiology, or purpose (e.g., SOC Investigations for laboratory test results). CTCAE terms are grouped by MedDRA Primary SOCs. Within each SOC, AEs are listed and accompanied by descriptions of severity (Grade).

CTCAE Terms

An Adverse Event (AE) is any unfavorable and unintended sign (including an abnormal laboratory finding), symptom, or disease temporally associated with the use of a medical treatment or procedure that may or may not be considered related to the medical treatment or procedure. An AE is a term that is a unique representation of a specific event used for medical documentation and scientific analyses. Each CTCAE v4.0 term is a MedDRA LLT (Lowest Level Term).

Definitions

A brief definition is provided to clarify the meaning of each AE term.

Grades

Grade refers to the severity of the AE. The CTCAE displays Grades 1 through 5 with unique clinical descriptions of severity for each AE based on this general guideline:

Grade 1	Mild; asymptomatic or mild symptoms; clinical or diagnostic observations only; intervention not indicated.
Grade 2	Moderate; minimal, local or noninvasive intervention indicated; limiting age-appropriate instrumental ADL*.
Grade 3	Severe or medically significant but not immediately life-threatening; hospitalization or prolongation of hospitalization indicated; disabling; limiting self care ADL**.

Grade 4	Life-threatening consequences; urgent intervention indicated.
Grade 5	Death related to AE.

A Semi-colon indicates ‘or’ within the description of the grade.

A single dash (-) indicates a grade is not available.

Not all Grades are appropriate for all AEs. Therefore, some AEs are listed with fewer than five options for Grade selection.

Grade 5

Grade 5 (Death) is not appropriate for some AEs and therefore is not an option.

Activities of Daily Living (ADL)

*Instrumental ADL refer to preparing meals, shopping for groceries or clothes, using the telephone, managing money, etc.

**Self care ADL refer to bathing, dressing and undressing, feeding self, using the toilet, taking medications, and not bedridden.

† CTCAE v4.0 incorporates certain elements of the MedDRA terminology. For further details on MedDRA refer to the MedDRA MSSO Web site (http://www.meddramsso.com).

MedDRA v12.0 Code	CTCAE v4.0 SOC	CTCAE v4.0 Term	Grade 1	Grade 2	Grade 3	Grade 4	Grade 5	CTCAE v4.0 AE Term Definition
10001718	Immune system disorders	Allergic reaction	Transient flushing or rash, drug fever <38 degrees C (<100.4 degrees F); intervention not indicated	Intervention or infusion interruption indicated; responds promptly to symptomatic treatment (e.g., antihistamines, NSAIDS, narcotics); prophylactic medications indicated for <=24 hrs	Prolonged (e.g., not rapidly responsive to symptomatic medication and/or brief interruption of infusion); recurrence of symptoms following initial improvement; hospitalization indicated for clinical sequelae (e.g., renal impairment, pulmonary infiltrates)	Life-threatening consequences; urgent intervention indicated	Death	A disorder characterized by an adverse local or general response from exposure to an allergen.
10002218	Immune system disorders	Anaphylaxis	-	-	Symptomatic bronchospasm, with or without urticaria; parenteral intervention indicated; allergy-related edema/angioedema; hypotension	Life-threatening consequences; urgent intervention indicated	Death	A disorder characterized by an acute inflammatory reaction resulting from the release of histamine and histamine-like substances from mast cells, causing a hypersensitivity immune response. Clinically, it presents with breathing difficulty, dizziness, hypotension, cyanosis and loss of consciousness and may lead to death.

MedDRA v12.0 Code	CTCAE v4.0 SOC	CTCAE v4.0 Term	Grade 1	Grade 2	Grade 3	Grade 4	Grade 5	CTCAE v4.0 AE Term Definition
10003239	Musculoskeletal and connective tissue disorders	Arthralgia	Mild pain	Moderate pain; limiting instrumental ADL	Severe pain; limiting self care ADL	-	-	A disorder characterized by a sensation of marked discomfort in a joint.
10008531	General disorders and administration site conditions	Chills	Mild sensation of cold; shivering; chattering of teeth	Moderate tremor of the entire body; narcotics indicated	Severe or prolonged, not responsive to narcotics	-	-	A disorder characterized by a sensation of cold that often marks a physiologic response to sweating after a fever.
10052015	Immune system disorders	Cytokine release syndrome	Mild reaction; infusion interruption not indicated; intervention not indicated	Therapy or infusion interruption indicated but responds promptly to symptomatic treatment (e.g., antihistamines, NSAIDS, narcotics, IV fluids); prophylactic medications indicated for <=24 hrs	Prolonged (e.g., not rapidly responsive to symptomatic medication and/or brief interruption of infusion); recurrence of symptoms following initial improvement; hospitalization indicated for clinical sequelae (e.g., renal impairment, pulmonary infiltrates)	Life-threatening consequences; pressor or ventilatory support indicated	Death	A disorder characterized by nausea, headache, tachycardia, hypotension, rash, and shortness of breath; it is caused by the release of cytokines from the cells.
10013573	Nervous system disorders	Dizziness	Mild unsteadiness or sensation of movement	Moderate unsteadiness or sensation of movement; limiting instrumental ADL	Severe unsteadiness or sensation of movement; limiting self care ADL	-	-	A disorder characterized by a disturbing sensation of lightheadedness, unsteadiness, giddiness, spinning or rocking.
10013963	Respiratory, thoracic and mediastinal disorders	Dyspnea	Shortness of breath with moderate exertion	Shortness of breath with minimal exertion; limiting instrumental ADL	Shortness of breath at rest; limiting self care ADL	Life-threatening consequences; urgent intervention indicated	Death	A disorder characterized by an uncomfortable sensation of difficulty breathing.

MedDRA v12.0 Code	CTCAE v4.0 SOC	CTCAE v4.0 Term	Grade 1	Grade 2	Grade 3	Grade 4	Grade 5	CTCAE v4.0 AE Term Definition
10015218	Skin and subcutaneous tissue disorders	Erythema multiforme	Target lesions covering <10% BSA and not associated with skin tenderness	Target lesions covering 10 - 30% BSA and associated with skin tenderness	Target lesions covering >30% BSA and associated with oral or genital erosions	Target lesions covering >30% BSA; associated with fluid or electrolyte abnormalities; ICU care or burn unit indicated	Death	A disorder characterized by target lesions (a pink-red ring around a pale center).
10016558	General disorders and administration site conditions	Fever	38.0 - 39.0 degrees C (100.4 - 102.2 degrees F)	>39.0 - 40.0 degrees C (102.3 - 104.0 degrees F)	>40.0 degrees C (>104.0 degrees F) for <=24 hrs	>40.0 degrees C (>104.0 degrees F) for >24 hrs	Death	A disorder characterized by elevation of the body's temperature above the upper limit of normal.
10016825	Vascular disorders	Flushing	Asymptomatic; clinical or diagnostic observations only; intervention not indicated	Moderate symptoms; medical intervention indicated; limiting instrumental ADL	Symptomatic, associated with hypotension and/or tachycardia; limiting self care ADL	-	-	A disorder characterized by episodic reddening of the face.
10019211	Nervous system disorders	Headache	Mild pain	Moderate pain; limiting instrumental ADL	Severe pain; limiting self care ADL	-	-	A disorder characterized by a sensation of marked discomfort in various parts of the head, not confined to the area of distribution of any nerve.

MedDRA v12.0 Code	CTCAE v4.0 SOC	CTCAE v4.0 Term	Grade 1	Grade 2	Grade 3	Grade 4	Grade 5	CTCAE v4.0 AE Term Definition
10020772	Vascular disorders	Hypertension	Prehypertension (systolic BP 120 - 139 mm Hg or diastolic BP 80 - 89 mm Hg)	Stage 1 hypertension (systolic BP 140 - 159 mm Hg or diastolic BP 90 - 99 mm Hg); medical intervention indicated; recurrent or persistent (>=24 hrs); symptomatic increase by >20 mm Hg (diastolic) or to >140/90 mm Hg if previously WNL; monotherapy indicated	Stage 2 hypertension (systolic BP >=160 mm Hg or diastolic BP >=100 mm Hg); medical intervention indicated; more than one drug or more intensive therapy than previously used indicated	Life-threatening consequences (e.g., malignant hypertension, transient or permanent neurologic deficit, hypertensive crisis); urgent intervention indicated	Death	A disorder characterized by a pathological increase in blood pressure; a repeatedly elevation in the blood pressure exceeding 140 over 90 mm Hg.
10021097	Vascular disorders	Hypotension	Asymptomatic, intervention not indicated	Non-urgent medical intervention indicated	Medical intervention or hospitalization indicated	Life-threatening and urgent intervention indicated	Death	A disorder characterized by a blood pressure that is below the normal expected for an individual in a given environment.
10051792	General disorders and administration site conditions	Infusion related reaction	Mild transient reaction; infusion interruption not indicated; intervention not indicated	Therapy or infusion interruption indicated but responds promptly to symptomatic treatment (e.g., antihistamines, NSAIDS, narcotics, IV fluids); prophylactic medications indicated for <=24 hrs	Prolonged (e.g., not rapidly responsive to symptomatic medication and/or brief interruption of infusion); recurrence of symptoms following initial improvement; hospitalization indicated for clinical sequelae	Life-threatening consequences; urgent intervention indicated	Death	A disorder characterized by adverse reaction to the infusion of pharmacological or biological substances.

MedDRA v12.0 Code	CTCAE v4.0 SOC	CTCAE v4.0 Term	Grade 1	Grade 2	Grade 3	Grade 4	Grade 5	CTCAE v4.0 AE Term Definition
10064774	General disorders and administration site conditions	Infusion site extravasation	-	Erythema with associated symptoms (e.g., edema, pain, induration, phlebitis)	Ulceration or necrosis; severe tissue damage; operative intervention indicated	Life-threatening consequences; urgent intervention indicated	Death	A disorder characterized by leakage of a pharmacologic or a biologic substance from the infusion site into the surrounding tissue. Signs and symptoms include induration, erythema, swelling, burning sensation and marked discomfort at the infusion site.
10022095	General disorders and administration site conditions	Injection site reaction	Tenderness with or without associated symptoms (e.g., warmth, erythema, itching)	Pain; lipodystrophy; edema; phlebitis	Ulceration or necrosis; severe tissue damage; operative intervention indicated	Life-threatening consequences; urgent intervention indicated	Death	A disorder characterized by an intense adverse reaction (usually immunologic) developing at the site of an injection.
10025482	General disorders and administration site conditions	Malaise	Uneasiness or lack of well being	Uneasiness or lack of well being; limiting instrumental ADL	-	-	-	A disorder characterized by a feeling of general discomfort or uneasiness, an out-of-sorts feeling.
10028411	Musculoskeletal and connective tissue disorders	Myalgia	Mild pain	Moderate pain; limiting instrumental ADL	Severe pain; limiting self care ADL	-	-	A disorder characterized by marked discomfort sensation originating from a muscle or group of muscles.

MedDRA v12.0 Code	CTCAE v4.0 SOC	CTCAE v4.0 Term	Grade 1	Grade 2	Grade 3	Grade 4	Grade 5	CTCAE v4.0 AE Term Definition
10028813	Gastrointestinal disorders	Nausea	Loss of appetite without alteration in eating habits	Oral intake decreased without significant weight loss, dehydration or malnutrition	Inadequate oral caloric or fluid intake; tube feeding, TPN, or hospitalization indicated	-	-	A disorder characterized by a queasy sensation and/or the urge to vomit.
10033371	General disorders and administration site conditions	Pain	Mild pain	Moderate pain; limiting instrumental ADL	Severe pain; limiting self care ADL	-	-	A disorder characterized by the sensation of marked discomfort, distress or agony.
10033557	Cardiac disorders	Palpitations	Mild symptoms; intervention not indicated	Intervention indicated	-	-	-	A disorder characterized by an unpleasant sensation of irregular and/or forceful beating of the heart.
10037087	Skin and subcutaneous tissue disorders	Pruritus	Mild or localized; topical intervention indicated	Intense or widespread; intermittent; skin changes from scratching (e.g., edema, papulation, excoriations, lichenification, oozing/crusts); oral intervention indicated; limiting instrumental ADL	Intense or widespread; constant; limiting self care ADL or sleep; oral corticosteroid or immunosuppressive therapy indicated	-	-	A disorder characterized by an intense itching sensation.

MedDRA v12.0 Code	CTCAE v4.0 SOC	CTCAE v4.0 Term	Grade 1	Grade 2	Grade 3	Grade 4	Grade 5	CTCAE v4.0 AE Term Definition
10037868	Skin and subcutaneous tissue disorders	Rash maculo-papular	Macules/papules covering <10% BSA with or without symptoms (e.g., pruritus, burning, tightness)	Macules/papules covering 10 - 30% BSA with or without symptoms (e.g., pruritus, burning, tightness); limiting instrumental ADL	Macules/papules covering >30% BSA with or without associated symptoms; limiting self care ADL	-	-	A disorder characterized by the presence of macules (flat) and papules (elevated). Also known as morbillform rash, it is one of the most common cutaneous adverse events, frequently affecting the upper trunk, spreading centripetally and associated with pruritis.

MedDRA v12.0 Code	CTCAE v4.0 SOC	CTCAE v4.0 Term	Grade 1	Grade 2	Grade 3	Grade 4	Grade 5	CTCAE v4.0 AE Term Definition
10040400	Immune system disorders	Serum sickness	Asymptomatic; clinical or diagnostic observations only; intervention not indicated	Moderate arthralgia; fever, rash, urticaria, antihistamines indicated	Severe arthralgia or arthritis; extensive rash; steroids or IV fluids indicated	Life-threatening consequences; pressor or ventilatory support indicated	Death	A disorder characterized by a delayed-type hypersensitivity reaction to foreign proteins derived from an animal serum. It occurs approximately six to twenty-one days following the administration of the foreign antigen. Symptoms include fever, arthralgias, myalgias, skin eruptions, lymphadenopathy, chest marked discomfort and dyspnea.
10051837	Skin and subcutaneous tissue disorders	Skin induration	Mild induration, able to move skin parallel to plane (sliding) and perpendicular to skin (pinching up)	Moderate induration, able to slide skin, unable to pinch skin; limiting instrumental ADL	Severe induration, unable to slide or pinch skin; limiting joint movement or orifice (e.g., mouth, anus); limiting self care ADL	Generalized; associated with signs or symptoms of impaired breathing or feeding	Death	A disorder characterized by an area of hardness in the skin.
10042241	Respiratory, thoracic and mediastinal disorders	Stridor	-	-	Respiratory distress limiting self care ADL; medical intervention indicated	Life-threatening airway compromise; urgent intervention indicated (e.g., tracheotomy or intubation)	Death	A disorder characterized by a high pitched breathing sound due to laryngeal or upper airway obstruction.

MedDRA v12.0 Code	CTCAE v4.0 SOC	CTCAE v4.0 Term	Grade 1	Grade 2	Grade 3	Grade 4	Grade 5	CTCAE v4.0 AE Term Definition
10046735	Skin and subcutaneous tissue disorders	Urticaria	Urticarial lesions covering <10% BSA; topical intervention indicated	Urticarial lesions covering 10 - 30% BSA; oral intervention indicated	Urticarial lesions covering >30% BSA; IV intervention indicated	-	-	A disorder characterized by an itchy skin eruption characterized by wheals with pale interiors and well-defined red margins.
10047700	Gastrointestinal disorders	Vomiting	1 - 2 episodes (separated by 5 minutes) in 24 hrs	3 - 5 episodes (separated by 5 minutes) in 24 hrs	>=6 episodes (separated by 5 minutes) in 24 hrs; tube feeding, TPN or hospitalization indicated	Life-threatening consequences; urgent intervention indicated	Death	A disorder characterized by the reflexive act of ejecting the contents of the stomach through the mouth.

APPENDIX G: DAIDS ADVERSE EVENT SEVERITY ASSESSMENT TABLE

Division of AIDS (DAIDS) Table for Grading the Severity of Adult and Pediatric Adverse Events

Version 2.0
November 2014

Division of AIDS
National Institute of Allergy and Infectious Diseases
National Institutes of Health
US Department of Health and Human Services

TABLE OF CONTENTS

Glossary and Acronyms 1
Introduction 3
Instructions for Use 4
Major Clinical Conditions 7
    Cardiovascular 7
    Dermatologic 9
    Endocrine and Metabolic 10
    Gastrointestinal 11
    Musculoskeletal 13
    Neurologic 14
    Pregnancy, Puerperium, and Perinatal 16
    Psychiatric 17
    Respiratory 18
    Sensory 19
    Systemic 20
    Urinary 22
Site Reactions to Injections and Infusions 23
Laboratory Values 24
    Chemistries 24
    Hematology 27
    Urinalysis 29
Appendix A. Total Bilirubin Table for Term and Preterm Neonates 30

GLOSSARY AND ACRONYMS

AE	Adverse event; Any unfavorable and unintended sign (including an abnormal laboratory finding), symptom, or disease temporally associated with the use of a medical treatment or procedure regardless of whether it is considered related to the medical treatment or procedure.
ALT (SGPT)	Alanine aminotransferase (serum glutamic pyruvic transaminase)
ANC	Absolute neutrophil count
AST (SGOT)	Aspartate aminotransferase (serum glutamic-oxaloacetic transaminase)
AV	Atrioventricular
Basic Self-care Functions	<u>Adult</u><br>Activities such as bathing, dressing, toileting, transfer or movement, continence, and feeding.<br><br><u>Young Children</u><br>Activities that are age and culturally appropriate, such as feeding one's self with culturally appropriate eating implements.
BMI z-score	Body mass index z- score; A body reference norm. Specifically, the number of standard deviations a participant's BMI differs from the average BMI for their age, sex, and ethnicity.
BMD t-score	Bone mineral density t-score; The number of standard deviations above or below the mean bone mineral density of a healthy 30 year old adult of the same sex and ethnicity as the participant.
BMD z-score	Bone mineral density z-score; The number of standard deviations a participant's BMD differs from the average BMD for their age, sex, and ethnicity.
BPAP	Bilevel positive airway pressure; A mode used during noninvasive positive pressure ventilation.
Chemical Pregnancy	A pregnancy in which a positive pregnancy test is followed by a negative pregnancy test without evidence of a clinical pregnancy loss.
CNS	Central nervous system
CPAP	Continuous positive airway pressure
DAERS	DAIDS Adverse Experience Reporting System; An internet-based system developed for clinical research sites to report Expedited Adverse Events (EAEs) to DAIDS. It facilitates timely EAE report submission and serves as a centralized location for accessing and processing EAE information for reporting purposes.
Disability	A substantial disruption of a person's ability to conduct normal life functions.
ECG	Electrocardiogram
eGFR	Estimated glomerular filtration rate
Hospitalization	Does not include the following hospital admissions: under 24 hours, unrelated to an adverse event (e.g., for labor and delivery, cosmetic surgery, social or administrative for temporary placement [for lack of a place to sleep]), protocol-specified, and for diagnosis or therapy of a condition that existed before the receipt of a study agent and which has not increased in severity or frequency.
INR	International normalized ratio

Glossary and Acronyms

Intervention	Medical, surgical, or other procedures recommended or provided by a healthcare professional for the treatment of an adverse event.
IV	Intravenous
IVIG	Intravenous immune globulin
LDL	Low density lipoprotein
LLN	Lower limit of normal
Life-threatening AE	Any adverse event that places the participant, in the view of the investigator, at immediate risk of death from the reaction when it occurred (i.e., it does not include a reaction that would have caused death if it had occurred in a more severe form).
NA	Not applicable
Participant ID	The identification number assigned to a study participant which is used to track study-related documentation, including any reported AEs.
PR Interval	The interval between the beginning of the P wave and the beginning of the QRS complex of an electrocardiogram that represents the time between the beginning of the contraction of the atria and the beginning of the contraction of the ventricles.
PT	Prothrombin time
PTT	Partial thromboplastin time
QTc Interval	The measure of time between the onset of ventricular depolarization and completion of ventricular repolarization corrected for ventricular rate.
RBC	Red blood cell
SI	Standard international unit
ULN	Upper limit of normal
Usual Social & Functional Activities	Activities which adults and children perform on a routine basis and those which are part of regular activities of daily living, for example:<br><br>Adults<br>Adaptive tasks and desirable activities, such as going to work, shopping, cooking, use of transportation, or pursuing a hobby.<br><br>Young Children<br>Activities that are age and culturally appropriate, such as social interactions, play activities, or learning tasks.
WBC	White blood cell
WHO	World Health Organization
WNL	Within normal limits

INTRODUCTION

The Division of AIDS (DAIDS) oversees clinical trials throughout the world which it sponsors and supports. The clinical trials evaluate the safety and efficacy of therapeutic products, vaccines, and other preventive modalities. Adverse event (AE) data collected during these clinical trials form the basis for subsequent safety and efficacy analyses of pharmaceutical products and medical devices. Incorrect and inconsistent AE severity grading can lead to inaccurate data analyses and interpretation, which in turn can impact the safety and well-being of clinical trial participants and future patients using pharmaceutical products.

The DAIDS AE grading table is a shared tool for assessing the severity of AEs (including clinical and laboratory abnormalities) in participants enrolled in clinical trials. Over the years as scientific knowledge and experience have expanded, revisions to the DAIDS AE grading table have become necessary.

The *Division of AIDS (DAIDS) Table for Grading the Severity of Adult and Pediatric Adverse Events, Version 2.0* replaces the grading table published in 2004 and updated in 2009. In version 2.0, AEs not previously included, but which now are deemed medically important events, are included while other AEs have been removed. Some AE severity grading descriptions have been revised to more appropriately reflect the presentation of these events in clinical settings and their impact on clinical trials. For example, DAIDS performed an extensive literature search and reviews of select DAIDS clinical trial data in revising certain hematology parameters (i.e., hemoglobin, white cell counts, and absolute neutrophil counts). DAIDS also took into consideration the U.S. Food and Drug Administration's guidance regarding the use of local laboratory reference values and ethnic differences among certain healthy adolescent and adult populations in defining parameter limits. Finally, the revised DAIDS AE grading table also contains an updated glossary and acronyms section, an expanded instructions for use section, and an appendix that provides more age-specific information for an AE of concern to DAIDS.

DAIDS is grateful to the DAIDS Grading Table Working Group, numerous government and non-government affiliated medical subject matter experts and reviewers who were instrumental in the revision of the DAIDS AE grading table.

Instructions for Use

General Considerations
The *Division of AIDS (DAIDS) Table for Grading the Severity of Adult and Pediatric Adverse Events, Version 2.0* consists of parameters, or AEs, with severity grading guidance that are to be used in DAIDS clinical trials for safety data reporting to maintain accuracy and consistency in the evaluation of AEs. The term "severe" is not the same as the term "serious" in classifying AEs. The severity of a specific event describes its intensity, and it is the intensity which is graded. Seriousness, which is not graded, relates to an outcome of an AE and is a regulatory definition.

Clinical sites are encouraged to report parameters in the DAIDS AE grading table as they are written to maintain data consistency across clinical trials. However, since some parameters can be reported with more specificity, clinical sites are encouraged to report parameters that convey additional clinical information. For example, diarrhea could be reported as neonatal diarrhea; seizures, as febrile seizures; and pain, as jaw pain.

The DAIDS AE grading table provides an AE severity grading scale ranging from grades 1 to 5 with descriptions for each AE based on the following general guidelines:

- Grade 1 indicates a mild event
- Grade 2 indicates a moderate event
- Grade 3 indicates a severe event
- Grade 4 indicates a potentially life-threatening event
- Grade 5 indicates death (*Note*: This grade is not specifically listed on each page of the grading table).

Other points to consider include:

- Use parameters defined by age and sex values as applicable.
- Male and female sex are defined as sex at birth.
- Unless noted, laboratory values are for term neonates. Preterm neonates should be assessed using local laboratory normal ranges.
- Where applicable, Standard International (SI) units are included in italics.

Selecting and Reporting a Primary AE Term
When selecting a primary AE term to report, sites should select the term that best describes what occurred to the participant. For example, a participant may present with itching, urticaria, flushing, angioedema of the face, and dyspnea. If the underlying diagnosis is determined to be an acute allergic reaction, sites should report "Acute Allergic Reaction" as the primary AE term.

Primary AE terms should be reported using the DAIDS Adverse Experience Reporting System (DAERS) only if they meet expedited reporting criteria. However, all primary AE terms should be reported using protocol-specific case report forms (CRFs). Because the reported information is stored in different databases (i.e., safety and clinical), sites should report primary AE terms using the same terminology for data consistency.

Instructions for Use

When reporting using DAERS, other clinically significant events associated with a primary AE term that more fully describe the nature, severity, or complications of the primary AE term should be entered in the "Other Events" section. However, the severity grade for these events must be lower than or equal to the severity grade of the primary AE term. In the example above, dyspnea and angioedema of the face may be entered in the "Other Events" section, because they are more descriptive and provide additional information on the severity of the acute allergic reaction. However, their severity grades must be lower than or equal to the severity grade of the primary AE term of "Acute Allergic Reaction".

Differences exist in the reporting and recording of information (e.g., signs and symptoms, clinically significant events) in DAERS and CRFs. Therefore, sites should refer to their protocols and CRF requirements for further instructions.

Grading Adult and Pediatric AEs
When a single parameter is not appropriate for grading an AE in both adult and pediatric populations, separate parameters with specified age ranges are provided. If no distinction between adult and pediatric populations has been made, the listed parameter should be used for grading an AE in both populations.

Reporting Pregnancy Outcomes
In the *Pregnancy, Puerperium, and Perinatal* section, all parameters are pregnancy outcomes and should be reported using the mother's participant ID. If an infant is not enrolled in the same study as the mother, any identified birth defects should be reported using the mother's participant ID. However, if an infant is enrolled in the same study as the mother or in another study, any identified birth defects should be reported using the infant's participant ID. Sites should refer to the applicable network standards for reporting abnormal pregnancy outcomes on the CRFs.

Determining Severity Grade for Parameters between Grades
If the severity of an AE could fall in either one of two grades (i.e., the severity of an AE could be either grade 2 or grade 3), sites should select the higher of the two grades.

Laboratory Values
General. An asymptomatic, abnormal laboratory finding without an accompanying AE should not be reported to DAIDS in an expedited timeframe unless it meets protocol-specific reporting requirements. Sites should refer to the applicable network standards for reporting abnormal laboratory findings on the CRFs.

Values below Grade 1. Any laboratory value that is between the ULN and grade 1 (for high values) or the LLN and grade 1 (for low values) should not be graded or reported as an AE. Sites should consult the *Manual for Expedited Reporting of Adverse Events to DAIDS, Version 2.0* and their protocol when making an assessment of the need to report an AE.

Overlap of Local Laboratory Normal Values with Grading Table Ranges. When local laboratory normal values fall within grading table laboratory ranges, the severity grading is based on the ranges in the grading table unless there is a protocol-specific grading criterion for the laboratory

value. For example, “Magnesium, Low" has a grade 1 range of 1.2 to < 1.4 mEq/L, while a particular laboratory’s normal range for magnesium may be 1.3 to 2.8 mEq/L. If a study participant’s magnesium laboratory value is 1.3 mEq/L, the laboratory value should be graded as grade 1.

Appendix Usage
Appendix A takes priority over the main grading table in all assessments of total bilirubin for term and preterm neonates.

Using Addenda 1-3: Grading Tables Used in Microbicide Studies
In protocols involving topical application of products to the female and male genital tracts or rectum, strong consideration should be given to using Addenda 1-3 (see below) as the primary grading tables for these areas. Although these grading tables are used specifically in microbicide studies, they may be used in other protocols as adjuncts to the main grading table (i.e., the *Division of AIDS (AIDS) Table for Grading the Severity of Adult and Pediatric Adverse Events, Version 2.0*). It should be clearly stated in a protocol which addendum is being used as the primary grading table (and thus takes precedence over the main grading table) and which addendum is being used in a complementary fashion.

- Addendum 1 – Female Genital Grading Table for Use in Microbicide Studies – PDF
- Addendum 2 – Male Genital Grading Table for Use in Microbicide Studies – PDF
- Addendum 3 – Rectal Grading Table for Use in Microbicide Studies – PDF

Estimating Severity Grade for Parameters Not Identified in the Grading Table
The functional table below should be used to grade the severity of an AE that is not specifically identified in the grading table. In addition, all deaths related to an AE are to be classified as grade 5.

PARAMETER	GRADE 1 MILD	GRADE 2 MODERATE	GRADE 3 SEVERE	GRADE 4 POTENTIALLY LIFE-THREATENING
**Clinical** adverse event **NOT** identified elsewhere in the grading table	Mild symptoms causing no or minimal interference with usual social & functional activities with intervention not indicated	Moderate symptoms causing greater than minimal interference with usual social & functional activities with intervention indicated	Severe symptoms causing inability to perform usual social & functional activities with intervention or hospitalization indicated	Potentially life-threatening symptoms causing inability to perform basic self-care functions with intervention indicated to prevent permanent impairment, persistent disability, or death

MAJOR CLINICAL CONDITIONS
CARDIOVASCULAR

PARAMETER	GRADE 1 MILD	GRADE 2 MODERATE	GRADE 3 SEVERE	GRADE 4 POTENTIALLY LIFE-THREATENING
**Arrhythmia** (by ECG or physical examination) *Specify type, if applicable*	No symptoms AND No intervention indicated	No symptoms AND Non-urgent intervention indicated	Non-life-threatening symptoms AND Non-urgent intervention indicated	Life-threatening arrhythmia OR Urgent intervention indicated
**Blood Pressure Abnormalities**[1] ***Hypertension*** *(with the lowest reading taken after repeat testing during a visit)* *≥ 18 years of age*	140 to < 160 mmHg systolic OR 90 to < 100 mmHg diastolic	≥ 160 to < 180 mmHg systolic OR ≥ 100 to < 110 mmHg diastolic	≥ 180 mmHg systolic OR ≥ 110 mmHg diastolic	Life-threatening consequences in a participant not previously diagnosed with hypertension (e.g., malignant hypertension) OR Hospitalization indicated
< 18 years of age	> 120/80 mmHg	≥ 95th to < 99th percentile + 5 mmHg adjusted for age, height, and gender (systolic and/or diastolic)	≥ 99th percentile + 5 mmHg adjusted for age, height, and gender (systolic and/or diastolic)	Life-threatening consequences in a participant not previously diagnosed with hypertension (e.g., malignant hypertension) OR Hospitalization indicated
Hypotension	No symptoms	Symptoms corrected with oral fluid replacement	Symptoms AND IV fluids indicated	Shock requiring use of vasopressors or mechanical assistance to maintain blood pressure
**Cardiac Ischemia or Infarction** *Report only one*	NA	NA	New symptoms with ischemia (stable angina) OR New testing consistent with ischemia	Unstable angina OR Acute myocardial infarction
**Heart Failure**	No symptoms AND Laboratory or cardiac imaging abnormalities	Symptoms with mild to moderate activity or exertion	Symptoms at rest or with minimal activity or exertion (e.g., hypoxemia) OR Intervention indicated (e.g., oxygen)	Life-threatening consequences OR Urgent intervention indicated (e.g., vasoactive medications, ventricular assist device, heart transplant)
**Hemorrhage** (with significant acute blood loss)	NA	Symptoms AND No transfusion indicated	Symptoms AND Transfusion of ≤ 2 units packed RBCs indicated	Life-threatening hypotension OR Transfusion of > 2 units packed RBCs (for children, packed RBCs > 10 cc/kg) indicated

[1] Blood pressure norms for children < 18 years of age can be found in: Expert Panel on Integrated Guidelines for Cardiovascular Health and Risk Reduction in Children and Adolescents. *Pediatrics* 2011;128;S213; originally published online November 14, 2011; DOI: 10.1542/peds.2009-2107C.

CARDIOVASCULAR

PARAMETER	GRADE 1 MILD	GRADE 2 MODERATE	GRADE 3 SEVERE	GRADE 4 POTENTIALLY LIFE-THREATENING
**Prolonged PR Interval or AV Block** *Report only one* *> 16 years of age*	PR interval 0.21 to < 0.25 seconds	PR interval ≥ 0.25 seconds OR Type I 2nd degree AV block	Type II 2nd degree AV block OR Ventricular pause ≥ 3.0 seconds	Complete AV block
≤ 16 years of age	1st degree AV block (PR interval > normal for age and rate)	Type I 2nd degree AV block	Type II 2nd degree AV block OR Ventricular pause ≥ 3.0 seconds	Complete AV block
**Prolonged QTc Interval[2]**	0.45 to 0.47 seconds	> 0.47 to 0.50 seconds	> 0.50 seconds OR ≥ 0.06 seconds above baseline	Life-threatening consequences (e.g., Torsade de pointes, other associated serious ventricular dysrhythmia)
**Thrombosis or Embolism** *Report only one*	NA	Symptoms AND No intervention indicated	Symptoms AND Intervention indicated	Life-threatening embolic event (e.g., pulmonary embolism, thrombus)

[2] As per Bazett's formula.

DERMATOLOGIC

PARAMETER	GRADE 1 MILD	GRADE 2 MODERATE	GRADE 3 SEVERE	GRADE 4 POTENTIALLY LIFE-THREATENING
**Alopecia** (scalp only)	Detectable by study participant, caregiver, or physician AND Causing no or minimal interference with usual social & functional activities	Obvious on visual inspection AND Causing greater than minimal interference with usual social & functional activities	NA	NA
**Bruising**	Localized to one area	Localized to more than one area	Generalized	NA
**Cellulitis**	NA	Non-parenteral treatment indicated (e.g., oral antibiotics, antifungals, antivirals)	IV treatment indicated (e.g., IV antibiotics, antifungals, antivirals)	Life-threatening consequences (e.g., sepsis, tissue necrosis)
**Hyperpigmentation**	Slight or localized causing no or minimal interference with usual social & functional activities	Marked or generalized causing greater than minimal interference with usual social & functional activities	NA	NA
**Hypopigmentation**	Slight or localized causing no or minimal interference with usual social & functional activities	Marked or generalized causing greater than minimal interference with usual social & functional activities	NA	NA
**Petechiae**	Localized to one area	Localized to more than one area	Generalized	NA
**Pruritus**[3] (without skin lesions)	Itching causing no or minimal interference with usual social & functional activities	Itching causing greater than minimal interference with usual social & functional activities	Itching causing inability to perform usual social & functional activities	NA
**Rash** *Specify type, if applicable*	Localized rash	Diffuse rash OR Target lesions	Diffuse rash AND Vesicles or limited number of bullae or superficial ulcerations of mucous membrane limited to one site	Extensive or generalized bullous lesions OR Ulceration of mucous membrane involving two or more distinct mucosal sites OR Stevens-Johnson syndrome OR Toxic epidermal necrolysis

[3] For pruritus associated with injections or infusions, see the *Site Reactions to Injections and Infusions* section (page 23).

Endocrine and Metabolic

PARAMETER	GRADE 1 MILD	GRADE 2 MODERATE	GRADE 3 SEVERE	GRADE 4 POTENTIALLY LIFE-THREATENING
**Diabetes Mellitus**	Controlled without medication	Controlled with medication OR Modification of current medication regimen	Uncontrolled despite treatment modification OR Hospitalization for immediate glucose control indicated	Life-threatening consequences (e.g., ketoacidosis, hyperosmolar non-ketotic coma, end organ failure)
**Gynecomastia**	Detectable by study participant, caregiver, or physician AND Causing no or minimal interference with usual social & functional activities	Obvious on visual inspection AND Causing pain with greater than minimal interference with usual social & functional activities	Disfiguring changes AND Symptoms requiring intervention or causing inability to perform usual social & functional activities	NA
**Hyperthyroidism**	No symptoms AND Abnormal laboratory value	Symptoms causing greater than minimal interference with usual social & functional activities OR Thyroid suppression therapy indicated	Symptoms causing inability to perform usual social & functional activities OR Uncontrolled despite treatment modification	Life-threatening consequences (e.g., thyroid storm)
**Hypothyroidism**	No symptoms AND Abnormal laboratory value	Symptoms causing greater than minimal interference with usual social & functional activities OR Thyroid replacement therapy indicated	Symptoms causing inability to perform usual social & functional activities OR Uncontrolled despite treatment modification	Life-threatening consequences (e.g., myxedema coma)
**Lipoatrophy**[4]	Detectable by study participant, caregiver, or physician AND Causing no or minimal interference with usual social & functional activities	Obvious on visual inspection AND Causing greater than minimal interference with usual social & functional activities	Disfiguring changes	NA
**Lipohypertrophy**[5]	Detectable by study participant, caregiver, or physician AND Causing no or minimal interference with usual social & functional activities	Obvious on visual inspection AND Causing greater than minimal interference with usual social & functional activities	Disfiguring changes	NA

[4] Definition: A disorder characterized by fat loss in the face, extremities, and buttocks.
[5] Definition: A disorder characterized by abnormal fat accumulation on the back of the neck, breasts, and abdomen.

GASTROINTESTINAL

PARAMETER	GRADE 1 MILD	GRADE 2 MODERATE	GRADE 3 SEVERE	GRADE 4 POTENTIALLY LIFE-THREATENING
**Anorexia**	Loss of appetite without decreased oral intake	Loss of appetite associated with decreased oral intake without significant weight loss	Loss of appetite associated with significant weight loss	Life-threatening consequences OR Aggressive intervention indicated (e.g., tube feeding, total parenteral nutrition)
**Ascites**	No symptoms	Symptoms AND Intervention indicated (e.g., diuretics, therapeutic paracentesis)	Symptoms recur or persist despite intervention	Life-threatening consequences
**Bloating or Distension** *Report only one*	Symptoms causing no or minimal interference with usual social & functional activities	Symptoms causing greater than minimal interference with usual social & functional activities	Symptoms causing inability to perform usual social & functional activities	NA
**Cholecystitis**	NA	Symptoms AND Medical intervention indicated	Radiologic, endoscopic, or operative intervention indicated	Life-threatening consequences (e.g., sepsis, perforation)
**Constipation**	NA	Persistent constipation requiring regular use of dietary modifications, laxatives, or enemas	Obstipation with manual evacuation indicated	Life-threatening consequences (e.g., obstruction)
**Diarrhea** *≥ 1 year of age*	Transient or intermittent episodes of unformed stools OR Increase of ≤ 3 stools over baseline per 24-hour period	Persistent episodes of unformed to watery stools OR Increase of 4 to 6 stools over baseline per 24-hour period	Increase of ≥ 7 stools per 24-hour period OR IV fluid replacement indicated	Life-threatening consequences (e.g., hypotensive shock)
< 1 year of age	Liquid stools (more unformed than usual) but usual number of stools	Liquid stools with increased number of stools OR Mild dehydration	Liquid stools with moderate dehydration	Life-threatening consequences (e.g., liquid stools resulting in severe dehydration, hypotensive shock)
**Dysphagia or Odynophagia** *Report only one and specify location*	Symptoms but able to eat usual diet	Symptoms causing altered dietary intake with no intervention indicated	Symptoms causing severely altered dietary intake with intervention indicated	Life-threatening reduction in oral intake
**Gastrointestinal Bleeding**	Not requiring intervention other than iron supplement	Endoscopic intervention indicated	Transfusion indicated	Life-threatening consequences (e.g., hypotensive shock)

GASTROINTESTINAL

PARAMETER	GRADE 1 MILD	GRADE 2 MODERATE	GRADE 3 SEVERE	GRADE 4 POTENTIALLY LIFE-THREATENING
**Mucositis or Stomatitis** *Report only one and specify location*	Mucosal erythema	Patchy pseudomembranes or ulcerations	Confluent pseudomembranes or ulcerations OR Mucosal bleeding with minor trauma	Life-threatening consequences (e.g., aspiration, choking) OR Tissue necrosis OR Diffuse spontaneous mucosal bleeding
**Nausea**	Transient (< 24 hours) or intermittent AND No or minimal interference with oral intake	Persistent nausea resulting in decreased oral intake for 24 to 48 hours	Persistent nausea resulting in minimal oral intake for > 48 hours OR Rehydration indicated (e.g., IV fluids)	Life-threatening consequences (e.g., hypotensive shock)
**Pancreatitis**	NA	Symptoms with hospitalization not indicated	Symptoms with hospitalization indicated	Life-threatening consequences (e.g., circulatory failure, hemorrhage, sepsis)
**Perforation** (colon or rectum)	NA	NA	Intervention indicated	Life-threatening consequences
**Proctitis**	Rectal discomfort with no intervention indicated	Symptoms causing greater than minimal interference with usual social & functional activities OR Medical intervention indicated	Symptoms causing inability to perform usual social & functional activities OR Operative intervention indicated	Life-threatening consequences (e.g., perforation)
**Rectal Discharge**	Visible discharge	Discharge requiring the use of pads	NA	NA
**Vomiting**	Transient or intermittent AND No or minimal interference with oral intake	Frequent episodes with no or mild dehydration	Persistent vomiting resulting in orthostatic hypotension OR Aggressive rehydration indicated (e.g., IV fluids)	Life-threatening consequences (e.g., hypotensive shock)

MUSCULOSKELETAL

PARAMETER	GRADE 1 MILD	GRADE 2 MODERATE	GRADE 3 SEVERE	GRADE 4 POTENTIALLY LIFE-THREATENING
**Arthralgia**	Joint pain causing no or minimal interference with usual social & functional activities	Joint pain causing greater than minimal interference with usual social & functional activities	Joint pain causing inability to perform usual social & functional activities	Disabling joint pain causing inability to perform basic self-care functions
**Arthritis**	Stiffness or joint swelling causing no or minimal interference with usual social & functional activities	Stiffness or joint swelling causing greater than minimal interference with usual social & functional activities	Stiffness or joint swelling causing inability to perform usual social & functional activities	Disabling joint stiffness or swelling causing inability to perform basic self-care functions
**Myalgia** (generalized)	Muscle pain causing no or minimal interference with usual social & functional activities	Muscle pain causing greater than minimal interference with usual social & functional activities	Muscle pain causing inability to perform usual social & functional activities	Disabling muscle pain causing inability to perform basic self-care functions
**Osteonecrosis**	NA	No symptoms but with radiographic findings AND No operative intervention indicated	Bone pain with radiographic findings OR Operative intervention indicated	Disabling bone pain with radiographic findings causing inability to perform basic self-care functions
**Osteopenia**[6] *≥ 30 years of age*	BMD t-score -2.5 to -1	NA	NA	NA
< 30 years of age	BMD z-score -2 to -1	NA	NA	NA
**Osteoporosis**[6] *≥ 30 years of age*	NA	BMD t-score < -2.5	Pathologic fracture (e.g., compression fracture causing loss of vertebral height)	Pathologic fracture causing life-threatening consequences
< 30 years of age	NA	BMD z-score < -2	Pathologic fracture (e.g., compression fracture causing loss of vertebral height)	Pathologic fracture causing life-threatening consequences

[6] BMD t and z scores can be found in: Kanis JA on behalf of the World Health Organization Scientific Group (2007). Assessment of osteoporosis at the primary health-care level. Technical Report. World Health Organization Collaborating Centre for Metabolic Bone Diseases, University of Sheffield, UK. 2007: Printed by the University of Sheffield.

NEUROLOGIC

PARAMETER	GRADE 1 MILD	GRADE 2 MODERATE	GRADE 3 SEVERE	GRADE 4 POTENTIALLY LIFE-THREATENING
**Acute CNS Ischemia**	NA	NA	Transient ischemic attack	Cerebral vascular accident (e.g., stroke with neurological deficit)
**Altered Mental Status** (for Dementia, see *Cognitive, Behavioral, or Attentional Disturbance* below)	Changes causing no or minimal interference with usual social & functional activities	Mild lethargy or somnolence causing greater than minimal interference with usual social & functional activities	Confusion, memory impairment, lethargy, or somnolence causing inability to perform usual social & functional activities	Delirium OR Obtundation OR Coma
**Ataxia**	Symptoms causing no or minimal interference with usual social & functional activities OR No symptoms with ataxia detected on examination	Symptoms causing greater than minimal interference with usual social & functional activities	Symptoms causing inability to perform usual social & functional activities	Disabling symptoms causing inability to perform basic self-care functions
**Cognitive, Behavioral, or Attentional Disturbance** (includes dementia and attention deficit disorder)<br>*Specify type, if applicable*	Disability causing no or minimal interference with usual social & functional activities OR Specialized resources not indicated	Disability causing greater than minimal interference with usual social & functional activities OR Specialized resources on part-time basis indicated	Disability causing inability to perform usual social & functional activities OR Specialized resources on a full-time basis indicated	Disability causing inability to perform basic self-care functions OR Institutionalization indicated
**Developmental Delay**<br>*< 18 years of age*<br>*Specify type, if applicable*	Mild developmental delay, either motor or cognitive, as determined by comparison with a developmental screening tool appropriate for the setting	Moderate developmental delay, either motor or cognitive, as determined by comparison with a developmental screening tool appropriate for the setting	Severe developmental delay, either motor or cognitive, as determined by comparison with a developmental screening tool appropriate for the setting	Developmental regression, either motor or cognitive, as determined by comparison with a developmental screening tool appropriate for the setting
**Headache**	Symptoms causing no or minimal interference with usual social & functional activities	Symptoms causing greater than minimal interference with usual social & functional activities	Symptoms causing inability to perform usual social & functional activities	Symptoms causing inability to perform basic self-care functions OR Hospitalization indicated OR Headache with significant impairment of alertness or other neurologic function

NEUROLOGIC

PARAMETER	GRADE 1 MILD	GRADE 2 MODERATE	GRADE 3 SEVERE	GRADE 4 POTENTIALLY LIFE-THREATENING
**Neuromuscular Weakness** (includes myopathy and neuropathy)<br>*Specify type, if applicable*	Minimal muscle weakness causing no or minimal interference with usual social & functional activities OR No symptoms with decreased strength on examination	Muscle weakness causing greater than minimal interference with usual social & functional activities	Muscle weakness causing inability to perform usual social & functional activities	Disabling muscle weakness causing inability to perform basic self-care functions OR Respiratory muscle weakness impairing ventilation
**Neurosensory Alteration** (includes paresthesia and painful neuropathy)<br>*Specify type, if applicable*	Minimal paresthesia causing no or minimal interference with usual social & functional activities OR No symptoms with sensory alteration on examination	Sensory alteration or paresthesia causing greater than minimal interference with usual social & functional activities	Sensory alteration or paresthesia causing inability to perform usual social & functional activities	Disabling sensory alteration or paresthesia causing inability to perform basic self-care functions
**Seizures**<br>***New Onset Seizure***<br>*≥ 18 years of age*	NA	NA	1 to 3 seizures	Prolonged and repetitive seizures (e.g., status epilepticus) OR Difficult to control (e.g., refractory epilepsy)
< 18 years of age (includes new or pre-existing febrile seizures)	Seizure lasting < 5 minutes with < 24 hours postictal state	Seizure lasting 5 to < 20 minutes with < 24 hours postictal state	Seizure lasting ≥ 20 minutes OR > 24 hours postictal state	Prolonged and repetitive seizures (e.g., status epilepticus) OR Difficult to control (e.g., refractory epilepsy)
Pre-existing Seizure	NA	Increased frequency from previous level of control without change in seizure character	Change in seizure character either in duration or quality (e.g., severity or focality)	Prolonged and repetitive seizures (e.g., status epilepticus) OR Difficult to control (e.g., refractory epilepsy)
**Syncope**	Near syncope without loss of consciousness (e.g., pre-syncope)	Loss of consciousness with no intervention indicated	Loss of consciousness AND Hospitalization or intervention required	NA

Pregnancy, Puerperium, and Perinatal

PARAMETER	GRADE 1 MILD	GRADE 2 MODERATE	GRADE 3 SEVERE	GRADE 4 POTENTIALLY LIFE-THREATENING
**Fetal Death or Stillbirth** (report using mother's participant ID)<br>*Report only one*	NA	NA	Fetal loss occurring at ≥ 20 weeks gestation	NA
**Preterm Delivery**[7] (report using mother's participant ID)	Delivery at 34 to < 37 weeks gestational age	Delivery at 28 to < 34 weeks gestational age	Delivery at 24 to < 28 weeks gestational age	Delivery at < 24 weeks gestational age
**Spontaneous Abortion or Miscarriage**[8] (report using mother's participant ID)<br>*Report only one*	Chemical pregnancy	Uncomplicated spontaneous abortion or miscarriage	Complicated spontaneous abortion or miscarriage	NA

[7] Definition: A delivery of a live-born neonate occurring at ≥ 20 to < 37 weeks gestational age.
[8] Definition: A clinically recognized pregnancy occurring at < 20 weeks gestational

PSYCHIATRIC

PARAMETER	GRADE 1 MILD	GRADE 2 MODERATE	GRADE 3 SEVERE	GRADE 4 POTENTIALLY LIFE-THREATENING
**Insomnia**	Mild difficulty falling asleep, staying asleep, or waking up early	Moderate difficulty falling asleep, staying asleep, or waking up early	Severe difficulty falling asleep, staying asleep, or waking up early	NA
**Psychiatric Disorders** (includes anxiety, depression, mania, and psychosis) *Specify disorder*	Symptoms with intervention not indicated OR Behavior causing no or minimal interference with usual social & functional activities	Symptoms with intervention indicated OR Behavior causing greater than minimal interference with usual social & functional activities	Symptoms with hospitalization indicated OR Behavior causing inability to perform usual social & functional activities	Threatens harm to self or others OR Acute psychosis OR Behavior causing inability to perform basic self-care functions
**Suicidal Ideation or Attempt** *Report only one*	Preoccupied with thoughts of death AND No wish to kill oneself	Preoccupied with thoughts of death AND Wish to kill oneself with no specific plan or intent	Thoughts of killing oneself with partial or complete plans but no attempt to do so OR Hospitalization indicated	Suicide attempted

RESPIRATORY

PARAMETER	GRADE 1 MILD	GRADE 2 MODERATE	GRADE 3 SEVERE	GRADE 4 POTENTIALLY LIFE-THREATENING
**Acute Bronchospasm**	Forced expiratory volume in 1 second or peak flow reduced to ≥ 70 to < 80% OR Mild symptoms with intervention not indicated	Forced expiratory volume in 1 second or peak flow 50 to < 70% OR Symptoms with intervention indicated OR Symptoms causing greater than minimal interference with usual social & functional activities	Forced expiratory volume in 1 second or peak flow 25 to < 50% OR Symptoms causing inability to perform usual social & functional activities	Forced expiratory volume in 1 second or peak flow < 25% OR Life-threatening respiratory or Hemodynamic compromise OR Intubation
**Dyspnea or Respiratory Distress** *Report only one*	Dyspnea on exertion with no or minimal interference with usual social & functional activities OR Wheezing OR Minimal increase in respiratory rate for age	Dyspnea on exertion causing greater than minimal interference with usual social & functional activities OR Nasal flaring OR Intercostal retractions OR Pulse oximetry 90 to < 95%	Dyspnea at rest causing inability to perform usual social & functional activities OR Pulse oximetry < 90%	Respiratory failure with ventilator support indicated (e.g., CPAP, BPAP, intubation)

SENSORY

PARAMETER	GRADE 1 MILD	GRADE 2 MODERATE	GRADE 3 SEVERE	GRADE 4 POTENTIALLY LIFE-THREATENING
**Hearing Loss** *≥ 12 years of age*	NA	Hearing aid or intervention not indicated	Hearing aid or intervention indicated	Profound bilateral hearing loss (> 80 dB at 2 kHz and above) OR Non-serviceable hearing (i.e., >50 dB audiogram and <50% speech discrimination)
< 12 years of age (based on a 1, 2, 3, 4, 6 and 8 kHz audiogram)	> 20 dB hearing loss at ≤ 4 kHz	> 20 dB hearing loss at > 4 kHz	> 20 dB hearing loss at ≥ 3 kHz in one ear with additional speech language related services indicated (where available) OR Hearing loss sufficient to indicate therapeutic intervention, including hearing aids	Audiologic indication for cochlear implant and additional speech-language related services indicated (where available)
**Tinnitus**	Symptoms causing no or minimal interference with usual social & functional activities with intervention not indicated	Symptoms causing greater than minimal interference with usual social & functional activities with intervention indicated	Symptoms causing inability to perform usual social & functional activities	NA
**Uveitis**	No symptoms AND Detectable on examination	Anterior uveitis with symptoms OR Medicamylasal intervention indicated	Posterior or pan-uveitis OR Operative intervention indicated	Disabling visual loss in affected eye(s)
**Vertigo**	Vertigo causing no or minimal interference with usual social & functional activities	Vertigo causing greater than minimal interference with usual social & functional activities	Vertigo causing inability to perform usual social & functional activities	Disabling vertigo causing inability to perform basic self-care functions
**Visual Changes** (assessed from baseline)	Visual changes causing no or minimal interference with usual social & functional activities	Visual changes causing greater than minimal interference with usual social & functional activities	Visual changes causing inability to perform usual social & functional activities	Disabling visual loss in affected eye(s)

SYSTEMIC

PARAMETER	GRADE 1 MILD	GRADE 2 MODERATE	GRADE 3 SEVERE	GRADE 4 POTENTIALLY LIFE-THREATENING
**Acute Allergic Reaction**	Localized urticaria (wheals) with no medical intervention indicated	Localized urticaria with intervention indicated OR Mild angioedema with no intervention indicated	Generalized urticaria OR Angioedema with intervention indicated OR Symptoms of mild bronchospasm	Acute anaphylaxis OR Life-threatening bronchospasm OR Laryngeal edema
**Chills**	Symptoms causing no or minimal interference with usual social & functional activities	Symptoms causing greater than minimal interference with usual social & functional activities	Symptoms causing inability to perform usual social & functional activities	NA
**Cytokine Release Syndrome**[9]	Mild signs and symptoms AND Therapy (i.e., antibody infusion) interruption not indicated	Therapy (i.e., antibody infusion) interruption indicated AND Responds promptly to symptomatic treatment OR Prophylactic medications indicated for ≤ 24 hours	Prolonged severe signs and symptoms OR Recurrence of symptoms following initial improvement	Life-threatening consequences (e.g., requiring pressor or ventilator support)
**Fatigue or Malaise** *Report only one*	Symptoms causing no or minimal interference with usual social & functional activities	Symptoms causing greater than minimal interference with usual social & functional activities	Symptoms causing inability to perform usual social & functional activities	Incapacitating symptoms of fatigue or malaise causing inability to perform basic self-care functions
**Fever** (non-axillary temperatures only)	38.0 to < 38.6°C or 100.4 to < 101.5°F	≥ 38.6 to < 39.3°C or ≥101.5 to < 102.7°F	≥ 39.3 to < 40.0°C or ≥ 102.7 to < 104.0°F	≥ 40.0°C or ≥ 104.0°F
**Pain**[10] (not associated with study agent injections and not specified elsewhere) *Specify location*	Pain causing no or minimal interference with usual social & functional activities	Pain causing greater than minimal interference with usual social & functional activities	Pain causing inability to perform usual social & functional activities	Disabling pain causing inability to perform basic self-care functions OR Hospitalization indicated
**Serum Sickness**[11]	Mild signs and symptoms	Moderate signs and symptoms AND Intervention indicated (e.g., antihistamines)	Severe signs and symptoms AND Higher level intervention indicated (e.g., steroids or IV fluids)	Life-threatening consequences (e.g., requiring pressor or ventilator support)

SYSTEMIC

PARAMETER	GRADE 1 MILD	GRADE 2 MODERATE	GRADE 3 SEVERE	GRADE 4 POTENTIALLY LIFE-THREATENING
**Underweight[12]** *> 5 to 19 years of age*	NA	WHO BMI z-score < -2 to ≤ -3	WHO BMI z-score < -3	WHO BMI z-score < -3 with life-threatening consequences
2 to 5 years of age	NA	WHO Weight-for-height z-score < -2 to ≤-3	WHO Weight-for-height z-score < -3	WHO Weight-for-height z-score < -3 with life-threatening consequences
< 2 years of age	NA	WHO Weight-for-length z-score < -2 to ≤-3	WHO Weight-for-length z-score < -3	WHO Weight-for-length z-score < -3 with life-threatening consequences
**Weight Loss** (excludes postpartum weight loss)	NA	5 to < 9% loss in body weight from baseline	≥ 9 to < 20% loss in body weight from baseline	≥ 20% loss in body weight from baseline OR Aggressive intervention indicated (e.g., tube feeding, total parenteral nutrition)

[9] Definition: A disorder characterized by nausea, headache, tachycardia, hypotension, rash, and/or shortness of breath.
[10] For pain associated with injections or infusions, see the *Site Reactions to Injections and Infusions* section (page 23).
[11] Definition: A disorder characterized by fever, arthralgia, myalgia, skin eruptions, lymphadenopathy, marked discomfort, and/or dyspnea.

[12] WHO reference tables may be accesed by clicking the desired age range or by accessing the following URLs: http://www.who.int/growthref/who2007 bmi for age/en/ for participants > 5 to 19 years of age and http://www.who.int/childgrowth/standards/chart catalogue/en/ for those < 5 years of age.

URINARY

PARAMETER	GRADE 1 MILD	GRADE 2 MODERATE	GRADE 3 SEVERE	GRADE 4 POTENTIALLY LIFE-THREATENING
**Urinary Tract Obstruction**	NA	Signs or symptoms of urinary tract obstruction without hydronephrosis or renal dysfunction	Signs or symptoms of urinary tract obstruction with hydronephrosis or renal dysfunction	Obstruction causing life-threatening consequences

Site Reactions to Injections and Infusions

PARAMETER	GRADE 1 MILD	GRADE 2 MODERATE	GRADE 3 SEVERE	GRADE 4 POTENTIALLY LIFE-THREATENING
**Injection Site Pain or Tenderness**<br>*Report only one*	Pain or tenderness causing no or minimal limitation of use of limb	Pain or tenderness causing greater than minimal limitation of use of limb	Pain or tenderness causing inability to perform usual social & functional activities	Pain or tenderness causing inability to perform basic self-care function OR<br>Hospitalization indicated
**Injection Site Erythema or Redness**[13]<br>*Report only one*<br>*> 15 years of age*	2.5 to < 5 cm in diameter OR 6.25 to < 25 cm² surface area AND<br>Symptoms causing no or minimal interference with usual social & functional activities	≥ 5 to < 10 cm in diameter OR ≥ 25 to < 100 cm² surface area OR Symptoms causing greater than minimal interference with usual social & functional activities	≥ 10 cm in diameter OR ≥ 100 cm² surface area OR Ulceration OR Secondary infection OR Phlebitis OR Sterile abscess OR Drainage OR<br>Symptoms causing inability to perform usual social & functional activities	Potentially life-threatening consequences (e.g., abscess, exfoliative dermatitis, necrosis involving dermis or deeper tissue)
≤15 years of age	≤ 2.5 cm in diameter	> 2.5 cm in diameter with < 50% surface area of the extremity segment involved (e.g., upper arm or thigh)	≥ 50% surface area of the extremity segment involved (e.g., upper arm or thigh) OR Ulceration OR Secondary infection OR Phlebitis OR Sterile abscess OR Drainage	Potentially life-threatening consequences (e.g., abscess, exfoliative dermatitis, necrosis involving dermis or deeper tissue)
**Injection Site Induration or Swelling**<br>*Report only one*<br>*> 15 years of age*	Same as for **Injection Site Erythema or Redness,** *> 15 years of age*	Same as for **Injection Site Erythema or Redness,** *> 15 years of age*	Same as for **Injection Site Erythema or Redness,** *> 15 years of age*	Same as for **Injection Site Erythema or Redness,** *> 15 years of age*
≤ 15 years of age	Same as for **Injection Site Erythema or Redness,** *≤ 15 years of age*	Same as for **Injection Site Erythema or Redness,** *≤ 15 years of age*	Same as for **Injection Site Erythema or Redness,** *≤ 15 years of age*	Same as for **Injection Site Erythema or Redness,** *≤ 15 years of age*
**Injection Site Pruritus**	Itching localized to the injection site that is relieved spontaneously or in < 48 hours of treatment	Itching beyond the injection site that is not generalized OR<br>Itching localized to the injection site requiring ≥ 48 hours treatment	Generalized itching causing inability to perform usual social & functional activities	NA

[13] Injection Site Erythema or Redness should be evaluated and graded using the greatest single diameter or measured surface area.

[14] Direct bilirubin > 1.5 mg/dL in a participant < 28 days of age should be graded as grade 2, if < 10% of the total bilirubin.

Laboratory Values Chemistries

PARAMETER	GRADE 1 MILD	GRADE 2 MODERATE	GRADE 3 SEVERE	GRADE 4 POTENTIALLY LIFE-THREATENING
**Acidosis**	NA	pH ≥ 7.3 to < LLN	pH < 7.3 without life-threatening consequences	pH < 7.3 with life-threatening consequences
**Albumin, Low** (g/dL; *g/L*)	3.0 to < LLN<br>*30 to < LLN*	≥ 2.0 to < 3.0<br>*≥ 20 to < 30*	< 2.0<br>*< 20*	NA
**Alkaline Phosphatase, High**	1.25 to < 2.5 x ULN	2.5 to < 5.0 x ULN	5.0 to < 10.0 x ULN	≥ 10.0 x ULN
**Alkalosis**	NA	pH > ULN to ≤ 7.5	pH > 7.5 without life-threatening consequences	pH > 7.5 with life-threatening consequences
**ALT or SGPT, High**<br>*Report only one*	1.25 to < 2.5 x ULN	2.5 to < 5.0 x ULN	5.0 to < 10.0 x ULN	≥ 10.0 x ULN
**Amylase (Pancreatic) or Amylase (Total), High**<br>*Report only one*	1.1 to < 1.5 x ULN	1.5 to < 3.0 x ULN	3.0 to < 5.0 x ULN	≥ 5.0 x ULN
**AST or SGOT, High**<br>*Report only one*	1.25 to < 2.5 x ULN	2.5 to < 5.0 x ULN	5.0 to < 10.0 x ULN	≥ 10.0 x ULN
**Bicarbonate, Low** (mEq/L; *mmol/L*)	16.0 to < LLN<br>*16.0 to < LLN*	11.0 to < 16.0<br>*11.0 to < 16.0*	8.0 to < 11.0<br>*8.0 to < 11.0*	< 8.0<br>*< 8.0*
**Bilirubin**<br>***Direct Bilirubin[14], High***<br>*> 28 days of age*	NA	NA	> ULN	> ULN with life-threatening consequences (e.g., signs and symptoms of liver failure)
≤ 28 days of age	ULN to ≤ 1 mg/dL	> 1 to ≤ 1.5 mg/dL	> 1.5 to ≤ 2 mg/dL	> 2 mg/dL
Total Bilirubin, High<br>*> 28 days of age*	1.1 to < 1.6 x ULN	1.6 to < 2.6 x ULN	2.6 to < 5.0 x ULN	≥ 5.0 x ULN
≤ 28 days of age	See Appendix A. Total Bilirubin for Term and Preterm Neonates	See Appendix A. Total Bilirubin for Term and Preterm Neonates	See Appendix A. Total Bilirubin for Term and Preterm Neonates	See Appendix A. Total Bilirubin for Term and Preterm Neonates
**Calcium, High** (mg/dL; *mmol/L*)<br>*≥ 7 days of age*	10.6 to < 11.5<br>*2.65 to < 2.88*	11.5 to < 12.5<br>*2.88 to < 3.13*	12.5 to < 13.5<br>*3.13 to < 3.38*	≥ 13.5<br>*≥ 3.38*
< 7 days of age	11.5 to < 12.4<br>*2.88 to < 3.10*	12.4 to < 12.9<br>*3.10 to < 3.23*	12.9 to < 13.5<br>*3.23 to < 3.38*	≥ 13.5<br>*≥ 3.38*

CHEMISTRIES

PARAMETER	GRADE 1 MILD	GRADE 2 MODERATE	GRADE 3 SEVERE	GRADE 4 POTENTIALLY LIFE-THREATENING
**Calcium (Ionized), High** (mg/dL; *mmol/L*)	> ULN to < 6.0<br>*> ULN to < 1.5*	6.0 to < 6.4<br>*1.5 to < 1.6*	6.4 to < 7.2<br>*1.6 to < 1.8*	≥ 7.2<br>*≥ 1.8*
**Calcium, Low** (mg/dL; *mmol/L*)<br>*≥ 7 days of age*	7.8 to < 8.4<br>*1.95* to *< 2.10*	7.0 to < 7.8<br>*1.75* to *< 1.95*	6.1 to < 7.0<br>*1.53* to *< 1.75*	< 6.1<br>*< 1.53*
< 7 days of age	6.5 to < 7.5<br>*1.63* to *< 1.88*	6.0 to < 6.5<br>*1.50* to *< 1.63*	5.50 to < 6.0<br>*1.38* to *< 1.50*	< 5.50<br>*< 1.38*
**Calcium (Ionized), Low** (mg/dL; *mmol/L*)	< LLN to 4.0<br>*< LLN to 1.0*	3.6 to < 4.0<br>*0.9 to < 1.0*	3.2 to < 3.6<br>*0.8 to < 0.9*	< 3.2<br>*< 0.8*
**Cardiac Troponin I, High**	NA	NA	NA	Levels consistent with myocardial infarction or unstable angina as defined by the local laboratory
**Creatine Kinase, High**	3 to < 6 x ULN	6 to < 10 x ULN	10 to < 20 x ULN	≥ 20 x ULN
**Creatinine, High**	1.1 to 1.3 x ULN	> 1.3 to 1.8 x ULN OR Increase of > 0.3 mg/dL above baseline	> 1.8 to < 3.5 x ULN OR Increase of 1.5 to < 2.0 x above baseline	≥ 3.5 x ULN OR Increase of ≥ 2.0 x above baseline
**Creatinine Clearance**[15] **or eGFR, Low**<br>*Report only one*	NA	< 90 to 60 ml/min or ml/min/1.73 m^2 OR 10 to < 30% decrease from baseline	< 60 to 30 ml/min or ml/min/1.73 m^2 OR ≥ 30 to < 50% decrease from baseline	< 30 ml/min or ml/min/1.73 m2 OR ≥ 50% decrease from baseline or dialysis needed
**Glucose** (mg/dL; *mmol/L*)<br>***Fasting, High***	110 to 125<br>*6.11 to < 6.95*	> 125 to 250<br>*6.95 to < 13.89*	> 250 to 500<br>*13.89 to < 27.75*	> 500<br>*≥ 27.75*
Nonfasting, High	116 to 160<br>*6.44 to < 8.89*	> 160 to 250<br>*8.89 to < 13.89*	> 250 to 500<br>*13.89 to < 27.75*	> 500<br>*≥ 27.75*
**Glucose, Low** (mg/dL; *mmol/L*)<br>*≥ 1 month of age*	55 to 64	40 to < 55	30 to < 40	< 30
< 1 month of age	50 to 54<br>*2.78 to 3.00*	40 to < 50<br>*2.22 to < 2.78*	30 to < 40<br>*1.67 to < 2.22*	< 30<br>*< 1.67*
**Lactate, High**	ULN to < 2.0 x ULN without acidosis	≥ 2.0 x ULN without acidosis	Increased lactate with pH < 7.3 without life-threatening consequences	Increased lactate with pH < 7.3 with life-threatening consequences

15 Use the applicable formula (i.e., Cockroft-Gault in mL/min or Schwatrz in mL/min/1.73m^2).

CHEMISTRIES

PARAMETER	GRADE 1<br>MILD	GRADE 2<br>MODERATE	GRADE 3<br>SEVERE	GRADE 4<br>POTENTIALLY LIFE-THREATENING
**Lipase, High**	1.1 to < 1.5 x ULN	1.5 to < 3.0 x ULN	3.0 to < 5.0 x ULN	≥ 5.0 x ULN
**Lipid Disorders**<br>(mg/dL; *mmol/L*)<br>***Cholesterol, Fasting, High***<br>*≥18 years of age*	200 to < 240<br>*5.18 to < 6.19*	240 to < 300<br>*6.19 to < 7.77*	≥ 300<br>*≥ 7.77*	NA
< 18 years of age	170 to < 200<br>*4.40 to < 5.15*	200 to < 300<br>*5.15 to < 7.77*	≥ 300<br>*≥ 7.77*	NA
LDL, Fasting, High<br>*≥18 years of age*	130 to < 160<br>*3.37 to < 4.12*	160 to < 190<br>*4.12 to < 4.90*	≥ 190<br>*≥ 4.90*	NA
> 2 to < 18 years of age	110 to < 130<br>*2.85 to < 3.34*	130 to < 190<br>*3.34 to < 4.90*	≥ 190<br>*≥ 4.90*	NA
Triglycerides, Fasting, High	150 to 300<br>*1.71 to 3.42*	>300 to 500<br>*>3.42 to 5.7*	>500 to < 1,000<br>*>5.7 to 11.4*	> 1,000<br>*> 11.4*
**Magnesium[16], Low**<br>(mEq/L; *mmol/L*)	1.2 to < 1.4<br>*0.60 to < 0.70*	0.9 to < 1.2<br>*0.45 to < 0.60*	0.6 to < 0.9<br>*0.30 to < 0.45*	< 0.6<br>*< 0.30*
**Phosphate, Low**<br>(mg/dL;<br>*> 14 years of age*	2.0 to < LLN<br>*0.81 to < LLN*	1.4 to < 2.0<br>*0.65 to < 0.81*	1.0 to < 1.4<br>*0.32 to < 0.65*	< 1.0<br>*< 0.32*
1 to 14 years of age	3.0 to < 3.5<br>*0.97 to < 1.13*	2.5 to < 3.0<br>*0.81 to < 0.97*	1.5 to < 2.5<br>*0.48 to < 0.81*	< 1.5<br>*< 0.48*
< 1 year of age	3.5 to < 4.5<br>*1.13 to < 1.45*	2.5 to < 3.5<br>*0.81 to < 1.13*	1.5 to < 2.5<br>*0.48 to < 0.81*	< 1.5<br>*< 0.48*
**Potassium, High**<br>(mEq/L; *mmol/L*)	5.6 to < 6.0<br>*5.6 to < 6.0*	6.0 to < 6.5<br>*6.0 to < 6.5*	6.5 to < 7.0<br>*6.5 to < 7.0*	≥ 7.0<br>*≥ 7.0*
**Potassium, Low**<br>(mEq/L; *mmol/L*)	3.0 to < 3.4<br>*3.0 to < 3.4*	2.5 to < 3.0<br>*2.5 to < 3.0*	2.0 to < 2.5<br>*2.0 to < 2.5*	< 2.0<br>*< 2.0*
**Sodium, High**<br>(mEq/L; *mmol/L*)	146 to < 150<br>*146 to < 150*	150 to < 154<br>*150 to < 154*	154 to < 160<br>*154 to < 160*	≥ 160<br>*≥ 160*
**Sodium, Low**<br>(mEq/L; *mmol/L*)	130 to < 135<br>*130 to < 135*	125 to < 130<br>*125 to < 135*	121 to < 125<br>*121 to < 125*	≤ 120<br>*≤ 120*
**Uric Acid, High**<br>(mg/dL; *mmol/L*)	7.5 to < 10.0<br>*0.45 to < 0.59*	10.0 to < 12.0<br>*0.59 to < 0.71*	12.0 to < 15.0<br>*0.71 to < 0.89*	≥ 15.0<br>*≥ 0.89*

[16] To convert a magnesium value from mg/dL to mmol/L, laboratories should multiply by 0.4114.

HEMATOLOGY

PARAMETER	GRADE 1 MILD	GRADE 2 MODERATE	GRADE 3 SEVERE	GRADE 4 POTENTIALLY LIFE-THREATENING
**Absolute CD4+ Count, Low** (cell/mm^3; *cells/L*)<br>*> 5 years of age*<br>*(not HIV infected)*	300 to < 400<br>*300 to < 400*	200 to < 300<br>*200 to < 300*	100 to < 200<br>*100 to < 200*	< 100<br>*< 100*
**Absolute Lymphocyte Count, Low** (cell/mm^3; *cells/L*)<br>*> 5 years of age*<br>*(not HIV infected)*	600 to < 650<br>*0.600 x 10^9 to < 0.650 x 10^9*	500 to < 600<br>*0.500 x 10^9 to < 0.600 x 10^9*	350 to < 500<br>*0.350 x 10^9 to < 0.500 x 10^9*	< 350<br>*< 0.350 x 10^9*
**Absolute Neutrophil Count (ANC), Low** (cells/mm^3; *cells/L*)<br>*> 7 days of age*	800 to 1,000<br>*0.800 x 10^9 to 1.000 x 10^9*	600 to 799<br>*0.600 x 10^9 to 0.799 x 10^9*	400 to 599<br>*0.400 x 10^9 to 0.599 x 10^9*	< 400<br>*< 0.400 x 10^9*
2 to 7 days of age	1,250 to 1,500<br>*1.250 x 10^9 to 1.500 x 10^9*	1,000 to 1,249<br>*1.000 x 10^9 to 1.249 x 10^9*	750 to 999<br>*0.750 x 10^9 to 0.999 x 10^9*	< 750<br>*< 0.750 x 10^9*
≤ 1 day of age	4,000 to 5,000<br>*4.000 x 10^9 to 5.000 x 10_9*	3,000 to 3,999<br>*3.000 x 10^9 to 3.999 x 10^9*	1,500 to 2,999<br>*1.500 x 10^9 to 2.999 x 10^9*	< 1,500<br>*< 1.500 x 10^9*
**Fibrinogen, Decreased** (mg/dL; *g/L*)	100 to < 200<br>*1.00 to < 2.00*<br>OR<br>0.75 to < 1.00 x LLN	75 to < 100<br>*0.75 to < 1.00*<br>OR<br>≥ 0.50 to < 0.75 x LLN	50 to < 75<br>*0.50 to < 0.75*<br>OR<br>0.25 to < 0.50 x LLN	< 50<br>*< 0.50*<br>OR<br>< 0.25 x LLN<br>OR Associated with gross bleeding
**Hemoglobin[17], Low** (g/dL; *mmol/L*)[18]<br>*≥ 13 years of age*<br>*(male only)*	10.0 to 10.9<br>*6.19 to 6.76*	9.0 to < 10.0<br>*5.57 to < 6.19*	7.0 to < 9.0<br>*4.34 to < 5.57*	< 7.0<br>*< 4.34*
≥ 13 years of age<br>*(female only)*	9.5 to 10.4<br>*5.88 to 6.48*	8.5 to < 9.5<br>*5.25 to < 5.88*	6.5 to < 8.5<br>*4.03 to < 5.25*	< 6.5<br>*< 4.03*

[17] Male and female sex are defined as sex at birth.
[18] The conversion factor used to convert g/dL to mmol/L is 0.6206 and is the most commonly used conversion factor. For grading hemoglobin results obtained by an analytic method with a conversion factor other than 0.6206, the result must be converted to g/dL using the appropriate conversion factor for the particular laboratory.

HEMATOLOGY

PARAMETER	GRADE 1 MILD	GRADE 2 MODERATE	GRADE 3 SEVERE	GRADE 4 POTENTIALLY LIFE-THREATENING
57 days of age to < 13 years of age (male and female)	9.5 to 10.4<br>*5.88 to 6.48*	8.5 to < 9.5<br>*5.25 to < 5.88*	6.5 to < 8.5<br>*4.03 to < 5.25*	< 6.5<br>*< 4.03*
36 to 56 days of age (male and female)	8.5 to 9.6<br>*5.26 to 5.99*	7.0 to < 8.5<br>*4.32 to < 5.26*	6.0 to < 7.0<br>*3.72 to < 4.32*	< 6.0<br>*< 3.72*
22 to 35 days of age (male and female)	9.5 to 11.0<br>*5.88 to 6.86*	8.0 to < 9.5<br>*4.94 to < 5.88*	6.7 to < 8.0<br>*4.15 to < 4.94*	< 6.7<br>*< 4.15*
8 to ≤ 21 days of age (male and female)	11.0 to 13.0<br>*6.81 to 8.10*	9.0 to < 11.0<br>*5.57 to < 6.81*	8.0 to < 9.0<br>*4.96 to < 5.57*	< 8.0<br>*< 4.96*
≤ 7 days of age (male and female)	13.0 to 14.0<br>*8.05 to 8.72*	10.0 to < 13.0<br>*6.19 to < 8.05*	9.0 to < 10.0<br>*5.59 to < 6.19*	< 9.0<br>*< 5.59*
**INR, High** (not on anticoagulation therapy)	1.1 to < 1.5 x ULN	1.5 to < 2.0 x ULN	2.0 to < 3.0 x ULN	≥ 3.0 x ULN
**Methemoglobin** (% hemoglobin)	5.0 to < 10.0%	10.0 to < 15.0%	15.0 to < 20.0%	≥ 20.0%
**PTT, High** (not on anticoagulation therapy)	1.1 to < 1.66 x ULN	1.66 to < 2.33 x ULN	2.33 to < 3.00 x ULN	≥ 3.00 x ULN
**Platelets, Decreased** (cells/mm^3; *cells/L*)	100,000 to < 124,999<br>*100.000 x 10^9 to < 124.999 x 10^9*	50,000 to < 100,000<br>*50.000 x 10^9 to < 100.000 x 10^9*	25,000 to < 50,000<br>*25.000 x 10^9 to < 50.000 x 10^9*	< 25,000<br>*< 25.000 x 10^9*
**PT, High** (not on anticoagulation therapy	1.1 to < 1.25 x ULN	1.25 to < 1.50 x ULN	1.50 to < 3.00 x ULN	≥ 3.00 x ULN
**WBC, Decreased** (cells/mm^3; *cells/L*)<br>*> 7 days of age*	2,000 to 2,499<br>*2.000 x 10^9 to 2.499 x 10^9*	1,500 to 1,999<br>*1.500 x 10^9 to 1.999 x 10^9*	1,000 to 1,499<br>*1.000 x 10^9 to 1.499 x 10^9*	< 1,000<br>*< 1.000 x 10^9*
≤ 7 days of age	5,500 to 6,999<br>*5.500 x 10^9 to 6.999 x 10^9*	4,000 to 5,499<br>*4.000 x 10^9 to 5.499 x 10^9*	2,500 to 3,999<br>*2.500 x 10^9 to 3.999 x 10^9*	< 2,500<br>*< 2.500 x 10^9*

URINALYSIS

PARAMETER	GRADE 1 MILD	GRADE 2 MODERATE	GRADE 3 SEVERE	GRADE 4 POTENTIALLY LIFE-THREATENING
**Glycosuria** (random collection tested by dipstick)	Trace to 1+ or ≤ 250 mg	2+ or > 250 to < 500 mg	> 2+ or > 500 mg	NA
**Hematuria** (not to be reported based on dipstick findings or on blood believed to be of menstrual origin)	6 to < 10 RBCs per high power field	≥ 10 RBCs per high power field	Gross, with or without clots OR With RBC casts OR Intervention indicated	Life-threatening consequences
**Proteinuria** (random collection tested by dipstick)	1+	2+	3+ or higher	NA

International AIDS Vaccine Initiative

DOCUMENT NUMBER:

DOCUMENT TITLE:

DOCUMENT NOTES:

Document Information

Revision:	Vault:
Status:	Document Type:

Date Information

Effective Date:	Expiration Date:
Release Date:	Next Review Date:

Control Information

Author:	Owner:
Previous Number:	Change Number:

Signature Manifest

Document Number: TMF-02-0248 **Revision:** 2

Title: Protocol PGT121/PGDM1400

All dates and times are in Eastern Time Zone.

T002 Protocol Amendment

Change Request Approval

Name/Signature	Title	Date	Meaning/Reason
Carl Verlinde (CVERLINDE)			
Dani Vooijs (DVOOIJS)			
Jeniffer Kigera (JKIGERA)			
Lisa Sunner (LSUNNER)			
Harriet Park (HPARK)	Director Clincal Operations	27 Jul 2017, 01:55:16 PM	Approved

CMO Approval

Name/Signature	Title	Date	Meaning/Reason
Frances Priddy (FPRIDDY)	Chief Medical Officer	14 Aug 2017, 08:14:49 PM	Approved

QA Final Release

Name/Signature	Title	Date	Meaning/Reason
Jeniffer Kigera (JKIGERA)			
Lisa Sunner (LSUNNER)			
Carl Verlinde (CVERLINDE)			
Dani Vooijs (DVOOIJS)			
Harriet Park (HPARK)	Director Clincal Operations	14 Aug 2017, 08:49:38 PM	Approved

Notify

Name/Signature	Title	Date	Meaning/Reason
Lisa Sunner (LSUNNER)		14 Aug 2017, 08:49:39 PM	Email Sent

CONFIDENTIAL

**Protocol Title:**	A Phase 1 Randomized Placebo-controlled Clinical Trial of the Safety, Pharmacokinetics and Antiviral Activity of PGDM1400 and PGT121 and VRC07-523LS Monoclonal Antibodies in HIV-uninfected and HIV-infected Adults
**Protocol Number:**	IAVI T002
**Regulatory Investigational Product Number:**	IND#134270
**ClinicalTrials.gov Registry Number:**	NCT03205917
**Phase:**	Phase 1
**Sponsor:**	International AIDS Vaccine Initiative (IAVI)<br>125 Broad Street, 9th Floor<br>New York, New York 10004<br>USA
**Sponsor Status**	Not for-Profit Organization
**Date of Protocol Version:**	01 APRIL 2019<br>4.0<br><br>07 AUGUST 2017<br>3.0<br><br>29 JUNE 2017<br>2.0<br><br>12 MAY 2017<br>1.0

THE CONFIDENTIAL INFORMATION IN THIS DOCUMENT IS PROVIDED TO YOU AS AN INVESTIGATOR, POTENTIAL INVESTIGATOR, OR CONSULTANT, FOR REVIEW BY YOU, YOUR STAFF, AND APPLICABLE INSTITUTIONAL REVIEW BOARDS (IRBS) AND/OR INDEPENDENT ETHICS COMMITTEES (IECS). IT IS UNDERSTOOD THAT THE INFORMATION WILL NOT BE DISCLOSED TO OTHERS, EXCEPT TO THE EXTENT NECESSARY TO OBTAIN ETHICAL AND REGULATORY APPROVAL FROM THE RESPECTIVE COMMITTEE'S AGENCIES AND INFORMED CONSENT FROM THOSE PERSONS TO WHOM THE INVESTIGATIONAL PRODUCT MAY BE ADMINISTERED.

TMF-03635 | 4.0 | Status:Approved | 16 Apr 2019
This copy of the document was retrieved from the system by Lisa Sunner on 24 Apr 2019

PROTOCOL SYNOPSIS

**TITLE**	A Phase 1 Randomized Placebo-controlled Clinical Trial of the Safety, Pharmacokinetics and Antiviral Activity of PGDM1400, PGT121 and VRC07-523LS Monoclonal Antibodies (mAb) in HIV-uninfected and HIV-infected Adults
**PROTOCOL NUMBER**	IAVI T002
**CLINICAL TRIAL PHASE**	Phase 1
**IND SPONSOR**	International AIDS Vaccine Initiative (IAVI)<br>125 Broad Street, 9th Floor<br>New York, New York 10004,<br>USA
**SPONSOR STATUS**	Not for Profit Organization
**SAMPLE SIZE**	30-66
**STUDY POPULATION**	1. HIV-uninfected males or females aged 18-50 years old who are willing to maintain low risk behavior for HIV infection; principal exclusion criteria include confirmed HIV-infection, pregnancy or lactation, significant acute or chronic disease and clinically significant laboratory abnormalities (Groups 1 and 2, see below for full inclusion and exclusion criteria).<br>2. HIV-infected males or females aged 18-65 years old, not on antiretroviral therapy (ART) with HIV-1 viral load between 1000 and 100,000 copies/ml, CD4 cell count ≥ 300 cells/µl; principal exclusion criteria include significant acute or chronic medical condition other than HIV infection, and clinically significant laboratory abnormalities (Group 3, see below for full inclusion and exclusion criteria).
**STUDY DESIGN**	Double blind, randomized, placebo-controlled study for Groups 1 and 2. Open label study for Group 3. Groups 1 and 2, single intravenous (IV) infusion of PGDM1400 mAb alone at 3, 10 or 30 mg/kg or placebo, or a combination of PGDM1400 mAb and PGT121 mAb, each at 3, 10 or 30 mg/kg or placebo. Group 3, a combination of PGDM1400 mAb and PGT121 mAb each at the MTD from Part 1 or PGDM1400 mAb and PGT121 mAb and VRC07-523LS mAb each at 20 mg/kg. See study design table below for details.
**STUDY DURATION**	Up to 32 weeks per participant, screening up to 42 (HIV-infected) or up to 56 (HIV-uninfected) days before single IV infusion of investigational product on day 0, and 24 weeks of follow up.

This copy of the document was retrieved from the system by Lisa Sunner on 24 Apr 2019

**INVESTIGATIONAL PRODUCT DESCRIPTION**	**PGDM1400 mAb**: PGDM1400 mAb is a recombinant, fully human monoclonal antibody (mAb) of the IgG1 isotype that binds to the HIV envelope. The concentration of the product is 50 mg/ml. Vials will contain 6 ml of product @ 50 mg/ml = 300 mg.<br>**PGT121 mAb**: PGT121 mAb is a recombinant, fully human monoclonal antibody (mAb) of the IgG1 isotype that binds to the HIV envelope. The concentration of the product is 50 mg/ml. Vials will contain 6 ml of product @ 50 mg/ml = 300 mg.<br>**VRC07-523LS:** VRC07-523LS is a recombinant, fully human monoclonal antibody of the IgG1 isotype that binds to the HIV envelope. The concentration of the product is 100 mg/ml. Vials will contain 2 or 6 ml of product at 100 mg/ml = 200 or 600 mg.<br>**Placebo:** 0.9% Sodium Chloride for injection, USP
**OBJECTIVES**	**Primary Objectives**<br>• To evaluate the safety and tolerability of IV infusion of PGDM1400 mAb alone, and a combination of PGDM1400 mAb and PGT121 mAb, in HIV-uninfected adults and HIV-infected adults, and a combination of PGDM1400 mAb and PGT121 mAb and VRC07-523LS mAb in HIV-infected adults<br>• To evaluate the pharmacokinetic (PK) profile of IV infusion of PGDM1400 mAb alone, and a combination of PGDM1400 mAb and PGT121 mAb, in HIV-uninfected adults and HIV-infected adults, and a combination of PGDM1400 mAb and PGT121 mAb and VRC07-523LS mAb in HIV-infected adults<br>• To evaluate the antiviral activity of IV infusion of a combination of PGDM1400 mAb and PGT121 mAb, and a combination of PGDM1400 mAb and PGT121 mAb and VRC07-523LS mAb, in HIV-infected adults not on ART<br><br>**Secondary Objectives**<br>• To determine if PGDM1400 mAb alone, and in combination with PGT121 mAb, and in combination with PGT121 mAb and VRC07-523LS mAb, induces anti-PGDM1400 and/or anti-PGT121 and/or VRC07-523LS antibodies<br>• To determine the effect of PGDM1400 mAb in combination with PGT121 mAb, and PGDM1400 in combination with PGT121 mAb and VRC07-523LS mAb, on CD4+ T cell counts in HIV-infected adults<br>• To determine the effect of PGDM1400 mAb in combination with PGT121 mAb, and PGDM1400 in combination with PGT121 mAb and VRC07-523LS mAb, on viral escape in viremic HIV-infected adults not on ART

This copy of the document was retrieved from the system by Lisa Sunner on 24 Apr 2019

	**Exploratory Objectives**<br>• To determine if PGDM1400 ± PGT121 ± VRC07-523LS mAb has any impact on the host immune responses (i.e., HIV-specific cellular and humoral immune response).<br>• To determine the effect of PGDM1400 + PGT121 ± VRC07-523LS mAb on the size of the latent HIV reservoir in HIV-infected adults.<br>• To determine PGDM1400 mAb ± PGT121 ± VRC07-523LS mAb levels in mucosal secretions in HIV-uninfected and HIV-infected adults.<br>• To measure in vitro neutralization of HIV isolates with participant's serum post PGDM1400 ± PGT121 ± VRC07-523LS mAb IV infusion
**ENDPOINTS**	**Primary Endpoints**<br>*Safety and Tolerability*<br>1. Proportion of participants with moderate or greater reactogenicity (e.g., solicited adverse events) for 3 days following IV infusion of PGDM1400 mAb alone, and a combination of PGDM1400 mAb and PGT121 mAb, and a combination of PGDM1400 mAb and PGT121 mAb and VRC07-523LS mAb<br>2. Proportion of participants with adverse events (AEs), including safety laboratory (biochemical, hematological) parameters, during the first 56 days following IV infusion of PGDM1400 mAb alone and a combination of PGDM1400 mAb and PGT121 mAb, and a combination of PGDM1400 mAb and PGT121 mAb and VRC07-523LS mAb, that are moderate or greater, and/or related to PGDM1400 mAb or PGT121 mAb or VRC07-523LS mAb<br>3. Proportion of participants with serious adverse events (SAEs) throughout the study period following IV infusion of PGDM1400 mAb alone and a combination of PGDM1400 mAb and PGT121 mAb, and a combination of PGDM1400 mAb and PGT121 mAb and VRC07-523LS mAb, that are related to PGDM1400 mAb or PGT121 mAb or VRC07-523LS mAb<br><br>*Pharmacokinetics*<br>Pharmacokinetics following IV infusion of PGDM1400 mAb alone or a combination of PGDM1400 mAb and PGT121 mAb in HIV-uninfected and HIV-infected adults, or a combination of PGDM1400 mAb and PGT121 mAb and VRC07-523LS mAb in HIV-infected adults:<br>• Elimination half-life (t1/2)<br>• Clearance (CL/F)<br>• Volume of distribution (Vz/F)<br>• Area under the concentration decay curve (AUC)

This copy of the document was retrieved from the system by Lisa Sunner on 24 Apr 2019

- Impact of viral load and/or ART on PGDM1400 mAb and PGT121 mAb and VRC07-523LS mAb disposition (elimination half-life (t1/2), clearance (CL/F), volume of distribution (Vz/F), total exposure)

Antiviral activity
Antiviral activity following IV infusion of PGDM1400 mAb in combination with PGT121 mAb, or PGDM1400 mAb in combination with PGT121 mAb and VRC07-523LS mAb, in viremic HIV-infected adults not on ART:

1. Change in plasma HIV-1 RNA levels from baseline (mean of pre-entry and entry values)

Secondary Endpoints
Anti-PGDM1400, anti-PGT121 and anti-VRC07-523LS antibodies

1. Serum anti-PGDM1400 antibody titers
2. Serum anti-PGT121 antibody titers
3. Serum anti-VRC07-523LS antibody titers

CD4+ T cell count
We will calculate the following endpoint to determine if IV infusion of PGDM1400 mAb in combination with PGT121 mAb, or PGDM1400 mAb in combination with PGT121 mAb and VRC07-523LS mAb, has any impact on CD4+ T cell counts in HIV-infected adults:

1. Change in CD4+ T cell count and frequency compared to baseline as measured by single platform flow cytometry.

HIV genotyping/phenotyping of circulating virus for evaluation of PGDM1400 mAb and PGT121 mAb and/or VRC07-523LS mAb -induced escape mutations
We will compare plasma virus genotype and phenotypic activity before and after IV infusion of PGDM1400 mAb in combination with PGT121 mAb, or PGDM1400 mAb in combination with PGT121 mAb and VRC07-523LS mAb, to determine if PGDM1400 mAb and PGT121 mAb and/or VRC07-523LS mAb induced viral escape mutations have developed in viremic HIV-infected adults not on ART.

1. Genotypic analysis: Development of sequence variations in epitopes known to result in reduced PGDM1400 mAb and PGT121 mAb and/or VRC07-523LS mAb neutralization susceptibility or known to cause resistance to antiretroviral drugs.
2. Phenotypic analysis: Changes in viral susceptibility to PGDM1400 mAb and PGT121 mAb and/or VRC07-523LS mAb neutralization.

Exploratory Endpoints
Additional assessments may include but are not limited to

	the following: HIV-specific IgG/IgA binding responses by ELISA, HIV-specific cellular immune responses by ELISPOT, HIV-specific antibody function by ADCC, ADCP, and ADCVI assays, PGDM1400 mAb and/or PGT121 mAb and/or VRC07-523LS mAb levels in mucosal secretions, changes in total HIV-1 DNA and 2-long terminal repeat (LTR) circular HIV-1 DNA in resting or total CD4 T cells and in vitro neutralization of HIV isolates with participant's serum post IV infusion of PGDM1400 mAb + PGT121 mAb ± VRC07-523LS mAb.

This copy of the document was retrieved from the system by Lisa Sunner on 24 Apr 2019

STUDY DESIGN TABLE

	Group	Participants	Sub-Group	Regimen	N	Dose (mg/kg)
Part 1 – MTD	1	HIV-uninfected participants	1A	PGDM1400/Placebo	3/1 (6/2 if DLT)	3 IV
			1B	PGDM1400/Placebo	3/1 (6/2 if DLT)	10 IV
			1C	PGDM1400/Placebo	3/1 (6/2 if DLT)	30 IV
				Total Group 1	9/3 = 12 (max 18/6 = 24 if DLT)	
	2	HIV-uninfected participants	2A	PGDM1400 + PGT121/Placebo	3/1 (6/2 if DLT)	3 + 3 IV
			2B	PGDM1400 + PGT121/Placebo	3/1 (6/2 if DLT)	10 + 10 IV
			2C	PGDM1400 + PGT121/Placebo	3/1 (6/2 if DLT)	30 + 30 IV
				Total Group 2	9/3 = 12 (max 18/6 = 24 if DLT)	
				Total Groups 1 and 2	18/6 = 24 (max 36/12 = 48 if DLT)	
**Safety Monitoring Committee**						
Part 2 – antiviral effect	3	HIV-infected off ART (VL $1x10^3$ – $1x10^5$ copies/ml)	3A	PGDM1400 + PGT121 + VRC07-523LS	3 (max 9)	20 + 20 + 20 IV
			3B	PGDM1400 + PGT121	3 (max 9)	MTD IV
				Total Group 3	6 (max 18)	
				Total entire study	30 (max 66)	

DLT, dose limiting toxicity; MTD, maximum tolerated dose

TMF-03635 | 4.0 | Status:Approved | 16 Apr 2019
This copy of the document was retrieved from the system by Lisa Sunner on 24 Apr 2019

**SAFETY MONITORING**	Safety will continually be monitored by the Investigators, the Sponsor's Medical Monitor and a Protocol Safety Review Team (PSRT). Safety data will be reviewed by an independent Safety Monitoring Committee (SMC).<br><br>-*Sentinel participants in Groups 1 and 2 and 3A*<br>For each dose Subgroup in Groups 1 and 2 and in Subgroup 3A, the 1st 2 participants will be sentinel participants for whom investigational product infusion will be separated by at least 24 hours contingent on review of safety information prior to infusion of the next sentinel participant. Both sentinel participants are observed for at least 24 hours before IV infusion of investigational product to the 3rd and subsequent participants.<br><br>-*Dose limiting toxicity in Groups 1 and 2*<br>Dose limiting toxicity (DLT) will be defined as 1) any Grade 3 or greater adverse event considered possibly, probably or definitely related to investigational product or 2) any Grade 3 or greater reactogenicity or 3) any SAE considered possibly, probably or definitely related to investigational product.<br><br>-*Infusion related reactions*<br>Infusion related reactions, including cytokine release syndrome, will be graded using the NCI Common Terminology Criteria for Adverse Events (CTCAE) version 4.03 (June 14, 2010). All other adverse events will be graded using the Division of AIDS (DAIDS) Table for Grading the Severity of Adult and Pediatric Adverse Events version 2.0 (November 2014).
**DOSE ESCALATION IN GROUPS 1 AND 2**	Part 1 of this study is a dose-escalation trial in HIV-uninfected adults (Groups 1 and 2) to establish the MTD of PGDM1400 mAb and a combination of PGDM1400 mAb and PGT121 mAb.<br><br>-*PSRT review for dose escalation*<br>The Protocol Safety Review Team (PSRT) will review safety data through day 14 post-investigational product infusion for all participants in each dose Subgroup for PGDM1400 mAb alone (Group 1) prior to allowing enrolment of participants into the next higher dose Subgroup in Group 1 or the same dose level PGDM1400 mAb and PGT121 mAb combination dose Subgroup (Group 2).<br>o If no DLT occurs in the initial 4 participants of a dose Subgroup (e.g., 1A), the study can proceed with enrolment of the next dose Subgroup for PGDM1400 mAb alone (e.g., 1B), and the same dose level PGDM1400 mAb and PGT121 mAb combination dose Subgroup (e.g., 2A).<br>o If 1 DLT occurs in the initial 4 participants of a dose Subgroup (e.g., 1A), 4 additional participants will be enrolled in the same dose Subgroup.

TMF-03635 | 4.0 | Status:Approved | 16 Apr 2019
This copy of the document was retrieved from the system by Lisa Sunner on 24 Apr 2019

	▪ If no additional DLTs occur within 14 days of infusion in the 8 total participants, the study can proceed with enrolment of the next dose Subgroup for PGDM1400 mAb alone (e.g., 1B), and the same dose level PGDM1400 mAb and PGT121 mAb combination dose Subgroup (e.g., 2A).<br>▪ In Subgroups receiving PGDM1400 alone (1A, 1B, 1C), if 2 or more DLTs accumulate in a dose Subgroup (e.g., 1B) that are the same, similar, or in the same System Organ Class, infusion will be halted and the next lower dose level will be declared the maximum tolerated dose (MTD) within this Group (e.g., 3 mg/kg if the DLTs occurred in dose Subgroup 1B @ 10 mg/kg) , and the same dose level PGDM1400 mAb and PGT121 mAb combination dose Subgroup (2A in this example). Group 2 will not proceed with escalation beyond the MTDs of PGDM1400 alone and PGT121 alone.<br>▪ If no DLT occurs in the final dose Subgroup (1C) after 14 days of follow-up, the MTD for PGDM1400 alone will be the highest dose given (30mg/kg).<br>▪ In Subgroups receiving PGDM1400 and PGT121 (2A, 2B, 2C), if 2 or more DLTs occur in a dose Subgroup (e.g., 2B) that are the same, similar, or in the same System Organ Class, infusion will be halted and the next lower dose level will be declared the MTD for the combination of PGDM1400 and PGT121.<br>▪ If no DLT occurs in the final dose Subgroup (2C) after 14 days of follow-up , MTD for PGDM1400 and PGT121 will be the highest dose given (30mg/kg each).<br><br>-*SMC review to determine MTD of PGDM1400 alone*<br>Following IV infusion of investigational product in the last participant in Group 1, an independent Safety Monitoring Committee (SMC) will review safety data through day 14 post-investigational product infusion for all participants to confirm MTD of PGDM1400 alone.<br><br>-*SMC review to determine MTD of the combination of PGDM1400 and PGT121*<br>Following IV infusion of investigational product in the last participant in Group 2, the SMC will review safety data through day 14 post-investigational product infusion for all participants to confirm MTD of the combination of PGDM1400 and PGT121, and determine whether Group 3B can initiate enrollment.
**ANTIVIRAL ACTIVITY IN GROUP 3**	Part 2 of this study will establish the antiviral effect of PGDM1400 mAb in combination with PGT121 mAb and the effect of PGDM1400 mAb in combination with PGT121 mAb and VRC07-523LS mAb, in HIV infected adults not on ART (Group 3).

TMF-03635 | 4.0 | Status:Approved | 16 Apr 2019
This copy of the document was retrieved from the system by Lisa Sunner on 24 Apr 2019

	- *MTD determines dose in Subgroup 3B*<br>Subgroup 3B will receive PGDM1400 mAb + PGT121 mAb at a dose that combines the MTD for PGDM1400 mAb and the MTD for PGT121 mAb, respectively.
**PAUSE RULES**	The study will be paused for a safety review by the investigators and the independent SMC if:<br>1. 1 or more participants experience a Serious Adverse Event that is judged possibly, probably or definitely related to the investigational product.<br>2. There is a participant death regardless of relationship to the investigational product.<br>3. If 2 or more participants experience Grade 3 adverse events in the same System Organ Class that are considered possibly, probably, or definitely related to investigational product.<br>4. Any Grade 4 adverse event that is considered possibly, probably or definitely related to investigational product.
**EVALUATION FOR INTERCURRENT HIV INFECTION:**	Participants in Groups 1 and 2 (HIV-uninfected) will be tested for HIV according to the Schedule of Procedures. Test results will be interpreted according to a pre-determined diagnostic algorithm. HIV testing at additional time points may be performed upon the request of the participant and Principal Investigator or designee as medical or social circumstances warrant.
**INCLUSION CRITERIA**	**Inclusion criteria for all participants:**<br>1. Willing to comply with the requirements of the protocol and available for follow-up for the planned duration of the study.<br>2. In the opinion of the Principal Investigator or designee and based on Assessment of Informed Consent Understanding results, has understood the information provided and potential impact and/or risks linked to IV infusion and participation in the trial; written informed consent will be obtained from the participant before any study-related procedures are performed.<br>3. All heterosexually active female participants must commit to use an effective method of contraception for 3 months following investigational product administration, including:<br>a. Condoms (male or female) with or without spermicide<br>b. Diaphragm or cervical cap with spermicide<br>c. Intrauterine device, or contraceptive implant<br>d. Hormonal contraception<br>e. Successful vasectomy in the male partner (considered successful if a woman reports that a male partner has [1] documentation of azoospermia by microscopy (< 1 year ago), or [2] a vasectomy more than 2 years ago with no resultant pregnancy despite sexual activity post-vasectomy)<br>f. Not be of reproductive potential, such as having undergone hysterectomy, bilateral oophorectomy, or tubal ligation, postmenopausal (> 45 years of age with amenorrhea for at

This copy of the document was retrieved from the system by Lisa Sunner on 24 Apr 2019

least 2 years, or any age with amenorrhea for at least 6 months and a serum follicle stimulating hormone [FSH] level > 40 IU/L); surgically sterile: no additional contraception required.

Women, who are not heterosexually active at screening, must agree to utilize an effective method of contraception if they become hetero-sexually active, as outlined above.

4. All sexually active males, regardless of reproductive potential, must be willing to consistently use an effective method of contraception (such as consistent male condoms with male and/or female partners) from the day of investigational product administration until at least 3 months following investigational product administration to avoid exposure of partners to investigational product in ejaculate, and to prevent conception with female partners.
5. All female participants must be willing to undergo urine pregnancy tests at time points indicated in the Schedule of Procedures and must test negative prior to investigational product administration.
6. A female participant must agree not to donate eggs (ova, oocytes) for the purpose of assisted reproduction until 3 months after investigational product administration. A man must agree not to donate sperm until 3 months after investigational product administration.
7. Willing to forgo donations of blood and/or any other tissues, including bone marrow, during the study and, for those HIV-uninfected participants who test HIV-positive due to investigational product administration, until the anti-HIV antibody titers become undetectable.

Specific inclusion criteria for HIV-uninfected participants (Groups 1 and 2):

8. At least 18 years of age on the day of screening and has not reached his or her 51st birthday on the day of signing the Informed Consent Document.
9. Willing to undergo HIV testing, risk reduction counselling and receive HIV test results.
10. Low risk for HIV infection and willing to maintain low-risk behavior for the duration of the trial.
11. Healthy male or female, as assessed by a medical history, physical exam, and laboratory tests.

Specific inclusion criteria for HIV-infected participants (Group 3):

12. At least 18 years of age on the day of screening and has not reached his or her 66th birthday on the day of signing the Informed Consent Document.
13. Confirmed HIV-1 infection (HIV Ab+ or HIV RNA+) by documentation in the medical records or in-clinic HIV testing;
14. CD4 ≥ 300 cells/μl.
15. Not receiving cART, and (after appropriate counselling) willing to defer cART treatment for at least 56 days after

TMF-03635 | 4.0 | Status:Approved | 16 Apr 2019
This copy of the document was retrieved from the system by Lisa Sunner on 24 Apr 2019

	administration of investigational product.<br>16. HIV-1 viral load between 1000–100,000 copies/ml, confirmed at screening.<br>17. Under care of an HIV healthcare provider
**EXCLUSION CRITERIA**	**Exclusion criteria for all participants:**<br>1. Any clinically significant acute or chronic medical condition, other than HIV infection, that is considered progressive or in the opinion of the investigator makes the participant unsuitable for participation in the study.<br>2. If female, pregnant, lactating or planning a pregnancy during the period of screening through completion of the study.<br>3. In the past 6 months a history of alcohol or substance use, including marijuana, judged by the Investigator to potentially interfere with participant study compliance.<br>4. Bleeding disorder that was diagnosed by a physician (e.g., factor deficiency, coagulopathy or platelet disorder that requires special precautions). Note: A participant who states that he or she has easy bruising or bleeding, but does not have a formal diagnosis and has intramuscular injections and blood draws without any adverse experience, is eligible.<br>5. History of a splenectomy.<br>6. Receipt of live attenuated vaccine within the previous 30 days or planned receipt within 30 days after administration of investigational product; or receipt of other vaccine within the previous 14 days or planned receipt within 14 days after infusion with investigational product (exception is live attenuated influenza vaccine within 14 days).<br>7. Receipt of blood transfusion or blood-derived products within the previous 3 months.<br>8. Participation in another clinical trial of an investigational product currently, within the previous 3 months or expected participation during this study.<br>9. Prior receipt of an investigational HIV vaccine candidate, monoclonal antibody or polyclonal immunoglobulin (note: receipt of placebo in a previous HIV vaccine or monoclonal antibody trial will not exclude a participant from participation if documentation is available and the Medical Monitor gives approval).<br>10. History of severe local or systemic reactogenicity to injections or IV infusion (e.g., anaphylaxis, respiratory difficulties, angioedema);<br>11. Psychiatric condition that compromises safety of the participant and precludes compliance with the protocol. Specifically excluded are persons with psychoses within the past 3 years, ongoing risk for suicide, or history of suicide attempt or gesture within the past 3 years.<br>12. If, in the opinion of the Principal Investigator, it is not in the best interest of the participant to participate in the trial.<br>13. Seizure disorder: a participant who has had a seizure in the

This copy of the document was retrieved from the system by Lisa Sunner on 24 Apr 2019

last 3 years is excluded. (Not excluded: a participant with a history of seizures who has neither required medications nor had a seizure for 3 years.)

14. Body mass index ≥ 35 or ≤ 18.0.
15. Infectious disease: chronic hepatitis B infection (HbsAg), current hepatitis C infection (HCV Ab positive and HCV RNA positive) or interferon-alfa treatment for chronic hepatitis C infection in the past year, chlamydia, gonorrhea, or active syphilis.
16. A history of malignancy within the past 5 years (prior to screening) or ongoing malignancy;
17. Active, serious infections (other than HIV-1 infection) requiring parenteral antibiotic, antiviral or antifungal therapy within 30 days prior to enrollment.

Specific exclusion criteria for HIV-uninfected participants (Groups 1 and 2):

18. Confirmed HIV-1 or HIV-2 infection.
19. Any clinically relevant abnormality on history or examination including history of immunodeficiency or autoimmune disease; use of systemic corticosteroids, immunosuppressive, anticancer, or other medications considered significant by the investigator within the previous 6 months.

    The following exceptions are permitted and will not exclude study participation: use of corticosteroid nasal spray for rhinitis, topical corticosteroids for an acute uncomplicated dermatitis; or a short course (duration of 10 days or less, or a single injection) of corticosteroid for a non-chronic condition (based on investigator clinical judgment) at least 6 weeks prior to enrollment in this study.
20. Any of the following abnormal laboratory parameters listed below:

Hematology

- Hemoglobin < 10.5 g/dL in females; hemoglobin <11.0 g/dL in males
- Absolute Neutrophil Count (ANC): ≤ 1000/mm3
- Absolute Lymphocyte Count (ALC): < 650/mm3
- Platelets: < 125,000 mm3 or ≥ 550,000/mm3

Coagulation

- aPTT: >1.25 x ULN
- INR: ≥1.1 x ULN

Chemistry

- Sodium ≤ 135 mEq/L or ≥ 146 mEq/L
- Potassium ≤ 3.4 mEq/L or ≥ 5.6 mEq/L
- Creatinine ≥ 1.1 x ULN
- AST ≥ 1.25 x ULN
- ALT ≥ 1.25 x ULN
- Total bilirubin ≥ 1.25 x ULN
- Alkaline phosphatase ≥ 1.25 x ULN
- Albumin ≤ 3.0 g/dL or ≤ 30 g/L

This copy of the document was retrieved from the system by Lisa Sunner on 24 Apr 2019

- Creatine kinase ≥ 3.0 x ULN
- C-reactive protein > 10 mg/L
- C3 complement < 82 mg/dL
- C4 complement < 14 mg/dL

Urinalysis

Any of the following abnormal findings if consistent with clinically significant disease:

- Protein = greater than trace on dipstick confirmed by microscopic urinalysis outside institutional range.
- Blood = greater than trace on dipstick confirmed by >3 RBCs/hpf on microscopic urinalysis (not due to menses).

Specific exclusion criteria for HIV-infected participants who are not on ART (Group 3):

21. Any clinically relevant abnormality on history or examination including history of immunodeficiency or autoimmune disease, other than HIV; use of systemic corticosteroids, immunosuppressive, anticancer, or other medications considered significant by the investigator within the previous 6 months.

The following exceptions are permitted and will not exclude study participation: use of corticosteroid nasal spray for rhinitis, topical corticosteroids for an acute uncomplicated dermatitis; or a short course (duration of 10 days or less, or a single injection) of corticosteroid for a non-chronic condition (based on investigator clinical judgment) at least 6 weeks prior to enrollment in this study.

22. Any of the following abnormal laboratory parameters listed below:

Hematology

- Hemoglobin < 10.0 g/dL
- Absolute Neutrophil Count (ANC): < 1000 cells/mm3
- Platelets: < 100,000 cells/mm3

Coagulation

- aPTT: > 1.25 x ULN
- INR: ≥ 1.1 x ULN

Chemistry

- Estimated Glomerular filtration rate (GFR) < 80 mL/min according to the Cockcroft Gault formula for creatinine clearance
  - Male: (140 – age in years) x (wt in kg) = CLcr (mL/min) / 72 x (serum creatinine in mg/dL)
  - Female: (140 – age in years) x (wt in kg) x 0.85 = CLcr (mL/min) / 72 x (serum creatinine in mg/dL)
- AST ≥ 2.5 x ULN
- ALT ≥ 2.5 x ULN
- Total bilirubin ≥ 1.6 x ULN
- Alkaline phosphatase ≥ 5 x ULN

Urinalysis

Any of the following abnormal findings if consistent with

This copy of the document was retrieved from the system by Lisa Sunner on 24 Apr 2019

	clinically significant disease:<br>o Protein = greater than 1+ on dipstick confirmed by microscopic urinalysis outside institutional range.<br>o Blood = greater than 1+ on dipstick confirmed by > 10 RBCs/hpf on microscopic urinalysis (not due to menses).<br>o Leukocytes = greater than 1+ on dipstick confirmed by > 10 WBC/hpf on microscopic urinalysis.

This copy of the document was retrieved from the system by Lisa Sunner on 24 Apr 2019

TABLE OF CONTENTS

TABLE OF CONTENTS 16

ABBREVIATIONS 19

CONTACT INFORMATION 20

1.0 SIGNATURE PAGE 21

2.0 INTRODUCTION AND BACKGROUND INFORMATION 22

2.1 Study Rationale 22
2.2 Clinical experience with PGDM1400 and PGT121 and VRC07-523LS 24

3.0 STUDY OBJECTIVES 26

3.1 Primary Objectives 26
3.2 Secondary Objectives 26
3.3 Exploratory Objectives 26

4.0 STUDY ENDPOINTS 27

4.1 Primary Endpoints 27
4.2 Secondary Endpoints 27
4.3 Exploratory Endpoints 28

5.0 STUDY DESIGN 29

5.1 Definition of Dose Limiting Toxicity- Groups 1 and 2 29
5.2 Definition of Maximum Tolerated Dose- Groups 1 and 2 29
5.3 Sentinel Participants, Dose Escalation and Determination of Maximum Tolerated Dose 29
5.4 Determination of Antiviral Effect – Group 3 31
5.5 Duration of the Study 31
5.6 Study Population 31
5.7 Inclusion Criteria 32
5.8 Exclusion Criteria 33
5.9 Recruitment of Participants 36

6.0 STUDY VISITS 37

6.1 Screening Period 37
6.2 IV infusion of Investigational Product Visit 37
6.3 Post-IV infusion of Investigational Product Visits 38
6.4 Additional Follow-up Visits 39
6.5 Unscheduled Visits 39
6.6 Final Study Visit or Early Termination Visit 39

7.0 STUDY PROCEDURES 40

7.1 Informed Consent Process 40
7.2 Medical History and Physical Examination 40
7.3 HIV Testing and HIV-test Counselling (Groups 1 and 2) 41
7.4 HIV Risk Reduction Counselling 41
7.5 Family Planning Counselling 41
7.6 ART Counselling (Group 3) 41
7.7 Specimens 42

This copy of the document was retrieved from the system by Lisa Sunner on 24 Apr 2019

7.8 Reimbursement 42
7.9 Randomization and Blinding 42
7.10 Un-blinding Procedure for Individual Participants 43
7.11 Assessment of PGDM1400 mAb and/or PGT121 mAb related HIV sero-positivity 43

8.0 INVESTIGATIONAL PRODUCT 44

8.1 Description 44
8.2 Shipment and Storage 45
8.3 Preparation of investigational product 45
8.4 Administration of investigational product 45
8.5 Accountability and Disposal of investigational product 46

9.0 ASSESSMENTS 47

9.1 Safety Assessments 47
9.1.1 Local reactogenicity 47
9.1.2 Systemic reactogenicity 47
9.1.3 Vital Signs 47
9.1.4 Other Adverse Events 47
9.1.5 Concomitant Medications 48
9.1.6 Routine laboratory parameters 48
9.1.7 Specific screening tests: 48
9.2 Virologic Assessments 49
9.3 Exploratory Immunogenicity Assessments 49
9.3.1 Antibody Responses 49
9.3.2 Cellular Responses 50
9.3.3 PBMC, Serum and Plasma Storage 50
9.4 Other Assessments 50
9.4.1 HIV Antibody Testing (Groups 1 and 2) 50
9.4.2 Pharmacokinetics 50
9.4.3 HLA Typing 51
9.4.4 Pregnancy Test 51
9.4.5 HIV Risk Assessment (Group 1 and 2) 51
9.4.6 Social Impact Assessment 51

10.0 ADVERSE EVENTS 52

10.1 Definition 52
10.2 Assessment of Severity of Adverse Events 52
10.3 Relationship to Investigational Product 52
10.4 Serious Adverse Events 53
10.5 Reporting Potential Immune-Mediated Diseases 54
10.6 Clinical Management of Adverse Events 55
10.7 Clinical Management of Infusion-Related Reactions and Stopping Criteria 56
10.8 Pregnancy 57
10.9 Intercurrent HIV Infection (Group 1 and 2) 57

11.0 MANAGEMENT OF HIV ISSUES DURING AND FOLLOWING STUDY 58

11.1 HIV Testing – Groups 1 and 2 58
11.2 Social Discrimination as a Result of investigational product-related antibodies 58
11.3 HIV infection – Group 1 and 2 58
11.3.1 Counseling 58
11.3.2 Referral for Support/Care 59

This copy of the document was retrieved from the system by Lisa Sunner on 24 Apr 2019

12.0 WITHDRAWAL FROM STUDY 60
12.1 Deferral of IV infusion of investigational product 60
12.2 Withdrawal from the Study (Early Termination) 60
13.0 DATA HANDLING 61
13.1 Data Collection and Record Keeping at the Study Site 61
13.2 Data Entry at the Study Site 61
13.3 Data Analysis 61
14.0 STATISTICAL CONSIDERATIONS 62
14.1 Safety and Tolerability Analysis 62
14.2 Pharmacokinetic Analysis 63
14.3. Virologic Analysis for Group 3 64
14.4 Secondary and Exploratory Immunologic and Virologic Analyses 65
15.0 QUALITY CONTROL AND QUALITY ASSURANCE 66
16.0 DATA AND BIOLOGICAL MATERIAL 66
17.0 ADMINISTRATIVE STRUCTURE 67
17.1 Protocol Safety Review Team 67
17.2 Safety Monitoring Committee (SMC) 67
17.2.1 Content of Interim Safety Review 67
17.2.2 SMC Review of Group 1 and 2 data prior to starting Group 3 68
17.3 Criteria for Pausing the Study 68
17.4 Study Supervision 69
17.5 Study Monitoring 70
17.6 Investigator's Records 70
18.0 INDEMNITY 71
19.0 PUBLICATION 71
20.0 ETHICAL CONSIDERATIONS 71
APPENDIX A: SCHEDULE OF PROCEDURES – GROUP 1 (A, B, C) 72
APPENDIX B: SCHEDULE OF PROCEDURES – AND GROUP 2 (A, B, C) 74
APPENDIX C: SCHEDULE OF PROCEDURES – GROUP 3 (A, B) 76
APPENDIX D: LOW RISK CRITERIA 78
APPENDIX E REFERENCES 80
APPENDIX F CTCAE TABLE 83
APPENDIX G: DAIDS ADVERSE EVENT SEVERITY ASSESSMENT TABLE 94

This copy of the document was retrieved from the system by Lisa Sunner on 24 Apr 2019

ABBREVIATIONS

Abbreviation	Term
**AE**	Adverse Event
**AIDS**	Acquired Immunodeficiency Syndrome
**ALT**	Alanine-Aminotransferase
**ART**	Antiretroviral Therapy
**AST**	Aspartate-Aminotransferase
**CFC**	Cytokine Flow Cytometry
**CMI**	Cell Mediated Immunity
**CRF**	Case Report Form
**CTL**	Cytotoxic T Lymphocyte
**DCC**	Data Coordinating Center
**DLT**	Dose Limiting Toxicity
**DNA**	Deoxyribonucleic Acid
**ELISA**	Enzyme Linked Immunosorbent Assay
**GCP**	Good Clinical Practice
**GFR**	Glomerular Filtration Rate
**HIV**	Human Immunodeficiency Virus
**HLA**	Human Leukocyte Antigen
**HSV**	Herpes Simplex Virus
**IAVI**	International AIDS Vaccine Initiative
**ICH**	International Council for Harmonization of Technical Requirements for Pharmaceuticals for Human Use
**IP**	Investigational Product
**IND**	Investigational New Drug Application
**IV**	Intravenous
**Kg**	Kilogram
**mAb**	Monoclonal Antibody
**mg**	Milligram
**MTD**	Maximum Tolerated Dose
**NHP**	Non Human Primate
**PCR**	Polymerase Chain Reaction
**PBMC**	Peripheral Blood Mononuclear Cells
**PK**	Pharmacokinetic
**RPR**	Rapid Plasma Reagin
**SAE**	Serious Adverse Event
**SIV**	Simian Immunodeficiency Virus
**SOP**	Standard Operating Procedure
**SOM**	Study Operations Manual
**SMC**	Safety Monitoring Committee
**STD**	Sexually Transmitted Disease
**TPHA**	Treponema Pallidum Hemagglutination

TMF-03635 | 4.0 | Status:Approved | 16 Apr 2019
This copy of the document was retrieved from the system by Lisa Sunner on 24 Apr 2019

CONTACT INFORMATION

Detailed contact information provided in the Study Operation Manual (SOM)

Sponsor Contact:	
Frances Priddy MD MPH<br>Chief Medical Officer and Executive Director<br>International AIDS Vaccine Initiative<br>125 Broad Street, 9th Floor<br>New York, New York 10004	Phone: +1-212-328-7461<br>Mobile: +1-646-287-8943<br>Fax: +1-608-203-5501<br>E-mail: fpriddy@iavi.org
**Clinical Research Center Contacts:**	
Boris D. Juelg MD PhD<br>Center for Virology and Vaccine Research<br>Clinical Trials Unit<br>Beth Israel Deaconess Medical Center<br>E / CLS – 10th Floor, Room 1046<br>330 Brookline Avenue<br>Boston, Massachusetts 02215	Phone: +1-857-268-7088<br>Mobile: +1-617-401-6725<br>Fax: +1-617-735-4566<br>E-mail: bjulg@bidmc.harvard.edu

.

This copy of the document was retrieved from the system by Lisa Sunner on 24 Apr 2019

1.0 SIGNATURE PAGE

The signatures below constitute the approval of this protocol and the appendices and provide the necessary assurances that this study will be conducted in compliance with the protocol, Good Clinical Practices (GCP) and the applicable regulatory requirement(s).

Principal Investigator:

Signed: [signature] Date: 1 Feb 2021

Name (please print): BORIS JUELG

Name of institution (please print): BIDMC

2.0 INTRODUCTION AND BACKGROUND INFORMATION

In 2015, 36.7 million people globally were living with HIV, 2.1 million people became newly infected with HIV, and 1.1 million people died from AIDS-related illnesses (UNAIDS 2016). More than 78 million people have become infected with HIV and 39 million people have died since the beginning of the AIDS epidemic (UNAIDS 2016). One reason that such high rates of AIDS-related deaths continue to occur globally – despite the advent of drugs that are highly effective at suppressing HIV replication – is that by June 2016, 18.2 million people (two in four people living with HIV) were accessing antiretroviral therapy (ART) (UNAIDS 2016). Another reason for continued AIDS-related mortality is that ART does not cure HIV infection and must be maintained for a lifetime (Barouch and Deeks 2014). Even in the United States (US), only 30% of the 1.2 million people living with HIV have suppressed HIV to undetectable levels, likely because 1) not everyone is aware of their HIV-positive status, 2) not everyone is accessing ART, and 3) of the challenge of maintaining adequate continuity of ART over many years (CDC 2014).

2.1 Study Rationale

This is a Phase 1 study to evaluate the safety, tolerability, pharmacokinetics and anti-viral efficacy of the PGDM1400, PGT121 and VRC07-523LS mAbs for HIV prevention and therapy. PGDM1400, PGT121 and VRC07-523LS mAbs are recombinant human IgG1 monoclonal antibodies that target a V1V2 (PGDM1400), a V3 glycan-dependent (PGT121) and the CD4 binding site (VRC07-523LS) epitope region of the HIV envelope protein (Jardine, Julien et al. 2013, Rudicell, Kwon et al. 2014, Sok, Doores et al. 2014). PGDM1400, PGT121 and VRC07-523LS mAbs were chosen for this study because of their potency, their ability to neutralize a wide array of cross-clade HIV viruses in a complementary pattern, and their proven antiviral activity in animals studies e.g., their capacity to robustly prevent and treat simian-human immunodeficiency virus (SHIV) in rhesus monkeys.

Some HIV-infected individuals develop HIV specific antibodies capable of neutralizing a broad range of HIV virus strains (Simek, Rida et al. 2009, Walker, Phogat et al. 2009). By selecting and cloning B-cells from such HIV-infected individuals, monoclonal antibodies have been identified which have potent and broadly neutralizing activity against a wide range of HIV virus strains (Walker LM 2010). New developments in high throughput single-cell BCR-amplification and novel soluble Env baits have led to the isolation of monoclonal antibodies with extraordinary potency and breadth (Walker, Huber et al. 2011).

These HIV-specific antibodies target the HIV envelope (Env) and can prevent SHIV infection in rhesus monkeys and have been shown to reduce HIV RNA levels in humans temporarily (Hessell, Poignard et al. 2009, Hessell, Rakasz et al. 2009, Moldt, Rakasz et al. 2012, Barouch, Whitney et al. 2013, Caskey, Klein et al. 2015, Caskey, Schoofs et al. 2017). These broadly neutralizing antibodies (bnAbs) may be effective for prevention of HIV infection when administered passively (Haynes and McElrath 2013, Burton and Mascola 2015). It is likely that bnAbs targeting different HIV epitopes will need to be used in combination to prevent development of resistance and to adequately cover all global HIV strains.

This copy of the document was retrieved from the system by Lisa Sunner on 24 Apr 2019

PGDM1400 is a novel monoclonal antibody that targets the V1V2 loop on the HIV Env spike, distinct from VRC01 and 3BNC117 which both target the CD4 binding site, and PGT121 and 10-1074 which target the V3 loop. PGDM1400 can neutralize a wide array of HIV-1 viruses *in vitro* and can treat and prevent simian-human immunodeficiency virus (SHIV) in the NHP model (Sok, van Gils et al. 2014, Julg, Tartaglia et al. 2017)

PGT121 can neutralize a wide array of HIV-1 viruses in vitro and can treat and prevent simian-human immunodeficiency virus (SHIV) in the NHP model (Walker, Huber et al. 2011, Moldt, Rakasz et al. 2012, Barouch, Whitney et al. 2013).

VRC07-523 neutralizes more HIV envelope strains at lower concentrations than other CD4 binding site antibodies (CD4bs) such as VRC01, which was originally discovered in an individual infected with HIV-1 for more than 15 years and whose immune system controlled the virus without ART (Zhou, Georgiev et al. 2010). VRC07-523LS was found to be 5- to 8-fold more potent than VRC01, with an inhibitory concentration IC50 < 50 mcg/mL against 96% of HIV-1 pseudoviruses representing the major circulating HIV-1 clades, and an IC50 < 1 mcg/ml against 92% of HIV-1 viruses tested. In addition, it displayed minimal levels of autoreactivity. VRC07-523LS was shown to have a prolonged half-life over the original VRC07 by about 2-fold.

PGDM1400 mAb, PGT121 mAb and VRC07-523LS mAb were selected for development because of the following critical attributes:

1. PGDM1400 neutralizes 83% of global viral isolates at a median IC50 of 0.003 µg/mL (Sok et al 2014) and is therefore 10 to 100-fold more potent than the previous best-in-class CD4bs antibodies VRC01, VRC07, and 3BNC117 (Scheid, Mouquet et al. 2011, Walker, Huber et al. 2011, Sok, van Gils et al. 2014)
2. PGDM1400 protects against SHIV acquisition in monkeys at substantially lower dose levels compared to VRC01 and 3BNC117 (Julg, Tartaglia et al. 2017)
3. PGT121 mAb has superior therapeutic efficacy in SHIV-infected monkeys compared to VRC01, 3BNC117, and 10-1074 (Barouch, Whitney et al. 2013) (and unpublished data). Specifically, SHIV-infected monkeys with low baseline viral load achieved and maintained undetectable viral loads for up to 100 days after administration of PGT121 (Barouch, Whitney et al. 2013 and unpublished data).
4. PGT121 mAb may have a higher bar to escape in vivo as compared to other V3 glycan and CD4bs antibodies as a result of making multiple glycan contacts (Sok, van Gils et al. 2014).
5. VRC07-523LS is 5 to 8 times more potent than VRC01, and neutralizes 96% of viruses tested (Rudicell, Kwon et al. 2014).
6. VRC07-523LS provides protection against SHIV infection in monkeys at a 5-fold lower concentration than VRC01 (Rudicell, Kwon et al. 2014).
7. PGDM1400 and PGT121 are complementary in the coverage of global viral isolates and when combined neutralize 98-99% of global HIV-1 viruses tested and at unparalleled potency with a median IC50 of 0.007 µg/ml (Sok, van Gils et al. 2014).
8. PGDM1400, PGT121 and VRC07-523LS and are complementary in the coverage of global viral isolates due to targeting separate sites on the HIV-1 Env protein. The triple combination of PGDM1400 + PGT121 + VRC07-523LS is among the most potent and broad at in vitro neutralization (among 56 triple bnAb combinations evaluated) when focusing on all subtypes combined, subtype B alone, or subtype C alone. The coverage of IC80 neutralization at 10 mcg/mL (3.3 mcg/mL each bnAb) was 98.6% for the full cross clade virus panel. Based on theoretical modeling, 99%

This copy of the document was retrieved from the system by Lisa Sunner on 24 Apr 2019

of 208 viruses in a cross-clade panel are covered with at least one antibody while 86% are covered with at least 2 antibodies.

The potency and breadth of PGDM1400, PGT121 and VRC07-523LS raise the possibility that monoclonal antibodies may be effective for HIV prophylaxis at low dose and against global viruses. An antibody that is effective at low doses may eventually be given subcutaneously, which would reduce the cost. It is these features that make PGDM1400, PGT121 and VRC07-523LS particularly well-suited for preventing and/or treating HIV in the developing world, where it is critical that a public health intervention be low cost, easy to deliver, and effective in diverse settings. While VRC07- 523LS has a longer half-life, PGT121 and PGDM1400 are more potent. Neutralization sensitivity profiles are complementary; and the combination of these mAbs with unique epitope specificities will provide invaluable experience assessing the potential additive, synergistic, or antagonistic properties of three bnAbs given sequentially.

2.2 Clinical experience with PGDM1400 and PGT121 and VRC07-523LS

There is no previous clinical experience with PGDM1400 mAb, and limited experience with PGT121 mAb and VRC07-523LS.

The safety, pharmacokinetics and antiviral effects of PGT121 mAb alone are being evaluated in the ongoing T001 study (IND 126807, NCT02960581). As of December 2018, accrual is complete in Groups 1A-1C (HIV-uninfected, 3-30 mg/kg, IV route), Group 1D (HIV-uninfected, 3 mg/kg, SC route), Groups 2A-2C (HIV-infected on ART, 3-30 mg/kg, IV route) and Group 3A (HIV-infected off ART with baseline VL 2000-100.000 copies/mL, 30 mg/kg, IV route). Enrollment in Group 3D (HIV-infected off ART with baseline VL 100-2.000 copies/mL, 30 mg/kg, IV route) is ongoing. After intravenous administration of 3 mg/kg, 10 mg/kg or 30 mg/kg, there was short-lived, mild or moderate local and systemic reactogenicity. After subcutaneous administration of 3 mg/kg, there was short-lived, mild local reactogenicity but no systemic reactogenicity. There have been no related SAEs. There has been one unrelated SAE (hospital admission for orthopedic surgery). To date, there have been no study safety pauses for AEs and product administrations have been generally well tolerated.

The safety and pharmacokinetics of 1 to 3 administrations of VRC07-523LS antibody are being evaluated in healthy, HIV-uninfected adults in the study VRC 605 (NCT03015181). The doses being evaluated are a single administration of 1 mg/kg and 5 mg/kg IV and SC, and 20 mg/kg and 40 mg/kg IV, and three administrations (q 12 weeks) of 5 mg/kg SC and 20 mg/kg IV VRC07-523LS. The study is fully enrolled and study product administration is complete. There have been no serious adverse events (SAEs) or safety pauses and product administrations have been generally well tolerated, with no reports of grade 3 or higher related adverse events deemed related to the study product.

A second study, HVTN 127/HPTN 087 (NCT03387150), is evaluating the safety and serum concentrations of VRC07 523LS administered in multiple doses and routes to healthy, HIV-uninfected adults. Until october 2018, HVTN 127/HPTN 087 enrolled a total of 124 participants. No Grade 3 or higher AEs deemed related to VRC07-523LS and no serious adverse events (SAEs) have been reported in HVTN 127/HPTN 087. The majority of participants have reported no local or systemic solicited AEs and no unsolicited AEs. The few reported unsolicited AEs have been Grade 1 (mild) and the few reported solicited AEs have been Grade 1 (mild) or Grade 2 (moderate).

This copy of the document was retrieved from the system by Lisa Sunner on 24 Apr 2019

Several other HIV monoclonal antibodies are currently in clinical development as passive HIV immunoprophylaxis, or as potential therapeutics (10-1074, 3BNC117, VRC01, VRC01LS). Published data from phase 1 studies shows acceptable preliminary safety and tolerability profiles for these products, and similar anti-viral effects and pharmacokinetics (Caskey, Klein et al. 2015, Ledgerwood, Coates et al. 2015, Lynch, Boritz et al. 2015, Bar, Sneller et al. 2016, Scheid, Horwitz et al. 2016, Schoofs, Klein et al. 2016, Caskey, Schoofs et al. 2017). A comprehensive summary of phase 1 studies of HIV monoclonal antibodies can be found in the Investigator’s Brochures for PGT121 and PGDM1400.

This copy of the document was retrieved from the system by Lisa Sunner on 24 Apr 2019

3.0 STUDY OBJECTIVES

3.1 Primary Objectives

- To evaluate the safety and tolerability of IV infusion of PGDM1400 mAb alone, and a combination of PGDM1400 mAb and PGT121 mAb, in HIV-uninfected adults and HIV-infected adults, and a combination of PGDM1400 mAb and PGT121 mAb and VRC07-523LS mAb in HIV-infected adults
- To evaluate the pharmacokinetic (PK) profile of IV infusion of PGDM1400 mAb alone, and a combination of PGDM1400 mAb and PGT121 mAb, in HIV-uninfected adults and HIV-infected adults, and a combination of PGDM1400 mAb and PGT121 mAb and VRC07-523LS mAb in HIV-infected adults
- To evaluate the antiviral activity of IV infusion of a combination of PGDM1400 mAb and PGT121 mAb, and a combination of PGDM1400 mAb and PGT121 mAb and VRC07-523LS mAb, in HIV-infected adults not on ART

3.2 Secondary Objectives

- To determine if PGDM1400 mAb alone, and in combination with PGT121 mAb, and in combination with PGT121 mAb and VRC07-523LS mAb, induces anti-PGDM1400 and/or anti-PGT121 and/or VRC07-523LS antibodies
- To determine the effect of PGDM1400 mAb in combination with PGT121 mAb, and PGDM1400 in combination with PGT121 mAb and VRC07-523LS mAb, on CD4+ T cell counts in HIV-infected adults
- To determine the effect of PGDM1400 mAb in combination with PGT121 mAb, and PGDM1400 in combination with PGT121 mAb and VRC07-523LS mAb, on viral escape in viremic HIV-infected adults not on ART

3.3 Exploratory Objectives

- To determine if PGDM1400 ± PGT121 ± VRC07-523LS mAb has any impact on the host immune responses (i.e., HIV-specific cellular and humoral immune response).
- To determine the effect of PGDM1400 + PGT121 ± VRC07-523LS mAb on the size of the latent HIV reservoir in HIV-infected adults.
- To determine PGDM1400 mAb ± PGT121 ± VRC07-523LS mAb levels in mucosal secretions in HIV-uninfected and HIV-infected adults.
- To measure in vitro neutralization of HIV isolates with participant's serum post PGDM1400 ± PGT121 ± VRC07-523LS mAb IV infusion.

TMF-03635 | 4.0 | Status:Approved | 16 Apr 2019
This copy of the document was retrieved from the system by Lisa Sunner on 24 Apr 2019

4.0 STUDY ENDPOINTS

4.1 Primary Endpoints

Safety and Tolerability

1. Proportion of participants with moderate or greater reactogenicity (e.g., solicited adverse events) for 3 days following IV infusion of PGDM1400 mAb alone, and a combination of PGDM1400 mAb and PGT121 mAb, and a combination of PGDM1400 mAb and PGT121 mAb and VRC07-523LS mAb
2. Proportion of participants with adverse events (AEs), including safety laboratory (biochemical, hematological) parameters, during the first 56 days following IV infusion of PGDM1400 mAb alone and a combination of PGDM1400 mAb and PGT121 mAb, and a combination of PGDM1400 mAb and PGT121 mAb and VRC07-523LS mAb, that are moderate or greater, and/or related to PGDM1400 mAb or PGT121 mAb or VRC07-523LS mAb
3. Proportion of participants with serious adverse events (SAEs) throughout the study period following IV infusion of PGDM1400 mAb alone and a combination of PGDM1400 mAb and PGT121 mAb, and a combination of PGDM1400 mAb and PGT121 mAb and VRC07-523LS mAb, that are related to PGDM1400 mAb or PGT121 mAb or VRC07-523LS mAb

Pharmacokinetics

Pharmacokinetics following IV infusion of PGDM1400 mAb alone or a combination of PGDM1400 mAb and PGT121 mAb in HIV-uninfected and HIV-infected adults, or a combination of PGDM1400 mAb and PGT121 mAb and VRC07-523LS mAb in HIV-infected adults:

1. Elimination half-life (t1/2)
2. Clearance (CL/F)
3. Volume of distribution (Vz/F)
4. Area under the concentration decay curve (AUC)
5. Impact of viral load and/or ART on PGDM1400 mAb and PGT121 mAb and VRC07-523LS mAb disposition (elimination half-life (t1/2), clearance (CL/F), volume of distribution (Vz/F), total exposure)

Antiviral activity

Antiviral activity following IV infusion of PGDM1400 mAb in combination with PGT121 mAb, or PGDM1400 mAb in combination with PGT121 mAb and VRC07-523LS mAb, in viremic HIV-infected adults not on ART:

1. Change in plasma HIV-1 RNA levels from baseline (mean of pre-entry and entry values)

4.2 Secondary Endpoints

Anti-PGDM1400, anti-PGT121 and anti-VRC07-523LS antibodies

1. Serum anti-PGDM1400 antibody titers
2. Serum anti-PGT121 antibody titers
3. Serum anti-VRC07-523LS antibody titers

TMF-03635 | 4.0 | Status:Approved | 16 Apr 2019
This copy of the document was retrieved from the system by Lisa Sunner on 24 Apr 2019

CD4+ T cell count

We will calculate the following endpoint to determine if IV infusion of PGDM1400 mAb in combination with PGT121 mAb, or PGDM1400 mAb in combination with PGT121 mAb and VRC07-523LS mAb, has any impact on CD4+ T cell counts in HIV-infected adults:

1. Change in CD4+ T cell count and frequency compared to baseline as measured by single platform flow cytometry

HIV genotyping/phenotyping of circulating virus for evaluation of PGDM1400 mAb and/or PGT121 mAb and/or VRC07-523LS mAb -induced escape mutations

We will compare plasma virus genotype and phenotypic activity before and after IV infusion of PGDM1400 mAb in combination with PGT121 mAb, or PGDM1400 mAb in combination with PGT121 mAb and VRC07-523LS mAb to determine if PGDM1400 mAb and PGT121 mAb and/or VRC07-523LS mAb induced viral escape mutations have developed in viremic HIV-infected adults not on ART.

1. Genotypic analysis: Development of sequence variations in epitopes known to result in reduced PGDM1400 mAb and/or PGT121 mAb and/or VRC07-523LS mAb neutralization susceptibility or known to cause resistance to antiretroviral drugs.
2. Phenotypic analysis: Changes in viral susceptibility to PGDM1400 mAb and/or PGT121 mAb and/or VRC07-523LS mAb neutralization.

4.3 Exploratory Endpoints

Additional assessments may include but are not limited to the following: HIV-specific IgG/IgA binding responses by ELISA, HIV-specific cellular immune responses by ELISPOT, HIV-specific antibody function by ADCC, ADCP, and ADCVI assays, PGDM1400 mAb and/or PGT121 mAb and/or VRC07-523LS mAb levels in mucosal secretions, changes in total HIV-1 DNA and 2-long terminal repeat (LTR) circular HIV-1 DNA in resting or total CD4 T cells and in vitro neutralization of HIV isolates with participant's serum post IV infusion of PGDM1400 mAb ± PGT121 mAb ± VRC07-523LS mAb.

This copy of the document was retrieved from the system by Lisa Sunner on 24 Apr 2019

5.0 STUDY DESIGN

The study is a double-blind, randomized, placebo-controlled study for Groups 1 and 2, and open label for Group 3 who will not receive placebo. A single intravenous (IV) infusion of PGDM1400 mAb alone at 3, 10 or 30 mg/kg, or a combination of PGDM1400 mAb and PGT121 mAb at 3, 10 or 30 mg/kg each, or a combination of PGDM1400 mAb and PGT121 mAb and VRC07-523LS mAb at 20 mg/kg each, or placebo will be administered to participants. See study Table 5.0.1 for details.

Table 5.0.1 Study Design Table

	Group	Participants	Sub-Group	Regimen	N	Dose (mg/kg)
Part 1 – MTD	1	HIV-uninfected participants	1A	PGDM1400/Placebo	3/1 (6/2 if DLT)	3 IV
			1B	PGDM1400/Placebo	3/1 (6/2 if DLT)	10 IV
			1C	PGDM1400/Placebo	3/1 (6/2 if DLT)	30 IV
				Total Group 1	9/3 = 12 (max 18/6 = 24 if DLT)	
	2	HIV-uninfected participants	2A	PGDM1400 + PGT121/Placebo	3/1 (6/2 if DLT)	3 + 3 IV
			2B	PGDM1400 + PGT121/Placebo	3/1 (6/2 if DLT)	10 + 10 IV
			2C	PGDM1400 + PGT121/Placebo	3/1 (6/2 if DLT)	30 + 30 IV
				Total Group 2	9/3 = 12 (max 18/6 = 24 if DLT)	
				Total Groups 1 and 2	18/6 = 24 (max 36/12 = 48 if DLT)	
Safety Monitoring Committee						
Part 2 – antiviral effect	3	HIV-infected off ART (VL $1x10^3$ – $1x10^5$ copies/ml)	3A	PGDM1400 + PGT121 + VRC07-523LS	3 (max 9)	20 + 20 + 20 IV
			3B	PGDM1400 + PGT121	3 (max 9)	MTD IV
				Total Group 3	6 (max 18)	
				Total entire study	30 (max 66)	

DLT, dose limiting toxicity; MTD, maximum tolerated dose

5.1 Definition of Dose Limiting Toxicity- Groups 1 and 2

Dose limiting toxicity (DLT) will be defined as 1) any Grade 3 or greater adverse event considered possibly, probably or definitely related to investigational product or 2) any Grade 3 or greater reactogenicity or 3) any SAE considered possibly, probably or definitely related to investigational product.

5.2 Definition of Maximum Tolerated Dose- Groups 1 and 2

If 2 or more DLTs occur in a dose Subgroup (e.g., 1B) that are the same, similar, or in the same System Organ Class, infusion will be halted and the next lower dose level will be declared the maximum tolerated dose (MTD) within this Group. If no DLT occurs in the final dose Subgroup (1C and 2C), MTD will be the highest dose given (30mg/kg) after 14 days of follow-up.

5.3 Sentinel Participants, Dose Escalation and Determination of Maximum Tolerated Dose

Part 1 of this study is a dose-escalation trial in HIV-uninfected adults (Groups 1 and 2) to establish the MTD of PGDM1400 mAb alone and a combination of PGDM1400 mAb and

TMF-03635 | 4.0 | Status:Approved | 16 Apr 2019
This copy of the document was retrieved from the system by Lisa Sunner on 24 Apr 2019

PGT121 mAb. There will be sentinel participants in each dose Subgroup, and safety data will be reviewed to determine if dose escalation can proceed.

Sentinel participants in Groups 1 and 2 and 3A
For each dose Subgroup in Groups 1 and 2 and in Subgroup 3A, the 1st 2 participants will be sentinel participants for whom investigational product infusion will be separated by at least 24 hours contingent on review of safety information prior to infusion of the next sentinel participant. If no reactogenicity or adverse events that meet the DLT criteria occur within 24 hours after IV infusion of the 1st participant, the 2nd participant may be infused with investigational product. If no events meeting the DLT criteria occur within 24 hours after the 2nd participant is infused, then the remaining participants in that dose Subgroup will be infused. If events meeting the DLT criteria do occur for the first 2 participants in a dose Subgroup, the data will be reviewed by the Safety Monitoring Committee (SMC) to determine whether further infusions may proceed.

Staggered dose escalation design whereby PGDM1400 alone and PGT121 alone safety data are reviewed before combined infusion of PGDM1400 and PGT121 at the same dose
The combination Groups, starting with PGDM1400 3 mg/kg + PGT121 3 mg/kg can only start when 1) the PSRT has reviewed the safety data through day 14 post investigational product infusion from PGDM1400 alone at 3 mg/kg and has approved dose escalation to 10 mg/kg AND 2) the MTD of PGT121 as determined in the T001 study is 3 mg/kg or higher. Dosing levels of PGDM1400 mAb and PGT121 in the combination Subgroups can never exceed the MTDs for PGDM1400 and PGT121 as determined in the PGDM1400 alone Group 1 in this study, and the PGT121 alone Group in the T001 study.

PSRT review for dose escalation
The Protocol Safety Review Team (PSRT) will review safety data through day 14 post-investigational product infusion for all participants in each dose Subgroup for PGDM1400 mAb alone (Group 1) prior to allowing enrolment of participants into the next higher dose Subgroup in Group 1 or the same dose level PGDM1400 mAb and PGT121 mAb combination dose Subgroup (Group 2).

- If no DLT occurs in the initial 4 participants of a dose Subgroup (e.g., 1A), the study can proceed with enrolment of the next dose Subgroup for PGDM1400 mAb alone (e.g., 1B), and the same dose level PGDM1400 mAb and PGT121 mAb combination dose Subgroup (e.g., 2A).
- If 1 DLT occurs in the initial 4 participants of a dose Subgroup (e.g., 1A), 4 additional participants will be enrolled in the same dose Subgroup.
    - If no additional DLTs occur within 14 days of infusion in the 8 total participants, the study can proceed with enrolment of the next dose Subgroup for PGDM1400 mAb alone (e.g., 1B), and the same dose level PGDM1400 mAb and PGT121 mAb combination dose Subgroup (e.g., 2A).
    - If 2 or more DLTs accumulate in a dose Subgroup (e.g., 1B) that are the same, similar, or in the same System Organ Class, infusion will be halted and the next lower dose level will be declared the maximum tolerated dose (MTD) within this Group.
    - If no DLT occurs in the final dose Subgroup (e.g.,1C), the MTD for PGDM1400 alone will be the highest dose given (30mg/kg) after 14 days of follow-up.

This copy of the document was retrieved from the system by Lisa Sunner on 24 Apr 2019

- Dosing levels of PGDM1400 mAb and PGT121 in the combination Subgroups can never exceed the MTDs for PGDM1400 and PGT121 as determined in the PGDM1400 alone Group 1 in this study, and the PGT121 alone Group in the T001 study.

SMC review to determine MTD of PGDM1400 alone
Following IV infusion of of investigational product in the last participant in Group 1 , an independent Safety Monitoring Committee (SMC) will review safety data through day 14 post-investigational product infusion for all participants to confirm MTD of PGDM1400 alone.

SMC review to determine MTD of the combination of PGDM1400 and PGT121
Following IV infusion of investigational product in the last participant in Group 2, the SMC will review safety data through day 14 post-investigational product infusion for all participants to confirm MTD of the combination of PGDM1400 and PGT121, and determine whether Group 3B can initiate enrollment.

5.4 Determination of Antiviral Effect – Group 3

Part 2 of this study will establish the antiviral effect of the combination of PGDM1400 mAb plus PGT121 mAb, and the combination of PGDM1400 mAb plus PGT121 mAb plus VRC07-523LS mAb in HIV infected adults not on ART (Group 3).

Subgroup 3A will start with 20 mg/kg of PGDM1400 mAb, and 20 mg/kg for PGT121 mAb, and 20 mg/kg for VRC07-523LS mAb.

MTD determines dose in Subgroup 3B
Subgroup 3B will receive PGDM1400 mAb + PGT121 mAb at a dose that combines the MTD for PGDM1400 mAb and the MTD for PGT121 mAb (as determined in the ongoing T001 study IND 126807, NCT02960581).

5.5 Duration of the Study

Up to 32 weeks per participant, screening up to 42 (HIV-infected) or up to 56 (HIV-uninfected) days before single IV infusion of investigational product on day 0, and 24 weeks of follow up.

It will take approximately 11 months to enroll the entire study. The exact duration depends on the recruitment rate and how many participants will be required per dose Subgroup as specified in sections 5.3 and 5.4.

5.6 Study Population

The study population consists of HIV-uninfected male or female adults (Group 1 and 2) and HIV-infected males and female adults (Group 3) who meet the detailed inclusion and exclusion criteria listed below, and who in the opinion of the investigator or designee, understand the study and provide written informed consent.

Approximately 30-66 participants (54 active product recipients, 12 placebo recipients) who meet all eligibility criteria will be included in the study. An over-enrollment of up to 5% (up to 5 participants total) will be permitted in the study to facilitate rapid enrollment.

This copy of the document was retrieved from the system by Lisa Sunner on 24 Apr 2019

5.7 Inclusion Criteria

Inclusion criteria for all participants:

1. Willing to comply with the requirements of the protocol and available for follow-up for the planned duration of the study.

2. In the opinion of the Principal Investigator or designee and based on Assessment of Informed Consent Understanding results, has understood the information provided and potential impact and/or risks linked to IV infusion and participation in the trial; written informed consent will be obtained from the participant before any study-related procedures are performed.

3. All heterosexually active female participants must commit to use an effective method of contraception for 3 months following investigational product administration, including:

    a. Condoms (male or female) with or without spermicide

    b. Diaphragm or cervical cap with spermicide

    c. Intrauterine device, or contraceptive implant

    d. Hormonal contraception

    e. Successful vasectomy in the male partner (considered successful if a woman reports that a male partner has [1] documentation of azoospermia by microscopy (< 1 year ago), or [2] a vasectomy more than 2 years ago with no resultant pregnancy despite sexual activity post-vasectomy)

    f. Not be of reproductive potential, such as having undergone hysterectomy, bilateral oophorectomy, or tubal ligation, postmenopausal (> 45 years of age with amenorrhea for at least 2 years, or any age with amenorrhea for at least 6 months and a serum follicle stimulating hormone [FSH] level > 40 IU/L); surgically sterile: no additional contraception required.

    g. Women, who are not heterosexually active at screening, must agree to utilize an effective method of contraception if they become hetero-sexually active, as outlined above.

4. All sexually active males, regardless of reproductive potential, must be willing to consistently use an effective method of contraception (such as consistent male condoms with male and/or female partners) from the day of investigational product administration until at least 3 months following investigational product administration to avoid exposure of partners to investigational product in ejaculate, and to prevent conception with female partners.

5. All female participants must be willing to undergo urine pregnancy tests at time points indicated in the Schedule of Procedures and must test negative prior to investigational product administration.

TMF-03635 I 4.0 I Status:Approved I 16 Apr 2019
This copy of the document was retrieved from the system by Lisa Sunner on 24 Apr 2019

6. A female participant must agree not to donate eggs (ova, oocytes) for the purpose of assisted reproduction until 3 months after investigational product administration. A man must agree not to donate sperm until 3 months after investigational product administration.

7. Willing to forgo donations of blood and/or any other tissues, including bone marrow, during the study and, for those HIV-uninfected participants who test HIV-positive due to investigational product administration, until the anti-HIV antibody titers become undetectable.

Specific inclusion criteria for HIV-uninfected participants (Groups 1 and 2):

8. At least 18 years of age on the day of screening and has not reached his or her 51st birthday on the day of signing the Informed Consent Document.

9. Willing to undergo HIV testing, risk reduction counselling and receive HIV test results.

10. Low risk for HIV infection and willing to maintain low-risk behavior for the duration of the trial.

11. Healthy male or female, as assessed by a medical history, physical exam, and laboratory tests.

Specific inclusion criteria for HIV-infected participants (Groups 3):

12. At least 18 years of age on the day of screening and has not reached his or her 66th birthday on the day of signing the Informed Consent Document.

13. Confirmed HIV-1 infection (HIV Ab+ or HIV RNA+) by documentation in the medical records or in-clinic HIV testing;

14. CD4 ≥ 300 cells/µl.

15. Not receiving cART, and (after appropriate counselling) willing to defer cART treatment for at least 56 days after administration of investigational product.

16. HIV-1 viral load between 1000–100,000 copies/ml, confirmed at screening.

17. Under care of an HIV healthcare provider

5.8 Exclusion Criteria

Exclusion criteria for all participants:

1. Any clinically significant acute or chronic medical condition, other than HIV infection, that is considered progressive or in the opinion of the investigator makes the participant unsuitable for participation in the study.

2. If female, pregnant, lactating or planning a pregnancy during the period of screening through completion of the study.

This copy of the document was retrieved from the system by Lisa Sunner on 24 Apr 2019

3. In the past 6 months a history of alcohol or substance use, including marijuana, judged by the Investigator to potentially interfere with participant study compliance.

4. Bleeding disorder that was diagnosed by a physician (e.g., factor deficiency, coagulopathy or platelet disorder that requires special precautions). Note: A participant who states that he or she has easy bruising or bleeding, but does not have a formal diagnosis and has intramuscular injections and blood draws without any adverse experience, is eligible.

5. History of a splenectomy.

6. Receipt of live attenuated vaccine within the previous 30 days or planned receipt within 30 days after administration of investigational product; or receipt of other vaccine within the previous 14 days or planned receipt within 14 days after infusion with investigational product (exception is live attenuated influenza vaccine within 14 days).

7. Receipt of blood transfusion or blood-derived products within the previous 3 months.

8. Participation in another clinical trial of an investigational product currently, within the previous 3 months or expected participation during this study.

9. Prior receipt of an investigational HIV vaccine candidate, monoclonal antibody or polyclonal immunoglobulin (note: receipt of placebo in a previous HIV vaccine or monoclonal antibody trial will not exclude a participant from participation if documentation is available and the Medical Monitor gives approval).

10. History of severe local or systemic reactogenicity to injections or IV infusion (e.g., anaphylaxis, respiratory difficulties, angioedema);

11. Psychiatric condition that compromises safety of the participant and precludes compliance with the protocol. Specifically excluded are persons with psychoses within the past 3 years, ongoing risk for suicide, or history of suicide attempt or gesture within the past 3 years.

12. If, in the opinion of the Principal Investigator, it is not in the best interest of the participant to participate in the trial.

13. Seizure disorder: a participant who has had a seizure in the last 3 years is excluded. (Not excluded: a participant with a history of seizures who has neither required medications nor had a seizure for 3 years.)

14. Body mass index ≥ 35 or ≤ 18.0.

15. Infectious disease: chronic hepatitis B infection (HbsAg), current hepatitis C infection (HCV Ab positive and HCV RNA positive) or interferon-alfa treatment for chronic hepatitis C infection in the past year, chlamydia, gonorrhea, or active syphilis.

This copy of the document was retrieved from the system by Lisa Sunner on 24 Apr 2019

16. A history of malignancy within the past 5 years (prior to screening) or ongoing malignancy;

17. Active, serious infections (other than HIV-1 infection) requiring parenteral antibiotic, antiviral or antifungal therapy within 30 days prior to enrollment.

Specific exclusion criteria for HIV-uninfected participants (Group 1 and 2):

18. Confirmed HIV-1 or HIV-2 infection.

19. Any clinically relevant abnormality on history or examination including history of immunodeficiency or autoimmune disease; use of systemic corticosteroids, immunosuppressive, anticancer, or other medications considered significant by the investigator within the previous 6 months.

    The following exceptions are permitted and will not exclude study participation: use of corticosteroid nasal spray for rhinitis, topical corticosteroids for an acute uncomplicated dermatitis; or a short course (duration of 10 days or less, or a single injection) of corticosteroid for a non-chronic condition (based on investigator clinical judgment) at least 6 weeks prior to enrollment in this study.

20. Any of the following abnormal laboratory parameters listed below:

    <u>Hematology</u>
    Hemoglobin < 10.5 g/dL in females; hemoglobin <11.0 g/dL in males
    Absolute Neutrophil Count (ANC): ≤ 1000/mm3
    Absolute Lymphocyte Count (ALC): < 650/mm3
    Platelets: < 125,000 mm3 or ≥ 550,000/mm3
    <u>Coagulation</u>
    aPTT: >1.25 x ULN
    INR: ≥1.1 x ULN
    <u>Chemistry</u>
    - Sodium ≤ 135 mEq/L or ≥ 146 mEq/L
    - Potassium ≤ 3.4 mEq/L or ≥ 5.6 mEq/L
    - Creatinine ≥ 1.1 x ULN
    - AST ≥ 1.25 x ULN
    - ALT ≥ 1.25 x ULN
    - Total bilirubin ≥ 1.25 x ULN
    - Alkaline phosphatase ≥ 1.25 x ULN
    - Albumin ≤ 3.0 g/dL or ≤ 30 g/L
    - Creatine kinase ≥ 3.0 x ULN
    - C-reactive protein > 10 mg/L
    - C3 complement < 82 mg/dL
    - C4 complement < 14 mg/dL

    <u>Urinalysis</u>
    Any of the following abnormal findings if consistent with clinically significant disease:
    - Protein = greater than trace on dipstick confirmed by microscopic urinalysis outside institutional range.
    - Blood = greater than trace on dipstick confirmed by >3 RBCs/hpf on microscopic urinalysis (not due to menses).

This copy of the document was retrieved from the system by Lisa Sunner on 24 Apr 2019

Specific exclusion criteria for HIV-infected participants who are not on ART (Group 3):

21. Any clinically relevant abnormality on history or examination including history of immunodeficiency or autoimmune disease, other than HIV; use of systemic corticosteroids, immunosuppressive, anticancer, or other medications considered significant by the investigator within the previous 6 months.

    The following exceptions are permitted and will not exclude study participation: use of corticosteroid nasal spray for rhinitis, topical corticosteroids for an acute uncomplicated dermatitis; or a short course (duration of 10 days or less, or a single injection) of corticosteroid for a non-chronic condition (based on investigator clinical judgment) at least 6 weeks prior to enrollment in this study.

22. Any of the following abnormal laboratory parameters listed below:

    <u>Hematology</u>
    - Hemoglobin < 10.0 g/dL
    - Absolute Neutrophil Count (ANC): < 1000 cells/mm3
    - Platelets: < 100,000 cells/mm3

    <u>Coagulation</u>
    - aPTT: > 1.25 x ULN
    - INR: ≥ 1.1 x ULN

    <u>Chemistry</u>
    - Estimated Glomerular filtration rate (GFR) < 80 mL/min according to the Cockcroft Gault formula for creatinine clearance
        - Male: (140 – age in years) x (wt in kg) = CLcr (mL/min) / 72 x (serum creatinine in mg/dL)
        - Female: (140 – age in years) x (wt in kg) x 0.85 = CLcr (mL/min) / 72 x (serum creatinine in mg/dL)
    - AST ≥ 2.5 x ULN
    - ALT ≥ 2.5 x ULN
    - Total bilirubin ≥ 1.6 x ULN
    - Alkaline phosphatase ≥ 5 x ULN

    <u>Urinalysis</u>
    Any of the following abnormal findings if consistent with clinically significant disease:
    - Protein = greater than 1+ on dipstick confirmed by microscopic urinalysis outside institutional range.
    - Blood = greater than 1+ on dipstick confirmed by > 10 RBCs/hpf on microscopic urinalysis (not due to menses).
    - Leukocytes = greater than 1+ on dipstick confirmed by > 10 WBC/hpf on microscopic urinalysis.

5.9 Recruitment of Participants

Adult male and female participants may be recruited through in-clinic referrals, information presented to community organizations, hospitals, colleges, other institutions and/or advertisements to the general public or from existing cohorts. The information distributed will contain contact details of the trial site.

This copy of the document was retrieved from the system by Lisa Sunner on 24 Apr 2019

6.0 STUDY VISITS

6.1 Screening Period

During Screening, study staff will perform the following procedures:

- Provide and/or review the Informed Consent Document and answer any questions about the study prior to obtaining written informed consent.
- Complete Assessment of Informed Consent Understanding (AOU). Please refer to the Study Operations Manual (SOM)

If the participant agrees to participate, passes the AOU and provides written informed consent, study staff will:

- Conduct HIV test counselling, HIV testing, and HIV risk reduction counselling, as applicable
- Conduct family planning counselling, refer for pregnancy prevention counselling if necessary
- Administer HIV risk assessment (Group 1 and 2)
- Conduct ART counselling (Group 3)
- Perform a comprehensive medical history
- Collect concomitant medication information
- Perform a general physical examination (Refer to Section 7.2)
- Collect specimens for all tests as indicated in the Schedule of Procedures in Appendices A, B and C (for details see Analytical Plan (AP)).

When available, the screening laboratory tests will be reviewed by the trial physician. Screening laboratory test(s) may be repeated once at the discretion of the principal investigator or designee to investigate any isolated abnormalities.

If the screening visit occurs outside the allowable screening window, all screening procedures must be repeated except the comprehensive medical history may be replaced by an interim medical history and the Participant Information Sheet of the Informed Consent Document should be reviewed.

If a participant has signed the Consent Form but does not meet the eligibility criteria, the records must be kept at the site.

6.2 IV infusion of Investigational Product Visit

Prior to the infusion of investigational product, study staff will:

- Answer any questions the participant may have about the study
- Review the Informed Consent Document with the participant
- Review screening safety laboratory data
- Conduct HIV test counselling, and HIV risk reduction counselling , as applicable
- Conduct ART counselling (Group 3)
- Conduct family planning counselling as per site specific procedures and ensure compliance with respective pregnancy prevention method, and discuss male condom use with all male participants

This copy of the document was retrieved from the system by Lisa Sunner on 24 Apr 2019

- Review interim medical history
- Collect concomitant medication information
- Weigh participant and record vital signs
- Perform a symptom-directed physical examination (Refer to Section 7.2)
- Assess at baseline local and systemic signs and symptoms (this includes an examination of IV infusion site)
- Collect specimens for all tests as indicated in the Schedule of Procedures see Appendices A, B and C (for details see AP).
- Obtain pregnancy test results prior to infusion of investigational product.
- Assign an allocation number to the participant according to the instructions specified in the Study Operations Manual.

If a participant has an abnormal laboratory value that is known, at the time of infusion, follow the specified guidelines (Section 12.0).

At the time of infusion of investigational product and after IV infusion of investigational product, study staff will:

- Administer the investigational product as specified in Section 8.4, Administration of Investigational Product and according to the instructions specified in the SOM.
- Observe participant closely during the infusion of investigational product and for at least 30 minutes after IV infusion of investigational product has ended for any acute reactogenicity.
- Every 30 minutes after IV infusion of investigational product, for the 1st 4 hours, and every hour thereafter through 6 hours post-infusion, the study staff will:
  - Record vital signs (pulse, respiratory rate, blood pressure and temperature)
  - Assess any local and systemic reactogenicity
  - Assess any other adverse events
- Collect PK samples according to the Schedule of Procedures

6.3 Post-IV infusion of Investigational Product Visits

The participant will be asked to return to the clinic for post-investigational product infusion visits as indicated in the schedule of procedures (see appendices A, B and C) for an assessment by clinic staff. The participant will be asked to maintain a Memory Aid to track any local and systemic reactogenicity the participant experiences, including temperature, from the day of investigational product infusion for the next 3 days (for a total of 4 days including day of investigational product infusion). Participants will receive a thermometer to take their oral temperature at home once a day. Study staff will review the Memory Aid with the participant and determine the severity of the reactions through discussion with the participant.

The following procedures will be conducted at these visits:

- Review interim medical history
- Collect concomitant medication information
- Perform a symptom-directed physical examination if any signs or symptoms are present
- Assess vital signs (pulse, respiratory rate, blood pressure and temperature)

This copy of the document was retrieved from the system by Lisa Sunner on 24 Apr 2019

- Assess any adverse events and local and systemic reactogenicity (Days 1, 2, 3) including reviewing the Memory Aid.
- Collect specimens for all tests as indicated in the Schedule of Procedures (Appendices A, B and C and AP).

6.4 Additional Follow-up Visits

Assessments and procedures will be performed according to the Schedule of Procedures (Appendices A, B and C).

6.5 Unscheduled Visits

Unscheduled Visits/Contacts are visits/contacts that are not described in the Schedule of Procedures (Appendices A, B and C). Unscheduled visits may occur any time during the study:

- For administrative reasons, e.g., the participant may have questions for study staff or may need to re-schedule a follow-up visit.
- To obtain laboratory test results from a previous visit.
- For other reasons as requested by the participant or site investigator.

All unscheduled visits will be documented in the participants' study records on applicable source documents and entered into the Case Report Form (CRF).

6.6 Final Study Visit or Early Termination Visit

Assessments and procedures will be performed according to the Schedule of Procedures (Appendices A, B and C).

This copy of the document was retrieved from the system by Lisa Sunner on 24 Apr 2019

7.0 STUDY PROCEDURES

7.1 Informed Consent Process

A Master Informed Consent Document consisting of a Participant Information Sheet and a Consent Form is provided by the Sponsor to the trial site. This document is made site-specific and translated (if necessary), submitted and approved by the Institutional Review Board (IRB). The Master and site specific Informed Consent Documents are separate documents and should not be part of the protocol.

Participant Information Sheet
A qualified member of the study staff will conduct the informed consent process by reviewing the Participant Information Sheet and document in the clinic notes.

Consent Form
The participant's consent to participate must be obtained by him/her signing and dating the Consent Form. The person obtaining consent will also sign.

The signed and dated Informed Consent Document must remain at the study site. A copy of the signed/marked and dated Informed Consent Document will be offered to the participant to take home. Those participants who do not wish to take a copy will be required to document that they declined to do so.

7.2 Medical History and Physical Examination

Medical History
At screening, a comprehensive medical history will be collected including previous IV infusions and reaction to IV infusion, history of sexually transmitted infection (STI) and pregnancy prevention practices. At subsequent visits, an interim medical history will be performed.

Physical Examination

General Physical Examination
A general physical examination includes examination of head/ears/eyes/nose and throat, skin, respiratory, cardiovascular, abdominal, limited neurological and musculoskeletal and external ano-genital systems (for HIV-infected participants only) at the time points indicated in the Schedule of Procedures (see Appendices A, B and C).

Symptom-Directed Physical Examination
A symptom-directed physical examination is a targeted examination based on the participant's history or observation. If deemed necessary, this examination should be done at the time points indicated in the schedule of procedures (see Appendices A, B and C).

Measuring Height and Weight
Includes measuring the height and weight at the time points indicated in the Schedule of Procedures (see Appendices A, B and C).

TMF-03635 | 4.0 | Status:Approved | 16 Apr 2019
This copy of the document was retrieved from the system by Lisa Sunner on 24 Apr 2019

Vital Signs

Vital signs including pulse, respiratory rate, blood pressure and temperature are measured and recorded at the time points indicated in the Schedule of Procedures (see Appendices A, B and C)

7.3 HIV Testing and HIV-test Counselling (Groups 1 and 2)

Study staff will perform pre-HIV test counselling prior to collecting blood for an HIV test, and post-HIV test counselling when HIV test results are available. This is referred to as HIV-test counselling, and done according to the CDC guidelines. For more information on HIV testing and HIV-test counselling, see Section 11.0. A screening questionnaire and other tools may be used.

7.4 HIV Risk Reduction Counselling

HIV risk reduction counselling will be provided to all participants as outlined by site-specific SOPs.

Study staff will provide HIV risk reduction counselling based on reported individual risk and provide free condoms, as appropriate, at every visit. Group 1 and 2 will receive HIV risk reduction counselling and for Groups 3, HIV risk reduction counselling will be conducted as secondary prevention to reduce onward transmission.

7.5 Family Planning Counselling

Study staff will counsel participants about the importance of preventing pregnancies and of using condoms, as well as other effective family planning methods until at least 3 months following investigational product administration, as appropriate. Participants may be referred for family planning services as necessary according to site-specific SOPs as detailed in the SOM. Pregnancy prevention methods chosen and compliance will be documented.

7.6 ART Counselling (Group 3)

All HIV-infected participants will receive ART counselling upon entering the study and will be asked to postpone ART initiation for 56 days following infusion of investigational product. Upon completion of the 56 days, all HIV-infected participants will receive ART counselling again and will be asked to initiate ART with their HIV healthcare provider. At all counselling sessions, participants will be advised to discuss this study regarding delaying or starting ART with their HIV healthcare provider. Participants who have not initiated or made plans to initiate ART by the final study visit (day 168) will receive ART counselling again.

HIV-infected participants who are not on ART who achieve an undetectable HIV RNA level after IV infusion of PGDM1400 + PGT121 ± VRC07-523LS and who decide against ART initiation at day 56 despite ART counselling (as above), will be followed until HIV RNA becomes detectable or until the final study visit at which point these participants will be counseled again to initiate ART. HIV RNA in these participants will be assessed at every study visit, (see schedule of procedures, appendix C) and, if needed, participants will be approached for additional HIV RNA level assessments in between planned study visits in order to track individuals HIV RNA levels as needed.

This copy of the document was retrieved from the system by Lisa Sunner on 24 Apr 2019

7.7 Specimens

Approximately 50 ml of blood will be collected from participants in Group 1 and 2, and approximately 150 ml of blood will be collected from participants in Group 3 at the screening visit. At later visits, approximately 8.5 ml to 175 ml of blood will be collected, depending on study procedures and Group assignment (see Appendices A, B and C), usually from the antecubital fossa.

Optional collection of rectal and/or cervical mucosal secretions will be obtained using a rectal sponge (or comparable swab) or cervical Softcup (or comparable cervical fluid collection cup) for those participants that consent.

All specimens will be handled according to the procedures specified in the AP and relevant SOPs if applicable.

In the event of an abnormal laboratory value, participants may be asked to have an additional sample collected at the discretion of the Principal Investigator or designee.

7.8 Reimbursement

Participants will be reimbursed for their time, effort and for costs to cover their travel expenses to the study site and any inconvenience caused due to study participation. Site specific-reimbursement amounts will be documented in the site-specific Participant Information Sheet, and approved by the Institutional Review Board.

7.9 Randomization and Blinding

Participants will be identified by a unique study identification number.

Participants will be randomized according to the randomization schedule prepared by the statisticians at the Data Coordinating Center (DCC) prior to the start of the study. Participants will be automatically assigned a specific allocation number as they are enrolled into the data entry system. An unblinding list (Pharmacy List) will be provided to the unblinded site pharmacist by the DCC.

This a double blind, randomized, placebo-controlled study for Groups 1 and 2, and an open label study for Group 3. For Groups 1 and 2, study staff (investigator and clinical personnel monitoring the safety and laboratory assay results) and participants will be blinded with respect to the allocation of Investigational Product (PGDM1400 mAb alone or PGDM1400 and PGT121 in combination or placebo). A site pharmacist will be unblinded for the purposes of preparing investigational product.

A participant will be considered enrolled once he/she has been assigned an allocation number.

Blinded participants will be informed about their assignment (active product/placebo) once the data is locked.

TMF-03635 | 4.0 | Status:Approved | 16 Apr 2019
This copy of the document was retrieved from the system by Lisa Sunner on 24 Apr 2019

7.10 Un-blinding Procedure for Individual Participants

Un-blinding of an individual participant may be indicated in the event of a medical emergency if the clinical management of the participant would be altered by knowledge of the treatment assignment.

The un-blinded information should be restricted to a small Group of individuals involved in clinical management/medical treatment of the participant (e.g., treating physician) and the blind must be maintained for those responsible for the study assessments.

Should a study participant be unblinded during the study, the study participant will be followed up until the end of the study according to the Schedule of Procedures (Appendices A and B). The reasons for un-blinding should be documented and the IAVI Chief Medical Officer, the Medical Monitor and the DCC should be notified as soon as possible. The procedures and contact numbers for un-blinding are outlined in the SOM.

7.11 Assessment of PGDM1400 mAb and/or PGT121 mAb related HIV sero-positivity

It is possible that PGDM1400 and/or PGT121 or an immune response to PGDM1400 and/or PGT121 could cause a positive result on a diagnostic HIV antigen/antibody test. A Group 1 or 2 participant who tests HIV antigen and/or HIV antibody positive at the end of the study will have additional testing to distinguish actual HIV infection from investigational product-related responses. The participant will be informed of his/her positive HIV antigen and/or HIV antibody test result and offered continuing follow-up until the HIV antigen/antibody test becomes negative.

TMF-03635 | 4.0 | Status:Approved | 16 Apr 2019
This copy of the document was retrieved from the system by Lisa Sunner on 24 Apr 2019

8.0 INVESTIGATIONAL PRODUCT

8.1 Description

PGDM1400 and PGT121 active products are formulated in a 20 mM Acetate, 9% Sucrose, 0.008% polysorbate 80, pH 5.2 formulation buffer at a concentration of 50 mg/mL. Each 10 ml vial contains 6 ml of PGDM1400 or PGT121.

VRC07- 523LS is formulated at a concentration of 100±10mg/mL in a buffer composed of 50 mM histidine, 50 mM sodium chloride, 5% sucrose, and 2.5% sorbitol at pH 6.8. Each vial contains 6.25 ± 0.1 mL or 2.25 ± 0.1 mL of VRC07-523LS filled in a standard 10-mL or 3-mL glass vial, respectively.

A summary of the Investigational Products and example volumes needed for administration for each dose level are shown in Table 8.1-1.

Table 8.1-1 Investigational Products

Active Product / Placebo	Dosage level	Total volume in investigational product container	Total Active Product or placebo volume to be injected into a 100 mL saline IV bag^ (for an 88 kg body weight**)	Total volume to be Infused (for an 88 kg body weight**)
PGDM1400 (50 mg/mL)	3 mg/kg	6 mL per vial	5.3 mL	105.3 mL
	10 mg/kg		17.6 mL	117.6 mL
	20 mg/kg		35.2 mL	135.2 mL
	30 mg/kg		52.8 mL	152.8 mL
PGT121 (50 mg/mL)	3 mg/kg	6 mL per vial	5.3 mL	105.3 mL
	10 mg/kg		17.6 mL	117.6 mL
	20 mg/kg		35.2 mL	135.2 mL
	30 mg/kg		52.8 mL	152.8 mL
VRC07-523LS (100 mg/mL)	20mg/kg	2.25 or 6.25 mL per vial	17.6 mL	117.6 mL
Placebo: 0.9% Sodium Chloride for Injection USP (Saline)*	3 mg/kg matching***	NA	5.3 mL***	105.3 mL***
	10 mg/kg matching***		17.6 mL***	117.6 mL***
	30 mg/kg matching***		52.8 mL***	152.8 mL***

* The Placebo provided will be a commercially-available 0.9% sodium chloride for injection USP partial addition or flexible IV bag.
** The actual volume to be injected will be based on the dose Subgroup and the weight of the participant at the time of investigational product administration. The example included here is the average weight of an adult male in the US (88kg) (http://www.cdc.gov/nchs/data/series/sr_11/sr11_252.pdf)
*** For placebo IV infusions: saline from an additional IV bag will be injected into the saline IV bag intended for administration, to match the volume used for a PGDM1400 ± PGT121 mAb injection in the same dose Subgroup, to prevent unblinding. Placebo recipients in Group 2 will receive 2

This copy of the document was retrieved from the system by Lisa Sunner on 24 Apr 2019

sequential administrations of placebo to mimic administration of PGDM1400 followed by PGT121 to maintain blinding. See SOM for details.
^ In the case of a saline IV bag shortage, a larger size bag may be substituted as long as additional saline is aspirated out so that 100mL remains in the bag.

8.2 Shipment and Storage

Authorization to ship the PGDM1400 and PGT121 and VRC07-523LS to the site will be provided in writing by the Sponsor, upon confirmation that all required critical documents for shipment authorization are completed. PGDM1400 and PGT121 and VRC07-523LS will be shipped maintaining the required storage conditions and stored in a secure location in the clinical site's pharmacy.

PGDM1400 and PGT121 vials will be stored at - 20°C ± 5°C. VRC07-523LS vials will be stored at -35°C to -15°C. Each vial will be labelled with the name of the product, lot number, concentration, fill volume, storage temperature, date of manufacture, name and location of the manufacturer and a US cautionary statement. Several such vials will be packaged in a box. Each box will be labeled with similar information as the vial label, including an address and contact information for the manufacturer.

Each PGT121 and PGDM1400 vial will be labelled with the name of the product, lot number, concentration, fill volume, storage temperature, date of manufacture, name and location of the manufacturer and a US cautionary statement. Each PGT121 and PGDM1400 carton will be labelled with the same information as the vial label but will also include a complete address and contact information for the manufacturer.

Each VRC07-523LS vial label will contain the product description, VRC product number,lot number, fill volume, concentration, fill date, storage condition, US cautionary statement, and the name of the manufacturer. The carton label is identical to the vial label for the VRC07-523LS.

0.9% Sodium Chloride for Injection USP, in partial addition or flexible bags, will be used as the placebo and diluent for PGDM1400 and PGT121 and VRC07-523LS. It will be stored at room temperature.

8.3 Preparation of investigational product

Detailed instructions will be provided in the SOM for preparing each of the investigational products. Infusion of the PGT121 and PGDM1400 investigational product(s) must be completed within 4 hours of the IP being injected into the saline IV bag. Infusion of the VRC07-523LS investigational product must be completed within 8 hours of being injected into the saline IV bag. Example calculations for the volumes needed for IV infusion are illustrated in Table 8.1-1. Procedures for handling used and partially used vials of PGT121, PGDM1400 and VRC07-523LS will be provided in the SOM. Syringes or other components in direct contact with PGT121, PGDM1400 and VRC07-523LS will be disposed of properly in a biohazard container and incinerated or autoclaved as per site procedure.

8.4 Administration of investigational product

Investigational product will be administered at the enrollment visit. The investigational product will be injected into a 0.9% sodium chloride for injection USP partial addition or

This copy of the document was retrieved from the system by Lisa Sunner on 24 Apr 2019

flexible saline IV bag. The participant will receive the investigational product via IV infusion. If more than one investigational product will be administered, these will be administered sequentially in separate saline IV bags. For groups requiring multiple infusions, the desired order of infusion will be as follows: PGDM1400 followed by PGT121 followed by VRC07-523LS. Participants will receive each infusion over approximately 60 minutes, allowing for clinician discretion. Further information on the IV infusion of the investigational product is supplied in the SOM and other study documents.

8.5 Accountability and Disposal of investigational product

All used PGDM1400, PGT121 and VRC07-523LS vials will be handled according to instructions in the SOM. Throughout the study, the investigational product accountability forms including receipt and dispensing of vials will be kept and monitored. The vial label for the used vial(s) will be removed and retained for accountability purposes; the used vial(s) can be discarded as per site procedures.

At the end of the study, the unused PGDM1400, PGT121 and VRC07-523LS vials will be reconciled according to Sponsor instructions.

Further information on accountability and disposal of PGDM1400,PGT121 and VRC07-523LS is supplied in the SOM.

TMF-03635 | 4.0 | Status:Approved | 16 Apr 2019
This copy of the document was retrieved from the system by Lisa Sunner on 24 Apr 2019

9.0 ASSESSMENTS

9.1 Safety Assessments

Data on local and systemic reactogenicity (i.e., solicited AEs) will be collected by structured interview and medical examination. Data on other adverse events will be collected with open-ended questions. All data will be recorded on the appropriate source documents and entered into the study database. Participants will be given a Memory Aid, which is a tool to assist with collecting reactogenicity data.

Local and systemic reactogenicity events will be assessed by study staff prior to and during IV infusion of investigational product, approximately every 30 minutes after investigational product infusion for the first 4 hours after investigational product infusion and subsequently every hour for the first 6 hours post-investigational product infusion. Study staff will review the Memory Aid with the participant, and determine the severity of the reactions on days 1-3 through discussion with the participant.

9.1.1 Local reactogenicity

The presence of local reactogenicity will be assessed at the time points specified in the Schedule of Procedures (Appendices A, B and C).
Pain, tenderness, erythema/skin discoloration, swelling/hardening or pruritus will be assessed and graded using Appendix G, DAIDS Adverse Event Severity Assessment Table, as a guideline.

9.1.2 Systemic reactogenicity

The presence of systemic reactogenicity will be assessed at the time points specified in the Schedule of Procedures (Appendices A, B and C).
Fever, chills, headache, nausea, vomiting, malaise, myalgia and arthralgia will be assessed and graded using the Appendix G DAIDS Adverse Event Severity Assessment Table as a guideline.
For the first 24 hours after investigational product infusion, any infusion related reactions, including cytokine release syndrome, should be graded using the Common Terminology Criteria for Adverse Events (CTCAE) v4.03: June 14, 2010 (Appendix F).

9.1.3 Vital Signs

At the infusion visit, vital signs (pulse, respiratory rate, blood pressure and temperature) will be measured by study staff prior to investigational product infusion, approximately every 30 minutes for the first 4 hours post investigational product infusion and then hourly until 6 hours after IV infusion. For the other study visits vital signs will be assessed at the time points specified in the Schedule of Procedures (Appendices A, B and C).

9.1.4 Other Adverse Events

Other adverse events (AEs) will be collected through 56 days after investigational product infusion in all participants. Serious Adverse Events (SAEs) will be collected throughout the entire study period. Potential Immune Mediated Diseases (pIMDs), as defined in Section 10.5, will be collected throughout the study period, using the SAE reporting process. Open-ended questions will be asked at time points according to the Schedule of Procedures (Appendices A, B

TMF-03635 | 4.0 | Status:Approved | 16 Apr 2019
This copy of the document was retrieved from the system by Lisa Sunner on 24 Apr 2019

and C). All adverse events during the first 24 hours after the infusion will be graded using Appendix G, DAIDS Adverse Event Severity Assessment Table, as a guideline and will be assessed for relatedness to the investigational product. For more information regarding adverse events refer to Section 10.0, Adverse Events.

9.1.5 Concomitant Medications

Concomitant receipt of Investigational Products is prohibited during the study.

Contraceptive use and use of medication at study entry will be documented. (See DCF instructions).

During the study, information regarding concomitant medications and reasons for their use will be solicited from the study participants for 56 days. Ongoing concomitant medications will be recorded until end of study.

9.1.6 Routine laboratory parameters

Table 9.1.6-1 shows the laboratory parameters that will be measured routinely. The samples for these tests will be collected at the time points indicated in the Schedule of Procedures (Appendices A, B and C).

Table 9.1.6-1: Laboratory Parameters

Laboratory Parameter	Test
Hematology and Coagulation	Hemoglobin, hematocrit, leukocytes, platelets, absolute neutrophil count (ANC), absolute lymphocyte count (ALC), activate partial thromboplastin time (aPTT), international normalized ratio (INR)
Clinical Chemistry	Sodium, potassium, creatinine, aspartate aminotransferase (AST), alanine aminotransferase (ALT), total bilirubin, alkaline phosphatase<br>Groups 1 and 2 only: albumin, creatine kinase, C-reactive protein, C3 complement, C4 complement
Urinalysis	Dipstick test for protein, blood glucose, ketones, esterase (leukocytes) and nitrite. If clinically significant abnormalities (e.g., blood, protein, leukocytes) are found on dipstick test, then further test(s) will be performed (e.g., microscopy, culture)
T cell panel (Group 3)	CD4 T cell count and frequency by single platform flow cytometry

9.1.7 Specific screening tests:

Participants will be screened to exclude the following diseases:

- Hepatitis B: positive for hepatitis B surface antigen (HBsAg)
- Hepatitis C: positive for hepatitis C RNA (HCV antibody test, followed by HCV RNA test if HCV antibody positive)
- Chlamydia: confirmed diagnosis
- Gonorrhea: confirmed diagnosis
- Active syphilis: confirmed diagnosis.

This copy of the document was retrieved from the system by Lisa Sunner on 24 Apr 2019

A negative Hepatitis B and Hepatitis C result can be documented from the medical record only if the result is from a test administered less than 6 months ago.

9.1.8 Monitoring for anti-PGDM1400, anti-PGT121 and anti-VRC07-523LS antibodies:

Participants will be evaluated for the development of antibodies to PGDM1400 mAb, PGT121 mAb and VRC07-523LS mAb (anti-drug antibodies, ADA) by ELISA according to the Schedule of Procedures (Appendices A, B and C).

9.2 Virologic Assessments

Table 9.2-1 shows the virologic parameters that will be measured routinely. The samples for these tests will be collected at the time points indicated in the Schedule of Procedures (Appendix C).

Table 9.2-1: Virologic Assessment Table

Virologic Parameter	Test
Antiviral Activity	Plasma HIV RNA levels
Anti-reservoir activity	Cell-associated HIV-1 RNA levels in resting CD4 T cells; total HIV-1 DNA and 2-long terminal repeat (LTR) HIV-1 DNA circles in resting or total CD4 T cells; quantitative viral outgrowth assay (qVOA)
Other	Genotyping of plasma HIV RNA for evaluation of PGDM1400, PGT121 and VRC07-523LS-induced escape mutations and resistance to antiretroviral drugs; phenotyping of plasma HIV RNA for neutralization susceptibility to PGDM1400, PGT121 and VRC07-523LS in-vitro

9.3 Exploratory Immunogenicity Assessments

Humoral immune response assays will include, but are not limited to Env-specific Ab-binding assays, virus neutralization assay, and assays for Ab functionality. Cellular immune response assays will include, but are not limited to IFNγ ELISPOT assay, ICS, and multiparameter flow cytometry. Exploratory assessments on mucosal samples will include, but are not limited to characterization of Env-specific binding Abs. Priority assays are listed below.

9.3.1 Antibody Responses

- Env-specific binding Abs (titers and breadth).
- Env-specific nAbs (titers and breadth).
- Env-specific functional Abs (e.g. phagocytosis score and breadth).
- Env-specific binding Ab isotypes (IgA, IgG1-4) (titers and breadth).

This copy of the document was retrieved from the system by Lisa Sunner on 24 Apr 2019

9.3.2 Cellular Responses

- IFNγ peripheral blood mononuclear cell (PBMC) responders to peptide pools and subpools of Potential T-cell epitopes, PTE Env/Gag/Pol peptides.
- $CD4^+$ and $CD8^+$ T-cell functionality (% cells producing e.g., IFNγ, IL-2, IL-4, TNFα).
- T-cell development with emphasis on follicular helper T-cells and memory differentiation.

9.3.3 PBMC, Serum and Plasma Storage

Samples of cryopreserved PBMC, plasma and serum will be stored as indicated in the Schedule of Procedures in Appendices A, B and C (for details see Analytical Plan (AP)) and, if the participant consents, may be used for the purposes of standardization, quality control and for future assays related to HIV prevention or treatment research and development. These samples will be archived and the testing laboratories will be blinded to the participant's identity.

9.4 Other Assessments

9.4.1 HIV Antibody Testing (Groups 1 and 2)

All HIV-uninfected participants (Group 1 and 2) will be tested for HIV antibodies as indicated in the Schedule of Procedures (Appendix A and B) or as needed, if medical or social circumstances arise. All participants will receive HIV risk reduction counselling and pre- and post-HIV-test counselling, as specified in Section 7.3 Counselling.

9.4.2 Pharmacokinetics

Blood draws for pharmacokinetics will be done on the day of investigational product infusion immediately before starting IV infusion(s) of investigational product, at the end of the investigational product infusion(s), and 3, 6 and 24 hours after the investigational product infusions. Thereafter, pharmacokinetic draws will be done as indicated in the Schedule of Procedures (Appendices A, B and C).

PGDM1400 mAb, PGT121 mAb and VRC07-523LS mAb pharmacokinetic analyses will be performed using, but not limited to, standard non-compartmental analysis methods to estimate elimination half-life (t1/2), clearance (CL/F), volume of distribution (Vz/F), Area under the concentration decay curve (AUC), impact of viral load and/or ART on PGDM1400 mAb, PGT121 mAb and VRC07-523LS mAb disposition (elimination half-life (t1/2), clearance (CL/F), volume of distribution (Vz/F) and total exposure. PGDM1400 ± PGT121 ± VRC07-523LS accumulation will also be examined in rectal and cervical mucosal secretions collected with rectal sponges (or comparable swab) or cervical Softcups (or comparable cervical fluid collection cup) in study participants who specifically consented for these procedures. Descriptive results will be reported for the pharmacokinetic parameters by dose Subgroup.

Exploratory analysis using population analysis methods simultaneously combining all pharmacokinetic data across all doses and treatment Subgroups

This copy of the document was retrieved from the system by Lisa Sunner on 24 Apr 2019

and Groups will be performed for quantitative characterization of differences in PGDM1400 mAb, PGT121 mAb and VRC07-523LS mAb disposition by dose, participant Subgroup or Group or disease state.

9.4.3 HLA Typing

Samples for HLA typing will be collected as specified in the Schedule of Procedures in Appendices A, B and C (for details see Analytical Plan (AP)) and may be analyzed as warranted.

9.4.4 Pregnancy Test

A urine pregnancy test for all female participants will be performed by measurement of human chorionic gonadotrophin (βhCG) at time points indicated in the Schedule of Procedures (Appendices A, B and C). The results of the pregnancy test must be negative prior to IV infusion of investigational product. See section 10.7 for description of pregnancy after infusion of investigational product.

9.4.5 HIV Risk Assessment (Group 1 and 2)

Study staff will assess participants for their past and current risk of acquiring HIV at time points indicated in Schedule of Procedures (Appendix A and B).

9.4.6 Social Impact Assessment

A brief assessment of the impact of participation in the study will be administered to participants at their final study visit.

TMF-03635 | 4.0 | Status:Approved | 16 Apr 2019
This copy of the document was retrieved from the system by Lisa Sunner on 24 Apr 2019

10.0 ADVERSE EVENTS

10.1 Definition

An adverse event (AE) is any untoward medical occurrence in a participant administered an Investigational Product and which does not necessarily have a causal relationship with the Investigational Product. An AE can therefore be any unfavourable or unintended sign (including an abnormal laboratory finding), symptom, or disease, temporally associated with the use of Investigational Product whether or not related to the Investigational Product.

Assessment of severity of all AEs, including and seriousness of AEs, is ultimately the responsibility of the Principal Investigator of each site. Refer to the DIVISION OF AIDS (DAIDS) TABLE FOR GRADING THE SEVERITY OF ADULT AND PEDIATRIC ADVERSE EVENTS Version 2.0, November 2014 and the National Cancer Institute Common Terminology Criteria for Adverse Events (CTCAE) Version 4.03: June 14, 2010 for additional guidance.

10.2 Assessment of Severity of Adverse Events

The following general criteria should be used in assessing adverse events as mild, moderate, severe or very severe at the time of evaluation:

Grade 1 (Mild): Symptoms causing no or minimal interference with usual social & functional activities

Grade 2 (Moderate): Symptoms causing greater than minimal interference with usual social & functional activities

Grade 3 (Severe): Symptoms causing inability to perform usual social & functional activities

Grade 4 (Very Severe): Symptoms causing inability to perform basic self-care functions OR Medical or operative intervention indicated to prevent permanent impairment, persistent disability, or death

Guidelines for assessing the severity of specific adverse events and laboratory abnormalities are listed in Appendix G, DAIDS Adverse Event Severity Assessment Table.

10.3 Relationship to Investigational Product

Assessment of relationship of an AE or SAE to Investigational Product is the responsibility of the Principal Investigator or designee. All medically indicated and available diagnostic methods (e.g., laboratory, blood smear, culture, X-ray, etc.) should be used to assess the nature and cause of the AE/SAE. Best clinical and scientific judgment should be used to assess relationship of AE/SAEs to the investigational product and/or other cause.

TMF-03635 | 4.0 | Status:Approved | 16 Apr 2019
This copy of the document was retrieved from the system by Lisa Sunner on 24 Apr 2019

The following should be considered:

- Presence/absence of a clear temporal (time) sequence between administration of the investigational product and the onset of AE/SAE
- Presence/absence of another cause that could more likely explain the AE/SAE (concurrent disease, concomitant medication, environmental or toxic factors)
- Whether or not the AE/SAE follows a known response pattern associated with the investigational product

The relationship assessment should be reported as one of the following:

Not Related: clearly explained by another cause (concurrent disease, concomitant medication, environmental or toxic factors, etc.).

Unlikely: more likely explained by another cause (concurrent disease, concomitant medication, environmental or toxic factors, etc.).

Possibly: equally likely explained by another cause but the possibility of the investigational product relationship cannot be ruled out (e.g., reasonably well temporally related and/or follows a known investigational product response pattern but equally well explained by another cause).

Probably: more likely explained by the investigational product (e.g., reasonably well temporally related and/or follows a known investigational product response pattern and less likely explained by another cause).

Definitely: clearly related and most likely explained by the investigational product.

For the purpose of expedited safety reporting, all possibly, probably or definitely related SAEs are considered investigational product-related SAEs.

10.4 Serious Adverse Events

An adverse event is reported as a "Serious Adverse Event" if it meets any of the following criteria (as per International Conference on Harmonisation [ICH] Good Clinical Practice [GCP] Guidelines):

- Results in death
- Is life threatening
- Results in persistent or significant disability/incapacity
- Requires in-participant hospitalization or prolongs existing hospitalization
- Is a congenital anomaly/birth defect or spontaneous abortion
- Any other important medical condition that requires medical or surgical intervention to prevent permanent impairment of a body function or structure

Elective surgery for pre-existing condition that did not increase in severity or frequency is not considered an SAE.

Serious Adverse Events (SAEs) should be reported within 24 hours of the site becoming aware of the event, and sent to the Sponsor as described in the SOM.

This copy of the document was retrieved from the system by Lisa Sunner on 24 Apr 2019

To discuss investigational product-related SAEs or any urgent medical questions related to the SAE, the site investigator should contact one of the IAVI Medical Monitors directly (see Contact List in the SOM).

The IAVI SAE Report Form should be completed with all the available information at the time of reporting and sent to the Sponsor as described in the SOM. The minimum data required in reporting an SAE are the study identification number, date of birth, gender, event description (in as much detail as is known at the time), onset date of event (if available), reason event is classified as serious, reporting source (name of Principal Investigator or designee), and relationship to the investigational product as assessed by the investigator.

The Principal Investigator or designee is required to prepare a detailed written report with follow up until resolution or until it is judged by the Principal Investigator or designee to have stabilized.

The Principal Investigator or designee must notify the local IRB/IEC of all SAEs as appropriate. In case of investigational product-related SAEs, the Sponsor will notify responsible regulatory authorities, Safety Monitoring Committee (SMC), and other study sites where the same investigational product is being tested.

More details on SAE definitions and reporting requirements are provided in the SOM.

Serious Event Prior to Investigational Product Administration

If a serious event occurs in the period between the participant signing the Informed Consent Form and receiving the IV infusion of investigational product, the event will be reported using the SAE form and following the same procedures for SAE reporting, as indicated in Section 10.4. The timing of the event will be indicated by using the relevant checkbox on the SAE form.

10.5 Reporting Potential Immune-Mediated Diseases

Potential immune-mediated diseases (pIMDs) are a subset of AEs that include both clearly autoimmune diseases and also other inflammatory and/or neurologic disorders that may or may not have an autoimmune etiology. These events are of special interest since they could potentially be caused by immune responses to the investigational product. The investigator/designee should report such adverse events within the same time limits (following confirmation of an AE as a pIMD; see last paragraph of this section below), and using the same CRF pages, as utilized for SAEs. The investigator or his/her designee will evaluate the occurrence of pIMDs at every visit/contact during the study. IAVI will also expect investigators/designee to provide additional information about pIMD events. AEs to be reported and documented as pIMDs include:

Neuroinflammatory disorders: optic neuritis, cranial nerve disorders (including Bell's palsy), multiple sclerosis, demyelinating disease, transverse myelitis, Guillain-Barré syndrome, myasthenia gravis, encephalitis, neuritis.

Musculoskeletal disorders: systemic lupus erythematosus, cutaneous lupus, Sjögren's syndrome, scleroderma, dermatomyositis, polymyositis, myopathy, rheumatoid arthritis and juvenile rheumatoid arthritis, polymyalgia rheumatica or temporal arteritis, reactive

This copy of the document was retrieved from the system by Lisa Sunner on 24 Apr 2019

arthritis, psoriatic arthropathy, ankylosing spondylitis, undifferentiated spondyloarthropathy.

Gastrointestinal disorders: Crohn's disease, ulcerative colitis or proctitis, celiac disease.

Metabolic diseases: autoimmune thyroiditis, Grave's or Basedow's disease, Hashimoto thyroiditis, insulin-dependent diabetes mellitus (IDDM), Addison's disease.

Skin disorders: psoriasis, vitiligo, Raynaud's phenomenon, erythema nodosum, autoimmune bullous skin diseases.

Others: autoimmune hemolytic anemia, thrombocytopenia, antiphospholipid syndrome, *vasculitis, pernicious anemia, autoimmune hepatitis, primary biliary cirrhosis, primary sclerosing cholangitis, autoimmune glomerulonephritis, autoimmune uveitis, autoimmune myocarditis/cardiomyopathy, sarcoidosis, Stevens-Johnson syndrome, Behçet's syndrome.

Infusion site reactions: Grade 3 or 4 infusion site reactions lasting more than 2 days.

*Vasculitis: Vasculitis, Diffuse vasculitis, leucocytoclastic vasculitis, polyarteritis nodosa, microscopic polyangiitis, Wegener's granulomatosis, anti-neutrophil cytoplasmic antibody positive vasculitis, Henoch-schönlein purpura, allergic granulomatous angiitis (Churg-Strauss disease), Kawasaki disease, Takayasu's arteritis, temporal arteritis (giant cell arteritis), renal vasculitis.

Medical judgement should be exercised in deciding whether other disorders/diseases have an autoimmune origin and should also be reported as described above, and this judgement is the investigator's prerogative. Whenever sufficient data exist to substantiate any of the diagnoses in the above list, the event must be reported as a pIMD. While the intent of pIMD reporting is to be inclusive, isolated nonspecific symptoms, which might (or might not) represent the above diagnoses, should be captured as AEs but not reported as pIMDs until the diagnosis can be defended.

10.6 Clinical Management of Adverse Events

Adverse events (AEs) will be managed by the clinical study team who will assess, provide first line of care as appropriate and refer to health care and treatment facilities as warranted. If any treatment/medical care is required as a result of the harm caused by the investigational product or study procedures, this will be provided free of charge.

If a participant has an AE and/or abnormal laboratory value that is known at the time of IV infusion of investigational product, the specifications of Section 12.0 will be followed.

Participants will be followed until the AE resolves or stabilizes or up to the end of the study, whichever comes last. If at the end of the study, an AE (including clinically significant laboratory abnormality) that is considered possibly, probably or definitely related to the investigational product is unresolved, follow-up will continue until resolution if possible and/or the participant will be referred.

If a participant from Group 3 experiences a significant decrease in CD4 cell count (e.g., – 20% of baseline, or decline to <200 cells/μL) during the course of the trial, participants

will be monitored closely until their CD4 count returns to baseline or until the participant initiates ART. Participants whose CD4 cell counts decrease to <200 cells/µL will be promptly informed and will be referred to their primary HIV care provider. Appropriate prophylaxis against opportunistic infections will be instituted according to accepted U.S. HIV treatment guidelines.

10.7 Clinical Management of Infusion-Related Reactions and Stopping Criteria

Infusion-related reactions can be local or systemic. Depending on the severity of the reaction, administration of IP can continue, be paused or stopped. Use clinical judgement and follow site-specific SOPs for anaphylaxis / infusion-related reactions. At a minimum, the following medications–or equivalent–should be available for immediate use during the infusion: 25 mg IV diphenhydramine x 1, 125 mg IV methylprednisolone x 1, epinephrine (1:1000) 0.5 mg/ 0.5 mL IV x 1, albuterol inhaler, and albuterol nebulizer. These medications should be included with the prescription for IP, if necessary (some sites may stock these medications on site already), so that they are available at the bedside to be administered consistent with site SOPs for anaphylaxis / infusion-related reactions.

For infusion related reactions, including cytokine release syndrome, use the Common Terminology Criteria for Adverse Events (CTCAE), Version 4.03 June 2010 grading scale.

Infusion stopping criteria:

Grade	
1	Monitor participant closely, intervention not indicated
2	Stop and re-start if clinically acceptable
3	Stop, do not re-start
4	Stop, do not re-start

Mild reaction (Grade 1)

- Inform medical staff
- Monitor volunteer for signs of systemic reaction. If there is no sign of systemic reaction continue the infusion of IP at the same rate
- Continue to monitor volunteer, manage volunteer according to local guidelines and SOPs
- Document the events
- Do not continue the infusion of any IP (1st or 2nd or 3rd) if there is any concern
- If infusion of 1st or 2nd IP is not continued, do not proceed with infusion of 2nd or 3rd IP, respectively

Systemic reaction (Grade 2)

- Stop administration of IP at the catheter level, i.e., as close to the arm as possible to prevent any more IP from entering the body. Remember that there is IP in the intravenous line downstream from the IP IV saline bag (removing the IP IV saline bag and replacing with a 0.9% saline bag or another IV fluid on the 1st intravenous line will result in additional administration of the several ml of IP that is in the line).
- Inform medical staff
- Monitor volunteer and continue to monitor until the volunteer is stable

This copy of the document was retrieved from the system by Lisa Sunner on 24 Apr 2019

- Ensure that 0.9% saline is running on the 2nd intravenous line
- The Principal Investigator, or study physician on call, must decide whether to restart or stop the administration of IP
    - If the volunteer improves discuss restarting infusion of IP, and at what rate, with Principal Investigator, or study physician on call.
    - If the volunteer does not improve or condition becomes worse, commence basic resuscitation according to site guidelines and SOPs and administer intravenous methylprednisolone and intravenous diphenhydramine. Replace the intravenous line connected to the 1st intravenous catheter (these lines contain IP).
- Document the events

Cytokine Release Syndrome or anaphylactic or anaphylactoid reaction (Grade 3 or 4)

- Stop administration of IP at the catheter level, i.e., as close to the arm as possible to prevent any more IP from entering the body. Replace the intravenous line connected to the 1st intravenous catheter (these lines contain IP).
- Call for assistance
- Monitor volunteer and continue to monitor until the volunteer is stable
- Ensure that 0.9% saline is running on the 2nd intravenous line
- Commence management of anaphylaxis according to site guidelines and SOPs
- Document the events

10.8 Pregnancy

Although not considered an AE, if a female participant becomes pregnant during the study, it is the responsibility of the Principal Investigator or designee to report the pregnancy promptly to IAVI using the designated forms. The participant will be followed for safety until the end of pregnancy or study completion, whichever occurs last. If possible, approximately 2–4 weeks after delivery, the baby will be examined by a physician to assess its health status and the results will be reported to the Sponsor. The baby will be examined again by a Physician around age 1, and the results will be reported to the Sponsor.

Complications of pregnancy that meet criteria for SAEs, specified in Section 10.4 of this Protocol (e.g., hospitalization for eclampsia, spontaneous abortion, etc.) should be reported as SAEs.

10.9 Intercurrent HIV Infection (Group 1 and 2)

HIV infection cannot be directly caused by the investigational product. If a participant acquires HIV through exposure in the community, at any time after the IV infusion of investigational product, the participant should be offered referral to appropriate care and treatment facilities. The participant will continue to be followed in the study for safety assessments.

Intercurrent HIV infection in study participants, although not considered an SAE, must be reported promptly to IAVI using the designated forms. However, medical conditions associated with the HIV infection that meet criteria for being serious specified in the Section 10.4 of this Protocol (e.g., sepsis, *Pneumocystis jiroveci [carinii] pneumonia*, etc.) should be reported as SAEs using the SAE Report Form.

TMF-03635 | 4.0 | Status:Approved | 16 Apr 2019
This copy of the document was retrieved from the system by Lisa Sunner on 24 Apr 2019

11.0 MANAGEMENT OF HIV ISSUES DURING AND FOLLOWING STUDY

11.1 HIV Testing – Groups 1 and 2

Group 1 and 2 participants will be tested for HIV antibodies as indicated in the Schedule of Procedures (Appendix A and B) or as needed, if medical or social circumstances arise. All participants will receive HIV risk reduction counselling and pre- and post-HIV-test counselling, as specified in Section 11.3.1, Counselling (Group 1 and 2).

It is possible that the Investigational Product(s) or an immune response to the Investigational Product(s) could cause a positive result on a diagnostic HIV antibody test. An investigational product recipient who falsely tests HIV positive with a diagnostic HIV antibody test at the end of the study will be informed of his/her positive test result and offered continuing follow-up until the test becomes negative.

If a participant acquires HIV through exposure in the community, at any time after the administration of investigational product, the participant will be offered referral to appropriate care and treatment facilities. The participant will continue to be followed in the study for safety assessments.

Should a participant require HIV testing outside of the study for personal reasons, it is recommended that the participant contact the study staff first. HIV testing can be done at the study site and then processed at an independent laboratory as above. Written evidence of HIV status (HIV-infected or HIV-uninfected) will be provided upon request.

11.2 Social Discrimination as a Result of investigational product-related antibodies

In order to minimize the possibility of social discrimination in participants (if any) who test positive on a diagnostic HIV antibody test due to investigational product-related antibodies, appropriate diagnostic HIV testing and certification will be provided both during and after the study as needed.

11.3 HIV infection – Group 1 and 2

Group 1 and 2 participants who are diagnosed with HIV infection at screening or during the study (intercurrent HIV-infection) will be provided the following:

11.3.1 Counseling

The participant will be counselled by the study investigators or designated counsellors. The counselling process will assist the participant with the following issues:

- Psychological and social implications of HIV infection
- Who to inform and what to say
- Implications for sexual partners
- Implications for child-bearing
- Avoidance of transmission to others in future
- Mandatory reporting to the state, in some instances

TMF-03635 | 4.0 | Status:Approved | 16 Apr 2019
This copy of the document was retrieved from the system by Lisa Sunner on 24 Apr 2019

11.3.2 Referral for Support/Care

Participants will be referred to a participant support center or institution of his/her choice for a full discussion of the clinical aspects of HIV infection. Referral will be made to a designated physician or center

TMF-03635 | 4.0 | Status:Approved | 16 Apr 2019
This copy of the document was retrieved from the system by Lisa Sunner on 24 Apr 2019

12.0 WITHDRAWAL FROM STUDY

12.1 Deferral of IV infusion of investigational product

An IV infusion of investigational product may be temporarily deferred if the participant is clinically ill at the time of the administration of investigational product visit and/or presents with fever (>100.4°F; >38.0°C) at the time of the administration of investigational product. A participant must be clinically well and afebrile for a minimum of a 24-hour consecutive period prior to administration of investigational product.

Any planned or unplanned deferral of infusion of investigational product will be discussed with the Sponsor. Participants will be deferred from infusion of investigational product for any of the following reasons:

- Pregnancy
- A disease or condition or adverse event that may develop, regardless of relationship to Investigational Product, if the Principal Investigator or designee is of the opinion that administration of investigational product will jeopardize the safety of the participant
- Participant's request to defer infusion

The following events require resolution and/or review of clinical history by the Principal Investigator or designee and consultation with the Medical Monitor, prior to administration of investigational product:

- Any abnormal laboratory value, as outlined in section 5.7, Exclusion Criteria, Hematology, Chemistry, Urinalysis that is known at the time of infusion and have not resolved.
- Receipt of inactivated/killed/subunit vaccines (non-HIV) or immunoglobulin within the previous 14 days. Receipt of live attenuated vaccines within the previous 30 days.
- Participating in another clinical study of an Investigational Product

12.2 Withdrawal from the Study (Early Termination)

Participants may be withdrawn from the study permanently for the following reasons:

1. Participants may withdraw from the study at any time if they wish, for any reason
2. The Principal Investigator or designee has reason to believe that the participant is not complying with the protocol
3. If the Sponsor decides to terminate or suspend the study

If a participant withdraws or is withdrawn from the study, all termination visit procedures will be performed according to the Schedule of Procedures (Appendices A, B and C) where possible. Every effort will be made to determine and document the reason for withdrawal.

This copy of the document was retrieved from the system by Lisa Sunner on 24 Apr 2019

13.0 DATA HANDLING

13.1 Data Collection and Record Keeping at the Study Site

Data Collection: All study data will be collected by the clinical study staff using designated source documents and entered onto the appropriate electronic CRFs (eCRFs). Access to eCRFs will be provided via an electronic data entry system hosted by the Data Coordination Center. All study data must be verifiable to the source documentation. A file will be held for each participant at the clinic(s) containing all the source documents. Source documentation will be available for review to ensure that the collected data are consistent with the CRFs.

All CRFs and laboratory reports will be reviewed by the clinical team, who will ensure that they are accurate and complete.

Source documents and other supporting documents will be kept in a secure location. Standard GCP practices will be followed to ensure accurate, reliable and consistent data collection.

Source documents include but are not limited to:

- Signed Informed Consent Documents
- Progress notes
- Data collection forms
- Documentation of any existing conditions or past conditions relevant to eligibility
- Printed laboratory results
- Print out of the generated enrollment confirmation
- All Adverse Events
- Concomitant medications
- Local and systemic reactogenicity events

13.2 Data Entry at the Study Site

The data collected at the site will be recorded onto the eCRFs by the study staff and entered into a database. To provide for real time assessment of safety, data should be entered as soon as reasonably feasible after a visit occurs.

13.3 Data Analysis

The Sponsor, PIs and Product Developers will agree on how data will be analyzed and presented prior to unblinding.

The DCC will conduct the data analysis and will provide interim safety and final study reports for the Sponsor, Principal Investigators, the PSRT and SMC and the regulatory authorities, as appropriate.

TMF-03635 | 4.0 | Status:Approved | 16 Apr 2019
This copy of the document was retrieved from the system by Lisa Sunner on 24 Apr 2019

14.0 STATISTICAL CONSIDERATIONS

14.1 Safety and Tolerability Analysis

14.1.1 Sample Size

The sample size for safety and tolerability analysis will be 30-66 participants according to the dose escalation design used to characterize the safety profile of one IV infusion of PGDM1400 mAb + PGT121 mAb ± VRC07-523LS mAb, at one of 4 dose levels.

14.1.2 Null Hypothesis

As this is an exploratory proof of concept trial and analysis will be descriptive, no formal null hypothesis will be tested.

14.1.3 Statistical Power and Analysis and Dose Escalation Rules

The frequency (percentage) of moderate or greater local and systemic reactogenicity events (along with 95% confidence intervals) will be determined for each active Group and placebo.

The frequency of SAEs judged possibly, probably or definitely related to the investigational product will be determined.

All AEs will be analyzed and grouped by seriousness, severity and relationship to the investigational product (as judged by the investigator).

For life-threatening adverse events related to investigational product: if none of the 18 (max 36) participants receiving active product experience such reactions, then the exact 95 % upper confidence bound for the rate of these adverse events in the population is 18.5% (or 9.7% if n=36).

All AEs will be analysed and grouped by seriousness, severity and relationship to the investigational product (as judged by the investigator).

For life-threatening adverse events related to active product: if none of the 9 (max 18) participants in either Group 1 or Group 2 who receive the active product experience such reactions then the exact 95% upper confidence bound for the rate of these adverse events in the population is 33.6% (or 18.5% if n=18).

An interim analysis of Subgroup and Group data will be carried out according to the study schema (Table 5.3.1) without unblinding the study to investigators or participants. At the end of the study, a full analysis will be prepared.

Based on previous experience with IAVI Phase 1 investigational product studies, it is expected that the amount of missing, unused or spurious data will be insignificant. Unused and spurious data will be listed separately and excluded from the statistical analysis. Missing data will be considered missing completely at random and excluded from the statistical analysis.

This copy of the document was retrieved from the system by Lisa Sunner on 24 Apr 2019

14.2 Pharmacokinetic Analysis

14.2.1 Sample Size

The sample size for pharmacokinetic analysis will be 3 per dose Subgroup, to provide sufficient information for the planned analyses.

14.2.2 Null Hypothesis

As this is an exploratory proof of concept trial and analysis will be descriptive, no formal null hypothesis will be tested.

14.2.3 Statistical Power and Analysis

Disposition of PGDM1400 mAb, PGT121 mAb and VRC07-523LS mAb will be evaluated in this study. Based on the PK profile of other human monoclonal antibodies, it is expected that the half-life of PGDM1400 mAb and PGT121 mAb will be 14 to 21 days, and the preliminary half life of VRC07-523LS is suggested to be ~33 days. Previously published data indicate that the pharmacokinetics of VRC01, 3BNC117 and 10-1074 are fairly similar across phase 1 studies (Table).

Table 14.2.3-1: Key parameters of select Antibodies for the Prevention and Treatment of HIV Infection

	3BNC117 (Caskey, Klein et al. 2015)	VRC01 (Ledgerwood, Coates et al. 2015, Lynch, Boritz et al. 2015)	10-1074 (Caskey, Schoofs et al. 2017)
**Binding site on HIV ENV spike**	CD4 binding site	CD4 binding site	V3 loop
**Human safety data available**	Yes, safe and well tolerated	Yes, safe and well tolerated	Yes, safe and well tolerated
**PK HIV-uninfected, terminal half life (days)**	17	15	24
**PK HIV-infected & viremic, terminal half life (days)**	9	12 (i.v.), 11 (s.c.)	12.8
**Antiviral effect (average or range log_{10} decrease HIV RNA)**	1.48	1.1-1.8	1.52

Commonly reported PK parameters will be calculated using, but not limited to, standard non-compartmental slope/height/area/moment (SHAM) analysis methods. Summary descriptive results of PK parameters, including AUC, Cmax, T1/2, and clearance results will be reported by dose cohort. Dose normalized plots of PK parameters will be presented. Correlation between PK and reported safety and pharmacodynamic outcomes will also be explored parameters in order to examine exposure-effect relationships.

A more powerful exploratory analysis to quantitatively determine the dose, participant and disease impact on PGDM1400 mAb, PGT121 mAb and VRC07-523LS mAb pharmacokinetics, and correlate exposure with response, while correctly accounting for variance based on population intrinsic factors such as weight and gender will be performed. Using the proposed population analysis approach we will be able to simultaneously examine the magnitude and the rate of change to PGDM1400, PGT121

This copy of the document was retrieved from the system by Lisa Sunner on 24 Apr 2019

and VRC07-523LS disposition driven by HIV-1 RNA levels, and also examine the magnitude and the rate of decline in log copies/ml of HIV-1 RNA plasma levels from baseline.

The frequency and levels of anti-PGDM1400 antibodies, anti-PGT121 antibodies and anti-VRC07-523LS antibodies will be calculated and tabulated.

14.3. Virologic Analysis for Group 3

14.3.1 Sample Size

The sample size for virologic analysis in Groups 3A and 3B will be 6-18 participants according to the design described below.

14.3.2 Null Hypothesis

The null hypothesis is that there is a mixture of responders and non-responders with mixture probability 0.5 such that the difference-from-baseline is greater than -0.9 logs HIV RNA viral load in the responder group and 0 logs HIV RNA viral load in the non-responder group.

14.3.3 Statistical Power and Analysis

The virologic analysis described in this section relates to Subgroups 3A and 3B of the study design, in which antiviral activity of PGDM1400 mAb in combination with PGT121 and PGDM1400 mAb in combination with PGT121 mAb and VRC07-523LS mAb is measured in HIV-infected participants off ART with plasma HIV RNA levels of 1×10^3 – 1×10^5 copies/ml. This section assumes i) that Part 1 of the study has successfully demonstrated that there are safe dose levels of PGDM1400 mAb + PGT121 mAb, and ii) that PGDM1400 mAb + PGT121 mAb + VRC07-523LS mAb can be administered safely at 20 mg/kg. No placebo participants are enrolled as part of this design.

The primary efficacy outcome for this analysis is defined as change in log10 viral load between Day 0 (day of infusion) and Day 7. The minimum clinically significant value for this outcome is defined as a difference of –0.9 log10.

Group 3A: this Group will enroll participants sequentially. After administering PGDM1400 + PGT121 + VRC07-523LS at 20 + 20 + 20 mg/kg, the day 7 post-infusion viral load measurement will be compared to the baseline viral load measurement. If the difference in viral load at 7 days post-infusion is greater than a 0.5 log10 drop, the participant is categorized as a responder; otherwise, the participant is categorized as a non-responder. Enrollment will continue until at least 3 responders or 9 total participants have been enrolled whichever occurs first.

Group 3B: this Group will enroll participants sequentially. After administering PGDM1400 mAb and PGT121 mAb at the MTD, as defined in part 1 of this study, the day 7 post-infusion viral load measurement will be compared to the baseline viral load measurement. If the difference in viral load at 7 days post-infusion is greater than a 0.5 log10 drop, the participant is categorized as a responder; otherwise, the participant is categorized as a non-responder. Enrollment will continue until at least 3 responders or 9 total participants have been enrolled whichever occurs first.

Antiviral activity in Subgroup 3A and 3B will be tested in the responder group only using a one-sided Wilcoxon signed rank test against the null hypothesis of a "shift" parameter

This copy of the document was retrieved from the system by Lisa Sunner on 24 Apr 2019

of -0.9 log_{10}. Based on a simulation study outlined in the SAP, the power to reject the null hypothesis is 80% when the responder group has a difference-from-baseline viral load drop of approximately 1.8 logs for a nominal alpha level of 0.05. Since the responder group is defined by the outcome it is not surprising that the Type I error is greater than the nominal alpha level which was deemed an acceptable trade-off between sample size and power to detect the desired effect under the alternative described above. Notably, the Type I error drops below the nominal error rate when the true shift in viral load is in the responder group is greater than -0.5 logs.

For the analysis of sample size and power, log10 viral load differences from baseline for each participant were simulated from a normal distribution, with a standard deviation of 0.5. This value was chosen by examining a study of the antiretroviral drug raltegravir, which demonstrated a mean estimated standard deviation of the change of baseline of 0.47(Andrade, Rosenkranz et al. 2013). This is a conservative estimate, as the variability of viral loads near the lower range might be expected to also be lower.

The statistical test performed will be the Signed-ranktest, which will incorporate the "shift" parameter of –0.9 log10 (the minimum clinically significant difference selected for this study). An evaluation of potential harm (increased viral load) will also be performed with the Signed ranktest; this test will examine the null hypothesis of no change in viral load (a shift of 0.0 log10 following investigational product administration) against the one-sided alternative hypothesis that the viral load is increased following investigational product administration. Each efficacy test will be performed at the level $\alpha = 0.05$. Each test for harm will be performed at level $2\alpha = 0.10$, in order to provide additional sensitivity to detect potential harm.

14.4 Secondary and Exploratory Immunologic and Virologic Analyses

14.4.1 Sample Size

The sample size for secondary immunologic and/or virologic analysis will be the 6-18 HIV infected participants in part 2 of the study.

14.4.2 Null Hypothesis

No formal hypothesis on immunologic or virologic responses will be tested, with the exception of the change in HIV viral load described in Section 14.3.

14.4.3 Statistical Power and Analysis

Descriptive statistics (actual values and changes from reference) will be calculated for continuous immunologic and virologic parameters at all time points. Graphical representations of changes in parameters will be made as applicable. Differences between Subgroups and/or Groups at a specific time point will be tested for exploratory purposes by a 2-sample t-test if the data appear to be normally distributed (after transformation if necessary). If not, the non-parametric Wilcoxon rank sum test will be used. If portions of the measurements are censored below the assay quantification limit, the Gehan-Wilcoxon test will be employed. All statistical tests will be two-sided and will be considered statistically significant if $p < 0.05$.

Frequency tabulations will be calculated for discrete (qualitative) immunologic and virologic parameters at all time points. Significant differences between Subgroups and/or Groups will be determined by a 2-sided Fisher's exact test.

This copy of the document was retrieved from the system by Lisa Sunner on 24 Apr 2019

Interim immunologic and virologic analyses of grouped data may be performed without unblinding the study to investigators or participants.

15.0 QUALITY CONTROL AND QUALITY ASSURANCE

To ensure the quality and reliability of the data collected and generated and the ethical conduct of this study, a Study Operations Manual (SOM) will be developed. All deviations will be reported and investigated. The SOM describes reporting and deviation documentation requirements and procedures.

Regular monitoring will be performed according to ICH-GCP as indicated in Section 17.5.

An independent audit of the study and study sites may be performed by the Sponsor or designee to establish the status of applicable quality systems. Inspection by regulatory authorities may also occur.

By signing the protocol, the Principal Investigators agree to facilitate study related monitoring, audits, IRB/IEC review and regulatory inspection(s) and direct access to source documents. Such information will be treated as strictly confidential and under no circumstances be made publicly available.

16.0 DATA AND BIOLOGICAL MATERIAL

All data and biological material collected through the study shall be managed in accordance with the Clinical Trial Agreement (CTA). Distribution and use of these data will be conducted by agreement of all parties.

The computerized raw data generated will be held by the DCC on behalf of the Sponsor. The study sites will also hold the final data files and tables generated for the purpose of analysis.

TMF-03635 | 4.0 | Status:Approved | 16 Apr 2019
This copy of the document was retrieved from the system by Lisa Sunner on 24 Apr 2019

17.0 ADMINISTRATIVE STRUCTURE

The Principal Investigator will be responsible for all aspects of the study at the study site.

17.1 Protocol Safety Review Team

A PSRT will be formed to monitor the clinical safety data. During the administration of investigational productphase of the trial, the PSRT will review the clinical safety data on a weekly basis via electronic distribution of reports. An ad hoc PSRT review meeting will occur if any of the members of the PSRT requests a special review to discuss a specific safety issue or as specified in the Study Operations Manual. After the administration of investigational product phase the PSRT will review the clinical safety data at least monthly.

The PSRT will consist of the IAVI Medical Monitor(s), and the PI or designee from each clinical team. The study chair or an IAVI Medical Monitor may be the PSRT chair. *Ex officio* members will include the IAVI Chief Medical Officer and an unblinded IAVI Medical Monitor. Additional PSRT participants may include the following, as needed:

- Co-investigators and trial site senior clinical research nursing staff
- Laboratory directors
- Data management, study statistician and regulatory staff

The PSRT membership and procedures are detailed in the PSRT charter.

17.2 Safety Monitoring Committee (SMC)

The SMC will consist of independent clinicians/scientists/statisticians/ethicists who are not involved in the study. Investigators responsible for the clinical care of participants or representative of the Sponsor may not be a member of the SMC. Details of membership, chair and co-chair and responsibilities are outlined in the SMC charter.

Principal Investigator(s) or designee and/or a Sponsor representative may be asked to join an open session of the SMC meeting to provide information on study conduct, present data or to respond to questions.

Safety data will be reviewed by the SMC at pre-specified time points and at an ad-hoc basis.

17.2.1 Content of Interim Safety Review

The SMC will be asked to review the following blinded data:

- Summary of reactogenicity (i.e., solicited adverse events)
- All adverse events judged by the Principal Investigator or designee to be possibly, probably or definitely related to investigational product
- All laboratory results confirmed on retest and judged by the Principal Investigator or designee to be clinically significant
- All SAEs and pIMDs

An unblinded presentation of all above noted events may also be made available for the SMC for their review if required by any member of the SMC.

This copy of the document was retrieved from the system by Lisa Sunner on 24 Apr 2019

17.2.2 SMC Review of Group 1 and 2 data prior to starting Group 3

Following IV infusion of investigational product of the last participant in Groups 1 and/or 2, the Safety Monitoring Committee (SMC) will review safety data through the day 14 post-IV infusion visit for all participants to confirm MTD in each Group, and determine whether, and at what dose level, Groups 3A and 3B can initiate enrollment. See section 5.3 for additional details.

17.3 Criteria for Pausing the Study

Enrollment and administration of investigational product will be stopped and a safety review conducted by the SMC for any of the following criteria:

- One or more participants experience an SAE that is judged possibly, probably or definitely related to investigational product.
- There is a participant death, regardless of relationship to the investigational product.
- Two or more participants experience Grade 3 adverse events in the same category System Organ Class that are considered possibly, probably or definitely related to investigational product or
- Any Grade 4 adverse event that is considered possibly, probably or definitely related to investigational product.

This copy of the document was retrieved from the system by Lisa Sunner on 24 Apr 2019

Table 17.3-1: AE notification and safety pause/AE review rules

Event and relationship to study product	Severity	Occurrence	Site PI action	PSRT or SMC action
SAE, possibly, probably or definitely related	Any	Any	Phone, email or fax forms to sponsor within 24 hours	Study pause within 24 hours, refer to SMC for review
SAE, probably not or not related	Death	Any	Phone, email or fax forms to sponsor within 24 hours	Study pause within 24 hours, refer to SMC for review
AE, possibly, probably or definitely related	Grade 4	Any	Phone, email or fax notification to sponsor within 24 hours	Study pause within 24 hours, refer to SMC for review
AE¶, possibly, probably or definitely related	Grade 3*	First	Phone, email or fax notification to sponsor within 24 hours	PSRT review within 2 business days to consider pause
AE¶, possibly, probably or definitely related	Grade 3*	Second‡	Phone, email or fax notification to sponsor within 24 hours	Study pause within 24 hours, refer to SMC for review

¶Does not include the following reactogenicity symptoms (fever, malaise, myalgia, arthralgia, chills, headache, nausea, vomiting).

*If no evidence of disease is present other than an abnormal laboratory value, the test must be repeated with a new blood sample at least one time within 72 hours after the investigator becoming aware of the abnormal laboratory value. When signs and symptoms are present, repeat test will not be needed.

‡PSRT will determine whether the reported related AE (Grade 3) means that 2 or more participants experience Grade 3 adverse events in the same category System Organ Class that are considered possibly, probably or definitely related to investigational product.

The Sponsor will request a review by the SMC, (or the SMC chair if other SMC members cannot be convened), to be held within 2 business days of the Sponsor learning of the event. The individual participant(s)/or study may be unblinded at the discretion of the SMC. Following this review, the SMC will make a recommendation regarding the continuation or suspension of the administration of the investigational product or the trial and communicate this decision immediately to the Sponsor. The Sponsor then will inform the Principal Investigators without delay.

Additional *ad hoc* review may be specifically requested by the Sponsor, the Principal Investigator(s) or by the SMC.

17.4 Study Supervision

The SMC, the IAVI Chief Medical Officer (CMO) and the IAVI Medical Monitor(s) have access to progress report(s) of this study. Close cooperation will be necessary to track study progress, respond to queries about proper study implementation and management, address issues in a timely manner, and assure consistent documentation, and share information effectively. Rates of accrual, retention, and other parameters

This copy of the document was retrieved from the system by Lisa Sunner on 24 Apr 2019

relevant to the site's performance will be regularly and closely monitored by the study team.

17.5 Study Monitoring

On-and/or off-site monitoring will ensure that the study is conducted in compliance with human subjects' protection and other research regulations and guidelines, recorded and reported in accordance with the protocol, is consistent with SOPs, GCP, applicable regulatory requirements and locally accepted practices. The monitor will confirm the quality and accuracy of data at the site by validation of CRFs against the source documents, such as clinical records. The investigators, as well as participants through consenting to the study, agree that the monitor may inspect study facilities and source records (e.g., informed consent forms, clinic and laboratory records, other source documents), as well as observe the performance of study procedures (in accordance with site IRB requirements). Such information will be treated as strictly confidential and will under no circumstances be made publicly available.

The monitoring will adhere to GCP guidelines. The Principal Investigator will permit inspection of the facilities and all study-related documentation by authorized representatives of IAVI, and Government and Regulatory Authorities responsible for this study.

17.6 Investigator's Records

Study records include administrative documentation—e.g., reports and correspondence relating to the study—as well as documentation related to each participant screened and/or enrolled in the study—including informed consent forms, case report forms, and all other source documents. The investigator will maintain and store, in a secure manner, complete, accurate, and current study records for a minimum of 2 years after marketing application approval or the study is discontinued and applicable national and local health authorities are notified. IAVI will notify the Principal Investigator of these events.

This copy of the document was retrieved from the system by Lisa Sunner on 24 Apr 2019

18.0 INDEMNITY

The Sponsor and Institution are responsible to have appropriate liability insurance. For research-related injuries and/or medical problems determined to result from receiving the investigational product, treatment including necessary emergency treatment and proper follow-up care will be made available to the participant free of charge at the expense of the Sponsor.

19.0 PUBLICATION

A primary manuscript describing safety, anti-viral effect and immune responses in this trial will be prepared promptly after the data analysis is available.

Authors will be representatives of each trial site, the data management and statistical analysis center, the laboratories, the product developer and the sponsor, participant to the generally accepted criteria of contributions to the design and conduct of the study, the analysis of data and writing of the manuscript. Precedence will be given to authors from the site enrolling the greatest number of participants. Manuscripts will be reviewed by representatives of each participating group as specified in the CTA**.**

20.0 ETHICAL CONSIDERATIONS

The Principal Investigator will ensure that the study is conducted in compliance with the protocol, SOPs in accordance with guidelines formulated by the ICH for GCP in clinical studies, the ethical principles that have their origins in the Declaration of Helsinki and applicable local standards and regulatory requirements.

TMF-03635 | 4.0 | Status:Approved | 16 Apr 2019
This copy of the document was retrieved from the system by Lisa Sunner on 24 Apr 2019

APPENDIX A: SCHEDULE OF PROCEDURES – GROUP 1 (A, B, C)

**Study Month**		**0**							**1**		**2**		**3**	**4**	**5**	**6**
**Study Week**		**0**				**1**	**2**	**3**	**4**	**6**	**8**	**10**	**12**	**16**	**20**	**24**
**Study Day**	**Scr**	**0**	**1**	**2**	**3**	**7**	**14**	**21**	**28**	**42**	**56**	**70**	**84**	**112**	**140**	**168/ET^**
**Visit Windows (Days)**	**-56**	**0**	**0**	**0**	**0**	**± 1**	**± 2**	**± 2**	**± 2**	**± 3**	**± 3**	**±3**	**±7**	**±7**	**±7**	**±7**
**INVESTIGATIONAL PRODUCT**																
Investigational Product		X														
**CONSENT/ASSESSMENTS/COUNSELLING**																
Informed Consent	X															
Assessment of Understanding	X															
HIV Risk Assessment	X															X
HIV Risk Reduction Counselling	X	X							X		X		X	X	X	X
HIV-test Counselling	X	X							X							X
Family Planning Counselling	X	X														
Social Impact Assessment																X
**CLINICAL SAFETY ASSESSMENTS**																
Comprehensive Medical History	X															
Interim Medical History		X	X	X	X	X	X	X	X	X	X					
Concomitant Medications	X	X	X	X	X	X	X	X	X	X	X					
General Physical Exam	X															X
Directed Physical Exam		X	X	X	X	X	X	X	X	X	X	X	X	X	X	
Weight	X	X														X
Height	X															
Vital Signs	X	X*	X	X	X	X	X	X	X	X	X	X	X	X	X	X
Local & Systemic Reactogenicity		X*	X	X	X											
Adverse Events		X	X	X	X	X	X	X	X	X	X					
Serious Adverse Events and pIMD	X	X	X	X	X	X	X	X	X	X	X	X	X	X	X	X
**CLINICAL LABORATORY TESTS**																
Hematology and Coagulation, Clinical Chemistry	X	X#	X		X	X	X		X		X		X	X	X	X
Urine Dipstick	X	X#	X		X	X	X		X		X		X	X	X	X
Urine Pregnancy test	X	X#							X		X		X			X
Active Syphilis, Chlamydia, Gonorrhea, Hepatitis B, Hepatitis C	X															
HIV screen (4th generation Ag/Ab test)	X															
Blinded HIV diagnostic testing		X#							X							X
**RESEARCH LABORATORY TESTS**																
Anti PGDM1400 and anti-PGT121 Antibodies (ADA)		X#							X		X		X			X
Humoral Assays**		X#			X	X	X		X		X		X			X
Cellular Assays**		X#					X		X		X		X			X
HLA typing		X#														

This copy of the document was retrieved from the system by Lisa Sunner on 24 Apr 2019

Study Month		0							1		2		3	4	5	6
Study Week		0				1	2	3	4	6	8	10	12	16	20	24
Study Day	Scr	0	1	2	3	7	14	21	28	42	56	70	84	112	140	168/ET^
Visit Windows (Days)	-56	0	0	0	0	± 1	± 2	± 2	± 2	± 3	± 3	±3	±7	±7	±7	±7
PHARMACOKINETICS PGDM1400 ± PGT121 ELISA		X#	X	X	X	X	X	X	X	X	X	X	X	X	X	X
MUCOSAL SAMPLING		X~	X			X	X									
PLASMA/SERUM STORAGE		X	X	X	X	X	X	X	X		X		X			X
PBMC STORAGE		X									X		X			X

Day 0 baseline sample collections must be done before infusion of investigational product. Additional day 0 pharmacokinetics sample collection will be done as outlined in Protocol section 9.4.2.
^ Early Termination (ET): Procedures to be performed at ET are the same as last visit procedures
* At baseline, approximately every 30 minutes for the first 4 hours after IP administration, and then every hour until 6 hours after IV infusion. Local and systemic reactogenicity will be assessed by clinic staff at visits on study days 1, 2 and 3. Local and Systemic reactogenicity will also be assessed by the participant using the Memory Aid on study days 1, 2, and 3.
** See Laboratory Analytical Plan for details
~ Cervico-vaginal and/or rectal mucosal sampling (optional) on Day 0 must be done prior to IV infusion of IP.

TMF-03635 | 4.0 | Status:Approved | 16 Apr 2019
This copy of the document was retrieved from the system by Lisa Sunner on 24 Apr 2019

APPENDIX B: SCHEDULE OF PROCEDURES – AND GROUP 2 (A, B, C)

Study Month		0							1		2		3	4	5	6
**Study Week**		**0**				**1**	**2**	**3**	**4**	**6**	**8**	**10**	**12**	**16**	**20**	**24**
**Study Day**	**Scr**	**0**	**1**	**2**	**3**	**7**	**14**	**21**	**28**	**42**	**56**	**70**	**84**	**112**	**140**	**168/ET^**
**Visit Windows (Days)**	**-56**	**0**	**0**	**0**	**0**	**± 1**	**± 2**	**± 2**	**± 2**	**± 3**	**± 3**	**±3**	**±7**	**±7**	**±7**	**±7**
**INVESTIGATIONAL PRODUCT**																
Investigational Product		X														
**CONSENT/ASSESSMENTS/COUNSELLING**																
Informed Consent	X															
Assessment of Understanding	X															
HIV Risk Assessment	X															X
HIV Risk Reduction Counselling	X	X							X		X		X	X	X	X
HIV-test Counselling	X	X							X							X
Family Planning Counselling	X	X														
Social Impact Assessment																X
**CLINICAL SAFETY ASSESSMENTS**																
Comprehensive Medical History	X															
Interim Medical History		X	X	X	X	X	X	X	X	X	X					
Concomitant Medications	X	X	X	X	X	X	X	X	X	X	X					
General Physical Exam	X															X
Directed Physical Exam		X	X	X	X	X	X	X	X	X	X	X	X	X	X	
Weight	X	X														X
Height	X															
Vital Signs	X	X*	X	X	X	X	X	X	X	X	X	X	X	X	X	X
Local & Systemic Reactogenicity		X*	X	X	X											
Adverse Events		X	X	X	X	X	X	X	X	X	X					
Serious Adverse Events and pIMD	X	X	X	X	X	X	X	X	X	X	X	X	X	X	X	X
**CLINICAL LABORATORY TESTS**																
Hematology and Coagulation, Clinical chemistry	X	X#	X		X	X	X		X		X		X	X	X	X
Urine Dipstick	X	X#	X		X	X	X		X		X		X	X	X	X
Urine Pregnancy test	X	X#							X		X		X			X
Active Syphilis, Chlamydia, Gonorrhea, Hepatitis B, Hepatitis C	X															
HIV screen (4th generation Ag/Ab test)	X															
Blinded HIV diagnostic testing		X#							X							X
**RESEARCH LABORATORY TESTS**																
Anti PGDM1400 and anti-PGT121 Antibodies (ADA)		X#							X		X		X			X
Humoral Assays**		X#			X	X	X		X		X		X			X
Cellular Assays**		X#					X		X		X		X			X
HLA typing		X#														
**PHARMACOKINETICS PGDM1400 ± PGT121 ELISA**		X#	X	X	X	X	X	X	X	X	X	X	X	X	X	X
**MUCOSAL SAMPLING**		X~	X			X	X									

TMF-03635 | 4.0 | Status:Approved | 16 Apr 2019
This copy of the document was retrieved from the system by Lisa Sunner on 24 Apr 2019

Study Month		0							1		2		3	4	5	6
Study Week		0				1	2	3	4	6	8	10	12	16	20	24
Study Day	Scr	0	1	2	3	7	14	21	28	42	56	70	84	112	140	168/ET^
Visit Windows (Days)	-56	0	0	0	0	± 1	± 2	± 2	± 2	± 3	± 3	±3	±7	±7	±7	±7
PLASMA/SERUM STORAGE		X	X	X	X	X	X	X	X		X		X			X
PBMCs STORAGE		X									X		X			X

Day 0 baseline sample collections must be done before infusion of investigational product. Additional day 0 pharmacokinetics sample collection will be done as outlined in Protocol section 9.4.2.
^ Early Termination (ET): Procedures to be performed at ET are the same as last visit procedures
* At baseline, approximately every 30 minutes for the first 4 hours after IP administration and then every hour until 6 hours after IV infusion. Local and systemic reactogenicity will be assessed by clinic staff at visits on study days 1, 2 and 3. Local and Systemic reactogenicity will also be assessed by the participant using the Memory Aid on study days 1, 2, and 3.
** See Laboratory Analytical Plan for details
~ Cervico-vaginal and/or rectal mucosal sampling (optional) on Day 0 must be done prior to IV infusion of IP.

TMF-03635 | 4.0 | Status:Approved | 16 Apr 2019
This copy of the document was retrieved from the system by Lisa Sunner on 24 Apr 2019

APPENDIX C: SCHEDULE OF PROCEDURES – GROUP 3 (A, B)

**Study Month**		**0**								**1**		**2**		**3**	**4**	**5**	**6**
**Study Week**		**0**				**1**		**2**	**3**	**4**	**6**	**8**	**10**	**12**	**16**	**20**	**24**
**Study Day**	**Scr**	**0**	**1**	**2**	**3**	**7**	**10**	**14**	**21**	**28**	**42**	**56**	**70**	**84**	**112**	**140**	**168/ET**
**Visit Windows (Days)**	**-42**	**0**	**0**	**0**	**0**	**± 1**	**0**	**± 2**	**± 2**	**± 2**	**± 3**	**± 3**	**±3**	**±7**	**±7**	**±7**	**±7**
**INVESTIGATIONAL PRODUCT**																	
Investigational Product		X															
**CONSENT/ASSESSMENTS/COUNSELLING**																	
Informed Consent, Assessment of Understanding	X																
HIV Risk Reduction Counselling	X	X								X		X		X	X	X	X
ART counselling	X	X										X					X
Family Planning Counselling	X	X															
Social Impact Assessment																	X
**CLINICAL SAFETY ASSESSMENTS**																	
Comprehensive Medical History	X																
Interim Medical History		X	X	X	X	X	X	X	X	X	X	X					
Concomitant Medications	X	X	X	X	X	X	X	X	X	X	X	X					
General Physical Exam	X																X
Directed Physical Exam		X	X	X	X	X	X	X	X	X	X	X	X	X	X	X	
Weight	X	X															X
Height	X																
Vital Signs	X	X*	X	X	X	X	X	X	X	X	X	X	X	X	X	X	X
Local & Systemic Reactogenicity		X*	X	X	X												
Adverse Events		X	X	X	X	X	X	X	X	X	X	X					
Serious Adverse Events and pIMD	X	X	X	X	X	X	X	X	X	X	X	X	X	X	X	X	X
**CLINICAL LABORATORY TESTS**																	
Hematology and Coagulation, Clinical Chemistry	X	X#	X		X	X		X		X		X		X	X	X	X
CD4	X	X#				X		X		X		X					X
Urine Dipstick¶	X	X#¶	X¶		X¶	X¶		X¶		X¶		X¶		X¶	X¶	X¶	X¶
Urine Pregnancy test	X	X#								X		X		X			X
Active Syphilis, Chlamydia, Gonorrhea, Hepatitis B, Hepatitis C	X																
HIV 4th generation Ag/Ab test***	X																
HIV Viral Load	X	X#	X	X	X	X	X	X	X	X	X	X	X	X	X	X	X
**RESEARCH LABORATORY TESTS**	-	-	-	-	-	-	-	-	-	-	-	-	-	-	-	-	-
Anti PGDM1400 and anti-PGT121 and anti-VRC07-523LS Antibodies (ADA)		X#								X		X		X			X
HIV phenotypic testing for PGDM1400 ± PGT121 ± VRC07-523LS susceptibility	X									X							X
HIV SGA sequencing	X									X							X
HIV genotypic testing for ART resistance	X									X				X			X
HIV reservoir size assessment	X							X						X			

CONFIDENTIAL

**Study Month**		**0**								**1**		**2**		**3**	**4**	**5**	**6**
**Study Week**		**0**				**1**		**2**	**3**	**4**	**6**	**8**	**10**	**12**	**16**	**20**	**24**
**Study Day**	**Scr**	**0**	**1**	**2**	**3**	**7**	**10**	**14**	**21**	**28**	**42**	**56**	**70**	**84**	**112**	**140**	**168/ET**
**Visit Windows (Days)**	**-42**	**0**	**0**	**0**	**0**	**± 1**	**0**	**± 2**	**± 2**	**± 2**	**± 3**	**± 3**	**±3**	**±7**	**±7**	**±7**	**±7**
Humoral Assays**		**X#**			**X**	**X**		**X**		**X**		**X**		**X**			**X**
Cellular Assays**		**X#**						**X**		**X**		**X**		**X**			**X**
HLA typing		**X#**															
**PHARMACOKINETICS PGDM1400 ± PGT121 ± VRC07-523LS ELISA**	**X**	**X#**	**X**	**X**	**X**	**X**		**X**	**X**	**X**	**X**	**X**	**X**	**X**	**X**	**X**	**X**
**MUCOSAL SAMPLING**		**X~**	**X**			**X**		**X**									
**PLASMA/SERUM STORAGE**	**X**	**X**	**X**	**X**	**X**	**X**	**X**	**X**	**X**	**X**		**X**		**X**			**X**
**PBMCs STORAGE**		**X**										**X**		**X**			**X**

Day 0 baseline sample collections must be done before infusion of investigational product. Additional day 0 pharmacokinetics sample collection will be done as outlined in Protocol section 9.4.2.
^ Early Termination (ET): Procedures to be performed at ET are the same as last visit procedures
* At baseline, approximately every 30 minutes for the first 4 hours after IP administration and then every hour until 6 hours after IV infusion. Local and systemic reactogenicity will be assessed by clinic staff at visits on study days 1, 2 and 3. Local and Systemic reactogenicity will also be assessed by the participant using the Memory Aid on study days 1, 2, and 3.
** See Laboratory Analytical Plan for details
*** Confirmed HIV-1 infection (HIV Ab+ or HIV RNA+) by documentation in the medical records or in-clinic HIV testing;
~ Cervico-vaginal and/or rectal mucosal sampling (optional) on Day 0 must be done prior to IV infusion of IP.
¶ Urinalysis will only be conducted at visits after screening if clinically indicated.

TMF-03635 | 4.0 | Status:Approved | 16 Apr 2019
This copy of the document was retrieved from the system by Lisa Sunner on 24 Apr 2019

APPENDIX D: LOW RISK CRITERIA

Low risk will be defined as:

1. **SEXUAL BEHAVIORS**

In the **last 12 months** did not:

- Have oral, vaginal or anal intercourse with an HIV-infected partner, or partner who uses injection drugs.
- Gave or receive money, drugs, gifts, or services in exchange for oral, vaginal or anal sex

  AND

  In the **last 6 months** has abstained from penile/anal or penile/vaginal intercourse

  OR

  In the **last 6 months**:

- Had 4 or fewer partners of the opposite birth sex for vaginal and/or anal intercourse, OR

Is MSM (person born male with partner(s) born male) who, in the **last 12 months**:

- Had 2 or fewer MSM partners for anal intercourse and had no unprotected anal sex with MSM, OR
- Had unprotected anal intercourse with only 1 MSM partner, within a monogamous relationship lasting at least 12 months (during which neither partner had any other partners). If the monogamous relationship ended, the participant may then have had protected anal intercourse with 1 other MSM partner (total 2 or fewer partners in the last 12 months).

Is a transgender person, regardless of the point on the transition spectrum, having sex with men (born male) and/or other transgender persons, who in the last 12 months:

- Had 2 or fewer partners for anal or vaginal intercourse, and had no unprotected anal or vaginal sex, OR
- Had unprotected anal or vaginal intercourse sex with 1 partner only within a monogamous relationship lasting at least 12 months (during which neither partner had any other partners). If the monogamous relationship ended, may then have had protected anal or vaginal sex with one other partner (total 2 or fewer partners in the last 12 months).

  AND

  Uses or intends to use condoms in situations which may include penile/anal or penile/vaginal intercourse with new partners of unknown HIV status, occasional partners, partners outside a primary relationship, and/or partners known to have other partners.

2. **NON-SEXUAL BEHAVIORS**

   In the **last 12 months** did not:

- Inject drugs or other substances without a prescription
- Use cocaine, methamphetamine, or excessive alcohol, which in the investigator's judgement, rendered the participant at greater than low risk for acquiring HIV infection

This copy of the document was retrieved from the system by Lisa Sunner on 24 Apr 2019

The investigator's judgement should consider local epidemiologic information about HIV prevalence in the area and community networks.

A participant is NOT appropriate for inclusion if he/she:

Acquired an STI (i.e., new infection) in the last 12 months:

- Syphilis
- Gonorrhea
- Non-gonococcal urethritis
- HSV-2
- Chlamydia
- Pelvic inflammatory disease (PID)
- Trichomonas
- Mucopurulent cervicitis
- Epididymitis
- Proctitis
- Lymphogranuloma venereum
- Chancroid
- Hepatitis B

TMF-03635 | 4.0 | Status:Approved | 16 Apr 2019
This copy of the document was retrieved from the system by Lisa Sunner on 24 Apr 2019

APPENDIX E REFERENCES

Andrade, A., S. L. Rosenkranz, A. R. Cillo, D. Lu, E. S. Daar, J. M. Jacobson, M. Lederman, E. P. Acosta, T. Campbell, J. Feinberg, C. Flexner, J. W. Mellors, D. R. Kuritzkes and A. C. T. G. A. Team (2013). "Three distinct phases of HIV-1 RNA decay in treatment-naive patients receiving raltegravir-based antiretroviral therapy: ACTG A5248." J Infect Dis **208**(6): 884-891.

Bar, K. J., M. C. Sneller, L. J. Harrison, J. S. Justement, E. T. Overton, M. E. Petrone, D. B. Salantes, C. A. Seamon, B. Scheinfeld, R. W. Kwan, G. H. Learn, M. A. Proschan, E. F. Kreider, J. Blazkova, M. Bardsley, E. W. Refsland, M. Messer, K. E. Clarridge, N. B. Tustin, P. J. Madden, K. Oden, S. J. O'Dell, B. Jarocki, A. R. Shiakolas, R. L. Tressler, N. A. Doria-Rose, R. T. Bailer, J. E. Ledgerwood, E. V. Capparelli, R. M. Lynch, B. S. Graham, S. Moir, R. A. Koup, J. R. Mascola, J. A. Hoxie, A. S. Fauci, P. Tebas and T. W. Chun (2016). "Effect of HIV Antibody VRC01 on Viral Rebound after Treatment Interruption." N Engl J Med **375**(21): 2037-2050.

Barouch, D. H. and S. G. Deeks (2014). "Immunologic strategies for HIV-1 remission and eradication." Science **345**(6193): 169-174.

Barouch, D. H., J. B. Whitney, B. Moldt, F. Klein, T. Y. Oliveira, J. Liu, K. E. Stephenson, H. W. Chang, K. Shekhar, S. Gupta, J. P. Nkolola, M. S. Seaman, K. M. Smith, E. N. Borducchi, C. Cabral, J. Y. Smith, S. Blackmore, S. Sanisetty, J. R. Perry, M. Beck, M. G. Lewis, W. Rinaldi, A. K. Chakraborty, P. Poignard, M. C. Nussenzweig and D. R. Burton (2013). "Therapeutic efficacy of potent neutralizing HIV-1-specific monoclonal antibodies in SHIV-infected rhesus monkeys." Nature **503**(7475): 224-228.

Burton, D. R. and J. R. Mascola (2015). "Antibody responses to envelope glycoproteins in HIV-1 infection." Nat Immunol **16**(6): 571-576.

Caskey, M., F. Klein, J. C. Lorenzi, M. S. Seaman, A. P. West, Jr., N. Buckley, G. Kremer, L. Nogueira, M. Braunschweig, J. F. Scheid, J. A. Horwitz, I. Shimeliovich, S. Ben-Avraham, M. Witmer-Pack, M. Platten, C. Lehmann, L. A. Burke, T. Hawthorne, R. J. Gorelick, B. D. Walker, T. Keler, R. M. Gulick, G. Fatkenheuer, S. J. Schlesinger and M. C. Nussenzweig (2015). "Viraemia suppressed in HIV-1-infected humans by broadly neutralizing antibody 3BNC117." Nature **522**(7557): 487-491.

Caskey, M., T. Schoofs, H. Gruell, A. Settler, T. Karagounis, E. F. Kreider, B. Murrell, N. Pfeifer, L. Nogueira, T. Y. Oliveira, G. H. Learn, Y. Z. Cohen, C. Lehmann, D. Gillor, I. Shimeliovich, C. Unson-O'Brien, D. Weiland, A. Robles, T. Kummerle, C. Wyen, R. Levin, M. Witmer-Pack, K. Eren, C. Ignacio, S. Kiss, A. P. West, Jr., H. Mouquet, B. S. Zingman, R. M. Gulick, T. Keler, P. J. Bjorkman, M. S. Seaman, B. H. Hahn, G. Fatkenheuer, S. J. Schlesinger, M. C. Nussenzweig and F. Klein (2017). "Antibody 10-1074 suppresses viremia in HIV-1-infected individuals." Nat Med.

CDC (2014). "CDC. Vital Signs: HIV Diagnosis, Care, and Treatment Among Persons Living with HIV-United States 2011." MMWR **4**(63): 1-6.

Haynes, B. F. and M. J. McElrath (2013). "Progress in HIV-1 vaccine development." Curr Opin HIV AIDS **8**(4): 326-332.

Hessell, A. J., P. Poignard, M. Hunter, L. Hangartner, D. M. Tehrani, W. K. Bleeker, P. W. Parren, P. A. Marx and D. R. Burton (2009). "Effective, low-titer antibody protection against low-dose repeated mucosal SHIV challenge in macaques." Nat Med **15**(8): 951-954.

Hessell, A. J., E. G. Rakasz, P. Poignard, L. Hangartner, G. Landucci, D. N. Forthal, W. C. Koff, D. I. Watkins and D. R. Burton (2009). "Broadly neutralizing human anti-HIV antibody 2G12 is effective in protection against mucosal SHIV challenge even at low serum neutralizing titers." PLoS Pathog **5**(5): e1000433.

This copy of the document was retrieved from the system by Lisa Sunner on 24 Apr 2019

Jardine, J., J. P. Julien, S. Menis, T. Ota, O. Kalyuzhniy, A. McGuire, D. Sok, P. S. Huang, S. MacPherson, M. Jones, T. Nieusma, J. Mathison, D. Baker, A. B. Ward, D. R. Burton, L. Stamatatos, D. Nemazee, I. A. Wilson and W. R. Schief (2013). "Rational HIV immunogen design to target specific germline B cell receptors." Science **340**(6133): 711-716.

Julg, B., L. J. Tartaglia, B. F. Keele, K. Wagh, A. Pegu, D. Sok, P. Abbink, S. D. Schmidt, K. Wang, X. Chen, M. G. Joyce, I. S. Georgiev, M. Choe, P. D. Kwong, N. A. Doria-Rose, K. Le, M. K. Louder, R. T. Bailer, P. L. Moore, B. Korber, M. S. Seaman, S. S. Abdool Karim, L. Morris, R. A. Koup, J. R. Mascola, D. R. Burton and D. H. Barouch (2017). "Broadly neutralizing antibodies targeting the HIV-1 envelope V2 apex confer protection against a clade C SHIV challenge." Sci Transl Med **9**(406).

Ledgerwood, J. E., E. E. Coates, G. Yamshchikov, J. G. Saunders, L. Holman, M. E. Enama, A. DeZure, R. M. Lynch, I. Gordon, S. Plummer, C. S. Hendel, A. Pegu, M. Conan-Cibotti, S. Sitar, R. T. Bailer, S. Narpala, A. McDermott, M. Louder, S. O'Dell, S. Mohan, J. P. Pandey, R. M. Schwartz, Z. Hu, R. A. Koup, E. Capparelli, J. R. Mascola, B. S. Graham and V. R. C. S. Team (2015). "Safety, pharmacokinetics and neutralization of the broadly neutralizing HIV-1 human monoclonal antibody VRC01 in healthy adults." Clin Exp Immunol.

Lynch, R. M., E. Boritz, E. E. Coates, A. DeZure, P. Madden, P. Costner, M. E. Enama, S. Plummer, L. Holman, C. S. Hendel, I. Gordon, J. Casazza, M. Conan-Cibotti, S. A. Migueles, R. Tressler, R. T. Bailer, A. McDermott, S. Narpala, S. O'Dell, G. Wolf, J. D. Lifson, B. A. Freemire, R. J. Gorelick, J. P. Pandey, S. Mohan, N. Chomont, R. Fromentin, T. W. Chun, A. S. Fauci, R. M. Schwartz, R. A. Koup, D. C. Douek, Z. Hu, E. Capparelli, B. S. Graham, J. R. Mascola, J. E. Ledgerwood and V. R. C. S. Team (2015). "Virologic effects of broadly neutralizing antibody VRC01 administration during chronic HIV-1 infection." Sci Transl Med **7**(319): 319ra206.

Moldt, B., E. G. Rakasz, N. Schultz, P. Y. Chan-Hui, K. Swiderek, K. L. Weisgrau, S. M. Piaskowski, Z. Bergman, D. I. Watkins, P. Poignard and D. R. Burton (2012). "Highly potent HIV-specific antibody neutralization in vitro translates into effective protection against mucosal SHIV challenge in vivo." Proc Natl Acad Sci U S A **109**(46): 18921-18925.

Rudicell, R. S., Y. D. Kwon, S. Y. Ko, A. Pegu, M. K. Louder, I. S. Georgiev, X. Wu, J. Zhu, J. C. Boyington, X. Chen, W. Shi, Z. Y. Yang, N. A. Doria-Rose, K. McKee, S. O'Dell, S. D. Schmidt, G. Y. Chuang, A. Druz, C. Soto, Y. Yang, B. Zhang, T. Zhou, J. P. Todd, K. E. Lloyd, J. Eudailey, K. E. Roberts, B. R. Donald, R. T. Bailer, J. Ledgerwood, N. C. S. Program, J. C. Mullikin, L. Shapiro, R. A. Koup, B. S. Graham, M. C. Nason, M. Connors, B. F. Haynes, S. S. Rao, M. Roederer, P. D. Kwong, J. R. Mascola and G. J. Nabel (2014). "Enhanced potency of a broadly neutralizing HIV-1 antibody in vitro improves protection against lentiviral infection in vivo." J Virol **88**(21): 12669-12682.

Scheid, J. F., J. A. Horwitz, Y. Bar-On, E. F. Kreider, C. L. Lu, J. C. Lorenzi, A. Feldmann, M. Braunschweig, L. Nogueira, T. Oliveira, I. Shimeliovich, R. Patel, L. Burke, Y. Z. Cohen, S. Hadrigan, A. Settler, M. Witmer-Pack, A. P. West, Jr., B. Juelg, T. Keler, T. Hawthorne, B. Zingman, R. M. Gulick, N. Pfeifer, G. H. Learn, M. S. Seaman, P. J. Bjorkman, F. Klein, S. J. Schlesinger, B. D. Walker, B. H. Hahn and M. C. Nussenzweig (2016). "HIV-1 antibody 3BNC117 suppresses viral rebound in humans during treatment interruption." Nature **535**(7613): 556-560.

Scheid, J. F., H. Mouquet, B. Ueberheide, R. Diskin, F. Klein, T. Y. Oliveira, J. Pietzsch, D. Fenyo, A. Abadir, K. Velinzon, A. Hurley, S. Myung, F. Boulad, P. Poignard, D. R. Burton, F. Pereyra, D. D. Ho, B. D. Walker, M. S. Seaman, P. J. Bjorkman, B. T. Chait and M. C. Nussenzweig (2011). "Sequence and structural convergence of broad and potent HIV antibodies that mimic CD4 binding." Science **333**(6049): 1633-1637.

This copy of the document was retrieved from the system by Lisa Sunner on 24 Apr 2019

Schoofs, T., F. Klein, M. Braunschweig, E. F. Kreider, A. Feldmann, L. Nogueira, T. Oliveira, J. C. Lorenzi, E. H. Parrish, G. H. Learn, A. P. West, Jr., P. J. Bjorkman, S. J. Schlesinger, M. S. Seaman, J. Czartoski, M. J. McElrath, N. Pfeifer, B. H. Hahn, M. Caskey and M. C. Nussenzweig (2016). "HIV-1 therapy with monoclonal antibody 3BNC117 elicits host immune responses against HIV-1." Science **352**(6288): 997-1001.

Simek, M. D., W. Rida, F. H. Priddy, P. Pung, E. Carrow, D. S. Laufer, J. K. Lehrman, M. Boaz, T. Tarragona-Fiol, G. Miiro, J. Birungi, A. Pozniak, D. A. McPhee, O. Manigart, E. Karita, A. Inwoley, W. Jaoko, J. Dehovitz, L. G. Bekker, P. Pitisuttithum, R. Paris, L. M. Walker, P. Poignard, T. Wrin, P. E. Fast, D. R. Burton and W. C. Koff (2009). "Human immunodeficiency virus type 1 elite neutralizers: individuals with broad and potent neutralizing activity identified by using a high-throughput neutralization assay together with an analytical selection algorithm." J Virol **83**(14): 7337-7348.

Sok, D., K. J. Doores, B. Briney, K. M. Le, K. L. Saye-Francisco, A. Ramos, D. W. Kulp, J. P. Julien, S. Menis, L. Wickramasinghe, M. S. Seaman, W. R. Schief, I. A. Wilson, P. Poignard and D. R. Burton (2014). "Promiscuous glycan site recognition by antibodies to the high-mannose patch of gp120 broadens neutralization of HIV." Sci Transl Med **6**(236): 236ra263.

Sok, D., M. J. van Gils, M. Pauthner, J. P. Julien, K. L. Saye-Francisco, J. Hsueh, B. Briney, J. H. Lee, K. M. Le, P. S. Lee, Y. Hua, M. S. Seaman, J. P. Moore, A. B. Ward, I. A. Wilson, R. W. Sanders and D. R. Burton (2014). "Recombinant HIV envelope trimer selects for quaternary-dependent antibodies targeting the trimer apex." Proc Natl Acad Sci U S A **111**(49): 17624-17629.

UNAIDS (2016). "UNAIDS Fact Sheet November 2016."

Walker LM, B. D. (2010). "Rational antibody-based HIV-1 vaccine design: current approaches and future directions. ." Curr Opin Immunol. **22**(3): 358-366.

Walker, L. M., M. Huber, K. J. Doores, E. Falkowska, R. Pejchal, J. P. Julien, S. K. Wang, A. Ramos, P. Y. Chan-Hui, M. Moyle, J. L. Mitcham, P. W. Hammond, O. A. Olsen, P. Phung, S. Fling, C. H. Wong, S. Phogat, T. Wrin, M. D. Simek, G. P. I. Protocol, W. C. Koff, I. A. Wilson, D. R. Burton and P. Poignard (2011). "Broad neutralization coverage of HIV by multiple highly potent antibodies." Nature **477**(7365): 466-470.

Walker, L. M., S. K. Phogat, P. Y. Chan-Hui, D. Wagner, P. Phung, J. L. Goss, T. Wrin, M. D. Simek, S. Fling, J. L. Mitcham, J. K. Lehrman, F. H. Priddy, O. A. Olsen, S. M. Frey, P. W. Hammond, G. P. I. Protocol, S. Kaminsky, T. Zamb, M. Moyle, W. C. Koff, P. Poignard and D. R. Burton (2009). "Broad and potent neutralizing antibodies from an African donor reveal a new HIV-1 vaccine target." Science **326**(5950): 285-289.

Zhou, T., I. Georgiev, X. Wu, Z. Y. Yang, K. Dai, A. Finzi, Y. D. Kwon, J. F. Scheid, W. Shi, L. Xu, Y. Yang, J. Zhu, M. C. Nussenzweig, J. Sodroski, L. Shapiro, G. J. Nabel, J. R. Mascola and P. D. Kwong (2010). "Structural basis for broad and potent neutralization of HIV-1 by antibody VRC01." Science **329**(5993): 811-817.

This copy of the document was retrieved from the system by Lisa Sunner on 24 Apr 2019

APPENDIX F CTCAE TABLE

CTCAE4.03 Relevant For T002
Common Terminology Criteria for Adverse Events (CTCAE)
Version 4.0 Published: May 28, 2009 (v4.03: June 14, 2010)

U.S. DEPARTMENT OF HEALTH AND HUMAN SERVICES National Institutes of Health National Cancer Institute

Quick Reference

The NCI Common Terminology Criteria for Adverse Events is a descriptive terminology which can be utilized for Adverse Event (AE) reporting. A grading (severity) scale is provided for each AE term.

Components and Organization

SOC

System Organ Class, the highest level of the MedDRA hierarchy, is identified by anatomical or physiological system, etiology, or purpose (e.g., SOC Investigations for laboratory test results). CTCAE terms are grouped by MedDRA Primary SOCs. Within each SOC, AEs are listed and accompanied by descriptions of severity (Grade).

CTCAE Terms

An Adverse Event (AE) is any unfavorable and unintended sign (including an abnormal laboratory finding), symptom, or disease temporally associated with the use of a medical treatment or procedure that may or may not be considered related to the medical treatment or procedure. An AE is a term that is a unique representation of a specific event used for medical documentation and scientific analyses. Each CTCAE v4.0 term is a MedDRA LLT (Lowest Level Term).

Definitions

A brief definition is provided to clarify the meaning of each AE term.

TMF-03635 | 4.0 | Status:Approved | 16 Apr 2019
This copy of the document was retrieved from the system by Lisa Sunner on 24 Apr 2019

Grades Grade refers to the severity of the AE. The CTCAE displays Grades 1 through 5 with unique clinical descriptions of severity for each AE based on this general guideline:

Grade 1	Mild; asymptomatic or mild symptoms; clinical or diagnostic observations only; intervention not indicated.
Grade 2	Moderate; minimal, local or noninvasive intervention indicated; limiting age-appropriate instrumental ADL*.
Grade 3	Severe or medically significant but not immediately life-threatening; hospitalization or prolongation of hospitalization indicated; disabling; limiting self care ADL**.
Grade 4	Life-threatening consequences; urgent intervention indicated.
Grade 5	Death related to AE.

A Semi-colon indicates 'or' within the description of the grade.

A single dash (-) indicates a grade is not available.

Not all Grades are appropriate for all AEs. Therefore, some AEs are listed with fewer than five options for Grade selection.

Grade 5

Grade 5 (Death) is not appropriate for some AEs and therefore is not an option.

Activities of Daily Living (ADL)

*Instrumental ADL refer to preparing meals, shopping for groceries or clothes, using the telephone, managing money, etc.

**Self care ADL refer to bathing, dressing and undressing, feeding self, using the toilet, taking medications, and not bedridden.

† CTCAE v4.0 incorporates certain elements of the MedDRA terminology. For further details on MedDRA refer to the MedDRA MSSO Web site (http://www.meddramsso.com).

This copy of the document was retrieved from the system by Lisa Sunner on 24 Apr 2019

MedDRA v12.0 Code	CTCAE v4.0 SOC	CTCAE v4.0 Term	Grade 1	Grade 2	Grade 3	Grade 4	Grade 5	CTCAE v4.0 AE Term Definition
10001718	Immune system disorders	Allergic reaction	Transient flushing or rash, drug fever <38 degrees C (<100.4 degrees F); intervention not indicated	Intervention or infusion interruption indicated; responds promptly to symptomatic treatment (e.g., antihistamines, NSAIDS, narcotics); prophylactic medications indicated for <=24 hrs	Prolonged (e.g., not rapidly responsive to symptomatic medication and/or brief interruption of infusion); recurrence of symptoms following initial improvement; hospitalization indicated for clinical sequelae (e.g., renal impairment, pulmonary infiltrates)	Life-threatening consequences; urgent intervention indicated	Death	A disorder characterized by an adverse local or general response from exposure to an allergen.
10002218	Immune system disorders	Anaphylaxis	-	-	Symptomatic bronchospasm, with or without urticaria; parenteral intervention indicated; allergy-related edema/angioedema; hypotension	Life-threatening consequences; urgent intervention indicated	Death	A disorder characterized by an acute inflammatory reaction resulting from the release of histamine and histamine-like substances from mast cells, causing a hypersensitivity immune response. Clinically, it presents with breathing difficulty, dizziness, hypotension, cyanosis and loss of consciousness and may lead to death.

This copy of the document was retrieved from the system by Lisa Sunner on 24 Apr 2019

CONFIDENTIAL

MedDRA v12.0 Code	CTCAE v4.0 SOC	CTCAE v4.0 Term	Grade 1	Grade 2	Grade 3	Grade 4	Grade 5	CTCAE v4.0 AE Term Definition
10003239	Musculoskeletal and connective tissue disorders	Arthralgia	Mild pain	Moderate pain; limiting instrumental ADL	Severe pain; limiting self care ADL	-	-	A disorder characterized by a sensation of marked discomfort in a joint.
10008531	General disorders and administration site conditions	Chills	Mild sensation of cold; shivering; chattering of teeth	Moderate tremor of the entire body; narcotics indicated	Severe or prolonged, not responsive to narcotics	-	-	A disorder characterized by a sensation of cold that often marks a physiologic response to sweating after a fever.
10052015	Immune system disorders	Cytokine release syndrome	Mild reaction; infusion interruption not indicated; intervention not indicated	Therapy or infusion interruption indicated but responds promptly to symptomatic treatment (e.g., antihistamines, NSAIDS, narcotics, IV fluids); prophylactic medications indicated for <=24 hrs	Prolonged (e.g., not rapidly responsive to symptomatic medication and/or brief interruption of infusion); recurrence of symptoms following initial improvement; hospitalization indicated for clinical sequelae (e.g., renal impairment, pulmonary infiltrates)	Life-threatening consequences; pressor or ventilatory support indicated	Death	A disorder characterized by nausea, headache, tachycardia, hypotension, rash, and shortness of breath; it is caused by the release of cytokines from the cells.
10013573	Nervous system disorders	Dizziness	Mild unsteadiness or sensation of movement	Moderate unsteadiness or sensation of movement; limiting instrumental ADL	Severe unsteadiness or sensation of movement; limiting self care ADL	-	-	A disorder characterized by a disturbing sensation of lightheadedness, unsteadiness, giddiness, spinning or rocking.
10013963	Respiratory, thoracic and mediastinal disorders	Dyspnea	Shortness of breath with moderate exertion	Shortness of breath with minimal exertion; limiting instrumental ADL	Shortness of breath at rest; limiting self care ADL	Life-threatening consequences; urgent intervention indicated	Death	A disorder characterized by an uncomfortable sensation of difficulty breathing.

TMF-03635 | 4.0 | Status:Approved | 16 Apr 2019
This copy of the document was retrieved from the system by Lisa Sunner on 24 Apr 2019

MedDRA v12.0 Code	CTCAE v4.0 SOC	CTCAE v4.0 Term	Grade 1	Grade 2	Grade 3	Grade 4	Grade 5	CTCAE v4.0 AE Term Definition
10015218	Skin and subcutaneous tissue disorders	Erythema multiforme	Target lesions covering <10% BSA and not associated with skin tenderness	Target lesions covering 10 - 30% BSA and associated with skin tenderness	Target lesions covering >30% BSA and associated with oral or genital erosions	Target lesions covering >30% BSA; associated with fluid or electrolyte abnormalities; ICU care or burn unit indicated	Death	A disorder characterized by target lesions (a pink-red ring around a pale center).
10016558	General disorders and administration site conditions	Fever	38.0 - 39.0 degrees C (100.4 - 102.2 degrees F)	>39.0 - 40.0 degrees C (102.3 - 104.0 degrees F)	>40.0 degrees C (>104.0 degrees F) for <=24 hrs	>40.0 degrees C (>104.0 degrees F) for >24 hrs	Death	A disorder characterized by elevation of the body's temperature above the upper limit of normal.
10016825	Vascular disorders	Flushing	Asymptomatic; clinical or diagnostic observations only; intervention not indicated	Moderate symptoms; medical intervention indicated; limiting instrumental ADL	Symptomatic, associated with hypotension and/or tachycardia; limiting self care ADL	-	-	A disorder characterized by episodic reddening of the face.
10019211	Nervous system disorders	Headache	Mild pain	Moderate pain; limiting instrumental ADL	Severe pain; limiting self care ADL	-	-	A disorder characterized by a sensation of marked discomfort in various parts of the head, not confined to the area of distribution of any nerve.

This copy of the document was retrieved from the system by Lisa Sunner on 24 Apr 2019

MedDRA v12.0 Code	CTCAE v4.0 SOC	CTCAE v4.0 Term	Grade 1	Grade 2	Grade 3	Grade 4	Grade 5	CTCAE v4.0 AE Term Definition
10020772	Vascular disorders	Hypertension	Prehypertension (systolic BP 120 - 139 mm Hg or diastolic BP 80 - 89 mm Hg)	Stage 1 hypertension (systolic BP 140 - 159 mm Hg or diastolic BP 90 - 99 mm Hg); medical intervention indicated; recurrent or persistent (>=24 hrs); symptomatic increase by >20 mm Hg (diastolic) or to >140/90 mm Hg if previously WNL; monotherapy indicated	Stage 2 hypertension (systolic BP >=160 mm Hg or diastolic BP >=100 mm Hg); medical intervention indicated; more than one drug or more intensive therapy than previously used indicated	Life-threatening consequences (e.g., malignant hypertension, transient or permanent neurologic deficit, hypertensive crisis); urgent intervention indicated	Death	A disorder characterized by a pathological increase in blood pressure; a repeatedly elevation in the blood pressure exceeding 140 over 90 mm Hg.
10021097	Vascular disorders	Hypotension	Asymptomatic, intervention not indicated	Non-urgent medical intervention indicated	Medical intervention or hospitalization indicated	Life-threatening and urgent intervention indicated	Death	A disorder characterized by a blood pressure that is below the normal expected for an individual in a given environment.
10051792	General disorders and administration site conditions	Infusion related reaction	Mild transient reaction; infusion interruption not indicated; intervention not indicated	Therapy or infusion interruption indicated but responds promptly to symptomatic treatment (e.g., antihistamines, NSAIDS, narcotics, IV fluids); prophylactic medications indicated for <=24 hrs	Prolonged (e.g., not rapidly responsive to symptomatic medication and/or brief interruption of infusion); recurrence of symptoms following initial improvement; hospitalization indicated for clinical sequelae	Life-threatening consequences; urgent intervention indicated	Death	A disorder characterized by adverse reaction to the infusion of pharmacological or biological substances.

This copy of the document was retrieved from the system by Lisa Sunner on 24 Apr 2019

MedDRA v12.0 Code	CTCAE v4.0 SOC	CTCAE v4.0 Term	Grade 1	Grade 2	Grade 3	Grade 4	Grade 5	CTCAE v4.0 AE Term Definition
10064774	General disorders and administration site conditions	Infusion site extravasation	-	Erythema with associated symptoms (e.g., edema, pain, induration, phlebitis)	Ulceration or necrosis; severe tissue damage; operative intervention indicated	Life-threatening consequences; urgent intervention indicated	Death	A disorder characterized by leakage of a pharmacologic or a biologic substance from the infusion site into the surrounding tissue. Signs and symptoms include induration, erythema, swelling, burning sensation and marked discomfort at the infusion site.
10022095	General disorders and administration site conditions	Injection site reaction	Tenderness with or without associated symptoms (e.g., warmth, erythema, itching)	Pain; lipodystrophy; edema; phlebitis	Ulceration or necrosis; severe tissue damage; operative intervention indicated	Life-threatening consequences; urgent intervention indicated	Death	A disorder characterized by an intense adverse reaction (usually immunologic) developing at the site of an injection.
10025482	General disorders and administration site conditions	Malaise	Uneasiness or lack of well being	Uneasiness or lack of well being; limiting instrumental ADL	-	-	-	A disorder characterized by a feeling of general discomfort or uneasiness, an out-of-sorts feeling.
10028411	Musculoskeletal and connective tissue disorders	Myalgia	Mild pain	Moderate pain; limiting instrumental ADL	Severe pain; limiting self care ADL	-	-	A disorder characterized by marked discomfort sensation originating from a muscle or group of muscles.

This copy of the document was retrieved from the system by Lisa Sunner on 24 Apr 2019

MedDRA v12.0 Code	CTCAE v4.0 SOC	CTCAE v4.0 Term	Grade 1	Grade 2	Grade 3	Grade 4	Grade 5	CTCAE v4.0 AE Term Definition
10028813	Gastrointestinal disorders	Nausea	Loss of appetite without alteration in eating habits	Oral intake decreased without significant weight loss, dehydration or malnutrition	Inadequate oral caloric or fluid intake; tube feeding, TPN, or hospitalization indicated	-	-	A disorder characterized by a queasy sensation and/or the urge to vomit.
10033371	General disorders and administration site conditions	Pain	Mild pain	Moderate pain; limiting instrumental ADL	Severe pain; limiting self care ADL	-	-	A disorder characterized by the sensation of marked discomfort, distress or agony.
10033557	Cardiac disorders	Palpitations	Mild symptoms; intervention not indicated	Intervention indicated	-	-	-	A disorder characterized by an unpleasant sensation of irregular and/or forceful beating of the heart.
10037087	Skin and subcutaneous tissue disorders	Pruritus	Mild or localized; topical intervention indicated	Intense or widespread; intermittent; skin changes from scratching (e.g., edema, papulation, excoriations, lichenification, oozing/crusts); oral intervention indicated; limiting instrumental ADL	Intense or widespread; constant; limiting self care ADL or sleep; oral corticosteroid or immunosuppressive therapy indicated	-	-	A disorder characterized by an intense itching sensation.

This copy of the document was retrieved from the system by Lisa Sunner on 24 Apr 2019

MedDRA v12.0 Code	CTCAE v4.0 SOC	CTCAE v4.0 Term	Grade 1	Grade 2	Grade 3	Grade 4	Grade 5	CTCAE v4.0 AE Term Definition
10037868	Skin and subcutaneous tissue disorders	Rash maculo-papular	Macules/papules covering <10% BSA with or without symptoms (e.g., pruritus, burning, tightness)	Macules/papules covering 10 - 30% BSA with or without symptoms (e.g., pruritus, burning, tightness); limiting instrumental ADL	Macules/papules covering >30% BSA with or without associated symptoms; limiting self care ADL	-	-	A disorder characterized by the presence of macules (flat) and papules (elevated). Also known as morbillform rash, it is one of the most common cutaneous adverse events, frequently affecting the upper trunk, spreading centripetally and associated with pruritis.

This copy of the document was retrieved from the system by Lisa Sunner on 24 Apr 2019

MedDRA v12.0 Code	CTCAE v4.0 SOC	CTCAE v4.0 Term	Grade 1	Grade 2	Grade 3	Grade 4	Grade 5	CTCAE v4.0 AE Term Definition
10040400	Immune system disorders	Serum sickness	Asymptomatic; clinical or diagnostic observations only; intervention not indicated	Moderate arthralgia; fever, rash, urticaria, antihistamines indicated	Severe arthralgia or arthritis; extensive rash; steroids or IV fluids indicated	Life-threatening consequences; pressor or ventilatory support indicated	Death	A disorder characterized by a delayed-type hypersensitivity reaction to foreign proteins derived from an animal serum. It occurs approximately six to twenty-one days following the administration of the foreign antigen. Symptoms include fever, arthralgias, myalgias, skin eruptions, lymphadenopathy, chest marked discomfort and dyspnea.
10051837	Skin and subcutaneous tissue disorders	Skin induration	Mild induration, able to move skin parallel to plane (sliding) and perpendicular to skin (pinching up)	Moderate induration, able to slide skin, unable to pinch skin; limiting instrumental ADL	Severe induration, unable to slide or pinch skin; limiting joint movement or orifice (e.g., mouth, anus); limiting self care ADL	Generalized; associated with signs or symptoms of impaired breathing or feeding	Death	A disorder characterized by an area of hardness in the skin.
10042241	Respiratory, thoracic and mediastinal disorders	Stridor	-	-	Respiratory distress limiting self care ADL; medical intervention indicated	Life-threatening airway compromise; urgent intervention indicated (e.g., tracheotomy or intubation)	Death	A disorder characterized by a high pitched breathing sound due to laryngeal or upper airway obstruction.

This copy of the document was retrieved from the system by Lisa Sunner on 24 Apr 2019

MedDRA v12.0 Code	CTCAE v4.0 SOC	CTCAE v4.0 Term	Grade 1	Grade 2	Grade 3	Grade 4	Grade 5	CTCAE v4.0 AE Term Definition
10046735	Skin and subcutaneous tissue disorders	Urticaria	Urticarial lesions covering <10% BSA; topical intervention indicated	Urticarial lesions covering 10 - 30% BSA; oral intervention indicated	Urticarial lesions covering >30% BSA; IV intervention indicated	-	-	A disorder characterized by an itchy skin eruption characterized by wheals with pale interiors and well-defined red margins.
10047700	Gastrointestinal disorders	Vomiting	1 - 2 episodes (separated by 5 minutes) in 24 hrs	3 - 5 episodes (separated by 5 minutes) in 24 hrs	>=6 episodes (separated by 5 minutes) in 24 hrs; tube feeding, TPN or hospitalization indicated	Life-threatening consequences; urgent intervention indicated	Death	A disorder characterized by the reflexive act of ejecting the contents of the stomach through the mouth.

This copy of the document was retrieved from the system by Lisa Sunner on 24 Apr 2019

APPENDIX G: DAIDS ADVERSE EVENT SEVERITY ASSESSMENT TABLE

Division of AIDS (DAIDS) Table for Grading the Severity of Adult and Pediatric Adverse Events

Version 2.0
November 2014

Division of AIDS
National Institute of Allergy and Infectious Diseases
National Institutes of Health
US Department of Health and Human Services

This copy of the document was retrieved from the system by Lisa Sunner on 24 Apr 2019

TABLE OF CONTENTS

Glossary and Acronyms 1
Introduction 3
Instructions for Use 4
Major Clinical Conditions 7
- Cardiovascular 7
- Dermatologic 9
- Endocrine and Metabolic 10
- Gastrointestinal 11
- Musculoskeletal 13
- Neurologic 14
- Pregnancy, Puerperium, and Perinatal 16
- Psychiatric 17
- Respiratory 18
- Sensory 19
- Systemic 20
- Urinary 22

Site Reactions to Injections and Infusions 23
Laboratory Values 24
- Chemistries 24
- Hematology 27
- Urinalysis 29

Appendix A. Total Bilirubin Table for Term and Preterm Neonates 30

This copy of the document was retrieved from the system by Lisa Sunner on 24 Apr 2019

GLOSSARY AND ACRONYMS

AE	Adverse event; Any unfavorable and unintended sign (including an abnormal laboratory finding), symptom, or disease temporally associated with the use of a medical treatment or procedure regardless of whether it is considered related to the medical treatment or procedure.
ALT (SGPT)	Alanine aminotransferase (serum glutamic pyruvic transaminase)
ANC	Absolute neutrophil count
AST (SGOT)	Aspartate aminotransferase (serum glutamic-oxaloacetic transaminase)
AV	Atrioventricular
Basic Self-care Functions	Adult<br>Activities such as bathing, dressing, toileting, transfer or movement, continence, and feeding.<br><br>Young Children<br>Activities that are age and culturally appropriate, such as feeding one's self with culturally appropriate eating implements.
BMI z-score	Body mass index z- score; A body reference norm. Specifically, the number of standard deviations a participant's BMI differs from the average BMI for their age, sex, and ethnicity.
BMD t-score	Bone mineral density t-score; The number of standard deviations above or below the mean bone mineral density of a healthy 30 year old adult of the same sex and ethnicity as the participant.
BMD z-score	Bone mineral density z-score; The number of standard deviations a participant's BMD differs from the average BMD for their age, sex, and ethnicity.
BPAP	Bilevel positive airway pressure; A mode used during noninvasive positive pressure ventilation.
Chemical Pregnancy	A pregnancy in which a positive pregnancy test is followed by a negative pregnancy test without evidence of a clinical pregnancy loss.
CNS	Central nervous system
CPAP	Continuous positive airway pressure
DAERS	DAIDS Adverse Experience Reporting System; An internet-based system developed for clinical research sites to report Expedited Adverse Events (EAEs) to DAIDS. It facilitates timely EAE report submission and serves as a centralized location for accessing and processing EAE information for reporting purposes.
Disability	A substantial disruption of a person's ability to conduct normal life functions.
ECG	Electrocardiogram
eGFR	Estimated glomerular filtration rate
Hospitalization	Does not include the following hospital admissions: under 24 hours, unrelated to an adverse event (e.g., for labor and delivery, cosmetic surgery, social or administrative for temporary placement [for lack of a place to sleep]), protocol-specified, and for diagnosis or therapy of a condition that existed before the receipt of a study agent and which has not increased in severity or frequency.
INR	International normalized ratio

This copy of the document was retrieved from the system by Lisa Sunner on 24 Apr 2019

Glossary and Acronyms

Intervention	Medical, surgical, or other procedures recommended or provided by a healthcare professional for the treatment of an adverse event.
IV	Intravenous
IVIG	Intravenous immune globulin
LDL	Low density lipoprotein
LLN	Lower limit of normal
Life-threatening AE	Any adverse event that places the participant, in the view of the investigator, at immediate risk of death from the reaction when it occurred (i.e., it does not include a reaction that would have caused death if it had occurred in a more severe form).
NA	Not applicable
Participant ID	The identification number assigned to a study participant which is used to track study-related documentation, including any reported AEs.
PR Interval	The interval between the beginning of the P wave and the beginning of the QRS complex of an electrocardiogram that represents the time between the beginning of the contraction of the atria and the beginning of the contraction of the ventricles.
PT	Prothrombin time
PTT	Partial thromboplastin time
QTc Interval	The measure of time between the onset of ventricular depolarization and completion of ventricular repolarization corrected for ventricular rate.
RBC	Red blood cell
SI	Standard international unit
ULN	Upper limit of normal
Usual Social & Functional Activities	Activities which adults and children perform on a routine basis and those which are part of regular activities of daily living, for example:<br><br><u>Adults</u><br>Adaptive tasks and desirable activities, such as going to work, shopping, cooking, use of transportation, or pursuing a hobby.<br><br><u>Young Children</u><br>Activities that are age and culturally appropriate, such as social interactions, play activities, or learning tasks.
WBC	White blood cell
WHO	World Health Organization
WNL	Within normal limits

This copy of the document was retrieved from the system by Lisa Sunner on 24 Apr 2019

INTRODUCTION

The Division of AIDS (DAIDS) oversees clinical trials throughout the world which it sponsors and supports. The clinical trials evaluate the safety and efficacy of therapeutic products, vaccines, and other preventive modalities. Adverse event (AE) data collected during these clinical trials form the basis for subsequent safety and efficacy analyses of pharmaceutical products and medical devices. Incorrect and inconsistent AE severity grading can lead to inaccurate data analyses and interpretation, which in turn can impact the safety and well-being of clinical trial participants and future patients using pharmaceutical products.

The DAIDS AE grading table is a shared tool for assessing the severity of AEs (including clinical and laboratory abnormalities) in participants enrolled in clinical trials. Over the years as scientific knowledge and experience have expanded, revisions to the DAIDS AE grading table have become necessary.

The Division of AIDS (DAIDS) Table for Grading the Severity of Adult and Pediatric Adverse Events, Version 2.0 replaces the grading table published in 2004 and updated in 2009. In version 2.0, AEs not previously included, but which now are deemed medically important events, are included while other AEs have been removed. Some AE severity grading descriptions have been revised to more appropriately reflect the presentation of these events in clinical settings and their impact on clinical trials. For example, DAIDS performed an extensive literature search and reviews of select DAIDS clinical trial data in revising certain hematology parameters (i.e., hemoglobin, white cell counts, and absolute neutrophil counts). DAIDS also took into consideration the U.S. Food and Drug Administration's guidance regarding the use of local laboratory reference values and ethnic differences among certain healthy adolescent and adult populations in defining parameter limits. Finally, the revised DAIDS AE grading table also contains an updated glossary and acronyms section, an expanded instructions for use section, and an appendix that provides more age-specific information for an AE of concern to DAIDS.

DAIDS is grateful to the DAIDS Grading Table Working Group, numerous government and non-government affiliated medical subject matter experts and reviewers who were instrumental in the revision of the DAIDS AE grading table.

INSTRUCTIONS FOR USE

General Considerations

The *Division of AIDS (DAIDS) Table for Grading the Severity of Adult and Pediatric Adverse Events, Version 2.0* consists of parameters, or AEs, with severity grading guidance that are to be used in DAIDS clinical trials for safety data reporting to maintain accuracy and consistency in the evaluation of AEs. The term "severe" is not the same as the term "serious" in classifying AEs. The severity of a specific event describes its intensity, and it is the intensity which is graded. Seriousness, which is not graded, relates to an outcome of an AE and is a regulatory definition.

Clinical sites are encouraged to report parameters in the DAIDS AE grading table as they are written to maintain data consistency across clinical trials. However, since some parameters can be reported with more specificity, clinical sites are encouraged to report parameters that convey additional clinical information. For example, diarrhea could be reported as neonatal diarrhea; seizures, as febrile seizures; and pain, as jaw pain.

The DAIDS AE grading table provides an AE severity grading scale ranging from grades 1 to 5 with descriptions for each AE based on the following general guidelines:

- Grade 1 indicates a mild event

- Grade 2 indicates a moderate event
- Grade 3 indicates a severe event
- Grade 4 indicates a potentially life-threatening event
- Grade 5 indicates death (*Note*: This grade is not specifically listed on each page of the grading table).

Other points to consider include:
- Use parameters defined by age and sex values as applicable.
- Male and female sex are defined as sex at birth.
- Unless noted, laboratory values are for term neonates. Preterm neonates should be assessed using local laboratory normal ranges.
- Where applicable, Standard International (SI) units are included in italics.

Selecting and Reporting a Primary AE Term

When selecting a primary AE term to report, sites should select the term that best describes what occurred to the participant. For example, a participant may present with itching, urticaria, flushing, angioedema of the face, and dyspnea. If the underlying diagnosis is determined to be an acute allergic reaction, sites should report “Acute Allergic Reaction” as the primary AE term.

Primary AE terms should be reported using the DAIDS Adverse Experience Reporting System (DAERS) only if they meet expedited reporting criteria. However, all primary AE terms should be reported using protocol-specific case report forms (CRFs). Because the reported information is stored in different databases (i.e., safety and clinical), sites should report primary AE terms using the same terminology for data consistency.

When reporting using DAERS, other clinically significant events associated with a primary AE term that more fully describe the nature, severity, or complications of the primary AE term should be entered in the “Other Events” section. However, the severity grade for these events must be lower than or equal to the severity grade of the primary AE term. In the example above, dyspnea and angioedema of the face may be entered in the “Other Events” section, because they are more descriptive and provide additional information on the severity of the acute allergic reaction. However, their severity grades must be lower than or equal to the severity grade of the primary AE term of “Acute Allergic Reaction”.

Differences exist in the reporting and recording of information (e.g., signs and symptoms, clinically significant events) in DAERS and CRFs. Therefore, sites should refer to their protocols and CRF requirements for further instructions.

Grading Adult and Pediatric AEs

When a single parameter is not appropriate for grading an AE in both adult and pediatric populations, separate parameters with specified age ranges are provided. If no distinction between adult and pediatric populations has been made, the listed parameter should be used for grading an AE in both populations.

Reporting Pregnancy Outcomes

In the *Pregnancy, Puerperium, and Perinatal* section, all parameters are pregnancy outcomes and should be reported using the mother's participant ID. If an infant is not enrolled in the same study as

This copy of the document was retrieved from the system by Lisa Sunner on 24 Apr 2019

the mother, any identified birth defects should be reported using the mother's participant ID. However, if an infant is enrolled in the same study as the mother or in another study, any identified birth defects should be reported using the infant's participant ID. Sites should refer to the applicable network standards for reporting abnormal pregnancy outcomes on the CRFs.

Determining Severity Grade for Parameters between Grades
If the severity of an AE could fall in either one of two grades (i.e., the severity of an AE could be either grade 2 or grade 3), sites should select the higher of the two grades.

Laboratory Values
General. An asymptomatic, abnormal laboratory finding without an accompanying AE should not be reported to DAIDS in an expedited timeframe unless it meets protocol-specific reporting requirements. Sites should refer to the applicable network standards for reporting abnormal laboratory findings on the CRFs.

Values below Grade 1. Any laboratory value that is between the ULN and grade 1 (for high values) or the LLN and grade 1 (for low values) should not be graded or reported as an AE. Sites should consult the *Manual for Expedited Reporting of Adverse Events to DAIDS, Version 2.0* and their protocol when making an assessment of the need to report an AE.

Overlap of Local Laboratory Normal Values with Grading Table Ranges. When local laboratory normal values fall within grading table laboratory ranges, the severity grading is based on the ranges in the grading table unless there is a protocol-specific grading criterion for the laboratory value. For example, "Magnesium, Low" has a grade 1 range of 1.2 to < 1.4 mEq/L, while a particular laboratory's normal range for magnesium may be 1.3 to 2.8 mEq/L. If a study participant's magnesium laboratory value is 1.3 mEq/L, the laboratory value should be graded as grade 1.

Appendix Usage
Appendix A takes priority over the main grading table in all assessments of total bilirubin for term and preterm neonates.

Using Addenda 1-3: Grading Tables Used in Microbicide Studies
In protocols involving topical application of products to the female and male genital tracts or rectum, strong consideration should be given to using Addenda 1-3 (see below) as the primary

grading tables for these areas. Although these grading tables are used specifically in microbicide studies, they may be used in other protocols as adjuncts to the main grading table (i.e., the *Division of AIDS (AIDS) Table for Grading the Severity of Adult and Pediatric Adverse Events, Version 2.0*). It should be clearly stated in a protocol which addendum is being used as the primary grading table (and thus takes precedence over the main grading table) and which addendum is being used in a complementary fashion.

- Addendum 1 – Female Genital Grading Table for Use in Microbicide Studies – PDF
- Addendum 2 – Male Genital Grading Table for Use in Microbicide Studies – PDF
- Addendum 3 – Rectal Grading Table for Use in Microbicide Studies – PDF

This copy of the document was retrieved from the system by Lisa Sunner on 24 Apr 2019

Estimating Severity Grade for Parameters Not Identified in the Grading Table

The functional table below should be used to grade the severity of an AE that is not specifically identified in the grading table. In addition, all deaths related to an AE are to be classified as grade 5.

PARAMETER	GRADE 1 MILD	GRADE 2 MODERATE	GRADE 3 SEVERE	GRADE 4 POTENTIALLY LIFE-THREATENING
**Clinical** adverse event **NOT** identified elsewhere in the grading table	Mild symptoms causing no or minimal interference with usual social & functional activities with intervention not indicated	Moderate symptoms causing greater than minimal interference with usual social & functional activities with intervention indicated	Severe symptoms causing inability to perform usual social & functional activities with intervention or hospitalization indicated	Potentially life-threatening symptoms causing inability to perform basic self-care functions with intervention indicated to prevent permanent impairment, persistent disability, or death

This copy of the document was retrieved from the system by Lisa Sunner on 24 Apr 2019

CARDIOVASCULAR

PARAMETER	GRADE 1 MILD	GRADE 2 MODERATE	GRADE 3 SEVERE	GRADE 4 POTENTIALLY LIFE-THREATENING
**Arrhythmia** (by ECG or physical examination) *Specify type, if applicable*	No symptoms AND No intervention indicated	No symptoms AND Non-urgent intervention indicated	Non-life-threatening symptoms AND Non-urgent intervention indicated	Life-threatening arrhythmia OR Urgent intervention indicated
**Blood Pressure Abnormalities**[1] ***Hypertension*** *(with the lowest reading taken after repeat testing during a visit)* *≥ 18 years of age*	140 to < 160 mmHg systolic OR 90 to < 100 mmHg diastolic	≥ 160 to < 180 mmHg systolic OR ≥ 100 to < 110 mmHg diastolic	≥ 180 mmHg systolic OR ≥ 110 mmHg diastolic	Life-threatening consequences in a participant not previously diagnosed with hypertension (e.g., malignant hypertension) OR Hospitalization indicated
< 18 years of age	> 120/80 mmHg	≥ 95th to < 99th percentile + 5 mmHg adjusted for age, height, and gender (systolic and/or diastolic)	≥ 99th percentile + 5 mmHg adjusted for age, height, and gender (systolic and/or diastolic)	Life-threatening consequences in a participant not previously diagnosed with hypertension (e.g., malignant hypertension) OR Hospitalization indicated
Hypotension	No symptoms	Symptoms corrected with oral fluid replacement	Symptoms AND IV fluids indicated	Shock requiring use of vasopressors or mechanical assistance to maintain blood pressure
**Cardiac Ischemia or Infarction** *Report only one*	NA	NA	New symptoms with ischemia (stable angina) OR New testing consistent with ischemia	Unstable angina OR Acute myocardial infarction
**Heart Failure**	No symptoms AND Laboratory or cardiac imaging abnormalities	Symptoms with mild to moderate activity or exertion	Symptoms at rest or with minimal activity or exertion (e.g., hypoxemia) OR Intervention indicated (e.g., oxygen)	Life-threatening consequences OR Urgent intervention indicated (e.g., vasoactive medications, ventricular assist device, heart transplant)
**Hemorrhage** (with significant acute blood loss)	NA	Symptoms AND No transfusion indicated	Symptoms AND Transfusion of ≤ 2 units packed RBCs indicated	Life-threatening hypotension OR Transfusion of > 2 units packed RBCs (for children, packed RBCs > 10 cc/kg) indicated

[1] Blood pressure norms for children < 18 years of age can be found in: Expert Panel on Integrated Guidelines for Cardiovascular Health and Risk Reduction in Children and Adolescents. *Pediatrics* 2011;128;S213; originally published online November 14, 2011; DOI: 10.1542/peds.2009 2107C

This copy of the document was retrieved from the system by Lisa Sunner on 24 Apr 2019

PARAMETER	GRADE 1 MILD	GRADE 2 MODERATE	GRADE 3 SEVERE	GRADE 4 POTENTIALLY LIFE-THREATENING
**Prolonged PR Interval or AV Block**<br>*Report only one*<br>*> 16 years of age*	PR interval 0.21 to < 0.25 seconds	PR interval ≥ 0.25 seconds OR Type I 2nd degree AV block	Type II 2nd degree AV block OR Ventricular pause ≥ 3.0 seconds	Complete AV block
≤ 16 years of age	1st degree AV block (PR interval > normal for age and rate)	Type I 2nd degree AV block	Type II 2nd degree AV block OR Ventricular pause ≥ 3.0 seconds	Complete AV block
**Prolonged QTc Interval**[2]	0.45 to 0.47 seconds	> 0.47 to 0.50 seconds	> 0.50 seconds OR ≥ 0.06 seconds above baseline	Life-threatening consequences (e.g., Torsade de pointes, other associated serious ventricular dysrhythmia)
**Thrombosis or Embolism**<br>*Report only one*	NA	Symptoms AND No intervention indicated	Symptoms AND Intervention indicated	Life-threatening embolic event (e.g., pulmonary embolism, thrombus)

[2] As per Bazett's formu

This copy of the document was retrieved from the system by Lisa Sunner on 24 Apr 2019

DERMATOLOGIC

PARAMETER	GRADE 1 MILD	GRADE 2 MODERATE	GRADE 3 SEVERE	GRADE 4 POTENTIALLY LIFE-THREATENING
**Alopecia** (scalp only)	Detectable by study participant, caregiver, or physician AND Causing no or minimal interference with usual social & functional activities	Obvious on visual inspection AND Causing greater than minimal interference with usual social & functional activities	NA	NA
**Bruising**	Localized to one area	Localized to more than one area	Generalized	NA
**Cellulitis**	NA	Non-parenteral treatment indicated (e.g., oral antibiotics, antifungals, antivirals)	IV treatment indicated (e.g., IV antibiotics, antifungals, antivirals)	Life-threatening consequences (e.g., sepsis, tissue necrosis)
**Hyperpigmentation**	Slight or localized causing no or minimal interference with usual social & functional activities	Marked or generalized causing greater than minimal interference with usual social & functional activities	NA	NA
**Hypopigmentation**	Slight or localized causing no or minimal interference with usual social & functional activities	Marked or generalized causing greater than minimal interference with usual social & functional activities	NA	NA
**Petechiae**	Localized to one area	Localized to more than one area	Generalized	NA
**Pruritus**[3] (without skin lesions)	Itching causing no or minimal interference with usual social & functional activities	Itching causing greater than minimal interference with usual social & functional activities	Itching causing inability to perform usual social & functional activities	NA
**Rash** *Specify type, if applicable*	Localized rash	Diffuse rash OR Target lesions	Diffuse rash AND Vesicles or limited number of bullae or superficial ulcerations of mucous membrane limited to one site	Extensive or generalized bullous lesions OR Ulceration of mucous membrane involving two or more distinct mucosal sites OR Stevens-Johnson syndrome OR Toxic epidermal necrolysis

[3] For pruritus associated with injections or infusions, see the *Site Reactions to Injections and Infusions* section (page 23).

This copy of the document was retrieved from the system by Lisa Sunner on 24 Apr 2019

ENDOCRINE AND METABOLIC

PARAMETER	GRADE 1 MILD	GRADE 2 MODERATE	GRADE 3 SEVERE	GRADE 4 POTENTIALLY LIFE-THREATENING
**Diabetes Mellitus**	Controlled without medication	Controlled with medication OR Modification of current medication regimen	Uncontrolled despite treatment modification OR Hospitalization for immediate glucose control indicated	Life-threatening consequences (e.g., ketoacidosis, hyperosmolar non-ketotic coma, end organ failure)
**Gynecomastia**	Detectable by study participant, caregiver, or physician AND Causing no or minimal interference with usual social & functional activities	Obvious on visual inspection AND Causing pain with greater than minimal interference with usual social & functional activities	Disfiguring changes AND Symptoms requiring intervention or causing inability to perform usual social & functional activities	NA
**Hyperthyroidism**	No symptoms AND Abnormal laboratory value	Symptoms causing greater than minimal interference with usual social & functional activities OR Thyroid suppression therapy indicated	Symptoms causing inability to perform usual social & functional activities OR Uncontrolled despite treatment modification	Life-threatening consequences (e.g., thyroid storm)
**Hypothyroidism**	No symptoms AND Abnormal laboratory value	Symptoms causing greater than minimal interference with usual social & functional activities OR Thyroid replacement therapy indicated	Symptoms causing inability to perform usual social & functional activities OR Uncontrolled despite treatment modification	Life-threatening consequences (e.g., myxedema coma)
**Lipoatrophy**[4]	Detectable by study participant, caregiver, or physician AND Causing no or minimal interference with usual social & functional activities	Obvious on visual inspection AND Causing greater than minimal interference with usual social & functional activities	Disfiguring changes	NA
**Lipohypertrophy**[5]	Detectable by study participant, caregiver, or physician AND Causing no or minimal interference with usual social & functional activities	Obvious on visual inspection AND Causing greater than minimal interference with usual social & functional activities	Disfiguring changes	NA

[4] Definition: A disorder characterized by fat loss in the face, extremities, and buttocks.
[5] Definition: A disorder characterized by abnormal fat accumulation on the back of the neck, breasts, and abdomen.

This copy of the document was retrieved from the system by Lisa Sunner on 24 Apr 2019

GASTROINTESTINAL

PARAMETER	GRADE 1 MILD	GRADE 2 MODERATE	GRADE 3 SEVERE	GRADE 4 POTENTIALLY LIFE-THREATENING
**Anorexia**	Loss of appetite without decreased oral intake	Loss of appetite associated with decreased oral intake without significant weight loss	Loss of appetite associated with significant weight loss	Life-threatening consequences OR Aggressive intervention indicated (e.g., tube feeding, total parenteral nutrition)
**Ascites**	No symptoms	Symptoms AND Intervention indicated (e.g., diuretics, therapeutic paracentesis)	Symptoms recur or persist despite intervention	Life-threatening consequences
**Bloating or Distension** *Report only one*	Symptoms causing no or minimal interference with usual social & functional activities	Symptoms causing greater than minimal interference with usual social & functional activities	Symptoms causing inability to perform usual social & functional activities	NA
**Cholecystitis**	NA	Symptoms AND Medical intervention indicated	Radiologic, endoscopic, or operative intervention indicated	Life-threatening consequences (e.g., sepsis, perforation)
**Constipation**	NA	Persistent constipation requiring regular use of dietary modifications, laxatives, or enemas	Obstipation with manual evacuation indicated	Life-threatening consequences (e.g., obstruction)
**Diarrhea** *≥ 1 year of age*	Transient or intermittent episodes of unformed stools OR Increase of ≤ 3 stools over baseline per 24-hour period	Persistent episodes of unformed to watery stools OR Increase of 4 to 6 stools over baseline per 24-hour period	Increase of ≥ 7 stools per 24-hour period OR IV fluid replacement indicated	Life-threatening consequences (e.g., hypotensive shock)
< 1 year of age	Liquid stools (more unformed than usual) but usual number of stools	Liquid stools with increased number of stools OR Mild dehydration	Liquid stools with moderate dehydration	Life-threatening consequences (e.g., liquid stools resulting in severe dehydration, hypotensive shock)
**Dysphagia or Odynophagia** *Report only one and specify location*	Symptoms but able to eat usual diet	Symptoms causing altered dietary intake with no intervention indicated	Symptoms causing severely altered dietary intake with intervention indicated	Life-threatening reduction in oral intake
**Gastrointestinal Bleeding**	Not requiring intervention other than iron supplement	Endoscopic intervention indicated	Transfusion indicated	Life-threatening consequences (e.g., hypotensive shock)

This copy of the document was retrieved from the system by Lisa Sunner on 24 Apr 2019

GASTROINTESTINAL

PARAMETER	GRADE 1 MILD	GRADE 2 MODERATE	GRADE 3 SEVERE	GRADE 4 POTENTIALLY LIFE-THREATENING
**Mucositis or Stomatitis** *Report only one and specify location*	Mucosal erythema	Patchy pseudomembranes or ulcerations	Confluent pseudomembranes or ulcerations OR Mucosal bleeding with minor trauma	Life-threatening consequences (e.g., aspiration, choking) OR Tissue necrosis OR Diffuse spontaneous mucosal bleeding
**Nausea**	Transient (< 24 hours) or intermittent AND No or minimal interference with oral intake	Persistent nausea resulting in decreased oral intake for 24 to 48 hours	Persistent nausea resulting in minimal oral intake for > 48 hours OR Rehydration indicated (e.g., IV fluids)	Life-threatening consequences (e.g., hypotensive shock)
**Pancreatitis**	NA	Symptoms with hospitalization not indicated	Symptoms with hospitalization indicated	Life-threatening consequences (e.g., circulatory failure, hemorrhage, sepsis)
**Perforation** (colon or rectum)	NA	NA	Intervention indicated	Life-threatening consequences
**Proctitis**	Rectal discomfort with no intervention indicated	Symptoms causing greater than minimal interference with usual social & functional activities OR Medical intervention indicated	Symptoms causing inability to perform usual social & functional activities OR Operative intervention indicated	Life-threatening consequences (e.g., perforation)
**Rectal Discharge**	Visible discharge	Discharge requiring the use of pads	NA	NA
**Vomiting**	Transient or intermittent AND No or minimal interference with oral intake	Frequent episodes with no or mild dehydration	Persistent vomiting resulting in orthostatic hypotension OR Aggressive rehydration indicated (e.g., IV fluids)	Life-threatening consequences (e.g., hypotensive shock)

This copy of the document was retrieved from the system by Lisa Sunner on 24 Apr 2019

MUSCULOSKELETAL

PARAMETER	GRADE 1 MILD	GRADE 2 MODERATE	GRADE 3 SEVERE	GRADE 4 POTENTIALLY LIFE-THREATENING
**Arthralgia**	Joint pain causing no or minimal interference with usual social & functional activities	Joint pain causing greater than minimal interference with usual social & functional activities	Joint pain causing inability to perform usual social & functional activities	Disabling joint pain causing inability to perform basic self-care functions
**Arthritis**	Stiffness or joint swelling causing no or minimal interference with usual social & functional activities	Stiffness or joint swelling causing greater than minimal interference with usual social & functional activities	Stiffness or joint swelling causing inability to perform usual social & functional activities	Disabling joint stiffness or swelling causing inability to perform basic self-care functions
**Myalgia** (generalized)	Muscle pain causing no or minimal interference with usual social & functional activities	Muscle pain causing greater than minimal interference with usual social & functional activities	Muscle pain causing inability to perform usual social & functional activities	Disabling muscle pain causing inability to perform basic self-care functions
**Osteonecrosis**	NA	No symptoms but with radiographic findings AND No operative intervention indicated	Bone pain with radiographic findings OR Operative intervention indicated	Disabling bone pain with radiographic findings causing inability to perform basic self-care functions
**Osteopenia**[6] *≥ 30 years of age*	BMD t-score -2.5 to -1	NA	NA	NA
< 30 years of age	BMD z-score -2 to -1	NA	NA	NA
**Osteoporosis**[6] *≥ 30 years of age*	NA	BMD t-score < -2.5	Pathologic fracture (e.g., compression fracture causing loss of vertebral height)	Pathologic fracture causing life-threatening consequences
< 30 years of age	NA	BMD z-score < -2	Pathologic fracture (e.g., compression fracture causing loss of vertebral height)	Pathologic fracture causing life-threatening consequences

[6] BMD t and z scores can be found in: Kanis JA on behalf of the World Health Organization Scientific Group (2007). Assessment of osteoporosis at the primary health-care level. Technical Report. World Health Organization Collaborating Centre for Metabolic Bone Diseases, University of Sheffield, UK. 2007: Printed by the University of Sheffield

This copy of the document was retrieved from the system by Lisa Sunner on 24 Apr 2019

NEUROLOGIC

PARAMETER	GRADE 1 MILD	GRADE 2 MODERATE	GRADE 3 SEVERE	GRADE 4 POTENTIALLY LIFE-THREATENING
**Acute CNS Ischemia**	NA	NA	Transient ischemic attack	Cerebral vascular accident (e.g., stroke with neurological deficit)
**Altered Mental Status** (for Dementia, see *Cognitive, Behavioral, or Attentional Disturbance* below)	Changes causing no or minimal interference with usual social & functional activities	Mild lethargy or somnolence causing greater than minimal interference with usual social & functional activities	Confusion, memory impairment, lethargy, or somnolence causing inability to perform usual social & functional activities	Delirium OR Obtundation OR Coma
**Ataxia**	Symptoms causing no or minimal interference with usual social & functional activities OR No symptoms with ataxia detected on examination	Symptoms causing greater than minimal interference with usual social & functional activities	Symptoms causing inability to perform usual social & functional activities	Disabling symptoms causing inability to perform basic self-care functions
**Cognitive, Behavioral, or Attentional Disturbance** (includes dementia and attention deficit disorder) *Specify type, if applicable*	Disability causing no or minimal interference with usual social & functional activities OR Specialized resources not indicated	Disability causing greater than minimal interference with usual social & functional activities OR Specialized resources on part-time basis indicated	Disability causing inability to perform usual social & functional activities OR Specialized resources on a full-time basis indicated	Disability causing inability to perform basic self-care functions OR Institutionalization indicated
**Developmental Delay** *< 18 years of age* *Specify type, if applicable*	Mild developmental delay, either motor or cognitive, as determined by comparison with a developmental screening tool appropriate for the setting	Moderate developmental delay, either motor or cognitive, as determined by comparison with a developmental screening tool appropriate for the setting	Severe developmental delay, either motor or cognitive, as determined by comparison with a developmental screening tool appropriate for the setting	Developmental regression, either motor or cognitive, as determined by comparison with a developmental screening tool appropriate for the setting
**Headache**	Symptoms causing no or minimal interference with usual social & functional activities	Symptoms causing greater than minimal interference with usual social & functional activities	Symptoms causing inability to perform usual social & functional activities	Symptoms causing inability to perform basic self-care functions OR Hospitalization indicated OR Headache with significant impairment of alertness or other neurologic function

This copy of the document was retrieved from the system by Lisa Sunner on 24 Apr 2019

NEUROLOGIC

PARAMETER	GRADE 1 MILD	GRADE 2 MODERATE	GRADE 3 SEVERE	GRADE 4 POTENTIALLY LIFE-THREATENING
**Neuromuscular Weakness** (includes myopathy and neuropathy)<br>*Specify type, if applicable*	Minimal muscle weakness causing no or minimal interference with usual social & functional activities OR No symptoms with decreased strength on examination	Muscle weakness causing greater than minimal interference with usual social & functional activities	Muscle weakness causing inability to perform usual social & functional activities	Disabling muscle weakness causing inability to perform basic self-care functions OR Respiratory muscle weakness impairing ventilation
**Neurosensory Alteration** (includes paresthesia and painful neuropathy)<br>*Specify type, if applicable*	Minimal paresthesia causing no or minimal interference with usual social & functional activities OR No symptoms with sensory alteration on examination	Sensory alteration or paresthesia causing greater than minimal interference with usual social & functional activities	Sensory alteration or paresthesia causing inability to perform usual social & functional activities	Disabling sensory alteration or paresthesia causing inability to perform basic self-care functions
**Seizures**<br>***New Onset Seizure***<br>*≥ 18 years of age*	NA	NA	1 to 3 seizures	Prolonged and repetitive seizures (e.g., status epilepticus) OR Difficult to control (e.g., refractory epilepsy)
< 18 years of age (includes new or pre-existing febrile seizures)	Seizure lasting < 5 minutes with < 24 hours postictal state	Seizure lasting 5 to < 20 minutes with < 24 hours postictal state	Seizure lasting ≥ 20 minutes OR > 24 hours postictal state	Prolonged and repetitive seizures (e.g., status epilepticus) OR Difficult to control (e.g., refractory epilepsy)
Pre-existing Seizure	NA	Increased frequency from previous level of control without change in seizure character	Change in seizure character either in duration or quality (e.g., severity or focality)	Prolonged and repetitive seizures (e.g., status epilepticus) OR Difficult to control (e.g., refractory epilepsy)
**Syncope**	Near syncope without loss of consciousness (e.g., pre-syncope)	Loss of consciousness with no intervention indicated	Loss of consciousness AND Hospitalization or intervention required	NA

This copy of the document was retrieved from the system by Lisa Sunner on 24 Apr 2019

Pregnancy, Puerperium, and Perinatal

PARAMETER	GRADE 1 MILD	GRADE 2 MODERATE	GRADE 3 SEVERE	GRADE 4 POTENTIALLY LIFE-THREATENING
**Fetal Death or Stillbirth** (report using mother's participant ID)<br>*Report only one*	NA	NA	Fetal loss occurring at ≥ 20 weeks gestation	NA
**Preterm Delivery**[7] (report using mother's participant ID)	Delivery at 34 to < 37 weeks gestational age	Delivery at 28 to < 34 weeks gestational age	Delivery at 24 to < 28 weeks gestational age	Delivery at < 24 weeks gestational age
**Spontaneous Abortion or Miscarriage**[8] (report using mother's participant ID)<br>*Report only one*	Chemical pregnancy	Uncomplicated spontaneous abortion or miscarriage	Complicated spontaneous abortion or miscarriage	NA

[7] Definition: A delivery of a live-born neonate occurring at ≥ 20 to < 37 weeks gestational age.
[8] Definition: A clinically recognized pregnancy occurring at < 20 weeks gestational

Psychiatric

PARAMETER	GRADE 1 MILD	GRADE 2 MODERATE	GRADE 3 SEVERE	GRADE 4 POTENTIALLY LIFE-THREATENING
**Insomnia**	Mild difficulty falling asleep, staying asleep, or waking up early	Moderate difficulty falling asleep, staying asleep, or waking up early	Severe difficulty falling asleep, staying asleep, or waking up early	NA
**Psychiatric Disorders** (includes anxiety, depression, mania, and psychosis)<br>*Specify disorder*	Symptoms with intervention not indicated OR Behavior causing no or minimal interference with usual social & functional activities	Symptoms with intervention indicated OR Behavior causing greater than minimal interference with usual social & functional activities	Symptoms with hospitalization indicated OR Behavior causing inability to perform usual social & functional activities	Threatens harm to self or others OR Acute psychosis OR Behavior causing inability to perform basic self-care functions
**Suicidal Ideation or Attempt**<br>*Report only one*	Preoccupied with thoughts of death AND No wish to kill oneself	Preoccupied with thoughts of death AND Wish to kill oneself with no specific plan or intent	Thoughts of killing oneself with partial or complete plans but no attempt to do so OR Hospitalization indicated	Suicide attempted

This copy of the document was retrieved from the system by Lisa Sunner on 24 Apr 2019

RESPIRATORY

PARAMETER	GRADE 1 MILD	GRADE 2 MODERATE	GRADE 3 SEVERE	GRADE 4 POTENTIALLY LIFE-THREATENING
**Acute Bronchospasm**	Forced expiratory volume in 1 second or peak flow reduced to ≥ 70 to < 80% OR Mild symptoms with intervention not indicated	Forced expiratory volume in 1 second or peak flow 50 to < 70% OR Symptoms with intervention indicated OR Symptoms causing greater than minimal interference with usual social & functional activities	Forced expiratory volume in 1 second or peak flow 25 to < 50% OR Symptoms causing inability to perform usual social & functional activities	Forced expiratory volume in 1 second or peak flow < 25% OR Life-threatening respiratory or Hemodynamic compromise OR Intubation
**Dyspnea or Respiratory Distress** *Report only one*	Dyspnea on exertion with no or minimal interference with usual social & functional activities OR Wheezing OR Minimal increase in respiratory rate for age	Dyspnea on exertion causing greater than minimal interference with usual social & functional activities OR Nasal flaring OR Intercostal retractions OR Pulse oximetry 90 to < 95%	Dyspnea at rest causing inability to perform usual social & functional activities OR Pulse oximetry < 90%	Respiratory failure with ventilator support indicated (e.g., CPAP, BPAP, intubation)

This copy of the document was retrieved from the system by Lisa Sunner on 24 Apr 2019

SENSORY

PARAMETER	GRADE 1 MILD	GRADE 2 MODERATE	GRADE 3 SEVERE	GRADE 4 POTENTIALLY LIFE-THREATENING
**Hearing Loss**<br>*≥ 12 years of age*	NA	Hearing aid or intervention not indicated	Hearing aid or intervention indicated	Profound bilateral hearing loss (> 80 dB at 2 kHz and above) OR Non-serviceable hearing (i.e., >50 dB audiogram and <50% speech discrimination)
< 12 years of age (based on a 1, 2, 3, 4, 6 and 8 kHz audiogram)	> 20 dB hearing loss at ≤ 4 kHz	> 20 dB hearing loss at > 4 kHz	> 20 dB hearing loss at ≥ 3 kHz in one ear with additional speech language related services indicated (where available) OR<br>Hearing loss sufficient to indicate therapeutic intervention, including hearing aids	Audiologic indication for cochlear implant and additional speech-language related services indicated (where available)
**Tinnitus**	Symptoms causing no or minimal interference with usual social & functional activities with intervention not indicated	Symptoms causing greater than minimal interference with usual social & functional activities with intervention indicated	Symptoms causing inability to perform usual social & functional activities	NA
**Uveitis**	No symptoms AND Detectable on examination	Anterior uveitis with symptoms OR Medicamylasal intervention indicated	Posterior or pan-uveitis OR Operative intervention indicated	Disabling visual loss in affected eye(s)
**Vertigo**	Vertigo causing no or minimal interference with usual social & functional activities	Vertigo causing greater than minimal interference with usual social & functional activities	Vertigo causing inability to perform usual social & functional activities	Disabling vertigo causing inability to perform basic self-care functions
**Visual Changes** (assessed from baseline)	Visual changes causing no or minimal interference with usual social & functional activities	Visual changes causing greater than minimal interference with usual social & functional activities	Visual changes causing inability to perform usual social & functional activities	Disabling visual loss in affected eye(s)

TMF-03635 | 4.0 | Status:Approved | 16 Apr 2019
This copy of the document was retrieved from the system by Lisa Sunner on 24 Apr 2019

SYSTEMIC

PARAMETER	GRADE 1 MILD	GRADE 2 MODERATE	GRADE 3 SEVERE	GRADE 4 POTENTIALLY LIFE-THREATENING
**Acute Allergic Reaction**	Localized urticaria (wheals) with no medical intervention indicated	Localized urticaria with intervention indicated OR Mild angioedema with no intervention indicated	Generalized urticaria OR Angioedema with intervention indicated OR Symptoms of mild bronchospasm	Acute anaphylaxis OR Life-threatening bronchospasm OR Laryngeal edema
**Chills**	Symptoms causing no or minimal interference with usual social & functional activities	Symptoms causing greater than minimal interference with usual social & functional activities	Symptoms causing inability to perform usual social & functional activities	NA
**Cytokine Release Syndrome**[9]	Mild signs and symptoms AND Therapy (i.e., antibody infusion) interruption not indicated	Therapy (i.e., antibody infusion) interruption indicated AND Responds promptly to symptomatic treatment OR Prophylactic medications indicated for ≤ 24 hours	Prolonged severe signs and symptoms OR Recurrence of symptoms following initial improvement	Life-threatening consequences (e.g., requiring pressor or ventilator support)
**Fatigue or Malaise** *Report only one*	Symptoms causing no or minimal interference with usual social & functional activities	Symptoms causing greater than minimal interference with usual social & functional activities	Symptoms causing inability to perform usual social & functional activities	Incapacitating symptoms of fatigue or malaise causing inability to perform basic self-care functions
**Fever** (non-axillary temperatures only)	38.0 to < 38.6°C or 100.4 to < 101.5°F	≥ 38.6 to < 39.3°C or ≥101.5 to < 102.7°F	≥ 39.3 to < 40.0°C or ≥ 102.7 to < 104.0°F	≥ 40.0°C or ≥ 104.0°F
**Pain**[10] (not associated with study agent injections and not specified elsewhere) *Specify location*	Pain causing no or minimal interference with usual social & functional activities	Pain causing greater than minimal interference with usual social & functional activities	Pain causing inability to perform usual social & functional activities	Disabling pain causing inability to perform basic self-care functions OR Hospitalization indicated
**Serum Sickness**[11]	Mild signs and symptoms	Moderate signs and symptoms AND Intervention indicated (e.g., antihistamines)	Severe signs and symptoms AND Higher level intervention indicated (e.g., steroids or IV fluids)	Life-threatening consequences (e.g., requiring pressor or ventilator support)

[9] Definition: A disorder characterized by nausea, headache, tachycardia, hypotension, rash, and/or shortness of breath.
[10] For pain associated with injections or infusions, see the *Site Reactions to Injections and Infusions* section (page 23).
[11] Definition: A disorder characterized by fever, arthralgia, myalgia, skin eruptions, lymphadenopathy, marked discomfort, and/or dyspnea.

This copy of the document was retrieved from the system by Lisa Sunner on 24 Apr 2019

SYSTEMIC

PARAMETER	GRADE 1 MILD	GRADE 2 MODERATE	GRADE 3 SEVERE	GRADE 4 POTENTIALLY LIFE-THREATENING
**Underweight**[12] *> 5 to 19 years of age*	NA	WHO BMI z-score < -2 to ≤ -3	WHO BMI z-score < -3	WHO BMI z-score < -3 with life-threatening consequences
2 to 5 years of age	NA	WHO Weight-for-height z-score < -2 to ≤-3	WHO Weight-for-height z-score < -3	WHO Weight-for-height z-score < -3 with life-threatening consequences
< 2 years of age	NA	WHO Weight-for-length z-score < -2 to ≤-3	WHO Weight-for-length z-score < -3	WHO Weight-for-length z-score < -3 with life-threatening consequences
**Weight Loss** (excludes postpartum weight loss)	NA	5 to < 9% loss in body weight from baseline	≥ 9 to < 20% loss in body weight from baseline	≥ 20% loss in body weight from baseline OR Aggressive intervention indicated (e.g., tube feeding, total parenteral nutrition)

12 WHO reference tables may be accesed by clicking the desired age range or by accessing the following URLs: http://www.who.int/growthref/who2007 bmi for age/en/ for participants > 5 to 19 years of age and http://www.who.int/childgrowth/standards/chart catalogue/en/ for those < 5 years of age.

URINARY

PARAMETER	GRADE 1 MILD	GRADE 2 MODERATE	GRADE 3 SEVERE	GRADE 4 POTENTIALLY LIFE-THREATENING
**Urinary Tract Obstruction**	NA	Signs or symptoms of urinary tract obstruction without hydronephrosis or renal dysfunction	Signs or symptoms of urinary tract obstruction with hydronephrosis or renal dysfunction	Obstruction causing life-threatening consequences

This copy of the document was retrieved from the system by Lisa Sunner on 24 Apr 2019

SITE REACTIONS TO INJECTIONS AND INFUSIONS

PARAMETER	GRADE 1 MILD	GRADE 2 MODERATE	GRADE 3 SEVERE	GRADE 4 POTENTIALLY LIFE-THREATENING
**Injection Site Pain or Tenderness** *Report only one*	Pain or tenderness causing no or minimal limitation of use of limb	Pain or tenderness causing greater than minimal limitation of use of limb	Pain or tenderness causing inability to perform usual social & functional activities	Pain or tenderness causing inability to perform basic self-care function OR Hospitalization indicated
**Injection Site Erythema or Redness**[13] *Report only one* *> 15 years of age*	2.5 to < 5 cm in diameter OR 6.25 to < 25 cm^2 surface area AND Symptoms causing no or minimal interference with usual social & functional activities	≥ 5 to < 10 cm in diameter OR ≥ 25 to < 100 cm^2 surface area OR Symptoms causing greater than minimal interference with usual social & functional activities	≥ 10 cm in diameter OR ≥ 100 cm^2 surface area OR Ulceration OR Secondary infection OR Phlebitis OR Sterile abscess OR Drainage OR Symptoms causing inability to perform usual social & functional activities	Potentially life-threatening consequences (e.g., abscess, exfoliative dermatitis, necrosis involving dermis or deeper tissue)
≤15 years of age	≤ 2.5 cm in diameter	> 2.5 cm in diameter with < 50% surface area of the extremity segment involved (e.g., upper arm or thigh)	≥ 50% surface area of the extremity segment involved (e.g., upper arm or thigh) OR Ulceration OR Secondary infection OR Phlebitis OR Sterile abscess OR Drainage	Potentially life-threatening consequences (e.g., abscess, exfoliative dermatitis, necrosis involving dermis or deeper tissue)
**Injection Site Induration or Swelling** *Report only one* *> 15 years of age*	Same as for **Injection Site Erythema or Redness,** *> 15 years of age*	Same as for **Injection Site Erythema or Redness,** *> 15 years of age*	Same as for **Injection Site Erythema or Redness,** *> 15 years of age*	Same as for **Injection Site Erythema or Redness,** *> 15 years of age*
≤ 15 years of age	Same as for **Injection Site Erythema or Redness,** *≤ 15 years of age*	Same as for **Injection Site Erythema or Redness,** *≤ 15 years of age*	Same as for **Injection Site Erythema or Redness,** *≤ 15 years of age*	Same as for **Injection Site Erythema or Redness,** *≤ 15 years of age*
**Injection Site Pruritus**	Itching localized to the injection site that is relieved spontaneously or in < 48 hours of treatment	Itching beyond the injection site that is not generalized OR Itching localized to the injection site requiring ≥ 48 hours treatment	Generalized itching causing inability to perform usual social & functional activities	NA

[13] Injection Site Erythema or Redness should be evaluated and graded using the greatest single diameter or measured surface area.

Laboratory Values Chemistries

PARAMETER	GRADE 1 MILD	GRADE 2 MODERATE	GRADE 3 SEVERE	GRADE 4 POTENTIALLY LIFE-THREATENING
**Acidosis**	NA	pH ≥ 7.3 to < LLN	pH < 7.3 without life-threatening consequences	pH < 7.3 with life-threatening consequences
**Albumin, Low** (g/dL; *g/L*)	3.0 to < LLN<br>*30 to < LLN*	≥ 2.0 to < 3.0<br>*≥ 20 to < 30*	< 2.0<br>*< 20*	NA
**Alkaline Phosphatase, High**	1.25 to < 2.5 x ULN	2.5 to < 5.0 x ULN	5.0 to < 10.0 x ULN	≥ 10.0 x ULN
**Alkalosis**	NA	pH > ULN to ≤ 7.5	pH > 7.5 without life-threatening consequences	pH > 7.5 with life-threatening consequences
**ALT or SGPT, High**<br>*Report only one*	1.25 to < 2.5 x ULN	2.5 to < 5.0 x ULN	5.0 to < 10.0 x ULN	≥ 10.0 x ULN
**Amylase (Pancreatic) or Amylase (Total), High**<br>*Report only one*	1.1 to < 1.5 x ULN	1.5 to < 3.0 x ULN	3.0 to < 5.0 x ULN	≥ 5.0 x ULN
**AST or SGOT, High**<br>*Report only one*	1.25 to < 2.5 x ULN	2.5 to < 5.0 x ULN	5.0 to < 10.0 x ULN	≥ 10.0 x ULN
**Bicarbonate, Low** (mEq/L; *mmol/L*)	16.0 to < LLN<br>*16.0 to < LLN*	11.0 to < 16.0<br>*11.0 to < 16.0*	8.0 to < 11.0<br>*8.0 to < 11.0*	< 8.0<br>*< 8.0*
**Bilirubin**<br>***Direct Bilirubin[14], High***<br>*> 28 days of age*	NA	NA	> ULN with other signs and symptoms of hepatoxicity.	> ULN with life-threatening consequences (e.g., signs and symptoms of liver failure)
≤ 28 days of age	ULN to ≤ 1 mg/dL	> 1 to ≤ 1.5 mg/dL	> 1.5 to ≤ 2 mg/dL	> 2 mg/dL
Total Bilirubin, High<br>*> 28 days of age*	1.1 to < 1.6 x ULN	1.6 to < 2.6 x ULN	2.6 to < 5.0 x ULN	≥ 5.0 x ULN
≤ 28 days of age	See Appendix A. Total Bilirubin for Term and Preterm Neonates	See Appendix A. Total Bilirubin for Term and Preterm Neonates	See Appendix A. Total Bilirubin for Term and Preterm Neonates	See Appendix A. Total Bilirubin for Term and Preterm Neonates
**Calcium, High** (mg/dL; *mmol/L*)<br>*≥ 7 days of age*	10.6 to < 11.5<br>*2.65 to < 2.88*	11.5 to < 12.5<br>*2.88 to < 3.13*	12.5 to < 13.5<br>*3.13 to < 3.38*	≥ 13.5<br>*≥ 3.38*
< 7 days of age	11.5 to < 12.4<br>*2.88 to < 3.10*	12.4 to < 12.9<br>*3.10 to < 3.23*	12.9 to < 13.5<br>*3.23 to < 3.38*	≥ 13.5<br>*≥ 3.38*

[14] Direct bilirubin > 1.5 mg/dL in a participant < 28 days of age should be graded as grade 2, if < 10% of the total bilirubin.

This copy of the document was retrieved from the system by Lisa Sunner on 24 Apr 2019

PARAMETER	GRADE 1 MILD	GRADE 2 MODERATE	GRADE 3 SEVERE	GRADE 4 POTENTIALLY LIFE-THREATENING
**Calcium (Ionized), High** (mg/dL; *mmol/L*)	> ULN to < 6.0<br>*> ULN to < 1.5*	6.0 to < 6.4<br>*1.5 to < 1.6*	6.4 to < 7.2<br>*1.6 to < 1.8*	≥ 7.2<br>*≥ 1.8*
**Calcium, Low** (mg/dL; *mmol/L*)<br>*≥ 7 days of age*	7.8 to < 8.4<br>*1.95* to *< 2.10*	7.0 to < 7.8<br>*1.75* to *< 1.95*	6.1 to < 7.0<br>*1.53* to *< 1.75*	< 6.1<br>*< 1.53*
< 7 days of age	6.5 to < 7.5<br>*1.63* to *< 1.88*	6.0 to < 6.5<br>*1.50* to *< 1.63*	5.50 to < 6.0<br>*1.38* to *< 1.50*	< 5.50<br>*< 1.38*
**Calcium (Ionized), Low** (mg/dL; *mmol/L*)	< LLN to 4.0<br>*< LLN to 1.0*	3.6 to < 4.0<br>*0.9 to < 1.0*	3.2 to < 3.6<br>*0.8 to < 0.9*	< 3.2<br>*< 0.8*
**Cardiac Troponin I, High**	NA	NA	NA	Levels consistent with myocardial infarction or unstable angina as defined by the local laboratory
**Creatine Kinase, High**	3 to < 6 x ULN	6 to < 10 x ULN	10 to < 20 x ULN	≥ 20 x ULN
**Creatinine, High**	1.1 to 1.3 x ULN	> 1.3 to 1.8 x ULN OR Increase of > 0.3 mg/dL above baseline	> 1.8 to < 3.5 x ULN OR Increase of 1.5 to < 2.0 x above baseline	≥ 3.5 x ULN OR Increase of ≥ 2.0 x above baseline
**Creatinine Clearance**[15] **or eGFR, Low**<br>*Report only one*	NA	< 90 to 60 ml/min or ml/min/1.73 m^2 OR 10 to < 30% decrease from baseline	< 60 to 30 ml/min or ml/min/1.73 m^2 OR ≥ 30 to < 50% decrease from baseline	< 30 ml/min or ml/min/1.73 m^2 OR ≥ 50% decrease from baseline or dialysis needed
**Glucose** (mg/dL; *mmol/L*)<br>***Fasting, High***	110 to 125<br>*6.11 to < 6.95*	> 125 to 250<br>*6.95 to < 13.89*	> 250 to 500<br>*13.89 to < 27.75*	> 500<br>*≥ 27.75*
Nonfasting, High	116 to 160<br>*6.44 to < 8.89*	> 160 to 250<br>*8.89 to < 13.89*	> 250 to 500<br>*13.89 to < 27.75*	> 500<br>*≥ 27.75*
**Glucose, Low** (mg/dL; *mmol/L*)<br>*≥ 1 month of age*	55 to 64	40 to < 55	30 to < 40	< 30
< 1 month of age	50 to 54<br>*2.78 to 3.00*	40 to < 50<br>*2.22 to < 2.78*	30 to < 40<br>*1.67 to < 2.22*	< 30<br>*< 1.67*
**Lactate, High**	ULN to < 2.0 x ULN without acidosis	≥ 2.0 x ULN without acidosis	Increased lactate with pH < 7.3 without life-threatening consequences	Increased lactate with pH < 7.3 with life-threatening consequences

[15] Use the applicable formula (i.e., Cockroft-Gault in mL/min or Schwatrz in mL/min/1.73m^2).

This copy of the document was retrieved from the system by Lisa Sunner on 24 Apr 2019

CHEMISTRIES

PARAMETER	GRADE 1 MILD	GRADE 2 MODERATE	GRADE 3 SEVERE	GRADE 4 POTENTIALLY LIFE-THREATENING
**Lipase, High**	1.1 to < 1.5 x ULN	1.5 to < 3.0 x ULN	3.0 to < 5.0 x ULN	≥ 5.0 x ULN
**Lipid Disorders** (mg/dL; *mmol/L*) ***Cholesterol, Fasting, High*** *≥18 years of age*	200 to < 240 *5.18 to < 6.19*	240 to < 300 *6.19 to < 7.77*	≥ 300 *≥ 7.77*	NA
< 18 years of age	170 to < 200 *4.40 to < 5.15*	200 to < 300 *5.15 to < 7.77*	≥ 300 *≥ 7.77*	NA
LDL, Fasting, High *≥18 years of age*	130 to < 160 *3.37 to < 4.12*	160 to < 190 *4.12 to < 4.90*	≥ 190 *≥ 4.90*	NA
> 2 to < 18 years of age	110 to < 130 *2.85 to < 3.34*	130 to < 190 *3.34 to < 4.90*	≥ 190 *≥ 4.90*	NA
Triglycerides, Fasting, High	150 to 300 *1.71 to 3.42*	>300 to 500 *>3.42 to 5.7*	>500 to < 1,000 *>5.7 to 11.4*	> 1,000 *> 11.4*
**Magnesium[16], Low** (mEq/L; *mmol/L*)	1.2 to < 1.4 *0.60 to < 0.70*	0.9 to < 1.2 *0.45 to < 0.60*	0.6 to < 0.9 *0.30 to < 0.45*	< 0.6 *< 0.30*
**Phosphate, Low** (mg/dL; *> 14 years of age*	2.0 to < LLN *0.81 to < LLN*	1.4 to < 2.0 *0.65 to < 0.81*	1.0 to < 1.4 *0.32 to < 0.65*	< 1.0 *< 0.32*
1 to 14 years of age	3.0 to < 3.5 *0.97 to < 1.13*	2.5 to < 3.0 *0.81 to < 0.97*	1.5 to < 2.5 *0.48 to < 0.81*	< 1.5 *< 0.48*
< 1 year of age	3.5 to < 4.5 *1.13 to < 1.45*	2.5 to < 3.5 *0.81 to < 1.13*	1.5 to < 2.5 *0.48 to < 0.81*	< 1.5 *< 0.48*
**Potassium, High** (mEq/L; *mmol/L*)	5.6 to < 6.0 *5.6 to < 6.0*	6.0 to < 6.5 *6.0 to < 6.5*	6.5 to < 7.0 *6.5 to < 7.0*	≥ 7.0 *≥ 7.0*
**Potassium, Low** (mEq/L; *mmol/L*)	3.0 to < 3.4 *3.0 to < 3.4*	2.5 to < 3.0 *2.5 to < 3.0*	2.0 to < 2.5 *2.0 to < 2.5*	< 2.0 *< 2.0*
**Sodium, High** (mEq/L; *mmol/L*)	146 to < 150 *146 to < 150*	150 to < 154 *150 to < 154*	154 to < 160 *154 to < 160*	≥ 160 *≥ 160*
**Sodium, Low** (mEq/L; *mmol/L*)	130 to < 135 *130 to < 135*	125 to < 130 *125 to < 135*	121 to < 125 *121 to < 125*	≤ 120 *≤ 120*
**Uric Acid, High** (mg/dL; *mmol/L*)	7.5 to < 10.0 *0.45 to < 0.59*	10.0 to < 12.0 *0.59 to < 0.71*	12.0 to < 15.0 *0.71 to < 0.89*	≥ 15.0 *≥ 0.89*

[16] To convert a magnesium value from mg/dL to mmol/L, laboratories should multiply by 0.4114.

This copy of the document was retrieved from the system by Lisa Sunner on 24 Apr 2019

HEMATOLOGY

PARAMETER	GRADE 1 MILD	GRADE 2 MODERATE	GRADE 3 SEVERE	GRADE 4 POTENTIALLY LIFE-THREATENING
**Absolute CD4+ Count, Low** (cell/mm^3; *cells/L*)<br>*> 5 years of age (not HIV infected)*	300 to < 400<br>*300 to < 400*	200 to < 300<br>*200 to < 300*	100 to < 200<br>*100 to < 200*	< 100<br>*< 100*
**Absolute Lymphocyte Count, Low** (cell/mm^3; *cells/L*)<br>*> 5 years of age (not HIV infected)*	600 to < 650<br>*0.600 x 10^9 to < 0.650 x 10^9*	500 to < 600<br>*0.500 x 10^9 to < 0.600 x 10^9*	350 to < 500<br>*0.350 x 10^9 to < 0.500 x 10^9*	< 350<br>*< 0.350 x 10^9*
**Absolute Neutrophil Count (ANC), Low** (cells/mm^3; *cells/L*)<br>*> 7 days of age*	800 to 1,000<br>*0.800 x 10^9 to 1.000 x 10^9*	600 to 799<br>*0.600 x 10^9 to 0.799 x 10^9*	400 to 599<br>*0.400 x 10^9 to 0.599 x 10^9*	< 400<br>*< 0.400 x 10^9*
2 to 7 days of age	1,250 to 1,500<br>*1.250 x 10^9 to 1.500 x 10^9*	1,000 to 1,249<br>*1.000 x 10^9 to 1.249 x 10^9*	750 to 999<br>*0.750 x 10^9 to 0.999 x 10^9*	< 750<br>*< 0.750 x 10^9*
≤ 1 day of age	4,000 to 5,000<br>*4.000 x 10^9 to 5.000 x 10$_9$*	3,000 to 3,999<br>*3.000 x 10^9 to 3.999 x 10^9*	1,500 to 2,999<br>*1.500 x 10^9 to 2.999 x 10^9*	< 1,500<br>*< 1.500 x 10^9*
**Fibrinogen, Decreased** (mg/dL; *g/L*)	100 to < 200<br>*1.00 to < 2.00*<br>OR<br>0.75 to < 1.00 x LLN	75 to < 100<br>*0.75 to < 1.00*<br>OR<br>≥ 0.50 to < 0.75 x LLN	50 to < 75<br>*0.50 to < 0.75*<br>OR<br>0.25 to < 0.50 x LLN	< 50<br>*< 0.50*<br>OR<br>< 0.25 x LLN<br>OR Associated with gross bleeding
**Hemoglobin[17], Low** (g/dL; *mmol/L*)[18]<br>*≥ 13 years of age (male only)*	10.0 to 10.9<br>*6.19 to 6.76*	9.0 to < 10.0<br>*5.57 to < 6.19*	7.0 to < 9.0<br>*4.34 to < 5.57*	< 7.0<br>*< 4.34*
≥ 13 years of age (female only)	9.5 to 10.4<br>*5.88 to 6.48*	8.5 to < 9.5<br>*5.25 to < 5.88*	6.5 to < 8.5<br>*4.03 to < 5.25*	< 6.5<br>*< 4.03*

[17] Male and female sex are defined as sex at birth.
[18] The conversion factor used to convert g/dL to mmol/L is 0.6206 and is the most commonly used conversion factor. For grading hemoglobin results obtained by an analytic method with a conversion factor other than 0.6206, the result must be converted to g/dL using the appropriate conversion factor for the particular laboratory

TMF-03635 | 4.0 | Status:Approved | 16 Apr 2019
This copy of the document was retrieved from the system by Lisa Sunner on 24 Apr 2019

PARAMETER	GRADE 1 MILD	GRADE 2 MODERATE	GRADE 3 SEVERE	GRADE 4 POTENTIALLY LIFE-THREATENING
57 days of age to < 13 years of age (male and female)	9.5 to 10.4<br>*5.88 to 6.48*	8.5 to < 9.5<br>*5.25 to < 5.88*	6.5 to < 8.5<br>*4.03 to < 5.25*	< 6.5<br>*< 4.03*
36 to 56 days of age (male and female)	8.5 to 9.6<br>*5.26 to 5.99*	7.0 to < 8.5<br>*4.32 to < 5.26*	6.0 to < 7.0<br>*3.72 to < 4.32*	< 6.0<br>*< 3.72*
22 to 35 days of age (male and female)	9.5 to 11.0<br>*5.88 to 6.86*	8.0 to < 9.5<br>*4.94 to < 5.88*	6.7 to < 8.0<br>*4.15 to < 4.94*	< 6.7<br>*< 4.15*
8 to ≤ 21 days of age (male and female)	11.0 to 13.0<br>*6.81 to 8.10*	9.0 to < 11.0<br>*5.57 to < 6.81*	8.0 to < 9.0<br>*4.96 to < 5.57*	< 8.0<br>*< 4.96*
≤ 7 days of age (male and female)	13.0 to 14.0<br>*8.05 to 8.72*	10.0 to < 13.0<br>*6.19 to < 8.05*	9.0 to < 10.0<br>*5.59 to < 6.19*	< 9.0<br>*< 5.59*
**INR, High** (not on anticoagulation therapy)	1.1 to < 1.5 x ULN	1.5 to < 2.0 x ULN	2.0 to < 3.0 x ULN	≥ 3.0 x ULN
**Methemoglobin** (% hemoglobin)	5.0 to < 10.0%	10.0 to < 15.0%	15.0 to < 20.0%	≥ 20.0%
**PTT, High** (not on anticoagulation therapy)	1.1 to < 1.66 x ULN	1.66 to < 2.33 x ULN	2.33 to < 3.00 x ULN	≥ 3.00 x ULN
**Platelets, Decreased** (cells/mm^3; *cells/L*)	100,000 to < 124,999<br>*100.000 x 10^9 to < 124.999 x 10^9*	50,000 to < 100,000<br>*50.000 x 10^9 to < 100.000 x 10^9*	25,000 to < 50,000<br>*25.000 x 10^9 to < 50.000 x 10^9*	< 25,000<br>*< 25.000 x 10^9*
**PT, High** (not on anticoagulation therapy	1.1 to < 1.25 x ULN	1.25 to < 1.50 x ULN	1.50 to < 3.00 x ULN	≥ 3.00 x ULN
**WBC, Decreased** (cells/mm^3; *cells/L*)				
> 7 days of age	2,000 to 2,499<br>*2.000 x 10^9 to 2.499 x 10^9*	1,500 to 1,999<br>*1.500 x 10^9 to 1.999 x 10^9*	1,000 to 1,499<br>*1.000 x 10^9 to 1.499 x 10^9*	< 1,000<br>*< 1.000 x 10^9*
≤ 7 days of age	5,500 to 6,999<br>*5.500 x 10^9 to 6.999 x 10^9*	4,000 to 5,499<br>*4.000 x 10^9 to 5.499 x 10^9*	2,500 to 3,999<br>*2.500 x 10^9 to 3.999 x 10^9*	< 2,500<br>*< 2.500 x 10^9*

This copy of the document was retrieved from the system by Lisa Sunner on 24 Apr 2019

URINALYSIS

PARAMETER	GRADE 1 MILD	GRADE 2 MODERATE	GRADE 3 SEVERE	GRADE 4 POTENTIALLY LIFE-THREATENING
**Glycosuria** (random collection tested by dipstick)	Trace to 1+ or ≤ 250 mg	2+ or > 250 to < 500 mg	> 2+ or > 500 mg	NA
**Hematuria** (not to be reported based on dipstick findings or on blood believed to be of menstrual origin)	6 to < 10 RBCs per high power field	≥ 10 RBCs per high power field	Gross, with or without clots OR With RBC casts OR Intervention indicated	Life-threatening consequences
**Proteinuria** (random collection tested by dipstick)	1+	2+	3+ or higher	NA

This copy of the document was retrieved from the system by Lisa Sunner on 24 Apr 2019

Signature Page for TMF-03635 v4.0

Reason for signing: Approved	Name: Fran Priddy<br>Role: Chief Medical Officer<br>Date of signature: 05-Apr-2019 19:37:23 GMT+0000

Reason for signing: Approved	Name: Carl Verlinde<br>Role: Medical Affairs Document Review Team<br>Date of signature: 15-Apr-2019 14:50:22 GMT+0000

Signature Page for TMF-03635 v4.0
