# Supplementary material for: Safety and antiviral activity of triple combination broadly neutralizing monoclonal antibody therapy against HIV-1: a phase 1 clinical trial
Source: Nat Med. 2022 May 12;28(6):1288–96. doi: 10.1038/s41591-022-01815-1 (PMC9205771; doi:10.1038/s41591-022-01815-1)
Supplement: Supplementary file 2 — Reporting Summary [file 41591_2022_1815_MOESM2_ESM.pdf]

Corresponding author(s): Dan Barouch

Last updated by author(s): Mar 22, 2022

Reporting Summary

Nature Portfolio wishes to improve the reproducibility of the work that we publish. This form provides structure for consistency and transparency in reporting. For further information on Nature Portfolio policies, see our Editorial Policies and the Editorial Policy Checklist.

Statistics

For all statistical analyses, confirm that the following items are present in the figure legend, table legend, main text, or Methods section.

n/a	Confirmed	
☐	☒	The exact sample size (*n*) for each experimental group/condition, given as a discrete number and unit of measurement
☐	☒	A statement on whether measurements were taken from distinct samples or whether the same sample was measured repeatedly
☐	☒	The statistical test(s) used AND whether they are one- or two-sided<br>*Only common tests should be described solely by name; describe more complex techniques in the Methods section.*
☐	☒	A description of all covariates tested
☐	☒	A description of any assumptions or corrections, such as tests of normality and adjustment for multiple comparisons
☐	☒	A full description of the statistical parameters including central tendency (e.g. means) or other basic estimates (e.g. regression coefficient) AND variation (e.g. standard deviation) or associated estimates of uncertainty (e.g. confidence intervals)
☐	☒	For null hypothesis testing, the test statistic (e.g. *F*, *t*, *r*) with confidence intervals, effect sizes, degrees of freedom and *P* value noted<br>*Give P values as exact values whenever suitable.*
☒	☐	For Bayesian analysis, information on the choice of priors and Markov chain Monte Carlo settings
☐	☒	For hierarchical and complex designs, identification of the appropriate level for tests and full reporting of outcomes
☐	☒	Estimates of effect sizes (e.g. Cohen's *d*, Pearson's *r*), indicating how they were calculated

Our web collection on statistics for biologists contains articles on many of the points above.

Software and code

Policy information about availability of computer code

Data collection	eClinical V3.7
Data analysis	Monolix (version 2019R1, Antony, France: Lixoft SAS, 2019) for standard two-compartment population models using the Stochastic Approximation Expectation-Maximization (SAEM) estimation method (PK analysis).<br>Env gene sequences were extracted and codon-aligned using the webtool Gene Cutter on the Los Alamos HIV database (https://www.hiv.lanl.gov/content/sequence/GENE_CUTTER/cutter.html)<br>Highlighter plots were generated using the Highlighter webtool on the Los Alamos HIV database (https://www.hiv.lanl.gov/content/sequence/HIGHLIGHT/highlighter_top.html).<br>Phylogenetic tree for all participant viruses combined was inferred using Env nucleotide alignments using the IQ-TREE algorithm as implemented on the Los Alamos HIV Database (https://www.hiv.lanl.gov/content/sequence/IQTREE/iqtree.html).<br>Recombination analyses were performed using RAPR on the Los Alamos HIV database (https://www.hiv.lanl.gov/content/sequence/RAP2017/rap.html).<br>Sequence logos were obtained from the web tool AnalyzeAlign on the Los Alamos HIV database (https://www.hiv.lanl.gov/content/sequence/ANALYZEALIGN/analyze_align.html).<br>Recombination analyses were conducted using the LANL tool RAPR (https://www.hiv.lanl.gov/content/sequence/RAP2017/rap.html).<br>bNAb combinations against global heterologous viruses was predicted using the Bliss-Hill model as implemented in the webtool CombiNAber (https://www.hiv.lanl.gov/content/sequence/COMBINABER/combinaber.html).

For manuscripts utilizing custom algorithms or software that are central to the research but not yet described in published literature, software must be made available to editors and reviewers. We strongly encourage code deposition in a community repository (e.g. GitHub). See the Nature Portfolio guidelines for submitting code & software for further information.

Data

Policy information about availability of data

All manuscripts must include a data availability statement. This statement should provide the following information, where applicable:

- Accession codes, unique identifiers, or web links for publicly available datasets
- A description of any restrictions on data availability
- For clinical datasets or third party data, please ensure that the statement adheres to our policy

All viral sequences identified in this study are publicly available via GenBank (see Supplementary Table 12 for GenBank accession numbers). Comprehensive data on HIV genetic sequences and immunological epitopes used for analysis in this study are publicly available via Los Alamos National Laboratory (hiv.lanl.gov/content/index). Additional requests for access to the study data can be submitted to D.H.B. (dbarouch@bidmc.harvard.edu). Data containing protected health information or that may identify a participant are restricted, and therefore additional data requests must be reviewed before release.

Field-specific reporting

Please select the one below that is the best fit for your research. If you are not sure, read the appropriate sections before making your selection.

☒ Life sciences ☐ Behavioural & social sciences ☐ Ecological, evolutionary & environmental sciences

For a reference copy of the document with all sections, see nature.com/documents/nr-reporting-summary-flat.pdf

Life sciences study design

All studies must disclose on these points even when the disclosure is negative.

Sample size	(1) The sample size for safety and tolerability analysis was 30-66 participants according to the dose escalation design used to characterize the safety profile of one IV infusion of PGDM1400 mAb ± PGT121 mAb, at one of 3 dose levels. For life-threatening adverse events related to active product: if none of the 9 (max 18) participants in either Group 1 or Group 2 who receive the active product experience such reactions then the exact 95% upper confidence bound for the rate of these adverse events in the population is 33.6% (or 18.5% if n=18). This was an exploratory proof of concept trial and the analysis was descriptive, and no formal null hypothesis was tested. The frequency of moderate or greater reactogenicity events was determined and compared between groups. The frequency of SAEs judged possibly, probably or related to the IP was determined. All AEs were analyzed and, grouped by seriousness, severity and relationship to the IP (as judged by the investigators). An interim safety analysis of group data was carried out after each dose escalation according to the study schema without unblinding the study to investigators or participants. At the end of the study, a full analysis was prepared. Missing data was excluded from the statistical analysis. (2) The sample size for pharmacokinetic (PK) analysis was 3 per dose sub-group, sufficient for the planned analyses based on prior experience with PGT121 pharmacokinetics. The data were fit to standard two-compartment population models using the Stochastic Approximation Expectation-Maximization (SAEM) estimation method in Monolix (version 2019R1, Antony, France: Lixoft SAS, 2019). Population (non-linear mixed effects) PK (popPK) models were fit separately by analyte and HIV infection status. Fixed effects were used to model the population-level PK parameters and random effects were used to model the individual-level variability. The area under the concentration curve (AUC) was estimated by calculating the integral of the predicted concentration-time curve from the first infusion time to infinity. Additionally, peak concentration (Cmax) was computed as the maximum observed concentration. Summary descriptive results of PK parameters, including AUC, Cmax, T1/2, and clearance results were reported by bNAb and dose cohort. For each analyte, a Spearman correlation test was conducted to test for correlation between elimination half-life, clearance, volume of distribution, and dose- and weight-adjusted AUC with log10 viral load at baseline (null hypothesis: $\rho = 0$; $\alpha = 0.05$) using mid-ranks for tied scores and the approximate distribution. Correlation between PK and reported safety and pharmacodynamic outcomes were also explored parameters in order to examine exposure-effect relationships. The concordance correlation coefficient (CCC) was used to assess the concordance between the log10 concentrations from the binding and neutralizing antibody assays. (3) The sample size for virologic analysis was 6-18 participants across groups 3A and 3B. No placebo participants were enrolled in part 2 as per study design. For each participant, viral load difference-from-baseline was defined as the difference in Day 7 log10 plasma HIV-1 RNA levels from baseline (mean, on log10 scale, of screening and day 0 levels). Based on a simulation study, power to reject the null hypothesis was 80% when the responder group has a difference-from-baseline viral load drop of approximately 1.8 logs for a nominal alpha level of 0.05. As the study under enrolled for Group 3, the virologic outcome was not formally analyzed.
Data exclusions	None
Replication	This section does not apply to our study which was a clinical trial with unique participants who could not be replicated. There were, however, 29 participants enrolled who received some of the same interventions as outlined below.
Randomization	In Part 1, eligible participants were enrolled first into the lowest dose sub-group of PGDM1400 alone (Group 1A), and enrollment into the lowest PGDM1400 and PGT121 combination dose sub-group (Group 2A) only occurred after the Protocol Safety Review Team (PSRT) has reviewed the safety data through day 14 post administration of PGDM1400 alone and had approved dose escalation. This staggered dose escalation was continued for each dose group. Participants in each sub-group were identified by a unique study identification number. Participants were randomized according to the randomization schedule prepared by the statisticians at the Data Coordinating Center (DCC, Emmes Company, LLC) prior to the start of the study. Participants were automatically assigned a specific allocation number as they were enrolled into the data entry system. In Part 1, the 4 participants in each dose level sub-group (3mg/kg, 10mg/kg or 30mg/kg) in Group 1 and (3+3mg/kg, 10+10mg/kg or 30+30mg/kg) in Group 2 were randomized at a ratio of three antibody recipients to one placebo recipient, respectively (total of 9 antibody and 3 placebo recipients per Group). At each dose level in Part 1, IP administration was separated by at least 24 hours for each of the first 3 participants. Randomization in Part 1 ensured that at least 2 participants received active product and were

observed for at least 24 hours before administration to additional participants. IP administration was also separated by at least 24 hours for each of the first 3 participants in Part 2, Group 3A, that received the triple bNAb combination.

Blinding

An unblinding list (Pharmacy List) was provided to the unblinded site pharmacist by the DCC. Study staff (investigator and clinical personnel monitoring the safety and laboratory assay results) and participants were blinded with respect to the allocation of investigational product (IP). A site pharmacist was unblinded for the purposes of preparing the IP. Blinded participants were informed about their assignment (product/placebo) at study completion, once the data was locked. As the bNAbs and placebo (saline) looked identical in the infusion bag, no masking was required.

Reporting for specific materials, systems and methods

We require information from authors about some types of materials, experimental systems and methods used in many studies. Here, indicate whether each material, system or method listed is relevant to your study. If you are not sure if a list item applies to your research, read the appropriate section before selecting a response.

Materials & experimental systems

n/a	Involved in the study
☐	☒ Antibodies
☒	☐ Eukaryotic cell lines
☒	☐ Palaeontology and archaeology
☒	☐ Animals and other organisms
☐	☒ Human research participants
☐	☒ Clinical data
☒	☐ Dual use research of concern

Methods

n/a	Involved in the study
☒	☐ ChIP-seq
☒	☐ Flow cytometry
☒	☐ MRI-based neuroimaging

Antibodies

Antibodies used

PGT121, PGDM1400, VRC07-523LS

Validation

All detailed product informations on the respective bNAbs, PGDM1400, PGT121 and VRC07-523LS are available in the Investigator's Brochures which were submitted for this trial under IND 126807, including specifically Section 3, "Physical, Chemical and Pharmaceutical Properties." The IBs are available upon request from IAVI, the sponsor.

Human research participants

Policy information about studies involving human research participants

Population characteristics

Participants were eligible for the study across Groups if they did not have any clinically significant acute or chronic medical condition (besides HIV), such as chronic hepatitis B, active hepatitis C, significant psychiatric disorder, alcohol or substance use disorder, or chronic kidney or liver disease and if they had a body mass index >18 and <35. Sexually active participants had to be willing to use contraception for 3 months following IP administration, and could not be pregnant or breastfeeding. Participants were eligible for Group 1 and 2 if they were also 18-50 years of age and at low risk for HIV infection and willing to maintain low-risk behavior. Participants with HIV (Group 3) were eligible if they were 18-65 years of age, had CD4 ≥ 300 cells/µl, no history of AIDS-defining illness within the previous 5 years and if they were not on antiretroviral therapy for > 6 months with detectable HIV-1 RNA levels between 1,000 and 100,000 copies/ml and (after appropriate counseling) willing to defer ART treatment for at least 56 days after administration of IP. All participants gave written informed consent and successfully completed an assessment of understanding before the initiation of study procedures.

Recruitment

Adult male and female participants were recruited through in-clinic referrals, information presented to community organizations, hospitals, colleges, other institutions and/or advertisements to the general public or from existing cohorts. The information distributed contained information about the trial and contact information for the site. Study staff members also attended events related to public health, HIV/AIDS, sexual health, and other topics as appropriate. Because participants were recruited from North America, HIV sequence diversity was biased towards clade B viruses that may be less susceptible to PGDM1400 compared to other clades. For our HIV-negative population, recruits were from the Boston area and more frequently caucasian compared to the general population of people living with HIV; this may limit generalizability to other populations.

Ethics oversight

The protocol was approved by the BIDMC Institutional Review Board, the OIC Institutional Review Board, and the HART Committee for the Protection of Human Subjects.

Note that full information on the approval of the study protocol must also be provided in the manuscript.

Clinical data

Policy information about clinical studies

All manuscripts should comply with the ICMJE guidelines for publication of clinical research and a completed CONSORT checklist must be included with all submissions.

Clinical trial registration

NCT03205917

Study protocol	All protocol versions are provided as an appendix to the manuscript.
Data collection	62 volunteers without and with HIV were screened for study participation and 33 were found to be ineligible or excluded for other reasons (Extended Data Fig 1).The first participant was enrolled on November 27, 2017, and the last participant completed the study on April 20, 2020.
Outcomes	The primary endpoints were for safety and tolerability: (1) proportion of participants with moderate or greater reactogenicity (e.g., solicited AEs) for 3 days following IV infusion of PGDM1400 alone, a combination of PGDM1400 and PGT121 bNAbs, and a combination of PGDM1400 and PGT121 and VRC07-523LS, (2) proportion of participants with moderate or greater and/or PGDM1400 and PGT121 and VRC07-523LS bNAb-related unsolicited AEs, including safety laboratory (biochemical, hematological) parameters, following IV infusion of PGDM1400 and/or PGT121 and/or VRC07-523LS for the first 56 days post administration of IP, and (3) proportion of participants with PGDM1400 and/or PGT121 and/or VRC07-523LS -related SAEs throughout the study period. The primary endpoints, for pharmacokinetics, were elimination half-life (t1/2), clearance (CL/F), volume of distribution (Vz/F), area under the concentration decay curve (AUC), and impact of HIV RNA levels on PGDM1400 and/or PGT121 and/or VRC07-523LS disposition (elimination half-life (t1/2), clearance (CL/F), volume of distribution (Vz/F), and total exposure. The primary endpoint for antiviral activity among viremic participants with HIV was the change in plasma HIV-1 RNA levels from baseline (mean of pre-entry and entry values). The secondary endpoints were change in CD4+ T cell count and frequency compared to baseline as measured by single platform flow cytometry, and development of HIV-1 sequence variations in epitopes known to result in reduced PGDM1400 and/or PGT121 and/or VRC07-523LS neutralization susceptibility. The primary endpoints for safety, tolerability and pharmacokinetics were changed in Protocol Version 4.0 to include the VRC07-523LS mAb, for the subgroup 3A.
